# Diagnosis and mitigation of the systemic impact of genome reduction in *Escherichia coli* DGF-298

**DOI:** 10.1128/mbio.00873-24

**Published:** 2024-08-29

**Authors:** Antoine Champie, Jean-Christophe Lachance, Anand Sastry, Dominick Matteau, Colton J. Lloyd, Frédéric Grenier, Cameron R. Lamoureux, Simon Jeanneau, Adam M. Feist, Pierre-Étienne Jacques, Bernhard O. Palsson, Sébastien Rodrigue

**Affiliations:** 1 Département de Biologie, Université de Sherbrooke, Sherbrooke, Quebec, Canada; 2 Department of Bioengineering, University of California, San Diego, La Jolla, California, USA; 3 Novo Nordisk Foundation Center for Biosustainability, Technical University of Denmark, Kongens, Lyngby, Denmark; 4 Bioinformatics and Systems Biology Program, University of California, San Diego, La Jolla, California, USA; 5 Department of Pediatrics, University of California, San Diego, La Jolla, California, USA; Korea Advanced Institute of Science and Technology, Daejeon, South Korea

**Keywords:** *Escherichia coli*, systems biology, genome-scale metabolic model, simplified genome, oxidative stress

## Abstract

Microorganisms with simplified genomes represent interesting cell chassis for systems and synthetic biology. However, genome reduction can lead to undesired traits, such as decreased growth rate and metabolic imbalances. To investigate the impact of genome reduction on *Escherichia coli* strain DGF-298, a strain in which ~ 36% of the genome has been removed, we reconstructed a strain-specific metabolic model (*i*AC1061), investigated the regulation of gene expression using iModulon-based transcriptome analysis, and performed adaptive laboratory evolution to let the strain correct potential imbalances that arose during its simplification. The model notably predicted that the removal of all three key pathways for glycolaldehyde disposal in this microorganism would lead to a metabolic bottleneck through folate starvation. Glycolaldehyde is also known to cause self-generation of reactive oxygen species, as evidenced by the increased expression of oxidative stress resistance genes in the SoxS iModulon. The reintroduction of the *aldA* gene, responsible for one native glycolaldehyde disposal route, alleviated the constitutive oxidative stress response. Our results suggest that systems-level approaches and adaptive laboratory evolution have additive benefits when trying to repair and optimize genome-engineered strains.

**IMPORTANCE:**

Genomic streamlining can be employed in model organisms to reduce complexity and enhance strain predictability. One of the most striking examples is the bacterial strain *Escherichia coli* DGF-298, notable for having over one-third of its genome deleted. However, such extensive genome modifications raise the question of how similar this simplified cell remains when compared with its parent, and what are the possible unintended consequences of this simplification. In this study, we used metabolic modeling along with iModulon-based transcriptomic analysis in different growth conditions to assess the impact of genome reduction on metabolism and gene regulation. We observed little impact of genomic reduction on the regulatory network of *E. coli* DGF-298 and identified a potential metabolic bottleneck leading to the constitutive activity of the SoxS iModulon. We then leveraged the model's predictions to successfully restore SoxS activity to the basal level.

## INTRODUCTION

The advent of synthetic biology and genome engineering has fueled a new era in the development of microbial strains with tailored properties, such as enhanced bioproduction capabilities, improved genomic stability, or minimized biosafety risks (1–3). In that context, genome reduction, a strategy that involves the elimination of non-essential genes, has been suggested as a means to create streamlined, more controllable and predictable cell factories that are easier to engineer. Non-essential genes can be identified using various approaches, including comparative genomics (4, 5), targeted gene knockout (6), and transposon mutagenesis followed by sequencing (TIS) (7, 8). Two different strategies can then be used to perform genome reduction: top-down or bottom-up. Top-down implies the removal or inactivation of specific genes or regions from an organism’s genome to create a reduced version, whereas bottom-up involves building from scratch by synthesizing and/or assembling a restricted set of genes necessary for basic cellular processes and other desired features (9).

Since the early 2000s, genome reduction projects have been undertaken in several model bacterial species, such as *Bacillus subtilis*, *Mycoplasma mycoides*, and the laboratory workhorse *Escherichia coli*. One of the most impressive achievements was the creation of *M. mycoides* JCVI-syn3.0, the first working approximation of a minimal cell (9). This artificial cell harbors a genome of only 531 kb, representing a reduction of ~50% compared with the original *M. mycoides* subsp. *Capri* genome (1.08 Mb). However, syn3.0 displayed important morphological and growth defects compared with its parent strain, which were later resolved by the reintroduction of 19 genes initially discarded from the design (10). Another striking example is the recently published *B. subtilis* strain MGP254 (11). This strain was engineered using homologous recombination to remove 1.48 Mb from the *B. subtilis* strain 168, corresponding to a genome reduction of approximately 25%. Although MGP254 exhibited emergent properties, such as a decreased mutation rate, it also had unintended shortcomings, including the loss of natural competence. Multiple parallel projects in *E. coli* have also been undertaken, with genome reduction ranging from 6.8% (CDΔ3456) (12) to 39% of the natural K-12 genome (Δ33a). This latter strain lost more than 1.8 Mb of its genome but also displayed altered cell yields and unexplained oxidative stress sensitivity (13). In the reduced strain MGF-01, approximately 22% (1.03 Mb) of the *E. coli* W3110 genome was removed, targeting insertion sequences, transporter genes, toxin-antitoxin pairs, and other non-essential genes (14). This strain was further reduced a few years later, creating DGF-298, a genome-reduced *E. coli* harboring a chromosome of only 2.99 Mb, which corresponds to a reduction of approximately 36% (15). This strain is of particular interest because of its ability to grow in minimal medium at rates similar to its parent, *E. coli* W3110, despite being one of the most genome-reduced *E. coli* strain available to date. During the reduction process of DGF-298, two deficient genes, *ilvG* and *pyrE*, were replaced with functional alleles sourced from the *E. coli* B strains line (15). The authors also reintroduced the same two alleles in the W3110 to generate W3110S and allow more consistent comparisons with DGF-298.

Although important genome reductions have been performed in many model organisms, most projects have been conducted without the use of integrative or predictive tools to guide reduction efforts. Consequently, genome reduction often results in unintended consequences, such as impaired growth rate and metabolic capabilities, altered morphological phenotypes (9), enhanced sensitivity to external stresses (13, 16), and the emergence of novel regulatory networks (17). There is thus a need to better predict and evaluate the impact of genome reduction on global cell functioning to more reliably produce simplified cells desirable characteristics . Understanding how the metabolic and transcriptional regulatory networks are altered in currently available genome-reduced strains might provide important insights for future projects. Adaptive laboratory evolution (ALE), a method where organisms are evolved under controlled laboratory conditions to select desired traits, and genome-scale metabolic models (GEMs), a process using a computational representations of an organism’s metabolism to predict cellular behavior, are interesting tools that can be used to identify and rectify metabolic imbalances and improve strain characteristics (18–21). ALE involves the continuous culturing of an organism over multiples generations with the aim to create and select mutations beneficial to growth in the selected conditions. This method has been widely used to generate phenotypes of interest, such as tolerance to certain stresses (22) or fast growth in specific medium (23). GEMs are computational frameworks that integrate the entirety of an organism’s known metabolic pathways. They are used to describe, analyze, and predict the metabolic behavior of organisms under specified conditions. GEMs have been used to guide the design of genetically engineered organisms with diverse objectives, such as aromatic polyester production optimization (24), or to help understand the metabolic dependencies of diseases, such as cancer (25). The most up-to-date GEM available for *E. coli* is *i*ML1515 (26), which is composed of 1,515 genes, 2,719 metabolic reactions, and 1,839 unique metabolites. It is one of the most complete GEM currently available and has an impressive 93% accuracy in predicting gene essentiality in minimal medium (26).

In this study, we generated a strain-specific GEM for the genome-reduced *E. coli* DGF-298 (15) to evaluate the state of its metabolic networks after reduction. We then combined experimental data, transcriptome profiling using iModulon analysis, ALE, and GEM-guided engineering to assess the impact of reduction on transcriptome, explore ways to troubleshoot systemic issues caused by genome reduction, and create an improved engineered strain for potential biotechnological applications.

## RESULTS

### Generation of the *E. coli* DGF-298 metabolic model

**Fig 1:**
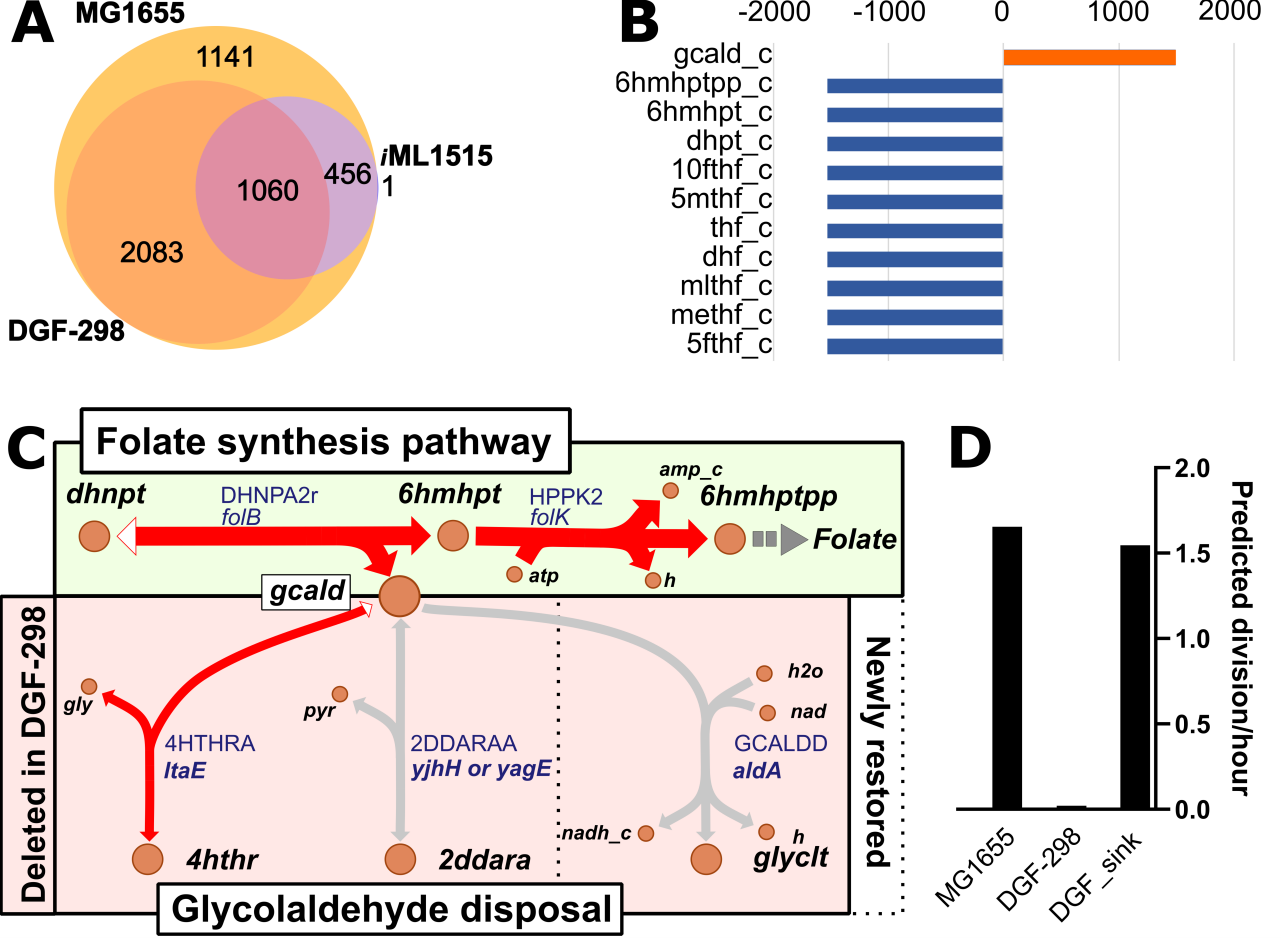
Construction of a metabolic model for DGF-298. (**A**) Comparative analysis of shared genes among *E. coli* K-12 MG1655 strain, DGF-298 strain, and the *i*ML1515 model. The single occurrence present in the models but absent from MG1655 is *s0001*, a proxy for spontaneous reactions. (**B**) Non-zero shadow prices in the *i*ML1515 model after eliminating 456 absent genes from DGF-298. (**C**) Visual representation of the glycolaldehyde (gcald) production segment in the folate synthesis pathway along with the three paths available in wild-type *E. coli* for glycolaldehyde disposal. Red arrows signify flux in wild-type metabolism in simulated conditions (EZ-Rich glucose-defined medium). For complete folate synthesis pathway, see Fig. S2 and S3. White arrowheads highlight unfavored directions in bidirectional reactions. Metabolite names are shown in black, whereas reaction and associated gene names are displayed in blue. For complete metabolite names, refer to the BiGG database (31). (**D**) Predicted growth rate of MG1655, DGF-298, and DGF-298 with glycolaldehyde sink pseudo-reaction added in simulated EZ-Rich glucose medium.

To investigate the metabolic capabilities of *E. coli* DGF-298 (15), a strain-specific GEM was first generated using the COBRApy (27) library. The recently published and high-quality *E. coli* MG1655 GEM, *i*ML1515 (26), was used as a foundation for the *i*AC1061 GEM. Although DGF-298 was derived from the *E. coli* K-12 W3110 strain, for which a strain-specific GEM exists (28), MG1655 and W3110 are among the most similar *E. coli* strains both in terms of genome sequence (29) and general gene expression correlation (28). This led us to favor the use of the more recent and highly characterized *i*ML1515 model instead of the W3110 model (28). To construct the DGF-298 model, the 456 genes present in *i*ML1515 but identified as missing or mutated in DGF-298 (Table S1) were removed (Fig. 1A). Some genes in DGF-298 are truncated or contain frameshift mutations, which are likely to result in non-functional proteins. The initial DGF-298 model included 1,060 genes (Table S1), 2,365 reactions, and 1,742 metabolites. Curiously, the production of biomass was not possible in the model after gene removal in simulated EZ-Rich glucose medium (Fig. S1), although the DGF-298 strain can grow in this medium. To identify the source of this issue, the shadow prices of the flux balance analysis (FBA) solution were examined. Shadow prices, an inherent property of linear optimization problems, help identify metabolites that most affect the objective functions, for example biomass objective function (30). Here, a negative shadow price value indicates that a simulated metabolite injection would increase growth rate, whereas a positive value signifies that a simulated injection would cause a metabolic burden and lead to reduced growth rate. In the initial DGF-298 GEM, 10 metabolites had negative shadow prices and were all related to folate metabolism (Fig. 1B). The only metabolite with a highly positive value was glycolaldehyde. All other metabolites had values equal or close (<10^−13^) to zero (Table S1). This suggested an inability to produce folate and its precursors and a potential issue with glycolaldehyde disposal.

Using the *i*ML1515 model as a database for reactions, the COBRApy gap-filling algorithm (27) was applied to find reactions restoring the growth phenotype in the DGF-298 model. Three reactions (2DDARAA, 4HTHRA, nd GCALDD) were identified, revealing that glycolaldehyde is a necessary by-product of folate metabolism in DGF-298 (Fig. 1C; Fig. S2 and S3). This is confirmed by the fact that adding the pseudo-reaction (SK_gcald_c), which freely consumes glycolaldehyde, was sufficient to make the model functional (Fig. 1D; Fig. S1 and S4B). Indeed, the absence of escape routes due to the removal of the three available pathways in DGF-298 — 1) 4-hydroxy-L-threonine aldolization via *ltaE* (b0870), 2) 2-dehydro-3-deoxy-D-arabinonate aldolization via *yjhH*/*yjhG* (b4298/b4297) or *yagE*/*yagF* (b0268/b0269), and 3) glycolaldehyde dehydrogenation via *aldA* (b1415) — caused a metabolic dead-end. This prevented the model from providing an optimal solution (no-growth phenotype prediction), which is inconsistent with the observed growth of the DGF-298 strain. This initial identification indicates a potential cellular issue due to the accumulation of glycolaldehyde and points towards an alternate, unannotated escape route for this reactive metabolite. Expanding the gap-filling solutions with all reactions from the BiGG database (31, 32) identified no potential match for a glycolaldehyde disposal reaction available in DGF-298.

To emulate the unknown glycolaldehyde sink allowing DGF-298 growth *in vivo*, the SK_gcald_c pseudo reaction was temporarily added in the DGF-298 GEM. To validate the prediction accuracy of the newly developed DGF-298 GEM, *in silico* knock-out of all individual genes in the model and in iML1515 were performed in parallel. Deletions causing a growth rate prediction <0.1 division/h were considered essential. This list of predicted essential genes was compared with transposon mutagenesis data obtained in the DGF-298 strain in EZ-Rich glucose medium (33), revealing an 82% prediction accuracy for both models (Table S2). More precisely, the *i*AC1061 model predicted the correct phenotype for 856 genes of the 1,030 comparable genes. This decrease in relative accuracy of the iAC1061 model’s prediction of the reduced strain when compared with the iML1515 prediction of the wild-type strain (93.4%) is expected because of the extensive modifications undertaken on the strain. Most notably, we expect to see the emergence of new essential genes resulting from the removal of genes in a synthetic lethal relationship, which were previously compensated by alternative pathways or interactions not considered in the model. This is supported by the fact that genes that are seen as essential via transposon mutagenesis but wrongly predicted non-essential by the model account for most (~84%) of the genes that are wrongly predicted by the model. This validates the general predictive capacity of the model and highlights areas of limited knowledge.

### Adaptive laboratory evolution enhances growth in genome-reduced strain

**Fig 2:**
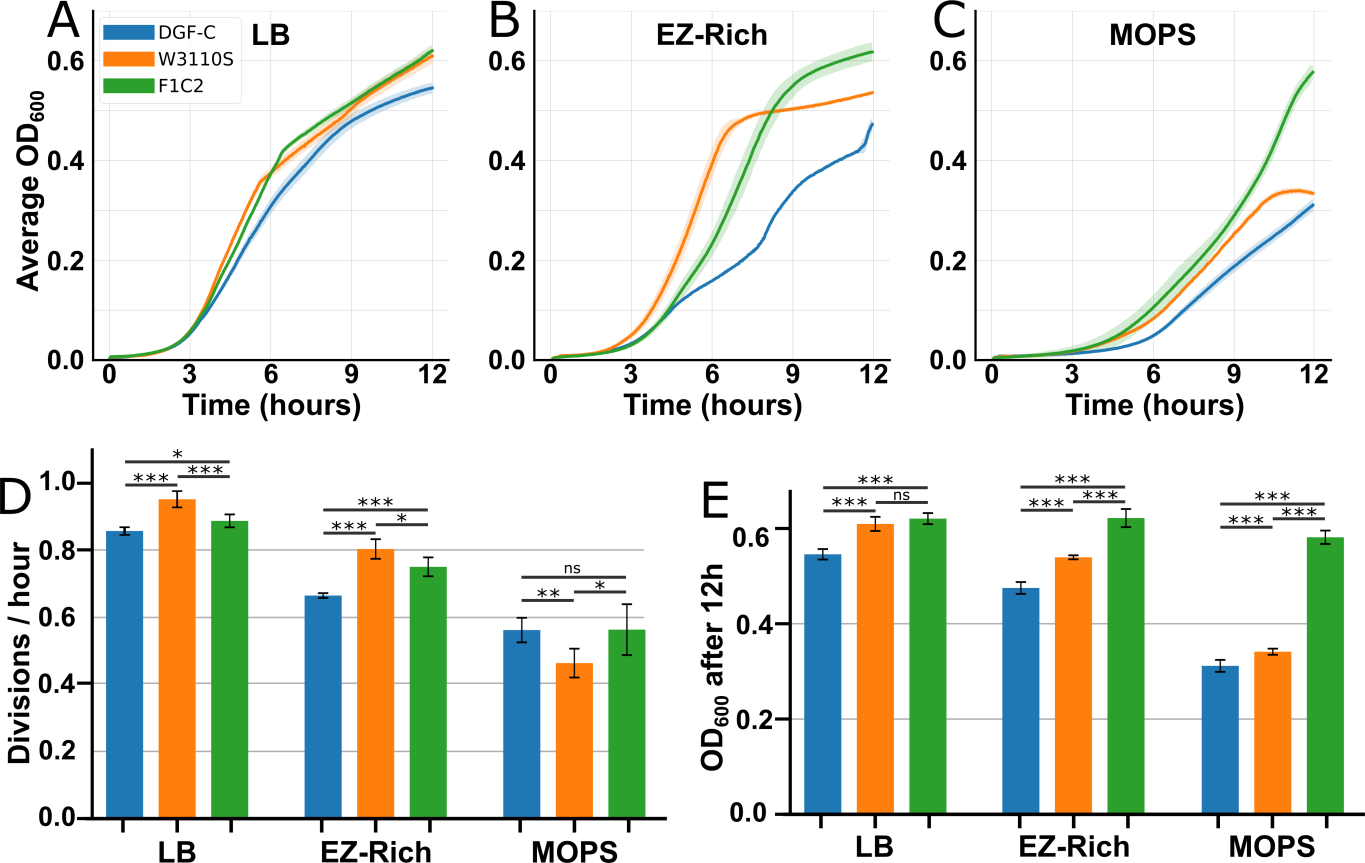
Growth profiles of the evolved strain F1C2 in various culture media. (**A–C**) Growth curves of wild-type *E. coli* (W3110S, orange), reduced strain derived from DGF-298 (DGF-C, blue), and evolved reduced strain (F1C2, green) in LB (**A**), EZ-Rich + glucose 0.2% (**B**), and MOPS + 0.2% glucose (**C**). Each data point represents the average optical density at 600 nm (OD_600_) of six technical replicates grown at 30°C with agitation in a plate reader over 12 h. Standard deviations are represented by colored shadings. (**D and E**) Calculated growth rates (**D**) and maximum OD_600_ after 12 h (**E**) of curves shown in A–C. Bars and whiskers show standard deviation calculated over six replicates. Data were compared with student’s *t*-test. Raw data are available in Table S3. *P < 0.05, **P < 0.01, ***P < 0.001; ns, not significant.

Metabolic models predict the optimal cellular state in a given environment. However, when conditions or genotypes change, the observed phenotype (e.g., growth rate) may differ from predicted values as cells likely need to acquire mutations that will adapt their regulation and metabolism to reach the predicted optimal state (34). For a more accurate comparison with the model and to minimize potential growth disruptions caused by selection genes, we scarlessly excised the residual *cat-sacB* selection cassette from the DGF-298 strain’s genome. This modification resulted in the creation of the DGF-C (DGF-Clean) strain. Then, to guide the genome-reduced strain DGF-C toward its optimal state and give it a chance to alleviate unintended metabolic imbalances and other regulatory defects that may have accumulated during genome reduction, ALE (34) was conducted over 28 daily passages (~180 generations) in MOPS minimal medium with glucose. The MP6 mutagenic plasmid (35) was transformed in DGF-C before the ALE experiments to accelerate the evolution of the strain. After the last passage, 12 clones were isolated, and their growth was tested in three usual laboratory media. One of the isolated strains, F1C2, exhibited improved growth rates and reached higher densities after 12 h than the reduced strain in most tested conditions (Fig. 2; Table S3). Surprisingly, the effect of evolution on the growth rate is more apparent in both rich media tested, suggesting that this improvement is not an adaptation specific to the evolution medium. Meanwhile, the improvement in cell density after 12 h is most noticeable in MOPS medium, offering a possible explanation why this mutant was more competitive in our ALE experiment.

To identify mutations that arose from the ALE experiment, the F1C2 clone was subjected to whole genome sequencing. The resulting genome sequences were added to the ALE database (ALEdb) (36), revealing 44 mutations (Table S4). Among these, three were located in intergenic regions but not in any known regulatory sequence, one was located in the *nadR* gene, responsible for repressing NAD transport and biosynthesis genes, and the other 40 were located within coding sequences. An in-depth literature review could not obviously explain the contribution of these mutations to the improved phenotype of the F1C2 clone. To assess the impact of those mutations at the functional level, we performed Gene Ontology overrepresentation tests on all mutated genes against all *E. coli* genes using the PantherDB Tool (http://pantherdb.org/, version 17.0). This test aims to find if any ontology (function) is enriched in the mutated population of genes over a random population of the same size. This analysis showed no statistically significant overrepresentation of any biological process or molecular function in the mutated genes. As a control to monitor the potential effects of adaptation to MOPS medium, we also subjected the parent strain, W3110S, to the same ALE experiment. An evolved clone, E1C2, was isolated from this evolution and sequenced. E1C2 harbored 107 mutations with no statistical overrepresentation of biological function or molecular function (see Table S4 for a list of mutations found in E1C2).

### DGF-298 genomic reduction led to a constitutive activation of the SoxS oxidative stress response iModulon

To evaluate the impact of genome reduction on transcription, we then assessed F1C2 transcription profiles through RNA sequencing (RNA-seq) under 11 different conditions in M9 minimal defined medium. This enabled comparison with the PRECISE database (37), a compendium of transcriptomic data generated in numerous conditions using *E. coli* MG1655. The 11 conditions were chosen to maximize the difference in gene expression between conditions using various stresses. This enables the observation of groups of genes whose regulation varies together across all conditions. Those groups are called independently modulated (iModulons) (37) and can be observed at high resolution using a practical number of transcriptomic conditions.

Interestingly, the impact of ALE on the transcription of the evolved strains E1C2 and F1C2 compared with their respective parents, W3110S and DGF-C, was minimal. The only differences found between the non-reduced strains were an increase in the activity of the GadXW (acid resistance) and gcvB (amino acid transport) iModulons (Fig. S7). Direct comparison of E1C2 against MG1655 displayed no additional unexpected differences in iModulon activity levels (Fig. S8). As for the reduced strain, only a decrease in the Translation iModulon was observed (Fig. S14).

**Fig 3:**
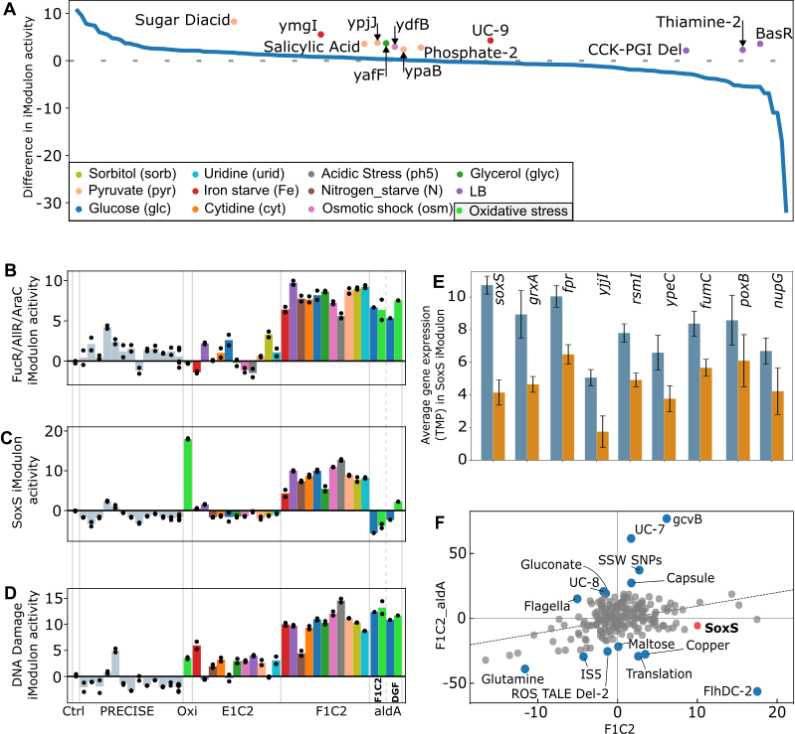
Comparative analysis of iModulon activity across different strains and conditions. (**A**) Difference in iModulon activity between E1C2 and F1C2 strains averaged across 11 growth conditions, with dots representing conditions with extreme difference (>2.5 standard deviations) from the average. Colors are constant across A–F. Oxidative stress condition (light green) only applies to sub-panels D–F. Values are available in Table S6. (**D–F**) Average activity levels of FucR/AllR/AraC (**D**), SoxS (**E**), and DNA damage (**F**) iModulons in various strains and conditions, including 15 conditions from PRECISE (32). In order: M9 glucose control (Ctrl), 13 samples from the core database to show expectable activity variation in MG1655 samples and the oxidative stress condition (Oxi), 11 conditions in E1C2 and F1C2, two conditions in F1C2-aldA and DGF-aldA. Complete activity levels for every iModulon can be found in File EV1. Black dots indicate the activity level of individual replicates in each condition. (**G**) Average expression of the nine most differently expressed genes of the SoxS iModulon between E1C2 (light blue) and F1C2 (light orange). Gene expression is given as the transcripts per million mapped reads (TPM). Error bars indicate standard deviation calculated over the 11 tested conditions. (**H**) Differential iModulon analysis (DIMA) plot of F1C2 and F1C2-aldA in M9+0.2% glucose. iModulons with highly differential activity are highlighted in blue, whereas the SoxS iModulon is highlighted in red. Dashed line shows the equivalent activity level in the two strains.

Comparing iModulons activity of the F1C2 strain against the E1C2 strain in all 11 tested conditions revealed that only a few iModulons display consistent differences in activity across all tested media (Fig. 3A). This suggests that most of the regulation has been kept consistent during genome reduction and laboratory evolution. Detailed analysis of the most positively and most negatively differentially activated iModulons revealed interesting patterns (Fig. S9; Table S6). For instance, the FucR/AllR/AraC iModulon showed significant increased activity in F1C2, which is expected because all three repressors controlling this iModulon (FucR, AllR*,* and AraC) have been removed during reduction (Fig. 3B). Three of the four genes regulated by the *nadR* repressor, *nadA*, *pnuC*, and *nadB* are overexpressed, a ~3-fold increase in the F1C2 strain, and contribute to the higher activity of the Leu_Val_Ile iModulon. By contrast, both the SoxS (Fig. 3C) and DNA damage (Fig. 3D) iModulons exhibited high activity levels across all F1C2 conditions, without, to our knowledge, any obvious regulatory deletion in DGF-298 explaining this difference. One initial hypothesis was the slight (eight amino acids) shortening of the *soxR* gene during the reduction of the DGF-298 strain that was performed to remove the overlapping *ryjA* small RNA, but this region appears to bear no functional domain in the SoxR protein (https://www.uniprot.org/uniprotkb/P0ACS2/entry). *ryjA* itself is mostly expressed in stationary phase and appears to be repressor of the *tig* gene (38), a protein chaperone. Because SoxR is an oxidative stress sensor and an activator of *soxS,* any loss of function should lead to a decrease of *soxS* expression (39, 40), the opposite of what is observed in the strain. This suggests an alternative cause for activation in the genome-reduced strain, such as a constitutive level of oxidative stress and subsequent DNA damage caused by the accumulation of reactive oxygen species (ROS) in the cell. This is supported by the fact that both iModulons showed similar activation patterns with MG1655 grown in oxidative conditions (Fig. 3C and D). As expected, expression levels of individual genes of the SoxS and DNA damage iModulons showed the same tendency (Fig. 3E; Fig. S10; Table S7).

The metabolic model previously identified that the removal of three key pathways may lead to the accumulation of glycolaldehyde in the cells (Fig. 1C), which has been shown to produce oxidative stress in other model cells (41, 42) and activate the SoxRS regulon (43). This regulon includes two hallmarks of oxidative stress resistance: the superoxide dismutase (*sodA*) and the oxidative stress-resistant (44) fumarate hydratase (*fumC*). Overexpression of both of these genes is observed in the reduced F1C2 strain (Fig. S11), suggesting the presence of a basal level of oxidative stress in this strain, which might be caused by the predicted accumulation of glycolaldehyde. It is to be noted that one of the mutations selected in the F1C2 clone is in the *fumC* gene, though not in any known protein domain.

**Fig 4:**
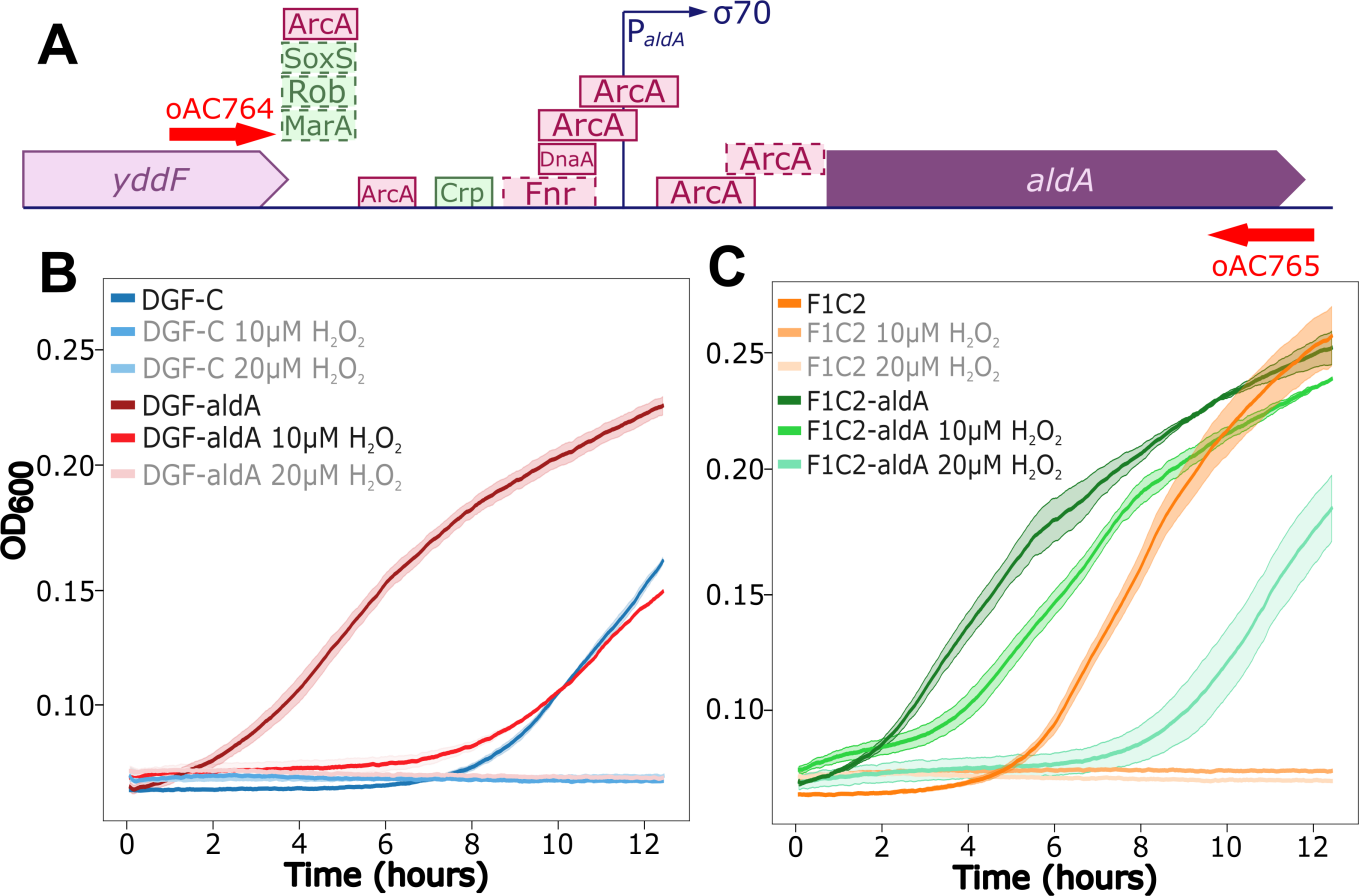
*aldA* complementation in genome-reduced strains. (**A**) Representation of the genomic locus amplified by PCR for *aldA* complementation. Selected primers (red arrows) were designed to include all known regulatory regions located immediately upstream of *aldA*. Green and fuchsia boxes indicate activator and repressor binding sites, respectively. Dashed outline indicates lower quality evidence for the presence of the binding site. (**B and C**) Growth data of strains DGF-C and DGF-aldA (**B**) as well as F1C2 and F1C2-aldA (**C**) in MOPS + 0.2% glucose supplemented with different concentrations of H_2_O_2_. The gray text in the legend represents conditions where no growth was observed. Each data point represents the average optical density at 600 nm (OD_600_) of two technical replicates grown at 30°C with agitation in a plate reader over 12 h. Standard deviations are represented by colored shadings.

This basal level of oxidative stress could explain the inability of the DGF-298 and F1C2 strains to grow under moderate (<10 µM) concentrations of hydrogen peroxide (Fig. 4B and C). Similar to the addition of exogenous H_2_O_2_, the accumulation of glycolaldehyde and glyoxal results in the production of ROS that are toxic to cells (45). The endogenous accumulation of glycolaldehyde through the production of folate in DGF-298 might explain why the cell cannot withstand higher amounts of H_2_O_2_. This is a common phenotype in genome-reduced strains (11, 13), but so far, no direct cause has been pinpointed.

### Reintroduction of *aldA* resolves constitutive SoxS iModulon activation

In an attempt to repair the metabolism of the F1C2 strain, we sought to remedy its constitutive oxidative stress issue and restore its ROS tolerance using the GEM as a diagnostic tool to help pinpoint the issue and propose a solution. Because the GEM highlighted that the three main routes for glycolaldehyde disposal were removed during the creation of DGF-298 and that glycolaldehyde accumulation is a likely culprit of the consistent oxidative stress of the cells (Fig. 1B and C), we decided to reintroduce a gene responsible for one of the native routes for glycolaldehyde treatment. Among the three pathways considered, 1: *aldA*, 2: *ltaE*, and 3: *yagE/yagF* or *yjhH/yjhG*, only *aldA* and *ltaE* were amenable to single-gene reintroduction, which was deemed more practical. The choice between *aldA* and *ltaE* was further refined based on their enzymatic specificities. *aldA* encodes for an enzyme with specificity for aldehyde dehydrogenation (46), making it a more straightforward candidate for evaluating the impact on the pathway of interest. Conversely, *ltaE* encodes for a low-specificity L-threonine aldolase, which may introduce additional variables due to its broader substrate range. Nevertheless, *in silico* reintroduction of either one of the three identified glycolaldehyde disposal pathways restored the growth to levels similar to the wild-type (Fig. 2; Fig. S1) The *aldA* gene was PCR amplified along with its proximal upstream regulatory elements(47) (Fig. 4A) from the genome of *E. coli* W3110S and reintroduced in the genome of both DGF-C and F1C2 strains through the use of the Tn7 transposase (48). This transposase inserted a mini transposon carrying the amplified *aldA* gene in the genome at its unique insertion site, downstream of the *glmS* gene, creating the DGF-aldA and F1C2-aldA strains respectively. In parallel, the *aldA* gene was also reintroduced in the DGF-298 GEM instead of the sink pseudoreaction (SK_gcald_c), yielding a functional model corresponding to the DGF-aldA strain and named *i*AC1061.

RNA-seq was performed on the complemented strains and showed that the activity of the SoxS iModulon was significantly reduced compared with the F1C2 strain (Fig. 3C and F), which was also the case for associated hallmark genes (Fig. S11; Table S7). Intriguingly, this shift in activity was not observed for the DNA damage iModulon (Fig. 3D). The *aldA* reintroduction also appeared to have influenced the activity levels of some non-oxidative stress-related iModulons (Fig. 3F). Because *aldA* has no known regulatory effect, this activity reduction is most probably mediated by a decrease in self-generated oxidative stress. This is supported by the fact that a slightly improved ROS tolerance was observed in minimal medium supplemented with low concentrations of H_2_O_2_, both for the DGF-298 and the evolved F1C2 strains (Fig. 4B and C). Indeed, no growth could be detected for non-complemented strains when as low as 10 µM of H_2_O_2_ was added to the medium. In contrast, both DGF-298 and F1C2 complemented with *aldA* showed growth at concentrations up to 20 µM of H_2_O_2_. However, the observed resistance improvement is limited and remains below the wild-type H_2_O_2_ resistance (>100 µM, Fig. S12), suggesting that complementary mechanisms involved in ROS tolerance were lost during genome reduction. No significant improvement of growth rate or OD_600_ after 12 h could be observed in either one of the two reduced strains after *aldA* reintroduction (Fig. S13).

## DISCUSSION

In this study, we successfully generated a GEM (*i*AC1061) for *E. coli* DGF-298, a strain with a significantly reduced genome, using the high-quality *E. coli* MG1655 GEM *i*ML1515 as a foundation. Comprised of 1,061 genes, 2,365 reactions, and 1,742 metabolites, we validated the *i*AC1061 essentiality prediction capability using TIS data and exploited it to investigate the impact of genome reduction on DGF-298 metabolism. To guide the genome-reduced strain DGF-298 toward the optimal state predicted by the model and to mitigate any unintended metabolic or regulatory imbalances incurred during genomic reduction, this strain was evolved in a defined minimal medium. This led to the isolation of a clone (F1C2) with an overall improvement in its growth rate and OD_600_ after 12 h in common laboratory growth media (Fig. 2D and E). Using this newly evolved strain we combined GEM predictions with iModulon-assisted transcriptional analysis to identify an unintended metabolic dead end, causing a suspected accumulation of glycolaldehyde and generation of oxidative stress. This led to a targeted improvement of this metabolic dead-end through specific gene reintroduction, and a return to basal activity level of oxidative stress resistance iModulon SoxS.

This investigation of the metabolism of a reduced strain has surfaced many interesting questions about the influence of genomic reduction on biological systems that could be explored by further research efforts. Despite a significant 30% gene count reduction from *i*ML1515 to *i*AC1061 (Fig. 1A), the relative decreases in reactions and metabolites counts are far less pronounced, at 13% and 8%, respectively. This discrepancy suggests that many of the excised genes in the model were functionally redundant, thus preserving a metabolic profile more similar to a wild-type bacterium than the heavy reduction might initially imply. The reduced accuracy in gene essentiality prediction of the new model, when compared with the original, might stem from the fact that fewer TIS data sets have been generated for this reduced strain (33). Alternatively, this reduced accuracy could arise from synthetic relations with non-metabolic genes that are unaccounted for in the *i*AC1061 model and that have been removed *in vivo*. This presents a compelling potential explanation and an exciting subject for future investigation. Another interesting avenue that could be explored is the optimization of the biomass function of the model. This critical element of the model is designed to mimic the consumption of essential cellular constituents necessary for cell division (49). This function has been established for wild-type *E. coli*, and it might have been affected by genome reduction, leading to a lower confidence in fitness simulations. Additionally, despite the strain’s overall simulated fitness aligning closely with *i*ML1515 (Fig. S1), it was observed that deletion of certain genes (*tcyN*, *acrE*, *tdcC*) results in minor (<2%) reductions of predicted fitness. Studying the effects of their reintroduction could prove insightful. The question of whether reduced organisms retain traits of their parent strains or should be regarded as novel organisms for engineering purposes remains open. However, the relatively minor difference in most iModulon’s activity level observed under a variety of stress conditions suggests that reduced organisms may indeed exhibit behaviors akin to their parent strains.

Most large-scale *E. coli* genome reduction projects (3, 12, 14, 17, 50) pre-dated the emergence of modern GEMs (51), relying on other, less integrative, approaches to gene essentiality predictions. According to TIS data, a significant number of non-essential genes persist in this reduced strain (33), suggesting that a sub 2.0-Mbp *E. coli* genome is possible. Given the increasing prevalence of GEMs (52) and the introduction of more sophisticated genome-reduction design strategies (11), it may be beneficial to reevaluate reduction efforts using GEMs as guiding frameworks. This approach could enable further simplification while preventing certain pitfalls associated with genome reduction, rather than *post hoc* troubleshooting. Although evolution alone can bring improvements to weakened cells, in our study, it only improved environmental H_2_O_2_ resistance when combined with model-driven gene reintroduction (Fig. 4B and C). Upcoming advanced models, such as the *E. coli* StressME (52), integrating expression costs and diverse stress resistance in addition to the current metabolic simulations could provide even more complete and accurate predictions.

The model highlighted the absence of key pathways involved in glycolaldehyde disposal (Fig. 1B and C; Fig. S3), which was consistent with experimental observations of increased expression of oxidative stress resistance genes (Fig. 3; Fig. S11). Notably, the SoxS iModulon, involved in the oxidative stress response, and the DNA damage iModulon were found to be consistently upregulated (Fig. 3; Fig. S9), suggesting a potential underlying issue. Guided by the model predictions (Fig. S1 and S4), reintroduction of the *aldA* gene, responsible for one of the native routes for glycolaldehyde disposal, led to a reduction in oxidative stress-related gene expression (Fig. 3; Fig. S11) and a slight increase in H_2_O_2_ tolerance (Fig. 4B and C; Fig. S15). However, the environmental ROS tolerance of the engineered strains remained markedly lower than the parental W3110S strain, suggesting that along the reduction process, other systems or mechanisms implicated in ROS tolerance have been affected. One hypothesis is that it is caused by the cumulated deletions of secondary mechanisms for oxidative stress resistance. One such example could be *adeD*, an adenine deaminase that can also function as a catalase (53), which was removed during reduction and could be an interesting target for genomic restoration. Interestingly, the primary genes hypothesized to contribute to the oxidative stress sensitivity of the reduced *E. coli* strain LD33a (13)—*aegA*, *ahpC*, *grxA*, *dsrA*, *gss*, and barA—are all still present in DGF-298, suggesting a different cause for oxidative stress sensitivity. As other reduced *E. coli* strains appear to suffer from similar issues as DGF-298, namely oxidative stress sensitivity (13), a decrease in fitness (54) or an increase in mutation rate (55) during genome reduction, a repeat of this approach in other reduced *E.coli* lines could help elucidate a common cause, and solution, for those undesirable phenotypes.

This study showcases the potential of combining genome-scale metabolic models, iModulon analysis, and adaptive laboratory evolution for addressing metabolic imbalances, and refining heavily engineered strain characteristics for use in potential biotechnological applications. Using a rational approach based on whole genome metabolic modeling, we identified a metabolic imbalance in DGF-298, which was further analyzed and corroborated with iModulon analysis. Nevertheless, despite utilizing current state-of-the-art models, a significant portion of the strain dynamics remains unaccounted for. It is in this gap where the knowledge-agnostic approach of ALE proves invaluable, offering corrective measures for discrepancies falling outside the model’s scope. The combination of these three techniques has enabled a multi-angled screening of the *E. coli* DGF-298-reduced strain, revealing its transcriptome and metabolic profile to be remarkably similar to that of its parent strain despite its highly reduced genome, while providing insight on how to fix the remaining metabolic issues.

## MATERIALS AND METHODS

### Biological methods

#### Bacterial strains and growth conditions

Bacterial strains used in this study and their corresponding genotypes are provided in Table S5. DGF-298 and W3110S strains were obtained from the Japan’s National BioResource Project (NBRP). W3110S is a variant of the W3110 strain in which the defective *ilvG* and *pyrE* genes present in all K-12 strains were replaced by functional versions of the *E. coli* B strain. This modification is also present in the DGF-298 strain (15). The DGF-C (DGF-Clean) strain was obtained by removing the *sacB-cat* cassette present in the DGF-298 strain by recombineering (see below). E1C2 and F1C2 clones were isolated from the evolution of DGF-C and W3110S strains, respectively (see below). All strains were grown at 37°C with agitation unless specified otherwise. LB medium was prepared from commercially available powder. M9 defined minimal medium, Neidhardt MOPS defined minimal medium, and EZ-Rich defined medium were prepared according to recipes available in the literature (56).

#### Generation of DGF-C by recombineering

Briefly, the 400-bp regions upstream and downstream of the *cat*/*sacB* cassette were PCR amplified from DGF-298 genomic DNA using primers oAC084/oAC085 and oAC086/oAC087 (Table S5), respectively. Those two fragments were then merged using fusion PCR (57) and subsequently transformed into a DGF-298 strain carrying pSIM6(58) following the usual recombineering protocol. Selection was performed on LB supplemented with 1M sucrose, and deletion was confirmed by PCR.

#### Evolution of DGF-C and W3110S strains

Before the ALE experiments, DGF-C and W3110S strains were transformed with the MP6 plasmid (35), which can be induced using arabinose to increase the mutation rate of the strain. Two cultures of W3110S (E1 and E2) and DGF-C (F1 and F2), both carrying the MP6 plasmid, were pre-cultured in MOPS with 25 mM glucose. The cultures were then diluted 1/100 in 200 mL of MOPS supplemented with 200 mM arabinose, to induce MP6, and left to grow overnight at 37°C with agitation at 225 rpm. Using 200 µL from each initial culture, two replicate tubes each containing 20 mL of MOPS 0.2% glucose were inoculated for each strain. Cultures were incubated at 37°C with agitation for 24 h. Daily passages using similar dilutions were performed for 28 days, totaling ~180 divisions. At the end of the experiment, 10 µL of each culture replicate was plated on MOPS agar plates from which individual clones were isolated (including the F1C2 and E1C2 clones). Mutation data for all isolated clones available on the ALEdb database (https://aledb.org/ale/project/78/).

#### DNA sequencing and mutation identification

Genomic sequencing of F1C2, E1C2, and F1C2-aldA clones was performed using the following protocol: Pellets were obtained by centrifugation of 2 mL of culture, and genomic DNA was extracted using PureLink Genomic DNA Kits (Invitrogen, Carlsbad, CA, USA) following the manufacturer’s protocol. The quality of extracted DNA was assessed with UV absorbance ratios using a nanodrop. The concentration of DNA was quantified using a Qubit ds-DNA high-sensitivity assay. Paired-end resequencing libraries were generated using a 300-cycle (150-bp paired end) kit from Illumina (San Diego, CA, USA) and then loaded on a on a Nextseq 1000 instrument with a loading concentration of 1.2 pico-Molar with a 1% PhiX spike (Illumina, San Diego, CA, USA) of total input DNA. Mutation identification was performed using a computational pipeline tool, as described in (36), based on breseq version 0.30.1(59) to map sequenced reads to the reference strain.

#### Growth profiling

Growth profiling was performed using a TECAN GENios Pro plate reader. The 96-well transparent plates were filled with 200 µL of assayed culture medium and were then inoculated using 2 µL of overnight culture. OD_600_ was measured every 90 s with shaking in between readings and temperature maintained at 30°C for 12 h. Doubling times were calculated using a previously established method (54). In short, blanked growth data were analyzed by measuring the density increase between every reading points separated by 30-min intervals and selecting the maximum average of four consecutive calculated growth rates.

#### RNA sequencing

Cells were grown with agitation at 37°C until early exponential phase in a series of different media (Table S5). Cells were then pelleted by centrifugation at 4°C and resuspended in 3 mL of RNA-protect (QIAGEN). RNA was extracted using the Zymo Quick-RNA Fungal/Bacterial Microprep Kit (Zymo Research) following manufacturer’s specifications. Ribosomal RNA was depleted using the RiboRid protocol (60), and RNA integrity was verified with a High-Sensitivity RNA ScreenTape Kit (Agilent Technologies). Strand-specific Illumina libraries were prepared using the KAPA RNA HyperPrep Kit (Kapa Biosystems). Paired-end sequencing was performed at UCSD internal sequencing facilities using a NovaSeq 6000. An average of ~23 million 40bp paired Illumina reads were obtained for each sample.

#### *aldA* complementation

*aldA* was PCR amplified along with its proximal upstream regulatory elements (47) from the genome of *E. coli* W3110S using primers oAC764 and oAC765 (Table S5). The amplified fragment was then cloned into pGRG36 (48) by restriction and ligation using the NotI and XhoI sites. The ligation product (pGRG36_aldA) was transformed in *E. coli* EC100 for screening and Sanger sequencing. Once verified, plasmid pGRG36_aldA was transformed and induced with 20 mM arabinose in both DGF-C and F1C2 strains to promote the integration of the *aldA* cassette into the genome. The resulting colonies were screened by PCR using primers oKN253 and oKN254 to search for successful integration. The clones bearing the correct integration (DGF-aldA, F1C2-aldA) were sequenced by Illumina and no additional mutations were detected.

### Computational methods

#### Flux balance analysis

Flux balance analysis was performed on the DGF-298 GEM using the COBRApy python module (27) with simulated minimal (MOPS/M9) or rich (EZ-Rich) media. Metabolic maps were generated using the Escher tool (61). Constraints used in FBA modeling M9/MOPS and EZ-Rich media are detailed in Table S1. Shadow prices were extracted from solution found by the COBRApy module after simulation in either MOPS- or EZ-Rich-simulated medium.

#### Independent component analysis

Briefly, iModulons are statistically independent groups of genes with correlated variation across a compendium of conditions. The latest version of this compendium, PRECISE-1K (62), includes most of the RNAseq data presented in this article under the nickname “minicoli”. Read trimming, alignment, filtering, and TPM calculation were performed as per the rest of PRECISE-1K. Expression data belonging to the genes removed in the DGF-298 strain were excluded to avoid inducing iModulon activity bias. Following standard iModulon activity calculation procedure, log2[TPM] values of remaining genes in each condition were then centered to the PRECISE-1K control condition (*E. coli* MG1655 in glucose M9 minimal media) by subtracting the control condition from them. Then, the infer_activities function from the pymodulon software package (37) was used on the centered log2[TPM] data to infer PRECISE-1K iModulon activities of all samples (File S1). Individual gene expression data (TPM) were taken from this PRECISE-1K analysis. As a control, RPKM were also calculated independently on those same reads, and DESeq analysis was performed on aligned reads. For DESeq analysis, raw reads were trimmed using fastp/0.23.2, filtered with samtools/1.14, and aligned with bowtie2/2.3.5 on either MG1655 (RefSeq NC_000913.3), W3110 (GenBank AP009048.1) modified to include functional *ilvG* and *pyrE* genes, or on the DGF-298 genome (GenBank CP127119.1), with or without the *aldA* gene (Table S7). Independent component analysis (ICA) was employed as a dimensionality reduction method to identify iModulons across strains and conditions (37). iModulon analysis was chosen for its ability to detect variations at the functional level and directly output information about the global transcription regulation, hence facilitating data interpretation. To ensure observed trends were not artifacts of library preparation, and control RNA-seq data were also generated for strains MG1655, W3110S, and E1C2 (Table S5) to evaluate if the evolution process itself could cause significant differences in the overall transcriptomic profile. Comparative analysis between the PRECISE control condition (MG1655 in M9 glucose) and our MG1655 or W3110S controls in M9 glucose showed consistent iModulon activity levels, with a few exceptions (Fig. S5 and S6). These deviations can be explained by factors, such as the addition of a cold centrifugation step during cell harvesting and documented strain particularities, such as the stochastic expression of motility genes in MG1655 (28, 63).

## Data Availability

RNA-seq data were deposited in the Gene Expression Omnibus database under series accession numbers GSE221155. DNA-seq Fastq files were deposited in the NCBI Sequence Read Archive under the following Bioproject : http://www.ncbi.nlm.nih.gov/bioproject/1006394

## SUPPLEMENTAL MATERIAL

File S1, part2 mbio.00873-24-s0001.pdf All i-modulon activities, part 2.

Supplemental figures mbio.00873-24-s0002.docx Figures S1 to S15.

File S1, part 1 mbio.00873-24-s0003.pdf All i-modulon activities, part 1.

Table S1 mbio.00873-24-s0004.xlsx DGF removed genes.

Table S2 mbio.00873-24-s0005.xlsx Model predictability test.

Table S3 mbio.00873-24-s0006.xlsx Growth data.

Table S4 mbio.00873-24-s0007.xlsx ALE mutations.

Table S5 mbio.00873-24-s0008.xlsx RNA-seq conditions.

Table S6 mbio.00873-24-s0009.xlsx iModulon activity changes.

Table S7 mbio.00873-24-s0010.xlsx Gene expression.

ASM does not own the copyrights to Supplemental Material that may be linked to, or accessed through, an article. The authors have granted ASM a non-exclusive, world-wide license to publish the Supplemental Material files. Please contact the corresponding author directly for reuse.
