# Supplementary material for: Diagnosis and mitigation of the systemic impact of genome reduction in Escherichia coli DGF-298
Source: mBio. 2024 Aug 29;15(10):e00873-24. doi: 10.1128/mbio.00873-24 (PMC11481515; doi:10.1128/mbio.00873-24)
Supplement: File S1, part2 — All i-modulon activities, part 2. [file mbio.00873-24-s0001.pdf]

Diagnosis and Mitigation of the Systemic Impact of Extensive Genome Reduction in *Escherichia coli* DGF-298

Antoine Champie[1], Jean-Christophe Lachance[1], Anand Sastry[2], Dominick Matteau[1], Colton J. Lloyd[2], Frédéric Grenier[1], Cameron R. Lamoureux[2], Simon Jeanneau[1], Adam M. Feist[2,5], Pierre-Étienne Jacques[1], Bernhard O. Palsson[2,3,4,5] & Sébastien Rodrigue[1†]

SUPPLEMENTARY FILE S1 : All iModulons activities

Activity level off all iModulons in the conditions tested. For color disambiguation conditions in the graphs are in the same order as the legend on the right.

Name of the groups of conditions :

- control = MG1655 in M9 glucose medium.
- ica = Diverse control conditions from the precise 1k database.
- miniEcoli_extra = Additional oxidative stress conditions on the reduced strains.
- miniEcoli = Main conditions tested in this article.
- oxidative = Oxidative stress conditions from the precise 1k database.

Legend of file S1 :

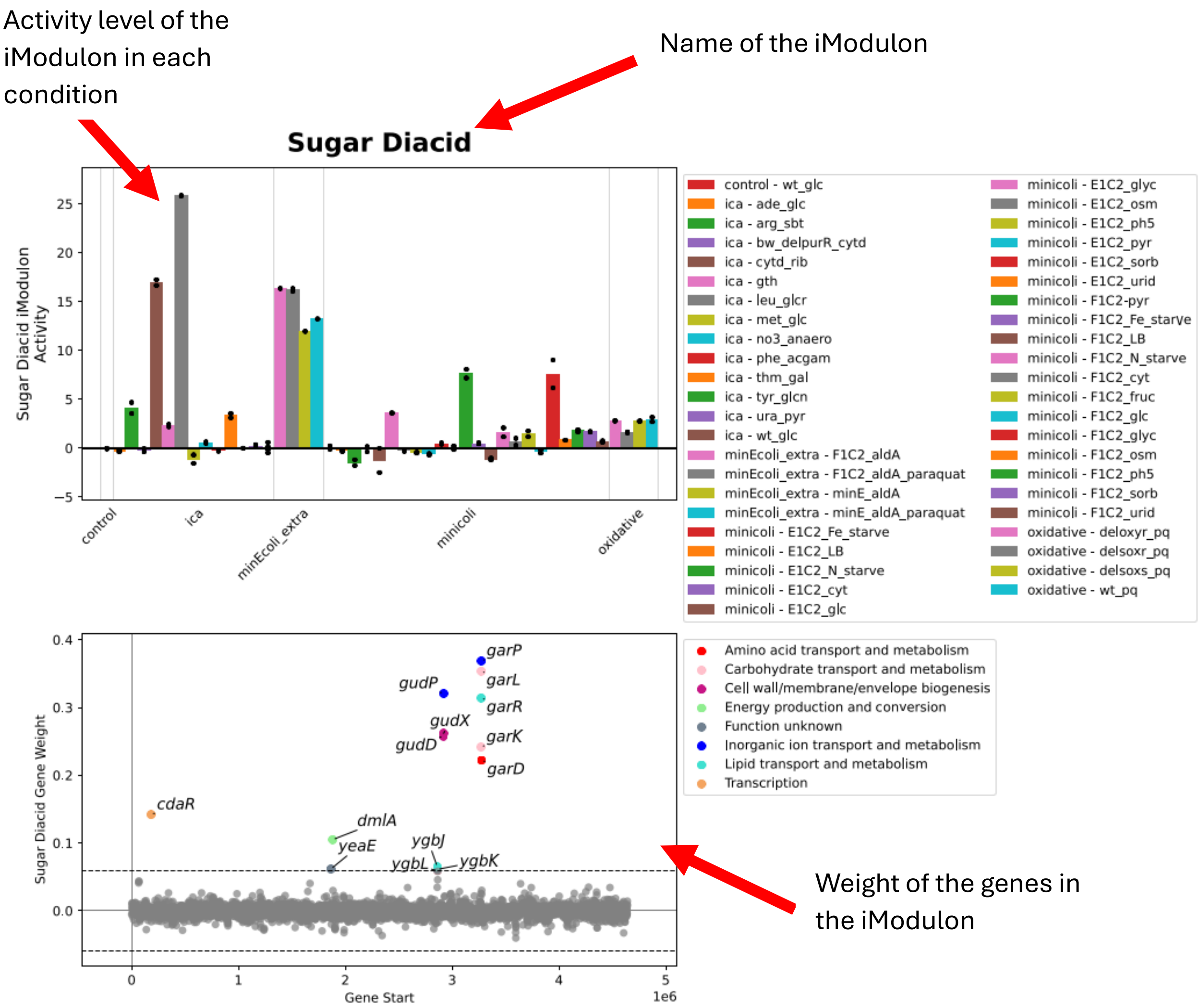

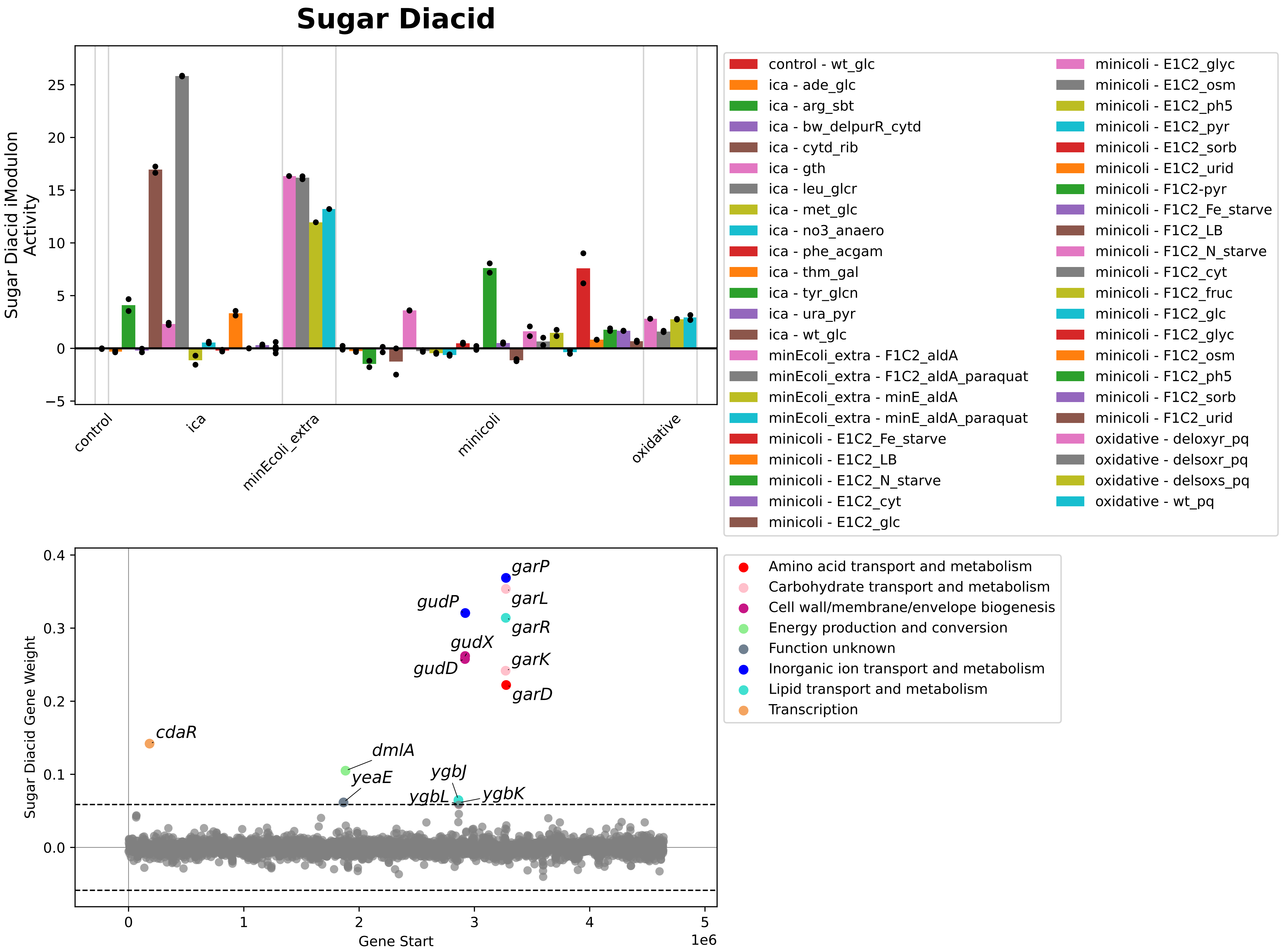
Sugar Diacid
Sugar Diacid iModulon Activity
control
ica
minEcoli_extra
minicoli
oxidative
control - wt_glc
ica - ade_glc
ica - arg_sbt
ica - bw_delpurR_cytd
ica - cytd_rib
ica - gth
ica - leu_glcr
ica - met_glc
ica - no3_anaero
ica - phe_acgam
ica - thm_gal
ica - tyr_glcn
ica - ura_pyr
ica - wt_glc
minEcoli_extra - F1C2_aldA
minEcoli_extra - F1C2_aldA_paraquat
minEcoli_extra - minE_aldA
minEcoli_extra - minE_aldA_paraquat
minicoli - E1C2_Fe_starve
minicoli - E1C2_LB
minicoli - E1C2_N_starve
minicoli - E1C2_cyt
minicoli - E1C2_glc
minicoli - E1C2_glyc
minicoli - E1C2_osm
minicoli - E1C2_ph5
minicoli - E1C2_pyr
minicoli - E1C2_sorb
minicoli - E1C2_urid
minicoli - F1C2-pyr
minicoli - F1C2_Fe_starve
minicoli - F1C2_LB
minicoli - F1C2_N_starve
minicoli - F1C2_cyt
minicoli - F1C2_fruc
minicoli - F1C2_glc
minicoli - F1C2_glyc
minicoli - F1C2_osm
minicoli - F1C2_ph5
minicoli - F1C2_sorb
minicoli - F1C2_urid
oxidative - deloxyr_pq
oxidative - delsoxr_pq
oxidative - delsoxs_pq
oxidative - wt_pq
Sugar Diacid Gene Weight
Gene Start
1e6
garP
garL
gudP
garR
gudX
garK
gudD
garD
cdaR
dmlA
yeaE
ygbJ
ygbL
ygbK
Amino acid transport and metabolism
Carbohydrate transport and metabolism
Cell wall/membrane/envelope biogenesis
Energy production and conversion
Function unknown
Inorganic ion transport and metabolism
Lipid transport and metabolism
Transcription

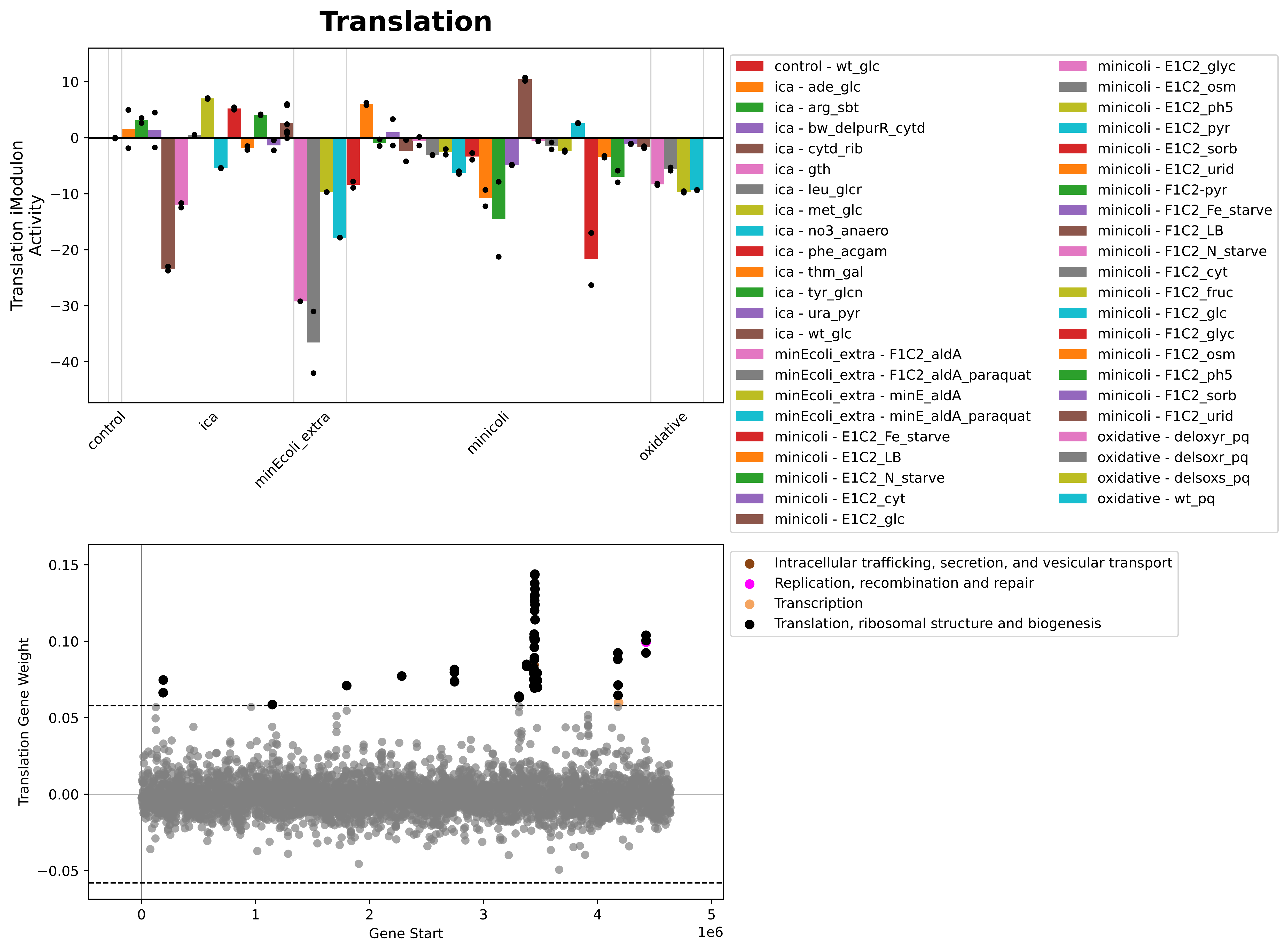

Translation
Translation iModulon Activity
control
ica
minEcoli_extra
minicoli
oxidative
control - wt_glc
ica - ade_glc
ica - arg_sbt
ica - bw_delpurR_cytd
ica - cytd_rib
ica - gth
ica - leu_glcr
ica - met_glc
ica - no3_anaero
ica - phe_acgam
ica - thm_gal
ica - tyr_glcn
ica - ura_pyr
ica - wt_glc
minEcoli_extra - F1C2_aldA
minEcoli_extra - F1C2_aldA_paraquat
minEcoli_extra - minE_aldA
minEcoli_extra - minE_aldA_paraquat
minicoli - E1C2_Fe_starve
minicoli - E1C2_LB
minicoli - E1C2_N_starve
minicoli - E1C2_cyt
minicoli - E1C2_glc
minicoli - E1C2_glyc
minicoli - E1C2_osm
minicoli - E1C2_ph5
minicoli - E1C2_pyr
minicoli - E1C2_sorb
minicoli - E1C2_urid
minicoli - F1C2-pyr
minicoli - F1C2_Fe_starve
minicoli - F1C2_LB
minicoli - F1C2_N_starve
minicoli - F1C2_cyt
minicoli - F1C2_fruc
minicoli - F1C2_glc
minicoli - F1C2_glyc
minicoli - F1C2_osm
minicoli - F1C2_ph5
minicoli - F1C2_sorb
minicoli - F1C2_urid
oxidative - deloxyr_pq
oxidative - delsoxr_pq
oxidative - delsoxs_pq
oxidative - wt_pq
Translation Gene Weight
Gene Start
1e6
Intracellular trafficking, secretion, and vesicular transport
Replication, recombination and repair
Transcription
Translation, ribosomal structure and biogenesis

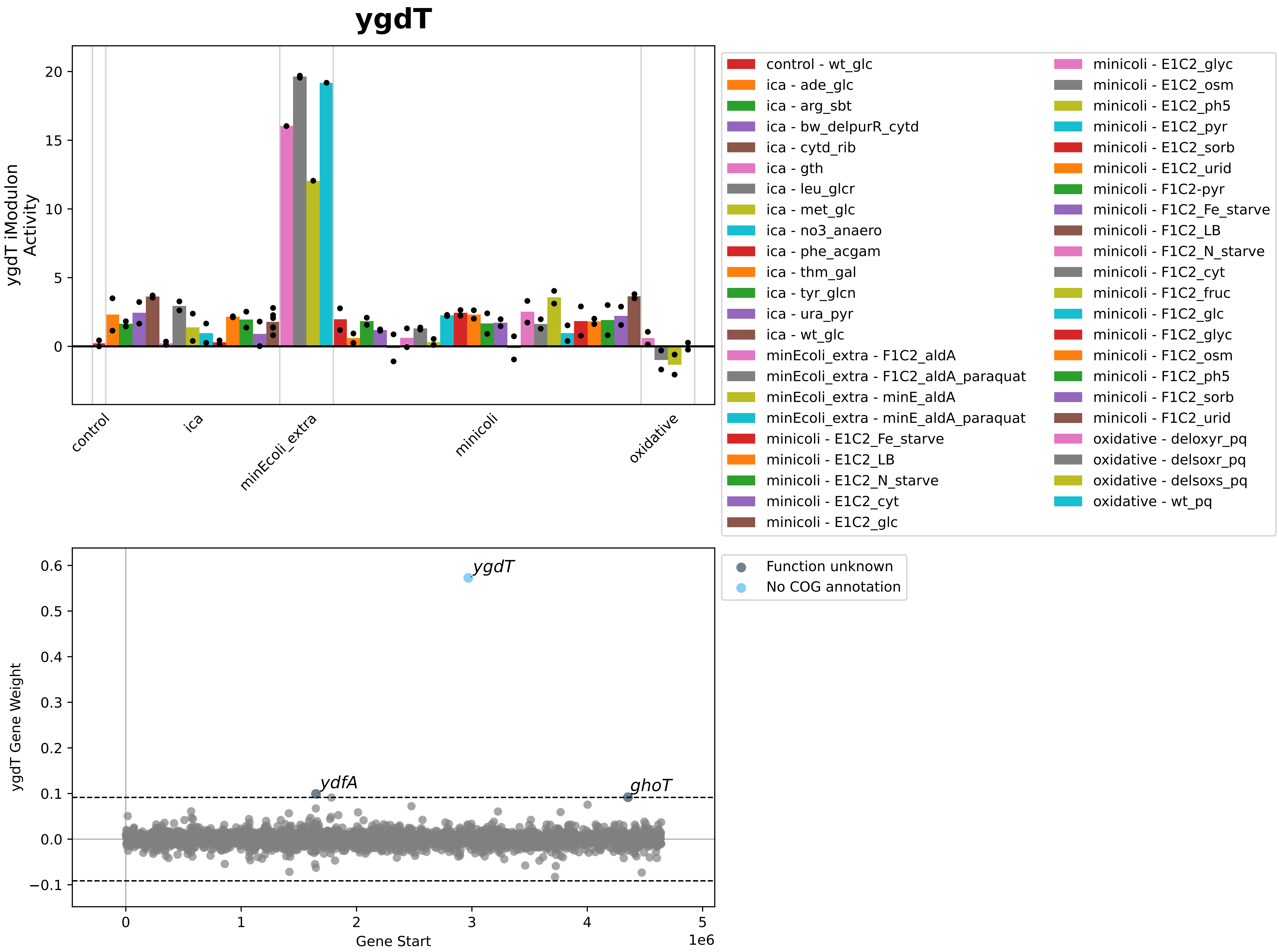

ygdT
ygdT iModulon Activity
control
ica
minEcoli_extra
minicoli
oxidative
control - wt_glc
ica - ade_glc
ica - arg_sbt
ica - bw_delpurR_cytd
ica - cytd_rib
ica - gth
ica - leu_glcr
ica - met_glc
ica - no3_anaero
ica - phe_acgam
ica - thm_gal
ica - tyr_glcn
ica - ura_pyr
ica - wt_glc
minEcoli_extra - F1C2_aldA
minEcoli_extra - F1C2_aldA_paraquat
minEcoli_extra - minE_aldA
minEcoli_extra - minE_aldA_paraquat
minicoli - E1C2_Fe_starve
minicoli - E1C2_LB
minicoli - E1C2_N_starve
minicoli - E1C2_cyt
minicoli - E1C2_glc
minicoli - E1C2_glyc
minicoli - E1C2_osm
minicoli - E1C2_ph5
minicoli - E1C2_pyr
minicoli - E1C2_sorb
minicoli - E1C2_urid
minicoli - F1C2-pyr
minicoli - F1C2_Fe_starve
minicoli - F1C2_LB
minicoli - F1C2_N_starve
minicoli - F1C2_cyt
minicoli - F1C2_fruc
minicoli - F1C2_glc
minicoli - F1C2_glyc
minicoli - F1C2_osm
minicoli - F1C2_ph5
minicoli - F1C2_sorb
minicoli - F1C2_urid
oxidative - deloxyr_pq
oxidative - delsoxr_pq
oxidative - delsoxs_pq
oxidative - wt_pq
ygdT Gene Weight
Gene Start
1e6
ygdT
ydfA
ghoT
Function unknown
No COG annotation

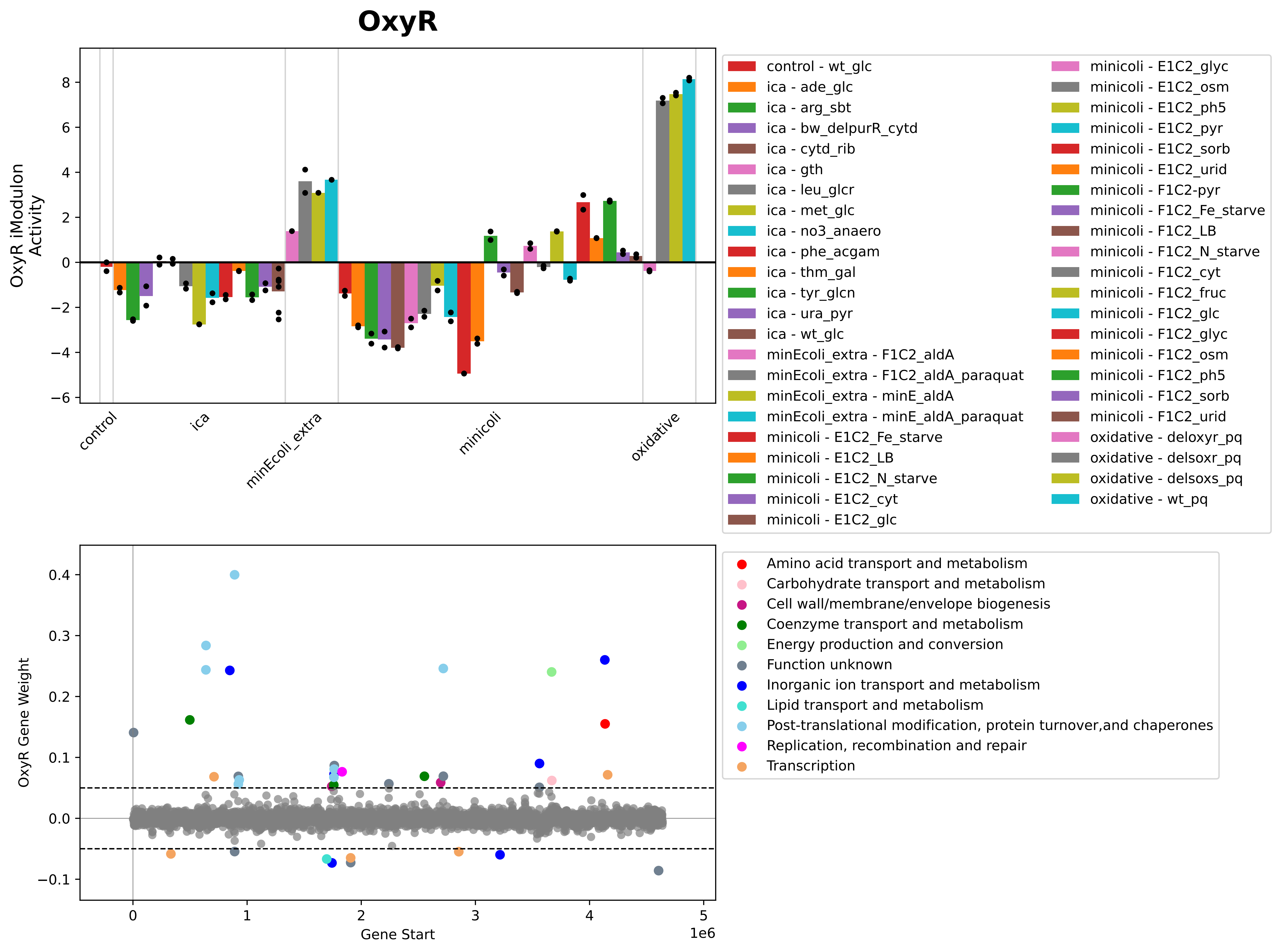
OxyR
OxyR iModulon Activity
control
ica
minEcoli_extra
minicoli
oxidative
control - wt_glc
ica - ade_glc
ica - arg_sbt
ica - bw_delpurR_cytd
ica - cytd_rib
ica - gth
ica - leu_glcr
ica - met_glc
ica - no3_anaero
ica - phe_acgam
ica - thm_gal
ica - tyr_glcn
ica - ura_pyr
ica - wt_glc
minEcoli_extra - F1C2_aldA
minEcoli_extra - F1C2_aldA_paraquat
minEcoli_extra - minE_aldA
minEcoli_extra - minE_aldA_paraquat
minicoli - E1C2_Fe_starve
minicoli - E1C2_LB
minicoli - E1C2_N_starve
minicoli - E1C2_cyt
minicoli - E1C2_glc
minicoli - E1C2_glyc
minicoli - E1C2_osm
minicoli - E1C2_ph5
minicoli - E1C2_pyr
minicoli - E1C2_sorb
minicoli - E1C2_urid
minicoli - F1C2-pyr
minicoli - F1C2_Fe_starve
minicoli - F1C2_LB
minicoli - F1C2_N_starve
minicoli - F1C2_cyt
minicoli - F1C2_fruc
minicoli - F1C2_glc
minicoli - F1C2_glyc
minicoli - F1C2_osm
minicoli - F1C2_ph5
minicoli - F1C2_sorb
minicoli - F1C2_urid
oxidative - deloxyr_pq
oxidative - delsoxr_pq
oxidative - delsoxs_pq
oxidative - wt_pq
OxyR Gene Weight
Gene Start
1e6
Amino acid transport and metabolism
Carbohydrate transport and metabolism
Cell wall/membrane/envelope biogenesis
Coenzyme transport and metabolism
Energy production and conversion
Function unknown
Inorganic ion transport and metabolism
Lipid transport and metabolism
Post-translational modification, protein turnover,and chaperones
Replication, recombination and repair
Transcription

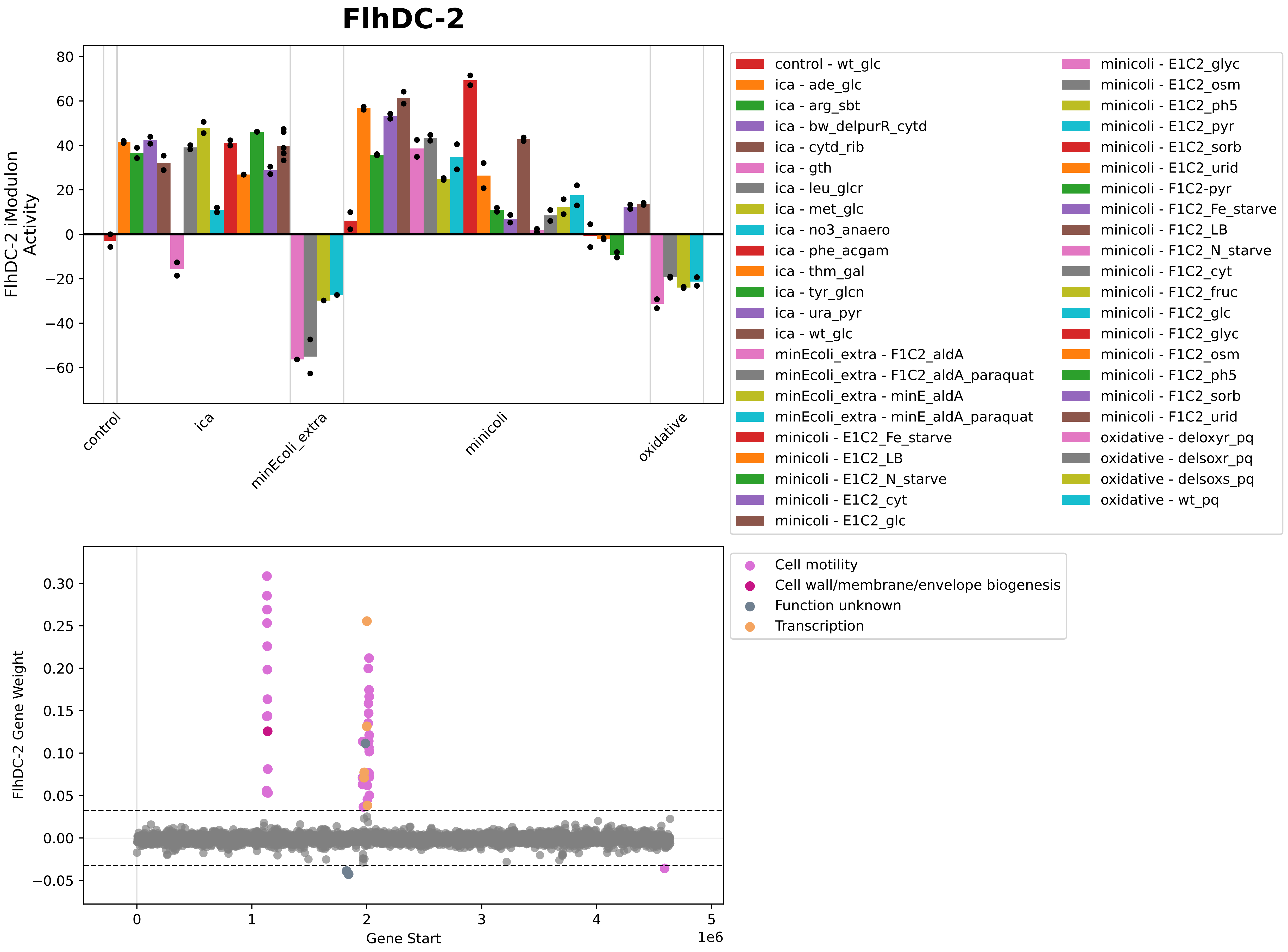

FlhDC-2
FlhDC-2 iModulon Activity
80
60
40
20
0
−20
−40
−60
control
ica
minEcoli_extra
minicoli
oxidative
control - wt_glc
ica - ade_glc
ica - arg_sbt
ica - bw_delpurR_cytd
ica - cytd_rib
ica - gth
ica - leu_glcr
ica - met_glc
ica - no3_anaero
ica - phe_acgam
ica - thm_gal
ica - tyr_glcn
ica - ura_pyr
ica - wt_glc
minEcoli_extra - F1C2_aldA
minEcoli_extra - F1C2_aldA_paraquat
minEcoli_extra - minE_aldA
minEcoli_extra - minE_aldA_paraquat
minicoli - E1C2_Fe_starve
minicoli - E1C2_LB
minicoli - E1C2_N_starve
minicoli - E1C2_cyt
minicoli - E1C2_glc
minicoli - E1C2_glyc
minicoli - E1C2_osm
minicoli - E1C2_ph5
minicoli - E1C2_pyr
minicoli - E1C2_sorb
minicoli - E1C2_urid
minicoli - F1C2-pyr
minicoli - F1C2_Fe_starve
minicoli - F1C2_LB
minicoli - F1C2_N_starve
minicoli - F1C2_cyt
minicoli - F1C2_fruc
minicoli - F1C2_glc
minicoli - F1C2_glyc
minicoli - F1C2_osm
minicoli - F1C2_ph5
minicoli - F1C2_sorb
minicoli - F1C2_urid
oxidative - deloxyr_pq
oxidative - delsoxr_pq
oxidative - delsoxs_pq
oxidative - wt_pq
FlhDC-2 Gene Weight
0.30
0.25
0.20
0.15
0.10
0.05
0.00
−0.05
0
1
2
3
4
5
1e6
Gene Start
Cell motility
Cell wall/membrane/envelope biogenesis
Function unknown
Transcription

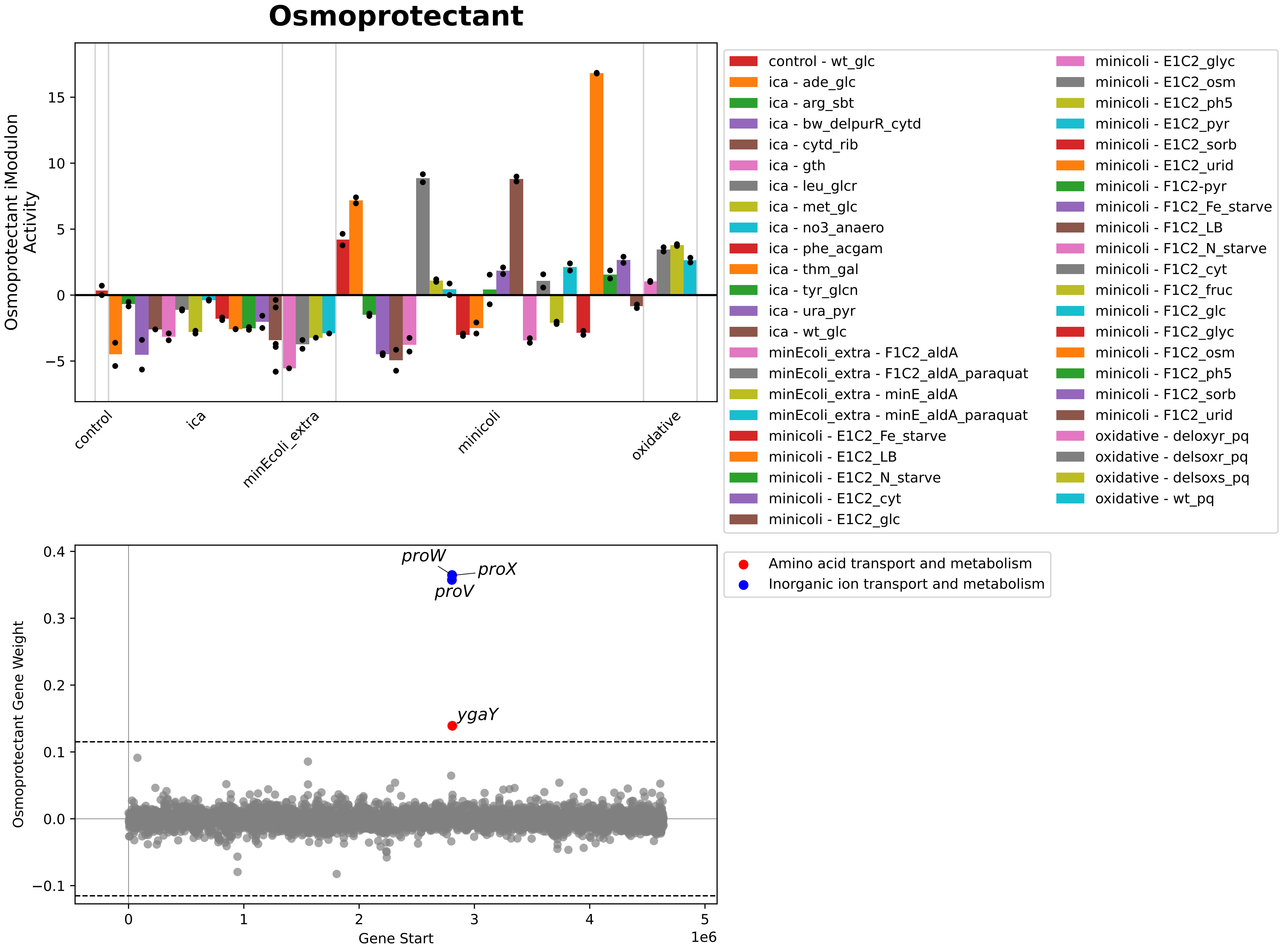
Osmoprotectant
Osmoprotectant iModulon Activity
control
ica
minEcoli_extra
minicoli
oxidative
control - wt_glc
ica - ade_glc
ica - arg_sbt
ica - bw_delpurR_cytd
ica - cytd_rib
ica - gth
ica - leu_glcr
ica - met_glc
ica - no3_anaero
ica - phe_acgam
ica - thm_gal
ica - tyr_glcn
ica - ura_pyr
ica - wt_glc
minEcoli_extra - F1C2_aldA
minEcoli_extra - F1C2_aldA_paraquat
minEcoli_extra - minE_aldA
minEcoli_extra - minE_aldA_paraquat
minicoli - E1C2_Fe_starve
minicoli - E1C2_LB
minicoli - E1C2_N_starve
minicoli - E1C2_cyt
minicoli - E1C2_glc
minicoli - E1C2_glyc
minicoli - E1C2_osm
minicoli - E1C2_ph5
minicoli - E1C2_pyr
minicoli - E1C2_sorb
minicoli - E1C2_urid
minicoli - F1C2-pyr
minicoli - F1C2_Fe_starve
minicoli - F1C2_LB
minicoli - F1C2_N_starve
minicoli - F1C2_cyt
minicoli - F1C2_fruc
minicoli - F1C2_glc
minicoli - F1C2_glyc
minicoli - F1C2_osm
minicoli - F1C2_ph5
minicoli - F1C2_sorb
minicoli - F1C2_urid
oxidative - deloxyr_pq
oxidative - delsoxr_pq
oxidative - delsoxs_pq
oxidative - wt_pq
proW
proX
proV
ygaY
Osmoprotectant Gene Weight
Gene Start
1e6
Amino acid transport and metabolism
Inorganic ion transport and metabolism

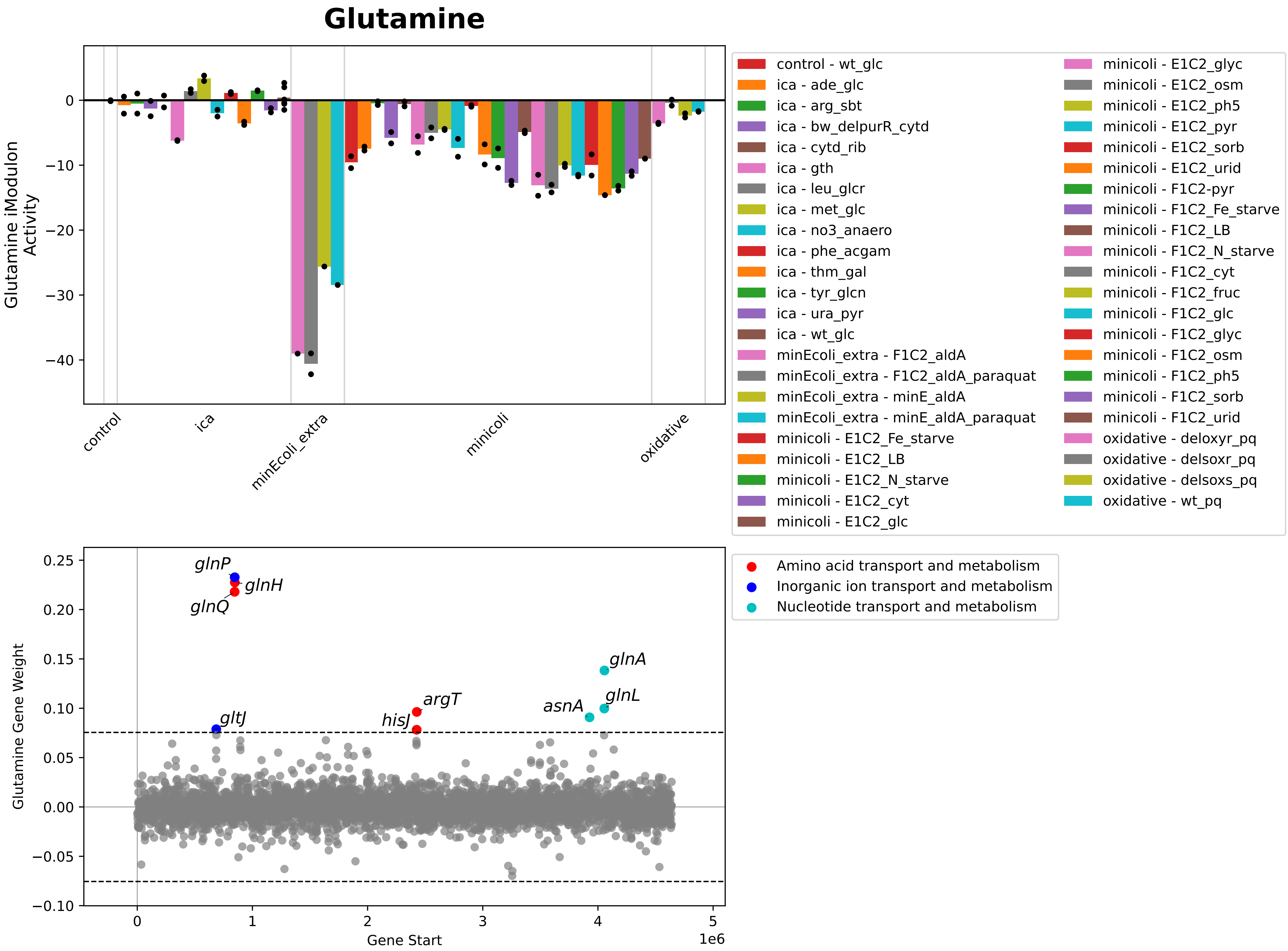
Glutamine
Glutamine iModulon Activity
0
−10
−20
−30
−40
control
ica
minEcoli_extra
minicoli
oxidative
control - wt_glc
ica - ade_glc
ica - arg_sbt
ica - bw_delpurR_cytd
ica - cytd_rib
ica - gth
ica - leu_glcr
ica - met_glc
ica - no3_anaero
ica - phe_acgam
ica - thm_gal
ica - tyr_glcn
ica - ura_pyr
ica - wt_glc
minEcoli_extra - F1C2_aldA
minEcoli_extra - F1C2_aldA_paraquat
minEcoli_extra - minE_aldA
minEcoli_extra - minE_aldA_paraquat
minicoli - E1C2_Fe_starve
minicoli - E1C2_LB
minicoli - E1C2_N_starve
minicoli - E1C2_cyt
minicoli - E1C2_glc
minicoli - E1C2_glyc
minicoli - E1C2_osm
minicoli - E1C2_ph5
minicoli - E1C2_pyr
minicoli - E1C2_sorb
minicoli - E1C2_urid
minicoli - F1C2-pyr
minicoli - F1C2_Fe_starve
minicoli - F1C2_LB
minicoli - F1C2_N_starve
minicoli - F1C2_cyt
minicoli - F1C2_fruc
minicoli - F1C2_glc
minicoli - F1C2_glyc
minicoli - F1C2_osm
minicoli - F1C2_ph5
minicoli - F1C2_sorb
minicoli - F1C2_urid
oxidative - deloxyr_pq
oxidative - delsoxr_pq
oxidative - delsoxs_pq
oxidative - wt_pq
Glutamine Gene Weight
0.25
0.20
0.15
0.10
0.05
0.00
−0.05
−0.10
glnP
glnH
glnQ
glnA
argT
asnA
glnL
gltJ
hisJ
0
1
2
3
4
5
1e6
Gene Start
Amino acid transport and metabolism
Inorganic ion transport and metabolism
Nucleotide transport and metabolism

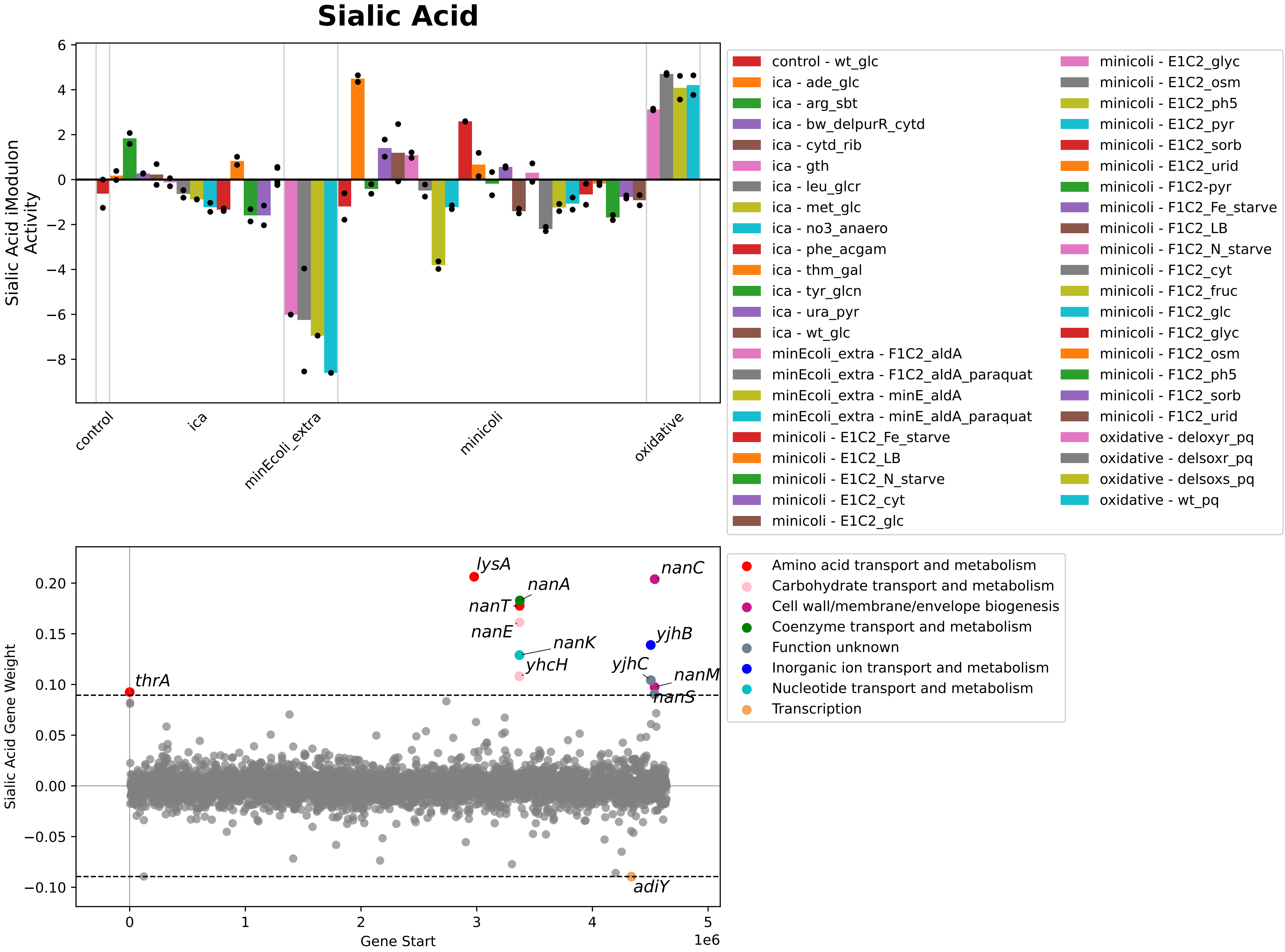

Sialic Acid
Sialic Acid iModulon Activity
control
ica
minEcoli_extra
minicoli
oxidative
control - wt_glc
ica - ade_glc
ica - arg_sbt
ica - bw_delpurR_cytd
ica - cytd_rib
ica - gth
ica - leu_glcr
ica - met_glc
ica - no3_anaero
ica - phe_acgam
ica - thm_gal
ica - tyr_glcn
ica - ura_pyr
ica - wt_glc
minEcoli_extra - F1C2_aldA
minEcoli_extra - F1C2_aldA_paraquat
minEcoli_extra - minE_aldA
minEcoli_extra - minE_aldA_paraquat
minicoli - E1C2_Fe_starve
minicoli - E1C2_LB
minicoli - E1C2_N_starve
minicoli - E1C2_cyt
minicoli - E1C2_glc
minicoli - E1C2_glyc
minicoli - E1C2_osm
minicoli - E1C2_ph5
minicoli - E1C2_pyr
minicoli - E1C2_sorb
minicoli - E1C2_urid
minicoli - F1C2-pyr
minicoli - F1C2_Fe_starve
minicoli - F1C2_LB
minicoli - F1C2_N_starve
minicoli - F1C2_cyt
minicoli - F1C2_fruc
minicoli - F1C2_glc
minicoli - F1C2_glyc
minicoli - F1C2_osm
minicoli - F1C2_ph5
minicoli - F1C2_sorb
minicoli - F1C2_urid
oxidative - deloxyr_pq
oxidative - delsoxr_pq
oxidative - delsoxs_pq
oxidative - wt_pq
Sialic Acid Gene Weight
Gene Start
1e6
lysA
nanC
nanA
nanT
nanE
nanK
yjhB
yhcH
yjhC
nanM
nanS
thrA
adiY
Amino acid transport and metabolism
Carbohydrate transport and metabolism
Cell wall/membrane/envelope biogenesis
Coenzyme transport and metabolism
Function unknown
Inorganic ion transport and metabolism
Nucleotide transport and metabolism
Transcription

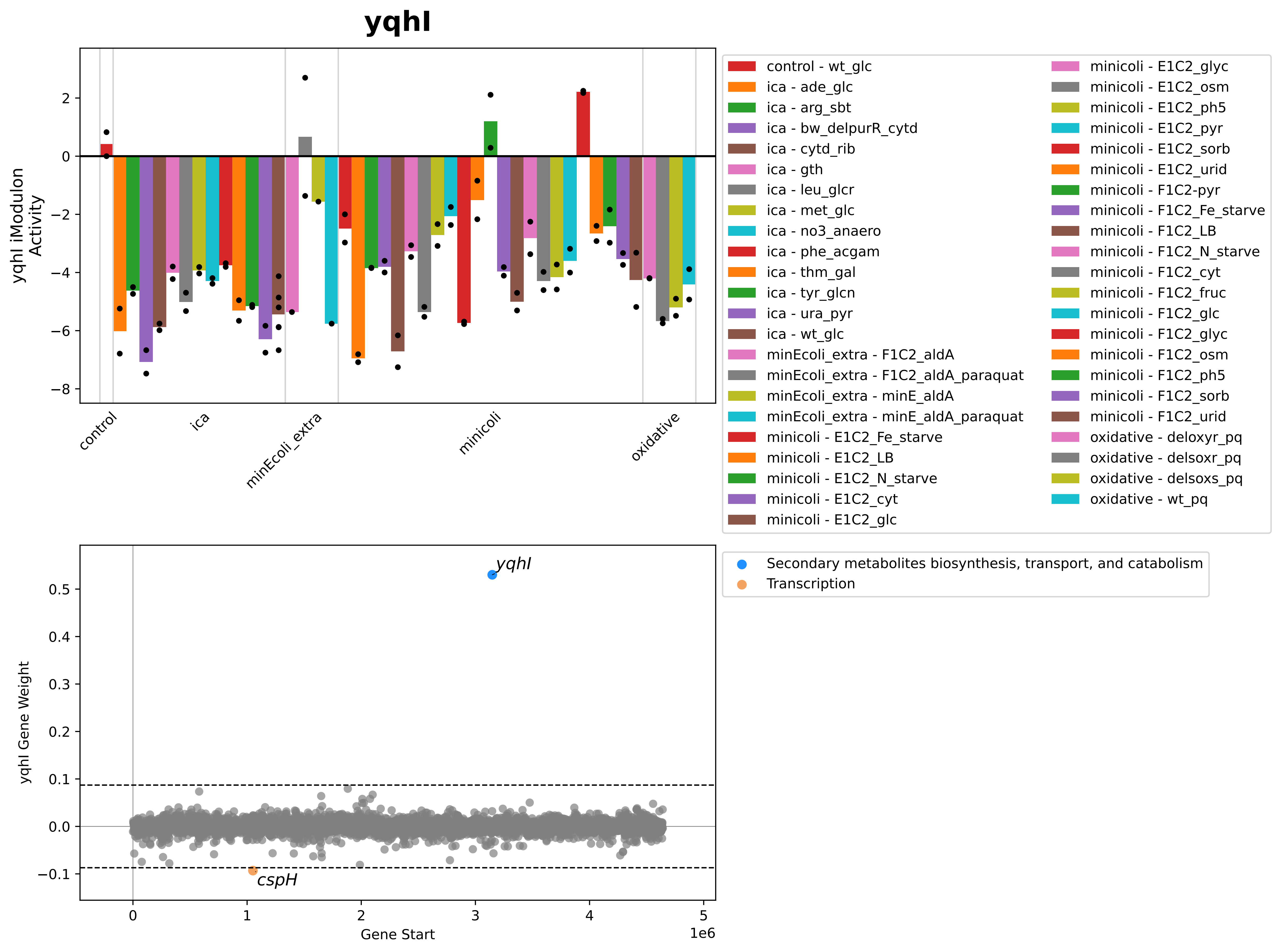

yqhI
yqhI iModulon Activity
control
ica
minEcoli_extra
minicoli
oxidative
control - wt_glc
ica - ade_glc
ica - arg_sbt
ica - bw_delpurR_cytd
ica - cytd_rib
ica - gth
ica - leu_glcr
ica - met_glc
ica - no3_anaero
ica - phe_acgam
ica - thm_gal
ica - tyr_glcn
ica - ura_pyr
ica - wt_glc
minEcoli_extra - F1C2_aldA
minEcoli_extra - F1C2_aldA_paraquat
minEcoli_extra - minE_aldA
minEcoli_extra - minE_aldA_paraquat
minicoli - E1C2_Fe_starve
minicoli - E1C2_LB
minicoli - E1C2_N_starve
minicoli - E1C2_cyt
minicoli - E1C2_glc
minicoli - E1C2_glyc
minicoli - E1C2_osm
minicoli - E1C2_ph5
minicoli - E1C2_pyr
minicoli - E1C2_sorb
minicoli - E1C2_urid
minicoli - F1C2-pyr
minicoli - F1C2_Fe_starve
minicoli - F1C2_LB
minicoli - F1C2_N_starve
minicoli - F1C2_cyt
minicoli - F1C2_fruc
minicoli - F1C2_glc
minicoli - F1C2_glyc
minicoli - F1C2_osm
minicoli - F1C2_ph5
minicoli - F1C2_sorb
minicoli - F1C2_urid
oxidative - deloxyr_pq
oxidative - delsoxr_pq
oxidative - delsoxs_pq
oxidative - wt_pq
yqhI
cspH
yqhI Gene Weight
Gene Start
1e6
Secondary metabolites biosynthesis, transport, and catabolism
Transcription

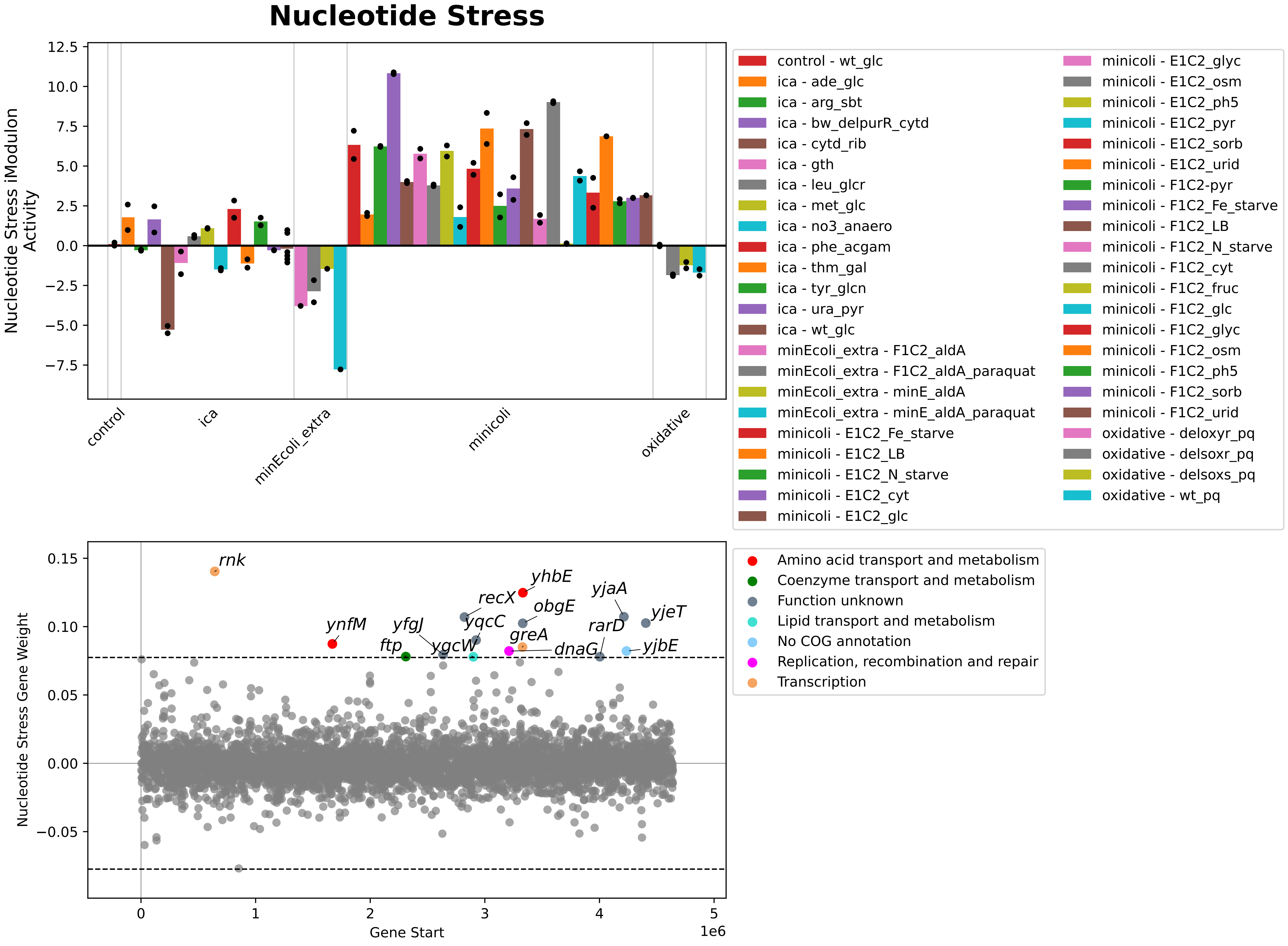

Nucleotide Stress
Nucleotide Stress iModulon Activity
control
ica
minEcoli_extra
minicoli
oxidative
control - wt_glc
ica - ade_glc
ica - arg_sbt
ica - bw_delpurR_cytd
ica - cytd_rib
ica - gth
ica - leu_glcr
ica - met_glc
ica - no3_anaero
ica - phe_acgam
ica - thm_gal
ica - tyr_glcn
ica - ura_pyr
ica - wt_glc
minEcoli_extra - F1C2_aldA
minEcoli_extra - F1C2_aldA_paraquat
minEcoli_extra - minE_aldA
minEcoli_extra - minE_aldA_paraquat
minicoli - E1C2_Fe_starve
minicoli - E1C2_LB
minicoli - E1C2_N_starve
minicoli - E1C2_cyt
minicoli - E1C2_glc
minicoli - E1C2_glyc
minicoli - E1C2_osm
minicoli - E1C2_ph5
minicoli - E1C2_pyr
minicoli - E1C2_sorb
minicoli - E1C2_urid
minicoli - F1C2-pyr
minicoli - F1C2_Fe_starve
minicoli - F1C2_LB
minicoli - F1C2_N_starve
minicoli - F1C2_cyt
minicoli - F1C2_fruc
minicoli - F1C2_glc
minicoli - F1C2_glyc
minicoli - F1C2_osm
minicoli - F1C2_ph5
minicoli - F1C2_sorb
minicoli - F1C2_urid
oxidative - deloxyr_pq
oxidative - delsoxr_pq
oxidative - delsoxs_pq
oxidative - wt_pq
Nucleotide Stress Gene Weight
Gene Start
1e6
rnk
yhbE
recX
obgE
yjaA
yjeT
ynfM
yfgJ
yqcC
greA
rarD
ftp
ygcW
dnaG
yjbE
Amino acid transport and metabolism
Coenzyme transport and metabolism
Function unknown
Lipid transport and metabolism
No COG annotation
Replication, recombination and repair
Transcription

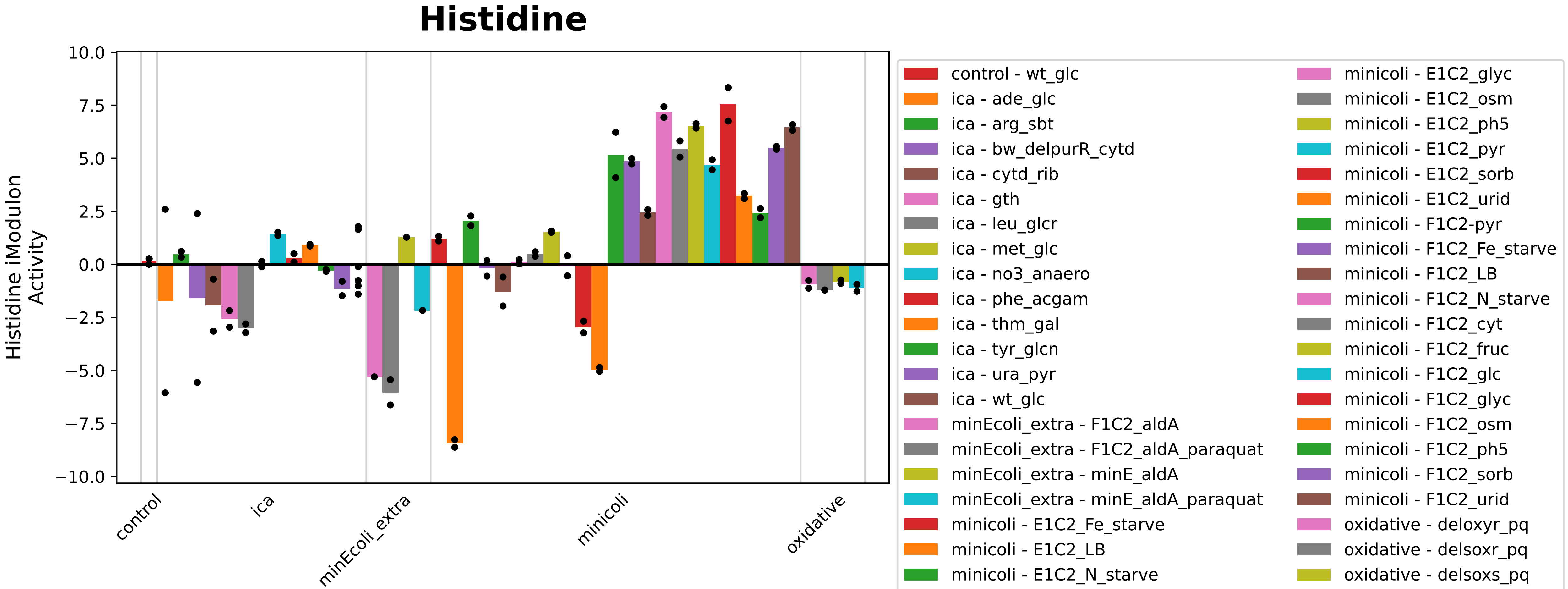

Histidine
Histidine iModulon Activity
10.0
7.5
5.0
2.5
0.0
−2.5
−5.0
−7.5
−10.0
control
ica
minEcoli_extra
minicoli
oxidative
control - wt_glc
ica - ade_glc
ica - arg_sbt
ica - bw_delpurR_cytd
ica - cytd_rib
ica - gth
ica - leu_glcr
ica - met_glc
ica - no3_anaero
ica - phe_acgam
ica - thm_gal
ica - tyr_glcn
ica - ura_pyr
ica - wt_glc
minEcoli_extra - F1C2_aldA
minEcoli_extra - F1C2_aldA_paraquat
minEcoli_extra - minE_aldA
minEcoli_extra - minE_aldA_paraquat
minicoli - E1C2_Fe_starve
minicoli - E1C2_LB
minicoli - E1C2_N_starve
minicoli - E1C2_cyt
minicoli - E1C2_glc
minicoli - E1C2_glyc
minicoli - E1C2_osm
minicoli - E1C2_ph5
minicoli - E1C2_pyr
minicoli - E1C2_sorb
minicoli - E1C2_urid
minicoli - F1C2-pyr
minicoli - F1C2_Fe_starve
minicoli - F1C2_LB
minicoli - F1C2_N_starve
minicoli - F1C2_cyt
minicoli - F1C2_fruc
minicoli - F1C2_glc
minicoli - F1C2_glyc
minicoli - F1C2_osm
minicoli - F1C2_ph5
minicoli - F1C2_sorb
minicoli - F1C2_urid
oxidative - deloxyr_pq
oxidative - delsoxr_pq
oxidative - delsoxs_pq
oxidative - wt_pq

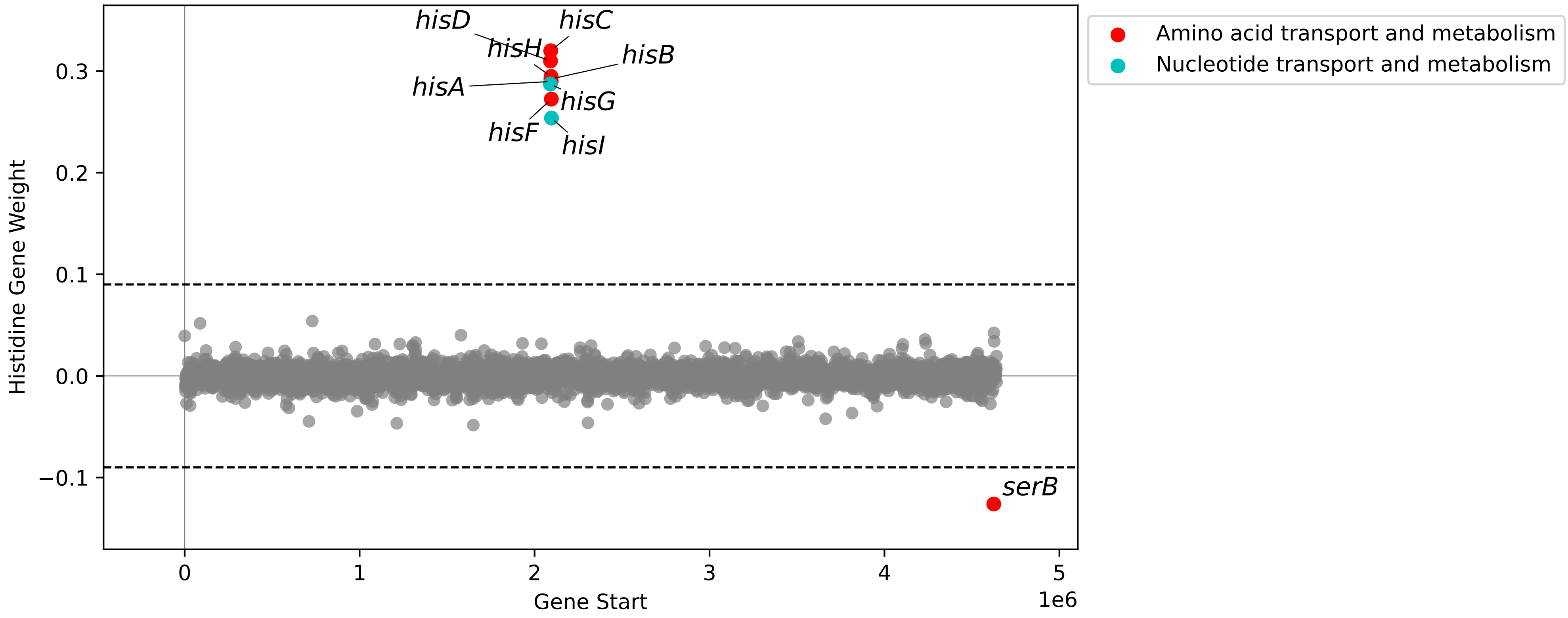

Histidine Gene Weight
0.3
0.2
0.1
0.0
−0.1
hisD
hisC
hisH
hisB
hisA
hisG
hisF
hisI
serB
0
1
2
3
4
5
Gene Start
1e6
Amino acid transport and metabolism
Nucleotide transport and metabolism

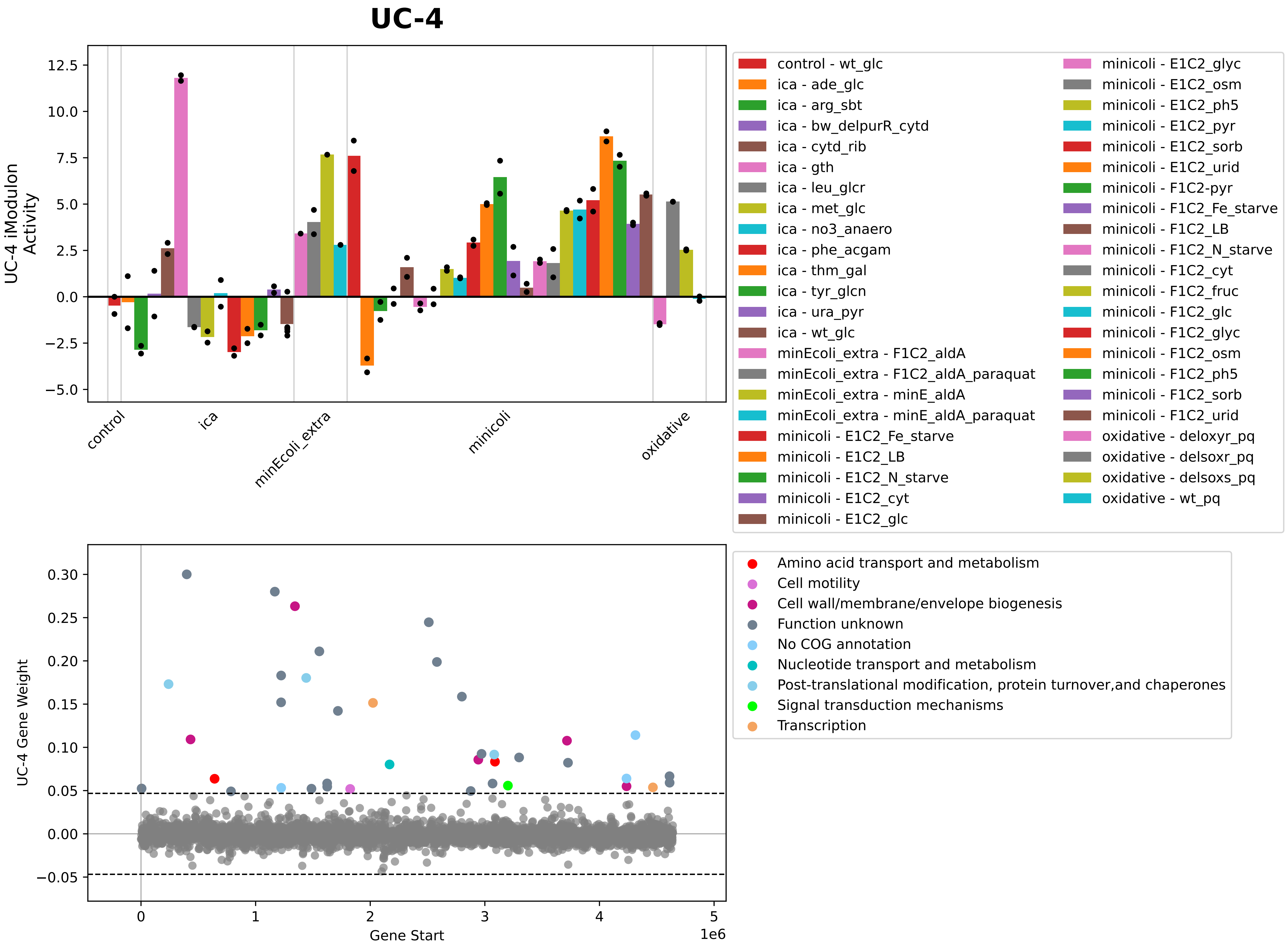

UC-4
UC-4 iModulon Activity
control
ica
minEcoli_extra
minicoli
oxidative
control - wt_glc
ica - ade_glc
ica - arg_sbt
ica - bw_delpurR_cytd
ica - cytd_rib
ica - gth
ica - leu_glcr
ica - met_glc
ica - no3_anaero
ica - phe_acgam
ica - thm_gal
ica - tyr_glcn
ica - ura_pyr
ica - wt_glc
minEcoli_extra - F1C2_aldA
minEcoli_extra - F1C2_aldA_paraquat
minEcoli_extra - minE_aldA
minEcoli_extra - minE_aldA_paraquat
minicoli - E1C2_Fe_starve
minicoli - E1C2_LB
minicoli - E1C2_N_starve
minicoli - E1C2_cyt
minicoli - E1C2_glc
minicoli - E1C2_glyc
minicoli - E1C2_osm
minicoli - E1C2_ph5
minicoli - E1C2_pyr
minicoli - E1C2_sorb
minicoli - E1C2_urid
minicoli - F1C2-pyr
minicoli - F1C2_Fe_starve
minicoli - F1C2_LB
minicoli - F1C2_N_starve
minicoli - F1C2_cyt
minicoli - F1C2_fruc
minicoli - F1C2_glc
minicoli - F1C2_glyc
minicoli - F1C2_osm
minicoli - F1C2_ph5
minicoli - F1C2_sorb
minicoli - F1C2_urid
oxidative - deloxyr_pq
oxidative - delsoxr_pq
oxidative - delsoxs_pq
oxidative - wt_pq
UC-4 Gene Weight
Gene Start
1e6
Amino acid transport and metabolism
Cell motility
Cell wall/membrane/envelope biogenesis
Function unknown
No COG annotation
Nucleotide transport and metabolism
Post-translational modification, protein turnover,and chaperones
Signal transduction mechanisms
Transcription

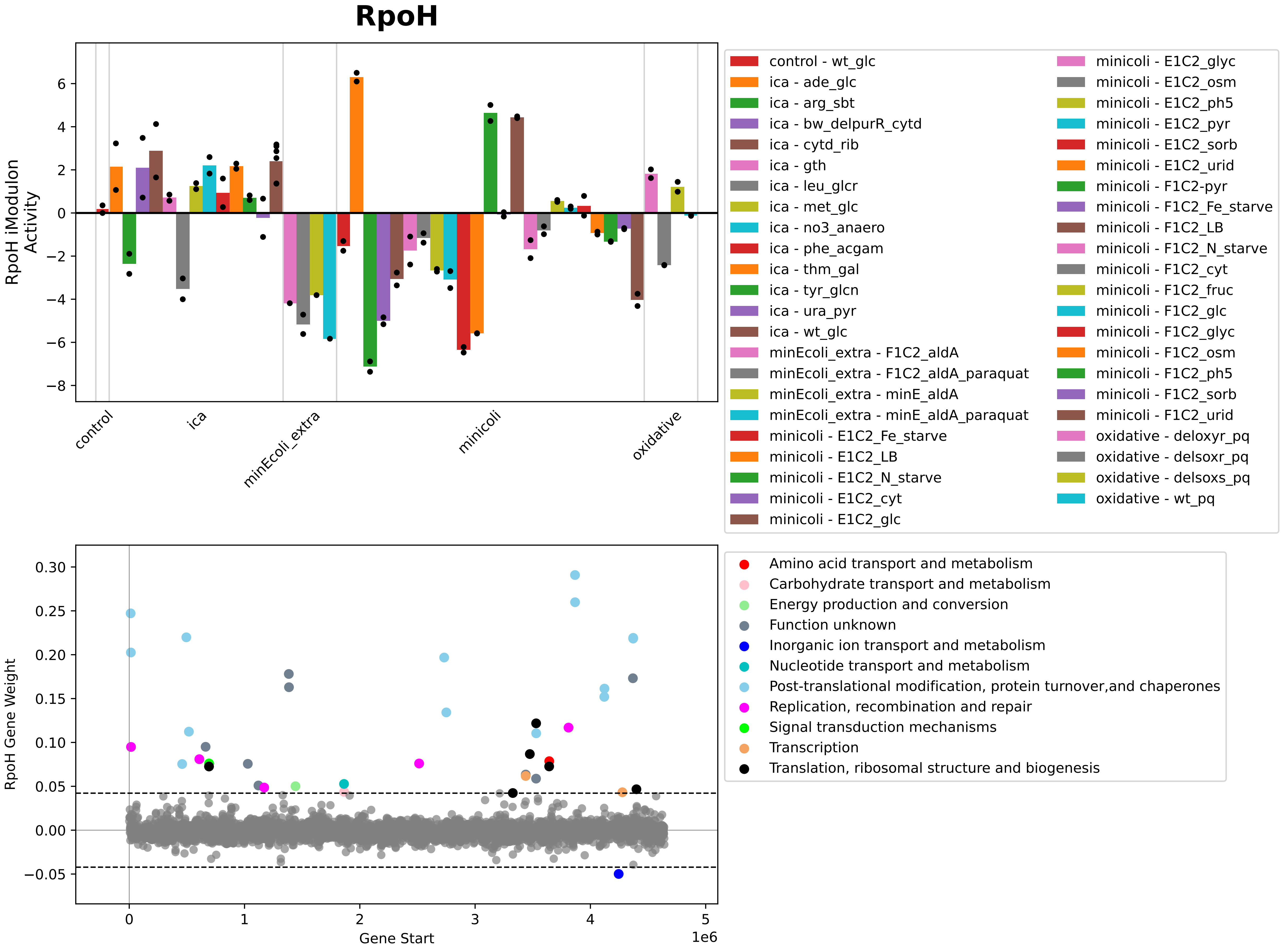
RpoH
RpoH iModulon Activity
control
ica
minEcoli_extra
minicoli
oxidative
control - wt_glc
ica - ade_glc
ica - arg_sbt
ica - bw_delpurR_cytd
ica - cytd_rib
ica - gth
ica - leu_glcr
ica - met_glc
ica - no3_anaero
ica - phe_acgam
ica - thm_gal
ica - tyr_glcn
ica - ura_pyr
ica - wt_glc
minEcoli_extra - F1C2_aldA
minEcoli_extra - F1C2_aldA_paraquat
minEcoli_extra - minE_aldA
minEcoli_extra - minE_aldA_paraquat
minicoli - E1C2_Fe_starve
minicoli - E1C2_LB
minicoli - E1C2_N_starve
minicoli - E1C2_cyt
minicoli - E1C2_glc
minicoli - E1C2_glyc
minicoli - E1C2_osm
minicoli - E1C2_ph5
minicoli - E1C2_pyr
minicoli - E1C2_sorb
minicoli - E1C2_urid
minicoli - F1C2-pyr
minicoli - F1C2_Fe_starve
minicoli - F1C2_LB
minicoli - F1C2_N_starve
minicoli - F1C2_cyt
minicoli - F1C2_fruc
minicoli - F1C2_glc
minicoli - F1C2_glyc
minicoli - F1C2_osm
minicoli - F1C2_ph5
minicoli - F1C2_sorb
minicoli - F1C2_urid
oxidative - deloxyr_pq
oxidative - delsoxr_pq
oxidative - delsoxs_pq
oxidative - wt_pq
RpoH Gene Weight
Gene Start
1e6
Amino acid transport and metabolism
Carbohydrate transport and metabolism
Energy production and conversion
Function unknown
Inorganic ion transport and metabolism
Nucleotide transport and metabolism
Post-translational modification, protein turnover,and chaperones
Replication, recombination and repair
Signal transduction mechanisms
Transcription
Translation, ribosomal structure and biogenesis

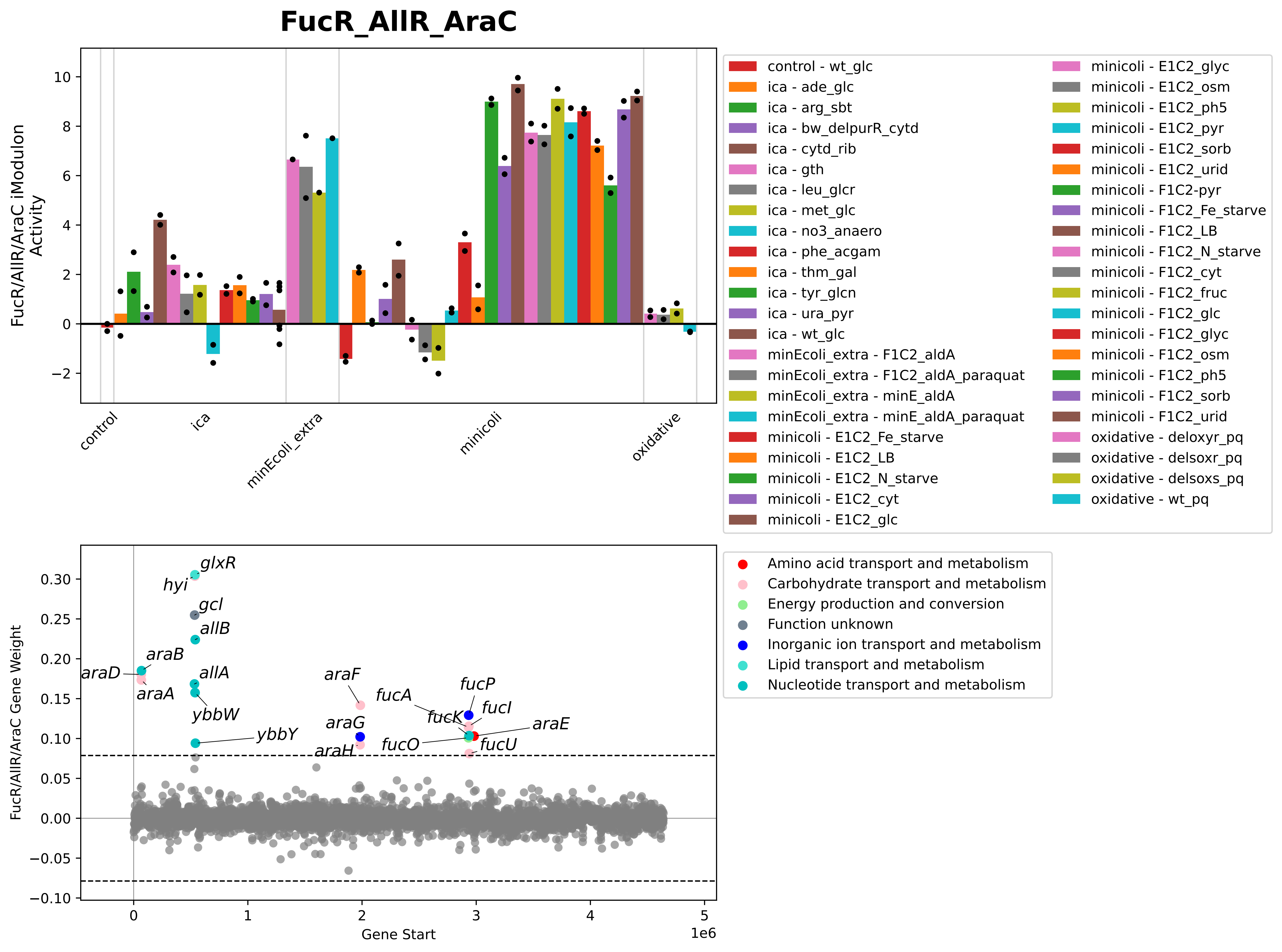
FucR_AllR_AraC
FucR/AllR/AraC iModulon Activity
control
ica
minEcoli_extra
minicoli
oxidative
control - wt_glc
ica - ade_glc
ica - arg_sbt
ica - bw_delpurR_cytd
ica - cytd_rib
ica - gth
ica - leu_glcr
ica - met_glc
ica - no3_anaero
ica - phe_acgam
ica - thm_gal
ica - tyr_glcn
ica - ura_pyr
ica - wt_glc
minEcoli_extra - F1C2_aldA
minEcoli_extra - F1C2_aldA_paraquat
minEcoli_extra - minE_aldA
minEcoli_extra - minE_aldA_paraquat
minicoli - E1C2_Fe_starve
minicoli - E1C2_LB
minicoli - E1C2_N_starve
minicoli - E1C2_cyt
minicoli - E1C2_glc
minicoli - E1C2_glyc
minicoli - E1C2_osm
minicoli - E1C2_ph5
minicoli - E1C2_pyr
minicoli - E1C2_sorb
minicoli - E1C2_urid
minicoli - F1C2-pyr
minicoli - F1C2_Fe_starve
minicoli - F1C2_LB
minicoli - F1C2_N_starve
minicoli - F1C2_cyt
minicoli - F1C2_fruc
minicoli - F1C2_glc
minicoli - F1C2_glyc
minicoli - F1C2_osm
minicoli - F1C2_ph5
minicoli - F1C2_sorb
minicoli - F1C2_urid
oxidative - deloxyr_pq
oxidative - delsoxr_pq
oxidative - delsoxs_pq
oxidative - wt_pq
FucR/AllR/AraC Gene Weight
Gene Start
1e6
glxR
hyi
gcl
allB
araB
araD
araA
allA
ybbW
ybbY
araF
araG
araH
fucA
fucP
fucI
fucK
araE
fucO
fucU
Amino acid transport and metabolism
Carbohydrate transport and metabolism
Energy production and conversion
Function unknown
Inorganic ion transport and metabolism
Lipid transport and metabolism
Nucleotide transport and metabolism

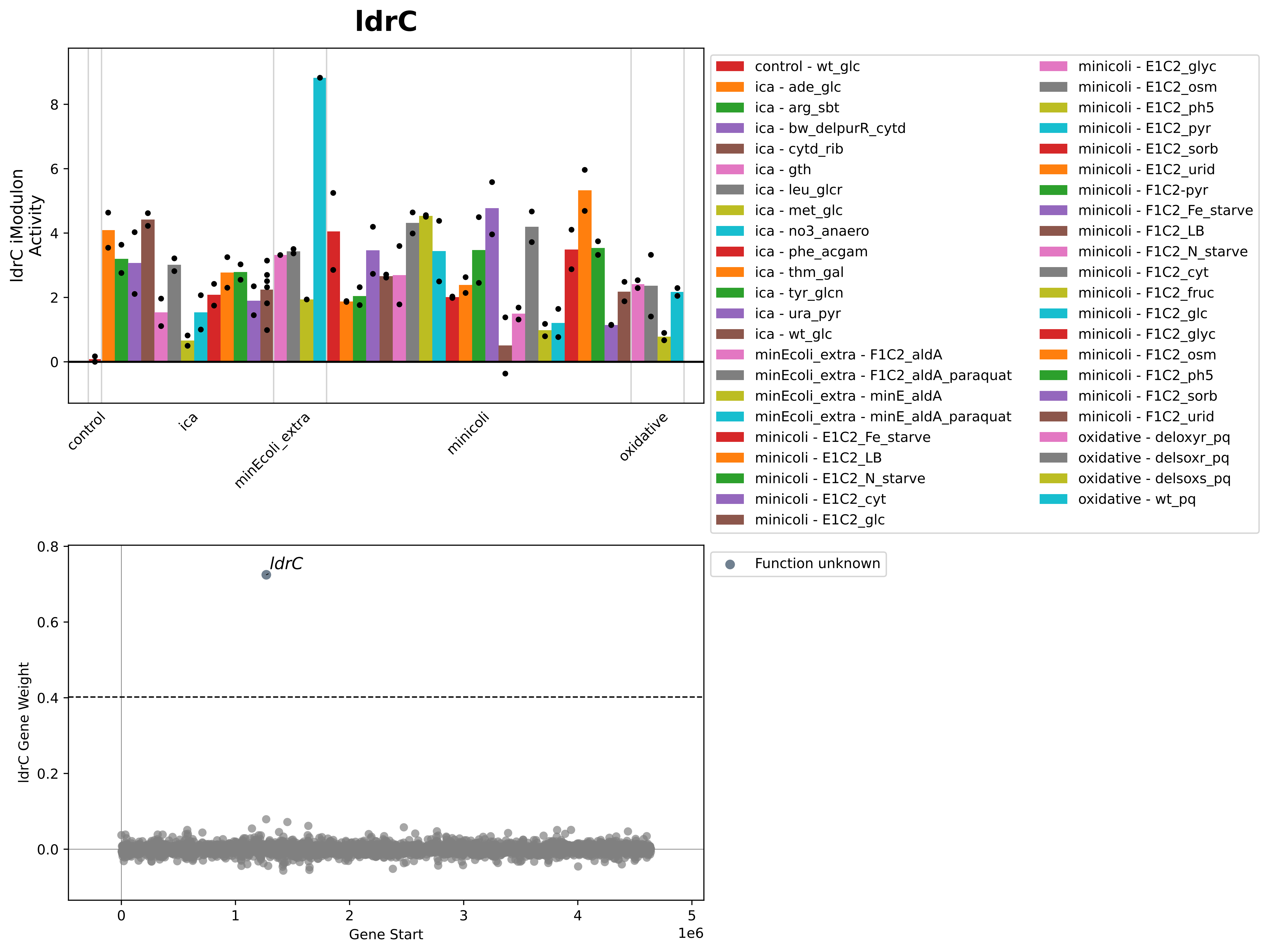
ldrC
ldrC iModulon Activity
control
ica
minEcoli_extra
minicoli
oxidative
control - wt_glc
ica - ade_glc
ica - arg_sbt
ica - bw_delpurR_cytd
ica - cytd_rib
ica - gth
ica - leu_glcr
ica - met_glc
ica - no3_anaero
ica - phe_acgam
ica - thm_gal
ica - tyr_glcn
ica - ura_pyr
ica - wt_glc
minEcoli_extra - F1C2_aldA
minEcoli_extra - F1C2_aldA_paraquat
minEcoli_extra - minE_aldA
minEcoli_extra - minE_aldA_paraquat
minicoli - E1C2_Fe_starve
minicoli - E1C2_LB
minicoli - E1C2_N_starve
minicoli - E1C2_cyt
minicoli - E1C2_glc
minicoli - E1C2_glyc
minicoli - E1C2_osm
minicoli - E1C2_ph5
minicoli - E1C2_pyr
minicoli - E1C2_sorb
minicoli - E1C2_urid
minicoli - F1C2-pyr
minicoli - F1C2_Fe_starve
minicoli - F1C2_LB
minicoli - F1C2_N_starve
minicoli - F1C2_cyt
minicoli - F1C2_fruc
minicoli - F1C2_glc
minicoli - F1C2_glyc
minicoli - F1C2_osm
minicoli - F1C2_ph5
minicoli - F1C2_sorb
minicoli - F1C2_urid
oxidative - deloxyr_pq
oxidative - delsoxr_pq
oxidative - delsoxs_pq
oxidative - wt_pq
ldrC
Function unknown
ldrC Gene Weight
Gene Start
1e6

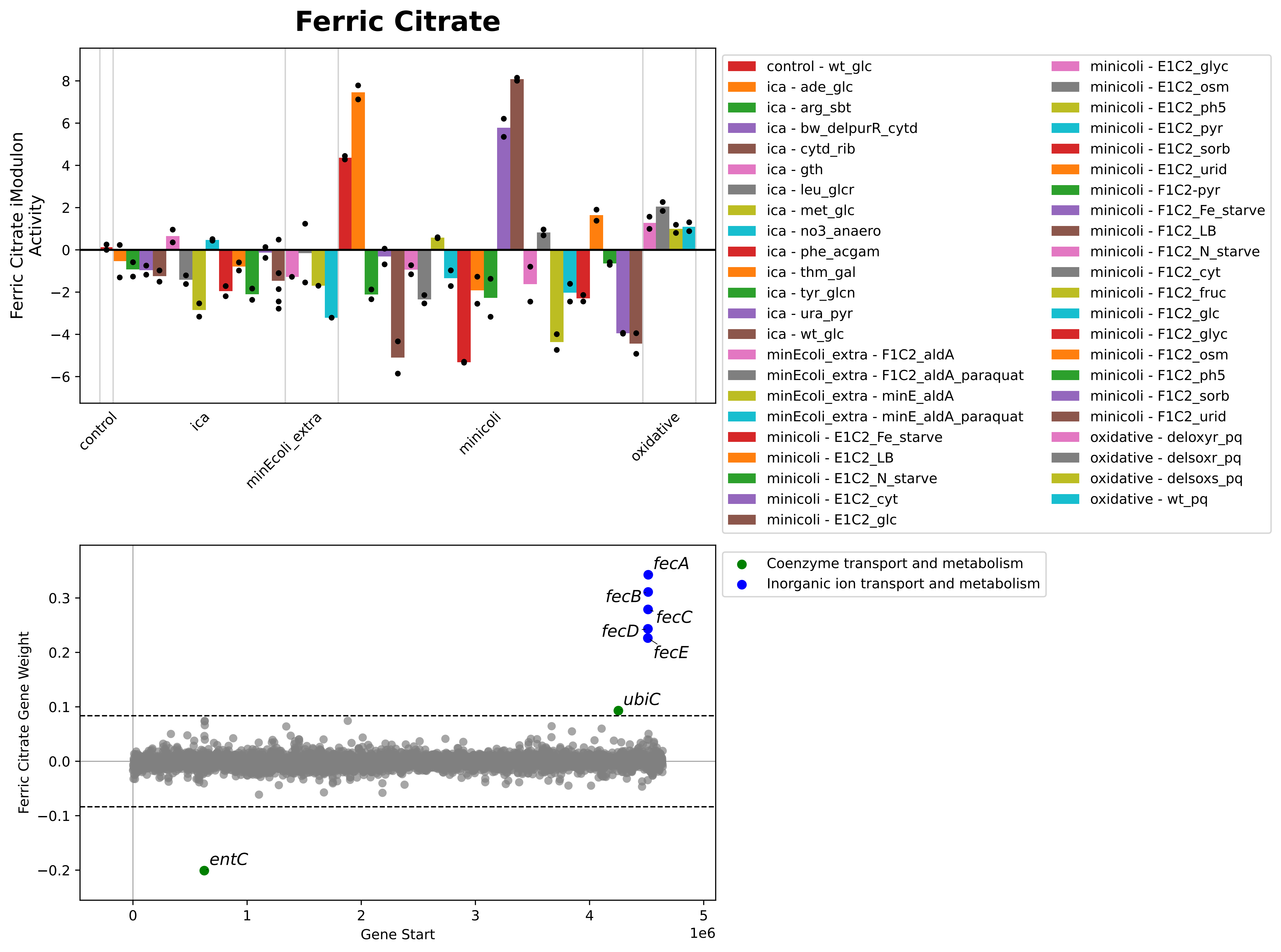

Ferric Citrate
Ferric Citrate iModulon Activity
control
ica
minEcoli_extra
minicoli
oxidative
control - wt_glc
ica - ade_glc
ica - arg_sbt
ica - bw_delpurR_cytd
ica - cytd_rib
ica - gth
ica - leu_glcr
ica - met_glc
ica - no3_anaero
ica - phe_acgam
ica - thm_gal
ica - tyr_glcn
ica - ura_pyr
ica - wt_glc
minEcoli_extra - F1C2_aldA
minEcoli_extra - F1C2_aldA_paraquat
minEcoli_extra - minE_aldA
minEcoli_extra - minE_aldA_paraquat
minicoli - E1C2_Fe_starve
minicoli - E1C2_LB
minicoli - E1C2_N_starve
minicoli - E1C2_cyt
minicoli - E1C2_glc
minicoli - E1C2_glyc
minicoli - E1C2_osm
minicoli - E1C2_ph5
minicoli - E1C2_pyr
minicoli - E1C2_sorb
minicoli - E1C2_urid
minicoli - F1C2-pyr
minicoli - F1C2_Fe_starve
minicoli - F1C2_LB
minicoli - F1C2_N_starve
minicoli - F1C2_cyt
minicoli - F1C2_fruc
minicoli - F1C2_glc
minicoli - F1C2_glyc
minicoli - F1C2_osm
minicoli - F1C2_ph5
minicoli - F1C2_sorb
minicoli - F1C2_urid
oxidative - deloxyr_pq
oxidative - delsoxr_pq
oxidative - delsoxs_pq
oxidative - wt_pq
Ferric Citrate Gene Weight
Gene Start
1e6
fecA
fecB
fecC
fecD
fecE
ubiC
entC
Coenzyme transport and metabolism
Inorganic ion transport and metabolism

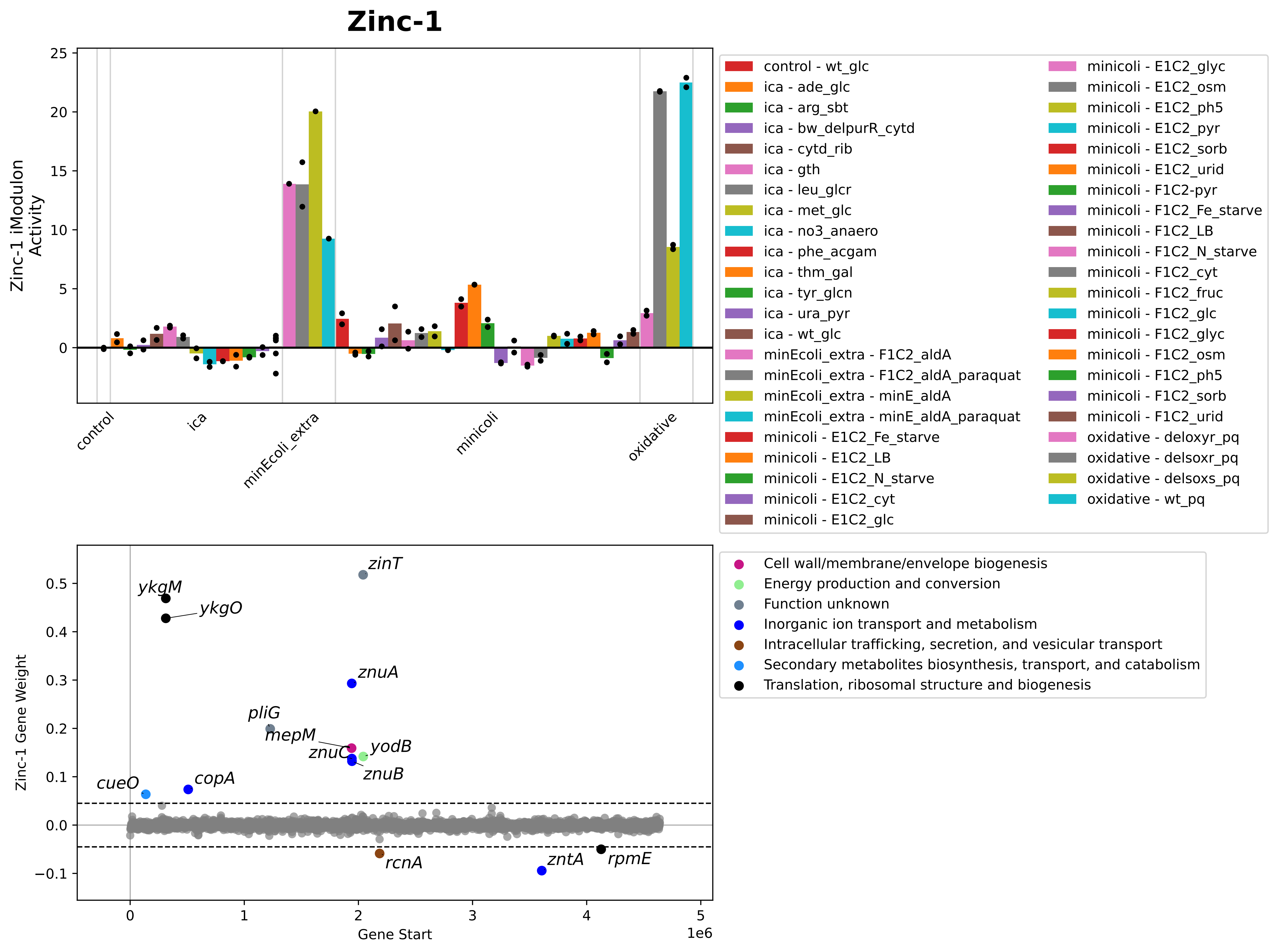

Zinc-1
Zinc-1 iModulon Activity
25
20
15
10
5
0
control
ica
minEcoli_extra
minicoli
oxidative
control - wt_glc
ica - ade_glc
ica - arg_sbt
ica - bw_delpurR_cytd
ica - cytd_rib
ica - gth
ica - leu_glcr
ica - met_glc
ica - no3_anaero
ica - phe_acgam
ica - thm_gal
ica - tyr_glcn
ica - ura_pyr
ica - wt_glc
minEcoli_extra - F1C2_aldA
minEcoli_extra - F1C2_aldA_paraquat
minEcoli_extra - minE_aldA
minEcoli_extra - minE_aldA_paraquat
minicoli - E1C2_Fe_starve
minicoli - E1C2_LB
minicoli - E1C2_N_starve
minicoli - E1C2_cyt
minicoli - E1C2_glc
minicoli - E1C2_glyc
minicoli - E1C2_osm
minicoli - E1C2_ph5
minicoli - E1C2_pyr
minicoli - E1C2_sorb
minicoli - E1C2_urid
minicoli - F1C2-pyr
minicoli - F1C2_Fe_starve
minicoli - F1C2_LB
minicoli - F1C2_N_starve
minicoli - F1C2_cyt
minicoli - F1C2_fruc
minicoli - F1C2_glc
minicoli - F1C2_glyc
minicoli - F1C2_osm
minicoli - F1C2_ph5
minicoli - F1C2_sorb
minicoli - F1C2_urid
oxidative - deloxyr_pq
oxidative - delsoxr_pq
oxidative - delsoxs_pq
oxidative - wt_pq
Zinc-1 Gene Weight
0.5
0.4
0.3
0.2
0.1
0.0
−0.1
ykgM
ykgO
zinT
znuA
pliG
mepM
znuC
yodB
znuB
cueO
copA
rcnA
zntA
rpmE
0
1
2
3
4
5
Gene Start
1e6
Cell wall/membrane/envelope biogenesis
Energy production and conversion
Function unknown
Inorganic ion transport and metabolism
Intracellular trafficking, secretion, and vesicular transport
Secondary metabolites biosynthesis, transport, and catabolism
Translation, ribosomal structure and biogenesis

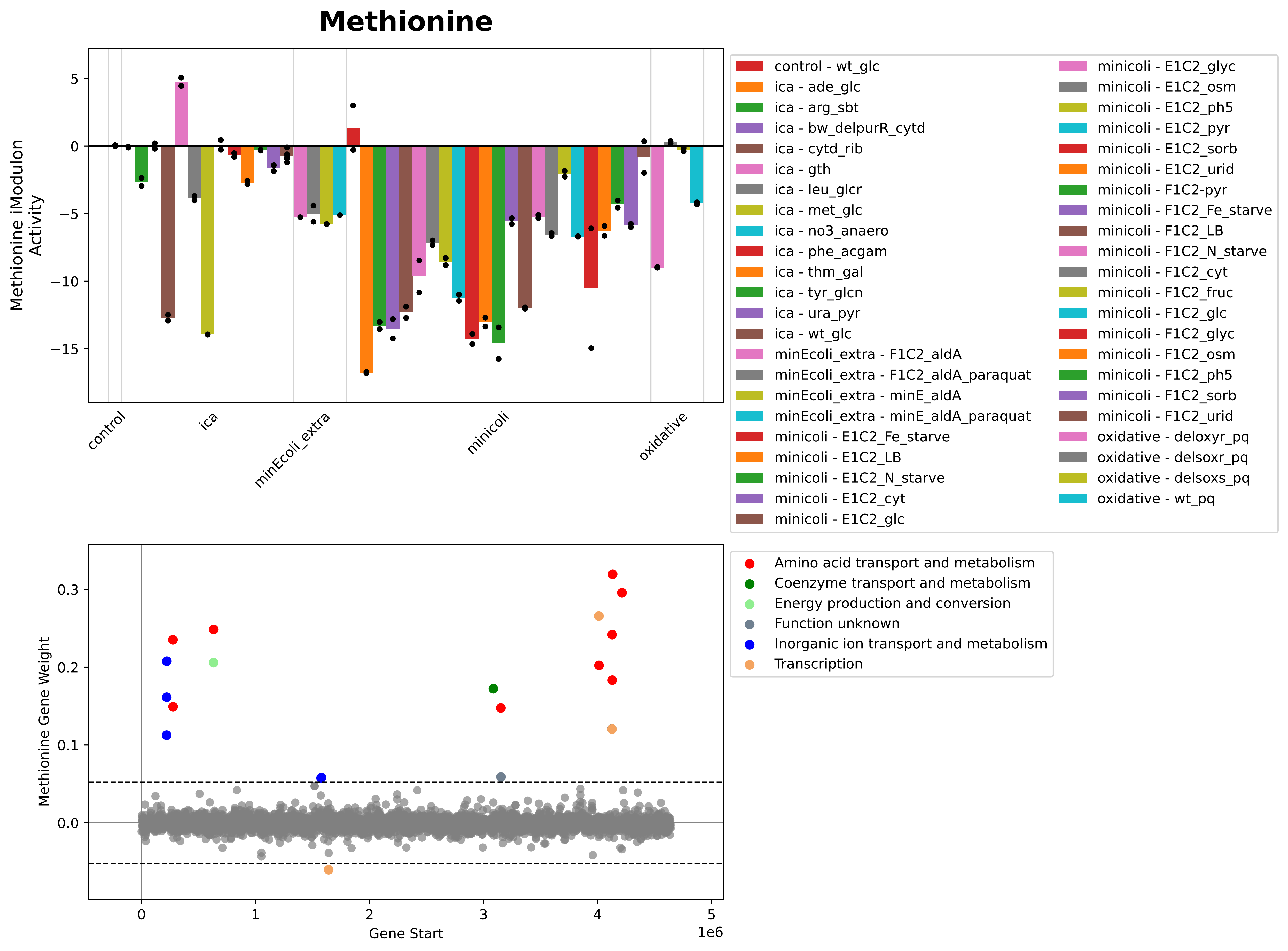

Methionine
Methionine iModulon Activity
control
ica
minEcoli_extra
minicoli
oxidative
control - wt_glc
ica - ade_glc
ica - arg_sbt
ica - bw_delpurR_cytd
ica - cytd_rib
ica - gth
ica - leu_glcr
ica - met_glc
ica - no3_anaero
ica - phe_acgam
ica - thm_gal
ica - tyr_glcn
ica - ura_pyr
ica - wt_glc
minEcoli_extra - F1C2_aldA
minEcoli_extra - F1C2_aldA_paraquat
minEcoli_extra - minE_aldA
minEcoli_extra - minE_aldA_paraquat
minicoli - E1C2_Fe_starve
minicoli - E1C2_LB
minicoli - E1C2_N_starve
minicoli - E1C2_cyt
minicoli - E1C2_glc
minicoli - E1C2_glyc
minicoli - E1C2_osm
minicoli - E1C2_ph5
minicoli - E1C2_pyr
minicoli - E1C2_sorb
minicoli - E1C2_urid
minicoli - F1C2-pyr
minicoli - F1C2_Fe_starve
minicoli - F1C2_LB
minicoli - F1C2_N_starve
minicoli - F1C2_cyt
minicoli - F1C2_fruc
minicoli - F1C2_glc
minicoli - F1C2_glyc
minicoli - F1C2_osm
minicoli - F1C2_ph5
minicoli - F1C2_sorb
minicoli - F1C2_urid
oxidative - deloxyr_pq
oxidative - delsoxr_pq
oxidative - delsoxs_pq
oxidative - wt_pq
Methionine Gene Weight
Gene Start
1e6
Amino acid transport and metabolism
Coenzyme transport and metabolism
Energy production and conversion
Function unknown
Inorganic ion transport and metabolism
Transcription

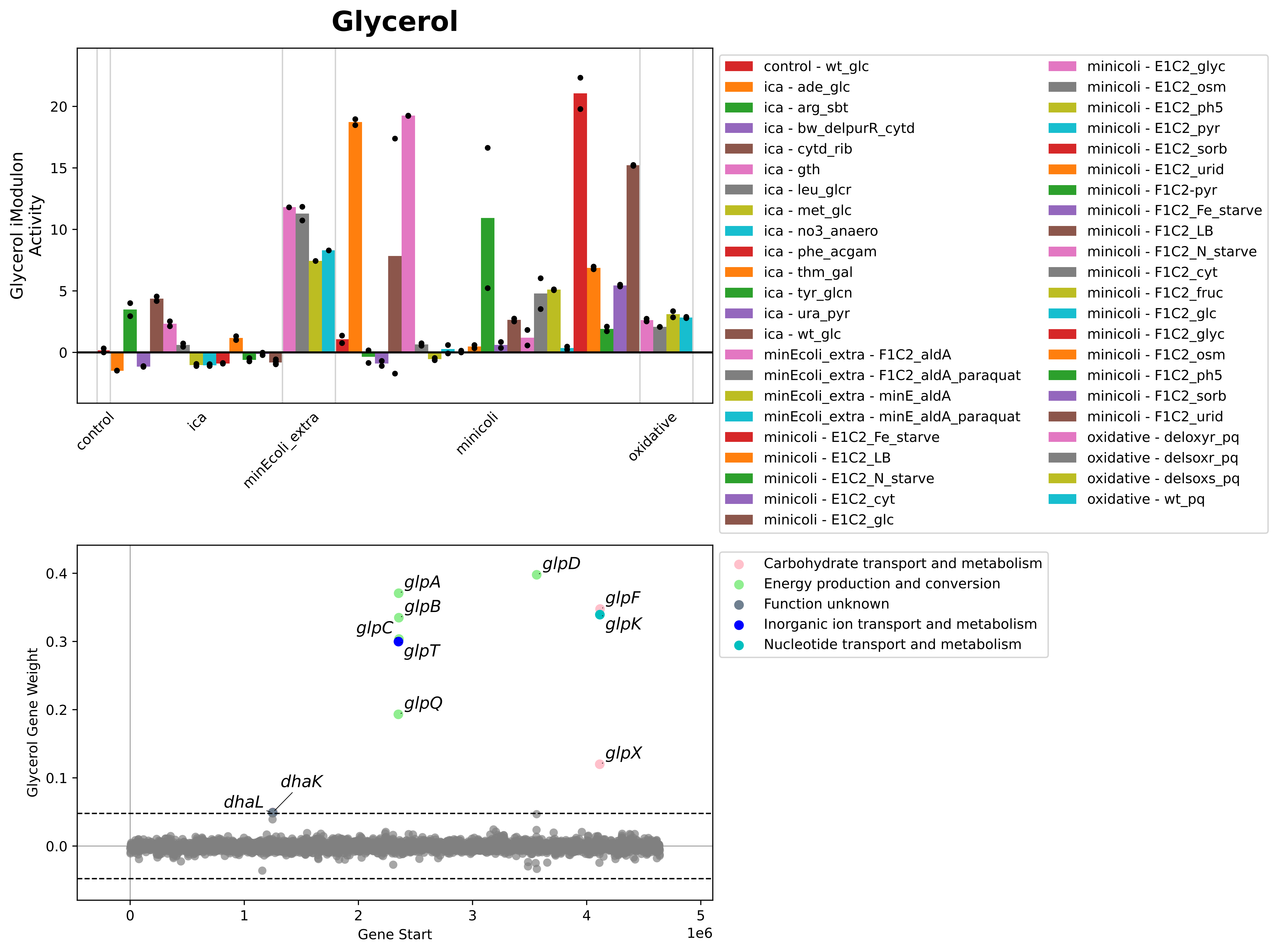
Glycerol
Glycerol iModulon Activity
control
ica
minEcoli_extra
minicoli
oxidative
control - wt_glc
ica - ade_glc
ica - arg_sbt
ica - bw_delpurR_cytd
ica - cytd_rib
ica - gth
ica - leu_glcr
ica - met_glc
ica - no3_anaero
ica - phe_acgam
ica - thm_gal
ica - tyr_glcn
ica - ura_pyr
ica - wt_glc
minEcoli_extra - F1C2_aldA
minEcoli_extra - F1C2_aldA_paraquat
minEcoli_extra - minE_aldA
minEcoli_extra - minE_aldA_paraquat
minicoli - E1C2_Fe_starve
minicoli - E1C2_LB
minicoli - E1C2_N_starve
minicoli - E1C2_cyt
minicoli - E1C2_glc
minicoli - E1C2_glyc
minicoli - E1C2_osm
minicoli - E1C2_ph5
minicoli - E1C2_pyr
minicoli - E1C2_sorb
minicoli - E1C2_urid
minicoli - F1C2-pyr
minicoli - F1C2_Fe_starve
minicoli - F1C2_LB
minicoli - F1C2_N_starve
minicoli - F1C2_cyt
minicoli - F1C2_fruc
minicoli - F1C2_glc
minicoli - F1C2_glyc
minicoli - F1C2_osm
minicoli - F1C2_ph5
minicoli - F1C2_sorb
minicoli - F1C2_urid
oxidative - deloxyr_pq
oxidative - delsoxr_pq
oxidative - delsoxs_pq
oxidative - wt_pq
Glycerol Gene Weight
Gene Start
1e6
glpD
glpA
glpB
glpC
glpT
glpF
glpK
glpQ
glpX
dhaK
dhaL
Carbohydrate transport and metabolism
Energy production and conversion
Function unknown
Inorganic ion transport and metabolism
Nucleotide transport and metabolism

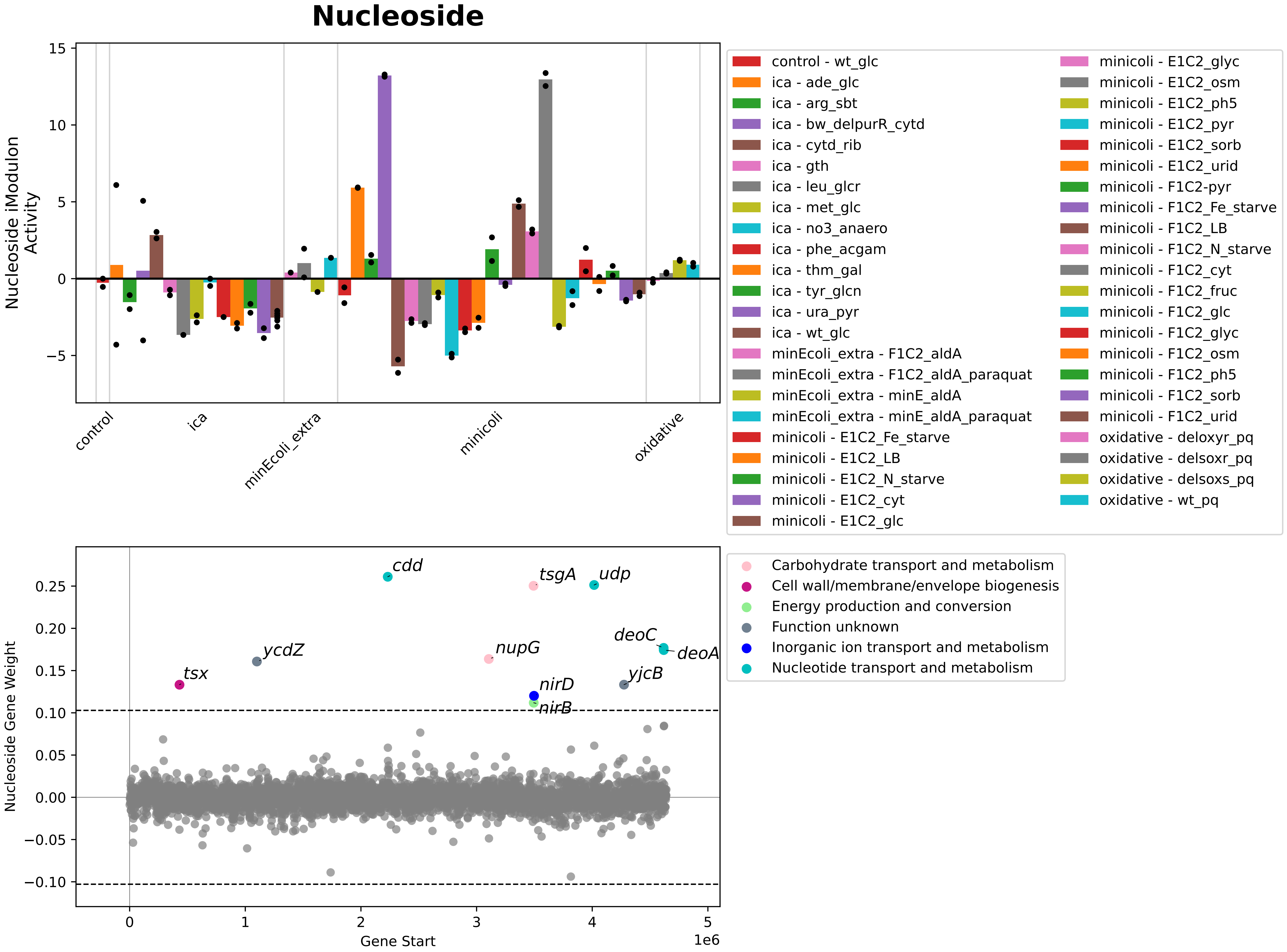

Nucleoside
Nucleoside iModulon Activity
control
ica
minEcoli_extra
minicoli
oxidative
control - wt_glc
ica - ade_glc
ica - arg_sbt
ica - bw_delpurR_cytd
ica - cytd_rib
ica - gth
ica - leu_glcr
ica - met_glc
ica - no3_anaero
ica - phe_acgam
ica - thm_gal
ica - tyr_glcn
ica - ura_pyr
ica - wt_glc
minEcoli_extra - F1C2_aldA
minEcoli_extra - F1C2_aldA_paraquat
minEcoli_extra - minE_aldA
minEcoli_extra - minE_aldA_paraquat
minicoli - E1C2_Fe_starve
minicoli - E1C2_LB
minicoli - E1C2_N_starve
minicoli - E1C2_cyt
minicoli - E1C2_glc
minicoli - E1C2_glyc
minicoli - E1C2_osm
minicoli - E1C2_ph5
minicoli - E1C2_pyr
minicoli - E1C2_sorb
minicoli - E1C2_urid
minicoli - F1C2-pyr
minicoli - F1C2_Fe_starve
minicoli - F1C2_LB
minicoli - F1C2_N_starve
minicoli - F1C2_cyt
minicoli - F1C2_fruc
minicoli - F1C2_glc
minicoli - F1C2_glyc
minicoli - F1C2_osm
minicoli - F1C2_ph5
minicoli - F1C2_sorb
minicoli - F1C2_urid
oxidative - deloxyr_pq
oxidative - delsoxr_pq
oxidative - delsoxs_pq
oxidative - wt_pq
Nucleoside Gene Weight
Gene Start
1e6
cdd
tsgA
udp
deoC
deoA
ycdZ
nupG
tsx
yjcB
nirD
nirB
Carbohydrate transport and metabolism
Cell wall/membrane/envelope biogenesis
Energy production and conversion
Function unknown
Inorganic ion transport and metabolism
Nucleotide transport and metabolism

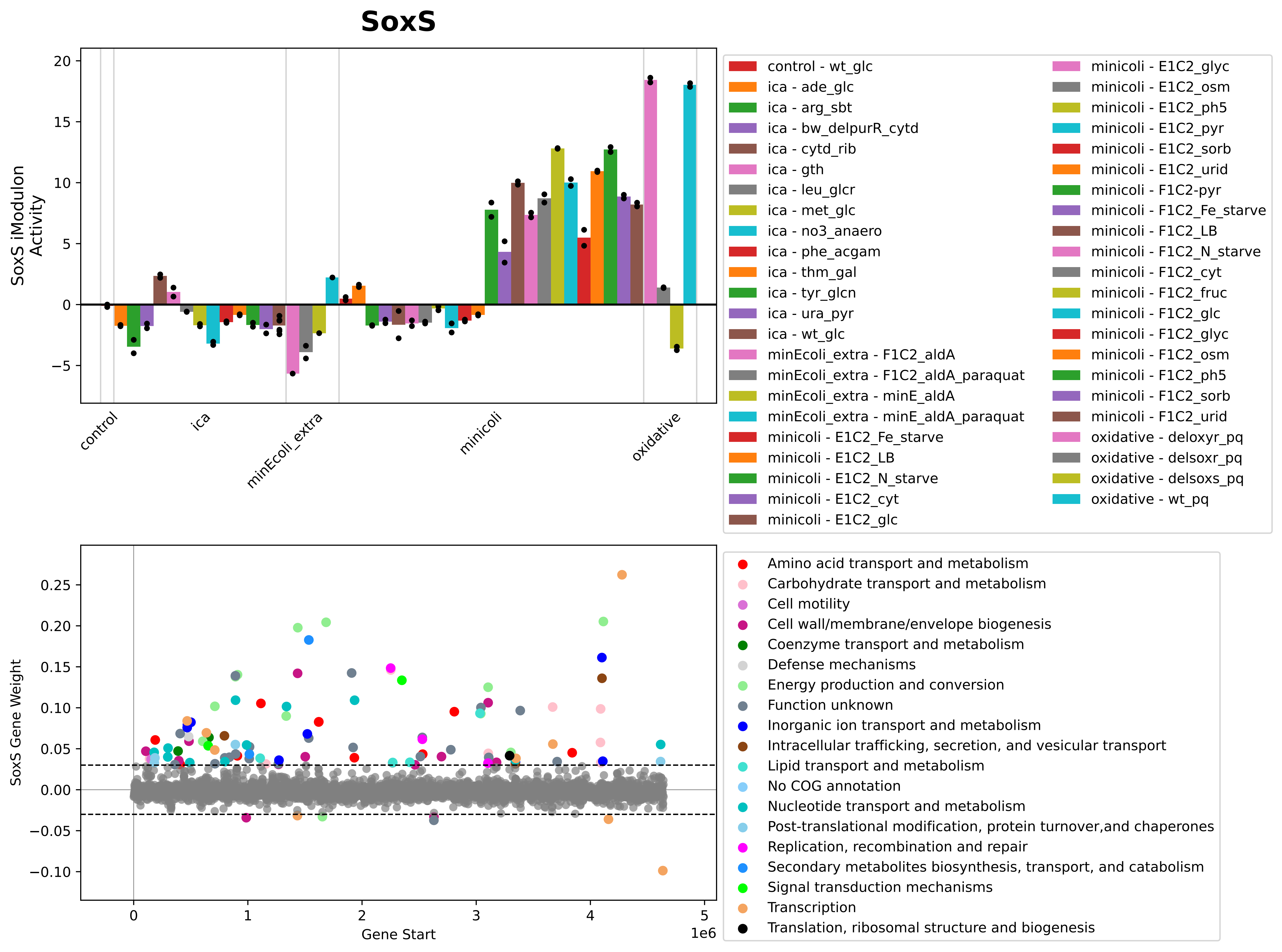

SoxS
SoxS iModulon Activity
20
15
10
5
0
−5
control
ica
minEcoli_extra
minicoli
oxidative
control - wt_glc
ica - ade_glc
ica - arg_sbt
ica - bw_delpurR_cytd
ica - cytd_rib
ica - gth
ica - leu_glcr
ica - met_glc
ica - no3_anaero
ica - phe_acgam
ica - thm_gal
ica - tyr_glcn
ica - ura_pyr
ica - wt_glc
minEcoli_extra - F1C2_aldA
minEcoli_extra - F1C2_aldA_paraquat
minEcoli_extra - minE_aldA
minEcoli_extra - minE_aldA_paraquat
minicoli - E1C2_Fe_starve
minicoli - E1C2_LB
minicoli - E1C2_N_starve
minicoli - E1C2_cyt
minicoli - E1C2_glc
minicoli - E1C2_glyc
minicoli - E1C2_osm
minicoli - E1C2_ph5
minicoli - E1C2_pyr
minicoli - E1C2_sorb
minicoli - E1C2_urid
minicoli - F1C2-pyr
minicoli - F1C2_Fe_starve
minicoli - F1C2_LB
minicoli - F1C2_N_starve
minicoli - F1C2_cyt
minicoli - F1C2_fruc
minicoli - F1C2_glc
minicoli - F1C2_glyc
minicoli - F1C2_osm
minicoli - F1C2_ph5
minicoli - F1C2_sorb
minicoli - F1C2_urid
oxidative - deloxyr_pq
oxidative - delsoxr_pq
oxidative - delsoxs_pq
oxidative - wt_pq
SoxS Gene Weight
0.25
0.20
0.15
0.10
0.05
0.00
−0.05
−0.10
0
1
2
3
4
5
Gene Start
1e6
Amino acid transport and metabolism
Carbohydrate transport and metabolism
Cell motility
Cell wall/membrane/envelope biogenesis
Coenzyme transport and metabolism
Defense mechanisms
Energy production and conversion
Function unknown
Inorganic ion transport and metabolism
Intracellular trafficking, secretion, and vesicular transport
Lipid transport and metabolism
No COG annotation
Nucleotide transport and metabolism
Post-translational modification, protein turnover,and chaperones
Replication, recombination and repair
Secondary metabolites biosynthesis, transport, and catabolism
Signal transduction mechanisms
Transcription
Translation, ribosomal structure and biogenesis

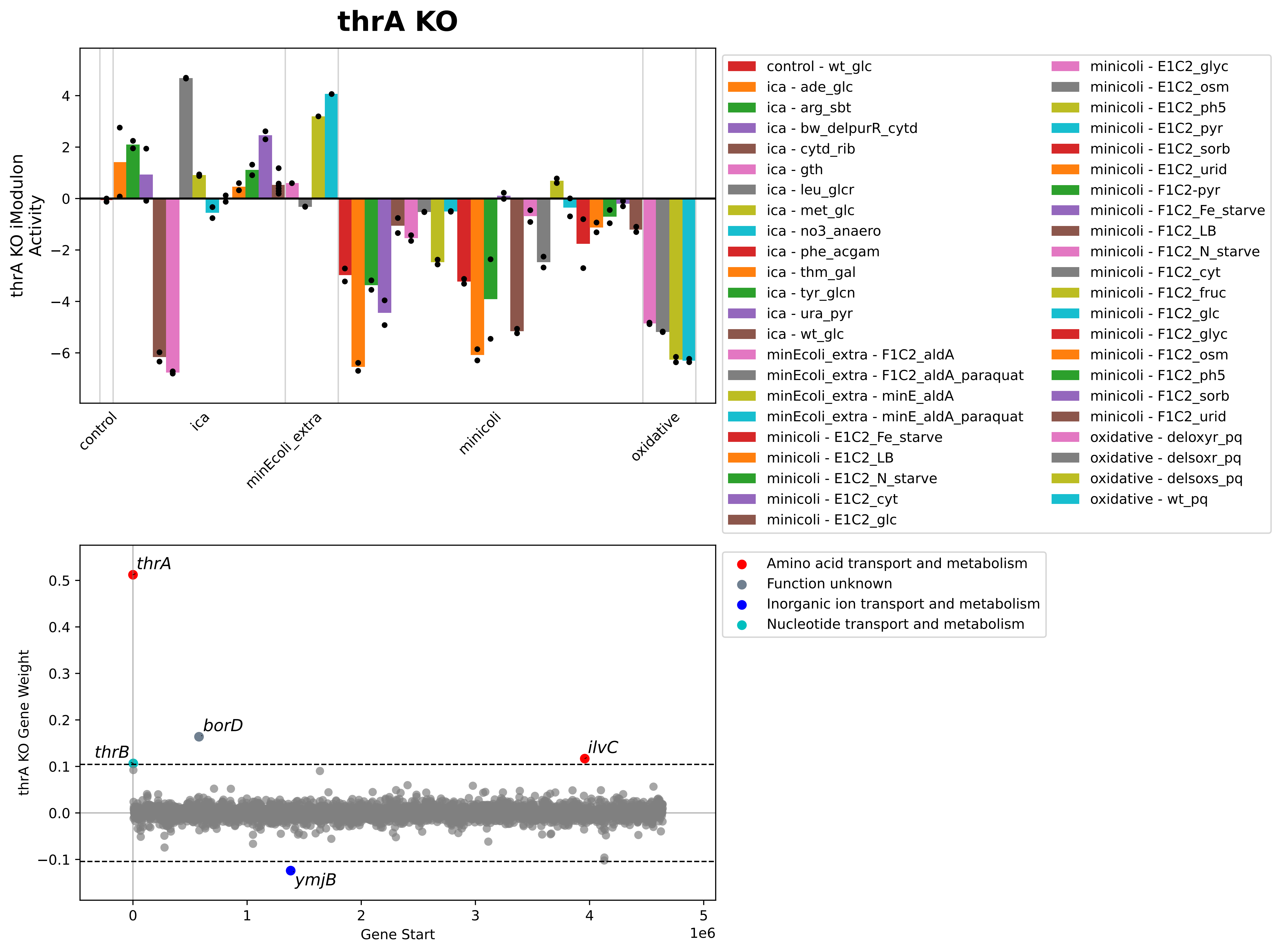

thrA KO
thrA KO iModulon Activity
control
ica
minEcoli_extra
minicoli
oxidative
control - wt_glc
ica - ade_glc
ica - arg_sbt
ica - bw_delpurR_cytd
ica - cytd_rib
ica - gth
ica - leu_glcr
ica - met_glc
ica - no3_anaero
ica - phe_acgam
ica - thm_gal
ica - tyr_glcn
ica - ura_pyr
ica - wt_glc
minEcoli_extra - F1C2_aldA
minEcoli_extra - F1C2_aldA_paraquat
minEcoli_extra - minE_aldA
minEcoli_extra - minE_aldA_paraquat
minicoli - E1C2_Fe_starve
minicoli - E1C2_LB
minicoli - E1C2_N_starve
minicoli - E1C2_cyt
minicoli - E1C2_glc
minicoli - E1C2_glyc
minicoli - E1C2_osm
minicoli - E1C2_ph5
minicoli - E1C2_pyr
minicoli - E1C2_sorb
minicoli - E1C2_urid
minicoli - F1C2-pyr
minicoli - F1C2_Fe_starve
minicoli - F1C2_LB
minicoli - F1C2_N_starve
minicoli - F1C2_cyt
minicoli - F1C2_fruc
minicoli - F1C2_glc
minicoli - F1C2_glyc
minicoli - F1C2_osm
minicoli - F1C2_ph5
minicoli - F1C2_sorb
minicoli - F1C2_urid
oxidative - deloxyr_pq
oxidative - delsoxr_pq
oxidative - delsoxs_pq
oxidative - wt_pq
thrA
borD
thrB
ilvC
ymjB
thrA KO Gene Weight
Gene Start
1e6
Amino acid transport and metabolism
Function unknown
Inorganic ion transport and metabolism
Nucleotide transport and metabolism

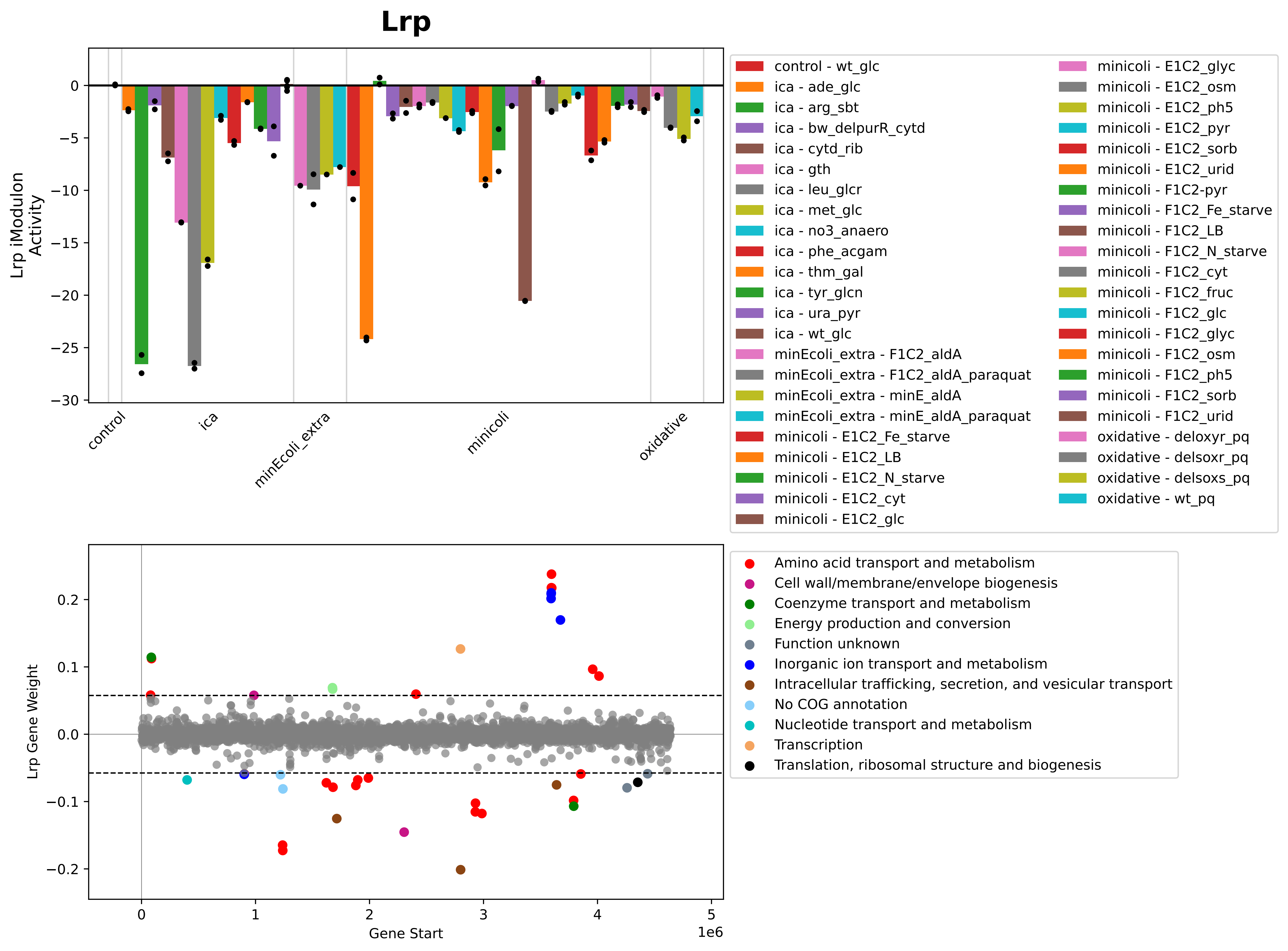
Lrp
Lrp iModulon Activity
control
ica
minEcoli_extra
minicoli
oxidative
control - wt_glc
ica - ade_glc
ica - arg_sbt
ica - bw_delpurR_cytd
ica - cytd_rib
ica - gth
ica - leu_glcr
ica - met_glc
ica - no3_anaero
ica - phe_acgam
ica - thm_gal
ica - tyr_glcn
ica - ura_pyr
ica - wt_glc
minEcoli_extra - F1C2_aldA
minEcoli_extra - F1C2_aldA_paraquat
minEcoli_extra - minE_aldA
minEcoli_extra - minE_aldA_paraquat
minicoli - E1C2_Fe_starve
minicoli - E1C2_LB
minicoli - E1C2_N_starve
minicoli - E1C2_cyt
minicoli - E1C2_glc
minicoli - E1C2_glyc
minicoli - E1C2_osm
minicoli - E1C2_ph5
minicoli - E1C2_pyr
minicoli - E1C2_sorb
minicoli - E1C2_urid
minicoli - F1C2-pyr
minicoli - F1C2_Fe_starve
minicoli - F1C2_LB
minicoli - F1C2_N_starve
minicoli - F1C2_cyt
minicoli - F1C2_fruc
minicoli - F1C2_glc
minicoli - F1C2_glyc
minicoli - F1C2_osm
minicoli - F1C2_ph5
minicoli - F1C2_sorb
minicoli - F1C2_urid
oxidative - deloxyr_pq
oxidative - delsoxr_pq
oxidative - delsoxs_pq
oxidative - wt_pq
Lrp Gene Weight
Gene Start
1e6
Amino acid transport and metabolism
Cell wall/membrane/envelope biogenesis
Coenzyme transport and metabolism
Energy production and conversion
Function unknown
Inorganic ion transport and metabolism
Intracellular trafficking, secretion, and vesicular transport
No COG annotation
Nucleotide transport and metabolism
Transcription
Translation, ribosomal structure and biogenesis

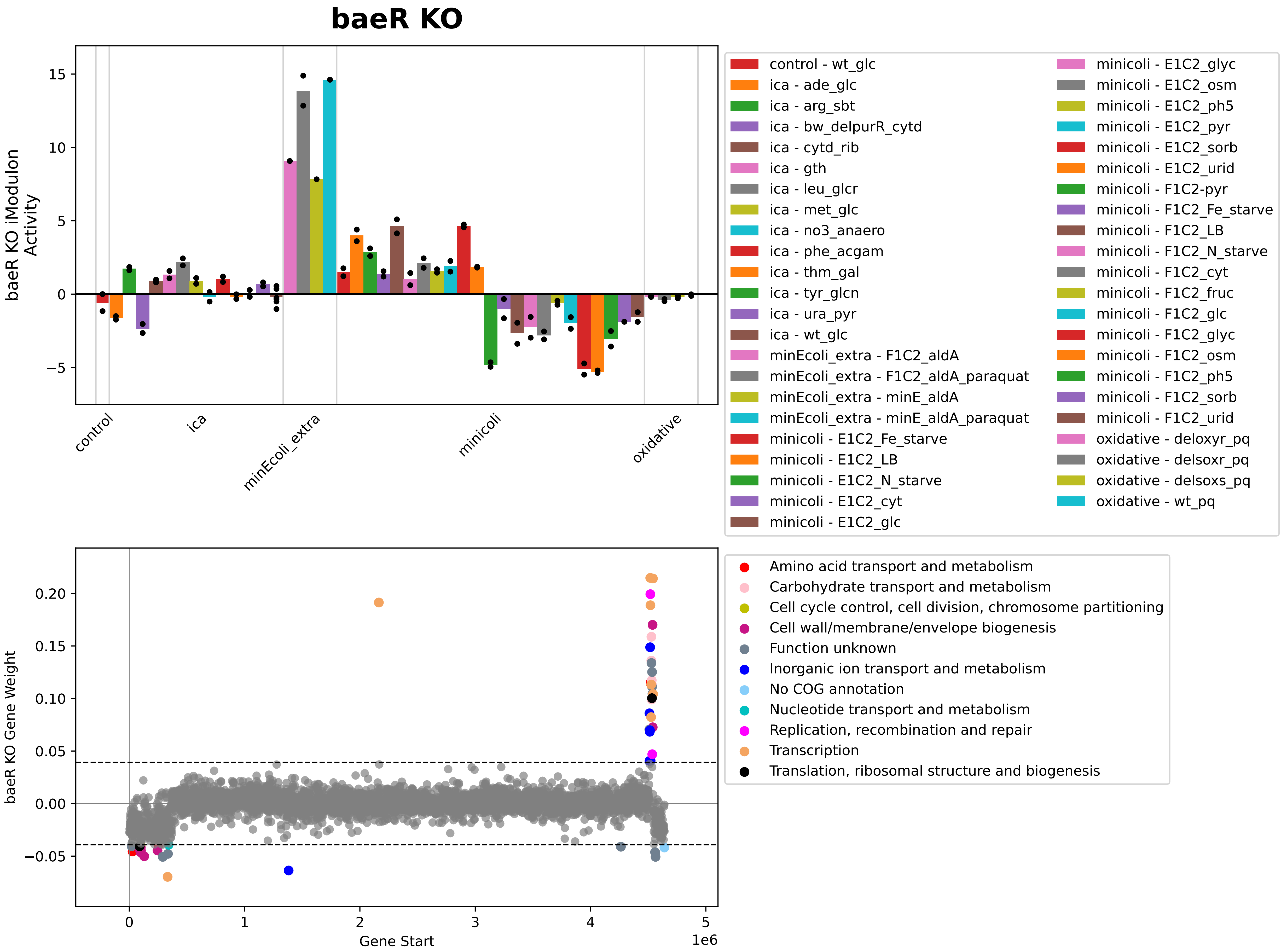
baeR KO
baeR KO iModulon Activity
control
ica
minEcoli_extra
minicoli
oxidative
control - wt_glc
ica - ade_glc
ica - arg_sbt
ica - bw_delpurR_cytd
ica - cytd_rib
ica - gth
ica - leu_glcr
ica - met_glc
ica - no3_anaero
ica - phe_acgam
ica - thm_gal
ica - tyr_glcn
ica - ura_pyr
ica - wt_glc
minEcoli_extra - F1C2_aldA
minEcoli_extra - F1C2_aldA_paraquat
minEcoli_extra - minE_aldA
minEcoli_extra - minE_aldA_paraquat
minicoli - E1C2_Fe_starve
minicoli - E1C2_LB
minicoli - E1C2_N_starve
minicoli - E1C2_cyt
minicoli - E1C2_glc
minicoli - E1C2_glyc
minicoli - E1C2_osm
minicoli - E1C2_ph5
minicoli - E1C2_pyr
minicoli - E1C2_sorb
minicoli - E1C2_urid
minicoli - F1C2-pyr
minicoli - F1C2_Fe_starve
minicoli - F1C2_LB
minicoli - F1C2_N_starve
minicoli - F1C2_cyt
minicoli - F1C2_fruc
minicoli - F1C2_glc
minicoli - F1C2_glyc
minicoli - F1C2_osm
minicoli - F1C2_ph5
minicoli - F1C2_sorb
minicoli - F1C2_urid
oxidative - deloxyr_pq
oxidative - delsoxr_pq
oxidative - delsoxs_pq
oxidative - wt_pq
baeR KO Gene Weight
Gene Start
1e6
Amino acid transport and metabolism
Carbohydrate transport and metabolism
Cell cycle control, cell division, chromosome partitioning
Cell wall/membrane/envelope biogenesis
Function unknown
Inorganic ion transport and metabolism
No COG annotation
Nucleotide transport and metabolism
Replication, recombination and repair
Transcription
Translation, ribosomal structure and biogenesis

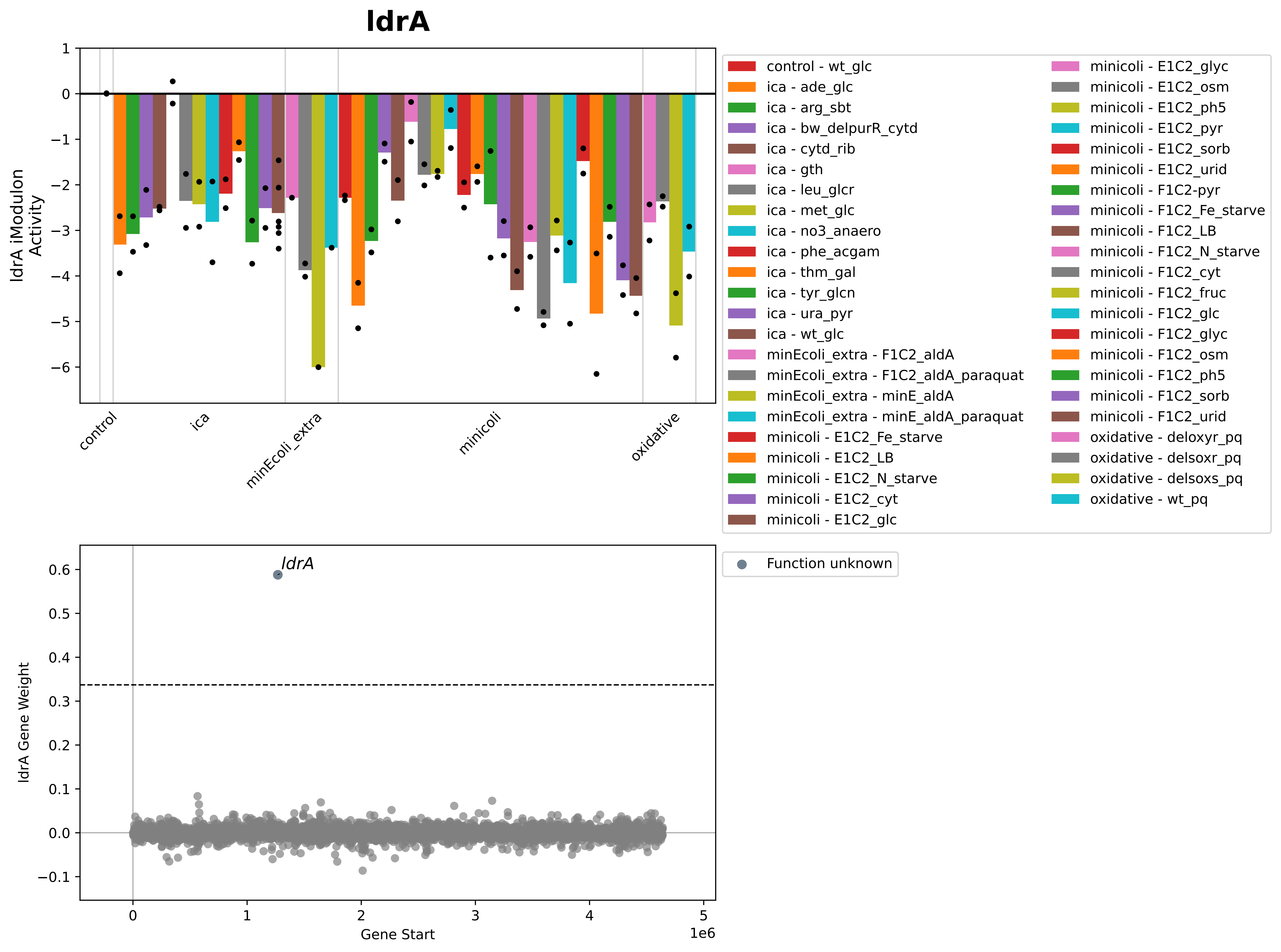

IdrA
IdrA iModulon Activity
control
ica
minEcoli_extra
minicoli
oxidative
control - wt_glc
ica - ade_glc
ica - arg_sbt
ica - bw_delpurR_cytd
ica - cytd_rib
ica - gth
ica - leu_glcr
ica - met_glc
ica - no3_anaero
ica - phe_acgam
ica - thm_gal
ica - tyr_glcn
ica - ura_pyr
ica - wt_glc
minEcoli_extra - F1C2_aldA
minEcoli_extra - F1C2_aldA_paraquat
minEcoli_extra - minE_aldA
minEcoli_extra - minE_aldA_paraquat
minicoli - E1C2_Fe_starve
minicoli - E1C2_LB
minicoli - E1C2_N_starve
minicoli - E1C2_cyt
minicoli - E1C2_glc
minicoli - E1C2_glyc
minicoli - E1C2_osm
minicoli - E1C2_ph5
minicoli - E1C2_pyr
minicoli - E1C2_sorb
minicoli - E1C2_urid
minicoli - F1C2-pyr
minicoli - F1C2_Fe_starve
minicoli - F1C2_LB
minicoli - F1C2_N_starve
minicoli - F1C2_cyt
minicoli - F1C2_fruc
minicoli - F1C2_glc
minicoli - F1C2_glyc
minicoli - F1C2_osm
minicoli - F1C2_ph5
minicoli - F1C2_sorb
minicoli - F1C2_urid
oxidative - deloxyr_pq
oxidative - delsoxr_pq
oxidative - delsoxs_pq
oxidative - wt_pq
IdrA
IdrA Gene Weight
Gene Start
1e6
Function unknown

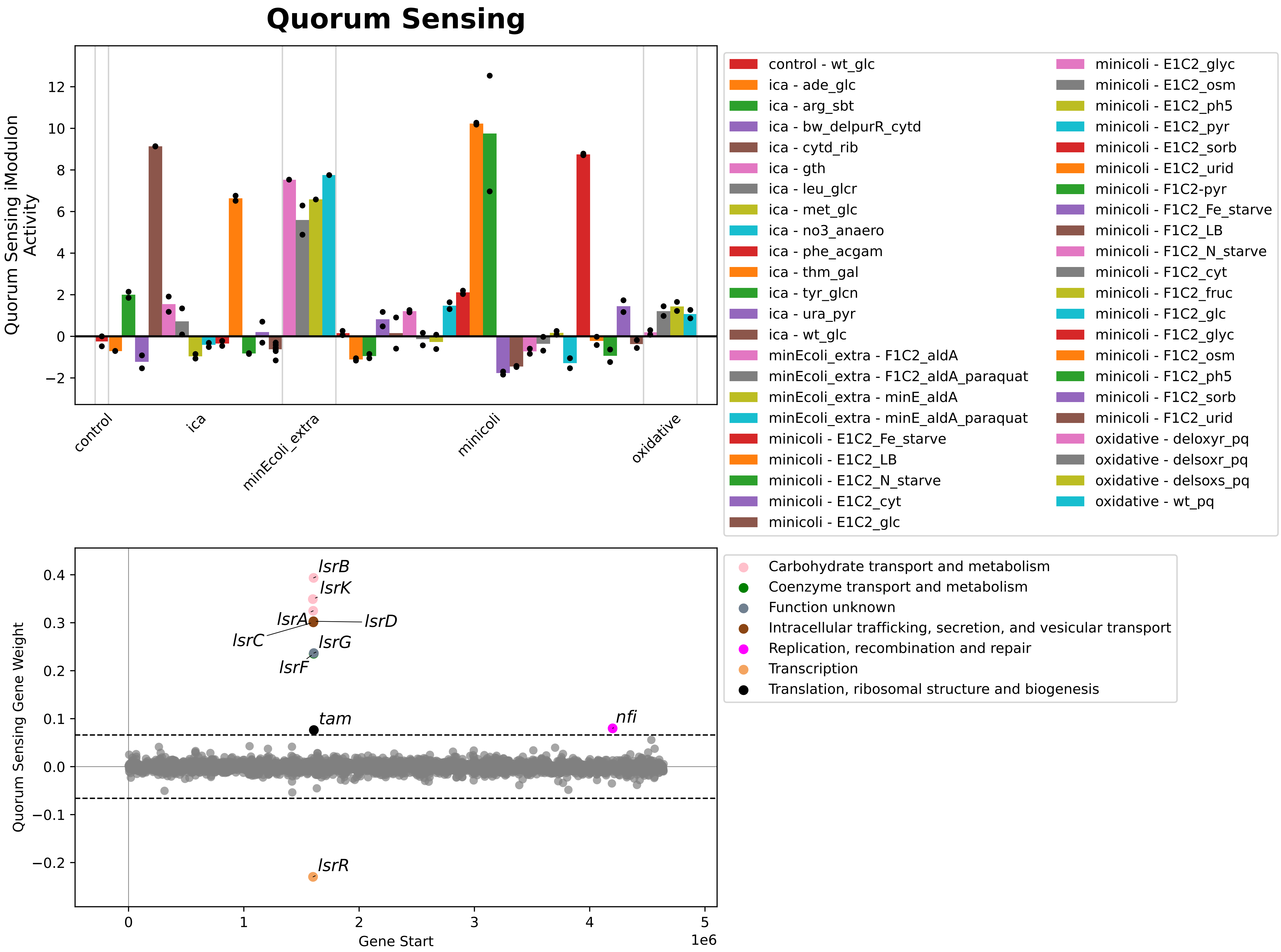

Quorum Sensing
Quorum Sensing iModulon Activity
control
ica
minEcoli_extra
minicoli
oxidative
control - wt_glc
ica - ade_glc
ica - arg_sbt
ica - bw_delpurR_cytd
ica - cytd_rib
ica - gth
ica - leu_glcr
ica - met_glc
ica - no3_anaero
ica - phe_acgam
ica - thm_gal
ica - tyr_glcn
ica - ura_pyr
ica - wt_glc
minEcoli_extra - F1C2_aldA
minEcoli_extra - F1C2_aldA_paraquat
minEcoli_extra - minE_aldA
minEcoli_extra - minE_aldA_paraquat
minicoli - E1C2_Fe_starve
minicoli - E1C2_LB
minicoli - E1C2_N_starve
minicoli - E1C2_cyt
minicoli - E1C2_glc
minicoli - E1C2_glyc
minicoli - E1C2_osm
minicoli - E1C2_ph5
minicoli - E1C2_pyr
minicoli - E1C2_sorb
minicoli - E1C2_urid
minicoli - F1C2-pyr
minicoli - F1C2_Fe_starve
minicoli - F1C2_LB
minicoli - F1C2_N_starve
minicoli - F1C2_cyt
minicoli - F1C2_fruc
minicoli - F1C2_glc
minicoli - F1C2_glyc
minicoli - F1C2_osm
minicoli - F1C2_ph5
minicoli - F1C2_sorb
minicoli - F1C2_urid
oxidative - deloxyr_pq
oxidative - delsoxr_pq
oxidative - delsoxs_pq
oxidative - wt_pq
Quorum Sensing Gene Weight
Gene Start
1e6
lsrB
lsrK
lsrA
lsrD
lsrC
lsrG
lsrF
tam
nfi
lsrR
Carbohydrate transport and metabolism
Coenzyme transport and metabolism
Function unknown
Intracellular trafficking, secretion, and vesicular transport
Replication, recombination and repair
Transcription
Translation, ribosomal structure and biogenesis

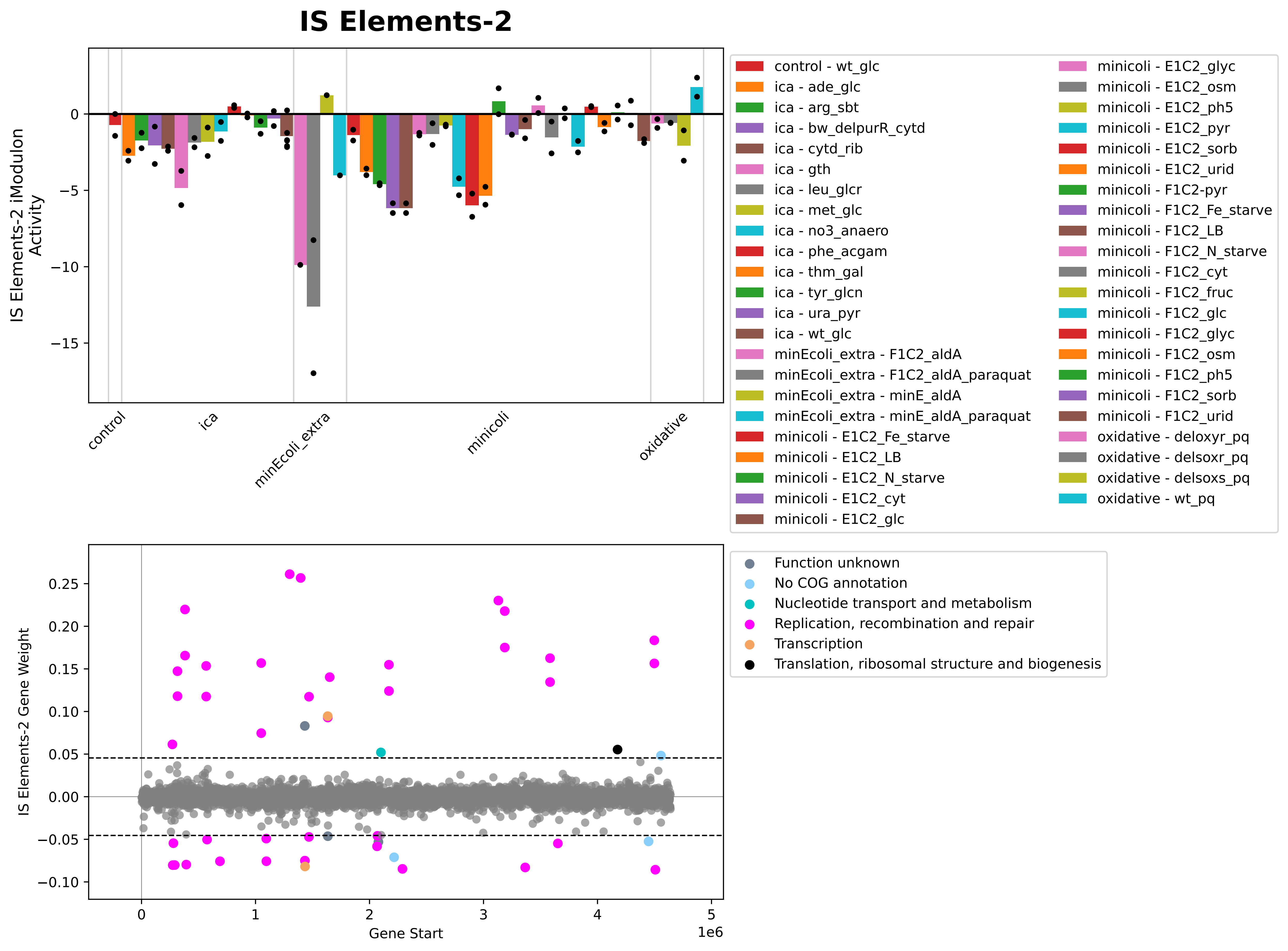
IS Elements-2
IS Elements-2 iModulon Activity
control
ica
minEcoli_extra
minicoli
oxidative
control - wt_glc
ica - ade_glc
ica - arg_sbt
ica - bw_delpurR_cytd
ica - cytd_rib
ica - gth
ica - leu_glcr
ica - met_glc
ica - no3_anaero
ica - phe_acgam
ica - thm_gal
ica - tyr_glcn
ica - ura_pyr
ica - wt_glc
minEcoli_extra - F1C2_aldA
minEcoli_extra - F1C2_aldA_paraquat
minEcoli_extra - minE_aldA
minEcoli_extra - minE_aldA_paraquat
minicoli - E1C2_Fe_starve
minicoli - E1C2_LB
minicoli - E1C2_N_starve
minicoli - E1C2_cyt
minicoli - E1C2_glc
minicoli - E1C2_glyc
minicoli - E1C2_osm
minicoli - E1C2_ph5
minicoli - E1C2_pyr
minicoli - E1C2_sorb
minicoli - E1C2_urid
minicoli - F1C2-pyr
minicoli - F1C2_Fe_starve
minicoli - F1C2_LB
minicoli - F1C2_N_starve
minicoli - F1C2_cyt
minicoli - F1C2_fruc
minicoli - F1C2_glc
minicoli - F1C2_glyc
minicoli - F1C2_osm
minicoli - F1C2_ph5
minicoli - F1C2_sorb
minicoli - F1C2_urid
oxidative - deloxyr_pq
oxidative - delsoxr_pq
oxidative - delsoxs_pq
oxidative - wt_pq
IS Elements-2 Gene Weight
Gene Start
1e6
Function unknown
No COG annotation
Nucleotide transport and metabolism
Replication, recombination and repair
Transcription
Translation, ribosomal structure and biogenesis

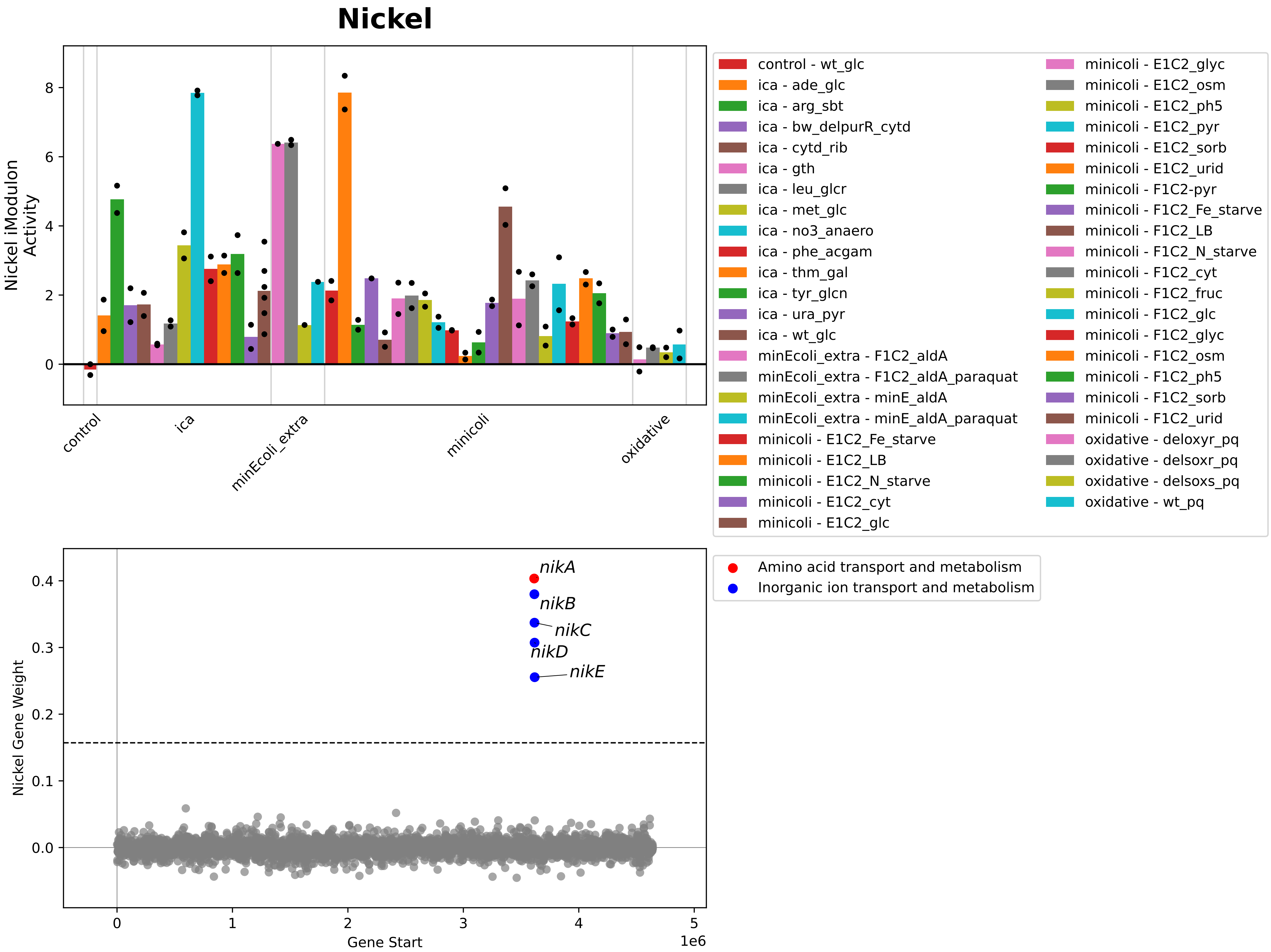
Nickel
Nickel iModulon Activity
control
ica
minEcoli_extra
minicoli
oxidative
control - wt_glc
ica - ade_glc
ica - arg_sbt
ica - bw_delpurR_cytd
ica - cytd_rib
ica - gth
ica - leu_glcr
ica - met_glc
ica - no3_anaero
ica - phe_acgam
ica - thm_gal
ica - tyr_glcn
ica - ura_pyr
ica - wt_glc
minEcoli_extra - F1C2_aldA
minEcoli_extra - F1C2_aldA_paraquat
minEcoli_extra - minE_aldA
minEcoli_extra - minE_aldA_paraquat
minicoli - E1C2_Fe_starve
minicoli - E1C2_LB
minicoli - E1C2_N_starve
minicoli - E1C2_cyt
minicoli - E1C2_glc
minicoli - E1C2_glyc
minicoli - E1C2_osm
minicoli - E1C2_ph5
minicoli - E1C2_pyr
minicoli - E1C2_sorb
minicoli - E1C2_urid
minicoli - F1C2-pyr
minicoli - F1C2_Fe_starve
minicoli - F1C2_LB
minicoli - F1C2_N_starve
minicoli - F1C2_cyt
minicoli - F1C2_fruc
minicoli - F1C2_glc
minicoli - F1C2_glyc
minicoli - F1C2_osm
minicoli - F1C2_ph5
minicoli - F1C2_sorb
minicoli - F1C2_urid
oxidative - deloxyr_pq
oxidative - delsoxr_pq
oxidative - delsoxs_pq
oxidative - wt_pq
nikA
nikB
nikC
nikD
nikE
Nickel Gene Weight
Gene Start
1e6
Amino acid transport and metabolism
Inorganic ion transport and metabolism

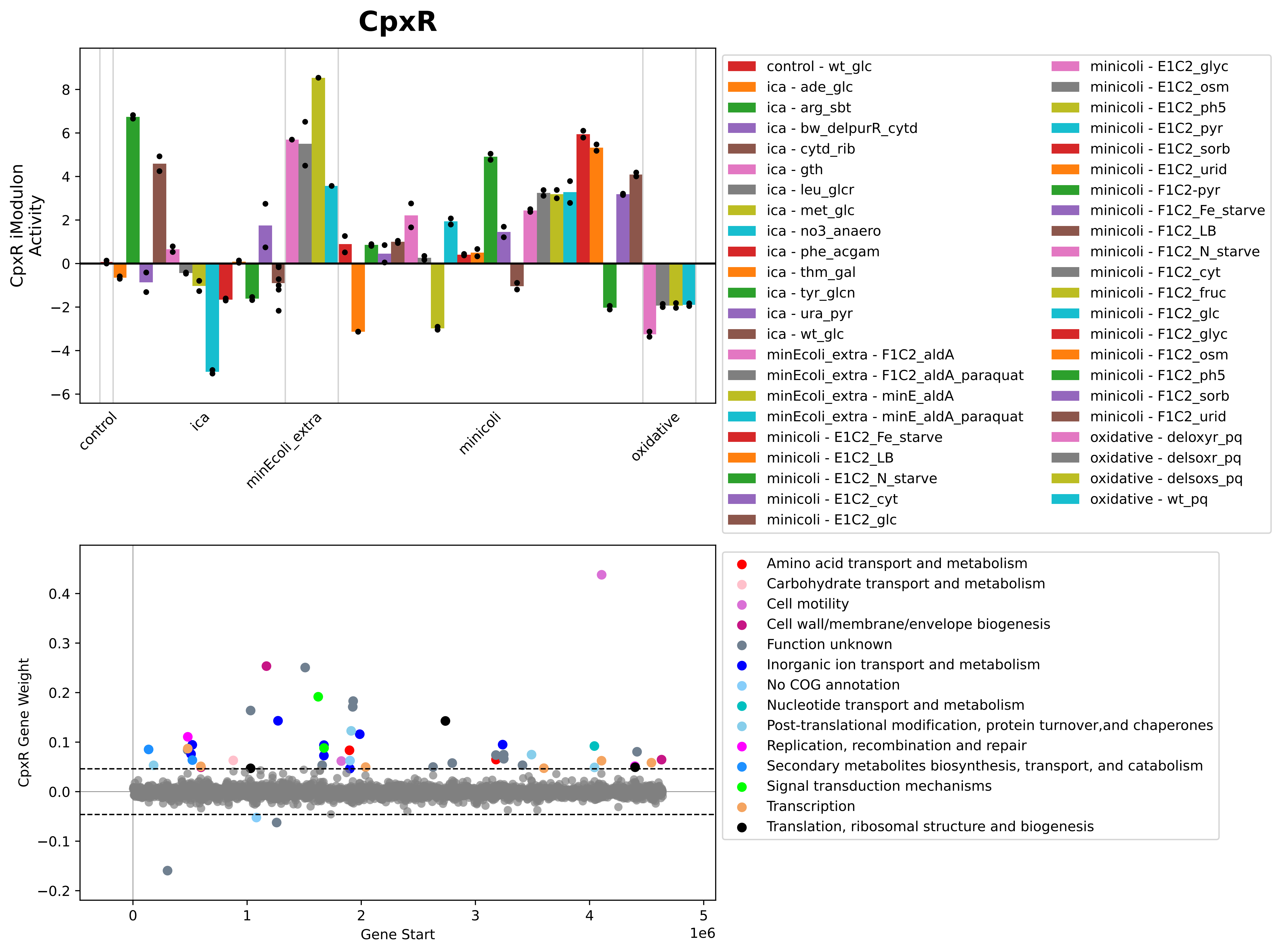
CpxR
CpxR iModulon Activity
control
ica
minEcoli_extra
minicoli
oxidative
control - wt_glc
ica - ade_glc
ica - arg_sbt
ica - bw_delpurR_cytd
ica - cytd_rib
ica - gth
ica - leu_glcr
ica - met_glc
ica - no3_anaero
ica - phe_acgam
ica - thm_gal
ica - tyr_glcn
ica - ura_pyr
ica - wt_glc
minEcoli_extra - F1C2_aldA
minEcoli_extra - F1C2_aldA_paraquat
minEcoli_extra - minE_aldA
minEcoli_extra - minE_aldA_paraquat
minicoli - E1C2_Fe_starve
minicoli - E1C2_LB
minicoli - E1C2_N_starve
minicoli - E1C2_cyt
minicoli - E1C2_glc
minicoli - E1C2_glyc
minicoli - E1C2_osm
minicoli - E1C2_ph5
minicoli - E1C2_pyr
minicoli - E1C2_sorb
minicoli - E1C2_urid
minicoli - F1C2-pyr
minicoli - F1C2_Fe_starve
minicoli - F1C2_LB
minicoli - F1C2_N_starve
minicoli - F1C2_cyt
minicoli - F1C2_fruc
minicoli - F1C2_glc
minicoli - F1C2_glyc
minicoli - F1C2_osm
minicoli - F1C2_ph5
minicoli - F1C2_sorb
minicoli - F1C2_urid
oxidative - deloxyr_pq
oxidative - delsoxr_pq
oxidative - delsoxs_pq
oxidative - wt_pq
CpxR Gene Weight
Gene Start
1e6
Amino acid transport and metabolism
Carbohydrate transport and metabolism
Cell motility
Cell wall/membrane/envelope biogenesis
Function unknown
Inorganic ion transport and metabolism
No COG annotation
Nucleotide transport and metabolism
Post-translational modification, protein turnover,and chaperones
Replication, recombination and repair
Secondary metabolites biosynthesis, transport, and catabolism
Signal transduction mechanisms
Transcription
Translation, ribosomal structure and biogenesis

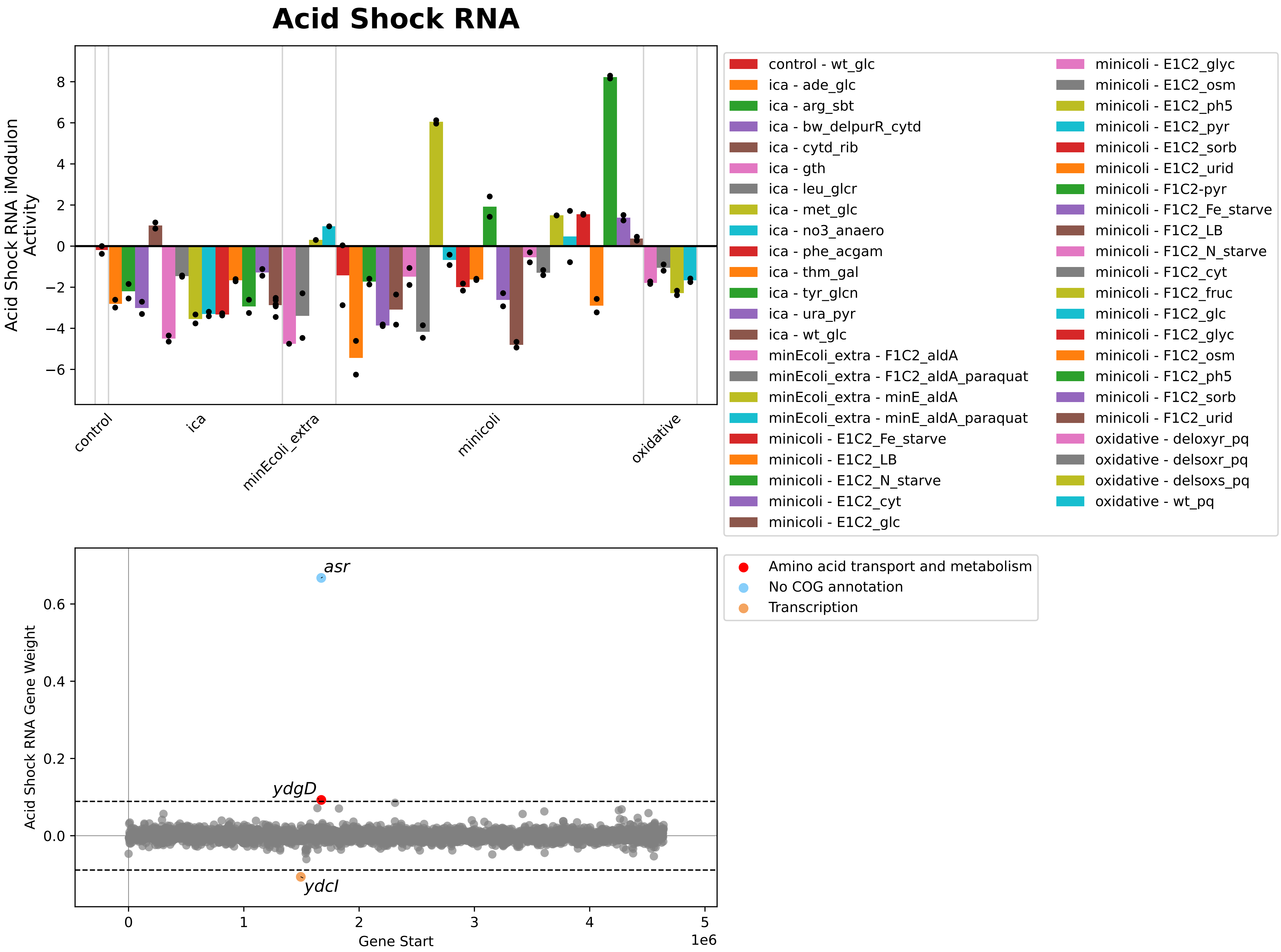

Acid Shock RNA
Acid Shock RNA iModulon Activity
control
ica
minEcoli_extra
minicoli
oxidative
control - wt_glc
ica - ade_glc
ica - arg_sbt
ica - bw_delpurR_cytd
ica - cytd_rib
ica - gth
ica - leu_glcr
ica - met_glc
ica - no3_anaero
ica - phe_acgam
ica - thm_gal
ica - tyr_glcn
ica - ura_pyr
ica - wt_glc
minEcoli_extra - F1C2_aldA
minEcoli_extra - F1C2_aldA_paraquat
minEcoli_extra - minE_aldA
minEcoli_extra - minE_aldA_paraquat
minicoli - E1C2_Fe_starve
minicoli - E1C2_LB
minicoli - E1C2_N_starve
minicoli - E1C2_cyt
minicoli - E1C2_glc
minicoli - E1C2_glyc
minicoli - E1C2_osm
minicoli - E1C2_ph5
minicoli - E1C2_pyr
minicoli - E1C2_sorb
minicoli - E1C2_urid
minicoli - F1C2-pyr
minicoli - F1C2_Fe_starve
minicoli - F1C2_LB
minicoli - F1C2_N_starve
minicoli - F1C2_cyt
minicoli - F1C2_fruc
minicoli - F1C2_glc
minicoli - F1C2_glyc
minicoli - F1C2_osm
minicoli - F1C2_ph5
minicoli - F1C2_sorb
minicoli - F1C2_urid
oxidative - deloxyr_pq
oxidative - delsoxr_pq
oxidative - delsoxs_pq
oxidative - wt_pq
asr
ydgD
ydcI
Acid Shock RNA Gene Weight
Gene Start
1e6
Amino acid transport and metabolism
No COG annotation
Transcription

FucR_ExuR

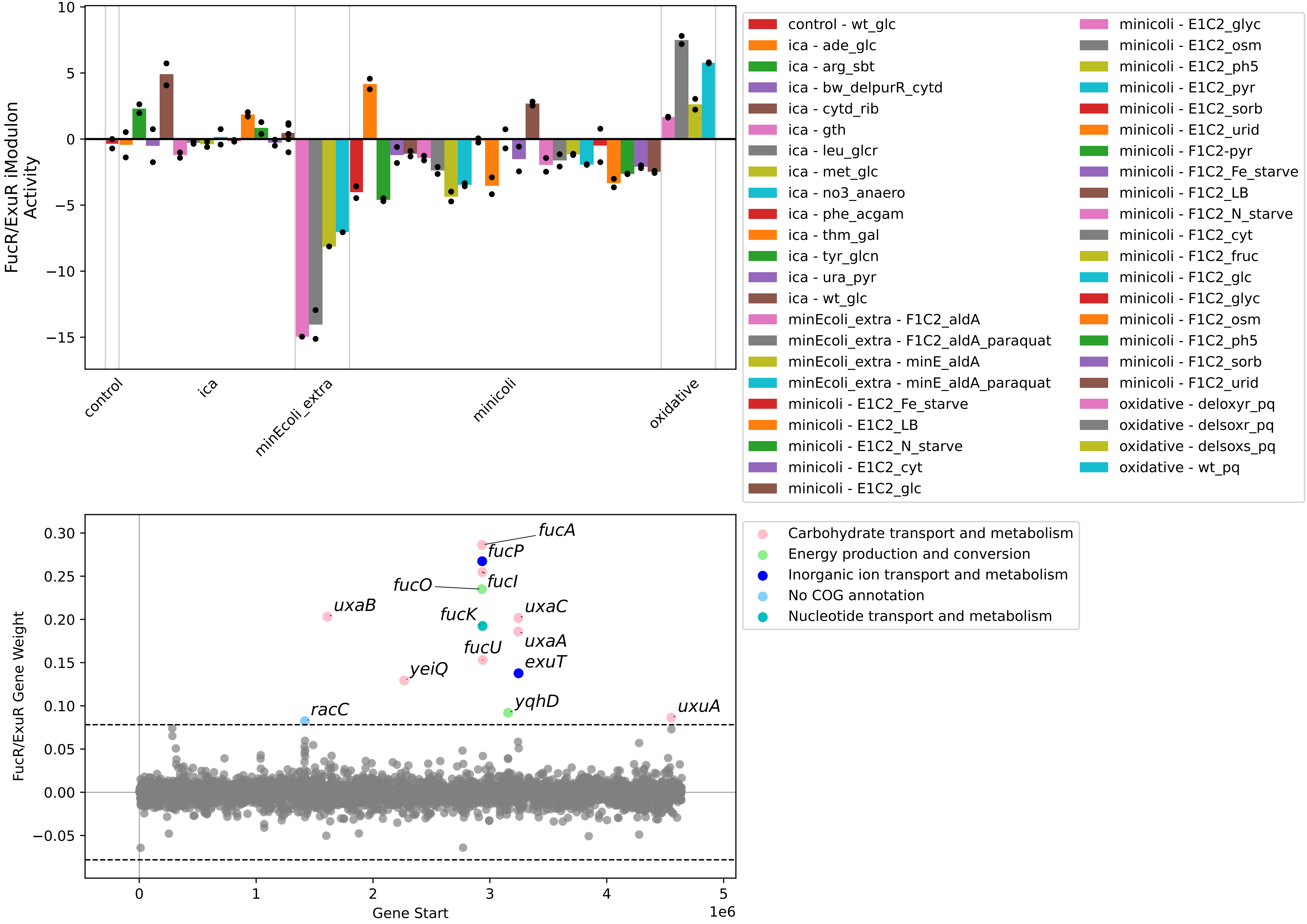
FucR/ExuR iModulon Activity
control
ica
minEcoli_extra
minicoli
oxidative
control - wt_glc
ica - ade_glc
ica - arg_sbt
ica - bw_delpurR_cytd
ica - cytd_rib
ica - gth
ica - leu_glcr
ica - met_glc
ica - no3_anaero
ica - phe_acgam
ica - thm_gal
ica - tyr_glcn
ica - ura_pyr
ica - wt_glc
minEcoli_extra - F1C2_aldA
minEcoli_extra - F1C2_aldA_paraquat
minEcoli_extra - minE_aldA
minEcoli_extra - minE_aldA_paraquat
minicoli - E1C2_Fe_starve
minicoli - E1C2_LB
minicoli - E1C2_N_starve
minicoli - E1C2_cyt
minicoli - E1C2_glc
minicoli - E1C2_glyc
minicoli - E1C2_osm
minicoli - E1C2_ph5
minicoli - E1C2_pyr
minicoli - E1C2_sorb
minicoli - E1C2_urid
minicoli - F1C2-pyr
minicoli - F1C2_Fe_starve
minicoli - F1C2_LB
minicoli - F1C2_N_starve
minicoli - F1C2_cyt
minicoli - F1C2_fruc
minicoli - F1C2_glc
minicoli - F1C2_glyc
minicoli - F1C2_osm
minicoli - F1C2_ph5
minicoli - F1C2_sorb
minicoli - F1C2_urid
oxidative - deloxyr_pq
oxidative - delsoxr_pq
oxidative - delsoxs_pq
oxidative - wt_pq
FucR/ExuR Gene Weight
Gene Start
1e6
fucA
fucP
fucI
fucO
uxaB
uxaC
fucK
uxaA
fucU
exuT
yeiQ
yqhD
racC
uxuA
Carbohydrate transport and metabolism
Energy production and conversion
Inorganic ion transport and metabolism
No COG annotation
Nucleotide transport and metabolism

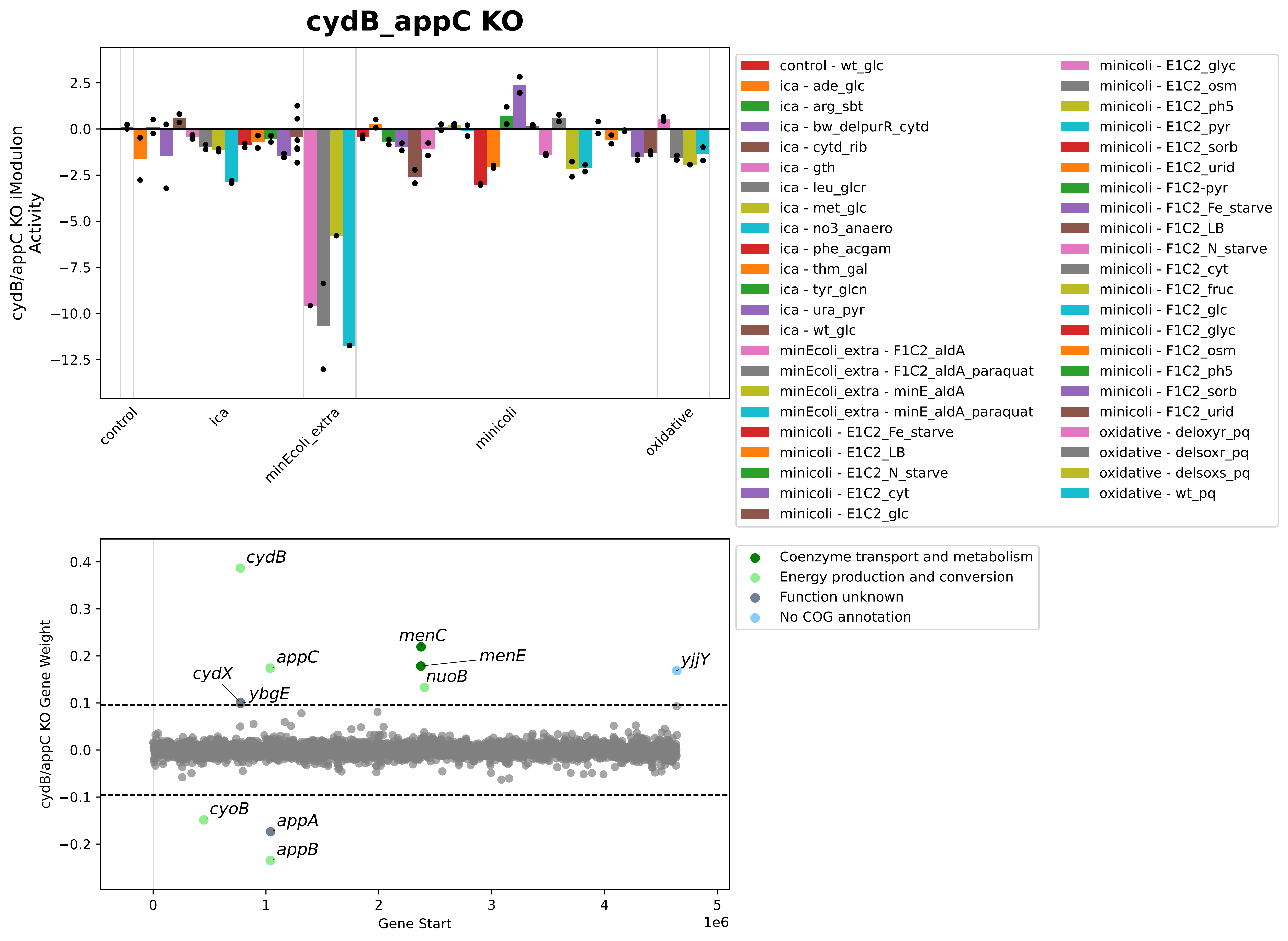
cydB_appC KO
cydB/appC KO iModulon Activity
2.5
0.0
−2.5
−5.0
−7.5
−10.0
−12.5
control
ica
minEcoli_extra
minicoli
oxidative
control - wt_glc
ica - ade_glc
ica - arg_sbt
ica - bw_delpurR_cytd
ica - cytd_rib
ica - gth
ica - leu_glcr
ica - met_glc
ica - no3_anaero
ica - phe_acgam
ica - thm_gal
ica - tyr_glcn
ica - ura_pyr
ica - wt_glc
minEcoli_extra - F1C2_aldA
minEcoli_extra - F1C2_aldA_paraquat
minEcoli_extra - minE_aldA
minEcoli_extra - minE_aldA_paraquat
minicoli - E1C2_Fe_starve
minicoli - E1C2_LB
minicoli - E1C2_N_starve
minicoli - E1C2_cyt
minicoli - E1C2_glc
minicoli - E1C2_glyc
minicoli - E1C2_osm
minicoli - E1C2_ph5
minicoli - E1C2_pyr
minicoli - E1C2_sorb
minicoli - E1C2_urid
minicoli - F1C2-pyr
minicoli - F1C2_Fe_starve
minicoli - F1C2_LB
minicoli - F1C2_N_starve
minicoli - F1C2_cyt
minicoli - F1C2_fruc
minicoli - F1C2_glc
minicoli - F1C2_glyc
minicoli - F1C2_osm
minicoli - F1C2_ph5
minicoli - F1C2_sorb
minicoli - F1C2_urid
oxidative - deloxyr_pq
oxidative - delsoxr_pq
oxidative - delsoxs_pq
oxidative - wt_pq
cydB/appC KO Gene Weight
0.4
0.3
0.2
0.1
0.0
−0.1
−0.2
cydB
menC
appC
menE
cydX
nuoB
ybgE
yjjY
cyoB
appA
appB
0
1
2
3
4
5
Gene Start
1e6
Coenzyme transport and metabolism
Energy production and conversion
Function unknown
No COG annotation

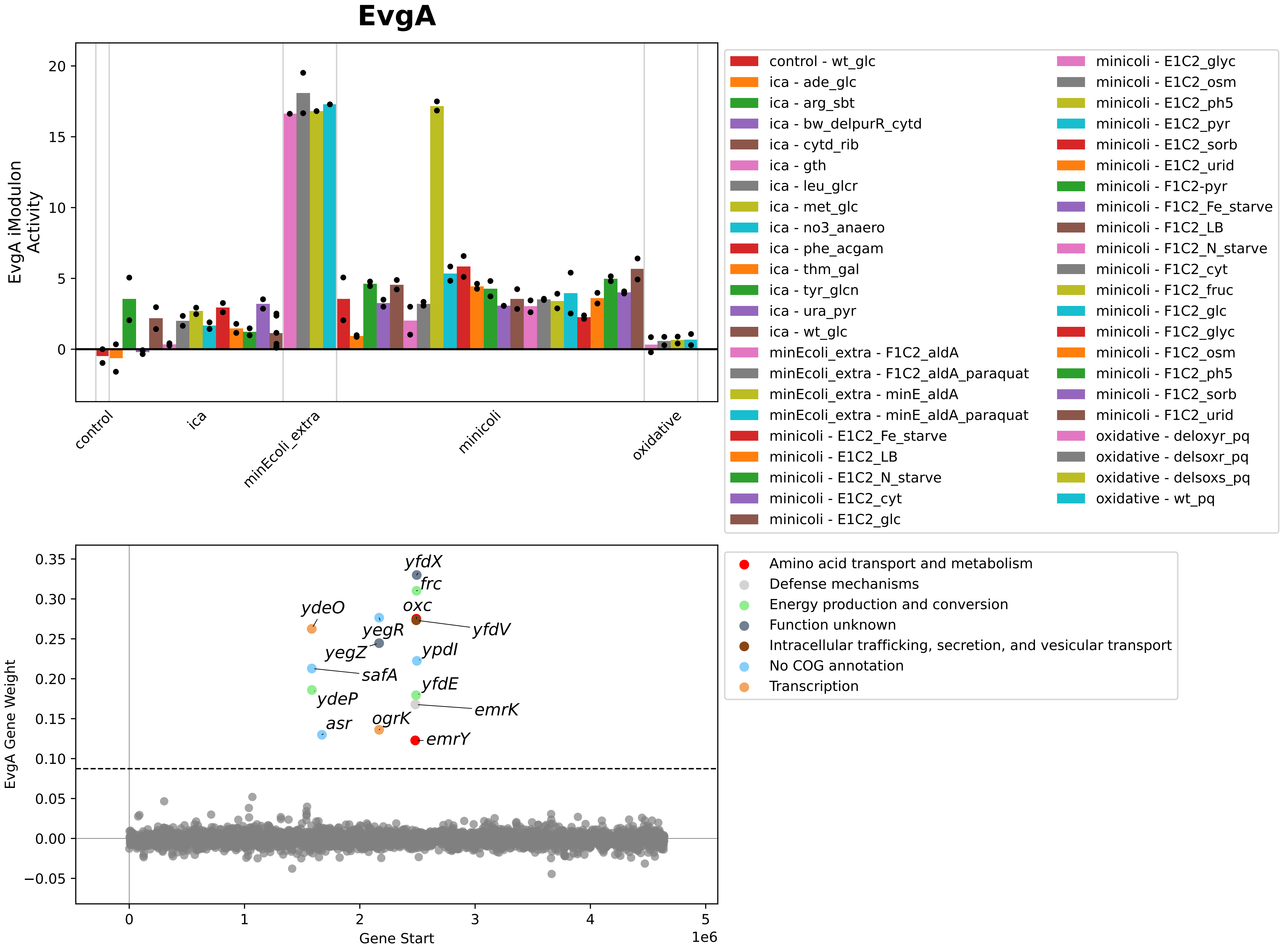

EvgA
EvgA iModulon Activity
control
ica
minEcoli_extra
minicoli
oxidative
control - wt_glc
ica - ade_glc
ica - arg_sbt
ica - bw_delpurR_cytd
ica - cytd_rib
ica - gth
ica - leu_glcr
ica - met_glc
ica - no3_anaero
ica - phe_acgam
ica - thm_gal
ica - tyr_glcn
ica - ura_pyr
ica - wt_glc
minEcoli_extra - F1C2_aldA
minEcoli_extra - F1C2_aldA_paraquat
minEcoli_extra - minE_aldA
minEcoli_extra - minE_aldA_paraquat
minicoli - E1C2_Fe_starve
minicoli - E1C2_LB
minicoli - E1C2_N_starve
minicoli - E1C2_cyt
minicoli - E1C2_glc
minicoli - E1C2_glyc
minicoli - E1C2_osm
minicoli - E1C2_ph5
minicoli - E1C2_pyr
minicoli - E1C2_sorb
minicoli - E1C2_urid
minicoli - F1C2-pyr
minicoli - F1C2_Fe_starve
minicoli - F1C2_LB
minicoli - F1C2_N_starve
minicoli - F1C2_cyt
minicoli - F1C2_fruc
minicoli - F1C2_glc
minicoli - F1C2_glyc
minicoli - F1C2_osm
minicoli - F1C2_ph5
minicoli - F1C2_sorb
minicoli - F1C2_urid
oxidative - deloxyr_pq
oxidative - delsoxr_pq
oxidative - delsoxs_pq
oxidative - wt_pq
EvgA Gene Weight
Gene Start
1e6
yfdX
frc
oxc
ydeO
yegR
yfdV
yegZ
ypdI
safA
yfdE
ydeP
emrK
asr
ogrK
emrY
Amino acid transport and metabolism
Defense mechanisms
Energy production and conversion
Function unknown
Intracellular trafficking, secretion, and vesicular transport
No COG annotation
Transcription

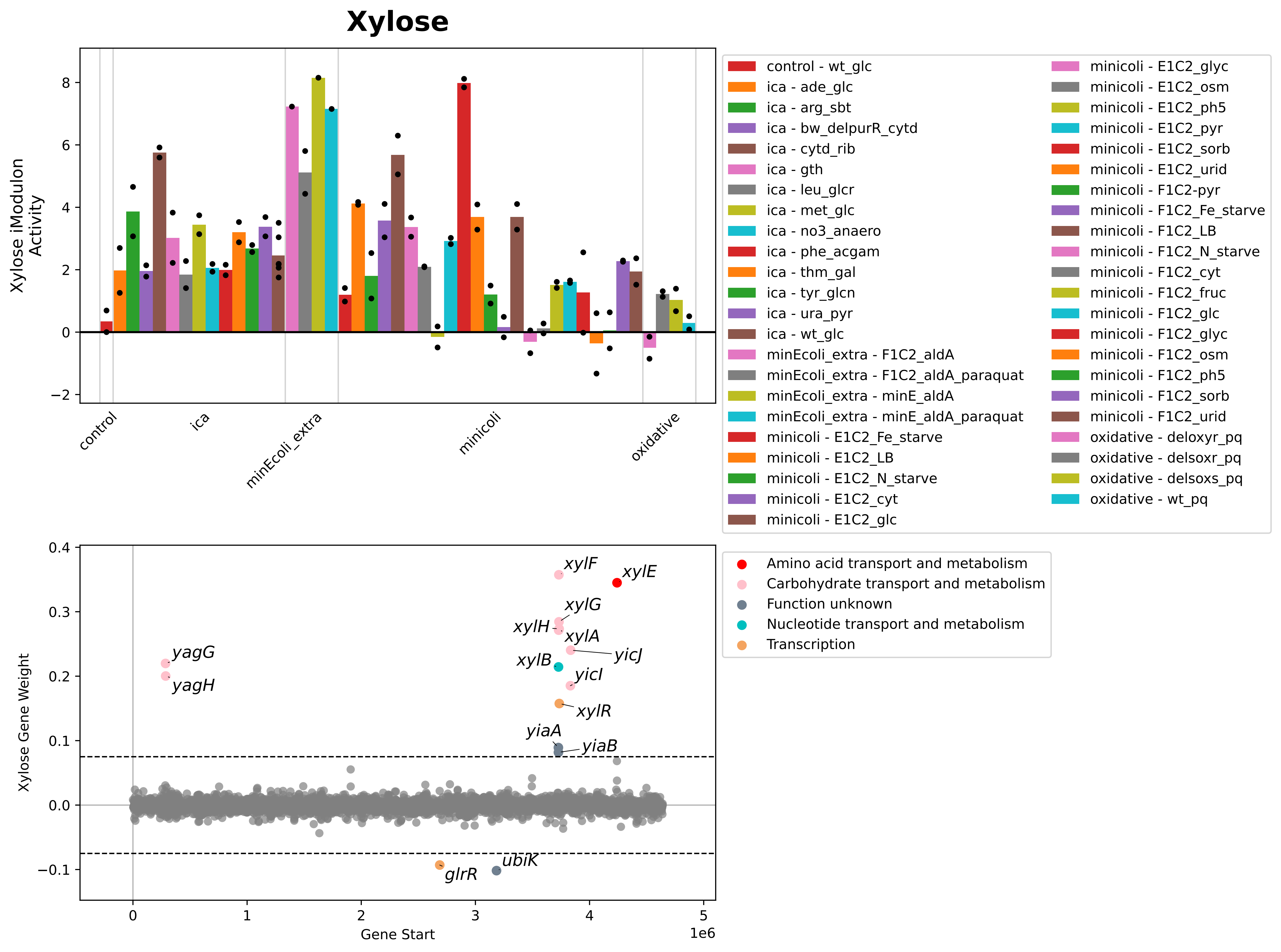

Xylose
Xylose iModulon Activity
control
ica
minEcoli_extra
minicoli
oxidative
control - wt_glc
ica - ade_glc
ica - arg_sbt
ica - bw_delpurR_cytd
ica - cytd_rib
ica - gth
ica - leu_glcr
ica - met_glc
ica - no3_anaero
ica - phe_acgam
ica - thm_gal
ica - tyr_glcn
ica - ura_pyr
ica - wt_glc
minEcoli_extra - F1C2_aldA
minEcoli_extra - F1C2_aldA_paraquat
minEcoli_extra - minE_aldA
minEcoli_extra - minE_aldA_paraquat
minicoli - E1C2_Fe_starve
minicoli - E1C2_LB
minicoli - E1C2_N_starve
minicoli - E1C2_cyt
minicoli - E1C2_glc
minicoli - E1C2_glyc
minicoli - E1C2_osm
minicoli - E1C2_ph5
minicoli - E1C2_pyr
minicoli - E1C2_sorb
minicoli - E1C2_urid
minicoli - F1C2-pyr
minicoli - F1C2_Fe_starve
minicoli - F1C2_LB
minicoli - F1C2_N_starve
minicoli - F1C2_cyt
minicoli - F1C2_fruc
minicoli - F1C2_glc
minicoli - F1C2_glyc
minicoli - F1C2_osm
minicoli - F1C2_ph5
minicoli - F1C2_sorb
minicoli - F1C2_urid
oxidative - deloxyr_pq
oxidative - delsoxr_pq
oxidative - delsoxs_pq
oxidative - wt_pq
Xylose Gene Weight
Gene Start
1e6
xylF
xylE
xylG
xylH
xylA
yicJ
xylB
yicI
xylR
yiaA
yiaB
yagG
yagH
glrR
ubiK
Amino acid transport and metabolism
Carbohydrate transport and metabolism
Function unknown
Nucleotide transport and metabolism
Transcription

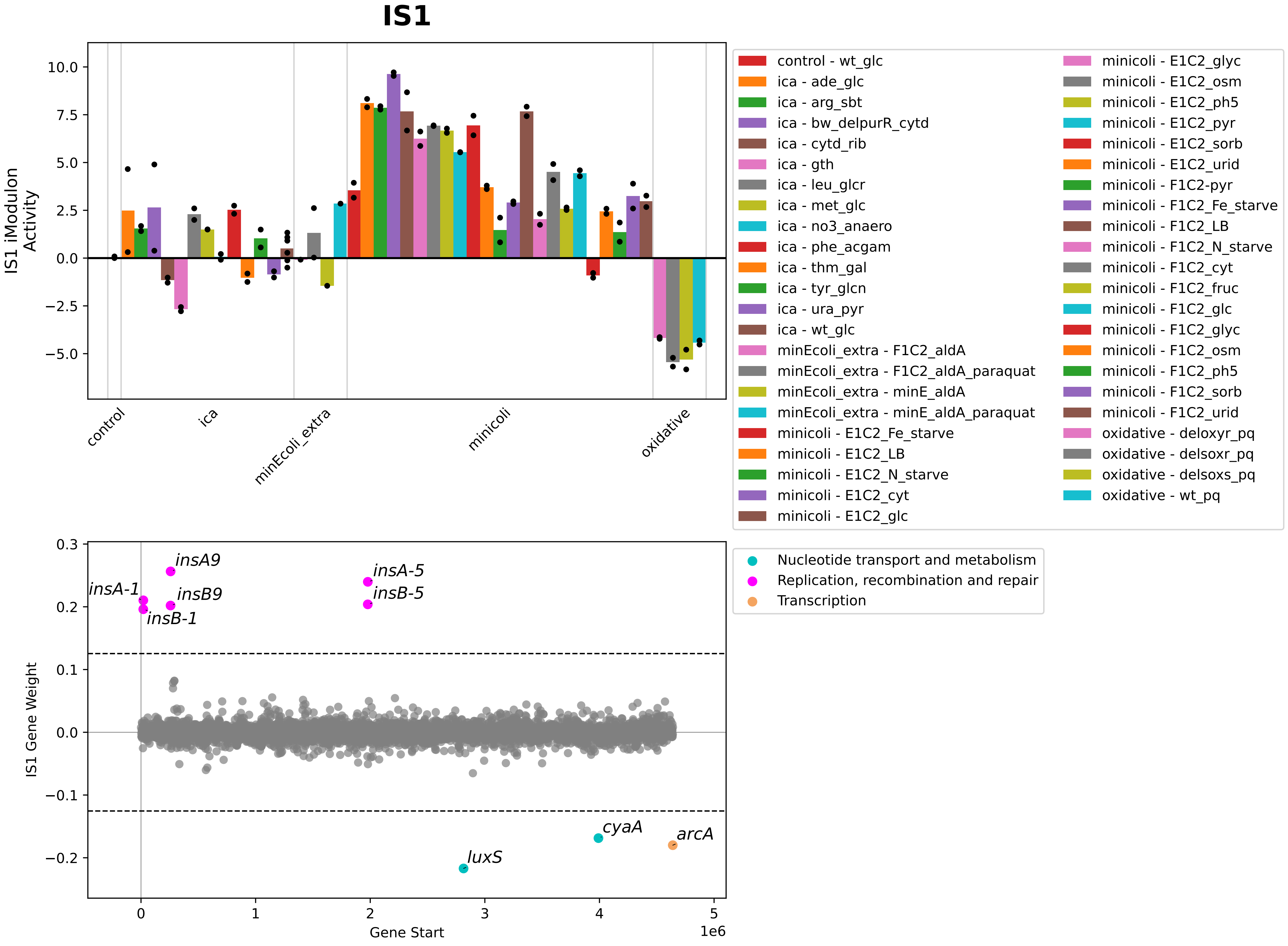
IS1
IS1 iModulon Activity
control
ica
minEcoli_extra
minicoli
oxidative
control - wt_glc
ica - ade_glc
ica - arg_sbt
ica - bw_delpurR_cytd
ica - cytd_rib
ica - gth
ica - leu_glcr
ica - met_glc
ica - no3_anaero
ica - phe_acgam
ica - thm_gal
ica - tyr_glcn
ica - ura_pyr
ica - wt_glc
minEcoli_extra - F1C2_aldA
minEcoli_extra - F1C2_aldA_paraquat
minEcoli_extra - minE_aldA
minEcoli_extra - minE_aldA_paraquat
minicoli - E1C2_Fe_starve
minicoli - E1C2_LB
minicoli - E1C2_N_starve
minicoli - E1C2_cyt
minicoli - E1C2_glc
minicoli - E1C2_glyc
minicoli - E1C2_osm
minicoli - E1C2_ph5
minicoli - E1C2_pyr
minicoli - E1C2_sorb
minicoli - E1C2_urid
minicoli - F1C2-pyr
minicoli - F1C2_Fe_starve
minicoli - F1C2_LB
minicoli - F1C2_N_starve
minicoli - F1C2_cyt
minicoli - F1C2_fruc
minicoli - F1C2_glc
minicoli - F1C2_glyc
minicoli - F1C2_osm
minicoli - F1C2_ph5
minicoli - F1C2_sorb
minicoli - F1C2_urid
oxidative - deloxyr_pq
oxidative - delsoxr_pq
oxidative - delsoxs_pq
oxidative - wt_pq
IS1 Gene Weight
Gene Start
1e6
insA9
insA-1
insB9
insB-1
insA-5
insB-5
cyaA
arcA
luxS
Nucleotide transport and metabolism
Replication, recombination and repair
Transcription

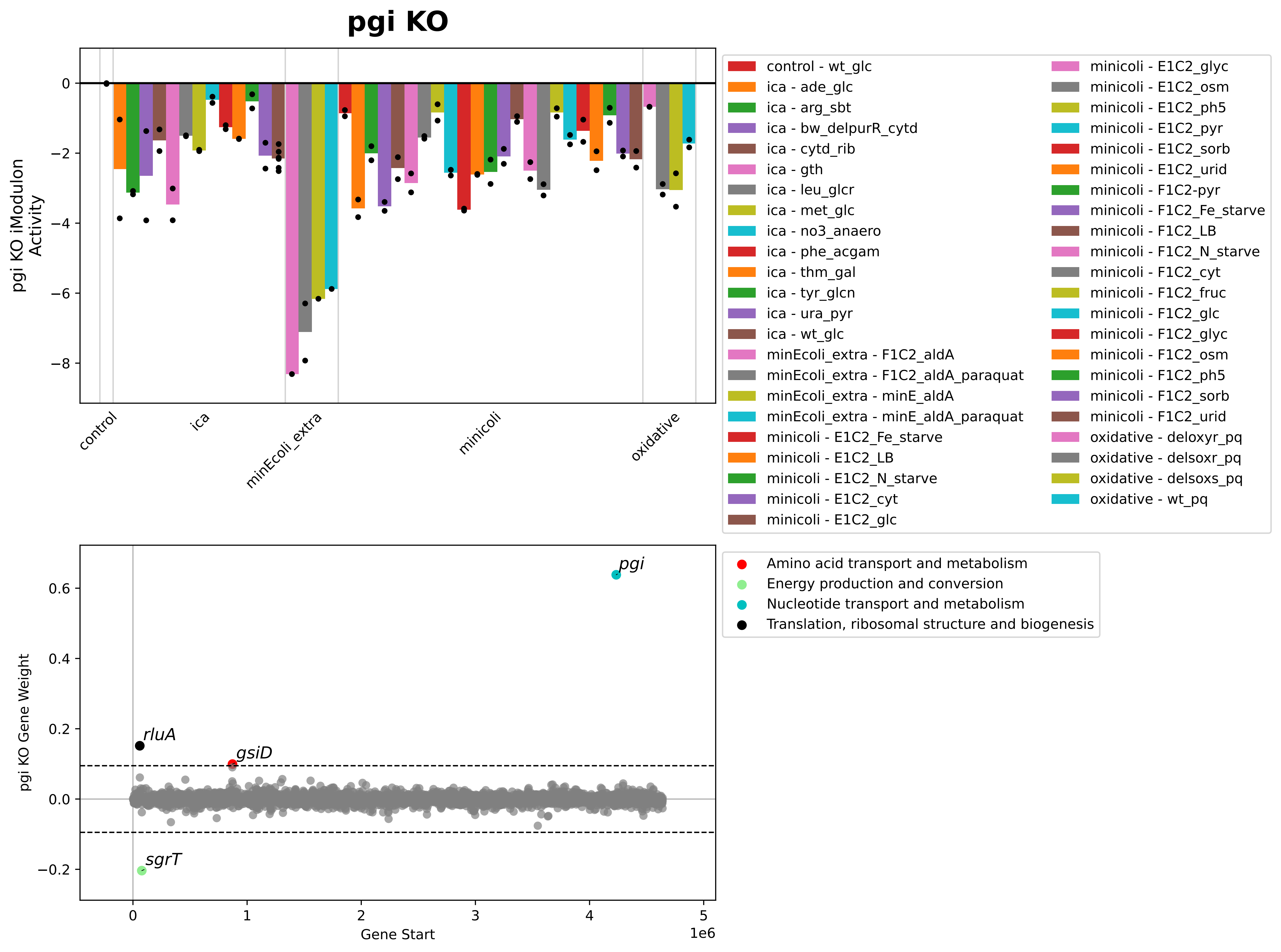

pgi KO
pgi KO iModulon Activity
0
−2
−4
−6
−8
control
ica
minEcoli_extra
minicoli
oxidative
control - wt_glc
ica - ade_glc
ica - arg_sbt
ica - bw_delpurR_cytd
ica - cytd_rib
ica - gth
ica - leu_glcr
ica - met_glc
ica - no3_anaero
ica - phe_acgam
ica - thm_gal
ica - tyr_glcn
ica - ura_pyr
ica - wt_glc
minEcoli_extra - F1C2_aldA
minEcoli_extra - F1C2_aldA_paraquat
minEcoli_extra - minE_aldA
minEcoli_extra - minE_aldA_paraquat
minicoli - E1C2_Fe_starve
minicoli - E1C2_LB
minicoli - E1C2_N_starve
minicoli - E1C2_cyt
minicoli - E1C2_glc
minicoli - E1C2_glyc
minicoli - E1C2_osm
minicoli - E1C2_ph5
minicoli - E1C2_pyr
minicoli - E1C2_sorb
minicoli - E1C2_urid
minicoli - F1C2-pyr
minicoli - F1C2_Fe_starve
minicoli - F1C2_LB
minicoli - F1C2_N_starve
minicoli - F1C2_cyt
minicoli - F1C2_fruc
minicoli - F1C2_glc
minicoli - F1C2_glyc
minicoli - F1C2_osm
minicoli - F1C2_ph5
minicoli - F1C2_sorb
minicoli - F1C2_urid
oxidative - deloxyr_pq
oxidative - delsoxr_pq
oxidative - delsoxs_pq
oxidative - wt_pq
pgi KO Gene Weight
0.6
0.4
0.2
0.0
−0.2
pgi
rluA
gsiD
sgrT
0
1
2
3
4
5
Gene Start
1e6
Amino acid transport and metabolism
Energy production and conversion
Nucleotide transport and metabolism
Translation, ribosomal structure and biogenesis

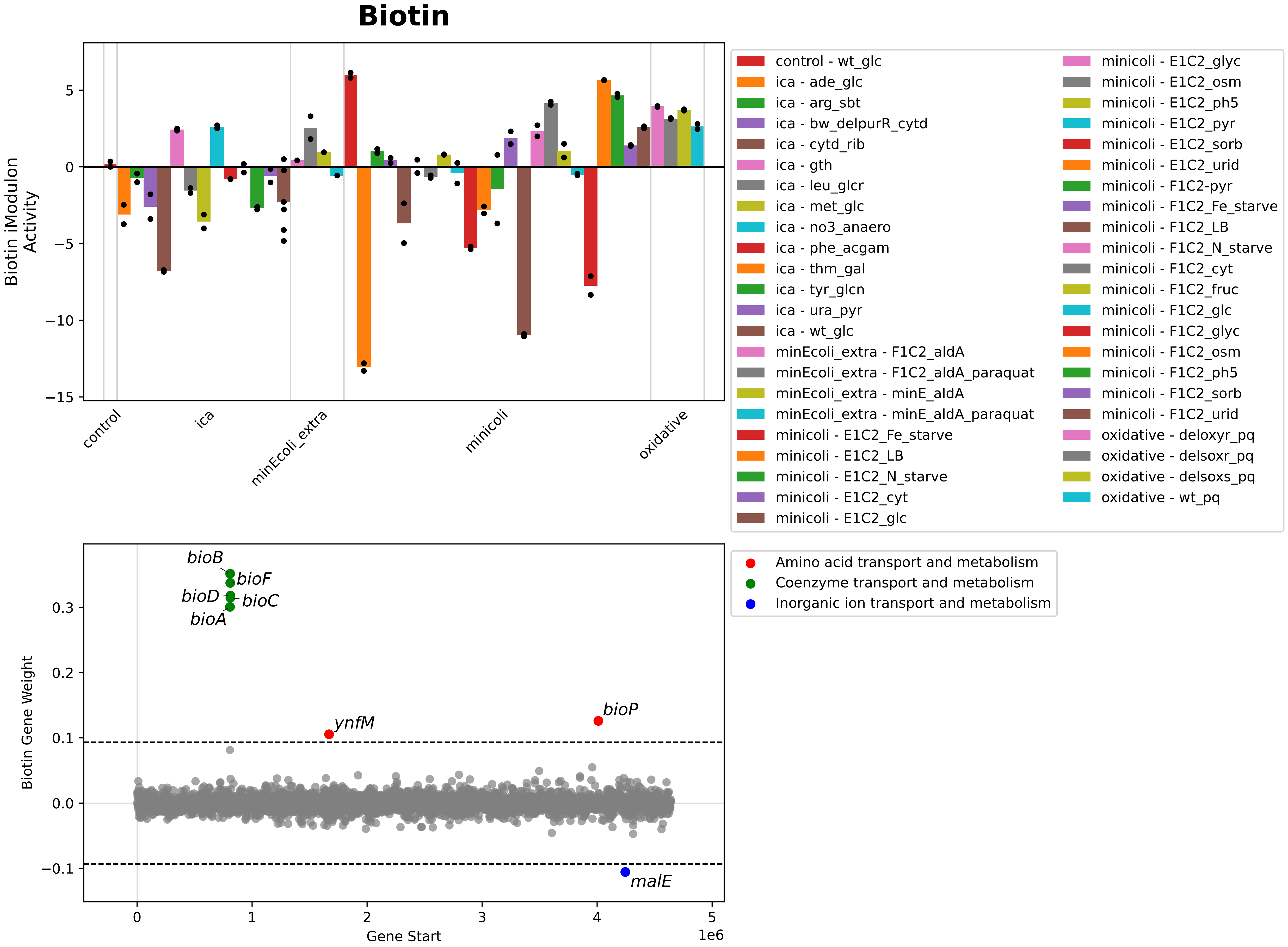
Biotin
Biotin iModulon Activity
control
ica
minEcoli_extra
minicoli
oxidative
control - wt_glc
ica - ade_glc
ica - arg_sbt
ica - bw_delpurR_cytd
ica - cytd_rib
ica - gth
ica - leu_glcr
ica - met_glc
ica - no3_anaero
ica - phe_acgam
ica - thm_gal
ica - tyr_glcn
ica - ura_pyr
ica - wt_glc
minEcoli_extra - F1C2_aldA
minEcoli_extra - F1C2_aldA_paraquat
minEcoli_extra - minE_aldA
minEcoli_extra - minE_aldA_paraquat
minicoli - E1C2_Fe_starve
minicoli - E1C2_LB
minicoli - E1C2_N_starve
minicoli - E1C2_cyt
minicoli - E1C2_glc
minicoli - E1C2_glyc
minicoli - E1C2_osm
minicoli - E1C2_ph5
minicoli - E1C2_pyr
minicoli - E1C2_sorb
minicoli - E1C2_urid
minicoli - F1C2-pyr
minicoli - F1C2_Fe_starve
minicoli - F1C2_LB
minicoli - F1C2_N_starve
minicoli - F1C2_cyt
minicoli - F1C2_fruc
minicoli - F1C2_glc
minicoli - F1C2_glyc
minicoli - F1C2_osm
minicoli - F1C2_ph5
minicoli - F1C2_sorb
minicoli - F1C2_urid
oxidative - deloxyr_pq
oxidative - delsoxr_pq
oxidative - delsoxs_pq
oxidative - wt_pq
Biotin Gene Weight
Gene Start
1e6
bioB
bioF
bioD
bioC
bioA
ynfM
bioP
malE
Amino acid transport and metabolism
Coenzyme transport and metabolism
Inorganic ion transport and metabolism

N Reduction

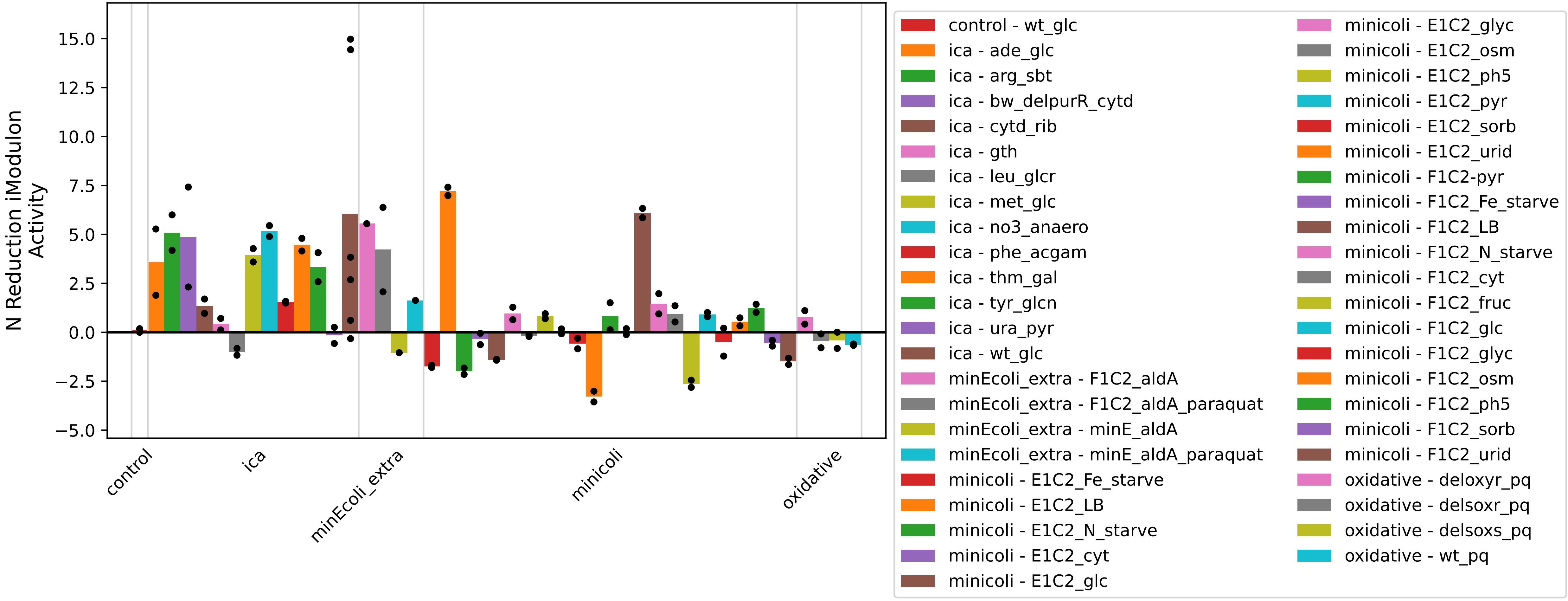

N Reduction iModulon Activity
15.0
12.5
10.0
7.5
5.0
2.5
0.0
−2.5
−5.0
control
ica
minEcoli_extra
minicoli
oxidative
control - wt_glc
ica - ade_glc
ica - arg_sbt
ica - bw_delpurR_cytd
ica - cytd_rib
ica - gth
ica - leu_glcr
ica - met_glc
ica - no3_anaero
ica - phe_acgam
ica - thm_gal
ica - tyr_glcn
ica - ura_pyr
ica - wt_glc
minEcoli_extra - F1C2_aldA
minEcoli_extra - F1C2_aldA_paraquat
minEcoli_extra - minE_aldA
minEcoli_extra - minE_aldA_paraquat
minicoli - E1C2_Fe_starve
minicoli - E1C2_LB
minicoli - E1C2_N_starve
minicoli - E1C2_cyt
minicoli - E1C2_glc
minicoli - E1C2_glyc
minicoli - E1C2_osm
minicoli - E1C2_ph5
minicoli - E1C2_pyr
minicoli - E1C2_sorb
minicoli - E1C2_urid
minicoli - F1C2-pyr
minicoli - F1C2_Fe_starve
minicoli - F1C2_LB
minicoli - F1C2_N_starve
minicoli - F1C2_cyt
minicoli - F1C2_fruc
minicoli - F1C2_glc
minicoli - F1C2_glyc
minicoli - F1C2_osm
minicoli - F1C2_ph5
minicoli - F1C2_sorb
minicoli - F1C2_urid
oxidative - deloxyr_pq
oxidative - delsoxr_pq
oxidative - delsoxs_pq
oxidative - wt_pq

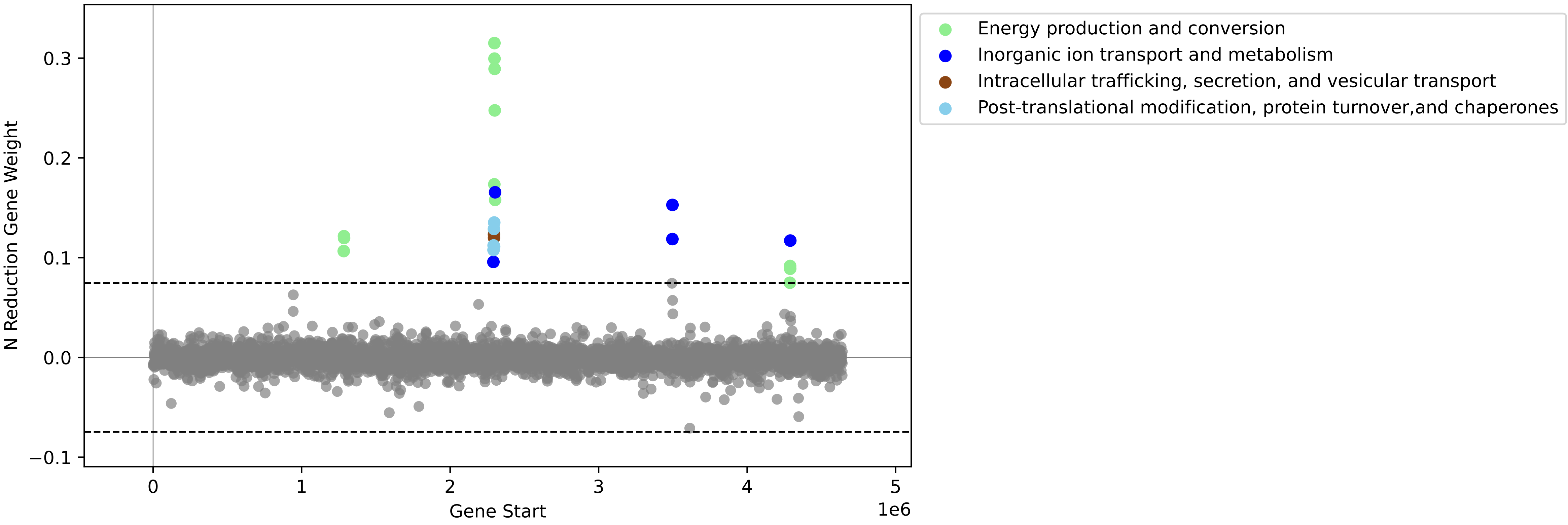

N Reduction Gene Weight
0.3
0.2
0.1
0.0
−0.1
0
1
2
3
4
5
1e6
Gene Start
Energy production and conversion
Inorganic ion transport and metabolism
Intracellular trafficking, secretion, and vesicular transport
Post-translational modification, protein turnover,and chaperones

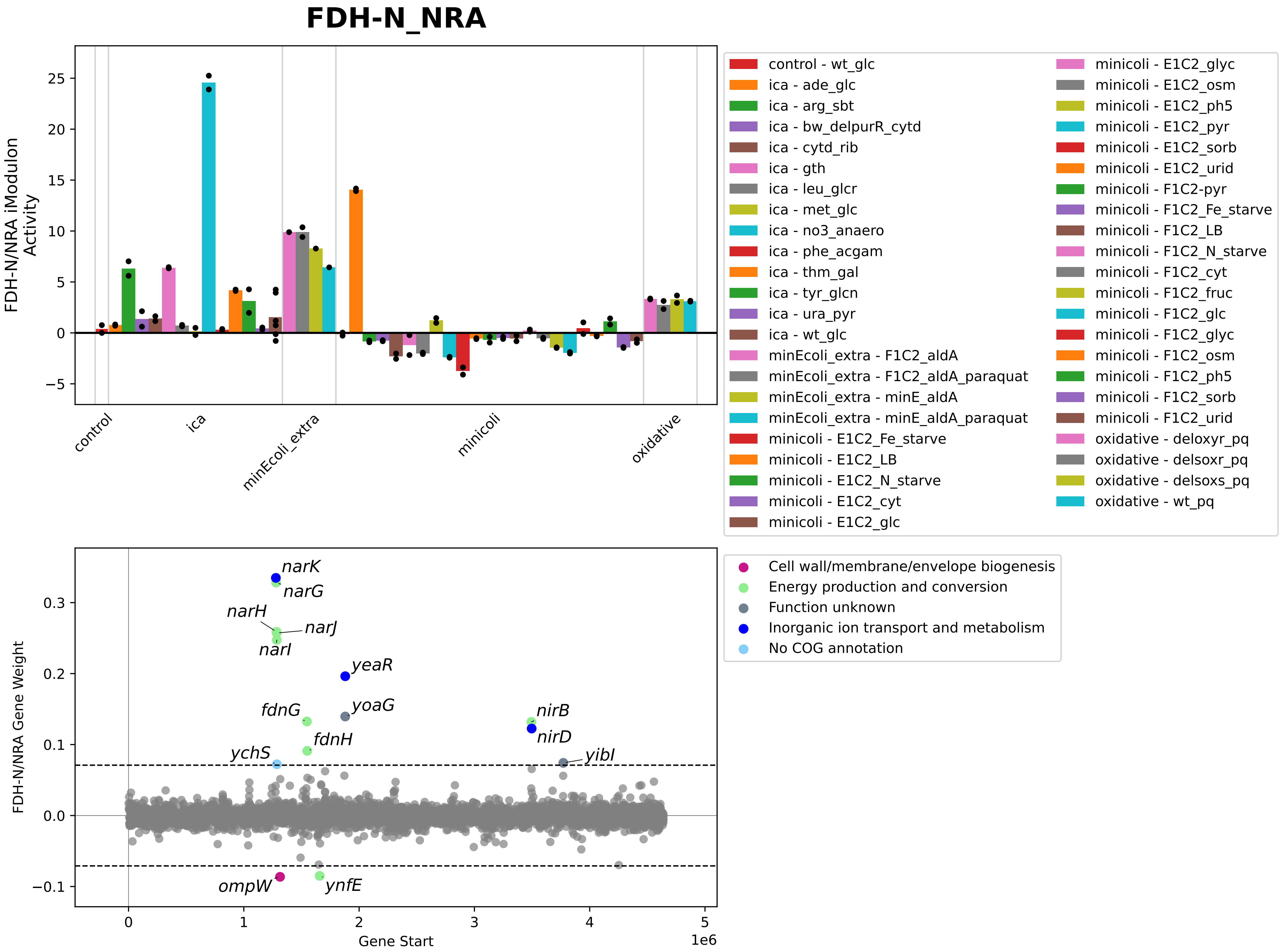
FDH-N_NRA
FDH-N/NRA iModulon Activity
control
ica
minEcoli_extra
minicoli
oxidative
control - wt_glc
ica - ade_glc
ica - arg_sbt
ica - bw_delpurR_cytd
ica - cytd_rib
ica - gth
ica - leu_glcr
ica - met_glc
ica - no3_anaero
ica - phe_acgam
ica - thm_gal
ica - tyr_glcn
ica - ura_pyr
ica - wt_glc
minEcoli_extra - F1C2_aldA
minEcoli_extra - F1C2_aldA_paraquat
minEcoli_extra - minE_aldA
minEcoli_extra - minE_aldA_paraquat
minicoli - E1C2_Fe_starve
minicoli - E1C2_LB
minicoli - E1C2_N_starve
minicoli - E1C2_cyt
minicoli - E1C2_glc
minicoli - E1C2_glyc
minicoli - E1C2_osm
minicoli - E1C2_ph5
minicoli - E1C2_pyr
minicoli - E1C2_sorb
minicoli - E1C2_urid
minicoli - F1C2-pyr
minicoli - F1C2_Fe_starve
minicoli - F1C2_LB
minicoli - F1C2_N_starve
minicoli - F1C2_cyt
minicoli - F1C2_fruc
minicoli - F1C2_glc
minicoli - F1C2_glyc
minicoli - F1C2_osm
minicoli - F1C2_ph5
minicoli - F1C2_sorb
minicoli - F1C2_urid
oxidative - deloxyr_pq
oxidative - delsoxr_pq
oxidative - delsoxs_pq
oxidative - wt_pq
FDH-N/NRA Gene Weight
Gene Start
1e6
narK
narG
narH
narJ
narI
yeaR
fdnG
yoaG
fdnH
ychS
nirB
nirD
yibI
ompW
ynfE
Cell wall/membrane/envelope biogenesis
Energy production and conversion
Function unknown
Inorganic ion transport and metabolism
No COG annotation

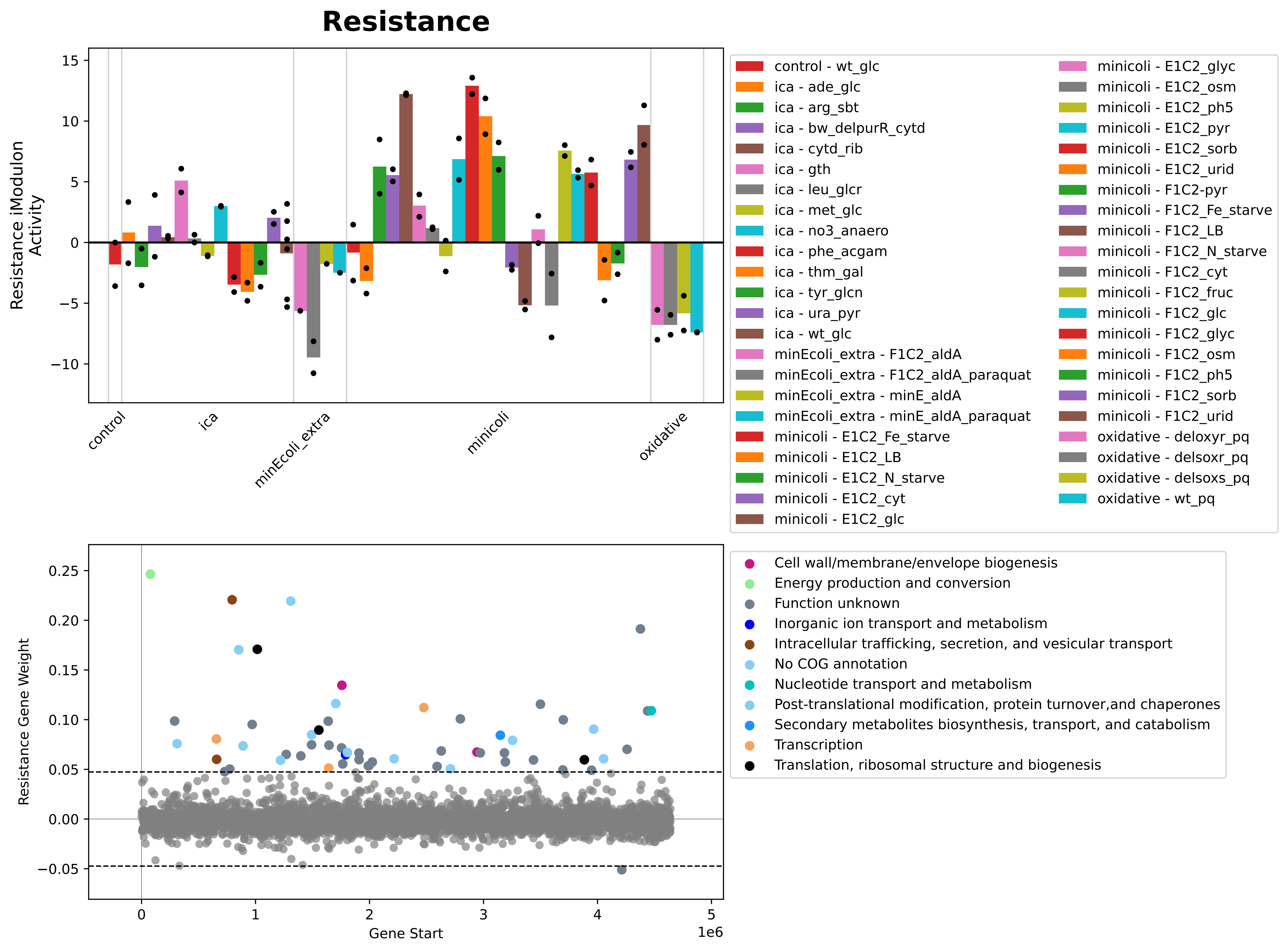
Resistance
Resistance iModulon Activity
control
ica
minEcoli_extra
minicoli
oxidative
control - wt_glc
ica - ade_glc
ica - arg_sbt
ica - bw_delpurR_cytd
ica - cytd_rib
ica - gth
ica - leu_glcr
ica - met_glc
ica - no3_anaero
ica - phe_acgam
ica - thm_gal
ica - tyr_glcn
ica - ura_pyr
ica - wt_glc
minEcoli_extra - F1C2_aldA
minEcoli_extra - F1C2_aldA_paraquat
minEcoli_extra - minE_aldA
minEcoli_extra - minE_aldA_paraquat
minicoli - E1C2_Fe_starve
minicoli - E1C2_LB
minicoli - E1C2_N_starve
minicoli - E1C2_cyt
minicoli - E1C2_glc
minicoli - E1C2_glyc
minicoli - E1C2_osm
minicoli - E1C2_ph5
minicoli - E1C2_pyr
minicoli - E1C2_sorb
minicoli - E1C2_urid
minicoli - F1C2-pyr
minicoli - F1C2_Fe_starve
minicoli - F1C2_LB
minicoli - F1C2_N_starve
minicoli - F1C2_cyt
minicoli - F1C2_fruc
minicoli - F1C2_glc
minicoli - F1C2_glyc
minicoli - F1C2_osm
minicoli - F1C2_ph5
minicoli - F1C2_sorb
minicoli - F1C2_urid
oxidative - deloxyr_pq
oxidative - delsoxr_pq
oxidative - delsoxs_pq
oxidative - wt_pq
Resistance Gene Weight
Gene Start
1e6
Cell wall/membrane/envelope biogenesis
Energy production and conversion
Function unknown
Inorganic ion transport and metabolism
Intracellular trafficking, secretion, and vesicular transport
No COG annotation
Nucleotide transport and metabolism
Post-translational modification, protein turnover,and chaperones
Secondary metabolites biosynthesis, transport, and catabolism
Transcription
Translation, ribosomal structure and biogenesis

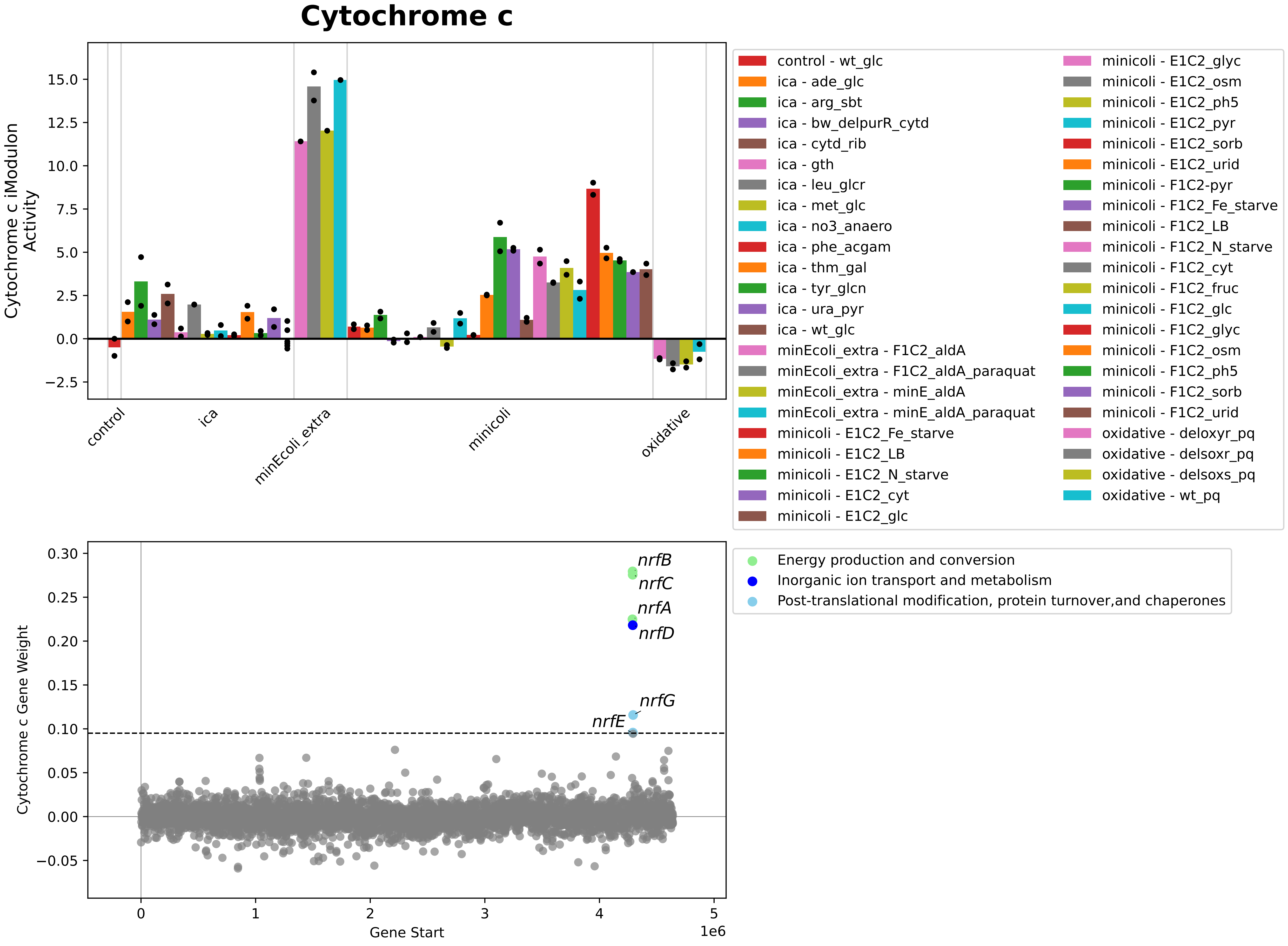

Cytochrome c
Cytochrome c iModulon Activity
control
ica
minEcoli_extra
minicoli
oxidative
control - wt_glc
ica - ade_glc
ica - arg_sbt
ica - bw_delpurR_cytd
ica - cytd_rib
ica - gth
ica - leu_glcr
ica - met_glc
ica - no3_anaero
ica - phe_acgam
ica - thm_gal
ica - tyr_glcn
ica - ura_pyr
ica - wt_glc
minEcoli_extra - F1C2_aldA
minEcoli_extra - F1C2_aldA_paraquat
minEcoli_extra - minE_aldA
minEcoli_extra - minE_aldA_paraquat
minicoli - E1C2_Fe_starve
minicoli - E1C2_LB
minicoli - E1C2_N_starve
minicoli - E1C2_cyt
minicoli - E1C2_glc
minicoli - E1C2_glyc
minicoli - E1C2_osm
minicoli - E1C2_ph5
minicoli - E1C2_pyr
minicoli - E1C2_sorb
minicoli - E1C2_urid
minicoli - F1C2-pyr
minicoli - F1C2_Fe_starve
minicoli - F1C2_LB
minicoli - F1C2_N_starve
minicoli - F1C2_cyt
minicoli - F1C2_fruc
minicoli - F1C2_glc
minicoli - F1C2_glyc
minicoli - F1C2_osm
minicoli - F1C2_ph5
minicoli - F1C2_sorb
minicoli - F1C2_urid
oxidative - deloxyr_pq
oxidative - delsoxr_pq
oxidative - delsoxs_pq
oxidative - wt_pq
Cytochrome c Gene Weight
Gene Start
1e6
nrfB
nrfC
nrfA
nrfD
nrfG
nrfE
Energy production and conversion
Inorganic ion transport and metabolism
Post-translational modification, protein turnover,and chaperones

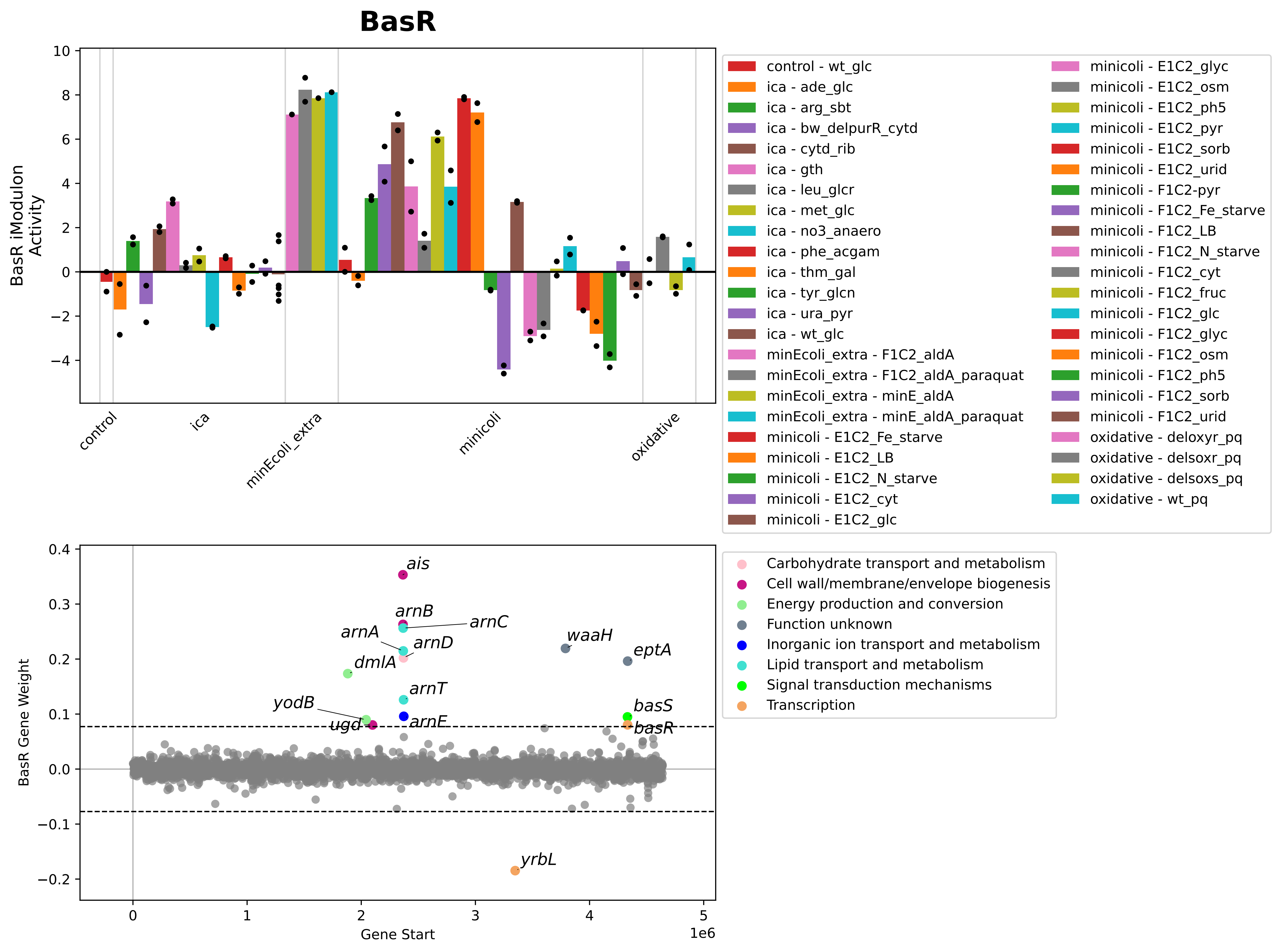

BasR
BasR iModulon Activity
control
ica
minEcoli_extra
minicoli
oxidative
control - wt_glc
ica - ade_glc
ica - arg_sbt
ica - bw_delpurR_cytd
ica - cytd_rib
ica - gth
ica - leu_glcr
ica - met_glc
ica - no3_anaero
ica - phe_acgam
ica - thm_gal
ica - tyr_glcn
ica - ura_pyr
ica - wt_glc
minEcoli_extra - F1C2_aldA
minEcoli_extra - F1C2_aldA_paraquat
minEcoli_extra - minE_aldA
minEcoli_extra - minE_aldA_paraquat
minicoli - E1C2_Fe_starve
minicoli - E1C2_LB
minicoli - E1C2_N_starve
minicoli - E1C2_cyt
minicoli - E1C2_glc
minicoli - E1C2_glyc
minicoli - E1C2_osm
minicoli - E1C2_ph5
minicoli - E1C2_pyr
minicoli - E1C2_sorb
minicoli - E1C2_urid
minicoli - F1C2-pyr
minicoli - F1C2_Fe_starve
minicoli - F1C2_LB
minicoli - F1C2_N_starve
minicoli - F1C2_cyt
minicoli - F1C2_fruc
minicoli - F1C2_glc
minicoli - F1C2_glyc
minicoli - F1C2_osm
minicoli - F1C2_ph5
minicoli - F1C2_sorb
minicoli - F1C2_urid
oxidative - deloxyr_pq
oxidative - delsoxr_pq
oxidative - delsoxs_pq
oxidative - wt_pq
BasR Gene Weight
Gene Start
1e6
ais
arnB
arnC
arnA
arnD
dmlA
waaH
eptA
arnT
yodB
basS
ugd
arnE
basR
yrbL
Carbohydrate transport and metabolism
Cell wall/membrane/envelope biogenesis
Energy production and conversion
Function unknown
Inorganic ion transport and metabolism
Lipid transport and metabolism
Signal transduction mechanisms
Transcription

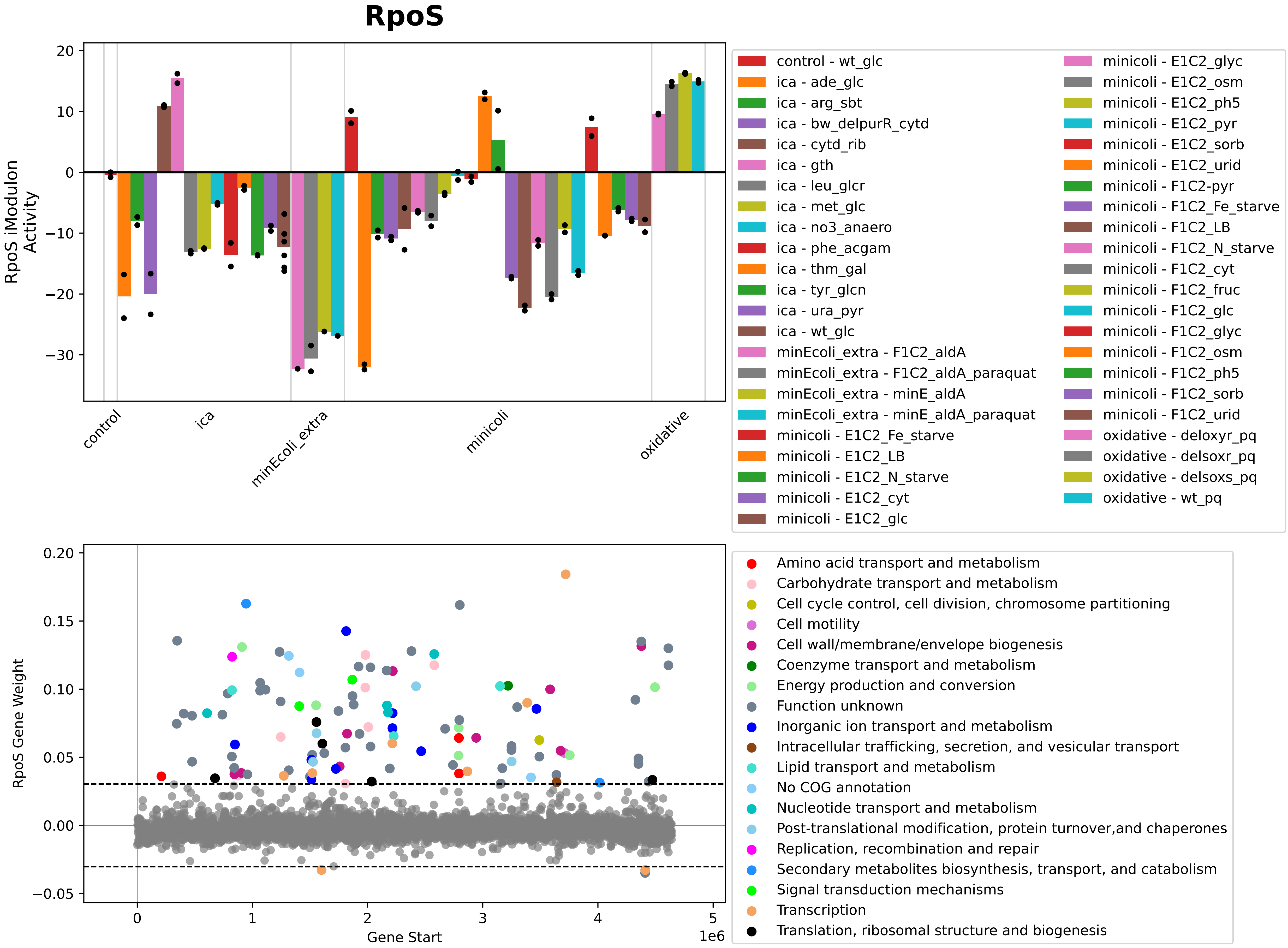
RpoS
RpoS iModulon Activity
20
10
0
−10
−20
−30
control
ica
minEcoli_extra
minicoli
oxidative
control - wt_glc
ica - ade_glc
ica - arg_sbt
ica - bw_delpurR_cytd
ica - cytd_rib
ica - gth
ica - leu_glcr
ica - met_glc
ica - no3_anaero
ica - phe_acgam
ica - thm_gal
ica - tyr_glcn
ica - ura_pyr
ica - wt_glc
minEcoli_extra - F1C2_aldA
minEcoli_extra - F1C2_aldA_paraquat
minEcoli_extra - minE_aldA
minEcoli_extra - minE_aldA_paraquat
minicoli - E1C2_Fe_starve
minicoli - E1C2_LB
minicoli - E1C2_N_starve
minicoli - E1C2_cyt
minicoli - E1C2_glc
minicoli - E1C2_glyc
minicoli - E1C2_osm
minicoli - E1C2_ph5
minicoli - E1C2_pyr
minicoli - E1C2_sorb
minicoli - E1C2_urid
minicoli - F1C2-pyr
minicoli - F1C2_Fe_starve
minicoli - F1C2_LB
minicoli - F1C2_N_starve
minicoli - F1C2_cyt
minicoli - F1C2_fruc
minicoli - F1C2_glc
minicoli - F1C2_glyc
minicoli - F1C2_osm
minicoli - F1C2_ph5
minicoli - F1C2_sorb
minicoli - F1C2_urid
oxidative - deloxyr_pq
oxidative - delsoxr_pq
oxidative - delsoxs_pq
oxidative - wt_pq
RpoS Gene Weight
0.20
0.15
0.10
0.05
0.00
−0.05
0
1
2
3
4
5
1e6
Gene Start
Amino acid transport and metabolism
Carbohydrate transport and metabolism
Cell cycle control, cell division, chromosome partitioning
Cell motility
Cell wall/membrane/envelope biogenesis
Coenzyme transport and metabolism
Energy production and conversion
Function unknown
Inorganic ion transport and metabolism
Intracellular trafficking, secretion, and vesicular transport
Lipid transport and metabolism
No COG annotation
Nucleotide transport and metabolism
Post-translational modification, protein turnover,and chaperones
Replication, recombination and repair
Secondary metabolites biosynthesis, transport, and catabolism
Signal transduction mechanisms
Transcription
Translation, ribosomal structure and biogenesis

lsrB KO

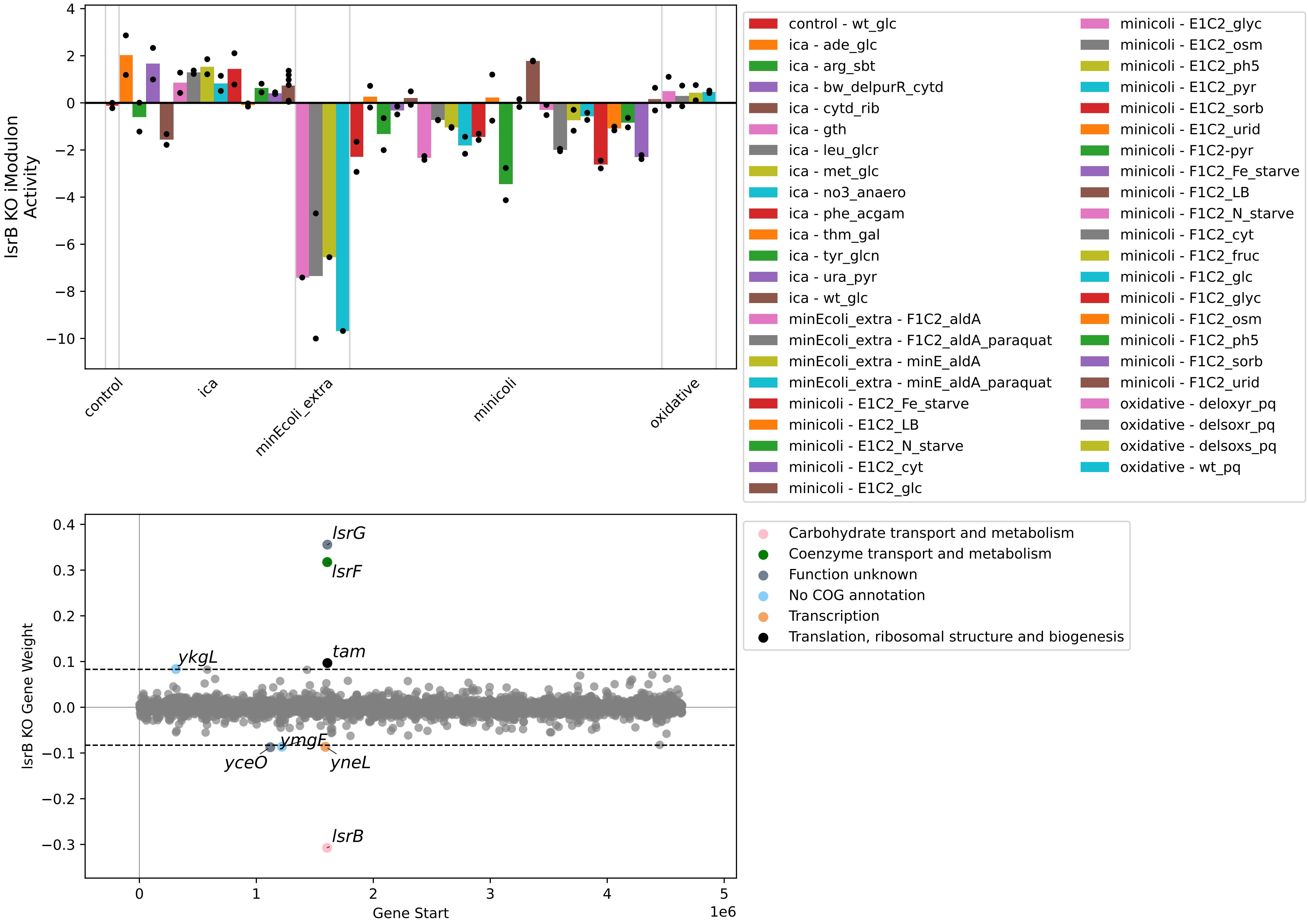

lsrB KO iModulon Activity
control
ica
minEcoli_extra
minicoli
oxidative
control - wt_glc
ica - ade_glc
ica - arg_sbt
ica - bw_delpurR_cytd
ica - cytd_rib
ica - gth
ica - leu_glcr
ica - met_glc
ica - no3_anaero
ica - phe_acgam
ica - thm_gal
ica - tyr_glcn
ica - ura_pyr
ica - wt_glc
minEcoli_extra - F1C2_aldA
minEcoli_extra - F1C2_aldA_paraquat
minEcoli_extra - minE_aldA
minEcoli_extra - minE_aldA_paraquat
minicoli - E1C2_Fe_starve
minicoli - E1C2_LB
minicoli - E1C2_N_starve
minicoli - E1C2_cyt
minicoli - E1C2_glc
minicoli - E1C2_glyc
minicoli - E1C2_osm
minicoli - E1C2_ph5
minicoli - E1C2_pyr
minicoli - E1C2_sorb
minicoli - E1C2_urid
minicoli - F1C2-pyr
minicoli - F1C2_Fe_starve
minicoli - F1C2_LB
minicoli - F1C2_N_starve
minicoli - F1C2_cyt
minicoli - F1C2_fruc
minicoli - F1C2_glc
minicoli - F1C2_glyc
minicoli - F1C2_osm
minicoli - F1C2_ph5
minicoli - F1C2_sorb
minicoli - F1C2_urid
oxidative - deloxyr_pq
oxidative - delsoxr_pq
oxidative - delsoxs_pq
oxidative - wt_pq
lsrG
lsrF
tam
ykgL
ymgF
yceO
yneL
lsrB
lsrB KO Gene Weight
Gene Start
1e6
Carbohydrate transport and metabolism
Coenzyme transport and metabolism
Function unknown
No COG annotation
Transcription
Translation, ribosomal structure and biogenesis

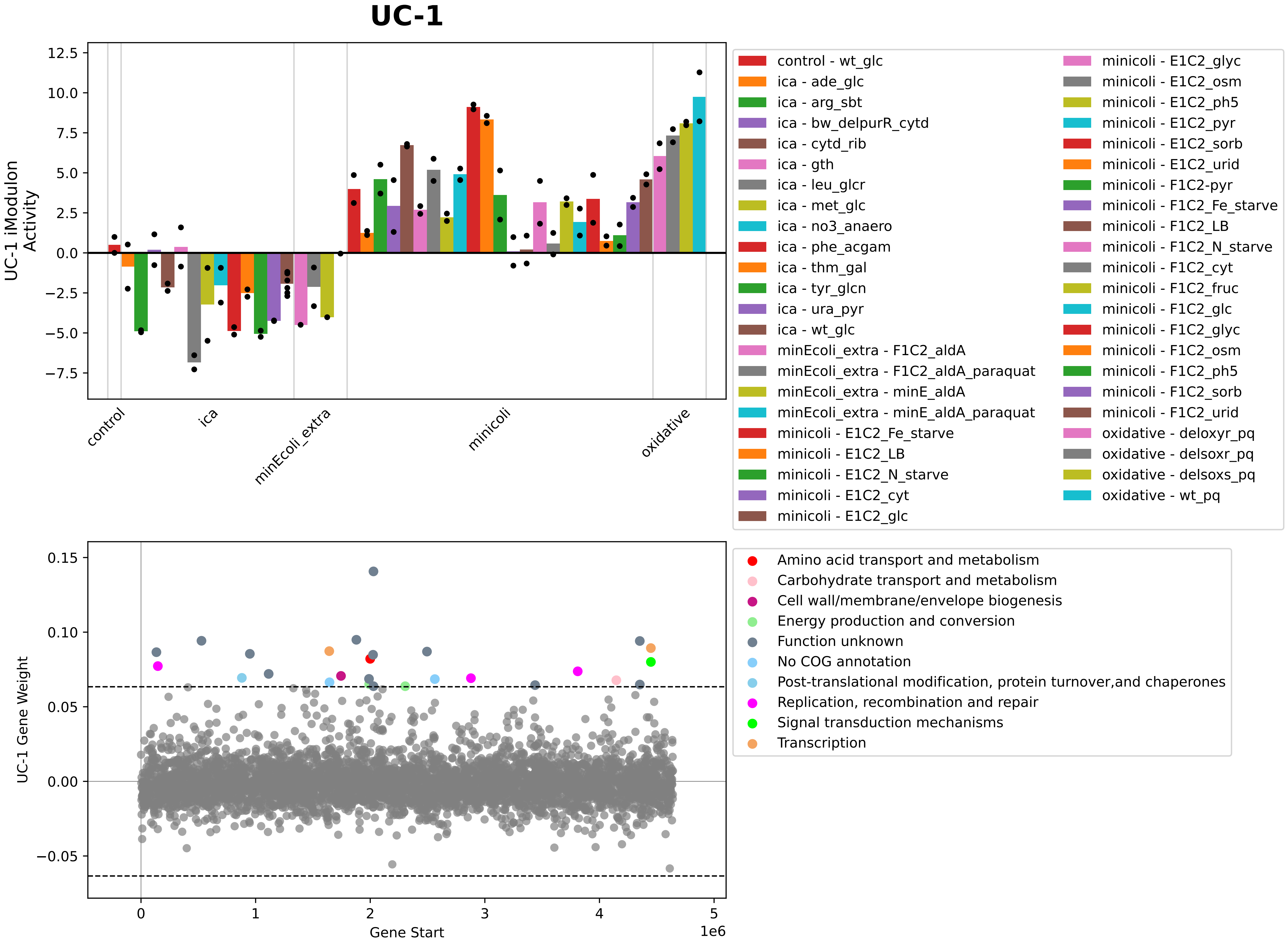

UC-1
UC-1 iModulon Activity
12.5
10.0
7.5
5.0
2.5
0.0
−2.5
−5.0
−7.5
control
ica
minEcoli_extra
minicoli
oxidative
control - wt_glc
ica - ade_glc
ica - arg_sbt
ica - bw_delpurR_cytd
ica - cytd_rib
ica - gth
ica - leu_glcr
ica - met_glc
ica - no3_anaero
ica - phe_acgam
ica - thm_gal
ica - tyr_glcn
ica - ura_pyr
ica - wt_glc
minEcoli_extra - F1C2_aldA
minEcoli_extra - F1C2_aldA_paraquat
minEcoli_extra - minE_aldA
minEcoli_extra - minE_aldA_paraquat
minicoli - E1C2_Fe_starve
minicoli - E1C2_LB
minicoli - E1C2_N_starve
minicoli - E1C2_cyt
minicoli - E1C2_glc
minicoli - E1C2_glyc
minicoli - E1C2_osm
minicoli - E1C2_ph5
minicoli - E1C2_pyr
minicoli - E1C2_sorb
minicoli - E1C2_urid
minicoli - F1C2-pyr
minicoli - F1C2_Fe_starve
minicoli - F1C2_LB
minicoli - F1C2_N_starve
minicoli - F1C2_cyt
minicoli - F1C2_fruc
minicoli - F1C2_glc
minicoli - F1C2_glyc
minicoli - F1C2_osm
minicoli - F1C2_ph5
minicoli - F1C2_sorb
minicoli - F1C2_urid
oxidative - deloxyr_pq
oxidative - delsoxr_pq
oxidative - delsoxs_pq
oxidative - wt_pq
UC-1 Gene Weight
0.15
0.10
0.05
0.00
−0.05
0
1
2
3
4
5
1e6
Gene Start
Amino acid transport and metabolism
Carbohydrate transport and metabolism
Cell wall/membrane/envelope biogenesis
Energy production and conversion
Function unknown
No COG annotation
Post-translational modification, protein turnover,and chaperones
Replication, recombination and repair
Signal transduction mechanisms
Transcription

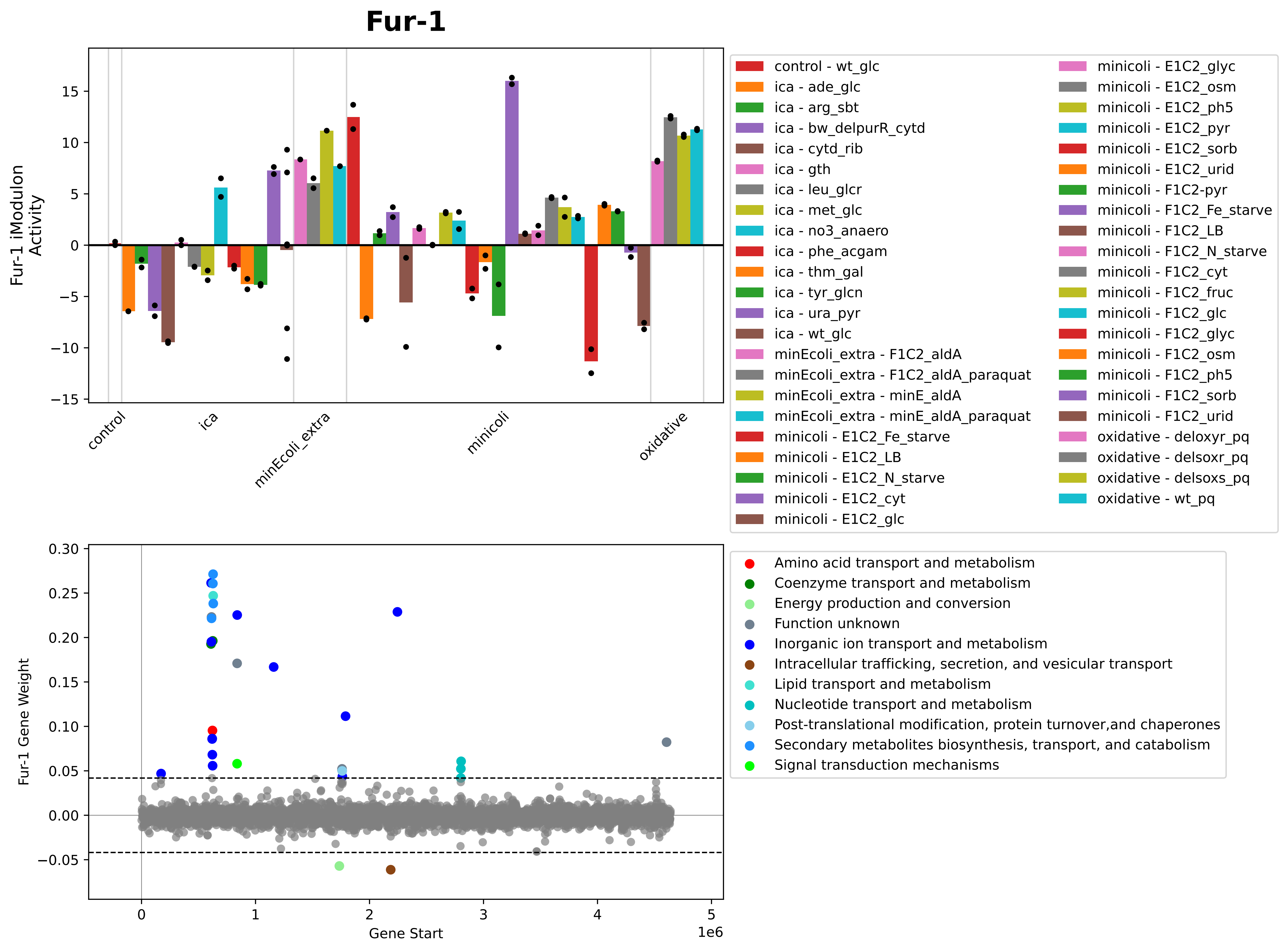
Fur-1
Fur-1 iModulon Activity
control
ica
minEcoli_extra
minicoli
oxidative
control - wt_glc
ica - ade_glc
ica - arg_sbt
ica - bw_delpurR_cytd
ica - cytd_rib
ica - gth
ica - leu_glcr
ica - met_glc
ica - no3_anaero
ica - phe_acgam
ica - thm_gal
ica - tyr_glcn
ica - ura_pyr
ica - wt_glc
minEcoli_extra - F1C2_aldA
minEcoli_extra - F1C2_aldA_paraquat
minEcoli_extra - minE_aldA
minEcoli_extra - minE_aldA_paraquat
minicoli - E1C2_Fe_starve
minicoli - E1C2_LB
minicoli - E1C2_N_starve
minicoli - E1C2_cyt
minicoli - E1C2_glc
minicoli - E1C2_glyc
minicoli - E1C2_osm
minicoli - E1C2_ph5
minicoli - E1C2_pyr
minicoli - E1C2_sorb
minicoli - E1C2_urid
minicoli - F1C2-pyr
minicoli - F1C2_Fe_starve
minicoli - F1C2_LB
minicoli - F1C2_N_starve
minicoli - F1C2_cyt
minicoli - F1C2_fruc
minicoli - F1C2_glc
minicoli - F1C2_glyc
minicoli - F1C2_osm
minicoli - F1C2_ph5
minicoli - F1C2_sorb
minicoli - F1C2_urid
oxidative - deloxyr_pq
oxidative - delsoxr_pq
oxidative - delsoxs_pq
oxidative - wt_pq
Fur-1 Gene Weight
Gene Start
1e6
Amino acid transport and metabolism
Coenzyme transport and metabolism
Energy production and conversion
Function unknown
Inorganic ion transport and metabolism
Intracellular trafficking, secretion, and vesicular transport
Lipid transport and metabolism
Nucleotide transport and metabolism
Post-translational modification, protein turnover,and chaperones
Secondary metabolites biosynthesis, transport, and catabolism
Signal transduction mechanisms

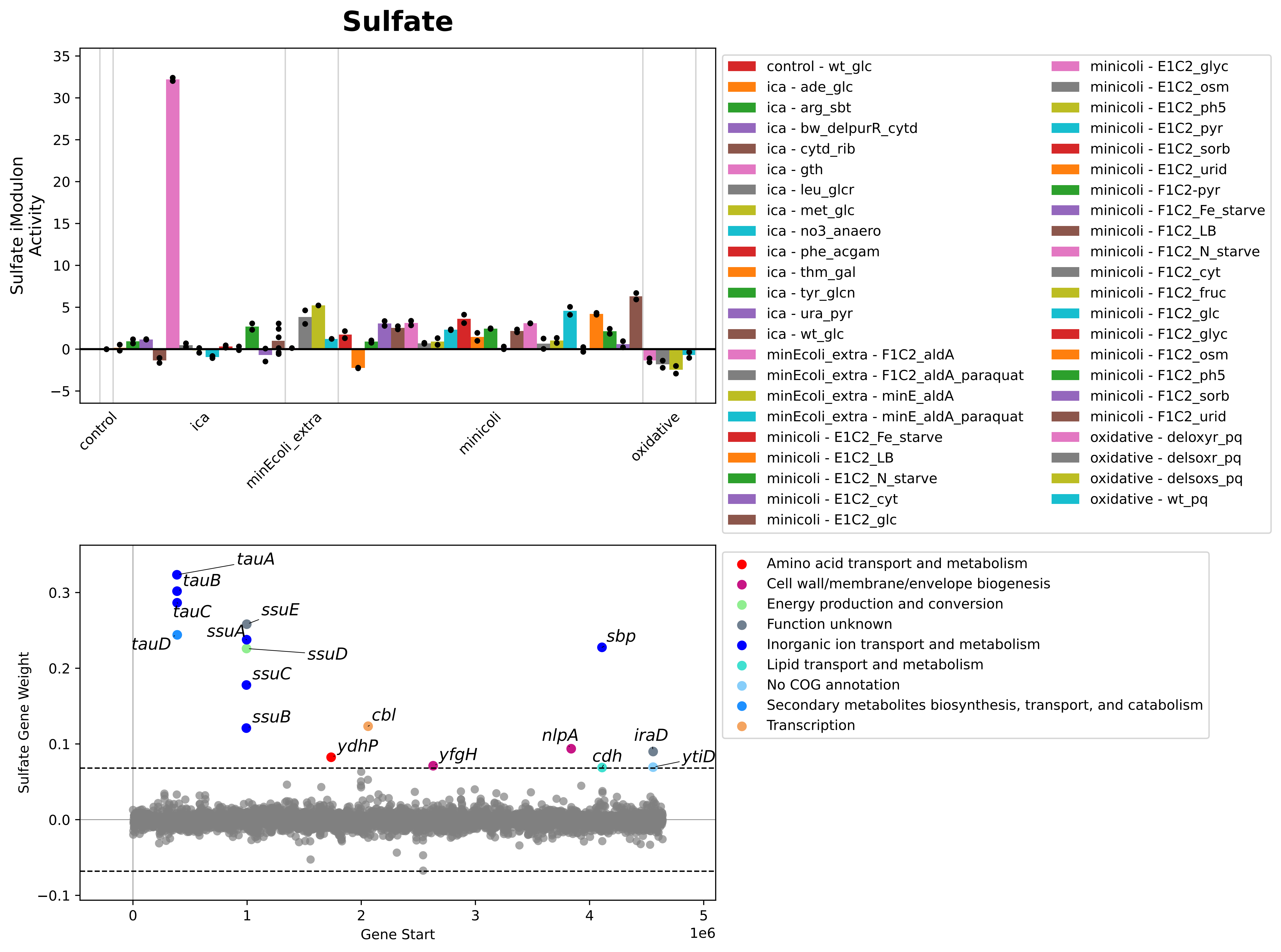

Sulfate
Sulfate iModulon Activity
control
ica
minEcoli_extra
minicoli
oxidative
control - wt_glc
ica - ade_glc
ica - arg_sbt
ica - bw_delpurR_cytd
ica - cytd_rib
ica - gth
ica - leu_glcr
ica - met_glc
ica - no3_anaero
ica - phe_acgam
ica - thm_gal
ica - tyr_glcn
ica - ura_pyr
ica - wt_glc
minEcoli_extra - F1C2_aldA
minEcoli_extra - F1C2_aldA_paraquat
minEcoli_extra - minE_aldA
minEcoli_extra - minE_aldA_paraquat
minicoli - E1C2_Fe_starve
minicoli - E1C2_LB
minicoli - E1C2_N_starve
minicoli - E1C2_cyt
minicoli - E1C2_glc
minicoli - E1C2_glyc
minicoli - E1C2_osm
minicoli - E1C2_ph5
minicoli - E1C2_pyr
minicoli - E1C2_sorb
minicoli - E1C2_urid
minicoli - F1C2-pyr
minicoli - F1C2_Fe_starve
minicoli - F1C2_LB
minicoli - F1C2_N_starve
minicoli - F1C2_cyt
minicoli - F1C2_fruc
minicoli - F1C2_glc
minicoli - F1C2_glyc
minicoli - F1C2_osm
minicoli - F1C2_ph5
minicoli - F1C2_sorb
minicoli - F1C2_urid
oxidative - deloxyr_pq
oxidative - delsoxr_pq
oxidative - delsoxs_pq
oxidative - wt_pq
Sulfate Gene Weight
Gene Start
1e6
tauA
tauB
tauC
tauD
ssuE
ssuA
ssuD
ssuC
ssuB
sbp
cbl
ydhP
yfgH
nlpA
iraD
cdh
ytiD
Amino acid transport and metabolism
Cell wall/membrane/envelope biogenesis
Energy production and conversion
Function unknown
Inorganic ion transport and metabolism
Lipid transport and metabolism
No COG annotation
Secondary metabolites biosynthesis, transport, and catabolism
Transcription

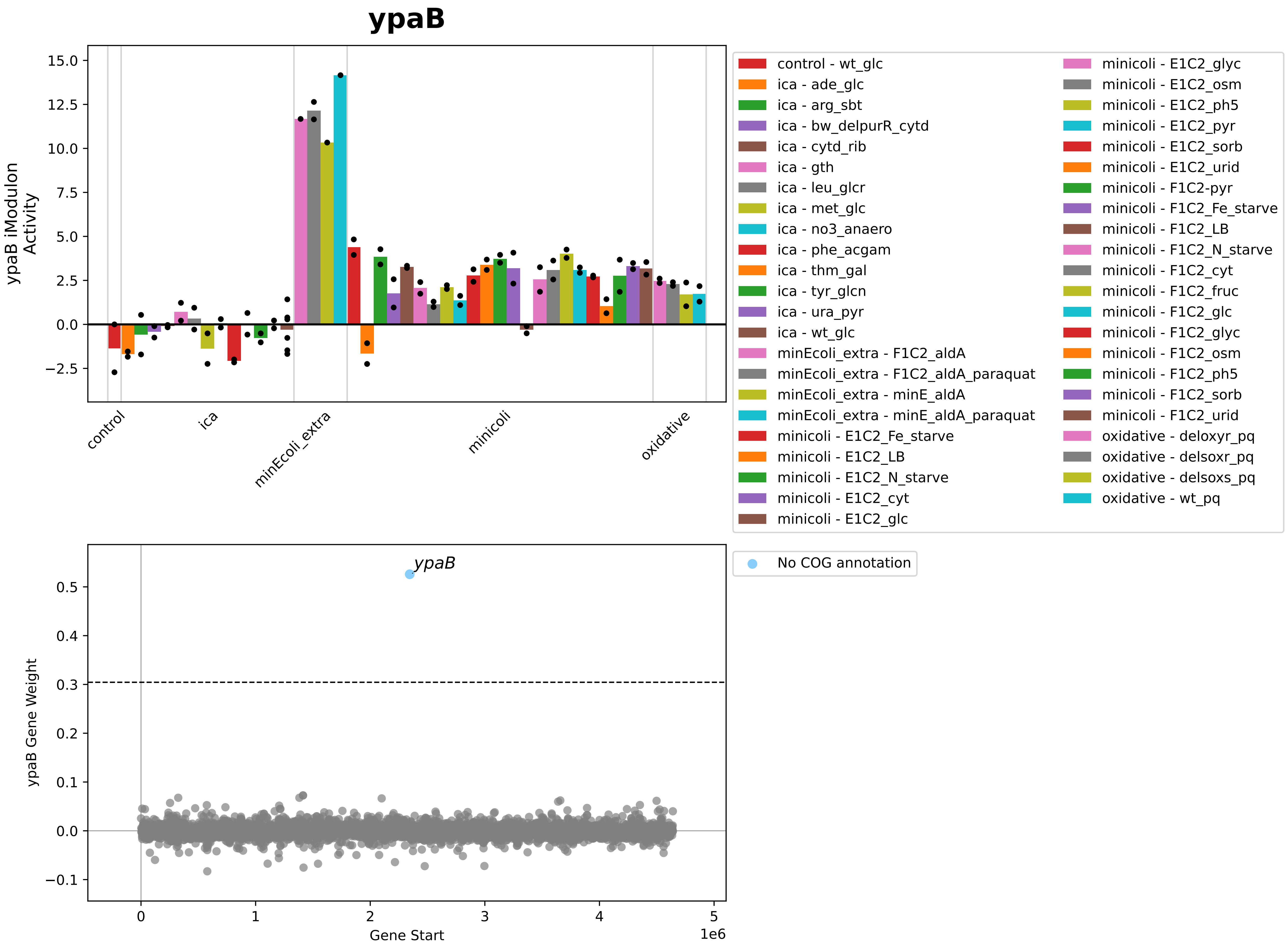
ypaB
ypaB iModulon Activity
control
ica
minEcoli_extra
minicoli
oxidative
control - wt_glc
ica - ade_glc
ica - arg_sbt
ica - bw_delpurR_cytd
ica - cytd_rib
ica - gth
ica - leu_glcr
ica - met_glc
ica - no3_anaero
ica - phe_acgam
ica - thm_gal
ica - tyr_glcn
ica - ura_pyr
ica - wt_glc
minEcoli_extra - F1C2_aldA
minEcoli_extra - F1C2_aldA_paraquat
minEcoli_extra - minE_aldA
minEcoli_extra - minE_aldA_paraquat
minicoli - E1C2_Fe_starve
minicoli - E1C2_LB
minicoli - E1C2_N_starve
minicoli - E1C2_cyt
minicoli - E1C2_glc
minicoli - E1C2_glyc
minicoli - E1C2_osm
minicoli - E1C2_ph5
minicoli - E1C2_pyr
minicoli - E1C2_sorb
minicoli - E1C2_urid
minicoli - F1C2-pyr
minicoli - F1C2_Fe_starve
minicoli - F1C2_LB
minicoli - F1C2_N_starve
minicoli - F1C2_cyt
minicoli - F1C2_fruc
minicoli - F1C2_glc
minicoli - F1C2_glyc
minicoli - F1C2_osm
minicoli - F1C2_ph5
minicoli - F1C2_sorb
minicoli - F1C2_urid
oxidative - deloxyr_pq
oxidative - delsoxr_pq
oxidative - delsoxs_pq
oxidative - wt_pq
ypaB
No COG annotation
ypaB Gene Weight
Gene Start
1e6

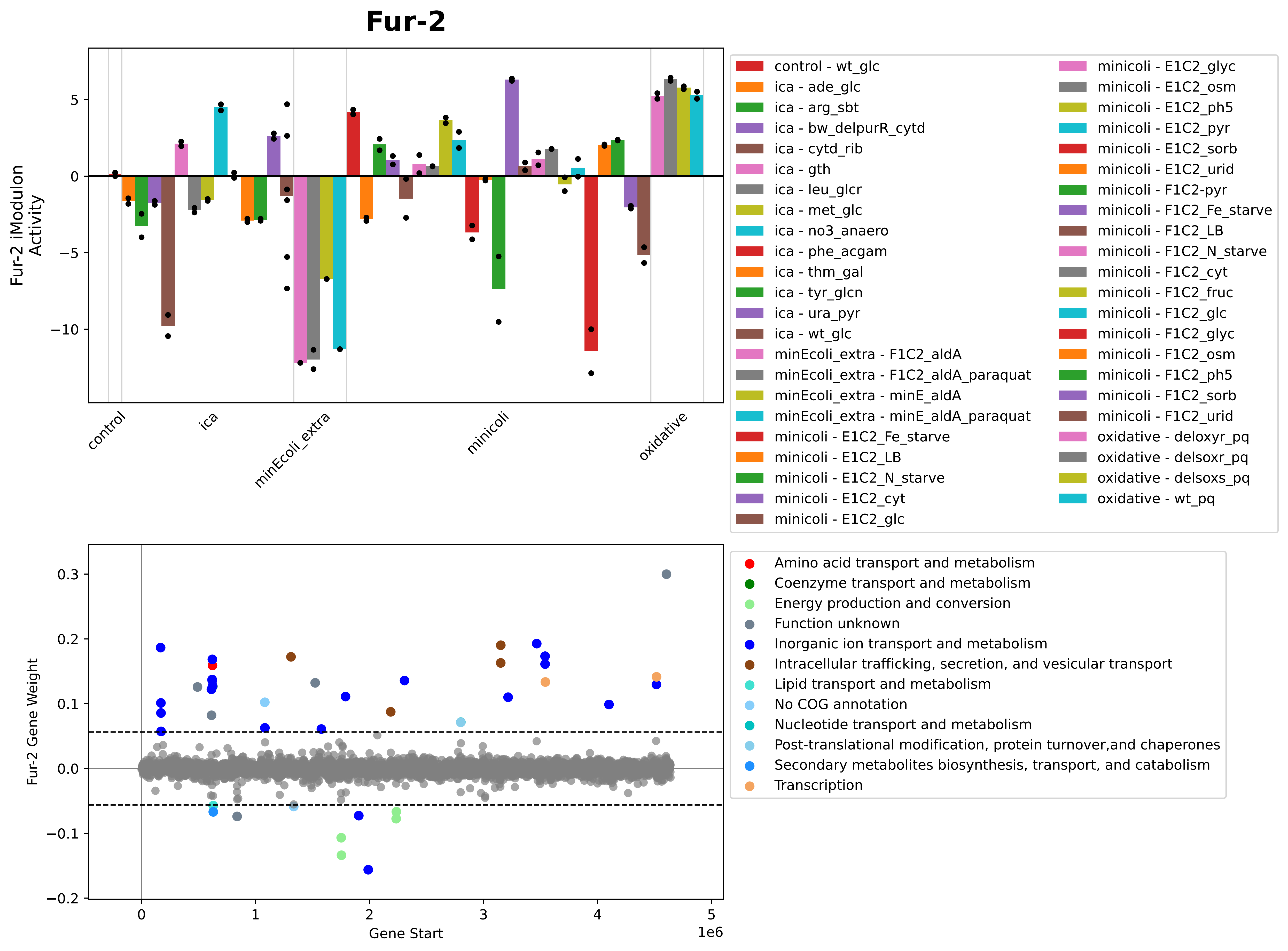

Fur-2
Fur-2 iModulon Activity
control
ica
minEcoli_extra
minicoli
oxidative
control - wt_glc
ica - ade_glc
ica - arg_sbt
ica - bw_delpurR_cytd
ica - cytd_rib
ica - gth
ica - leu_glcr
ica - met_glc
ica - no3_anaero
ica - phe_acgam
ica - thm_gal
ica - tyr_glcn
ica - ura_pyr
ica - wt_glc
minEcoli_extra - F1C2_aldA
minEcoli_extra - F1C2_aldA_paraquat
minEcoli_extra - minE_aldA
minEcoli_extra - minE_aldA_paraquat
minicoli - E1C2_Fe_starve
minicoli - E1C2_LB
minicoli - E1C2_N_starve
minicoli - E1C2_cyt
minicoli - E1C2_glc
minicoli - E1C2_glyc
minicoli - E1C2_osm
minicoli - E1C2_ph5
minicoli - E1C2_pyr
minicoli - E1C2_sorb
minicoli - E1C2_urid
minicoli - F1C2-pyr
minicoli - F1C2_Fe_starve
minicoli - F1C2_LB
minicoli - F1C2_N_starve
minicoli - F1C2_cyt
minicoli - F1C2_fruc
minicoli - F1C2_glc
minicoli - F1C2_glycl
minicoli - F1C2_osm
minicoli - F1C2_ph5
minicoli - F1C2_sorb
minicoli - F1C2_urid
oxidative - deloxyr_pq
oxidative - delsoxr_pq
oxidative - delsoxs_pq
oxidative - wt_pq
Fur-2 Gene Weight
Gene Start
1e6
Amino acid transport and metabolism
Coenzyme transport and metabolism
Energy production and conversion
Function unknown
Inorganic ion transport and metabolism
Intracellular trafficking, secretion, and vesicular transport
Lipid transport and metabolism
No COG annotation
Nucleotide transport and metabolism
Post-translational modification, protein turnover,and chaperones
Secondary metabolites biosynthesis, transport, and catabolism
Transcription

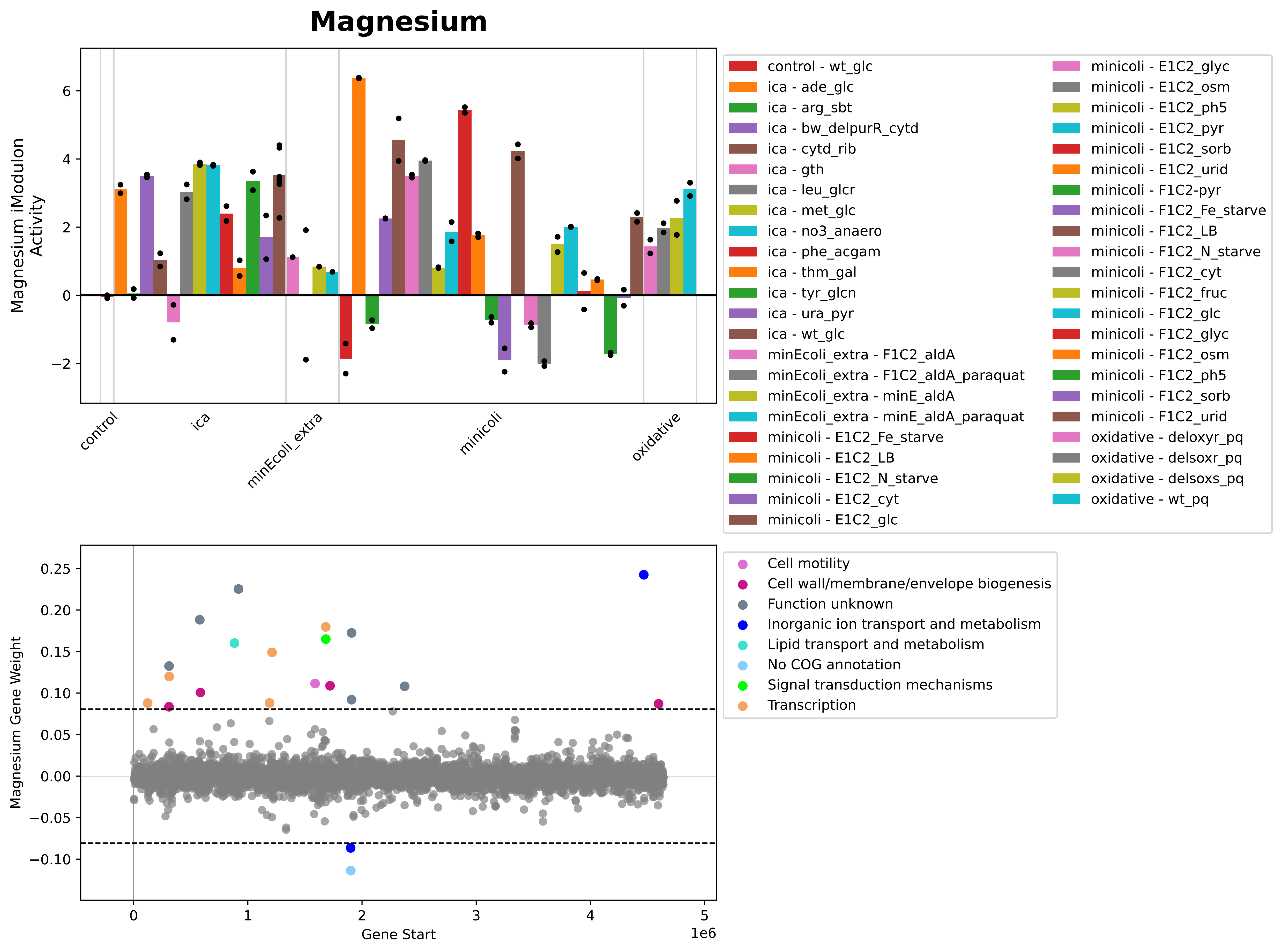

Magnesium
Magnesium iModulon Activity
control
ica
minEcoli_extra
minicoli
oxidative
control - wt_glc
ica - ade_glc
ica - arg_sbt
ica - bw_delpurR_cytd
ica - cytd_rib
ica - gth
ica - leu_glcr
ica - met_glc
ica - no3_anaero
ica - phe_acgam
ica - thm_gal
ica - tyr_glcn
ica - ura_pyr
ica - wt_glc
minEcoli_extra - F1C2_aldA
minEcoli_extra - F1C2_aldA_paraquat
minEcoli_extra - minE_aldA
minEcoli_extra - minE_aldA_paraquat
minicoli - E1C2_Fe_starve
minicoli - E1C2_LB
minicoli - E1C2_N_starve
minicoli - E1C2_cyt
minicoli - E1C2_glc
minicoli - E1C2_glyc
minicoli - E1C2_osm
minicoli - E1C2_ph5
minicoli - E1C2_pyr
minicoli - E1C2_sorb
minicoli - E1C2_urid
minicoli - F1C2-pyr
minicoli - F1C2_Fe_starve
minicoli - F1C2_LB
minicoli - F1C2_N_starve
minicoli - F1C2_cyt
minicoli - F1C2_fruc
minicoli - F1C2_glc
minicoli - F1C2_glyc
minicoli - F1C2_osm
minicoli - F1C2_ph5
minicoli - F1C2_sorb
minicoli - F1C2_urid
oxidative - deloxyr_pq
oxidative - delsoxr_pq
oxidative - delsoxs_pq
oxidative - wt_pq
Magnesium Gene Weight
Gene Start
1e6
Cell motility
Cell wall/membrane/envelope biogenesis
Function unknown
Inorganic ion transport and metabolism
Lipid transport and metabolism
No COG annotation
Signal transduction mechanisms
Transcription

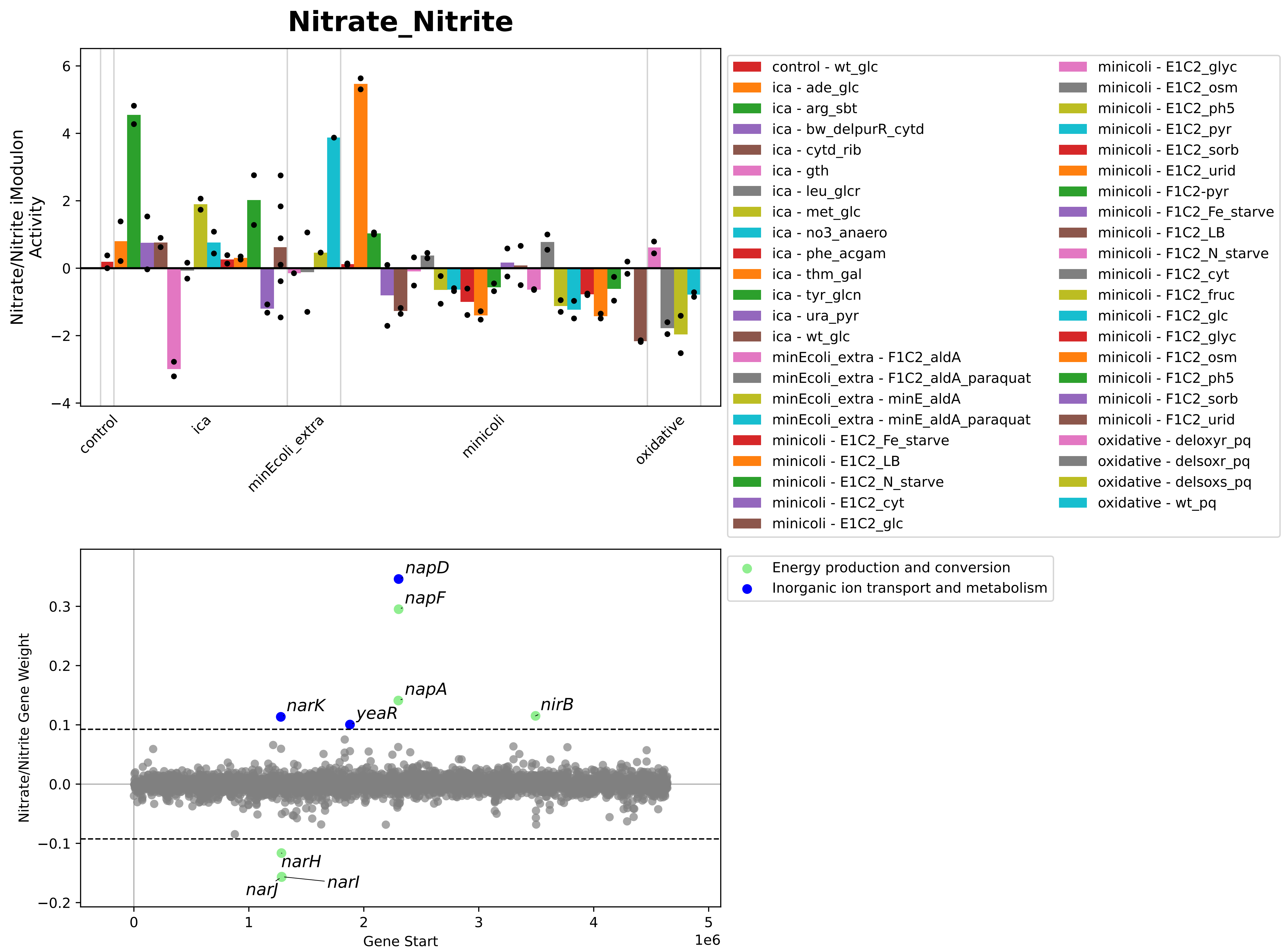

Nitrate_Nitrite
Nitrate/Nitrite iModulon Activity
control
ica
minEcoli_extra
minicoli
oxidative
control - wt_glc
ica - ade_glc
ica - arg_sbt
ica - bw_delpurR_cytd
ica - cytd_rib
ica - gth
ica - leu_glcr
ica - met_glc
ica - no3_anaero
ica - phe_acgam
ica - thm_gal
ica - tyr_glcn
ica - ura_pyr
ica - wt_glc
minEcoli_extra - F1C2_aldA
minEcoli_extra - F1C2_aldA_paraquat
minEcoli_extra - minE_aldA
minEcoli_extra - minE_aldA_paraquat
minicoli - E1C2_Fe_starve
minicoli - E1C2_LB
minicoli - E1C2_N_starve
minicoli - E1C2_cyt
minicoli - E1C2_glc
minicoli - E1C2_glyc
minicoli - E1C2_osm
minicoli - E1C2_ph5
minicoli - E1C2_pyr
minicoli - E1C2_sorb
minicoli - E1C2_urid
minicoli - F1C2-pyr
minicoli - F1C2_Fe_starve
minicoli - F1C2_LB
minicoli - F1C2_N_starve
minicoli - F1C2_cyt
minicoli - F1C2_fruc
minicoli - F1C2_glc
minicoli - F1C2_glyc
minicoli - F1C2_osm
minicoli - F1C2_ph5
minicoli - F1C2_sorb
minicoli - F1C2_urid
oxidative - deloxyr_pq
oxidative - delsoxr_pq
oxidative - delsoxs_pq
oxidative - wt_pq
Nitrate/Nitrite Gene Weight
napD
napF
napA
narK
yeaR
nirB
narH
narJ
narI
Gene Start
1e6
Energy production and conversion
Inorganic ion transport and metabolism

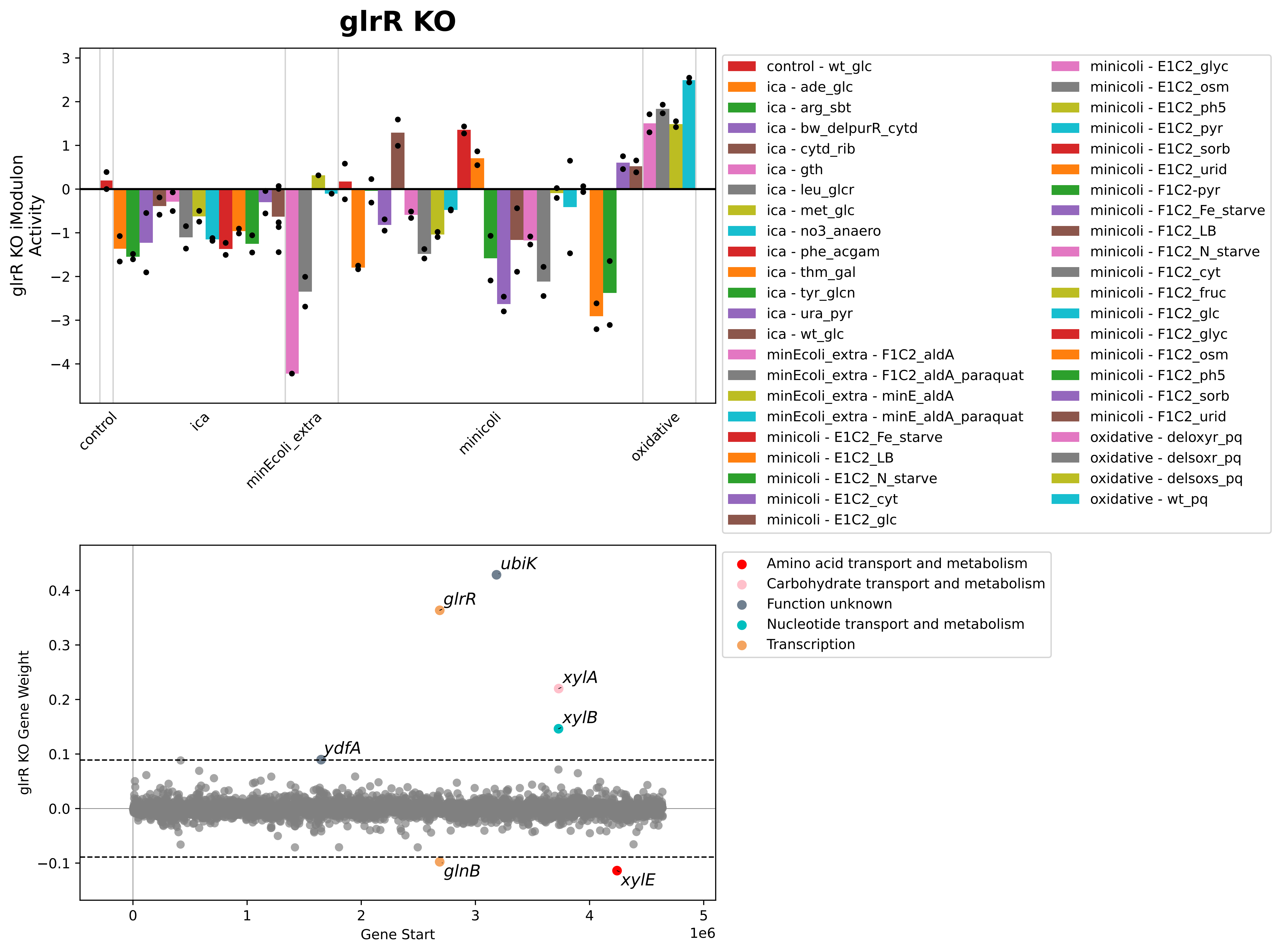
glrR KO
glrR KO iModulon Activity
control
ica
minEcoli_extra
minicoli
oxidative
control - wt_glc
ica - ade_glc
ica - arg_sbt
ica - bw_delpurR_cytd
ica - cytd_rib
ica - gth
ica - leu_glcr
ica - met_glc
ica - no3_anaero
ica - phe_acgam
ica - thm_gal
ica - tyr_glcn
ica - ura_pyr
ica - wt_glc
minEcoli_extra - F1C2_aldA
minEcoli_extra - F1C2_aldA_paraquat
minEcoli_extra - minE_aldA
minEcoli_extra - minE_aldA_paraquat
minicoli - E1C2_Fe_starve
minicoli - E1C2_LB
minicoli - E1C2_N_starve
minicoli - E1C2_cyt
minicoli - E1C2_glc
minicoli - E1C2_glyc
minicoli - E1C2_osm
minicoli - E1C2_ph5
minicoli - E1C2_pyr
minicoli - E1C2_sorb
minicoli - E1C2_urid
minicoli - F1C2-pyr
minicoli - F1C2_Fe_starve
minicoli - F1C2_LB
minicoli - F1C2_N_starve
minicoli - F1C2_cyt
minicoli - F1C2_fruc
minicoli - F1C2_glc
minicoli - F1C2_glyc
minicoli - F1C2_osm
minicoli - F1C2_ph5
minicoli - F1C2_sorb
minicoli - F1C2_urid
oxidative - deloxyr_pq
oxidative - delsoxr_pq
oxidative - delsoxs_pq
oxidative - wt_pq
glrR KO Gene Weight
Gene Start
1e6
ubiK
glrR
xylA
xylB
ydfA
glnB
xylE
Amino acid transport and metabolism
Carbohydrate transport and metabolism
Function unknown
Nucleotide transport and metabolism
Transcription

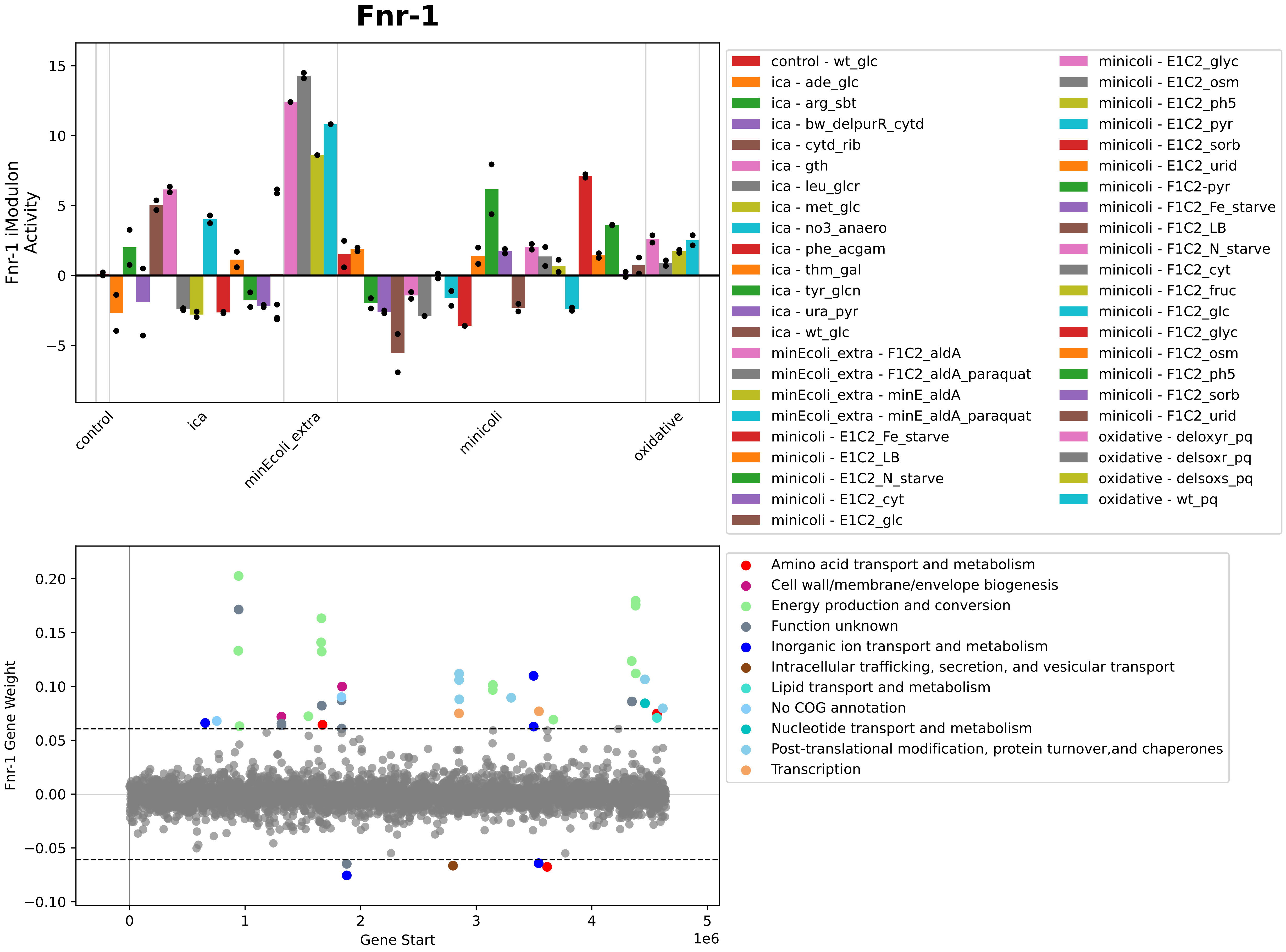

Fnr-1
Fnr-1 iModulon Activity
15
10
5
0
−5
control
ica
minEcoli_extra
minicoli
oxidative
control - wt_glc
ica - ade_glc
ica - arg_sbt
ica - bw_delpurR_cytd
ica - cytd_rib
ica - gth
ica - leu_glcr
ica - met_glc
ica - no3_anaero
ica - phe_acgam
ica - thm_gal
ica - tyr_glcn
ica - ura_pyr
ica - wt_glc
minEcoli_extra - F1C2_aldA
minEcoli_extra - F1C2_aldA_paraquat
minEcoli_extra - minE_aldA
minEcoli_extra - minE_aldA_paraquat
minicoli - E1C2_Fe_starve
minicoli - E1C2_LB
minicoli - E1C2_N_starve
minicoli - E1C2_cyt
minicoli - E1C2_glc
minicoli - E1C2_glyc
minicoli - E1C2_osm
minicoli - E1C2_ph5
minicoli - E1C2_pyr
minicoli - E1C2_sorb
minicoli - E1C2_urid
minicoli - F1C2-pyr
minicoli - F1C2_Fe_starve
minicoli - F1C2_LB
minicoli - F1C2_N_starve
minicoli - F1C2_cyt
minicoli - F1C2_fruc
minicoli - F1C2_glc
minicoli - F1C2_glyc
minicoli - F1C2_osm
minicoli - F1C2_ph5
minicoli - F1C2_sorb
minicoli - F1C2_urid
oxidative - deloxyr_pq
oxidative - delsoxr_pq
oxidative - delsoxs_pq
oxidative - wt_pq
Fnr-1 Gene Weight
0.20
0.15
0.10
0.05
0.00
−0.05
−0.10
0
1
2
3
4
5
Gene Start
1e6
Amino acid transport and metabolism
Cell wall/membrane/envelope biogenesis
Energy production and conversion
Function unknown
Inorganic ion transport and metabolism
Intracellular trafficking, secretion, and vesicular transport
Lipid transport and metabolism
No COG annotation
Nucleotide transport and metabolism
Post-translational modification, protein turnover,and chaperones
Transcription

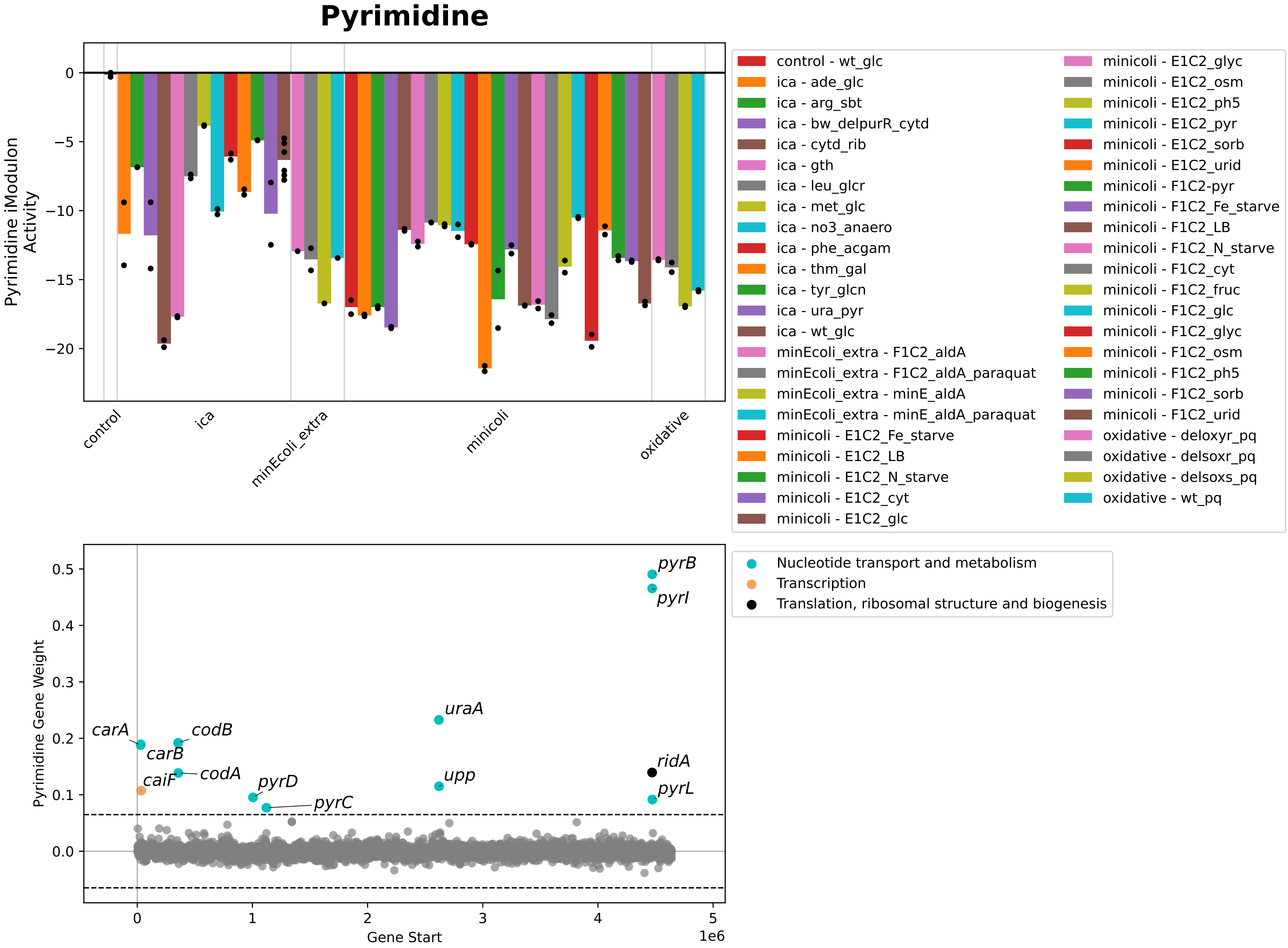

Pyrimidine
Pyrimidine iModulon Activity
0
−5
−10
−15
−20
control
ica
minEcoli_extra
minicoli
oxidative
control - wt_glc
ica - ade_glc
ica - arg_sbt
ica - bw_delpurR_cytd
ica - cytd_rib
ica - gth
ica - leu_glcr
ica - met_glc
ica - no3_anaero
ica - phe_acgam
ica - thm_gal
ica - tyr_glcn
ica - ura_pyr
ica - wt_glc
minEcoli_extra - F1C2_aldA
minEcoli_extra - F1C2_aldA_paraquat
minEcoli_extra - minE_aldA
minEcoli_extra - minE_aldA_paraquat
minicoli - E1C2_Fe_starve
minicoli - E1C2_LB
minicoli - E1C2_N_starve
minicoli - E1C2_cyt
minicoli - E1C2_glc
minicoli - E1C2_glyc
minicoli - E1C2_osm
minicoli - E1C2_ph5
minicoli - E1C2_pyr
minicoli - E1C2_sorb
minicoli - E1C2_urid
minicoli - F1C2-pyr
minicoli - F1C2_Fe_starve
minicoli - F1C2_LB
minicoli - F1C2_N_starve
minicoli - F1C2_cyt
minicoli - F1C2_fruc
minicoli - F1C2_glc
minicoli - F1C2_glyc
minicoli - F1C2_osm
minicoli - F1C2_ph5
minicoli - F1C2_sorb
minicoli - F1C2_urid
oxidative - deloxyr_pq
oxidative - delsoxr_pq
oxidative - delsoxs_pq
oxidative - wt_pq
Pyrimidine Gene Weight
0.5
0.4
0.3
0.2
0.1
0.0
pyrB
pyrI
uraA
carA
codB
carB
ridA
codA
upp
caiF
pyrD
pyrL
pyrC
0
1
2
3
4
5
1e6
Gene Start
Nucleotide transport and metabolism
Transcription
Translation, ribosomal structure and biogenesis

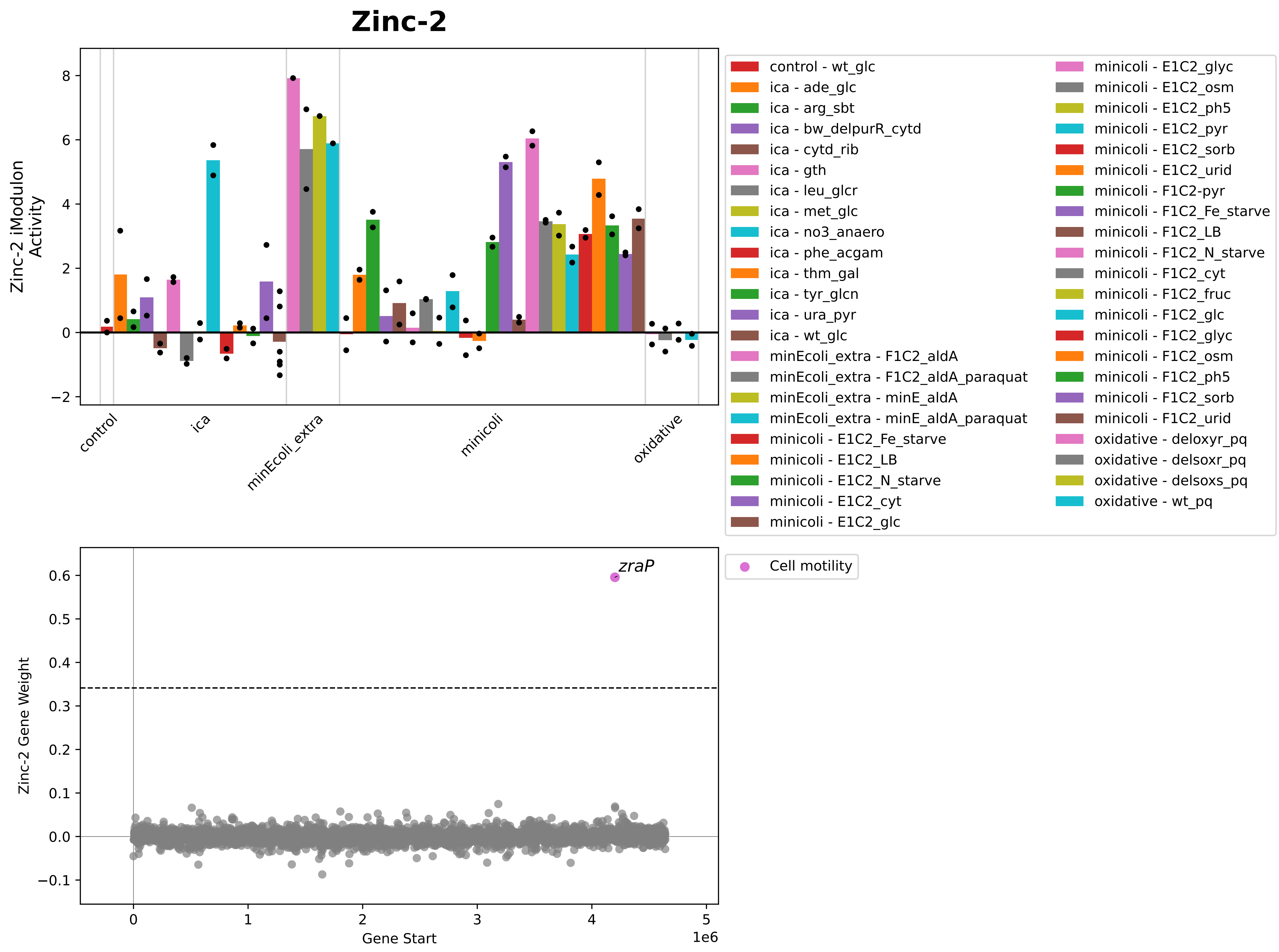

Zinc-2
Zinc-2 iModulon Activity
control
ica
minEcoli_extra
minicoli
oxidative
control - wt_glc
ica - ade_glc
ica - arg_sbt
ica - bw_delpurR_cytd
ica - cytd_rib
ica - gth
ica - leu_glcr
ica - met_glc
ica - no3_anaero
ica - phe_acgam
ica - thm_gal
ica - tyr_glcn
ica - ura_pyr
ica - wt_glc
minEcoli_extra - F1C2_aldA
minEcoli_extra - F1C2_aldA_paraquat
minEcoli_extra - minE_aldA
minEcoli_extra - minE_aldA_paraquat
minicoli - E1C2_Fe_starve
minicoli - E1C2_LB
minicoli - E1C2_N_starve
minicoli - E1C2_cyt
minicoli - E1C2_glc
minicoli - E1C2_glyc
minicoli - E1C2_osm
minicoli - E1C2_ph5
minicoli - E1C2_pyr
minicoli - E1C2_sorb
minicoli - E1C2_urid
minicoli - F1C2-pyr
minicoli - F1C2_Fe_starve
minicoli - F1C2_LB
minicoli - F1C2_N_starve
minicoli - F1C2_cyt
minicoli - F1C2_fruc
minicoli - F1C2_glc
minicoli - F1C2_glyc
minicoli - F1C2_osm
minicoli - F1C2_ph5
minicoli - F1C2_sorb
minicoli - F1C2_urid
oxidative - deloxyr_pq
oxidative - delsoxr_pq
oxidative - delsoxs_pq
oxidative - wt_pq
zraP
Cell motility
Zinc-2 Gene Weight
Gene Start
1e6

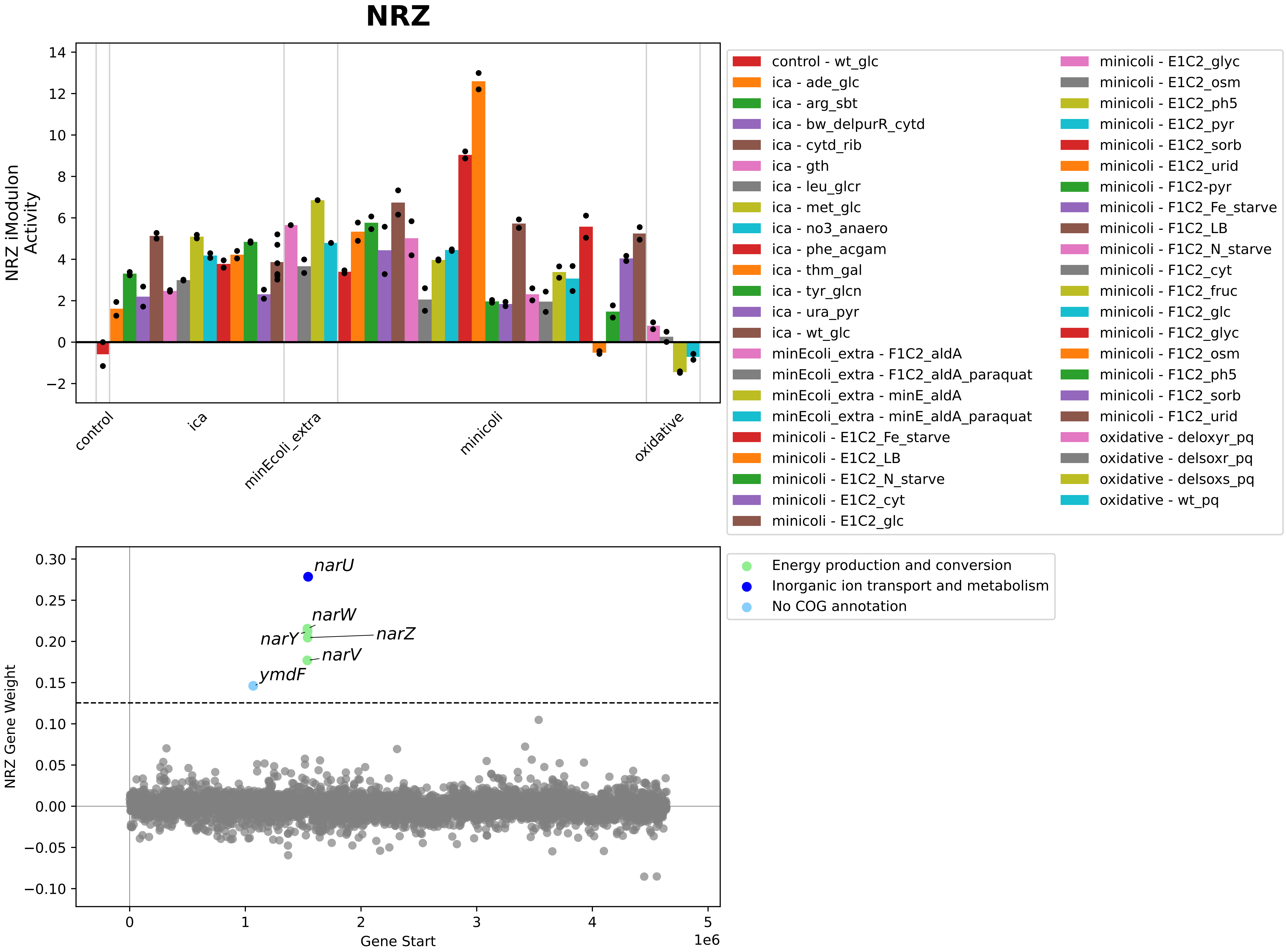
NRZ
NRZ iModulon Activity
control
ica
minEcoli_extra
minicoli
oxidative
control - wt_glc
ica - ade_glc
ica - arg_sbt
ica - bw_delpurR_cytd
ica - cytd_rib
ica - gth
ica - leu_glcr
ica - met_glc
ica - no3_anaero
ica - phe_acgam
ica - thm_gal
ica - tyr_glcn
ica - ura_pyr
ica - wt_glc
minEcoli_extra - F1C2_aldA
minEcoli_extra - F1C2_aldA_paraquat
minEcoli_extra - minE_aldA
minEcoli_extra - minE_aldA_paraquat
minicoli - E1C2_Fe_starve
minicoli - E1C2_LB
minicoli - E1C2_N_starve
minicoli - E1C2_cyt
minicoli - E1C2_glc
minicoli - E1C2_glyc
minicoli - E1C2_osm
minicoli - E1C2_ph5
minicoli - E1C2_pyr
minicoli - E1C2_sorb
minicoli - E1C2_urid
minicoli - F1C2-pyr
minicoli - F1C2_Fe_starve
minicoli - F1C2_LB
minicoli - F1C2_N_starve
minicoli - F1C2_cyt
minicoli - F1C2_fruc
minicoli - F1C2_glc
minicoli - F1C2_glyc
minicoli - F1C2_osm
minicoli - F1C2_ph5
minicoli - F1C2_sorb
minicoli - F1C2_urid
oxidative - deloxyr_pq
oxidative - delsoxr_pq
oxidative - delsoxs_pq
oxidative - wt_pq
NRZ Gene Weight
Gene Start
1e6
narU
narW
narY
narZ
narV
ymdF
Energy production and conversion
Inorganic ion transport and metabolism
No COG annotation

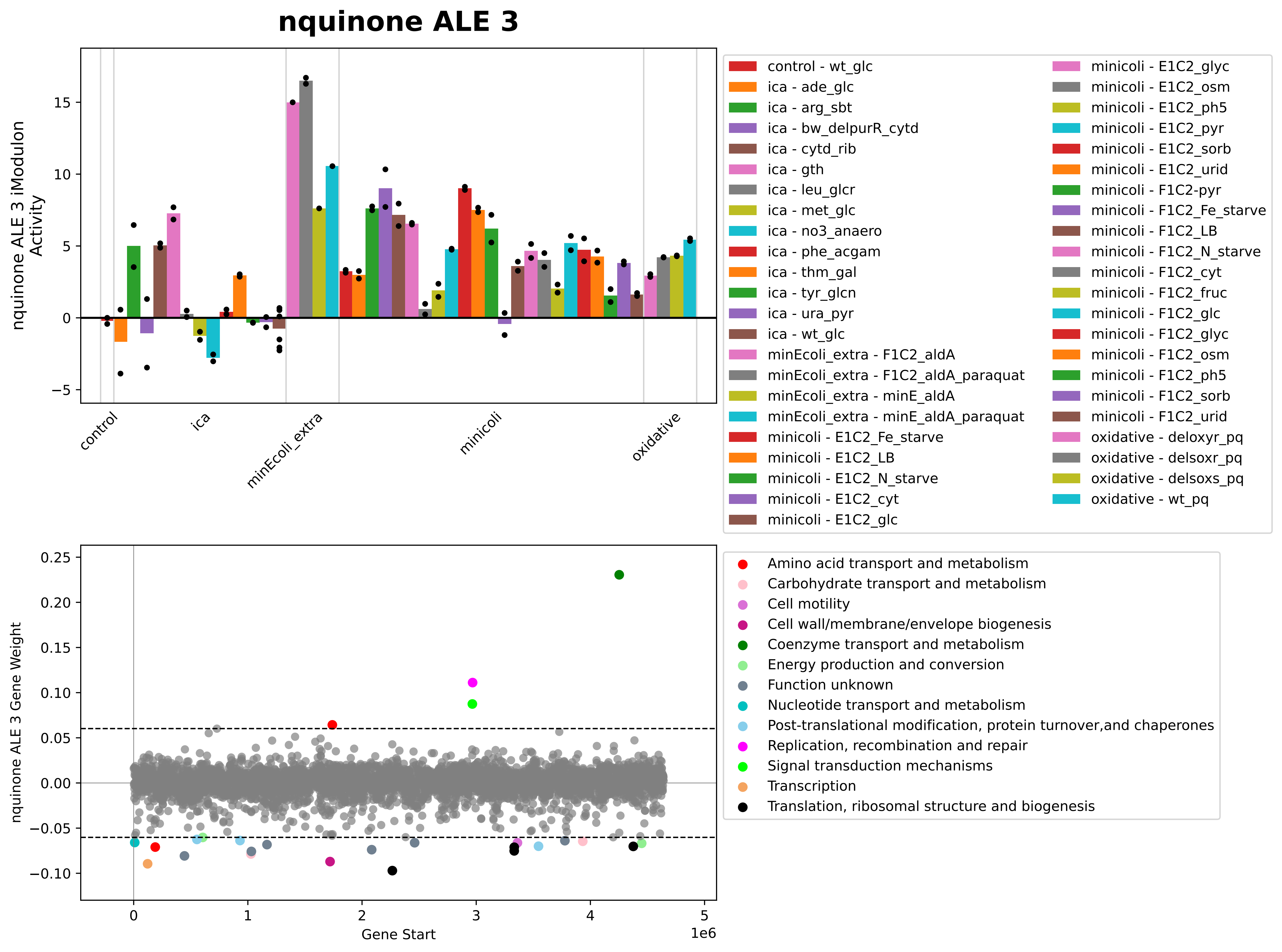

nquinone ALE 3
nquinone ALE 3 iModulon Activity
control
ica
minEcoli_extra
minicoli
oxidative
control - wt_glc
ica - ade_glc
ica - arg_sbt
ica - bw_delpurR_cytd
ica - cytd_rib
ica - gth
ica - leu_glcr
ica - met_glc
ica - no3_anaero
ica - phe_acgam
ica - thm_gal
ica - tyr_glcn
ica - ura_pyr
ica - wt_glc
minEcoli_extra - F1C2_aldA
minEcoli_extra - F1C2_aldA_paraquat
minEcoli_extra - minE_aldA
minEcoli_extra - minE_aldA_paraquat
minicoli - E1C2_Fe_starve
minicoli - E1C2_LB
minicoli - E1C2_N_starve
minicoli - E1C2_cyt
minicoli - E1C2_glc
minicoli - E1C2_glyc
minicoli - E1C2_osm
minicoli - E1C2_ph5
minicoli - E1C2_pyr
minicoli - E1C2_sorb
minicoli - E1C2_urid
minicoli - F1C2-pyr
minicoli - F1C2_Fe_starve
minicoli - F1C2_LB
minicoli - F1C2_N_starve
minicoli - F1C2_cyt
minicoli - F1C2_fruc
minicoli - F1C2_glc
minicoli - F1C2_glyc
minicoli - F1C2_osm
minicoli - F1C2_ph5
minicoli - F1C2_sorb
minicoli - F1C2_urid
oxidative - deloxyr_pq
oxidative - delsoxr_pq
oxidative - delsoxs_pq
oxidative - wt_pq
nquinone ALE 3 Gene Weight
Gene Start
1e6
Amino acid transport and metabolism
Carbohydrate transport and metabolism
Cell motility
Cell wall/membrane/envelope biogenesis
Coenzyme transport and metabolism
Energy production and conversion
Function unknown
Nucleotide transport and metabolism
Post-translational modification, protein turnover,and chaperones
Replication, recombination and repair
Signal transduction mechanisms
Transcription
Translation, ribosomal structure and biogenesis

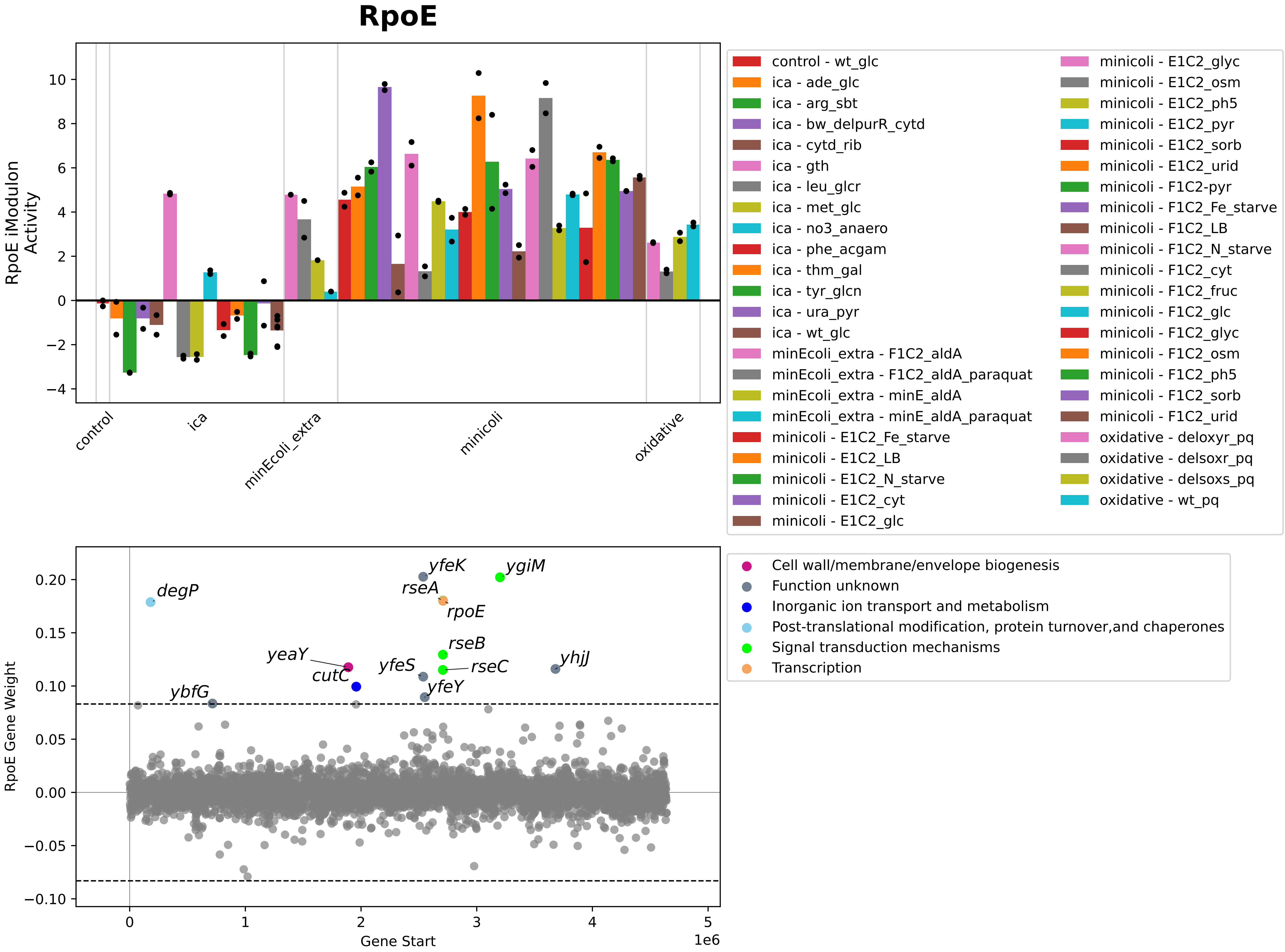
RpoE
RpoE iModulon Activity
control
ica
minEcoli_extra
minicoli
oxidative
control - wt_glc
ica - ade_glc
ica - arg_sbt
ica - bw_delpurR_cytd
ica - cytd_rib
ica - gth
ica - leu_glcr
ica - met_glc
ica - no3_anaero
ica - phe_acgam
ica - thm_gal
ica - tyr_glcn
ica - ura_pyr
ica - wt_glc
minEcoli_extra - F1C2_aldA
minEcoli_extra - F1C2_aldA_paraquat
minEcoli_extra - minE_aldA
minEcoli_extra - minE_aldA_paraquat
minicoli - E1C2_Fe_starve
minicoli - E1C2_LB
minicoli - E1C2_N_starve
minicoli - E1C2_cyt
minicoli - E1C2_glc
minicoli - E1C2_glyc
minicoli - E1C2_osm
minicoli - E1C2_ph5
minicoli - E1C2_pyr
minicoli - E1C2_sorb
minicoli - E1C2_urid
minicoli - F1C2-pyr
minicoli - F1C2_Fe_starve
minicoli - F1C2_LB
minicoli - F1C2_N_starve
minicoli - F1C2_cyt
minicoli - F1C2_fruc
minicoli - F1C2_glc
minicoli - F1C2_glyc
minicoli - F1C2_osm
minicoli - F1C2_ph5
minicoli - F1C2_sorb
minicoli - F1C2_urid
oxidative - deloxyr_pq
oxidative - delsoxr_pq
oxidative - delsoxs_pq
oxidative - wt_pq
RpoE Gene Weight
Gene Start
1e6
degP
yfeK
ygiM
rseA
rpoE
rseB
yeaY
cutC
yfeS
rseC
yhjJ
yfeY
ybfG
Cell wall/membrane/envelope biogenesis
Function unknown
Inorganic ion transport and metabolism
Post-translational modification, protein turnover,and chaperones
Signal transduction mechanisms
Transcription

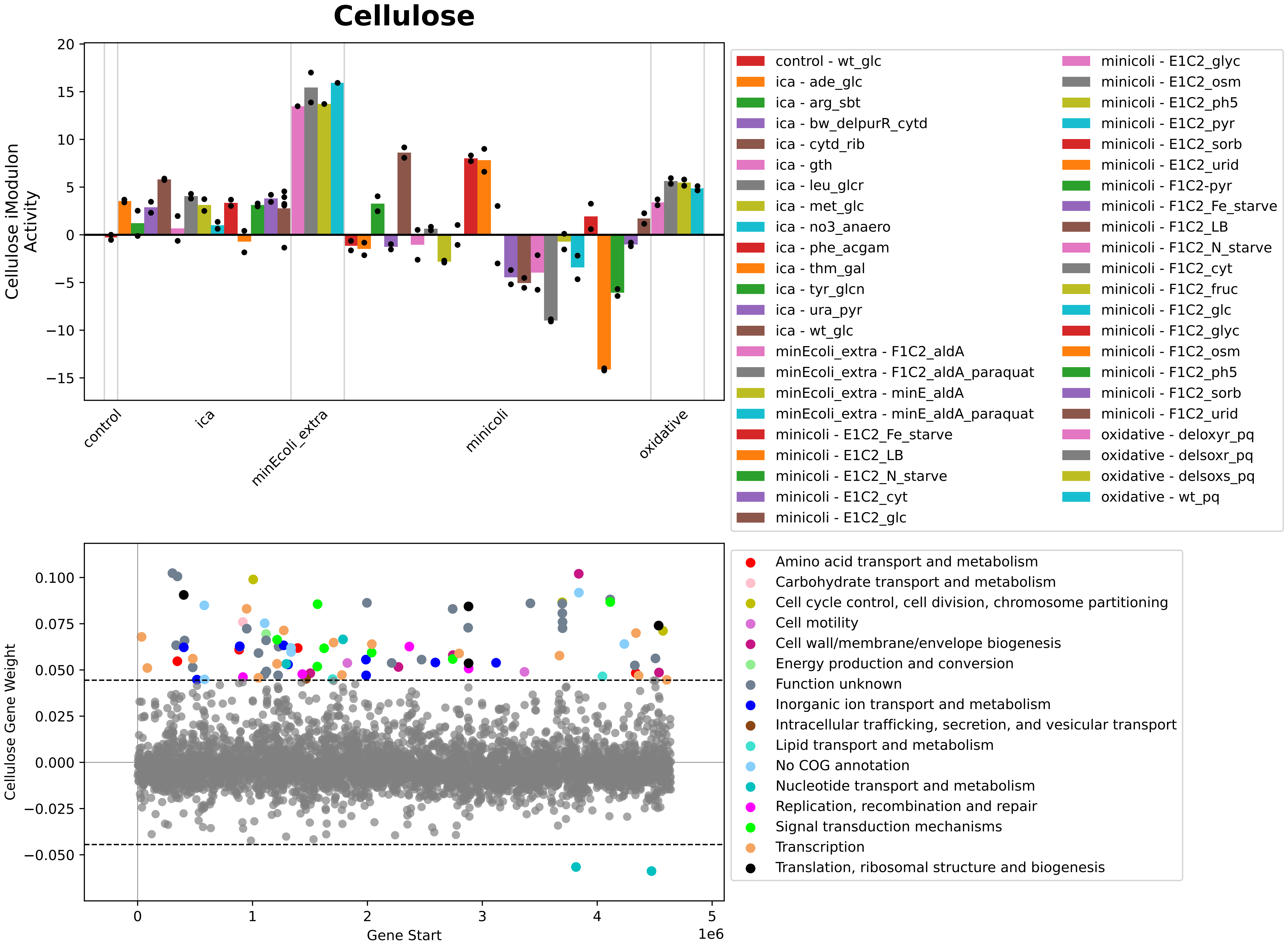
Cellulose
Cellulose iModulon Activity
control
ica
minEcoli_extra
minicoli
oxidative
control - wt_glc
ica - ade_glc
ica - arg_sbt
ica - bw_delpurR_cytd
ica - cytd_rib
ica - gth
ica - leu_glcr
ica - met_glc
ica - no3_anaero
ica - phe_acgam
ica - thm_gal
ica - tyr_glcn
ica - ura_pyr
ica - wt_glc
minEcoli_extra - F1C2_aldA
minEcoli_extra - F1C2_aldA_paraquat
minEcoli_extra - minE_aldA
minEcoli_extra - minE_aldA_paraquat
minicoli - E1C2_Fe_starve
minicoli - E1C2_LB
minicoli - E1C2_N_starve
minicoli - E1C2_cyt
minicoli - E1C2_glc
minicoli - E1C2_glyc
minicoli - E1C2_osm
minicoli - E1C2_ph5
minicoli - E1C2_pyr
minicoli - E1C2_sorb
minicoli - E1C2_urid
minicoli - F1C2-pyr
minicoli - F1C2_Fe_starve
minicoli - F1C2_LB
minicoli - F1C2_N_starve
minicoli - F1C2_cyt
minicoli - F1C2_fruc
minicoli - F1C2_glc
minicoli - F1C2_glyc
minicoli - F1C2_osm
minicoli - F1C2_ph5
minicoli - F1C2_sorb
minicoli - F1C2_urid
oxidative - deloxyr_pq
oxidative - delsoxr_pq
oxidative - delsoxs_pq
oxidative - wt_pq
Cellulose Gene Weight
Gene Start
1e6
Amino acid transport and metabolism
Carbohydrate transport and metabolism
Cell cycle control, cell division, chromosome partitioning
Cell motility
Cell wall/membrane/envelope biogenesis
Energy production and conversion
Function unknown
Inorganic ion transport and metabolism
Intracellular trafficking, secretion, and vesicular transport
Lipid transport and metabolism
No COG annotation
Nucleotide transport and metabolism
Replication, recombination and repair
Signal transduction mechanisms
Transcription
Translation, ribosomal structure and biogenesis

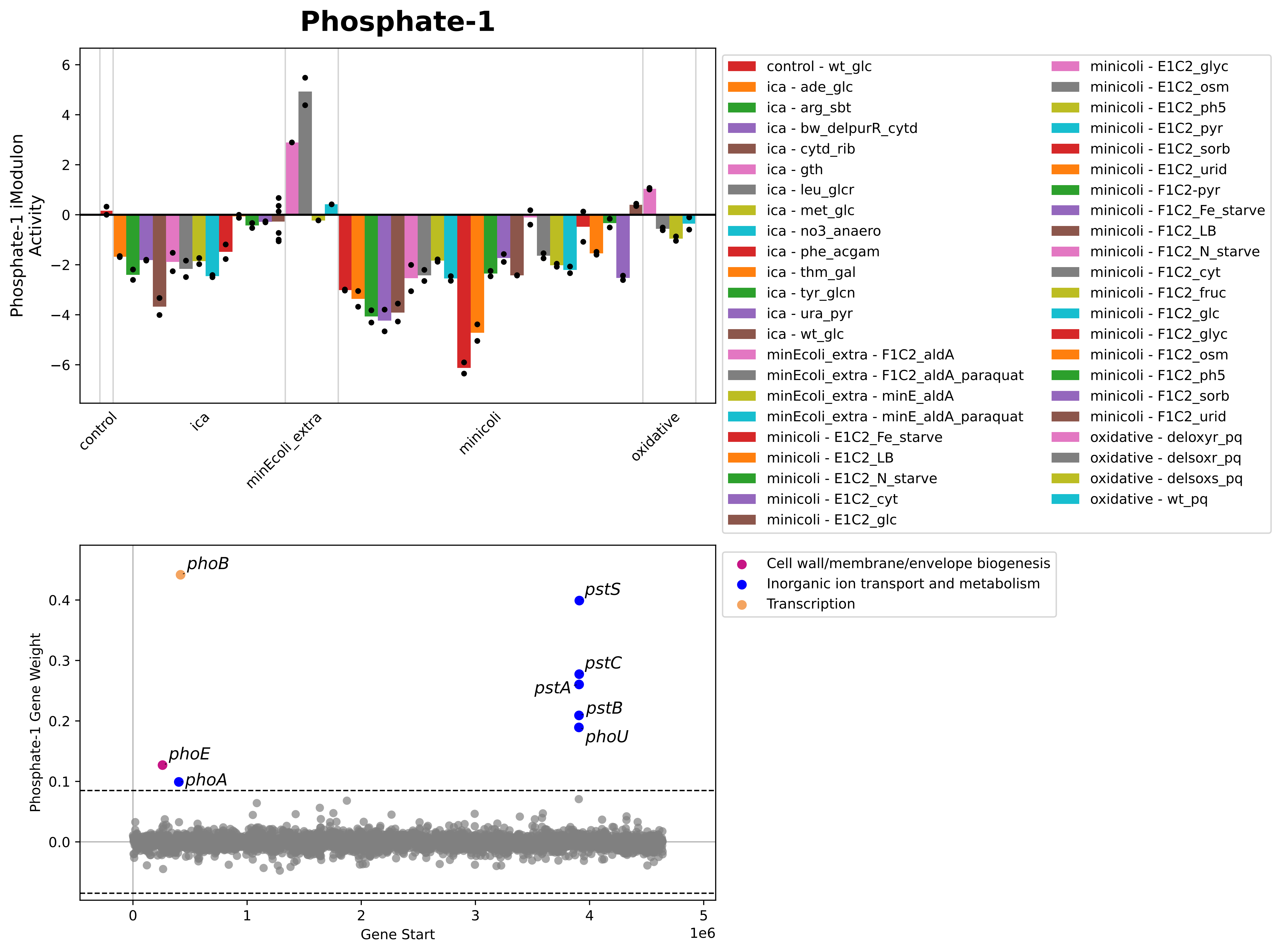

Phosphate-1
Phosphate-1 iModulon Activity
control
ica
minEcoli_extra
minicoli
oxidative
control - wt_glc
ica - ade_glc
ica - arg_sbt
ica - bw_delpurR_cytd
ica - cytd_rib
ica - gth
ica - leu_glcr
ica - met_glc
ica - no3_anaero
ica - phe_acgam
ica - thm_gal
ica - tyr_glcn
ica - ura_pyr
ica - wt_glc
minEcoli_extra - F1C2_aldA
minEcoli_extra - F1C2_aldA_paraquat
minEcoli_extra - minE_aldA
minEcoli_extra - minE_aldA_paraquat
minicoli - E1C2_Fe_starve
minicoli - E1C2_LB
minicoli - E1C2_N_starve
minicoli - E1C2_cyt
minicoli - E1C2_glc
minicoli - E1C2_glyc
minicoli - E1C2_osm
minicoli - E1C2_ph5
minicoli - E1C2_pyr
minicoli - E1C2_sorb
minicoli - E1C2_urid
minicoli - F1C2-pyr
minicoli - F1C2_Fe_starve
minicoli - F1C2_LB
minicoli - F1C2_N_starve
minicoli - F1C2_cyt
minicoli - F1C2_fruc
minicoli - F1C2_glc
minicoli - F1C2_glyc
minicoli - F1C2_osm
minicoli - F1C2_ph5
minicoli - F1C2_sorb
minicoli - F1C2_urid
oxidative - deloxyr_pq
oxidative - delsoxr_pq
oxidative - delsoxs_pq
oxidative - wt_pq
Phosphate-1 Gene Weight
Gene Start
1e6
phoB
pstS
pstC
pstA
pstB
phoU
phoE
phoA
Cell wall/membrane/envelope biogenesis
Inorganic ion transport and metabolism
Transcription

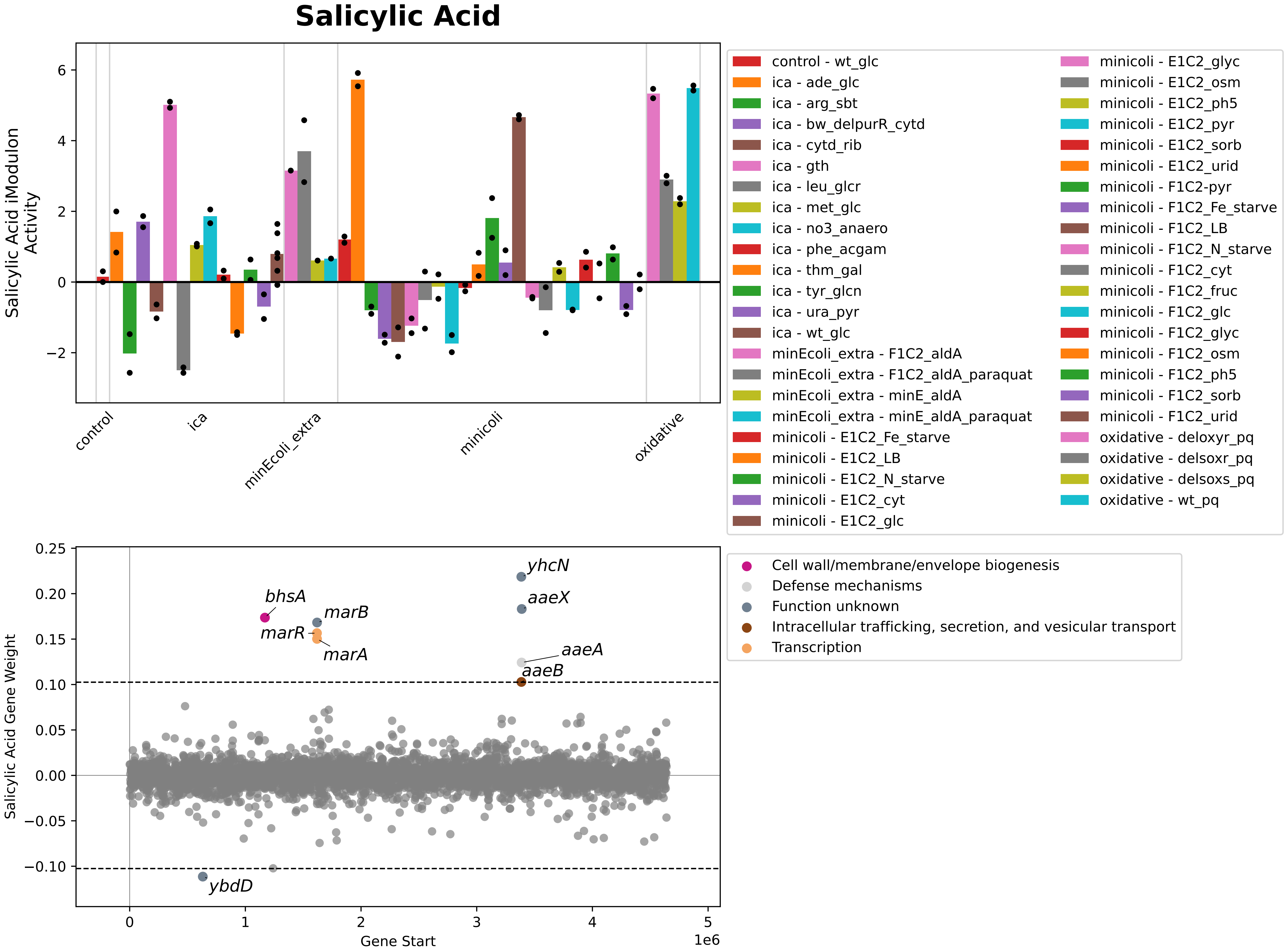

Salicylic Acid
Salicylic Acid iModulon Activity
control
ica
minEcoli_extra
minicoli
oxidative
control - wt_glc
ica - ade_glc
ica - arg_sbt
ica - bw_delpurR_cytd
ica - cytd_rib
ica - gth
ica - leu_glcr
ica - met_glc
ica - no3_anaero
ica - phe_acgam
ica - thm_gal
ica - tyr_glcn
ica - ura_pyr
ica - wt_glc
minEcoli_extra - F1C2_aldA
minEcoli_extra - F1C2_aldA_paraquat
minEcoli_extra - minE_aldA
minEcoli_extra - minE_aldA_paraquat
minicoli - E1C2_Fe_starve
minicoli - E1C2_LB
minicoli - E1C2_N_starve
minicoli - E1C2_cyt
minicoli - E1C2_glc
minicoli - E1C2_glyc
minicoli - E1C2_osm
minicoli - E1C2_ph5
minicoli - E1C2_pyr
minicoli - E1C2_sorb
minicoli - E1C2_urid
minicoli - F1C2-pyr
minicoli - F1C2_Fe_starve
minicoli - F1C2_LB
minicoli - F1C2_N_starve
minicoli - F1C2_cyt
minicoli - F1C2_fruc
minicoli - F1C2_glc
minicoli - F1C2_glyc
minicoli - F1C2_osm
minicoli - F1C2_ph5
minicoli - F1C2_sorb
minicoli - F1C2_urid
oxidative - deloxyr_pq
oxidative - delsoxr_pq
oxidative - delsoxs_pq
oxidative - wt_pq
Salicylic Acid Gene Weight
Gene Start
1e6
bhsA
marB
marR
marA
yhcN
aaeX
aaeA
aaeB
ybdD
Cell wall/membrane/envelope biogenesis
Defense mechanisms
Function unknown
Intracellular trafficking, secretion, and vesicular transport
Transcription

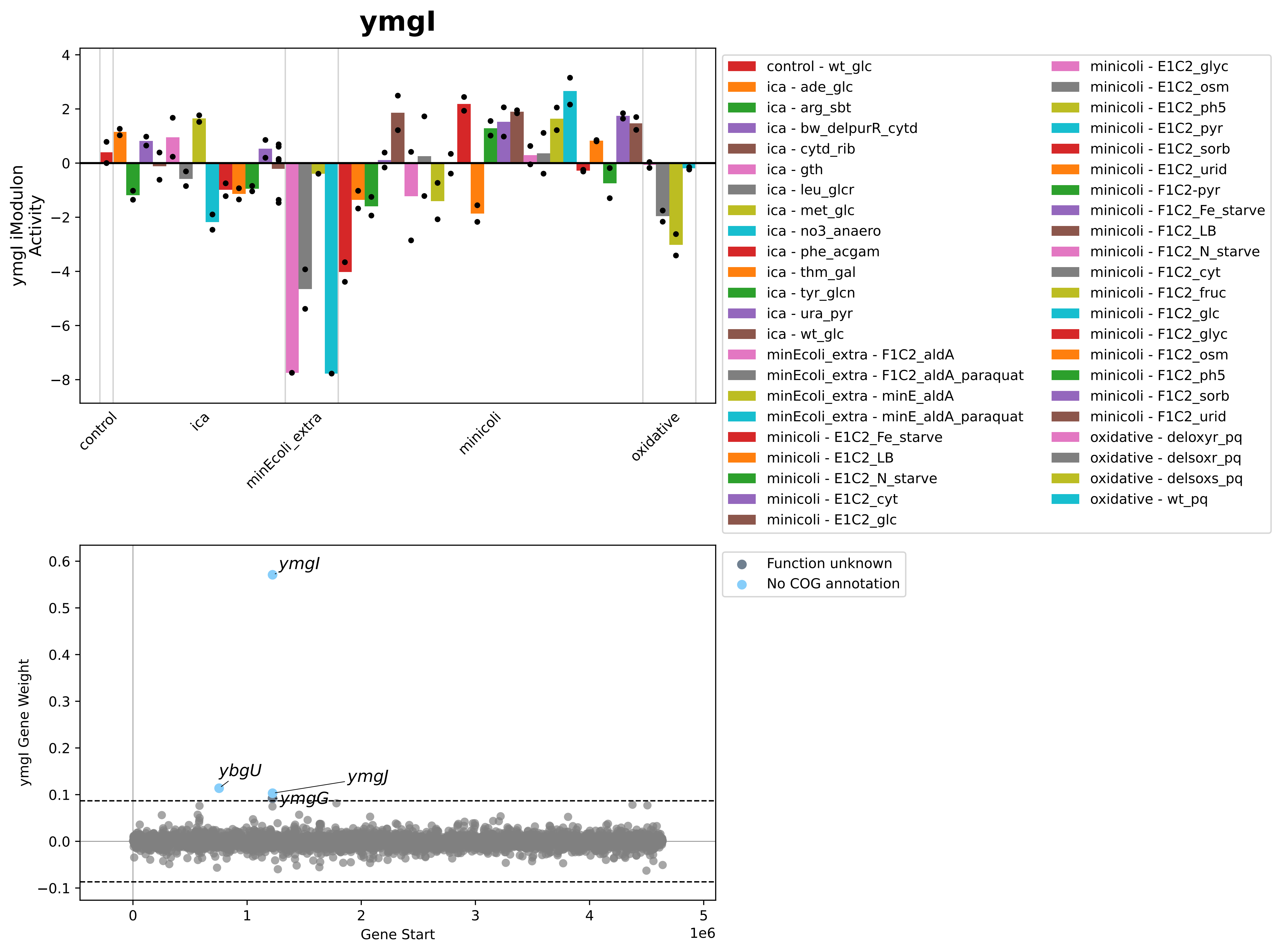
ymgI
ymgI iModulon Activity
control
ica
minEcoli_extra
minicoli
oxidative
control - wt_glc
ica - ade_glc
ica - arg_sbt
ica - bw_delpurR_cytd
ica - cytd_rib
ica - gth
ica - leu_glcr
ica - met_glc
ica - no3_anaero
ica - phe_acgam
ica - thm_gal
ica - tyr_glcn
ica - ura_pyr
ica - wt_glc
minEcoli_extra - F1C2_aldA
minEcoli_extra - F1C2_aldA_paraquat
minEcoli_extra - minE_aldA
minEcoli_extra - minE_aldA_paraquat
minicoli - E1C2_Fe_starve
minicoli - E1C2_LB
minicoli - E1C2_N_starve
minicoli - E1C2_cyt
minicoli - E1C2_glc
minicoli - E1C2_glyc
minicoli - E1C2_osm
minicoli - E1C2_ph5
minicoli - E1C2_pyr
minicoli - E1C2_sorb
minicoli - E1C2_urid
minicoli - F1C2-pyr
minicoli - F1C2_Fe_starve
minicoli - F1C2_LB
minicoli - F1C2_N_starve
minicoli - F1C2_cyt
minicoli - F1C2_fruc
minicoli - F1C2_glc
minicoli - F1C2_glyc
minicoli - F1C2_osm
minicoli - F1C2_ph5
minicoli - F1C2_sorb
minicoli - F1C2_urid
oxidative - deloxyr_pq
oxidative - delsoxr_pq
oxidative - delsoxs_pq
oxidative - wt_pq
ymgI Gene Weight
Gene Start
1e6
ymgI
ybgU
ymgJ
ymgG
Function unknown
No COG annotation

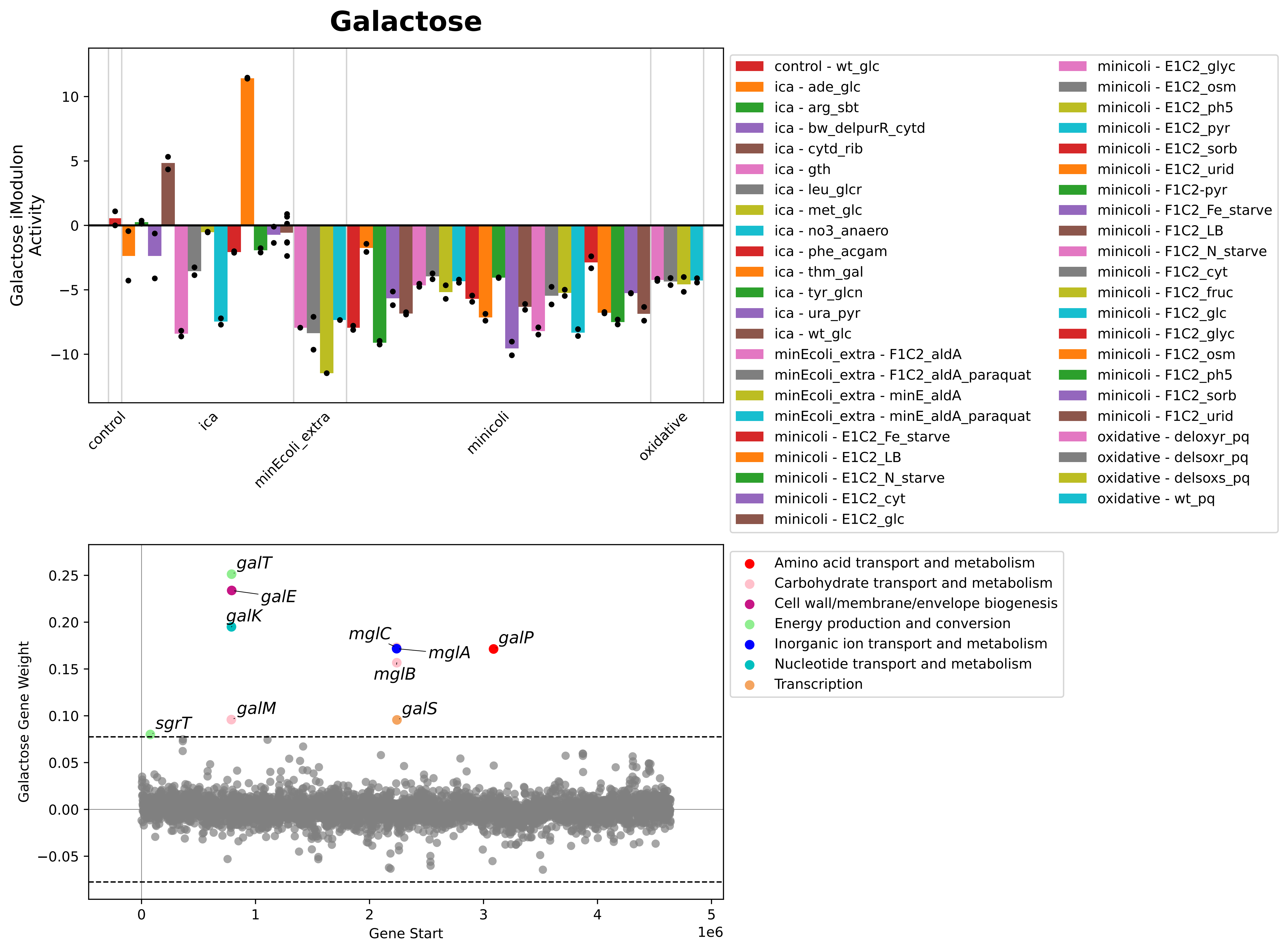

Galactose
Galactose iModulon Activity
control
ica
minEcoli_extra
minicoli
oxidative
control - wt_glc
ica - ade_glc
ica - arg_sbt
ica - bw_delpurR_cytd
ica - cytd_rib
ica - gth
ica - leu_glcr
ica - met_glc
ica - no3_anaero
ica - phe_acgam
ica - thm_gal
ica - tyr_glcn
ica - ura_pyr
ica - wt_glc
minEcoli_extra - F1C2_aldA
minEcoli_extra - F1C2_aldA_paraquat
minEcoli_extra - minE_aldA
minEcoli_extra - minE_aldA_paraquat
minicoli - E1C2_Fe_starve
minicoli - E1C2_LB
minicoli - E1C2_N_starve
minicoli - E1C2_cyt
minicoli - E1C2_glc
minicoli - E1C2_glyc
minicoli - E1C2_osm
minicoli - E1C2_ph5
minicoli - E1C2_pyr
minicoli - E1C2_sorb
minicoli - E1C2_urid
minicoli - F1C2-pyr
minicoli - F1C2_Fe_starve
minicoli - F1C2_LB
minicoli - F1C2_N_starve
minicoli - F1C2_cyt
minicoli - F1C2_fruc
minicoli - F1C2_glc
minicoli - F1C2_glyc
minicoli - F1C2_osm
minicoli - F1C2_ph5
minicoli - F1C2_sorb
minicoli - F1C2_urid
oxidative - deloxyr_pq
oxidative - delsoxr_pq
oxidative - delsoxs_pq
oxidative - wt_pq
Galactose Gene Weight
Gene Start
1e6
galT
galE
galK
mglC
mglA
mglB
galP
galM
galS
sgrT
Amino acid transport and metabolism
Carbohydrate transport and metabolism
Cell wall/membrane/envelope biogenesis
Energy production and conversion
Inorganic ion transport and metabolism
Nucleotide transport and metabolism
Transcription

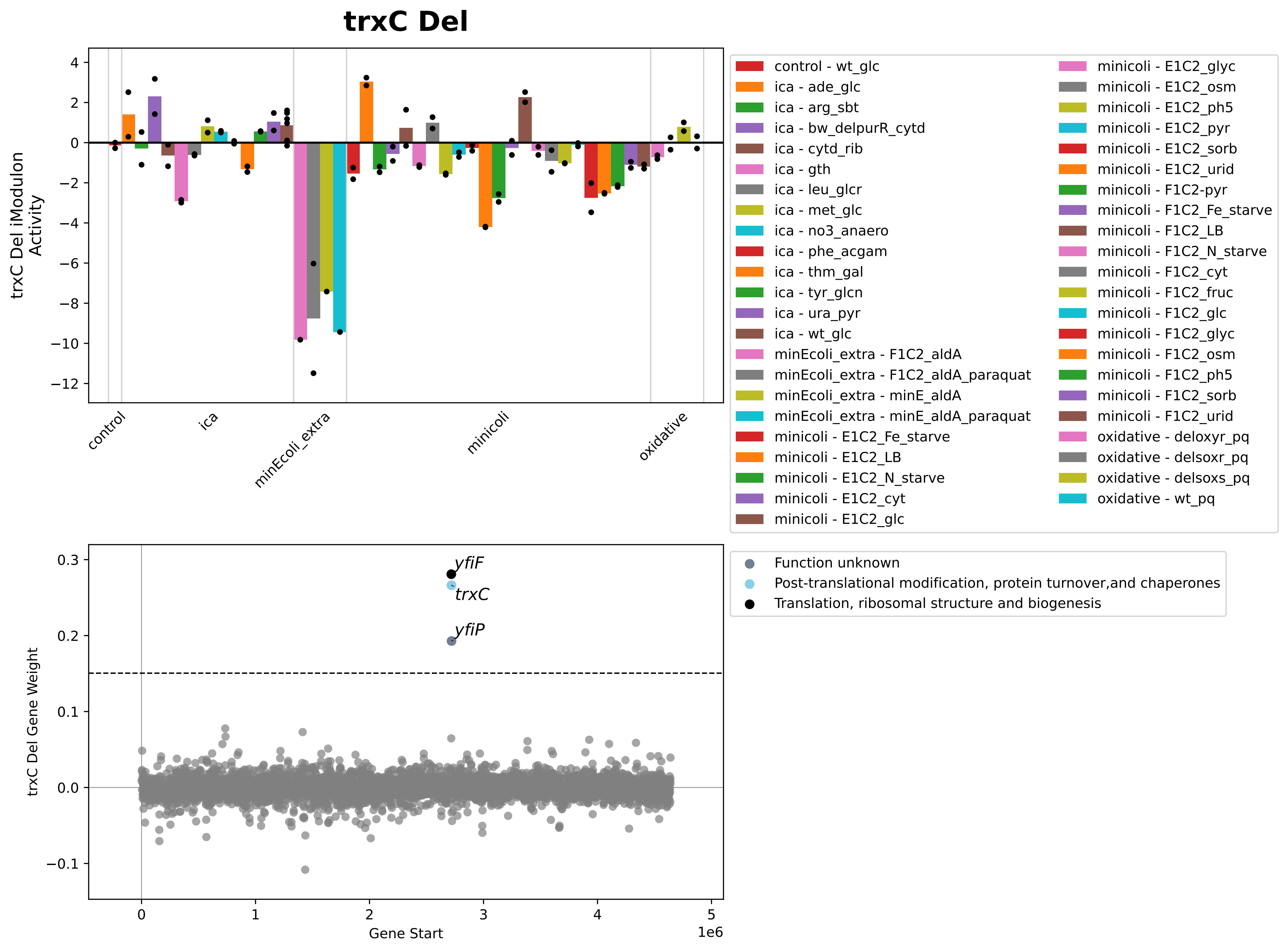

trxC Del
trxC Del iModulon Activity
control
ica
minEcoli_extra
minicoli
oxidative
control - wt_glc
ica - ade_glc
ica - arg_sbt
ica - bw_delpurR_cytd
ica - cytd_rib
ica - gth
ica - leu_glcr
ica - met_glc
ica - no3_anaero
ica - phe_acgam
ica - thm_gal
ica - tyr_glcn
ica - ura_pyr
ica - wt_glc
minEcoli_extra - F1C2_aldA
minEcoli_extra - F1C2_aldA_paraquat
minEcoli_extra - minE_aldA
minEcoli_extra - minE_aldA_paraquat
minicoli - E1C2_Fe_starve
minicoli - E1C2_LB
minicoli - E1C2_N_starve
minicoli - E1C2_cyt
minicoli - E1C2_glc
minicoli - E1C2_glyc
minicoli - E1C2_osm
minicoli - E1C2_ph5
minicoli - E1C2_pyr
minicoli - E1C2_sorb
minicoli - E1C2_urid
minicoli - F1C2-pyr
minicoli - F1C2_Fe_starve
minicoli - F1C2_LB
minicoli - F1C2_N_starve
minicoli - F1C2_cyt
minicoli - F1C2_fruc
minicoli - F1C2_glc
minicoli - F1C2_glyc
minicoli - F1C2_osm
minicoli - F1C2_ph5
minicoli - F1C2_sorb
minicoli - F1C2_urid
oxidative - deloxyr_pq
oxidative - delsoxr_pq
oxidative - delsoxs_pq
oxidative - wt_pq
yfiF
trxC
yfiP
trxC Del Gene Weight
Gene Start
1e6
Function unknown
Post-translational modification, protein turnover,and chaperones
Translation, ribosomal structure and biogenesis

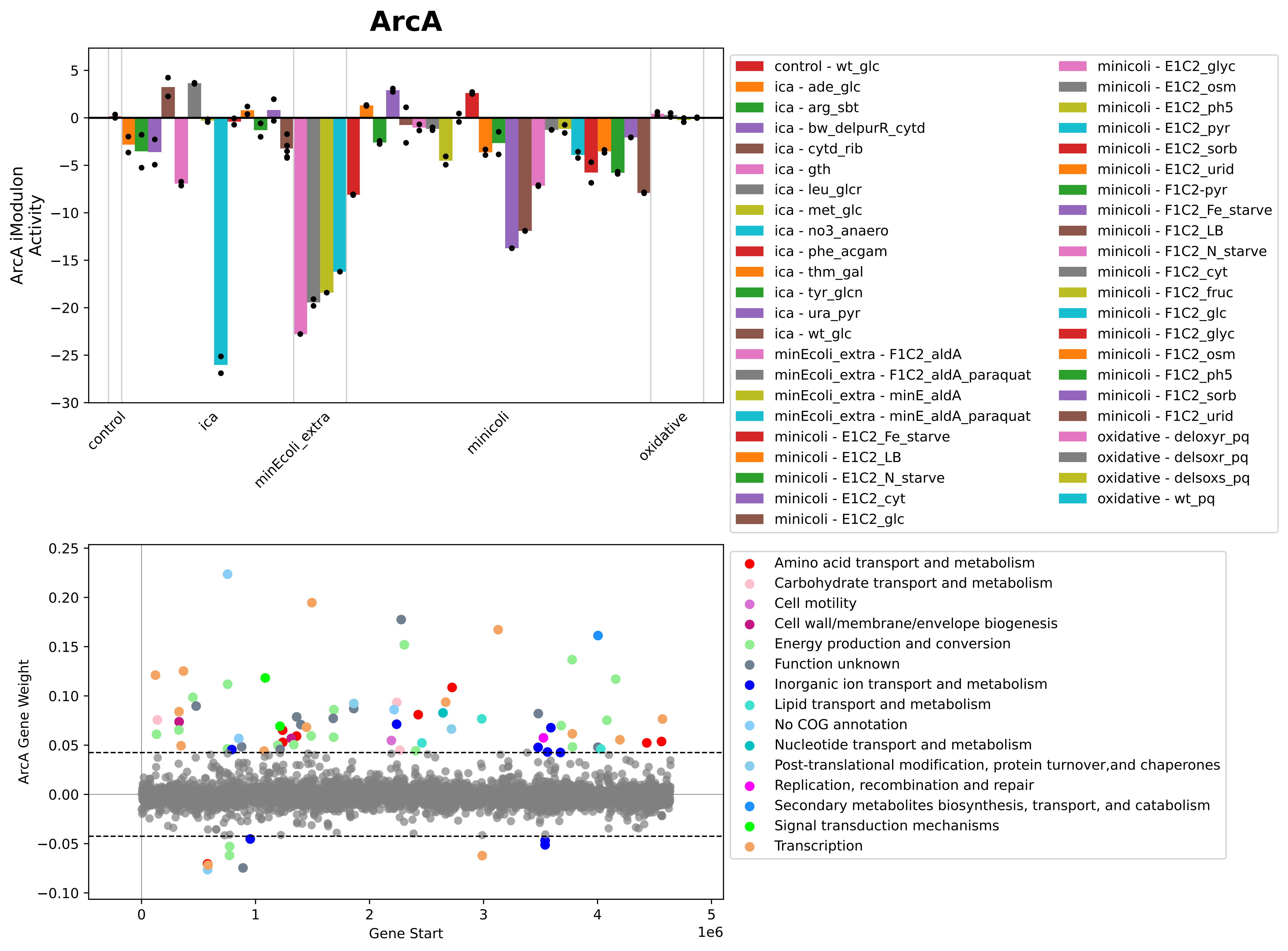

ArcA
ArcA iModulon Activity
control
ica
minEcoli_extra
minicoli
oxidative
control - wt_glc
ica - ade_glc
ica - arg_sbt
ica - bw_delpurR_cytd
ica - cytd_rib
ica - gth
ica - leu_glcr
ica - met_glc
ica - no3_anaero
ica - phe_acgam
ica - thm_gal
ica - tyr_glcn
ica - ura_pyr
ica - wt_glc
minEcoli_extra - F1C2_aldA
minEcoli_extra - F1C2_aldA_paraquat
minEcoli_extra - minE_aldA
minEcoli_extra - minE_aldA_paraquat
minicoli - E1C2_Fe_starve
minicoli - E1C2_LB
minicoli - E1C2_N_starve
minicoli - E1C2_cyt
minicoli - E1C2_glc
minicoli - E1C2_glyc
minicoli - E1C2_osm
minicoli - E1C2_ph5
minicoli - E1C2_pyr
minicoli - E1C2_sorb
minicoli - E1C2_urid
minicoli - F1C2-pyr
minicoli - F1C2_Fe_starve
minicoli - F1C2_LB
minicoli - F1C2_N_starve
minicoli - F1C2_cyt
minicoli - F1C2_fruc
minicoli - F1C2_glc
minicoli - F1C2_glyc
minicoli - F1C2_osm
minicoli - F1C2_ph5
minicoli - F1C2_sorb
minicoli - F1C2_urid
oxidative - deloxyr_pq
oxidative - delsoxr_pq
oxidative - delsoxs_pq
oxidative - wt_pq
ArcA Gene Weight
Gene Start
1e6
Amino acid transport and metabolism
Carbohydrate transport and metabolism
Cell motility
Cell wall/membrane/envelope biogenesis
Energy production and conversion
Function unknown
Inorganic ion transport and metabolism
Lipid transport and metabolism
No COG annotation
Nucleotide transport and metabolism
Post-translational modification, protein turnover,and chaperones
Replication, recombination and repair
Secondary metabolites biosynthesis, transport, and catabolism
Signal transduction mechanisms
Transcription

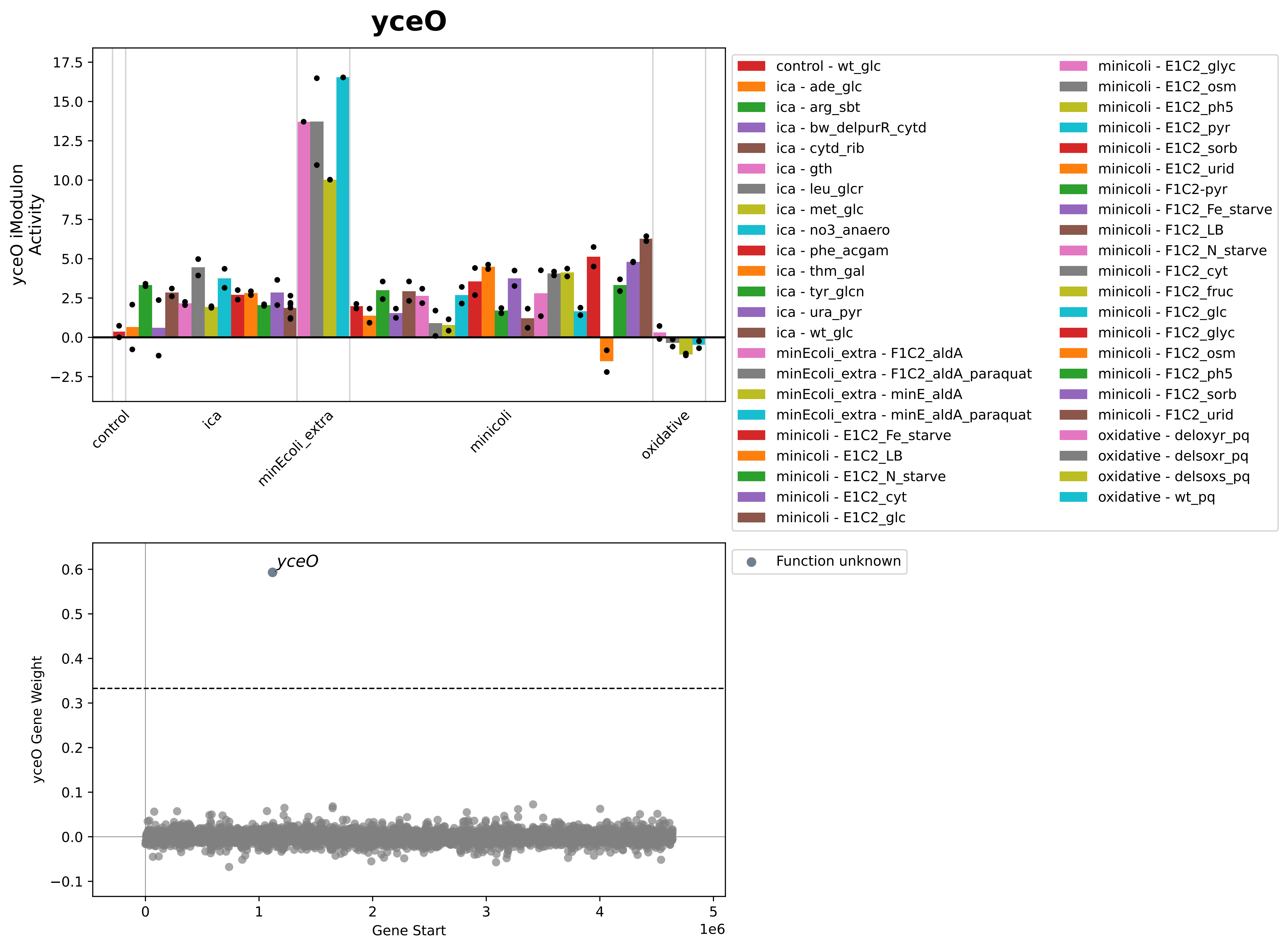

yceO
yceO iModulon Activity
control
ica
minEcoli_extra
minicoli
oxidative
control - wt_glc
ica - ade_glc
ica - arg_sbt
ica - bw_delpurR_cytd
ica - cytd_rib
ica - gth
ica - leu_glcr
ica - met_glc
ica - no3_anaero
ica - phe_acgam
ica - thm_gal
ica - tyr_glcn
ica - ura_pyr
ica - wt_glc
minEcoli_extra - F1C2_aldA
minEcoli_extra - F1C2_aldA_paraquat
minEcoli_extra - minE_aldA
minEcoli_extra - minE_aldA_paraquat
minicoli - E1C2_Fe_starve
minicoli - E1C2_LB
minicoli - E1C2_N_starve
minicoli - E1C2_cyt
minicoli - E1C2_glc
minicoli - E1C2_glyc
minicoli - E1C2_osm
minicoli - E1C2_ph5
minicoli - E1C2_pyr
minicoli - E1C2_sorb
minicoli - E1C2_urid
minicoli - F1C2-pyr
minicoli - F1C2_Fe_starve
minicoli - F1C2_LB
minicoli - F1C2_N_starve
minicoli - F1C2_cyt
minicoli - F1C2_fruc
minicoli - F1C2_glc
minicoli - F1C2_glyc
minicoli - F1C2_osm
minicoli - F1C2_ph5
minicoli - F1C2_sorb
minicoli - F1C2_urid
oxidative - deloxyr_pq
oxidative - delsoxr_pq
oxidative - delsoxs_pq
oxidative - wt_pq
yceO
yceO Gene Weight
Gene Start
1e6
Function unknown

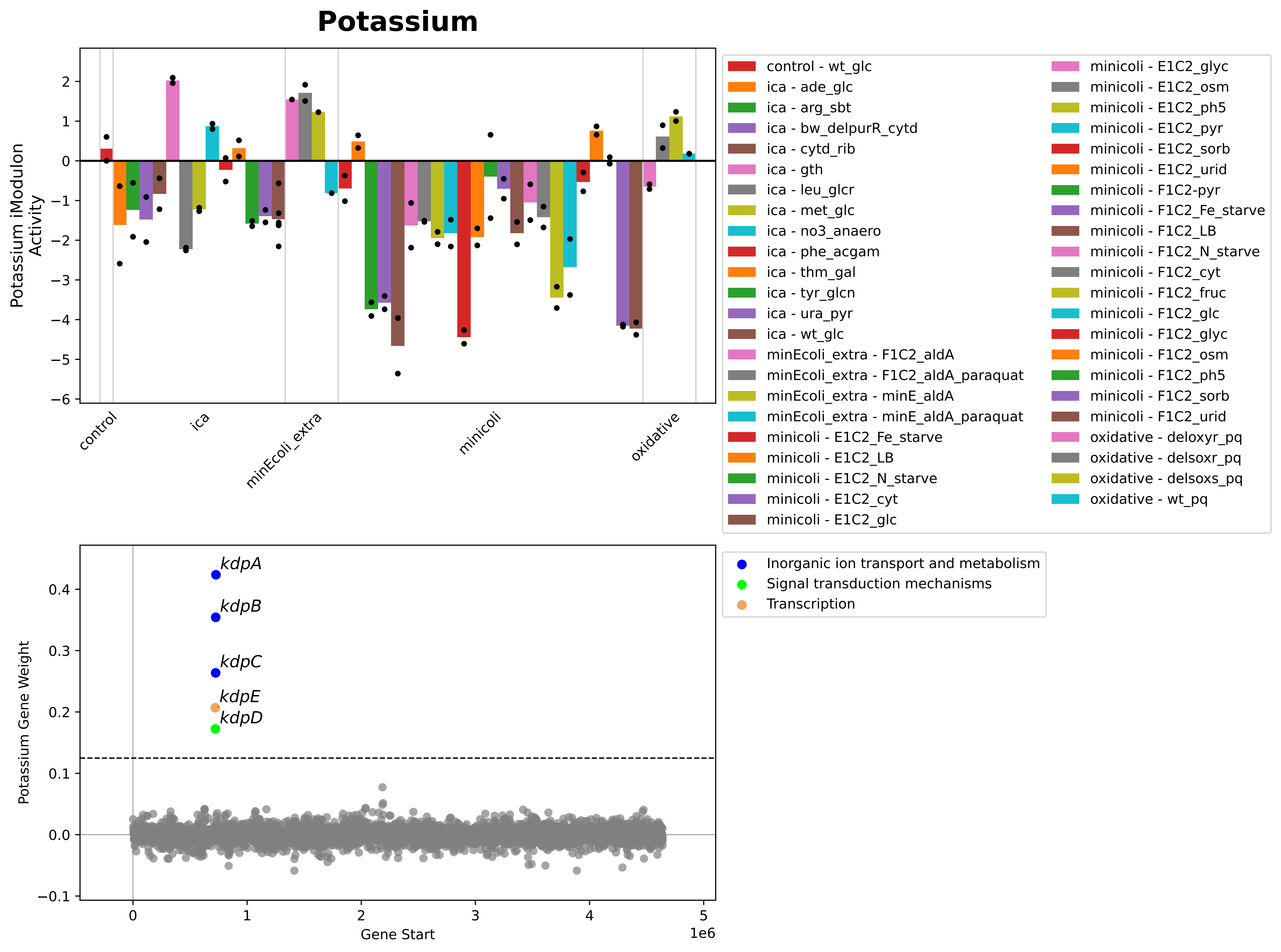

Potassium
Potassium iModulon Activity
control
ica
minEcoli_extra
minicoli
oxidative
control - wt_glc
ica - ade_glc
ica - arg_sbt
ica - bw_delpurR_cytd
ica - cytd_rib
ica - gth
ica - leu_glcr
ica - met_glc
ica - no3_anaero
ica - phe_acgam
ica - thm_gal
ica - tyr_glcn
ica - ura_pyr
ica - wt_glc
minEcoli_extra - F1C2_aldA
minEcoli_extra - F1C2_aldA_paraquat
minEcoli_extra - minE_aldA
minEcoli_extra - minE_aldA_paraquat
minicoli - E1C2_Fe_starve
minicoli - E1C2_LB
minicoli - E1C2_N_starve
minicoli - E1C2_cyt
minicoli - E1C2_glc
minicoli - E1C2_glyc
minicoli - E1C2_osm
minicoli - E1C2_ph5
minicoli - E1C2_pyr
minicoli - E1C2_sorb
minicoli - E1C2_urid
minicoli - F1C2-pyr
minicoli - F1C2_Fe_starve
minicoli - F1C2_LB
minicoli - F1C2_N_starve
minicoli - F1C2_cyt
minicoli - F1C2_fruc
minicoli - F1C2_glc
minicoli - F1C2_glyc
minicoli - F1C2_osm
minicoli - F1C2_ph5
minicoli - F1C2_sorb
minicoli - F1C2_urid
oxidative - deloxyr_pq
oxidative - delsoxr_pq
oxidative - delsoxs_pq
oxidative - wt_pq
kdpA
kdpB
kdpC
kdpE
kdpD
Potassium Gene Weight
Gene Start
1e6
Inorganic ion transport and metabolism
Signal transduction mechanisms
Transcription

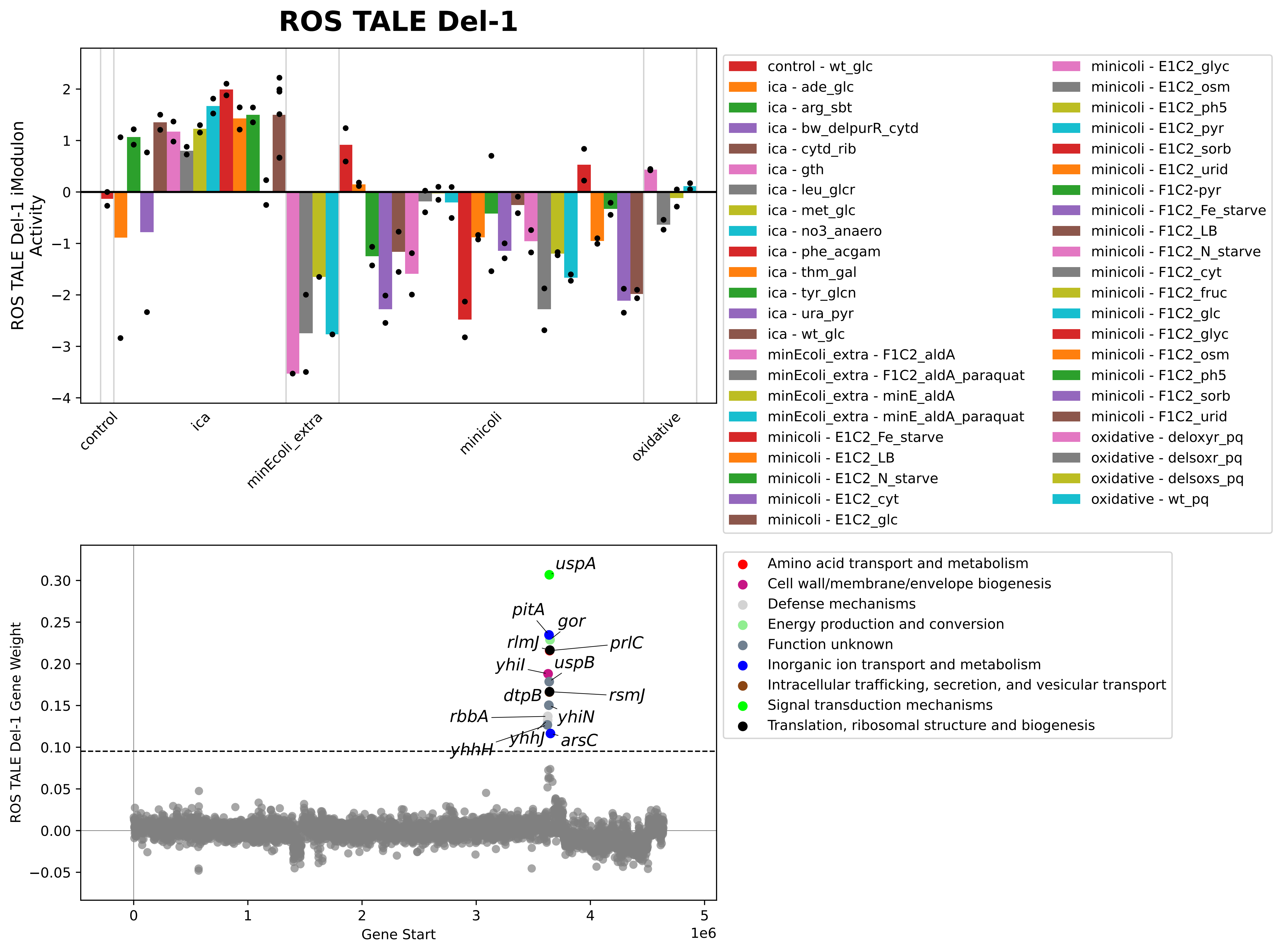
ROS TALE Del-1
ROS TALE Del-1 iModulon Activity
control
ica
minEcoli_extra
minicoli
oxidative
control - wt_glc
ica - ade_glc
ica - arg_sbt
ica - bw_delpurR_cytd
ica - cytd_rib
ica - gth
ica - leu_glcr
ica - met_glc
ica - no3_anaero
ica - phe_acgam
ica - thm_gal
ica - tyr_glcn
ica - ura_pyr
ica - wt_glc
minEcoli_extra - F1C2_aldA
minEcoli_extra - F1C2_aldA_paraquat
minEcoli_extra - minE_aldA
minEcoli_extra - minE_aldA_paraquat
minicoli - E1C2_Fe_starve
minicoli - E1C2_LB
minicoli - E1C2_N_starve
minicoli - E1C2_cyt
minicoli - E1C2_glc
minicoli - E1C2_glyc
minicoli - E1C2_osm
minicoli - E1C2_ph5
minicoli - E1C2_pyr
minicoli - E1C2_sorb
minicoli - E1C2_urid
minicoli - F1C2-pyr
minicoli - F1C2_Fe_starve
minicoli - F1C2_LB
minicoli - F1C2_N_starve
minicoli - F1C2_cyt
minicoli - F1C2_fruc
minicoli - F1C2_glc
minicoli - F1C2_glyc
minicoli - F1C2_osm
minicoli - F1C2_ph5
minicoli - F1C2_sorb
minicoli - F1C2_urid
oxidative - deloxyr_pq
oxidative - delsoxr_pq
oxidative - delsoxs_pq
oxidative - wt_pq
ROS TALE Del-1 Gene Weight
Gene Start
1e6
uspA
pitA
gor
rlmJ
prlC
yhiI
uspB
dtpB
rsmJ
rbbA
yhiN
yhhJ
arsC
yhhH
Amino acid transport and metabolism
Cell wall/membrane/envelope biogenesis
Defense mechanisms
Energy production and conversion
Function unknown
Inorganic ion transport and metabolism
Intracellular trafficking, secretion, and vesicular transport
Signal transduction mechanisms
Translation, ribosomal structure and biogenesis

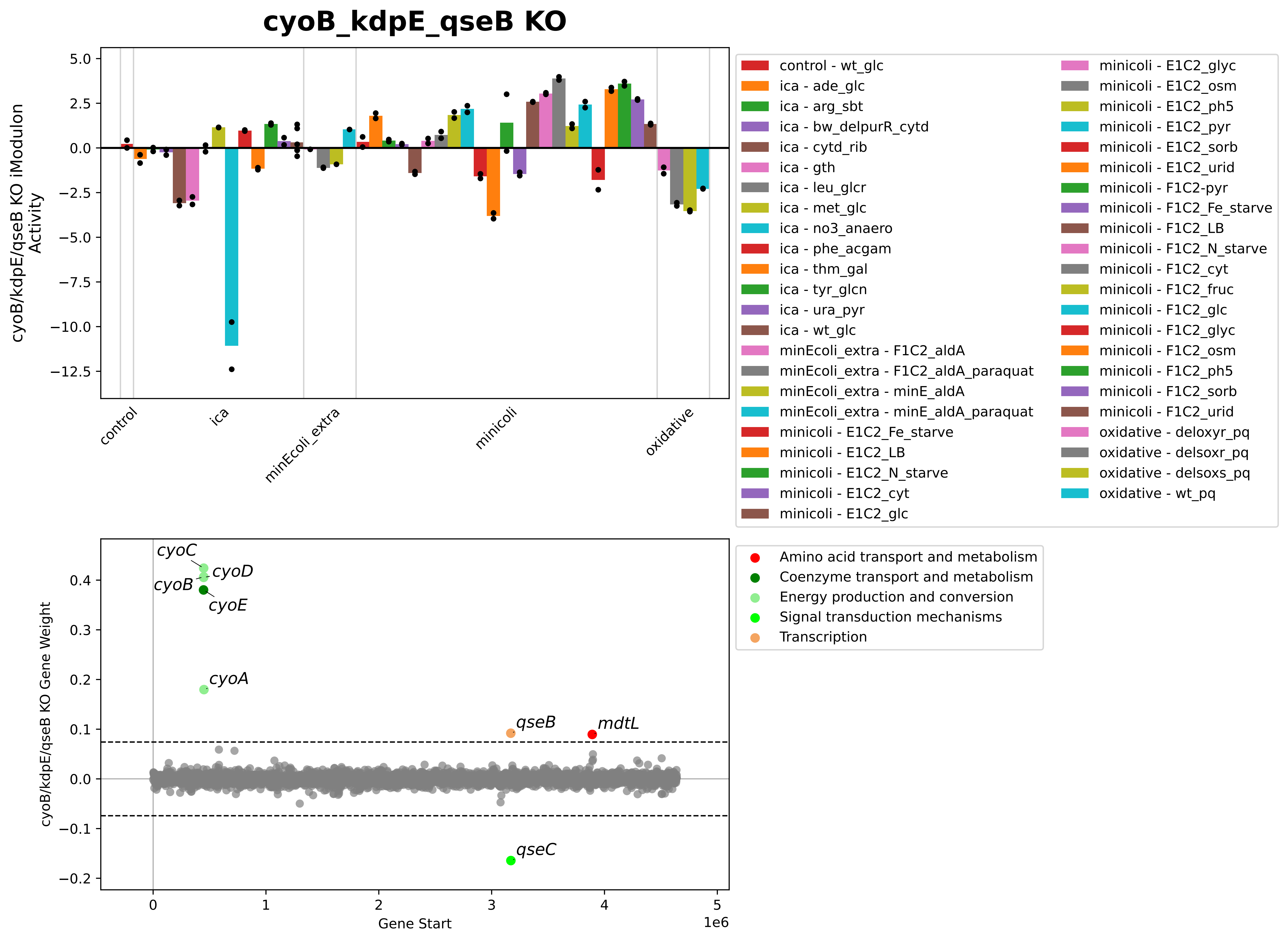

cyoB_kdpE_qseB KO
cyoB/kdpE/qseB KO iModulon Activity
5.0
2.5
0.0
−2.5
−5.0
−7.5
−10.0
−12.5
control
ica
minEcoli_extra
minicoli
oxidative
control - wt_glc
ica - ade_glc
ica - arg_sbt
ica - bw_delpurR_cytd
ica - cytd_rib
ica - gth
ica - leu_glcr
ica - met_glc
ica - no3_anaero
ica - phe_acgam
ica - thm_gal
ica - tyr_glcn
ica - ura_pyr
ica - wt_glc
minEcoli_extra - F1C2_aldA
minEcoli_extra - F1C2_aldA_paraquat
minEcoli_extra - minE_aldA
minEcoli_extra - minE_aldA_paraquat
minicoli - E1C2_Fe_starve
minicoli - E1C2_LB
minicoli - E1C2_N_starve
minicoli - E1C2_cyt
minicoli - E1C2_glc
minicoli - E1C2_glyc
minicoli - E1C2_osm
minicoli - E1C2_ph5
minicoli - E1C2_pyr
minicoli - E1C2_sorb
minicoli - E1C2_urid
minicoli - F1C2-pyr
minicoli - F1C2_Fe_starve
minicoli - F1C2_LB
minicoli - F1C2_N_starve
minicoli - F1C2_cyt
minicoli - F1C2_fruc
minicoli - F1C2_glc
minicoli - F1C2_glyc
minicoli - F1C2_osm
minicoli - F1C2_ph5
minicoli - F1C2_sorb
minicoli - F1C2_urid
oxidative - deloxyr_pq
oxidative - delsoxr_pq
oxidative - delsoxs_pq
oxidative - wt_pq
cyoB/kdpE/qseB KO Gene Weight
0.4
0.3
0.2
0.1
0.0
−0.1
−0.2
cyoC
cyoD
cyoB
cyoE
cyoA
qseB
mdtL
qseC
0
1
2
3
4
5
1e6
Gene Start
Amino acid transport and metabolism
Coenzyme transport and metabolism
Energy production and conversion
Signal transduction mechanisms
Transcription

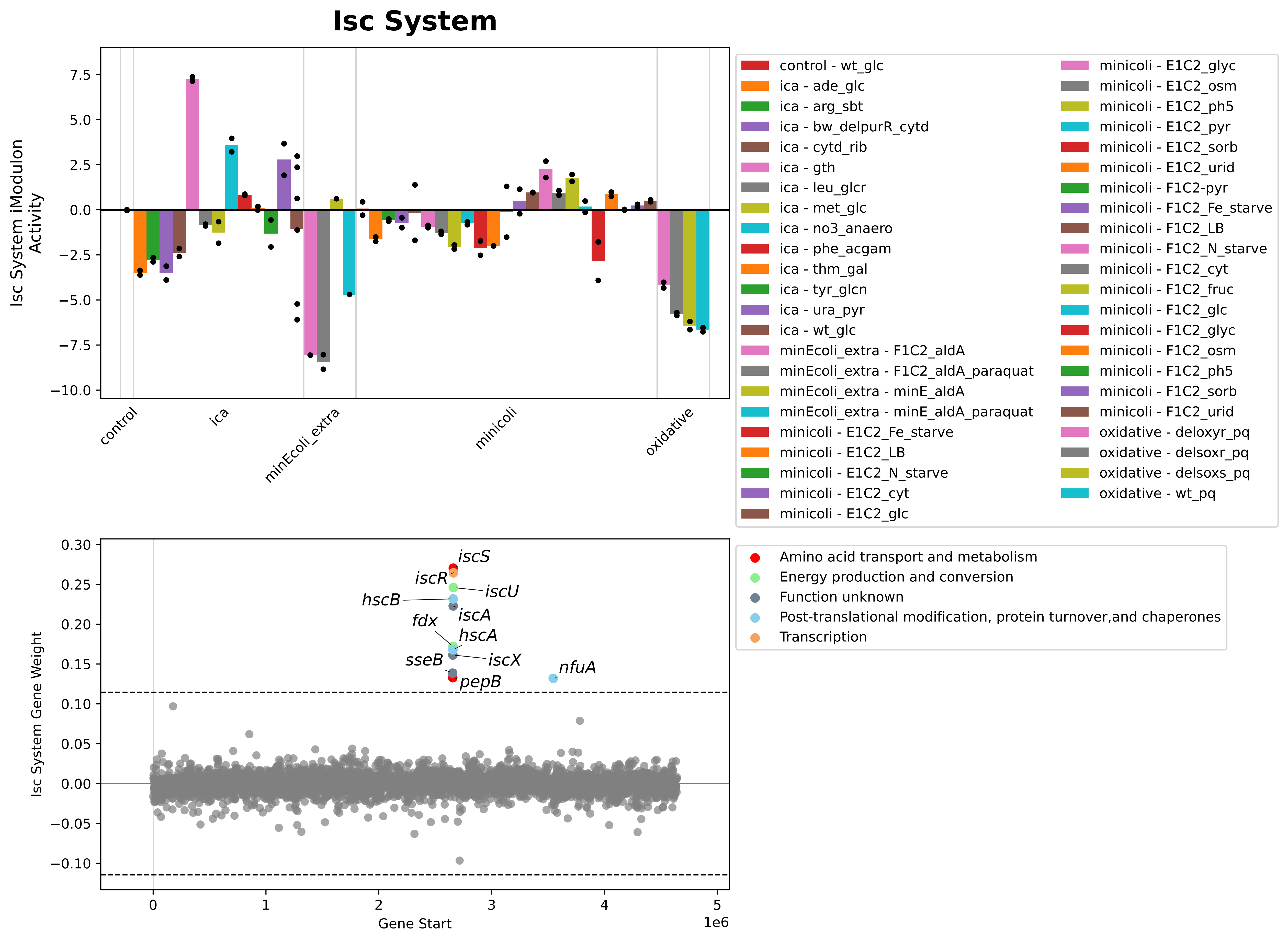
Isc System
Isc System iModulon Activity
control
ica
minEcoli_extra
minicoli
oxidative
control - wt_glc
ica - ade_glc
ica - arg_sbt
ica - bw_delpurR_cytd
ica - cytd_rib
ica - gth
ica - leu_glcr
ica - met_glc
ica - no3_anaero
ica - phe_acgam
ica - thm_gal
ica - tyr_glcn
ica - ura_pyr
ica - wt_glc
minEcoli_extra - F1C2_aldA
minEcoli_extra - F1C2_aldA_paraquat
minEcoli_extra - minE_aldA
minEcoli_extra - minE_aldA_paraquat
minicoli - E1C2_Fe_starve
minicoli - E1C2_LB
minicoli - E1C2_N_starve
minicoli - E1C2_cyt
minicoli - E1C2_glc
minicoli - E1C2_glyc
minicoli - E1C2_osm
minicoli - E1C2_ph5
minicoli - E1C2_pyr
minicoli - E1C2_sorb
minicoli - E1C2_urid
minicoli - F1C2-pyr
minicoli - F1C2_Fe_starve
minicoli - F1C2_LB
minicoli - F1C2_N_starve
minicoli - F1C2_cyt
minicoli - F1C2_fruc
minicoli - F1C2_glc
minicoli - F1C2_glyc
minicoli - F1C2_osm
minicoli - F1C2_ph5
minicoli - F1C2_sorb
minicoli - F1C2_urid
oxidative - deloxyr_pq
oxidative - delsoxr_pq
oxidative - delsoxs_pq
oxidative - wt_pq
iscS
iscR
iscU
hscB
iscA
fdx
hscA
sseB
iscX
nfuA
pepB
Isc System Gene Weight
Gene Start
1e6
Amino acid transport and metabolism
Energy production and conversion
Function unknown
Post-translational modification, protein turnover,and chaperones
Transcription

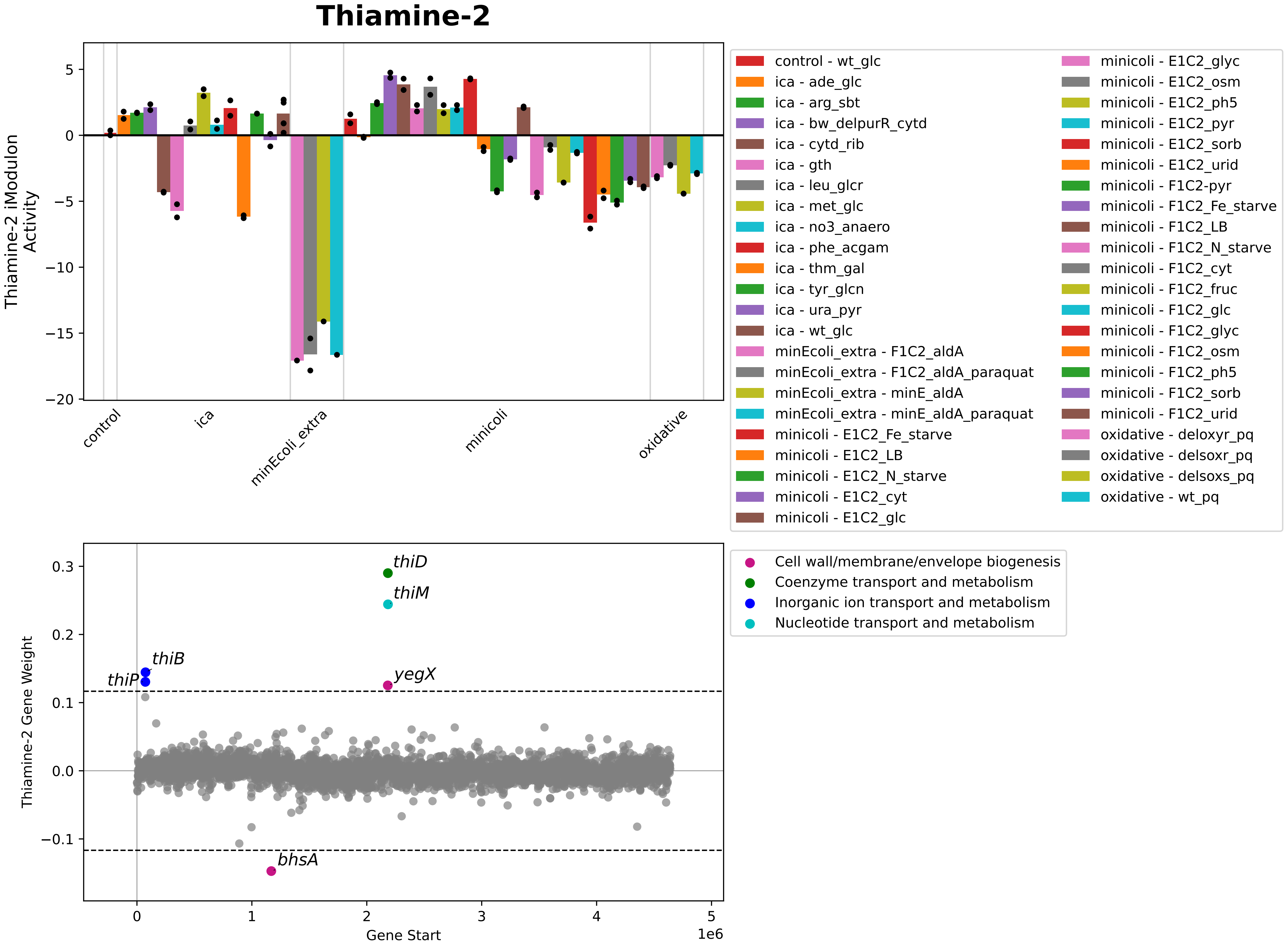

Thiamine-2
Thiamine-2 iModulon Activity
control
ica
minEcoli_extra
minicoli
oxidative
control - wt_glc
ica - ade_glc
ica - arg_sbt
ica - bw_delpurR_cytd
ica - cytd_rib
ica - gth
ica - leu_glcr
ica - met_glc
ica - no3_anaero
ica - phe_acgam
ica - thm_gal
ica - tyr_glcn
ica - ura_pyr
ica - wt_glc
minEcoli_extra - F1C2_aldA
minEcoli_extra - F1C2_aldA_paraquat
minEcoli_extra - minE_aldA
minEcoli_extra - minE_aldA_paraquat
minicoli - E1C2_Fe_starve
minicoli - E1C2_LB
minicoli - E1C2_N_starve
minicoli - E1C2_cyt
minicoli - E1C2_glc
minicoli - E1C2_glyc
minicoli - E1C2_osm
minicoli - E1C2_ph5
minicoli - E1C2_pyr
minicoli - E1C2_sorb
minicoli - E1C2_urid
minicoli - F1C2-pyr
minicoli - F1C2_Fe_starve
minicoli - F1C2_LB
minicoli - F1C2_N_starve
minicoli - F1C2_cyt
minicoli - F1C2_fruc
minicoli - F1C2_glc
minicoli - F1C2_glyc
minicoli - F1C2_osm
minicoli - F1C2_ph5
minicoli - F1C2_sorb
minicoli - F1C2_urid
oxidative - deloxyr_pq
oxidative - delsoxr_pq
oxidative - delsoxs_pq
oxidative - wt_pq
Thiamine-2 Gene Weight
Gene Start
1e6
thiD
thiM
thiB
thiP
yegX
bhsA
Cell wall/membrane/envelope biogenesis
Coenzyme transport and metabolism
Inorganic ion transport and metabolism
Nucleotide transport and metabolism

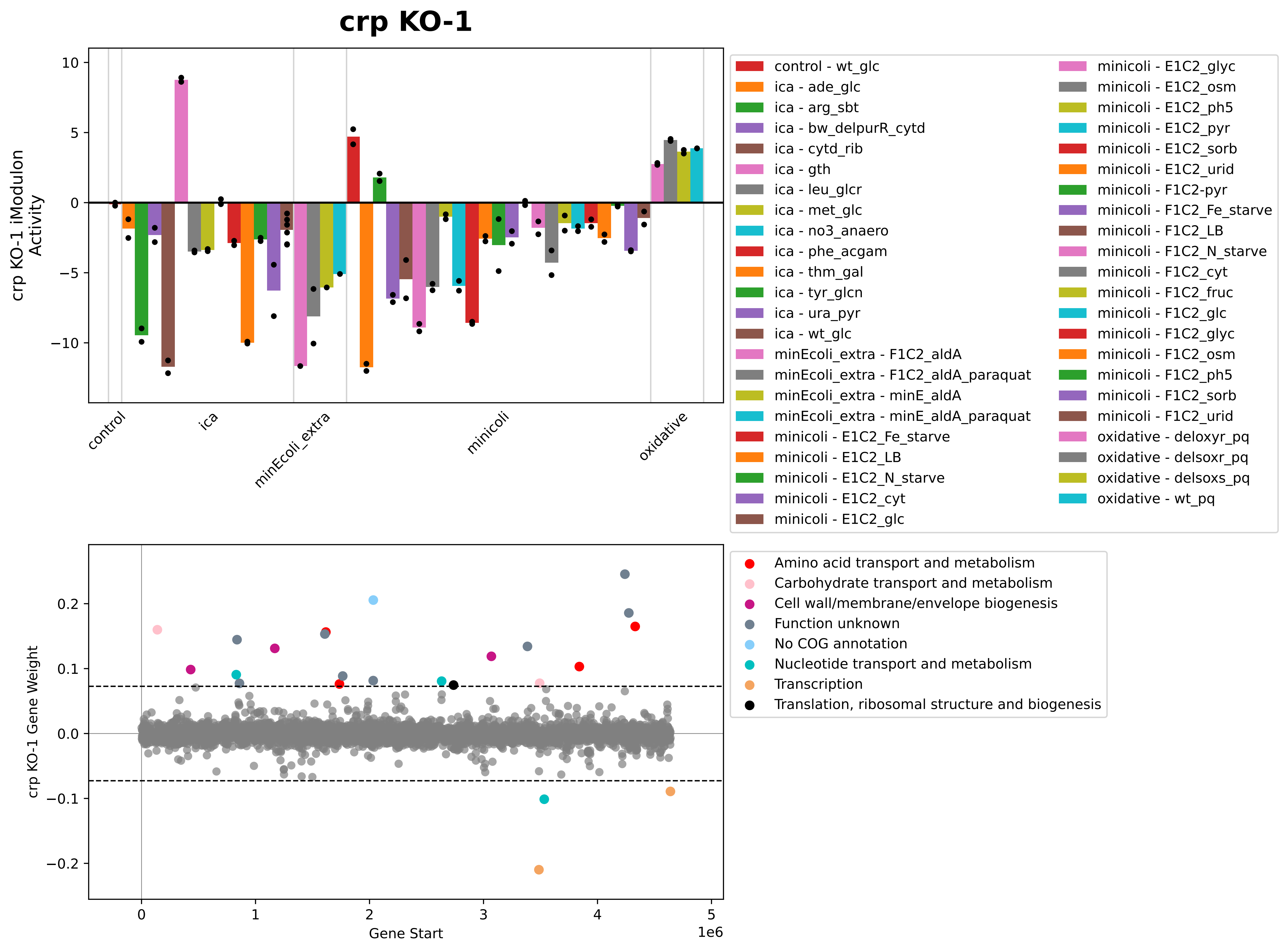
crp KO-1
crp KO-1 iModulon Activity
control
ica
minEcoli_extra
minicoli
oxidative
control - wt_glc
ica - ade_glc
ica - arg_sbt
ica - bw_delpurR_cytd
ica - cytd_rib
ica - gth
ica - leu_glcr
ica - met_glc
ica - no3_anaero
ica - phe_acgam
ica - thm_gal
ica - tyr_glcn
ica - ura_pyr
ica - wt_glc
minEcoli_extra - F1C2_aldA
minEcoli_extra - F1C2_aldA_paraquat
minEcoli_extra - minE_aldA
minEcoli_extra - minE_aldA_paraquat
minicoli - E1C2_Fe_starve
minicoli - E1C2_LB
minicoli - E1C2_N_starve
minicoli - E1C2_cyt
minicoli - E1C2_glc
minicoli - E1C2_glyc
minicoli - E1C2_osm
minicoli - E1C2_ph5
minicoli - E1C2_pyr
minicoli - E1C2_sorb
minicoli - E1C2_urid
minicoli - F1C2-pyr
minicoli - F1C2_Fe_starve
minicoli - F1C2_LB
minicoli - F1C2_N_starve
minicoli - F1C2_cyt
minicoli - F1C2_fruc
minicoli - F1C2_glc
minicoli - F1C2_glyc
minicoli - F1C2_osm
minicoli - F1C2_ph5
minicoli - F1C2_sorb
minicoli - F1C2_urid
oxidative - deloxyr_pq
oxidative - delsoxr_pq
oxidative - delsoxs_pq
oxidative - wt_pq
crp KO-1 Gene Weight
Gene Start
1e6
Amino acid transport and metabolism
Carbohydrate transport and metabolism
Cell wall/membrane/envelope biogenesis
Function unknown
No COG annotation
Nucleotide transport and metabolism
Transcription
Translation, ribosomal structure and biogenesis

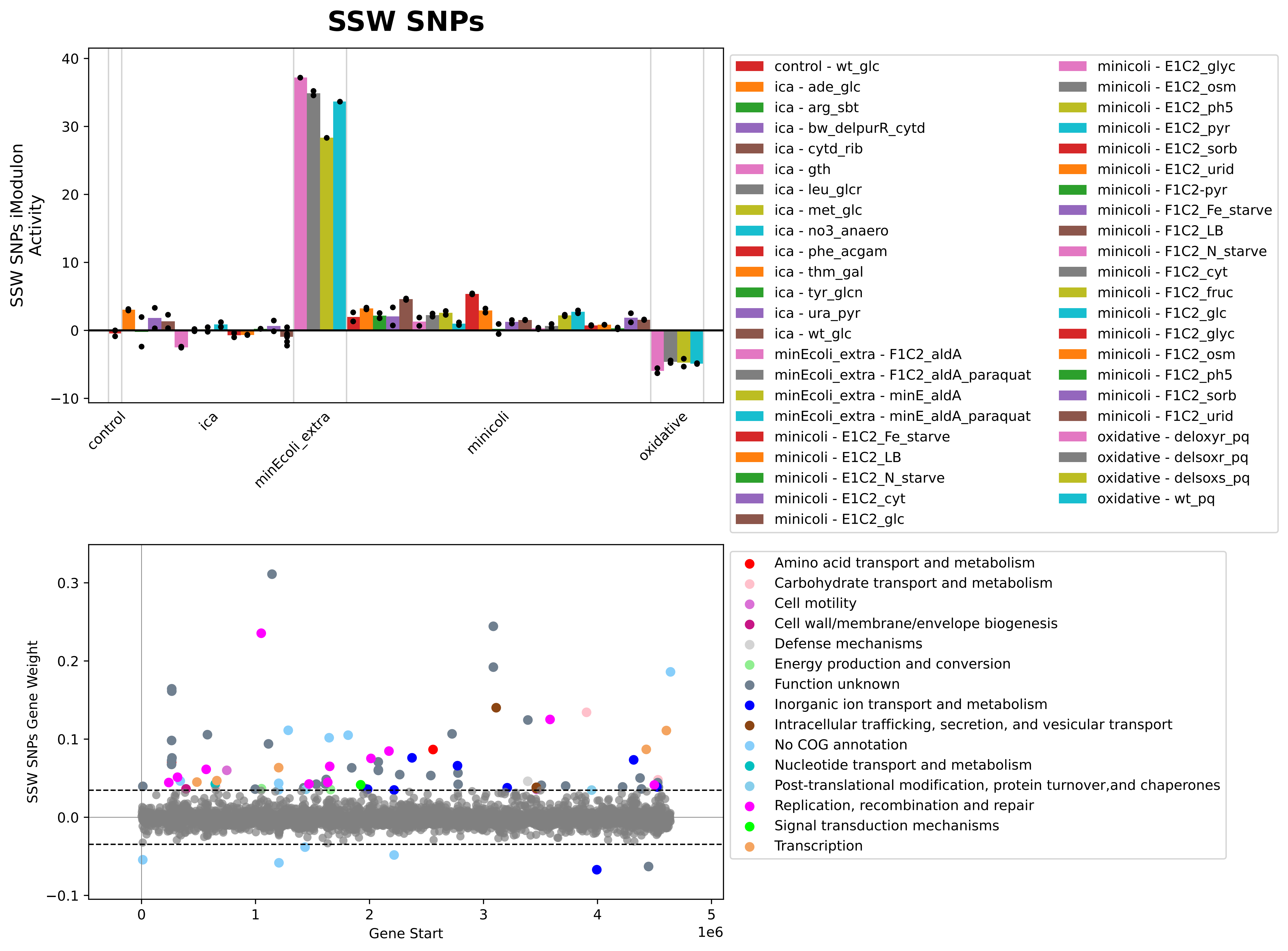

SSW SNPs
SSW SNPs iModulon Activity
control
ica
minEcoli_extra
minicoli
oxidative
control - wt_glc
ica - ade_glc
ica - arg_sbt
ica - bw_delpurR_cytd
ica - cytd_rib
ica - gth
ica - leu_glcr
ica - met_glc
ica - no3_anaero
ica - phe_acgam
ica - thm_gal
ica - tyr_glcn
ica - ura_pyr
ica - wt_glc
minEcoli_extra - F1C2_aldA
minEcoli_extra - F1C2_aldA_paraquat
minEcoli_extra - minE_aldA
minEcoli_extra - minE_aldA_paraquat
minicoli - E1C2_Fe_starve
minicoli - E1C2_LB
minicoli - E1C2_N_starve
minicoli - E1C2_cyt
minicoli - E1C2_glc
minicoli - E1C2_glyc
minicoli - E1C2_osm
minicoli - E1C2_ph5
minicoli - E1C2_pyr
minicoli - E1C2_sorb
minicoli - E1C2_urid
minicoli - F1C2-pyr
minicoli - F1C2_Fe_starve
minicoli - F1C2_LB
minicoli - F1C2_N_starve
minicoli - F1C2_cyt
minicoli - F1C2_fruc
minicoli - F1C2_glc
minicoli - F1C2_glyc
minicoli - F1C2_osm
minicoli - F1C2_ph5
minicoli - F1C2_sorb
minicoli - F1C2_urid
oxidative - deloxyr_pq
oxidative - delsoxr_pq
oxidative - delsoxs_pq
oxidative - wt_pq
SSW SNPs Gene Weight
Gene Start
1e6
Amino acid transport and metabolism
Carbohydrate transport and metabolism
Cell motility
Cell wall/membrane/envelope biogenesis
Defense mechanisms
Energy production and conversion
Function unknown
Inorganic ion transport and metabolism
Intracellular trafficking, secretion, and vesicular transport
No COG annotation
Nucleotide transport and metabolism
Post-translational modification, protein turnover,and chaperones
Replication, recombination and repair
Signal transduction mechanisms
Transcription

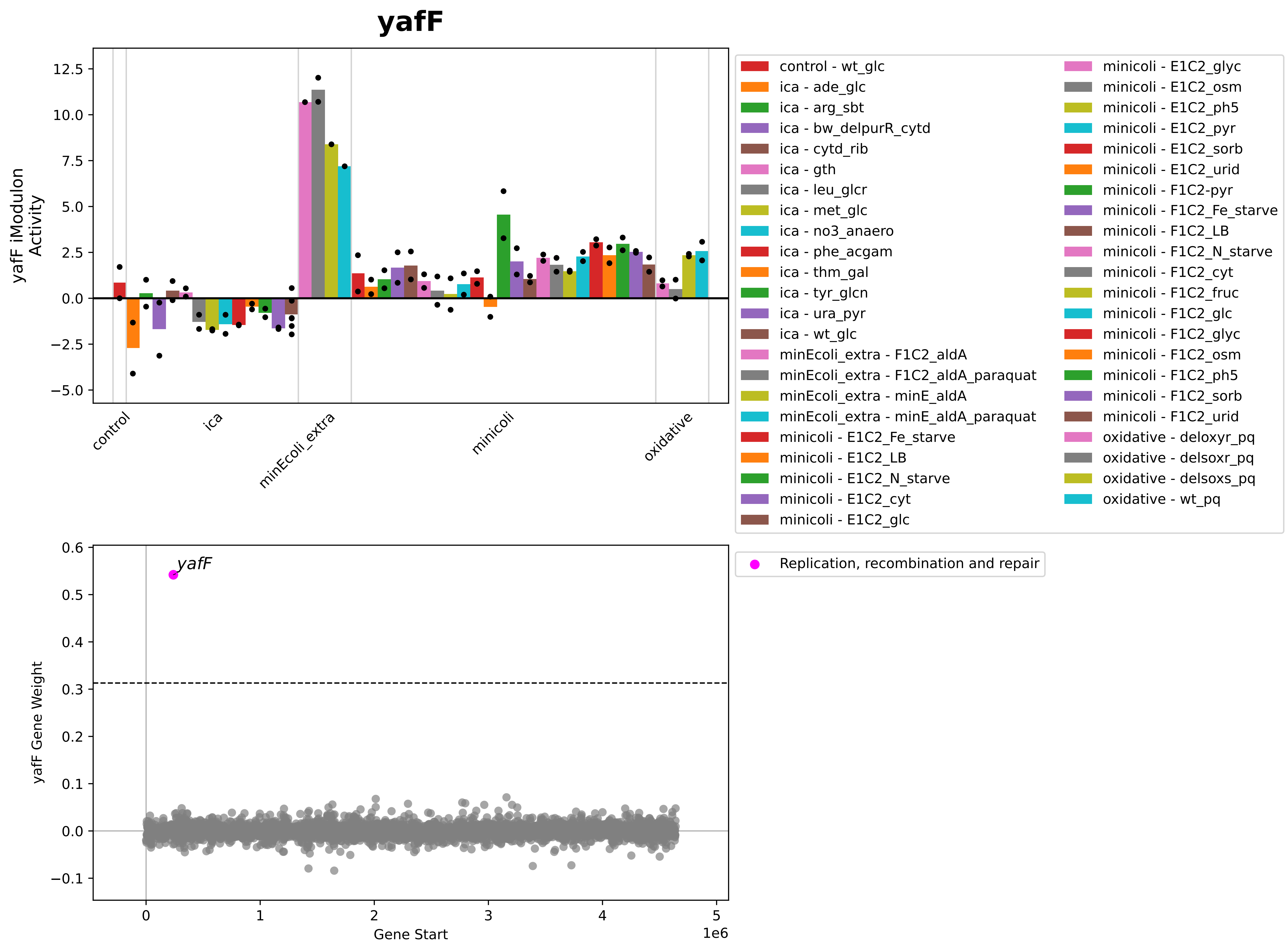
yafF
yafF iModulon Activity
control
ica
minEcoli_extra
minicoli
oxidative
control - wt_glc
ica - ade_glc
ica - arg_sbt
ica - bw_delpurR_cytd
ica - cytd_rib
ica - gth
ica - leu_glcr
ica - met_glc
ica - no3_anaero
ica - phe_acgam
ica - thm_gal
ica - tyr_glcn
ica - ura_pyr
ica - wt_glc
minEcoli_extra - F1C2_aldA
minEcoli_extra - F1C2_aldA_paraquat
minEcoli_extra - minE_aldA
minEcoli_extra - minE_aldA_paraquat
minicoli - E1C2_Fe_starve
minicoli - E1C2_LB
minicoli - E1C2_N_starve
minicoli - E1C2_cyt
minicoli - E1C2_glc
minicoli - E1C2_glyc
minicoli - E1C2_osm
minicoli - E1C2_ph5
minicoli - E1C2_pyr
minicoli - E1C2_sorb
minicoli - E1C2_urid
minicoli - F1C2-pyr
minicoli - F1C2_Fe_starve
minicoli - F1C2_LB
minicoli - F1C2_N_starve
minicoli - F1C2_cyt
minicoli - F1C2_fruc
minicoli - F1C2_glc
minicoli - F1C2_glyc
minicoli - F1C2_osm
minicoli - F1C2_ph5
minicoli - F1C2_sorb
minicoli - F1C2_urid
oxidative - deloxyr_pq
oxidative - delsoxr_pq
oxidative - delsoxs_pq
oxidative - wt_pq
yafF Gene Weight
yafF
Replication, recombination and repair
Gene Start
1e6

NtrC-2

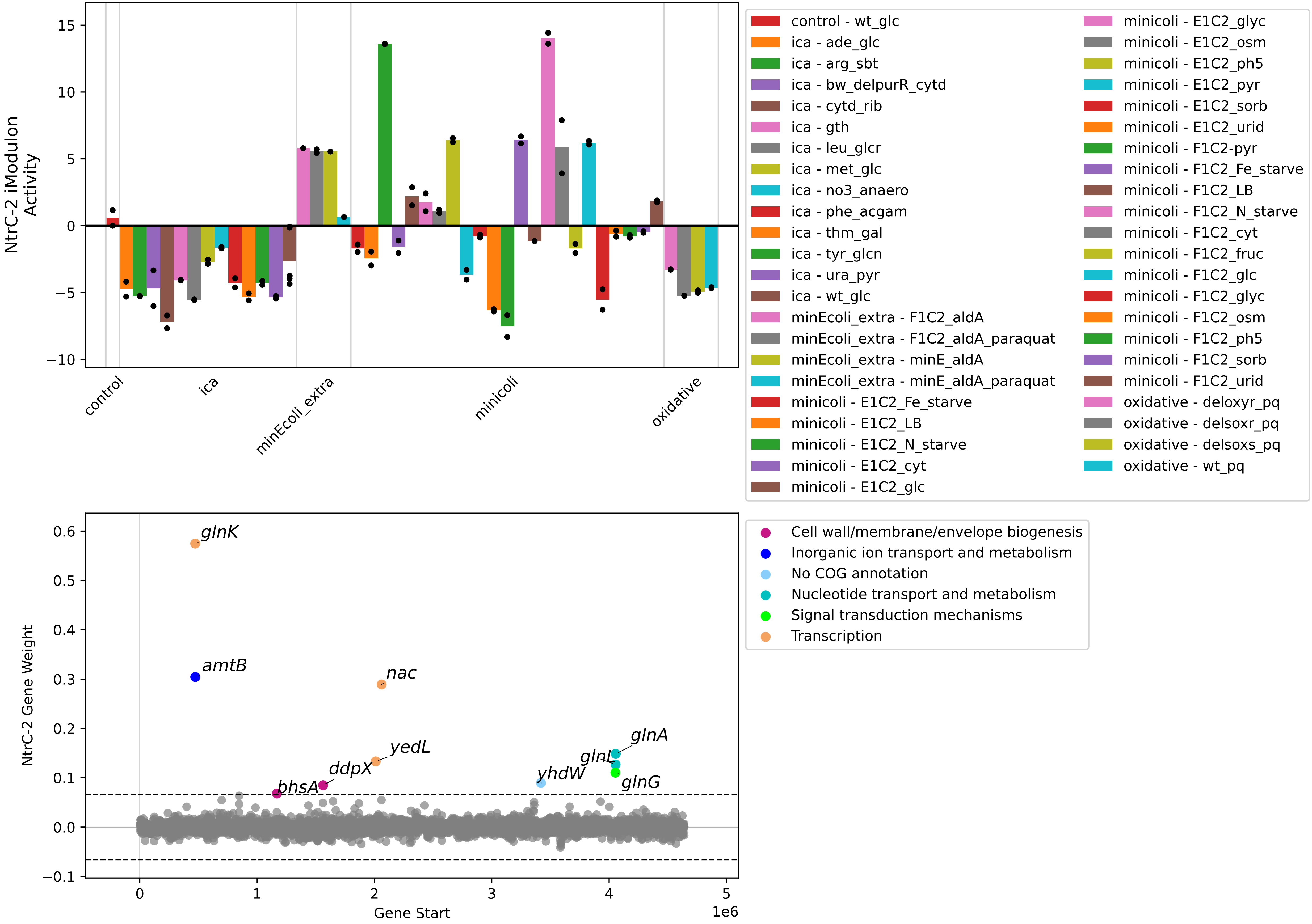

NtrC-2 iModulon Activity
control
ica
minEcoli_extra
minicoli
oxidative
control - wt_glc
ica - ade_glc
ica - arg_sbt
ica - bw_delpurR_cytd
ica - cytd_rib
ica - gth
ica - leu_glcr
ica - met_glc
ica - no3_anaero
ica - phe_acgam
ica - thm_gal
ica - tyr_glcn
ica - ura_pyr
ica - wt_glc
minEcoli_extra - F1C2_aldA
minEcoli_extra - F1C2_aldA_paraquat
minEcoli_extra - minE_aldA
minEcoli_extra - minE_aldA_paraquat
minicoli - E1C2_Fe_starve
minicoli - E1C2_LB
minicoli - E1C2_N_starve
minicoli - E1C2_cyt
minicoli - E1C2_glc
minicoli - E1C2_glyc
minicoli - E1C2_osm
minicoli - E1C2_ph5
minicoli - E1C2_pyr
minicoli - E1C2_sorb
minicoli - E1C2_urid
minicoli - F1C2-pyr
minicoli - F1C2_Fe_starve
minicoli - F1C2_LB
minicoli - F1C2_N_starve
minicoli - F1C2_cyt
minicoli - F1C2_fruc
minicoli - F1C2_glc
minicoli - F1C2_glyc
minicoli - F1C2_osm
minicoli - F1C2_ph5
minicoli - F1C2_sorb
minicoli - F1C2_urid
oxidative - deloxyr_pq
oxidative - delsoxr_pq
oxidative - delsoxs_pq
oxidative - wt_pq
NtrC-2 Gene Weight
Gene Start
1e6
glnK
amtB
nac
yedL
ddpX
bhsA
yhdW
glnL
glnA
glnG
Cell wall/membrane/envelope biogenesis
Inorganic ion transport and metabolism
No COG annotation
Nucleotide transport and metabolism
Signal transduction mechanisms
Transcription

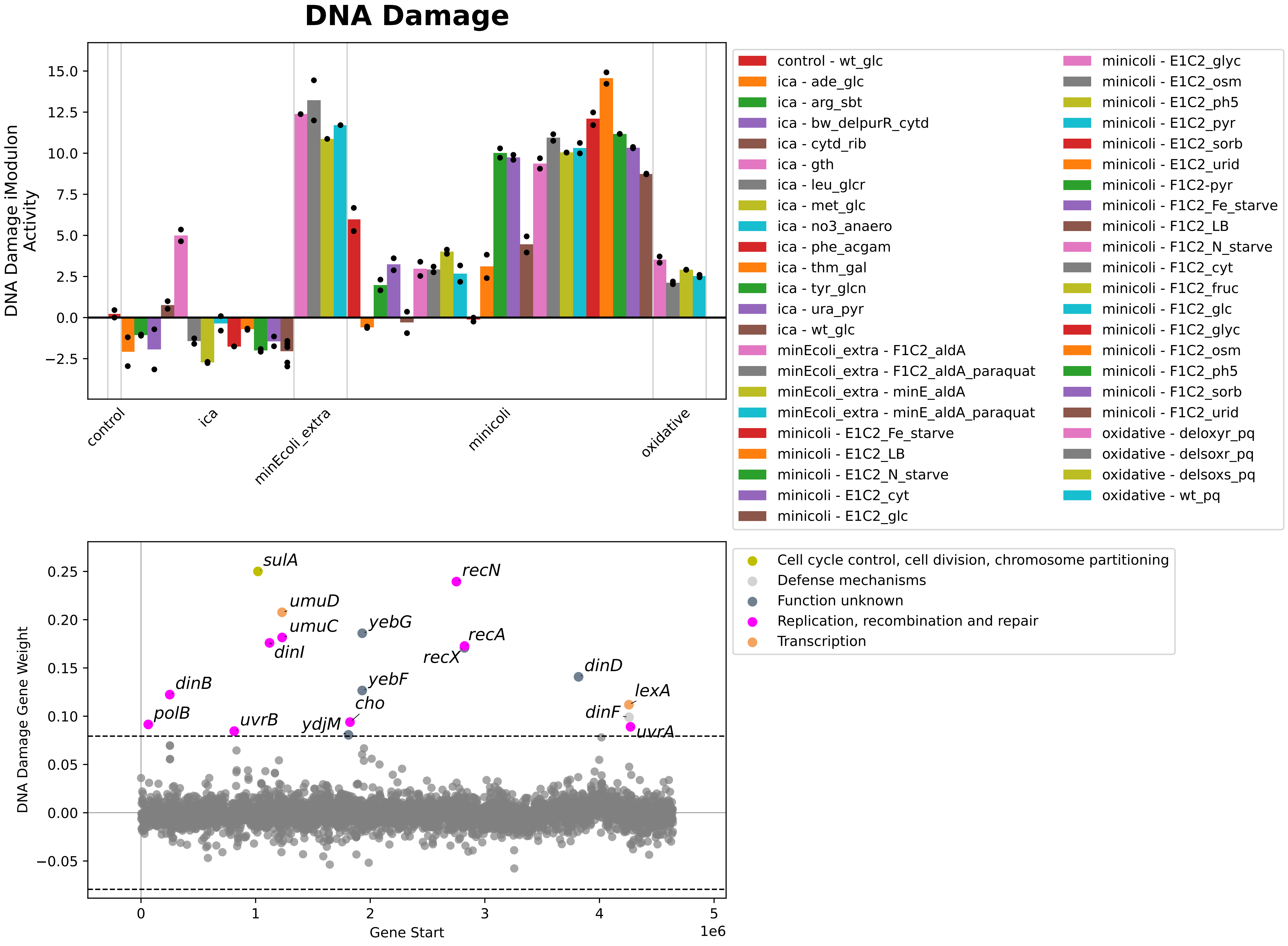

DNA Damage
DNA Damage iModulon Activity
control
ica
minEcoli_extra
minicoli
oxidative
control - wt_glc
ica - ade_glc
ica - arg_sbt
ica - bw_delpurR_cytd
ica - cytd_rib
ica - gth
ica - leu_glcr
ica - met_glc
ica - no3_anaero
ica - phe_acgam
ica - thm_gal
ica - tyr_glcn
ica - ura_pyr
ica - wt_glc
minEcoli_extra - F1C2_aldA
minEcoli_extra - F1C2_aldA_paraquat
minEcoli_extra - minE_aldA
minEcoli_extra - minE_aldA_paraquat
minicoli - E1C2_Fe_starve
minicoli - E1C2_LB
minicoli - E1C2_N_starve
minicoli - E1C2_cyt
minicoli - E1C2_glc
minicoli - E1C2_glyc
minicoli - E1C2_osm
minicoli - E1C2_ph5
minicoli - E1C2_pyr
minicoli - E1C2_sorb
minicoli - E1C2_urid
minicoli - F1C2-pyr
minicoli - F1C2_Fe_starve
minicoli - F1C2_LB
minicoli - F1C2_N_starve
minicoli - F1C2_cyt
minicoli - F1C2_fruc
minicoli - F1C2_glc
minicoli - F1C2_glyc
minicoli - F1C2_osm
minicoli - F1C2_ph5
minicoli - F1C2_sorb
minicoli - F1C2_urid
oxidative - deloxyr_pq
oxidative - delsoxr_pq
oxidative - delsoxs_pq
oxidative - wt_pq
DNA Damage Gene Weight
Gene Start
1e6
sulA
recN
umuD
umuC
yebG
recA
dinI
recX
dinD
dinB
yebF
lexA
cho
polB
uvrB
ydjM
dinF
uvrA
Cell cycle control, cell division, chromosome partitioning
Defense mechanisms
Function unknown
Replication, recombination and repair
Transcription

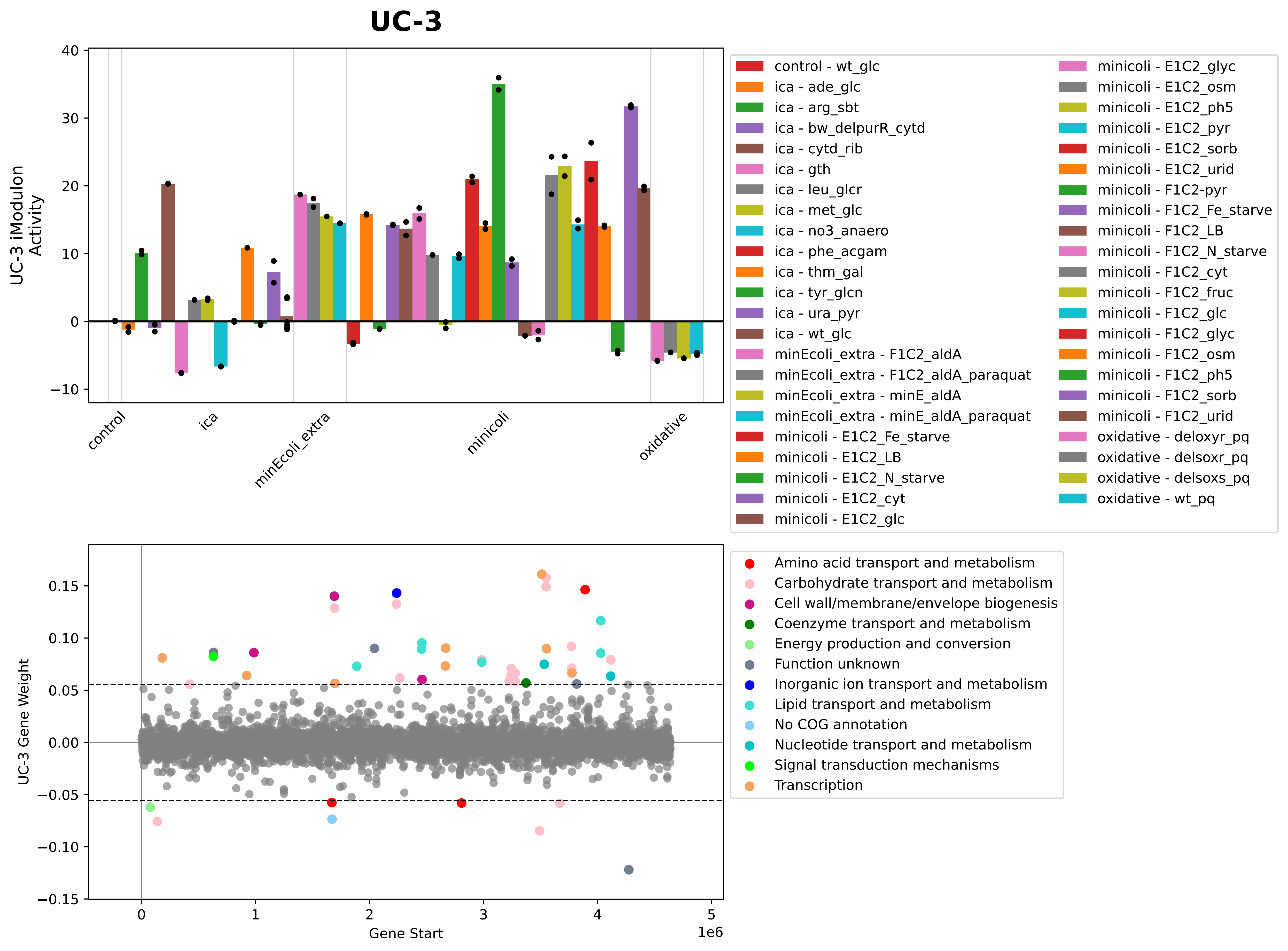
UC-3
UC-3 iModulon Activity
control
ica
minEcoli_extra
minicoli
oxidative
control - wt_glc
ica - ade_glc
ica - arg_sbt
ica - bw_delpurR_cytd
ica - cytd_rib
ica - gth
ica - leu_glcr
ica - met_glc
ica - no3_anaero
ica - phe_acgam
ica - thm_gal
ica - tyr_glcn
ica - ura_pyr
ica - wt_glc
minEcoli_extra - F1C2_aldA
minEcoli_extra - F1C2_aldA_paraquat
minEcoli_extra - minE_aldA
minEcoli_extra - minE_aldA_paraquat
minicoli - E1C2_Fe_starve
minicoli - E1C2_LB
minicoli - E1C2_N_starve
minicoli - E1C2_cyt
minicoli - E1C2_glc
minicoli - E1C2_glyc
minicoli - E1C2_osm
minicoli - E1C2_ph5
minicoli - E1C2_pyr
minicoli - E1C2_sorb
minicoli - E1C2_urid
minicoli - F1C2-pyr
minicoli - F1C2_Fe_starve
minicoli - F1C2_LB
minicoli - F1C2_N_starve
minicoli - F1C2_cyt
minicoli - F1C2_fruc
minicoli - F1C2_glc
minicoli - F1C2_glyc
minicoli - F1C2_osm
minicoli - F1C2_ph5
minicoli - F1C2_sorb
minicoli - F1C2_urid
oxidative - deloxyr_pq
oxidative - delsoxr_pq
oxidative - delsoxs_pq
oxidative - wt_pq
UC-3 Gene Weight
Gene Start
1e6
Amino acid transport and metabolism
Carbohydrate transport and metabolism
Cell wall/membrane/envelope biogenesis
Coenzyme transport and metabolism
Energy production and conversion
Function unknown
Inorganic ion transport and metabolism
Lipid transport and metabolism
No COG annotation
Nucleotide transport and metabolism
Signal transduction mechanisms
Transcription

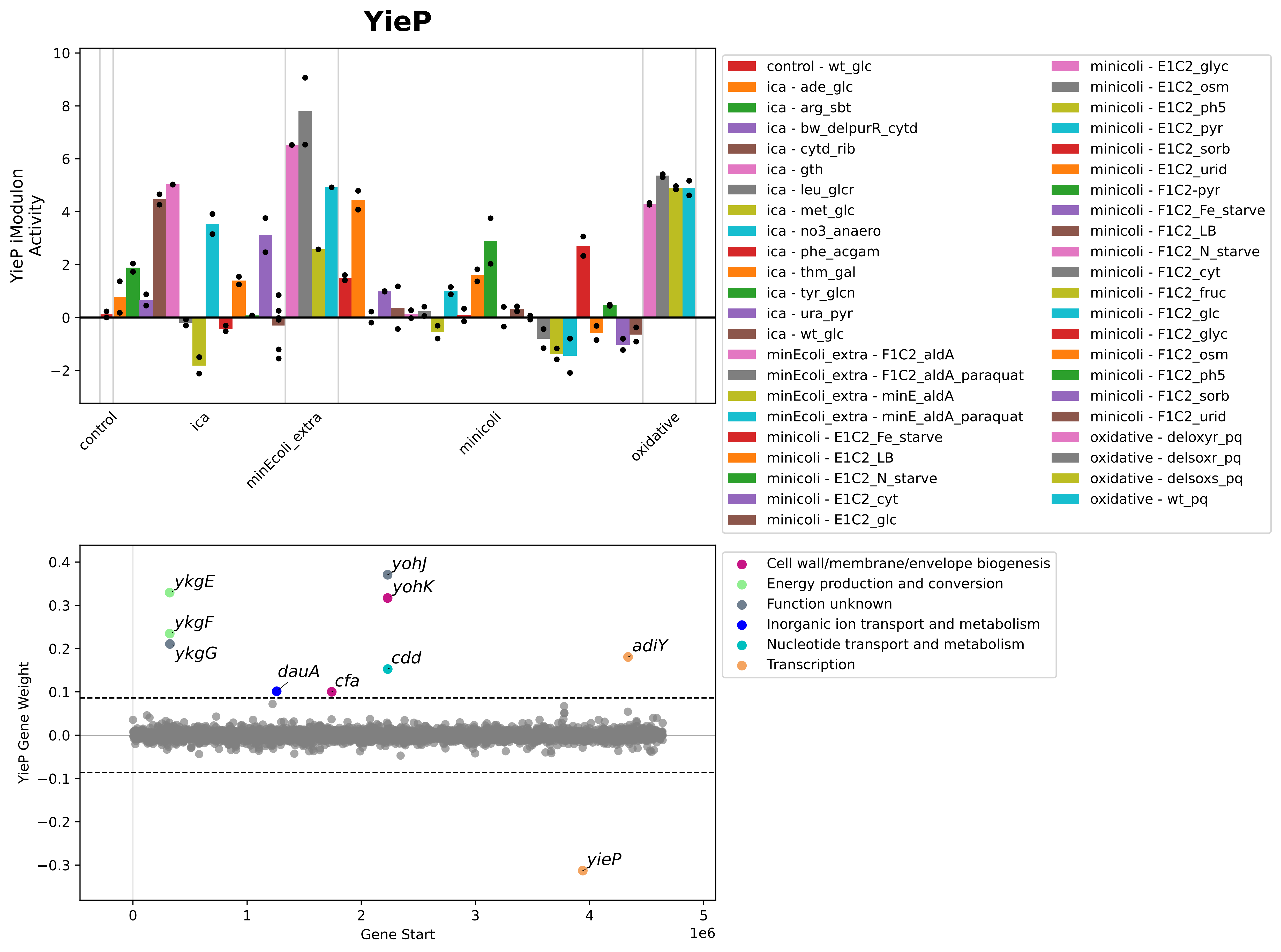

YieP
YieP iModulon Activity
control
ica
minEcoli_extra
minicoli
oxidative
control - wt_glc
ica - ade_glc
ica - arg_sbt
ica - bw_delpurR_cytd
ica - cytd_rib
ica - gth
ica - leu_glcr
ica - met_glc
ica - no3_anaero
ica - phe_acgam
ica - thm_gal
ica - tyr_glcn
ica - ura_pyr
ica - wt_glc
minEcoli_extra - F1C2_aldA
minEcoli_extra - F1C2_aldA_paraquat
minEcoli_extra - minE_aldA
minEcoli_extra - minE_aldA_paraquat
minicoli - E1C2_Fe_starve
minicoli - E1C2_LB
minicoli - E1C2_N_starve
minicoli - E1C2_cyt
minicoli - E1C2_glc
minicoli - E1C2_glyc
minicoli - E1C2_osm
minicoli - E1C2_ph5
minicoli - E1C2_pyr
minicoli - E1C2_sorb
minicoli - E1C2_urid
minicoli - F1C2-pyr
minicoli - F1C2_Fe_starve
minicoli - F1C2_LB
minicoli - F1C2_N_starve
minicoli - F1C2_cyt
minicoli - F1C2_fruc
minicoli - F1C2_glc
minicoli - F1C2_glyc
minicoli - F1C2_osm
minicoli - F1C2_ph5
minicoli - F1C2_sorb
minicoli - F1C2_urid
oxidative - deloxyr_pq
oxidative - delsoxr_pq
oxidative - delsoxs_pq
oxidative - wt_pq
YieP Gene Weight
Gene Start
1e6
ykgE
ykgF
ykgG
yohJ
yohK
dauA
cfa
cdd
adiY
yieP
Cell wall/membrane/envelope biogenesis
Energy production and conversion
Function unknown
Inorganic ion transport and metabolism
Nucleotide transport and metabolism
Transcription

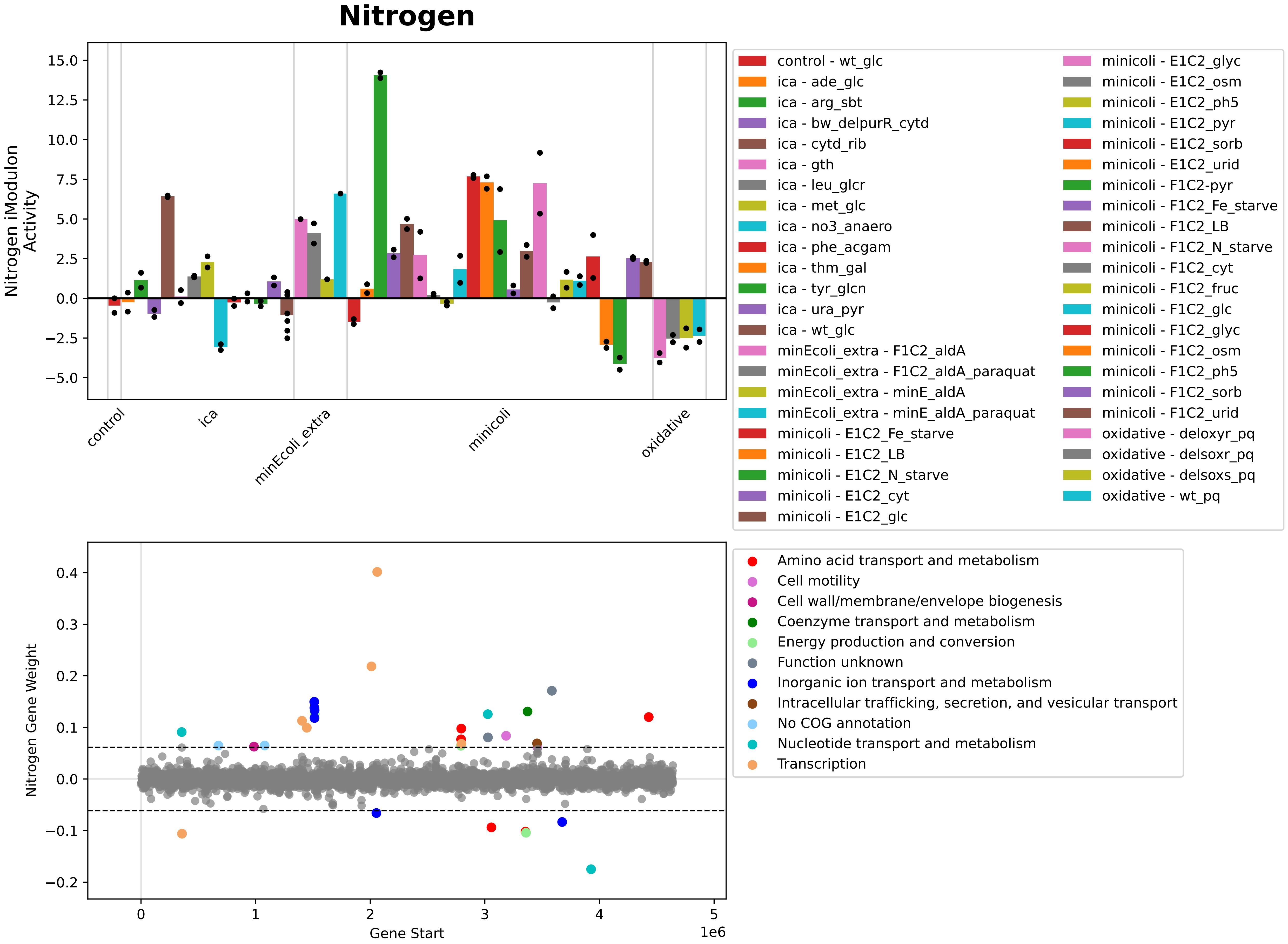
Nitrogen
Nitrogen iModulon Activity
control
ica
minEcoli_extra
minicoli
oxidative
control - wt_glc
ica - ade_glc
ica - arg_sbt
ica - bw_delpurR_cytd
ica - cytd_rib
ica - gth
ica - leu_glcr
ica - met_glc
ica - no3_anaero
ica - phe_acgam
ica - thm_gal
ica - tyr_glcn
ica - ura_pyr
ica - wt_glc
minEcoli_extra - F1C2_aldA
minEcoli_extra - F1C2_aldA_paraquat
minEcoli_extra - minE_aldA
minEcoli_extra - minE_aldA_paraquat
minicoli - E1C2_Fe_starve
minicoli - E1C2_LB
minicoli - E1C2_N_starve
minicoli - E1C2_cyt
minicoli - E1C2_glc
minicoli - E1C2_glyc
minicoli - E1C2_osm
minicoli - E1C2_ph5
minicoli - E1C2_pyr
minicoli - E1C2_sorb
minicoli - E1C2_urid
minicoli - F1C2-pyr
minicoli - F1C2_Fe_starve
minicoli - F1C2_LB
minicoli - F1C2_N_starve
minicoli - F1C2_cyt
minicoli - F1C2_fruc
minicoli - F1C2_glc
minicoli - F1C2_glyc
minicoli - F1C2_osm
minicoli - F1C2_ph5
minicoli - F1C2_sorb
minicoli - F1C2_urid
oxidative - deloxyr_pq
oxidative - delsoxr_pq
oxidative - delsoxs_pq
oxidative - wt_pq
Nitrogen Gene Weight
Gene Start
1e6
Amino acid transport and metabolism
Cell motility
Cell wall/membrane/envelope biogenesis
Coenzyme transport and metabolism
Energy production and conversion
Function unknown
Inorganic ion transport and metabolism
Intracellular trafficking, secretion, and vesicular transport
No COG annotation
Nucleotide transport and metabolism
Transcription

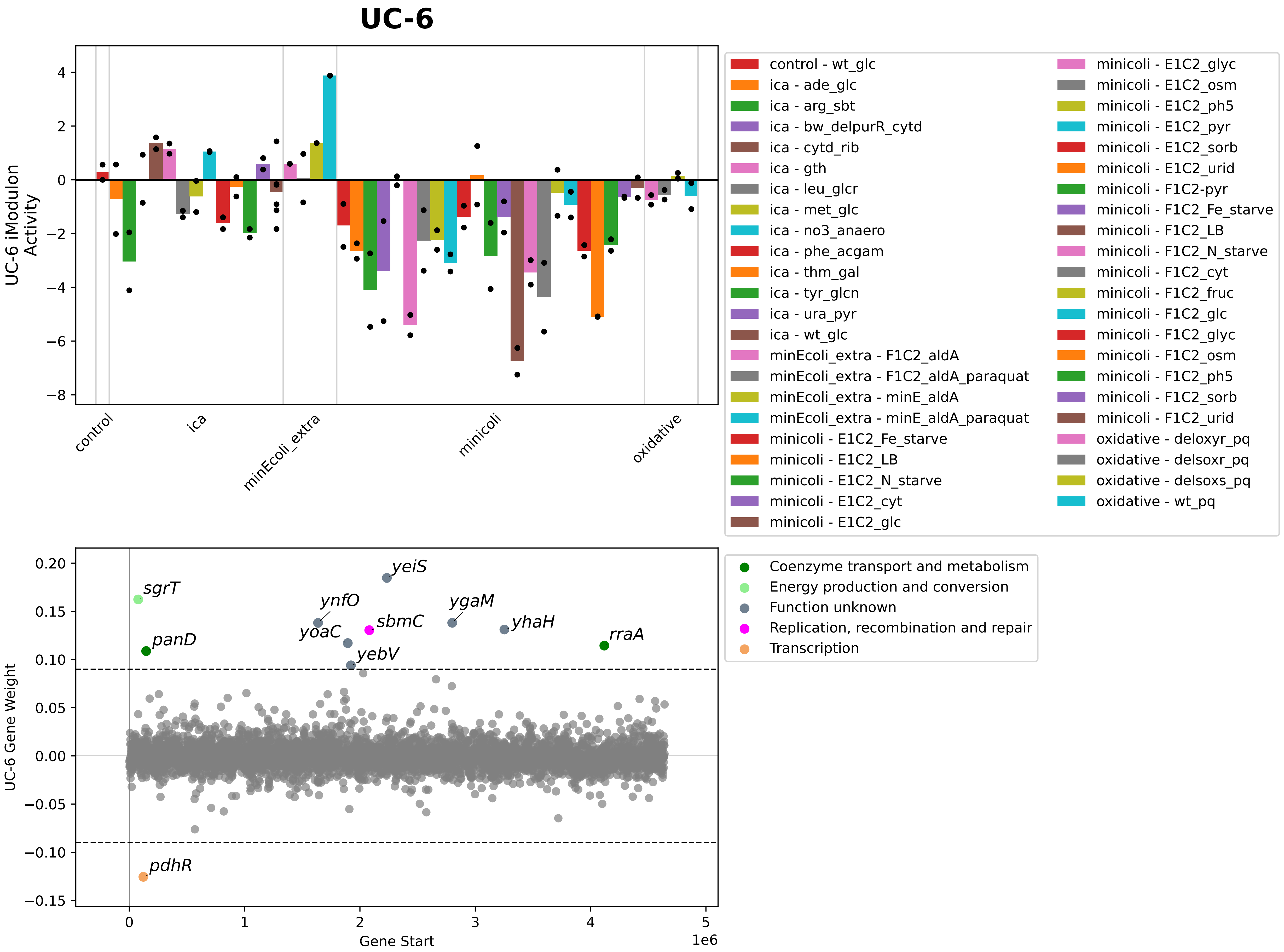
UC-6
UC-6 iModulon Activity
control
ica
minEcoli_extra
minicoli
oxidative
control - wt_glc
ica - ade_glc
ica - arg_sbt
ica - bw_delpurR_cytd
ica - cytd_rib
ica - gth
ica - leu_glcr
ica - met_glc
ica - no3_anaero
ica - phe_acgam
ica - thm_gal
ica - tyr_glcn
ica - ura_pyr
ica - wt_glc
minEcoli_extra - F1C2_aldA
minEcoli_extra - F1C2_aldA_paraquat
minEcoli_extra - minE_aldA
minEcoli_extra - minE_aldA_paraquat
minicoli - E1C2_Fe_starve
minicoli - E1C2_LB
minicoli - E1C2_N_starve
minicoli - E1C2_cyt
minicoli - E1C2_glc
minicoli - E1C2_glyc
minicoli - E1C2_osm
minicoli - E1C2_ph5
minicoli - E1C2_pyr
minicoli - E1C2_sorb
minicoli - E1C2_urid
minicoli - F1C2-pyr
minicoli - F1C2_Fe_starve
minicoli - F1C2_LB
minicoli - F1C2_N_starve
minicoli - F1C2_cyt
minicoli - F1C2_fruc
minicoli - F1C2_glc
minicoli - F1C2_glyc
minicoli - F1C2_osm
minicoli - F1C2_ph5
minicoli - F1C2_sorb
minicoli - F1C2_urid
oxidative - deloxyr_pq
oxidative - delsoxr_pq
oxidative - delsoxs_pq
oxidative - wt_pq
UC-6 Gene Weight
Gene Start
1e6
yeiS
sgrT
ynfO
ygaM
sbmC
yhaH
yoaC
panD
rraA
yebV
pdhR
Coenzyme transport and metabolism
Energy production and conversion
Function unknown
Replication, recombination and repair
Transcription

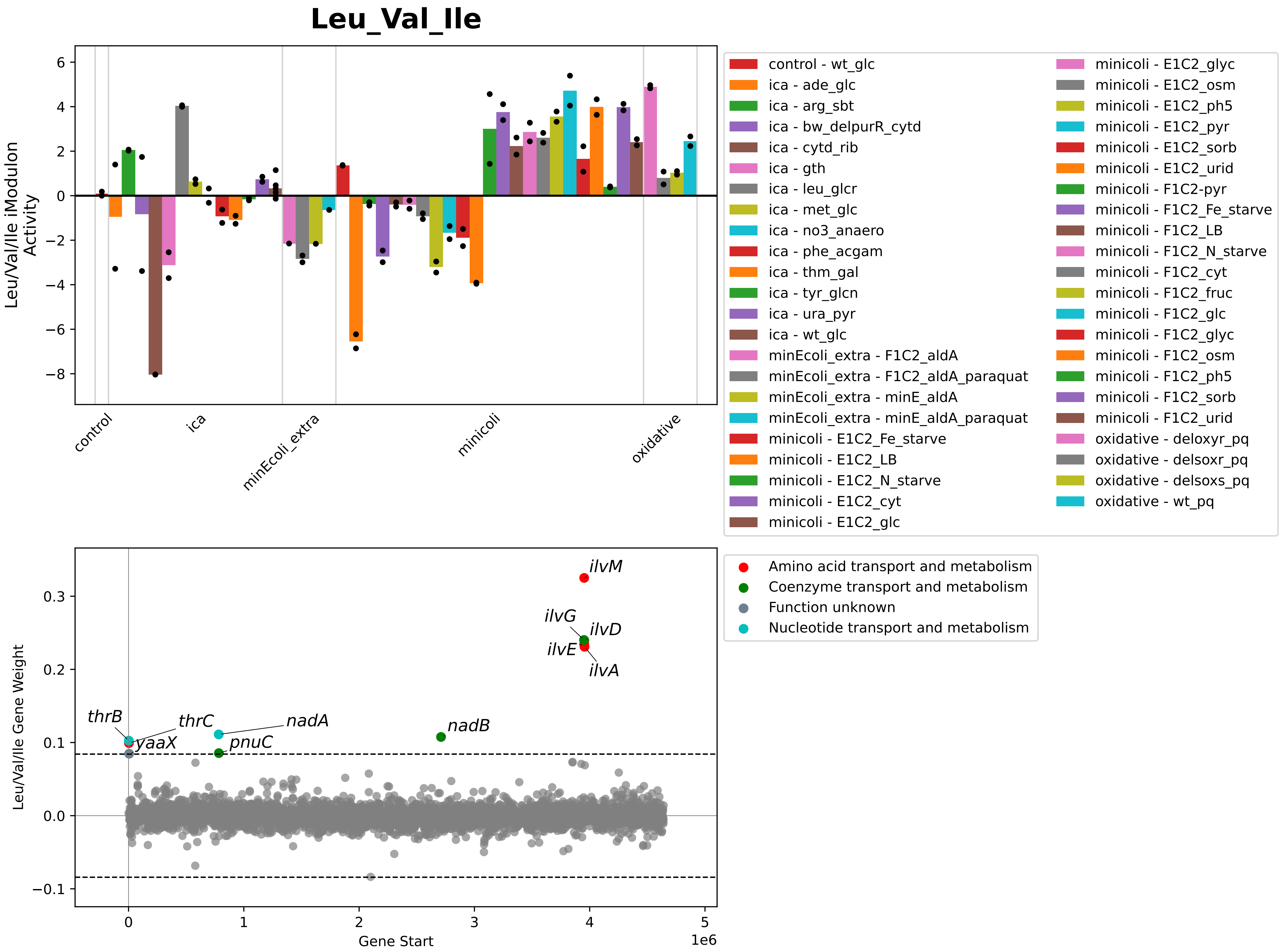

Leu_Val_Ile
Leu/Val/Ile iModulon Activity
control
ica
minEcoli_extra
minicoli
oxidative
control - wt_glc
ica - ade_glc
ica - arg_sbt
ica - bw_delpurR_cytd
ica - cytd_rib
ica - gth
ica - leu_glcr
ica - met_glc
ica - no3_anaero
ica - phe_acgam
ica - thm_gal
ica - tyr_glcn
ica - ura_pyr
ica - wt_glc
minEcoli_extra - F1C2_aldA
minEcoli_extra - F1C2_aldA_paraquat
minEcoli_extra - minE_aldA
minEcoli_extra - minE_aldA_paraquat
minicoli - E1C2_Fe_starve
minicoli - E1C2_LB
minicoli - E1C2_N_starve
minicoli - E1C2_cyt
minicoli - E1C2_glc
minicoli - E1C2_glyc
minicoli - E1C2_osm
minicoli - E1C2_ph5
minicoli - E1C2_pyr
minicoli - E1C2_sorb
minicoli - E1C2_urid
minicoli - F1C2-pyr
minicoli - F1C2_Fe_starve
minicoli - F1C2_LB
minicoli - F1C2_N_starve
minicoli - F1C2_cyt
minicoli - F1C2_fruc
minicoli - F1C2_glc
minicoli - F1C2_glyc
minicoli - F1C2_osm
minicoli - F1C2_ph5
minicoli - F1C2_sorb
minicoli - F1C2_urid
oxidative - deloxyr_pq
oxidative - delsoxr_pq
oxidative - delsoxs_pq
oxidative - wt_pq
Leu/Val/Ile Gene Weight
Gene Start
1e6
ilvM
ilvG
ilvD
ilvE
ilvA
thrB
thrC
nadA
yaaX
pnuC
nadB
Amino acid transport and metabolism
Coenzyme transport and metabolism
Function unknown
Nucleotide transport and metabolism

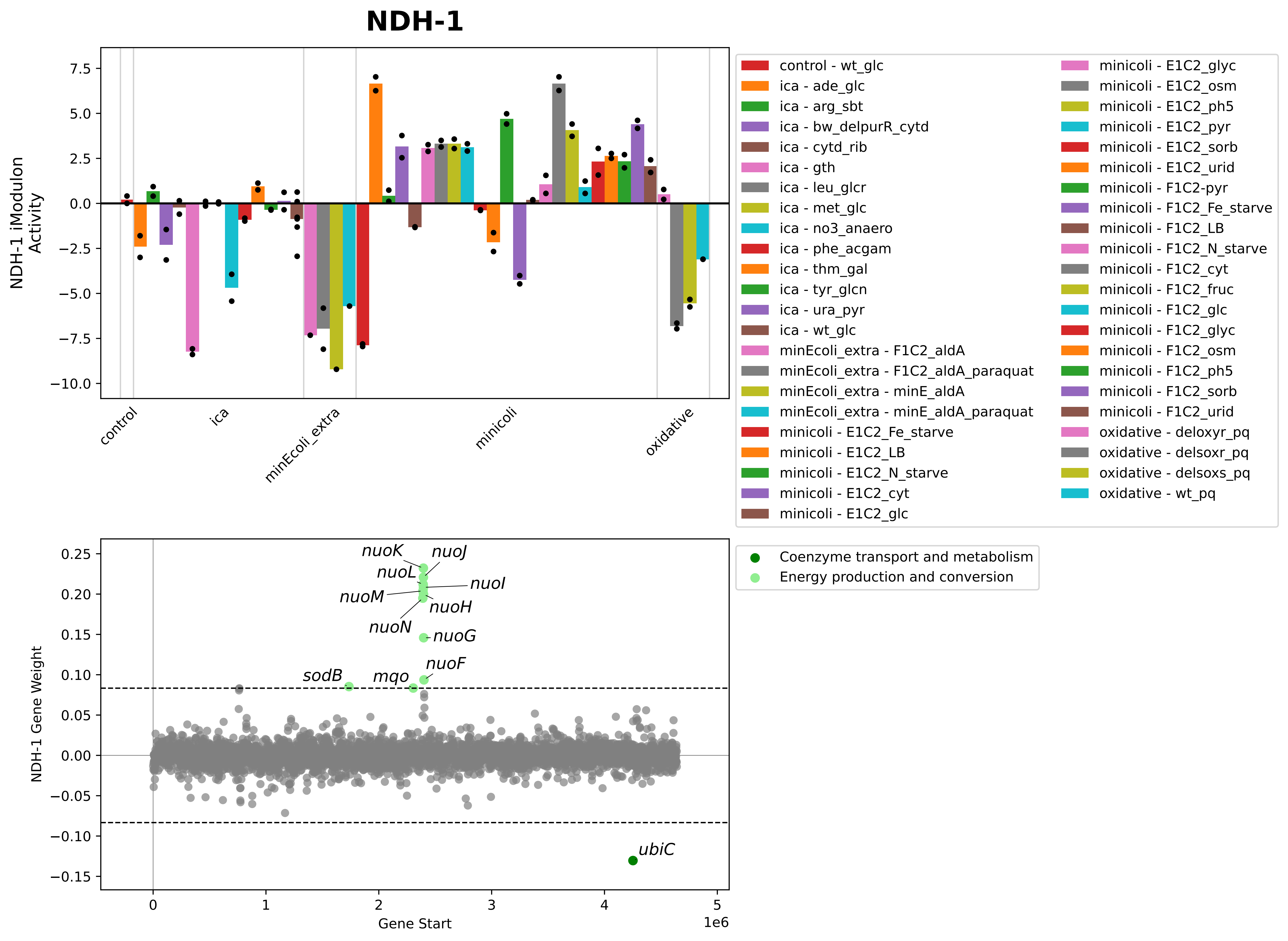

NDH-1
NDH-1 iModulon Activity
7.5
5.0
2.5
0.0
−2.5
−5.0
−7.5
−10.0
control
ica
minEcoli_extra
minicoli
oxidative
control - wt_glc
ica - ade_glc
ica - arg_sbt
ica - bw_delpurR_cytd
ica - cytd_rib
ica - gth
ica - leu_glcr
ica - met_glc
ica - no3_anaero
ica - phe_acgam
ica - thm_gal
ica - tyr_glcn
ica - ura_pyr
ica - wt_glc
minEcoli_extra - F1C2_aldA
minEcoli_extra - F1C2_aldA_paraquat
minEcoli_extra - minE_aldA
minEcoli_extra - minE_aldA_paraquat
minicoli - E1C2_Fe_starve
minicoli - E1C2_LB
minicoli - E1C2_N_starve
minicoli - E1C2_cyt
minicoli - E1C2_glc
minicoli - E1C2_glyc
minicoli - E1C2_osm
minicoli - E1C2_ph5
minicoli - E1C2_pyr
minicoli - E1C2_sorb
minicoli - E1C2_urid
minicoli - F1C2-pyr
minicoli - F1C2_Fe_starve
minicoli - F1C2_LB
minicoli - F1C2_N_starve
minicoli - F1C2_cyt
minicoli - F1C2_fruc
minicoli - F1C2_glc
minicoli - F1C2_glyc
minicoli - F1C2_osm
minicoli - F1C2_ph5
minicoli - F1C2_sorb
minicoli - F1C2_urid
oxidative - deloxyr_pq
oxidative - delsoxr_pq
oxidative - delsoxs_pq
oxidative - wt_pq
NDH-1 Gene Weight
0.25
0.20
0.15
0.10
0.05
0.00
−0.05
−0.10
−0.15
nuoK
nuoJ
nuoL
nuoI
nuoM
nuoH
nuoN
nuoG
nuoF
sodB
mqo
ubiC
0
1
2
3
4
5
1e6
Gene Start
Coenzyme transport and metabolism
Energy production and conversion

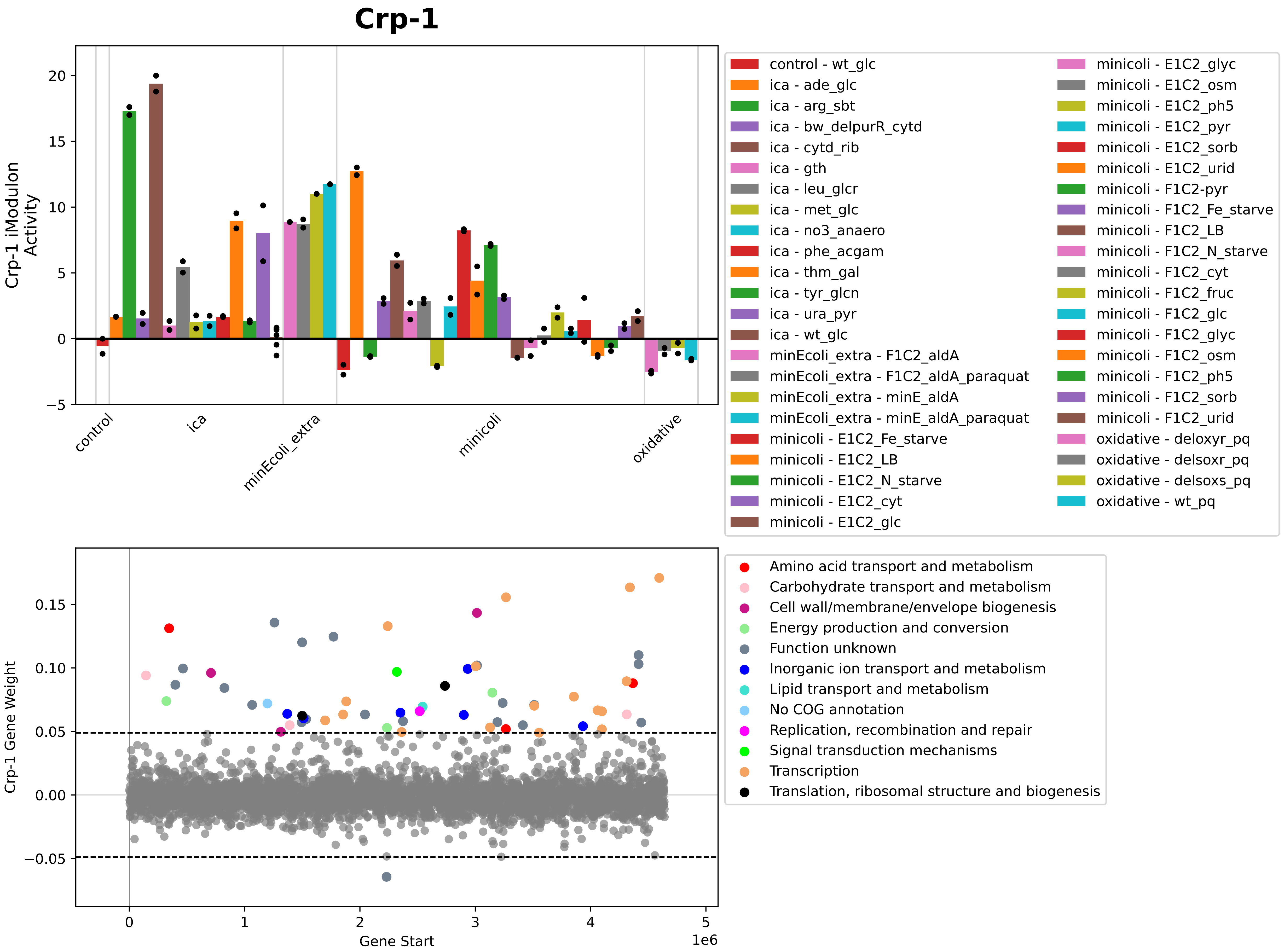

Crp-1
Crp-1 iModulon Activity
control
ica
minEcoli_extra
minicoli
oxidative
control - wt_glc
ica - ade_glc
ica - arg_sbt
ica - bw_delpurR_cytd
ica - cytd_rib
ica - gth
ica - leu_glcr
ica - met_glc
ica - no3_anaero
ica - phe_acgam
ica - thm_gal
ica - tyr_glcn
ica - ura_pyr
ica - wt_glc
minEcoli_extra - F1C2_aldA
minEcoli_extra - F1C2_aldA_paraquat
minEcoli_extra - minE_aldA
minEcoli_extra - minE_aldA_paraquat
minicoli - E1C2_Fe_starve
minicoli - E1C2_LB
minicoli - E1C2_N_starve
minicoli - E1C2_cyt
minicoli - E1C2_glc
minicoli - E1C2_glyc
minicoli - E1C2_osm
minicoli - E1C2_ph5
minicoli - E1C2_pyr
minicoli - E1C2_sorb
minicoli - E1C2_urid
minicoli - F1C2-pyr
minicoli - F1C2_Fe_starve
minicoli - F1C2_LB
minicoli - F1C2_N_starve
minicoli - F1C2_cyt
minicoli - F1C2_fruc
minicoli - F1C2_glc
minicoli - F1C2_glyc
minicoli - F1C2_osm
minicoli - F1C2_ph5
minicoli - F1C2_sorb
minicoli - F1C2_urid
oxidative - deloxyr_pq
oxidative - delsoxr_pq
oxidative - delsoxs_pq
oxidative - wt_pq
Crp-1 Gene Weight
Gene Start
1e6
Amino acid transport and metabolism
Carbohydrate transport and metabolism
Cell wall/membrane/envelope biogenesis
Energy production and conversion
Function unknown
Inorganic ion transport and metabolism
Lipid transport and metabolism
No COG annotation
Replication, recombination and repair
Signal transduction mechanisms
Transcription
Translation, ribosomal structure and biogenesis

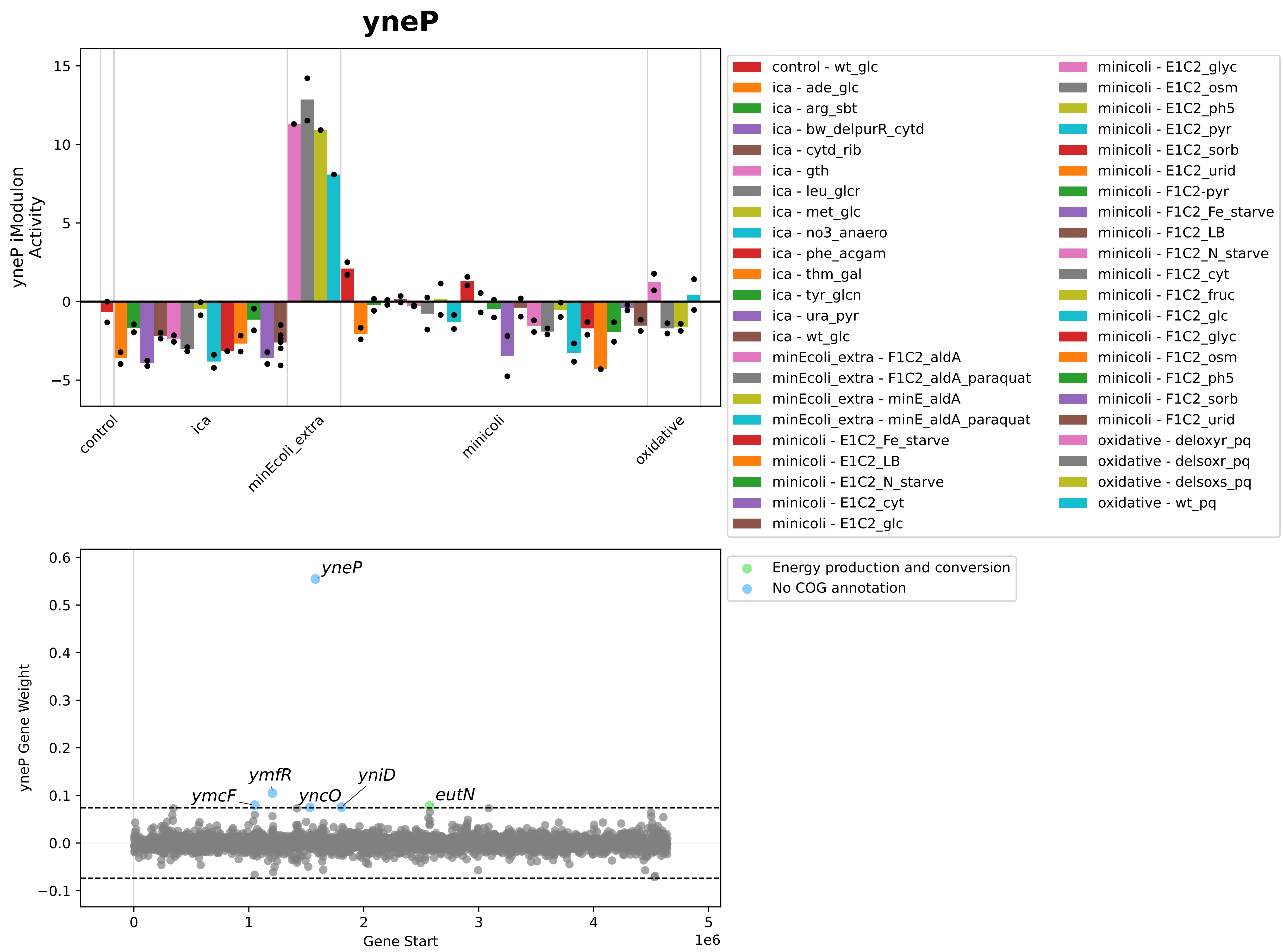

yneP
yneP iModulon Activity
15
10
5
0
−5
control
ica
minEcoli_extra
minicoli
oxidative
control - wt_glc
ica - ade_glc
ica - arg_sbt
ica - bw_delpurR_cytd
ica - cytd_rib
ica - gth
ica - leu_glcr
ica - met_glc
ica - no3_anaero
ica - phe_acgam
ica - thm_gal
ica - tyr_glcn
ica - ura_pyr
ica - wt_glc
minEcoli_extra - F1C2_aldA
minEcoli_extra - F1C2_aldA_paraquat
minEcoli_extra - minE_aldA
minEcoli_extra - minE_aldA_paraquat
minicoli - E1C2_Fe_starve
minicoli - E1C2_LB
minicoli - E1C2_N_starve
minicoli - E1C2_cyt
minicoli - E1C2_glc
minicoli - E1C2_glyc
minicoli - E1C2_osm
minicoli - E1C2_ph5
minicoli - E1C2_pyr
minicoli - E1C2_sorb
minicoli - E1C2_urid
minicoli - F1C2-pyr
minicoli - F1C2_Fe_starve
minicoli - F1C2_LB
minicoli - F1C2_N_starve
minicoli - F1C2_cyt
minicoli - F1C2_fruc
minicoli - F1C2_glc
minicoli - F1C2_glyc
minicoli - F1C2_osm
minicoli - F1C2_ph5
minicoli - F1C2_sorb
minicoli - F1C2_urid
oxidative - deloxyr_pq
oxidative - delsoxr_pq
oxidative - delsoxs_pq
oxidative - wt_pq
yneP Gene Weight
0.6
0.5
0.4
0.3
0.2
0.1
0.0
−0.1
yneP
ymfR
yniD
ymcF
yncO
eutN
0
1
2
3
4
5
1e6
Gene Start
Energy production and conversion
No COG annotation

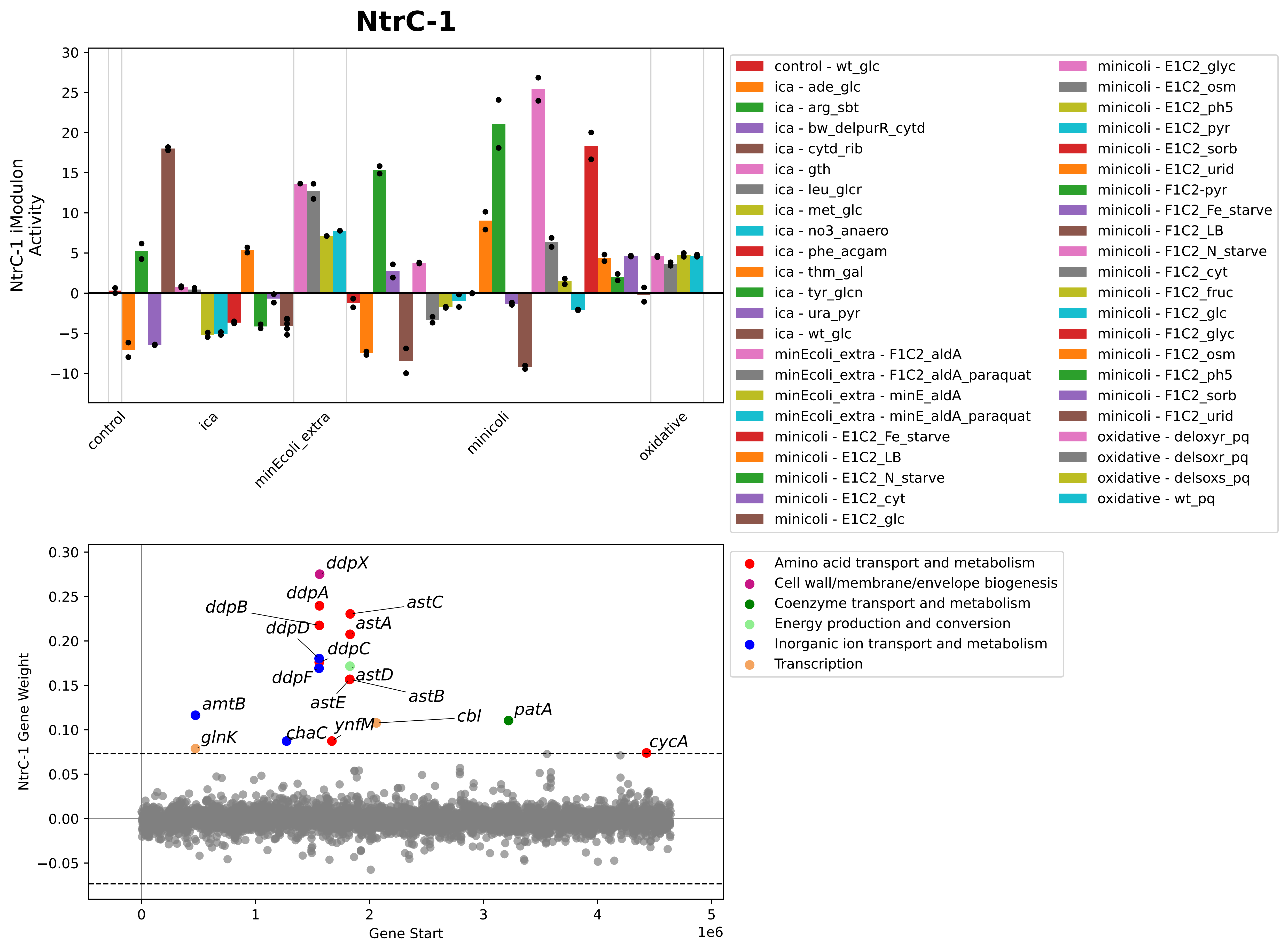
NtrC-1
NtrC-1 iModulon Activity
control
ica
minEcoli_extra
minicoli
oxidative
control - wt_glc
ica - ade_glc
ica - arg_sbt
ica - bw_delpurR_cytd
ica - cytd_rib
ica - gth
ica - leu_glcr
ica - met_glc
ica - no3_anaero
ica - phe_acgam
ica - thm_gal
ica - tyr_glcn
ica - ura_pyr
ica - wt_glc
minEcoli_extra - F1C2_aldA
minEcoli_extra - F1C2_aldA_paraquat
minEcoli_extra - minE_aldA
minEcoli_extra - minE_aldA_paraquat
minicoli - E1C2_Fe_starve
minicoli - E1C2_LB
minicoli - E1C2_N_starve
minicoli - E1C2_cyt
minicoli - E1C2_glc
minicoli - E1C2_glyc
minicoli - E1C2_osm
minicoli - E1C2_ph5
minicoli - E1C2_pyr
minicoli - E1C2_sorb
minicoli - E1C2_urid
minicoli - F1C2-pyr
minicoli - F1C2_Fe_starve
minicoli - F1C2_LB
minicoli - F1C2_N_starve
minicoli - F1C2_cyt
minicoli - F1C2_fruc
minicoli - F1C2_glc
minicoli - F1C2_glyc
minicoli - F1C2_osm
minicoli - F1C2_ph5
minicoli - F1C2_sorb
minicoli - F1C2_urid
oxidative - deloxyr_pq
oxidative - delsoxr_pq
oxidative - delsoxs_pq
oxidative - wt_pq
NtrC-1 Gene Weight
Gene Start
1e6
ddpX
ddpA
ddpB
astC
astA
ddpD
ddpC
ddpF
astD
astB
astE
amtB
cbl
patA
glnK
chaC
ynfM
cycA
Amino acid transport and metabolism
Cell wall/membrane/envelope biogenesis
Coenzyme transport and metabolism
Energy production and conversion
Inorganic ion transport and metabolism
Transcription

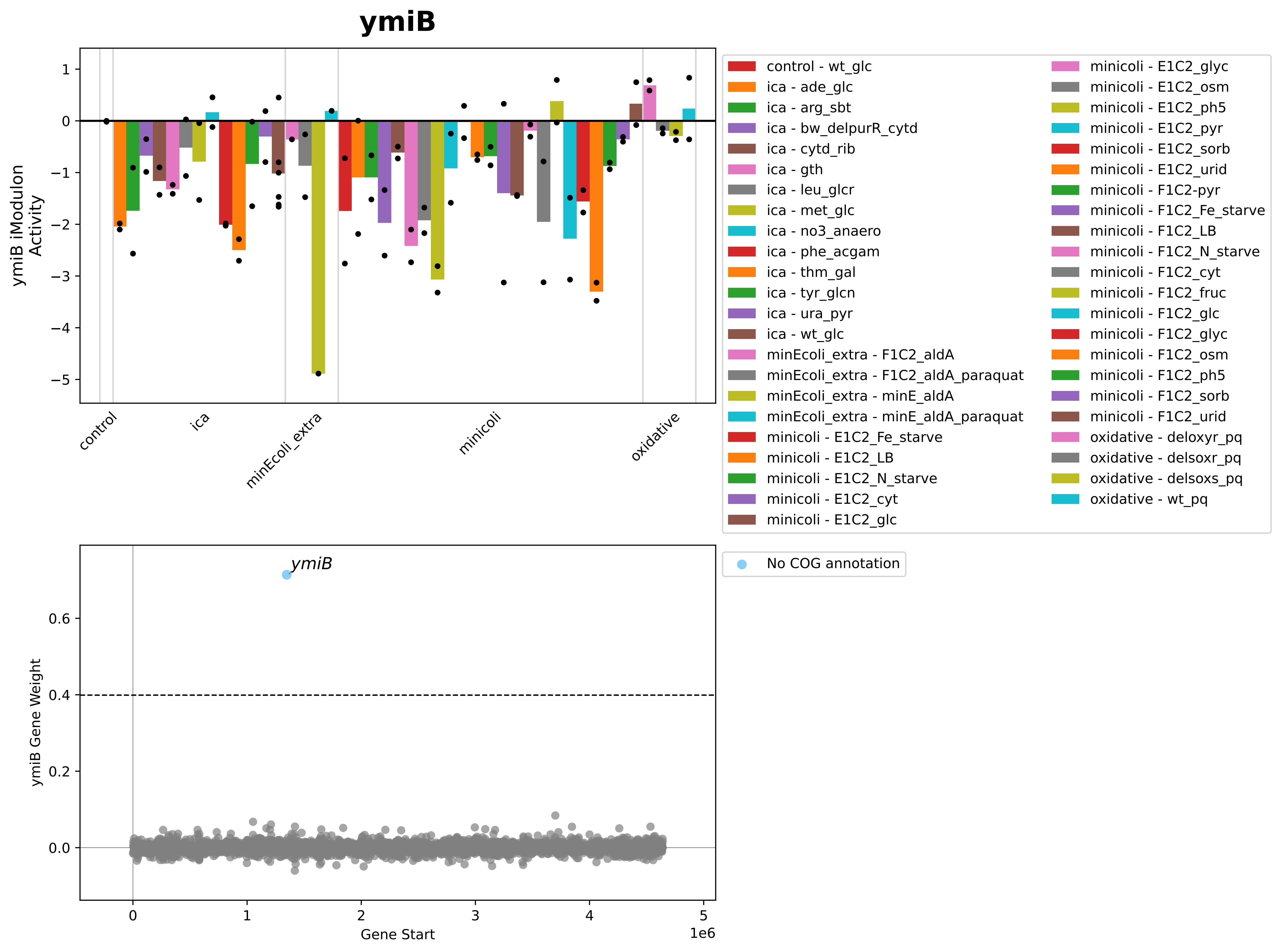
ymiB
ymiB iModulon Activity
control
ica
minEcoli_extra
minicoli
oxidative
control - wt_glc
ica - ade_glc
ica - arg_sbt
ica - bw_delpurR_cytd
ica - cytd_rib
ica - gth
ica - leu_glcr
ica - met_glc
ica - no3_anaero
ica - phe_acgam
ica - thm_gal
ica - tyr_glcn
ica - ura_pyr
ica - wt_glc
minEcoli_extra - F1C2_aldA
minEcoli_extra - F1C2_aldA_paraquat
minEcoli_extra - minE_aldA
minEcoli_extra - minE_aldA_paraquat
minicoli - E1C2_Fe_starve
minicoli - E1C2_LB
minicoli - E1C2_N_starve
minicoli - E1C2_cyt
minicoli - E1C2_glc
minicoli - E1C2_glyc
minicoli - E1C2_osm
minicoli - E1C2_ph5
minicoli - E1C2_pyr
minicoli - E1C2_sorb
minicoli - E1C2_urid
minicoli - F1C2-pyr
minicoli - F1C2_Fe_starve
minicoli - F1C2_LB
minicoli - F1C2_N_starve
minicoli - F1C2_cyt
minicoli - F1C2_fruc
minicoli - F1C2_glc
minicoli - F1C2_glyc
minicoli - F1C2_osm
minicoli - F1C2_ph5
minicoli - F1C2_sorb
minicoli - F1C2_urid
oxidative - deloxyr_pq
oxidative - delsoxr_pq
oxidative - delsoxs_pq
oxidative - wt_pq
ymiB
No COG annotation
ymiB Gene Weight
Gene Start
1e6

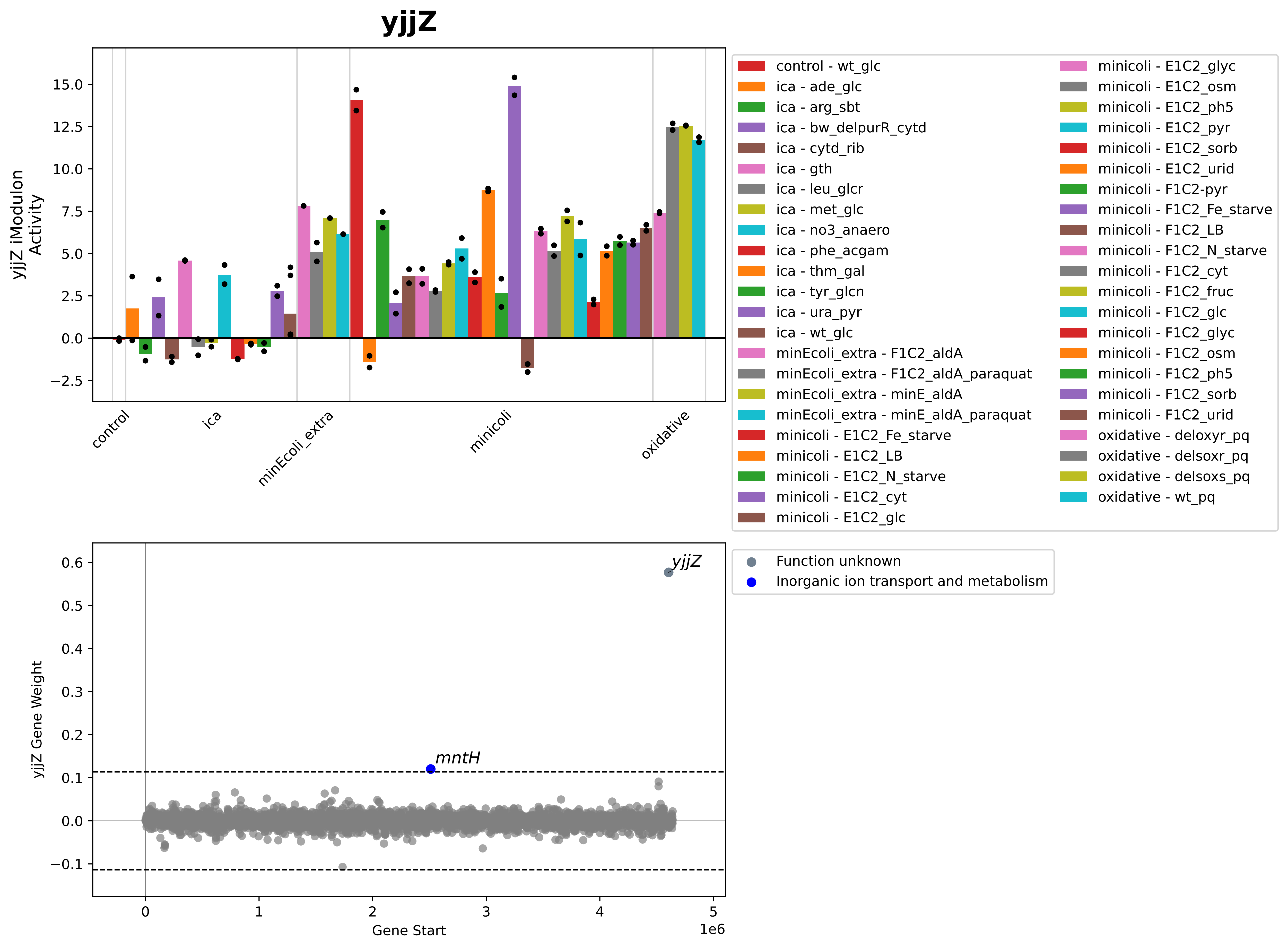
yjjZ
yjjZ iModulon Activity
control
ica
minEcoli_extra
minicoli
oxidative
control - wt_glc
ica - ade_glc
ica - arg_sbt
ica - bw_delpurR_cytd
ica - cytd_rib
ica - gth
ica - leu_glcr
ica - met_glc
ica - no3_anaero
ica - phe_acgam
ica - thm_gal
ica - tyr_glcn
ica - ura_pyr
ica - wt_glc
minEcoli_extra - F1C2_aldA
minEcoli_extra - F1C2_aldA_paraquat
minEcoli_extra - minE_aldA
minEcoli_extra - minE_aldA_paraquat
minicoli - E1C2_Fe_starve
minicoli - E1C2_LB
minicoli - E1C2_N_starve
minicoli - E1C2_cyt
minicoli - E1C2_glc
minicoli - E1C2_glyc
minicoli - E1C2_osm
minicoli - E1C2_ph5
minicoli - E1C2_pyr
minicoli - E1C2_sorb
minicoli - E1C2_urid
minicoli - F1C2-pyr
minicoli - F1C2_Fe_starve
minicoli - F1C2_LB
minicoli - F1C2_N_starve
minicoli - F1C2_cyt
minicoli - F1C2_fruc
minicoli - F1C2_glc
minicoli - F1C2_glyc
minicoli - F1C2_osm
minicoli - F1C2_ph5
minicoli - F1C2_sorb
minicoli - F1C2_urid
oxidative - deloxyr_pq
oxidative - delsoxr_pq
oxidative - delsoxs_pq
oxidative - wt_pq
yjjZ Gene Weight
Gene Start
1e6
yjjZ
mntH
Function unknown
Inorganic ion transport and metabolism

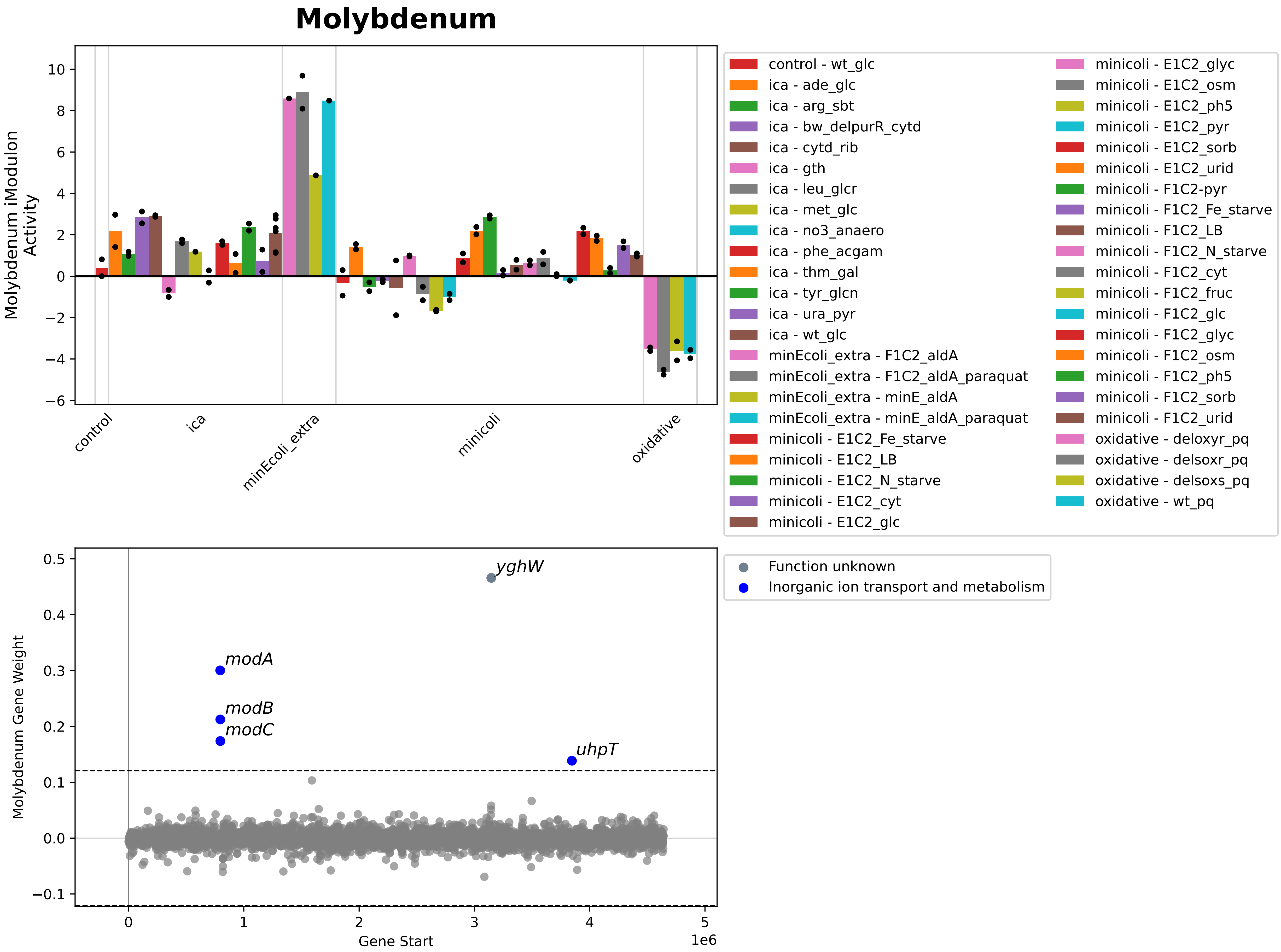

Molybdenum
Molybdenum iModulon Activity
control
ica
minEcoli_extra
minicoli
oxidative
control - wt_glc
ica - ade_glc
ica - arg_sbt
ica - bw_delpurR_cytd
ica - cytd_rib
ica - gth
ica - leu_glcr
ica - met_glc
ica - no3_anaero
ica - phe_acgam
ica - thm_gal
ica - tyr_glcn
ica - ura_pyr
ica - wt_glc
minEcoli_extra - F1C2_aldA
minEcoli_extra - F1C2_aldA_paraquat
minEcoli_extra - minE_aldA
minEcoli_extra - minE_aldA_paraquat
minicoli - E1C2_Fe_starve
minicoli - E1C2_LB
minicoli - E1C2_N_starve
minicoli - E1C2_cyt
minicoli - E1C2_glc
minicoli - E1C2_glyc
minicoli - E1C2_osm
minicoli - E1C2_ph5
minicoli - E1C2_pyr
minicoli - E1C2_sorb
minicoli - E1C2_urid
minicoli - F1C2-pyr
minicoli - F1C2_Fe_starve
minicoli - F1C2_LB
minicoli - F1C2_N_starve
minicoli - F1C2_cyt
minicoli - F1C2_fruc
minicoli - F1C2_glc
minicoli - F1C2_glyc
minicoli - F1C2_osm
minicoli - F1C2_ph5
minicoli - F1C2_sorb
minicoli - F1C2_urid
oxidative - deloxyr_pq
oxidative - delsoxr_pq
oxidative - delsoxs_pq
oxidative - wt_pq
Molybdenum Gene Weight
Gene Start
1e6
yghW
modA
modB
modC
uhpT
Function unknown
Inorganic ion transport and metabolism

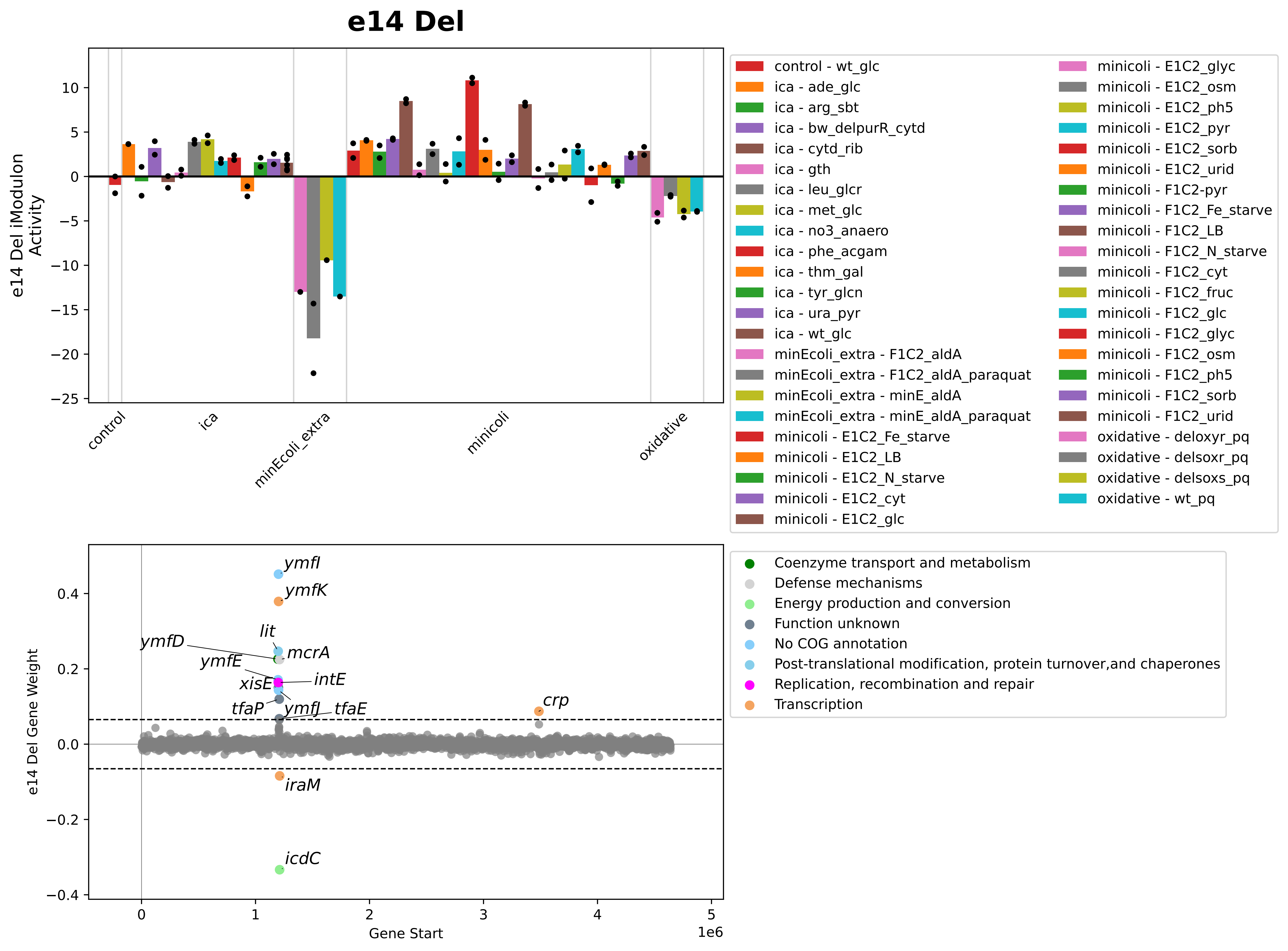

e14 Del
e14 Del iModulon Activity
control
ica
minEcoli_extra
minicoli
oxidative
control - wt_glc
ica - ade_glc
ica - arg_sbt
ica - bw_delpurR_cytd
ica - cytd_rib
ica - gth
ica - leu_glcr
ica - met_glc
ica - no3_anaero
ica - phe_acgam
ica - thm_gal
ica - tyr_glcn
ica - ura_pyr
ica - wt_glc
minEcoli_extra - F1C2_aldA
minEcoli_extra - F1C2_aldA_paraquat
minEcoli_extra - minE_aldA
minEcoli_extra - minE_aldA_paraquat
minicoli - E1C2_Fe_starve
minicoli - E1C2_LB
minicoli - E1C2_N_starve
minicoli - E1C2_cyt
minicoli - E1C2_glc
minicoli - E1C2_glyc
minicoli - E1C2_osm
minicoli - E1C2_ph5
minicoli - E1C2_pyr
minicoli - E1C2_sorb
minicoli - E1C2_urid
minicoli - F1C2-pyr
minicoli - F1C2_Fe_starve
minicoli - F1C2_LB
minicoli - F1C2_N_starve
minicoli - F1C2_cyt
minicoli - F1C2_fruc
minicoli - F1C2_glc
minicoli - F1C2_glyc
minicoli - F1C2_osm
minicoli - F1C2_ph5
minicoli - F1C2_sorb
minicoli - F1C2_urid
oxidative - deloxyr_pq
oxidative - delsoxr_pq
oxidative - delsoxs_pq
oxidative - wt_pq
e14 Del Gene Weight
Gene Start
1e6
ymfI
ymfK
lit
ymfD
mcrA
ymfE
intE
xisE
tfaP
ymfJ
tfaE
crp
iraM
icdC
Coenzyme transport and metabolism
Defense mechanisms
Energy production and conversion
Function unknown
No COG annotation
Post-translational modification, protein turnover,and chaperones
Replication, recombination and repair
Transcription

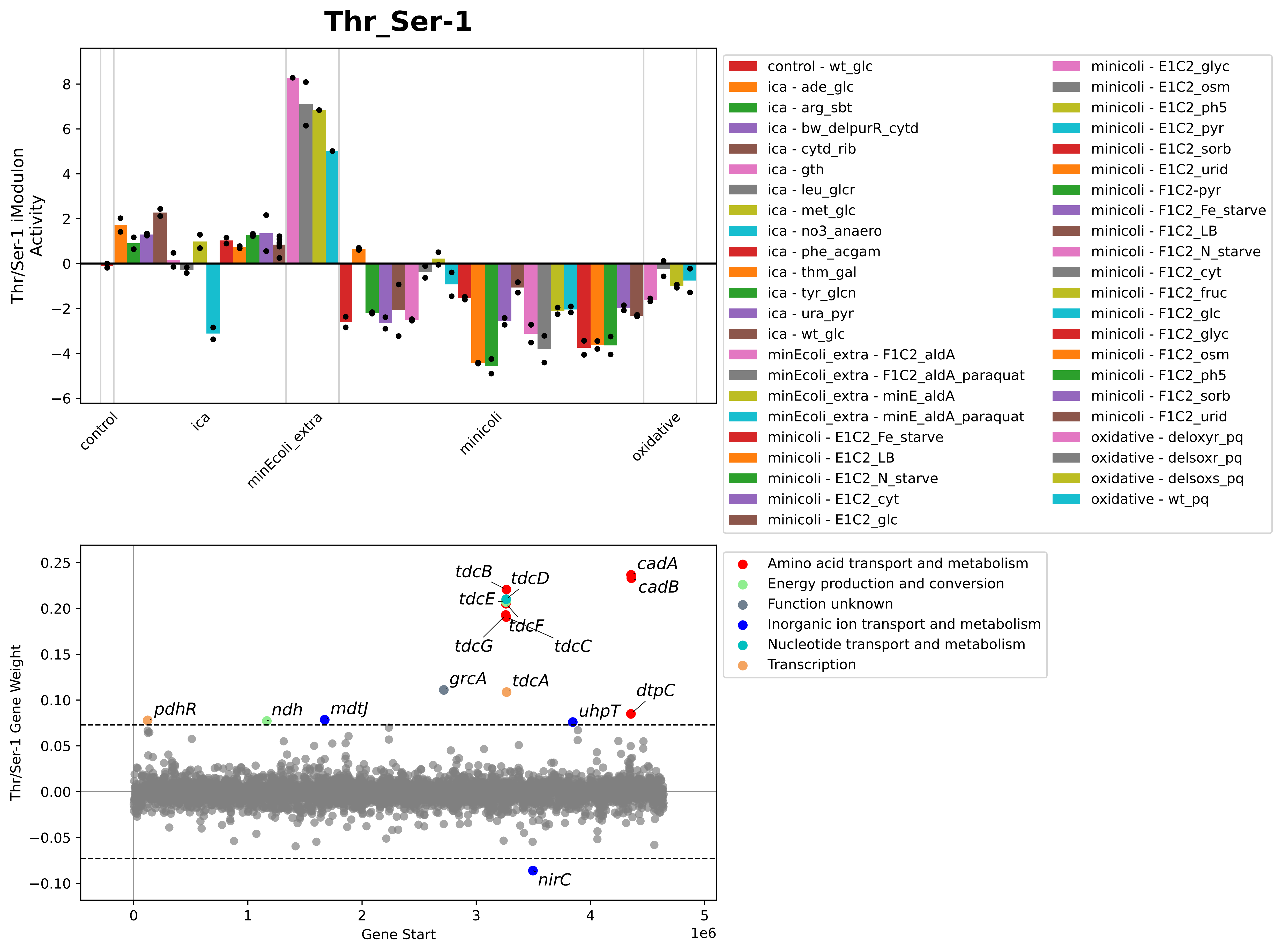
Thr_Ser-1
Thr/Ser-1 iModulon Activity
control
ica
minEcoli_extra
minicoli
oxidative
control - wt_glc
ica - ade_glc
ica - arg_sbt
ica - bw_delpurR_cytd
ica - cytd_rib
ica - gth
ica - leu_glcr
ica - met_glc
ica - no3_anaero
ica - phe_acgam
ica - thm_gal
ica - tyr_glcn
ica - ura_pyr
ica - wt_glc
minEcoli_extra - F1C2_aldA
minEcoli_extra - F1C2_aldA_paraquat
minEcoli_extra - minE_aldA
minEcoli_extra - minE_aldA_paraquat
minicoli - E1C2_Fe_starve
minicoli - E1C2_LB
minicoli - E1C2_N_starve
minicoli - E1C2_cyt
minicoli - E1C2_glc
minicoli - E1C2_glyc
minicoli - E1C2_osm
minicoli - E1C2_ph5
minicoli - E1C2_pyr
minicoli - E1C2_sorb
minicoli - E1C2_urid
minicoli - F1C2-pyr
minicoli - F1C2_Fe_starve
minicoli - F1C2_LB
minicoli - F1C2_N_starve
minicoli - F1C2_cyt
minicoli - F1C2_fruc
minicoli - F1C2_glc
minicoli - F1C2_glyc
minicoli - F1C2_osm
minicoli - F1C2_ph5
minicoli - F1C2_sorb
minicoli - F1C2_urid
oxidative - deloxyr_pq
oxidative - delsoxr_pq
oxidative - delsoxs_pq
oxidative - wt_pq
Thr/Ser-1 Gene Weight
Gene Start
1e6
tdcB
tdcD
tdcE
tdcF
tdcG
tdcC
cadA
cadB
grcA
tdcA
dtpC
pdhR
ndh
mdtJ
uhpT
nirC
Amino acid transport and metabolism
Energy production and conversion
Function unknown
Inorganic ion transport and metabolism
Nucleotide transport and metabolism
Transcription
