# Supplementary material for: Diagnosis and mitigation of the systemic impact of genome reduction in Escherichia coli DGF-298
Source: mBio. 2024 Aug 29;15(10):e00873-24. doi: 10.1128/mbio.00873-24 (PMC11481515; doi:10.1128/mbio.00873-24)
Supplement: Supplemental figures — Figures S1 to S15. [file mbio.00873-24-s0002.docx]

**SUPPLEMENTARY MATERIALS**

**Diagnosis and Mitigation of the Systemic Impact of Extensive Genome Reduction in *Escherichia coli* DGF-298**

Antoine Champie^1^, Jean-Christophe Lachance^1^, Anand Sastry^2^, Dominick Matteau^1^, Colton J. Lloyd^2^, Frédéric Grenier^1^, Cameron R. Lamoureux^2^, Simon Jeanneau^1^, Adam M. Feist^2,5^, Pierre-Étienne Jacques^1^, Bernhard O. Palsson^2,3,4,5^ & Sébastien Rodrigue^1†^

### **Author affiliations**

1. Département de Biologie, Université de Sherbrooke, Sherbrooke, Québec, Canada
2. Department of Bioengineering, University of California, San Diego, La Jolla, USA
3. Bioinformatics and Systems Biology Program, University of California, San Diego, La Jolla, USA
4. Department of Pediatrics, University of California, San Diego, La Jolla, CA, USA
5. Novo Nordisk Foundation Center for Biosustainability, Technical University of Denmark, Kemitorvet, Building 220, 2800 Kongens, Lyngby, Denmark

### **Correspondence**

^†^To whom correspondence should be addressed:

Sébastien Rodrigue

Université de Sherbrooke, Sherbrooke

2500 Boul. de l’Université

Sherbrooke, QC J1K 2R1

+18198218000 #62939 (tel.)

+18198218049 (fax)

sebastien.rodrigue@usherbrooke.ca

## **Supplementary Materials include:**

## Supplementary Figures:

- **Supplementary Figure S1. Simulated growth rate of *E. coli* MG1655 and the genome-reduced DGF-298 strain complemented with different genes to compensate for the accumulation of glycolaldehyde.**
- **Supplementary Figure S2. Metabolic map and predicted fluxes of glycolaldehyde disposal in DGF-298 upon different gene/reaction complementations.**
- **Supplementary Figure S3. Metabolic map and predicted fluxes of folate synthesis in *E. coli* MG1655.**
- **Supplementary Figure S4. Metabolic map and predicted fluxes of folate synthesis in DGF-298.**
- **Supplementary Figure S5. Differential iModulon analysis (DIMA) plot of MG1655 from PRECISE database vs MG1655 in our dataset.**
- **Supplementary Figure S6. Differential iModulon analysis (DIMA) plot of MG1655 from PRECISE database vs W3110S in our dataset.**
- **Supplementary Figure S7. Differential iModulon analysis (DIMA) plot of W3110S vs E1C2 in two growth conditions.**
- **Supplementary Figure S8. Individual gene expression of the DNA damage iModulon.**
- **Supplementary Figure S9. Most extreme differences in iModulon activity between E1C2 and F1C2 strains across 11 growth conditions.**
- **Supplementary Figure S10. Expression values of the *sodA* and *fumC* genes in different strains or conditions.**
- **Supplementary Figure S11. Growth curves of W3110S in M9 glucose medium with various concentrations of H_2_O_2_.**
- **Supplementary Figure S12. Growth profiling of W3110S, DGF-C, F1C2, DGF-aldA, and F1C2-aldA in M9 glucose.**
- **Supplementary Figure S13. Differential iModulon analysis (DIMA) plot of MG1655 vs E1C2 in M9 glucose medium.**
- **Supplementary Figure S14. Differential iModulon analysis (DIMA) plot of DGF_aldA vs F1C2_aldA in M9 glucose medium.**
- **Supplementary Figure S15. Growth data for the DGF-C, DGF-aldA, F1C2, F1C2-aldA, and W3110S grown in MOPS + 0.2% glucose supplemented with different concentrations of H_2_O_2_**.


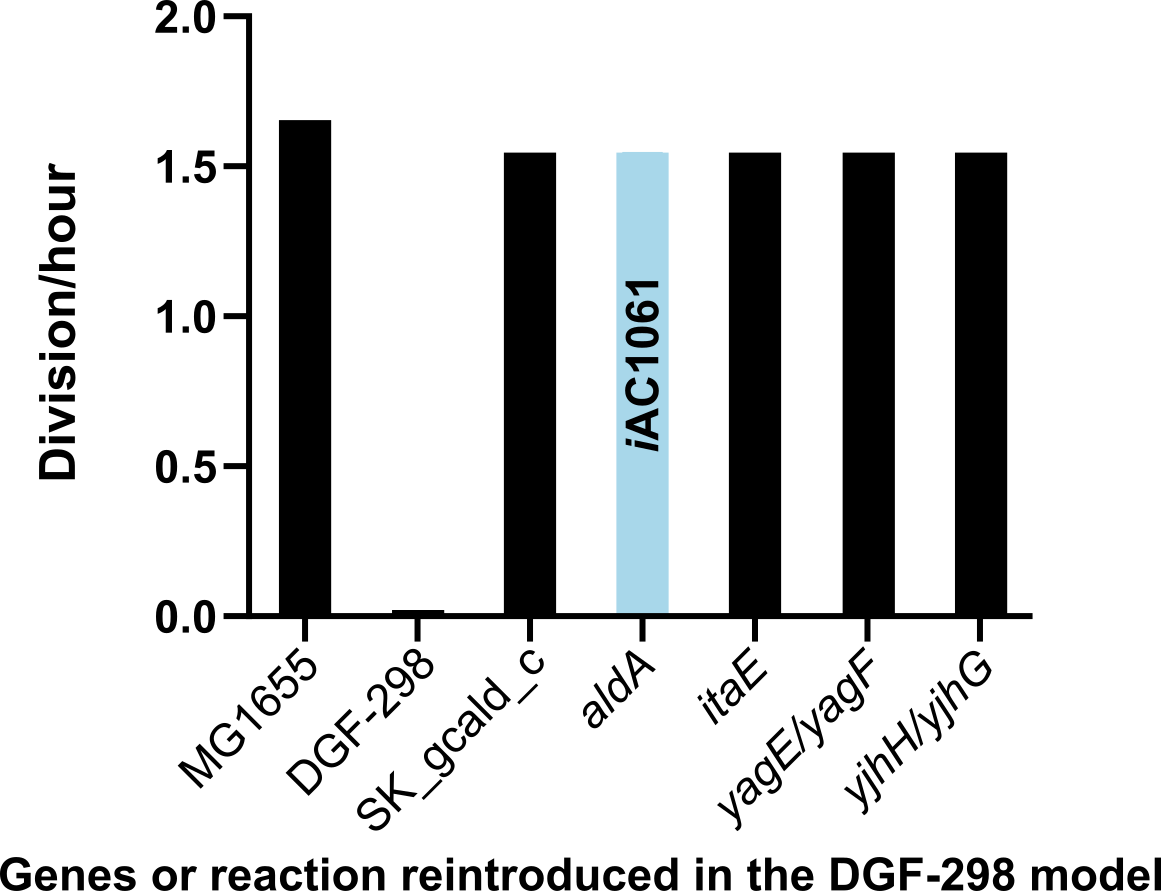


**Supplementary Figure S1. Simulated growth rate of *E. coli* MG1655 and the genome-reduced DGF-298 strain complemented with different genes or metabolic reactions to compensate for the accumulation of glycolaldehyde.** EZ-Rich glucose medium was used as the *in silico* medium in all simulated conditions. SK_gcald_c: pseudo-reaction freely consuming glycolaldehyde. Light blue color indicates the *i*AC1061 model.


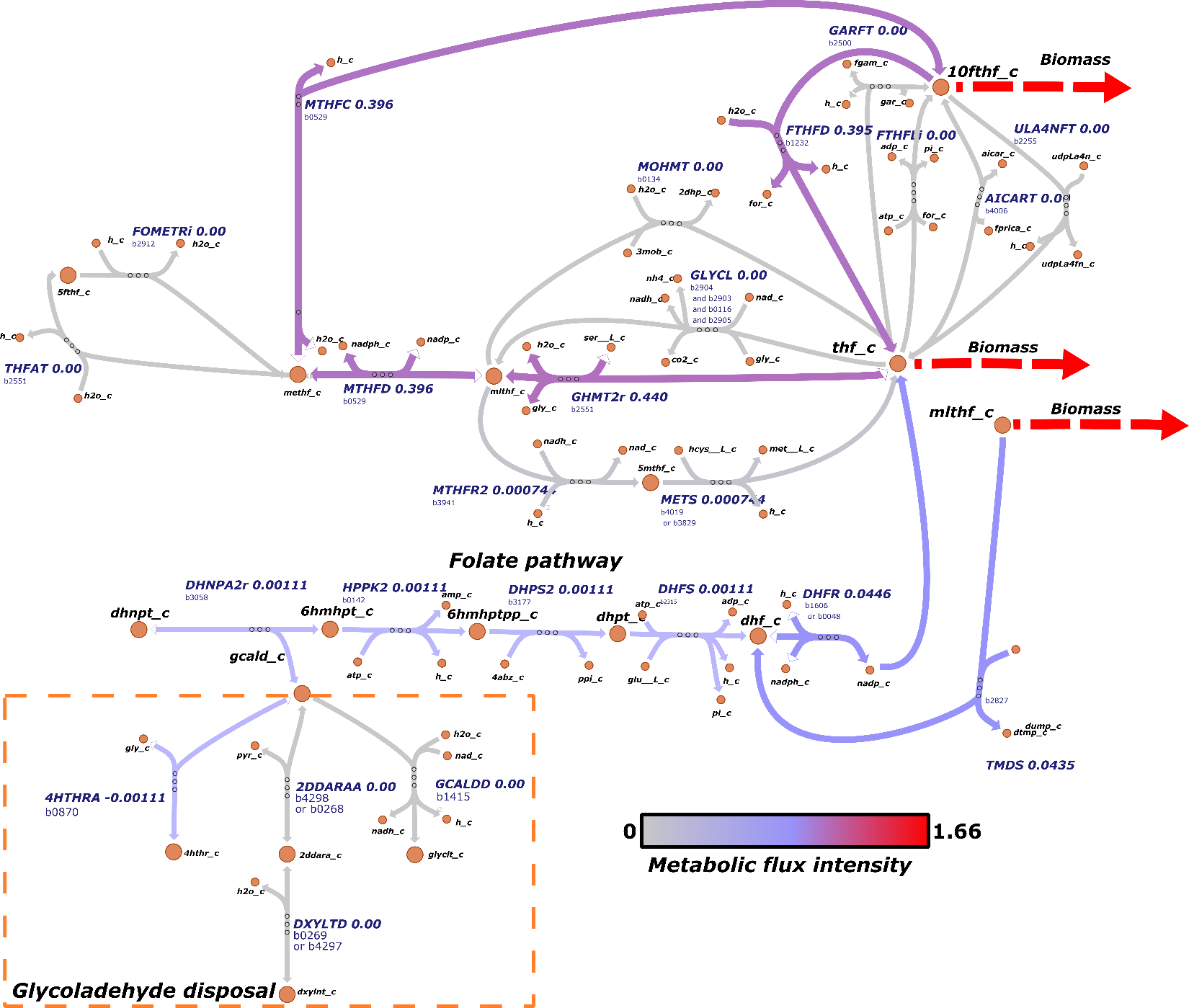
 **Supplementary Figure S2. Metabolic map and predicted fluxes of folate synthesis in *E. coli* MG1655.** EZ-Rich was used as the *in silico* medium. White arrowheads highlight unfavored directions in bidirectional reactions. Metabolite names are shown in black, while reaction and associated gene names are displayed in blue. For complete metabolite names, refer to the [BiGG Database](http://bigg.ucsd.edu/).


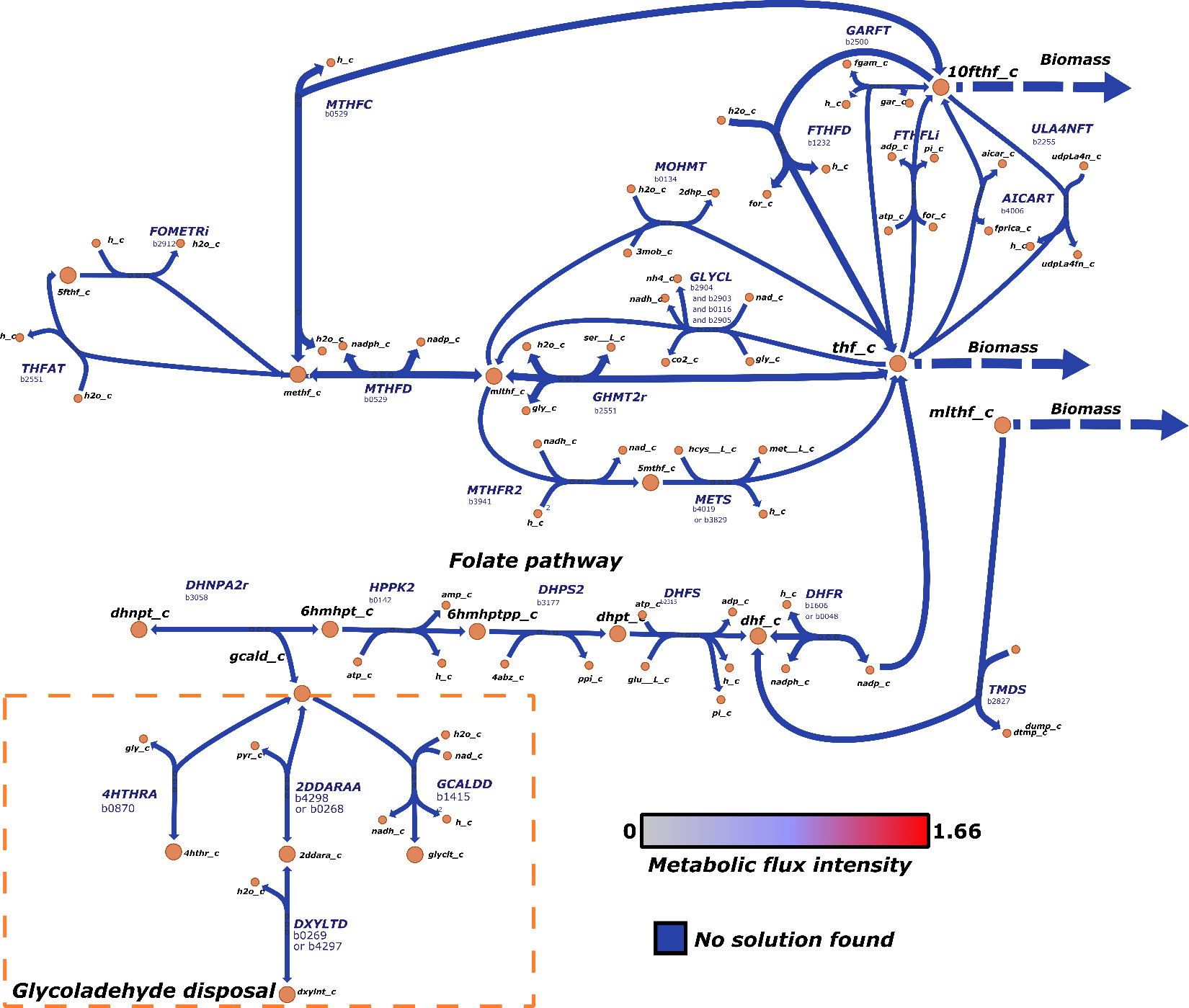


**Supplementary Figure S3. Metabolic map and predicted fluxes of folate synthesis in DGF-298.** EZ-Rich was used as the *in silico* medium. White arrowheads highlight unfavored directions in bidirectional reactions. Metabolite names are shown in black, while reaction and associated gene names are displayed in blue. For complete metabolite names, refer to the [BiGG Database](http://bigg.ucsd.edu/). Dark blue arrows indicate that no viable solution could be found.


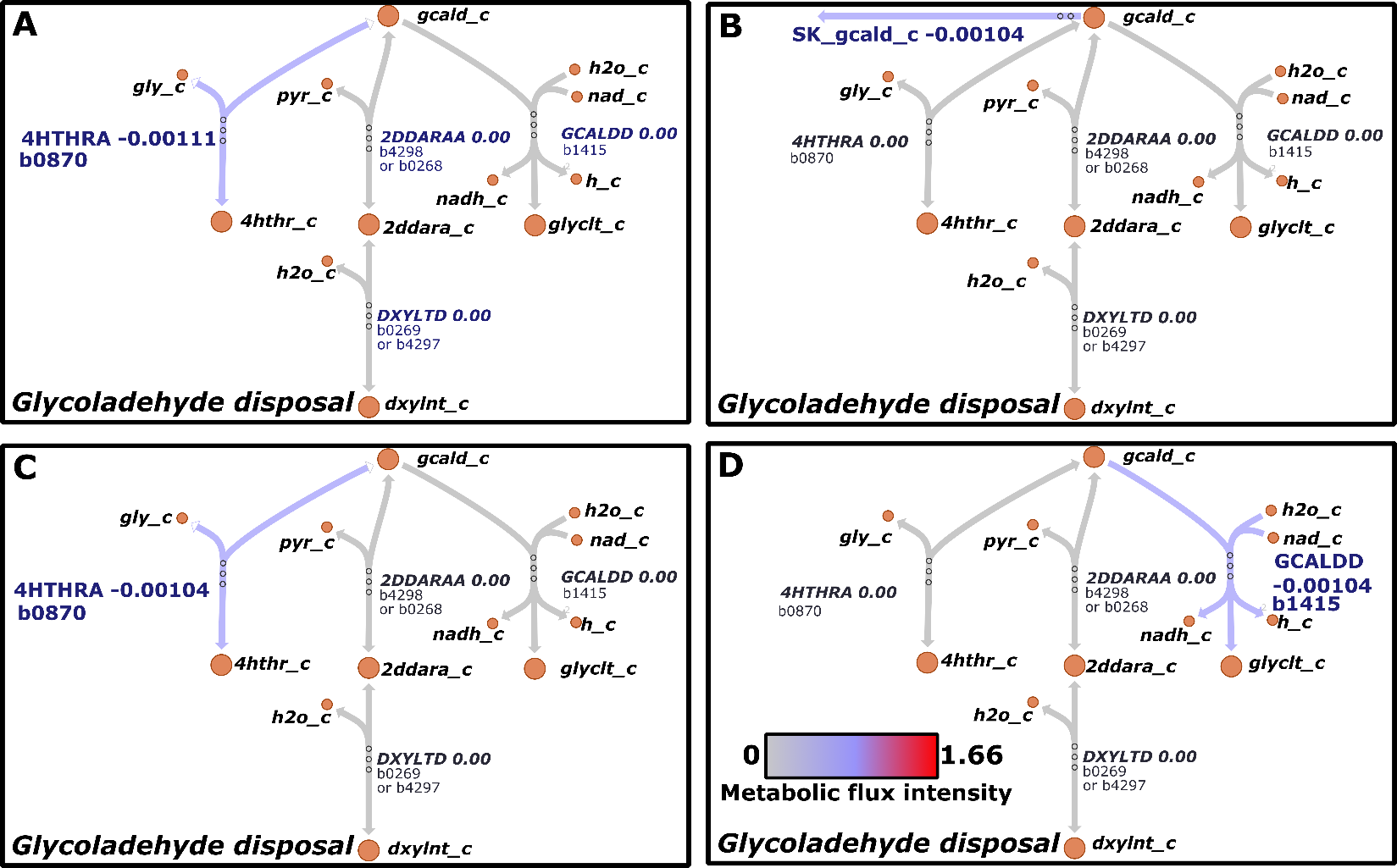
 **Supplementary Figure S4. Metabolic map and predicted fluxes of glycolaldehyde disposal in DGF-298 upon different gene/reaction complementations.** **A.** Glycolaldehyde disposal in wild-type *E. coli* MG1655. **B.** DGF-298 glycolaldehyde disposal upon introduction of sink reaction (SK_gcald_c). **C.** DGF-298 glycolaldehyde disposal upon *in silico* reintroduction of *ltaE*. **D.** DGF-298 glycolaldehyde disposal upon *in silico* reintroduction of *aldA*. EZ-Rich was used as the *in silico* medium. White arrowheads highlight unfavored directions in bidirectional reactions. For complete metabolite names, refer to the [BiGG Database](http://bigg.ucsd.edu/).


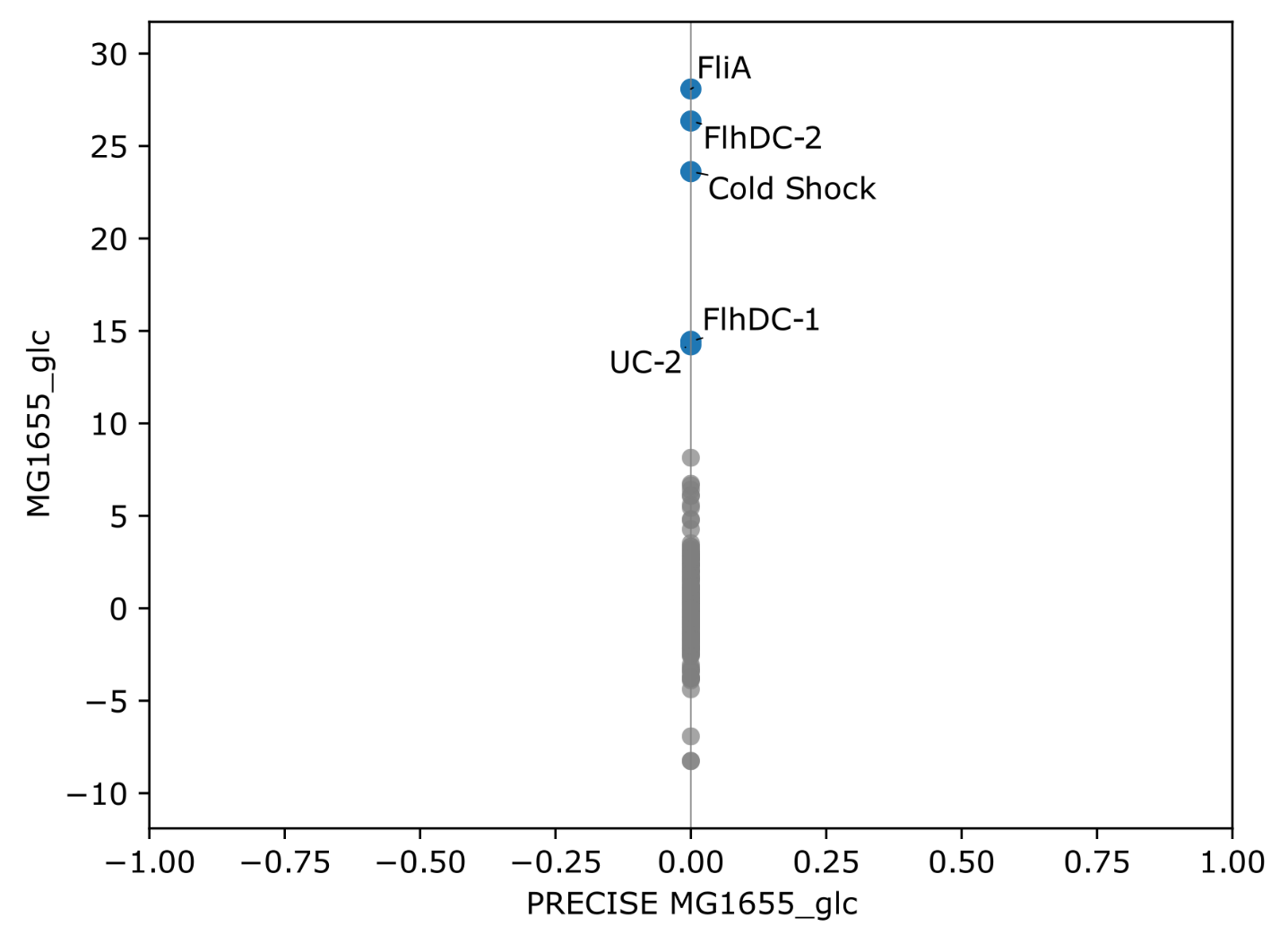


|  | difference | p-value | q-value | PRECISE MG1655_glc | MG1655_glc |
| --- | --- | --- | --- | --- | --- |
| FliA | 28.08 | 0.000113 | 0.007558 | 0 | 28.08 |
| FlhDC-2 | 26.35 | 0.000544 | 0.019172 | 0 | 26.35 |
| Cold Shock | 23.61 | 6.92E-05 | 0.006953 | 0 | 23.61 |
| FlhDC-1 | 14.44 | 2.62E-05 | 0.005272 | 0 | 14.44 |
| UC-2 | 14.25 | 0.000572 | 0.019172 | 0 | 14.25 |

**Supplementary Figure S5. Differential iModulon analysis (DIMA) plot of MG1655 from PRECISE database vs MG1655 in our dataset.** PRECISE_glc is the base activity of MG1655 from the PRECISE database, whereas MG1655_glc is the control experiment in our study. iModulons with differential activity >10 are highlighted in blue and listed below the graph, along with their associated activities and significance values. Cutoff is at 10 units of differential activity.


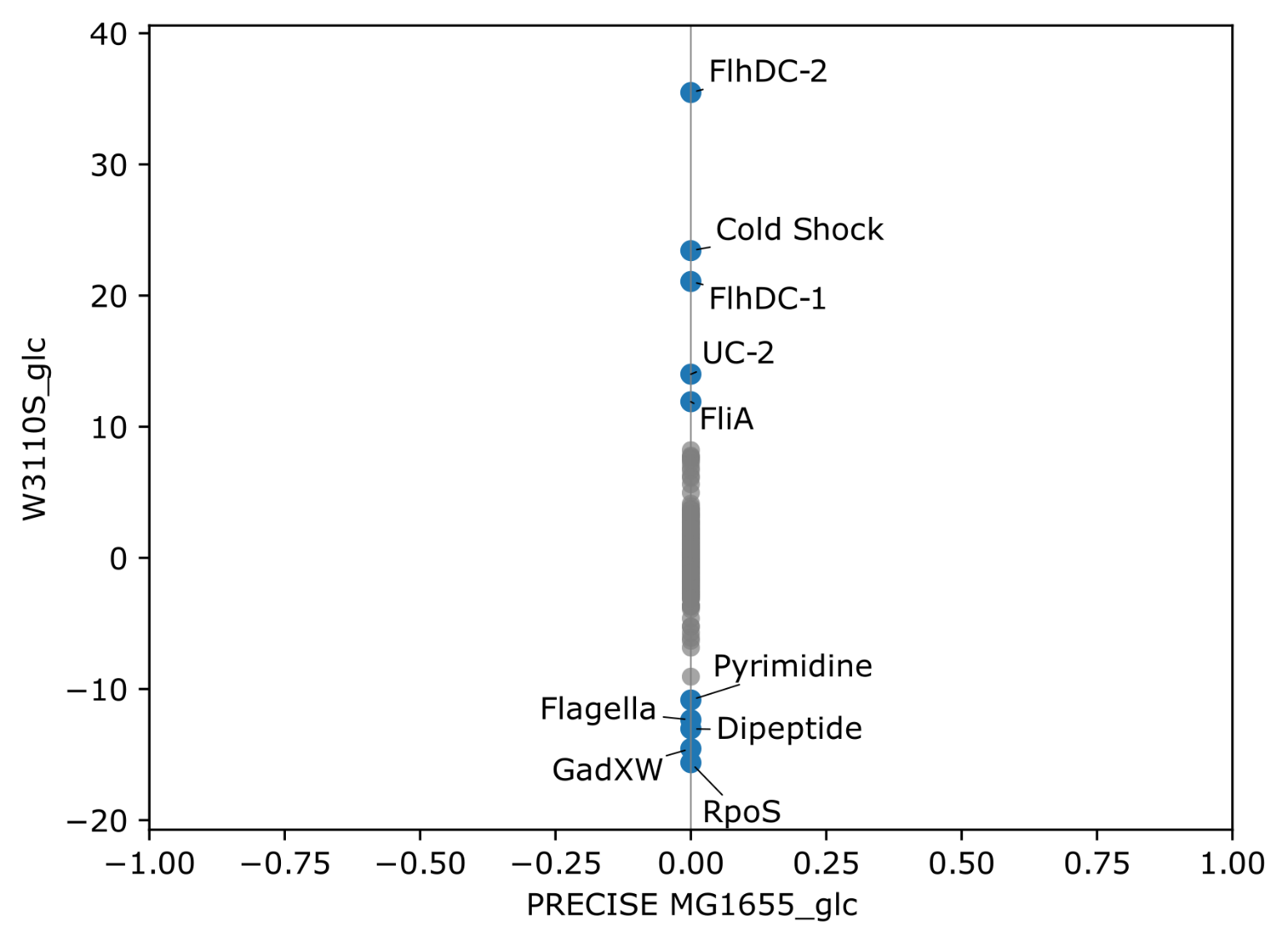


|  | difference | p-value | q-value | PRECISE MG1655_glc | W3110S_glc |
| --- | --- | --- | --- | --- | --- |
| **FlhDC-2** | **35.48** | **0.000227** | **0.007619** | **0** | **35.48** |
| **Cold Shock** | **23.42** | **7.16E-05** | **0.004799** | **0** | **23.42** |
| **FlhDC-1** | **21.07** | **3.78E-06** | **0.000759** | **0** | **21.07** |
| **UC-2** | **14** | **0.000606** | **0.013305** | **0** | **14** |
| **FliA** | **11.91** | **0.001462** | **0.024204** | **0** | **11.91** |
| Pyrimidine | -10.83 | 0.001763 | 0.024204 | 0 | -10.83 |
| Flagella | -12.35 | 2.13E-05 | 0.002136 | 0 | -12.35 |
| Dipeptide | -13.03 | 0.000111 | 0.005047 | 0 | -13.03 |
| GadXW | -14.54 | 0.000474 | 0.011919 | 0 | -14.54 |
| RpoS | -15.62 | 0.003649 | 0.031579 | 0 | -15.62 |

**Supplementary Figure S6. Differential iModulon analysis (DIMA) plot of MG1655 from PRECISE database vs W3110S in our dataset.** PRECISE_glc is the base activity from MG1655 from the PRECISE database, whereas W3110S_glc is the W3110S control experiment in our study. iModulons with differential activity >10 are highlighted in blue and listed below the graph, along with their associated activity and significance values. Cutoff is at 10 units of differential activity.


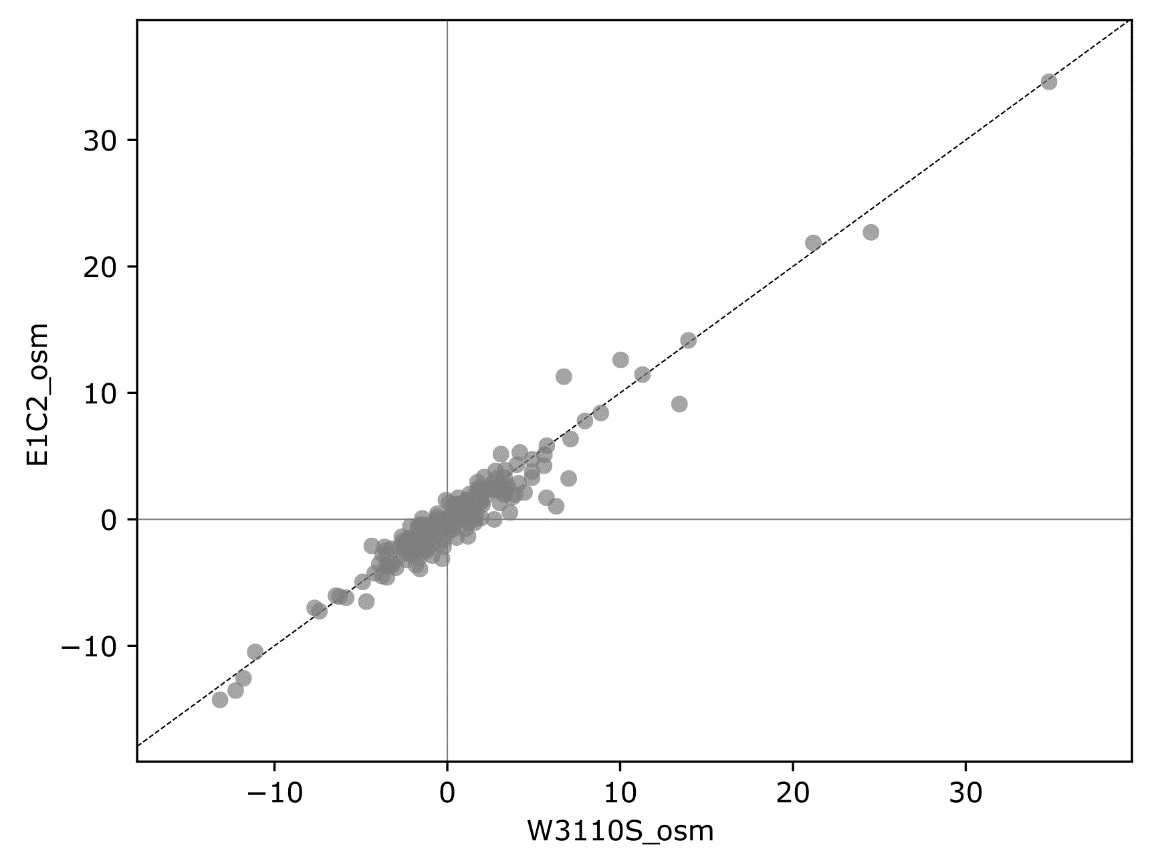

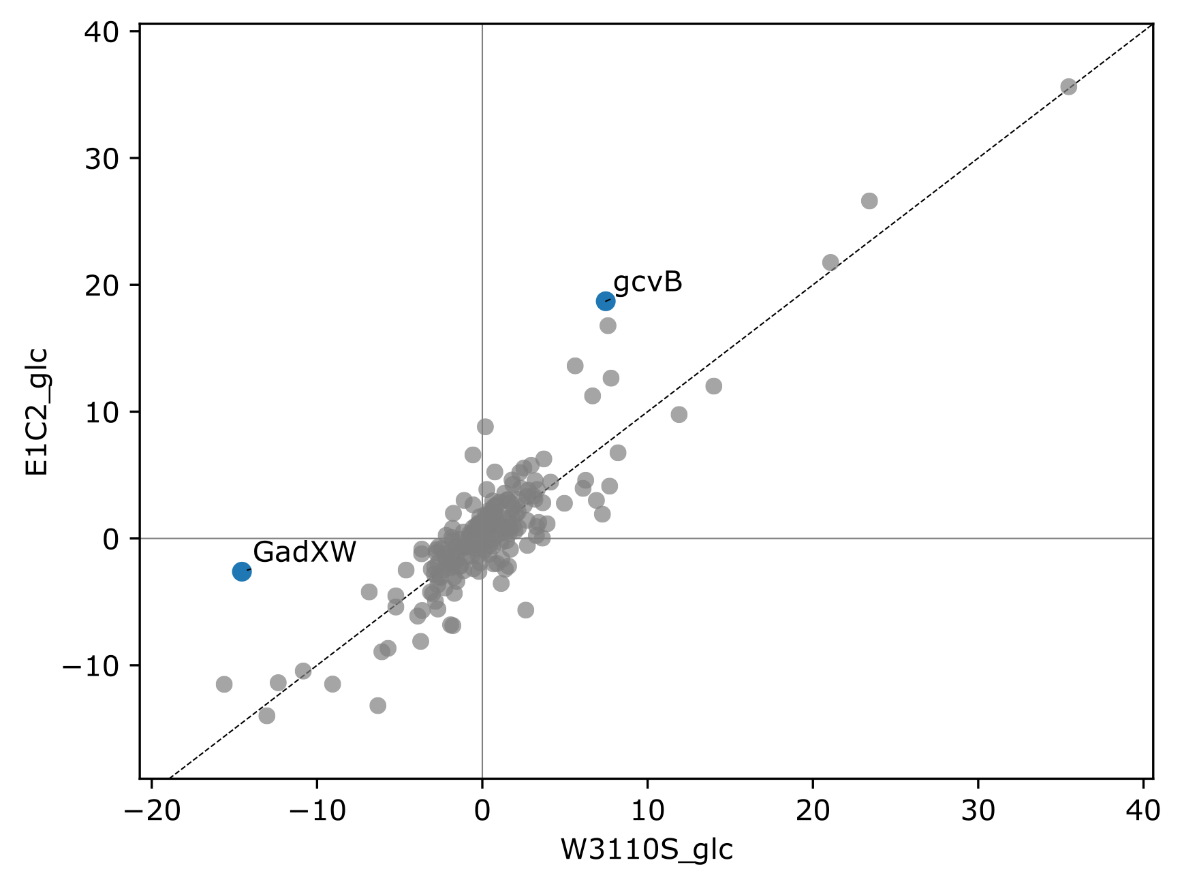


**A**

|  | difference | p-value | q-value | W3110S_glc | E1C2_glc |  |
| --- | --- | --- | --- | --- | --- | --- |
| **GadXW** | **11.92** | **0.000889** | **0.057037** | **-14.54** | **-2.62** |  |
| gcvB | 11.24 | 0.008313 | 0.092906 | 7.47 | 18.71 |  |

**Supplementary Figure S7. Differential iModulon analysis (DIMA) plot of W3110S vs E1C2 in two growth conditions. A.** Comparison in M9 glucose. **B.** Comparison in M9 glucose with osmotic stress. Cutoff is at 10 units of differential activity. iModulons with differential activity >10 are highlighted in blue and listed below the graph, along with their associated activity and significance values. Dashed lines show equivalent activity levels.

**B**


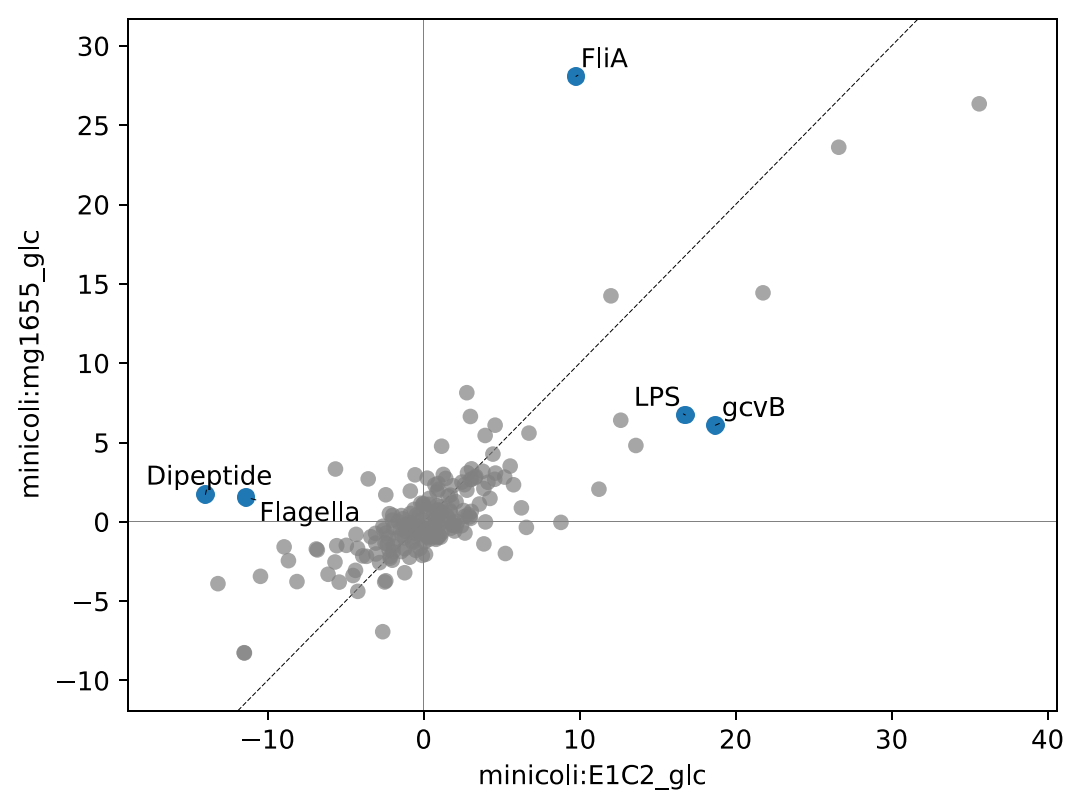


|  | difference | p-value | q-value | E1C2_glc | MG1655_glc |
| --- | --- | --- | --- | --- | --- |
| FliA | 18.31 | 0.000429 | 0.01490 | 9.770 | 28.08 |
| Dipeptide | 15.71 | 0.000052 | 0.005243 | -13.98 | 1.725 |
| Flagella | 12.92 | 0.000017 | 0.003367 | -11.38 | 1.548 |
| LPS | -10.04 | 0.02343 | 0.08120 | 16.78 | 6.733 |
| gcvB | -12.62 | 0.006180 | 0.05176 | 18.71 | 6.089 |

**Supplementary Figure S8. Differential iModulon analysis (DIMA) plot of MG1655 vs E1C2 in M9 glucose medium.**  Cutoff is at 10 units of differential activity. iModulons with differential activity >10 are highlighted in blue and listed below the graph, along with their associated activity and significance values. Dashed line shows equivalent activity levels.


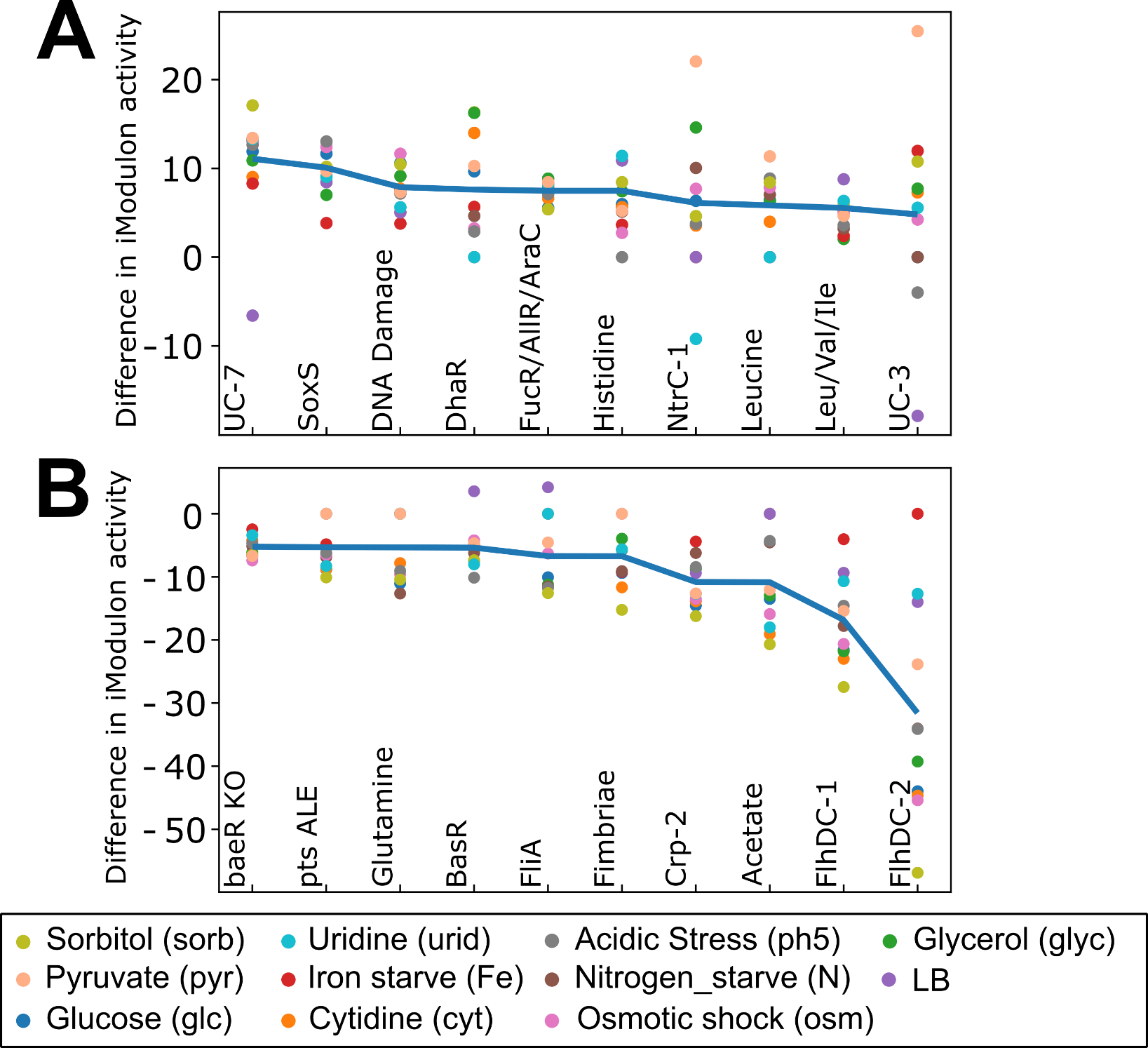


**Supplementary Figure S9. Most extreme differences in iModulon activity between E1C2 and F1C2 strains across 11 growth conditions.** Ten most positively (**A**) and most negatively (**B**) differentially activated iModulons between E1C2 and F1C2.


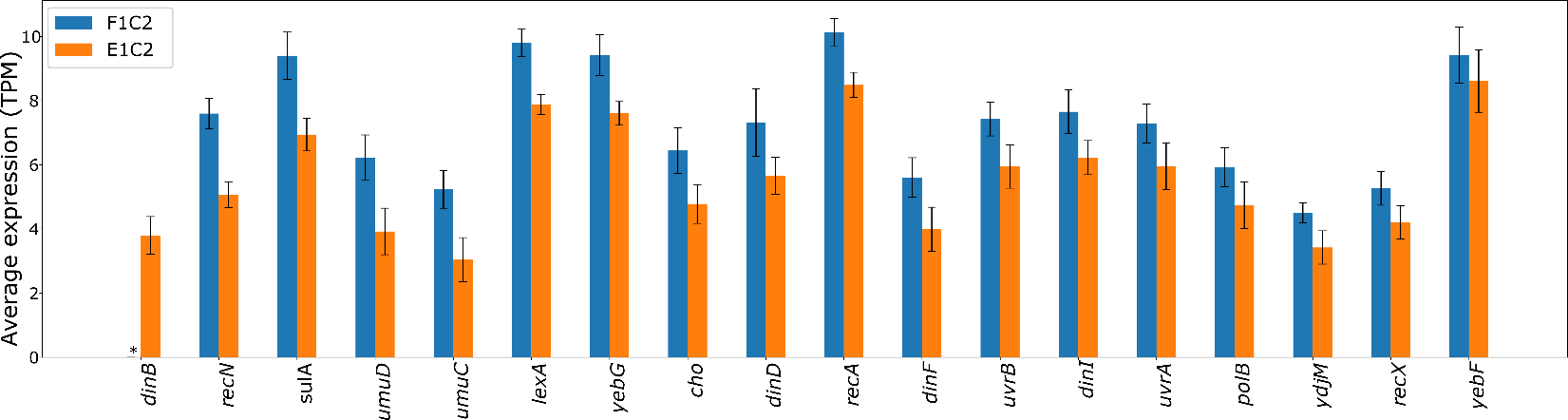


**Supplementary Figure S10. Individual gene expression of the DNA damage iModulon.** Average expression of the differently expressed genes of the DNA damage iModulon between E1C2 (orange) and F1C2 (blue). Gene expression is given as the transcripts per million mapped reads (TPM). Error bars indicate standard deviation calculated over eleven conditions. Star indicates gene deletion in F1C2.

**Supplementary Figure S11. Expression values of the *sodA* and *fumC* genes in different strains or conditions.** Gene expression is given as the number of reads per kilobase of transcript per million mapped reads (RPKM). Base medium is M9 0.2% glucose.


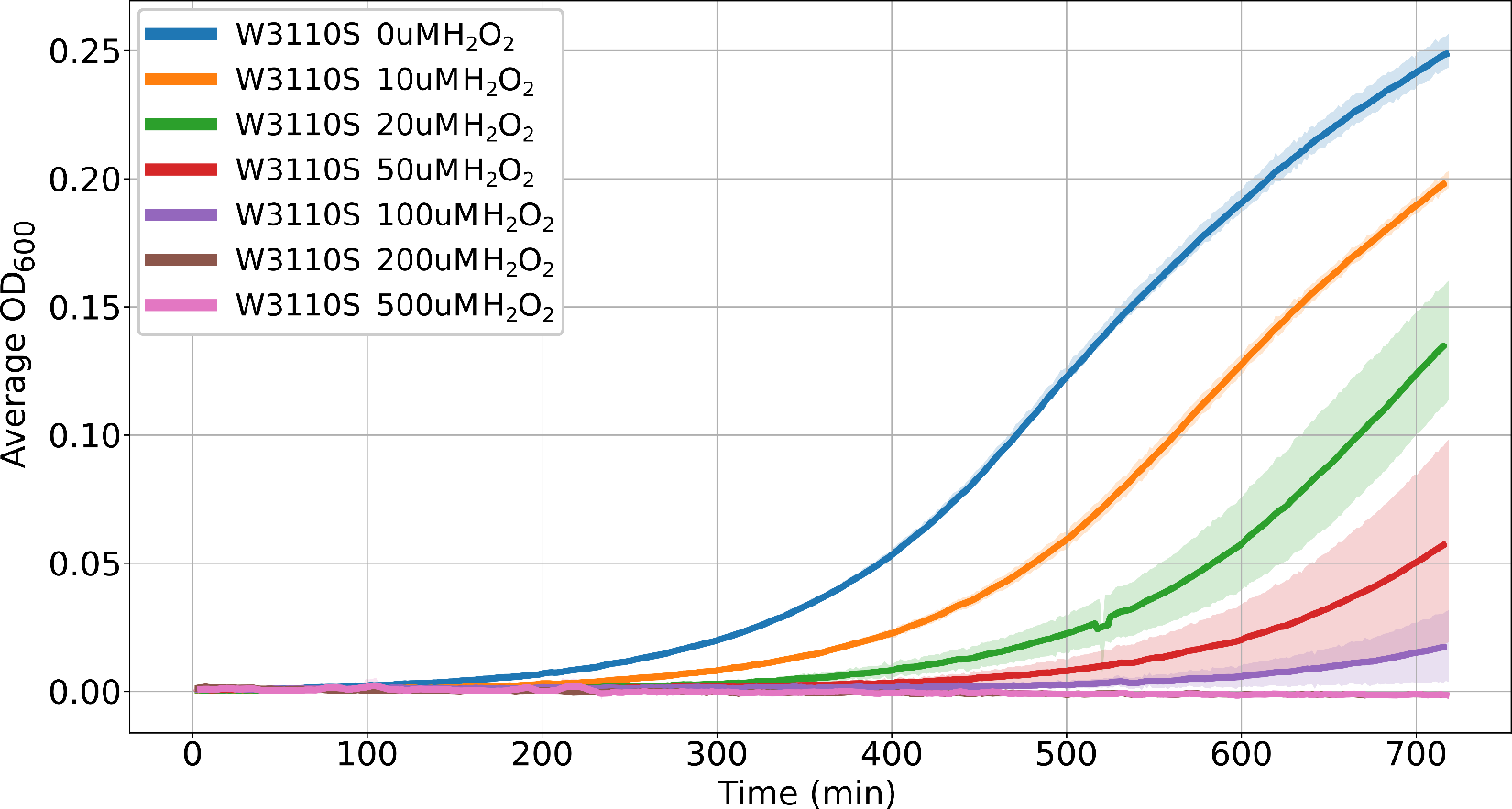


**Supplementary Figure S12. Growth curves of W3110S in M9 glucose medium with various concentrations of H_2_O_2_.** Average of triplicates. Standard deviations are represented by colored shadings.


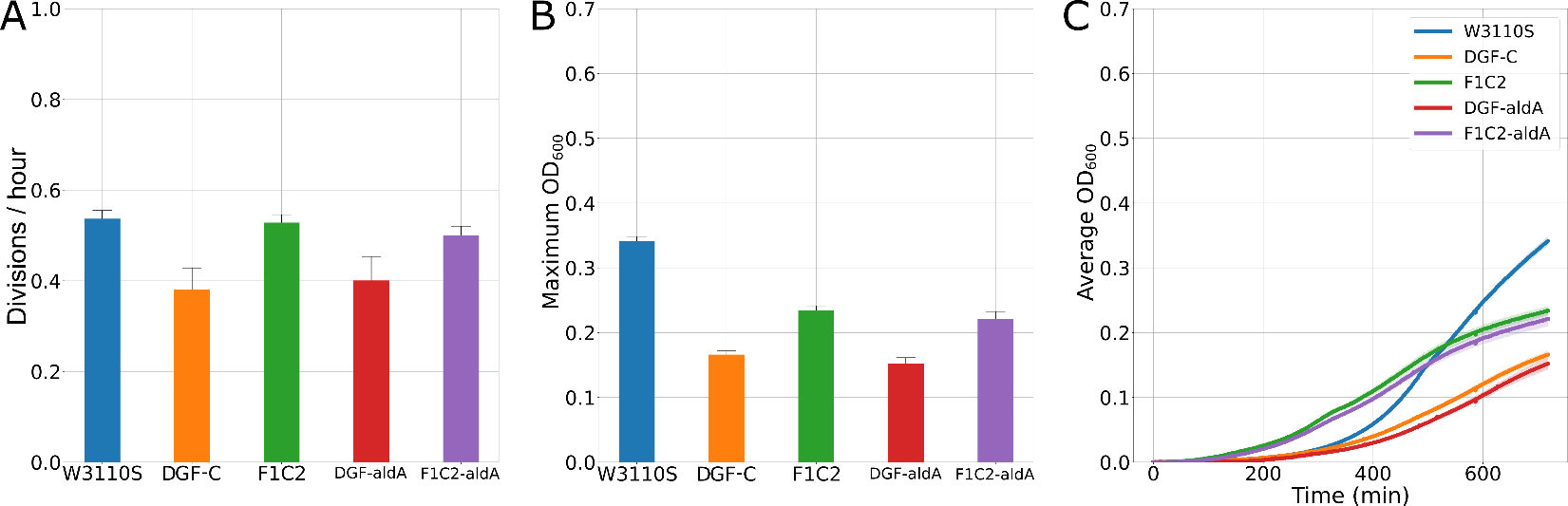


**Supplementary Figure S13 Growth profiling of W3110S, DGF-C, F1C2, DGF-aldA, and F1C2-aldA in M9 glucose. A & B :** Average of six replicates, errors bars represents standart deviation. **C.** Average of six replicates. Standard deviations are represented by colored shadings.

|  | | difference | | pvalue | | qvalue | | minE_aldA | | F1C2_aldA | |
| --- | --- | --- | --- | --- | --- | --- | --- | --- | --- | --- | --- |
| Translation | | -21.8 | | 0.001453948 | | 0.03836003 | | -3.91 | | -25.72 | |
|  |  | |  | |  | |  | |  | |  |


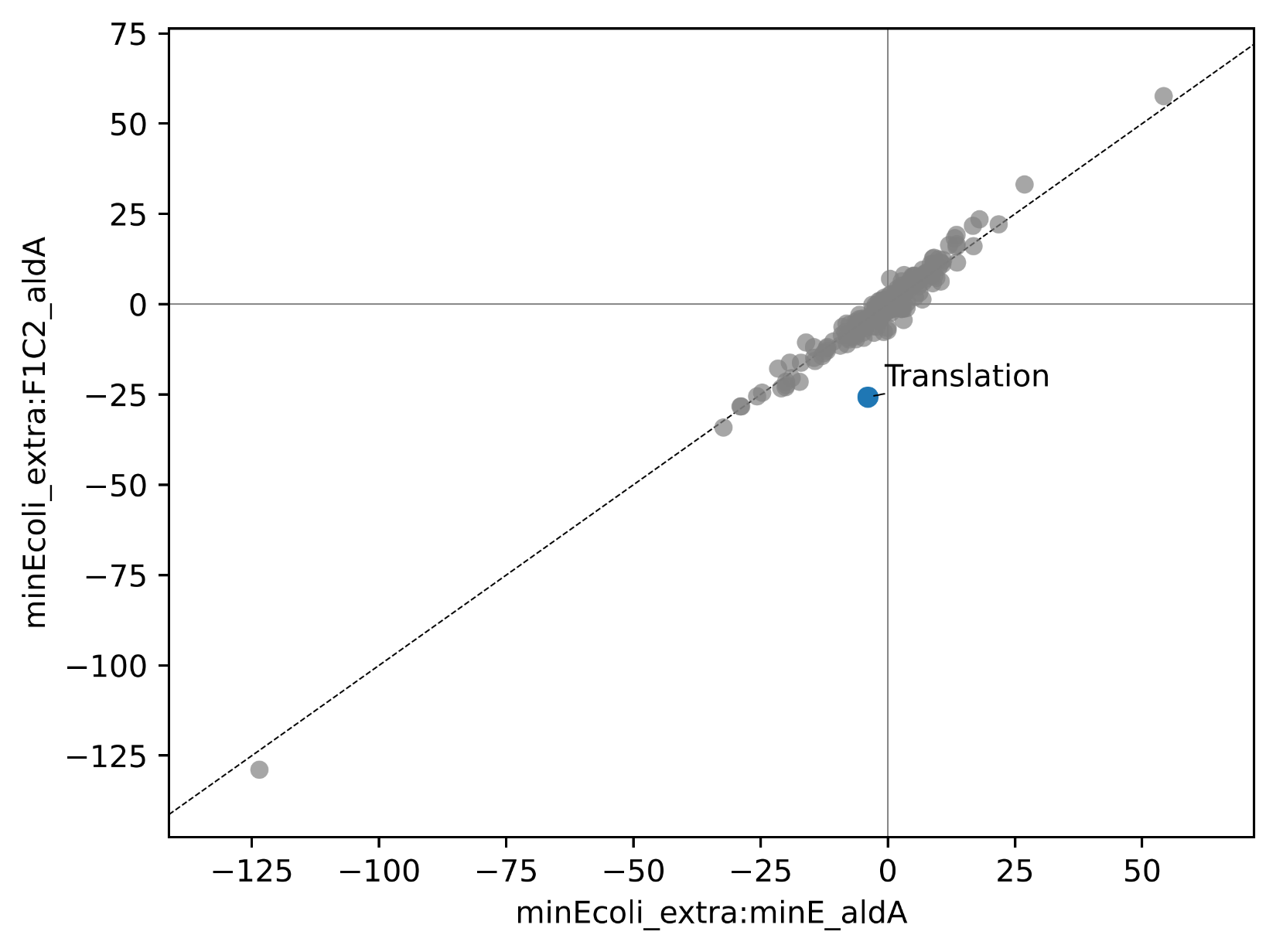
**Supplementary Figure S14. Differential iModulon analysis (DIMA) plot of DGF_aldA vs F1C2_aldA in M9 glucose medium.**  Cutoff is at 10 units of differential activity. iModulons with differential activity >10 are highlighted in blue and listed below the graph, along with their associated activity and significance values. Dashed line shows equivalent activity levels.


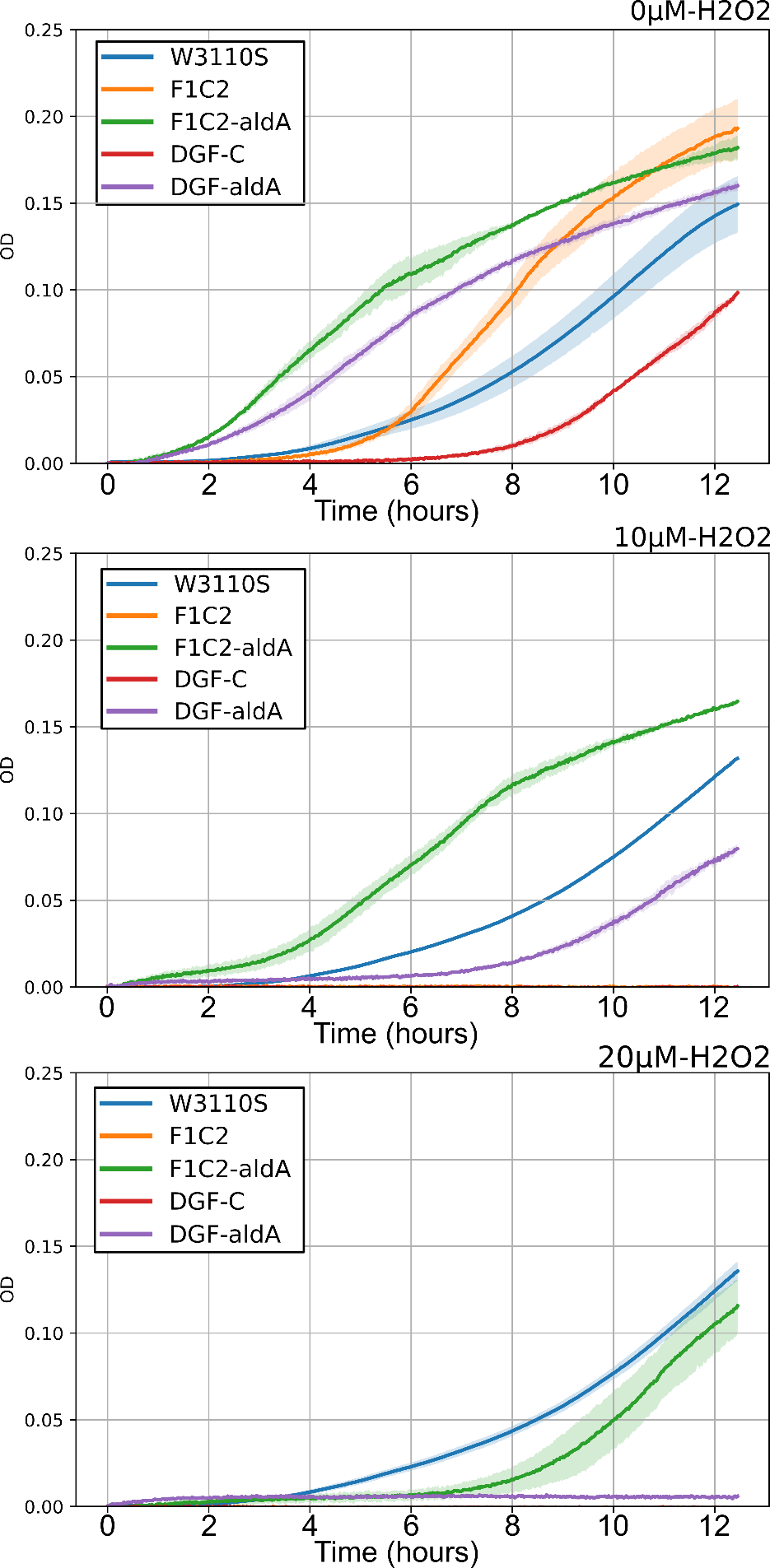


**Supplementary Figure S15. Growth data for the DGF-C, DGF-aldA, F1C2, F1C2-aldA, and W3110S grown in MOPS + 0.2% glucose supplemented with different concentrations of H_2_O_2_**. Each data point represents the average optical density at 600nm (OD_600_) of two technical replicates grown at 30°C with agitation in a plate reader over 12h. Coloured shadings represent standard deviations.
