# Supplementary material for: Diagnosis and mitigation of the systemic impact of genome reduction in Escherichia coli DGF-298
Source: mBio. 2024 Aug 29;15(10):e00873-24. doi: 10.1128/mbio.00873-24 (PMC11481515; doi:10.1128/mbio.00873-24)
Supplement: File S1, part 1 — All i-modulon activities, part 1. [file mbio.00873-24-s0003.pdf]

Diagnosis and Mitigation of the Systemic Impact of Extensive Genome Reduction in *Escherichia coli* DGF-298

Antoine Champie[1], Jean-Christophe Lachance[1], Anand Sastry[2], Dominick Matteau[1], Colton J. Lloyd[2], Frédéric Grenier[1], Cameron R. Lamoureux[2], Simon Jeanneau[1], Adam M. Feist[2,5], Pierre-Étienne Jacques[1], Bernhard O. Palsson[2,3,4,5] & Sébastien Rodrigue[1†]

SUPPLEMENTARY FILE S1_2 : All iModulons activities

Activity level off all iModulons in the conditions tested. For color disambiguation conditions in the graphs are in the same order as the legend on the right.

Name of the groups of conditions :

- control = MG1655 in M9 glucose medium.
- ica = Diverse control conditions from the precise 1k database.
- miniEcoli_extra = Additional oxidative stress conditions on the reduced strains.
- miniEcoli = Main conditions tested in this article.
- oxidative = Oxidative stress conditions from the precise 1k database.

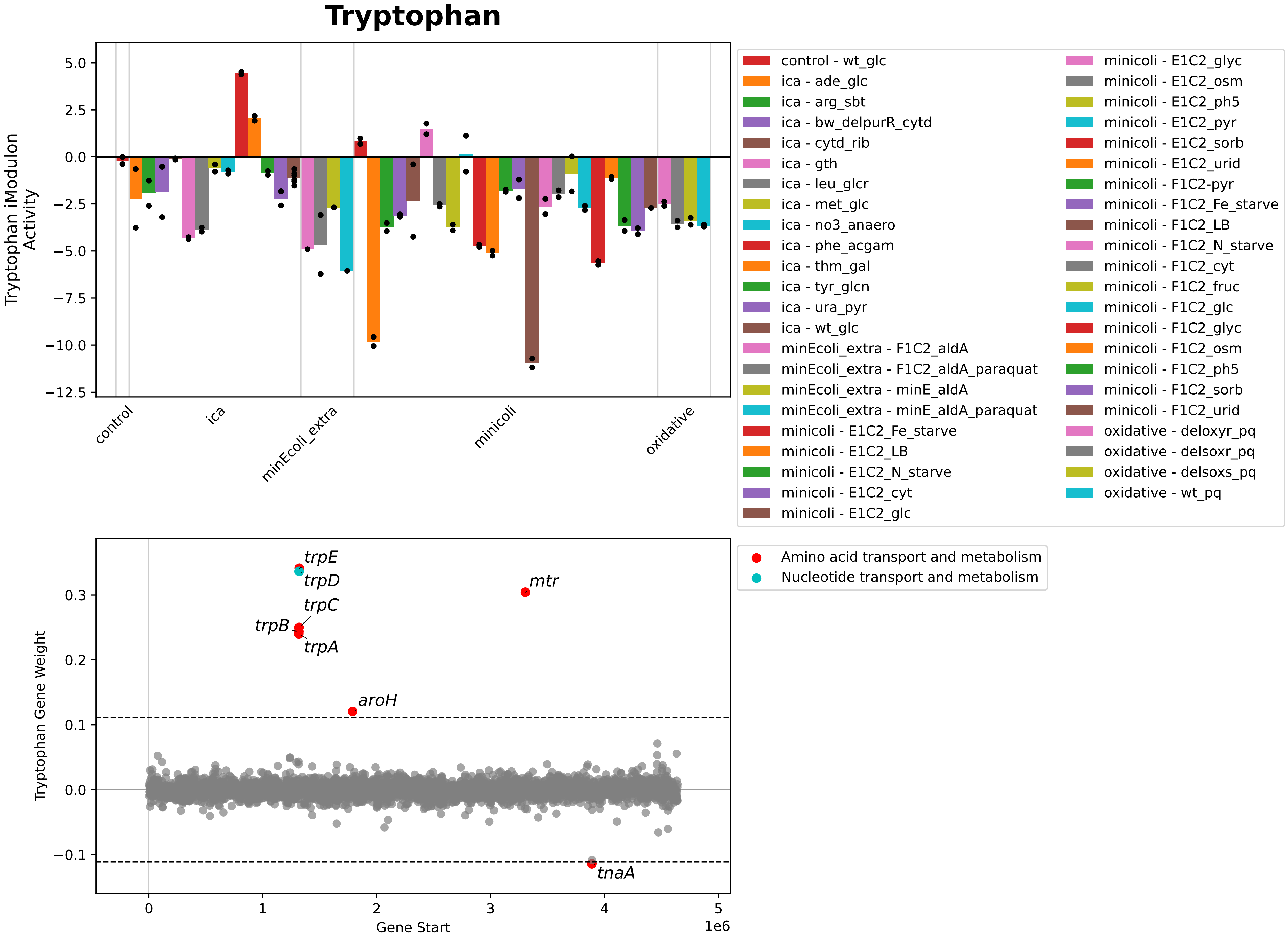

Tryptophan
Tryptophan iModulon Activity
5.0
2.5
0.0
−2.5
−5.0
−7.5
−10.0
−12.5
control
ica
minEcoli_extra
minicoli
oxidative
control - wt_glc
ica - ade_glc
ica - arg_sbt
ica - bw_delpurR_cytd
ica - cytd_rib
ica - gth
ica - leu_glcr
ica - met_glc
ica - no3_anaero
ica - phe_acgam
ica - thm_gal
ica - tyr_glcn
ica - ura_pyr
ica - wt_glc
minEcoli_extra - F1C2_aldA
minEcoli_extra - F1C2_aldA_paraquat
minEcoli_extra - minE_aldA
minEcoli_extra - minE_aldA_paraquat
minicoli - E1C2_Fe_starve
minicoli - E1C2_LB
minicoli - E1C2_N_starve
minicoli - E1C2_cyt
minicoli - E1C2_glc
minicoli - E1C2_glyc
minicoli - E1C2_osm
minicoli - E1C2_ph5
minicoli - E1C2_pyr
minicoli - E1C2_sorb
minicoli - E1C2_urid
minicoli - F1C2-pyr
minicoli - F1C2_Fe_starve
minicoli - F1C2_LB
minicoli - F1C2_N_starve
minicoli - F1C2_cyt
minicoli - F1C2_fruc
minicoli - F1C2_glc
minicoli - F1C2_glyc
minicoli - F1C2_osm
minicoli - F1C2_ph5
minicoli - F1C2_sorb
minicoli - F1C2_urid
oxidative - deloxyr_pq
oxidative - delsoxr_pq
oxidative - delsoxs_pq
oxidative - wt_pq
Tryptophan Gene Weight
0.3
0.2
0.1
0.0
−0.1
trpE
trpD
trpC
trpB
trpA
mtr
aroH
tnaA
0
1
2
3
4
5
1e6
Gene Start
Amino acid transport and metabolism
Nucleotide transport and metabolism

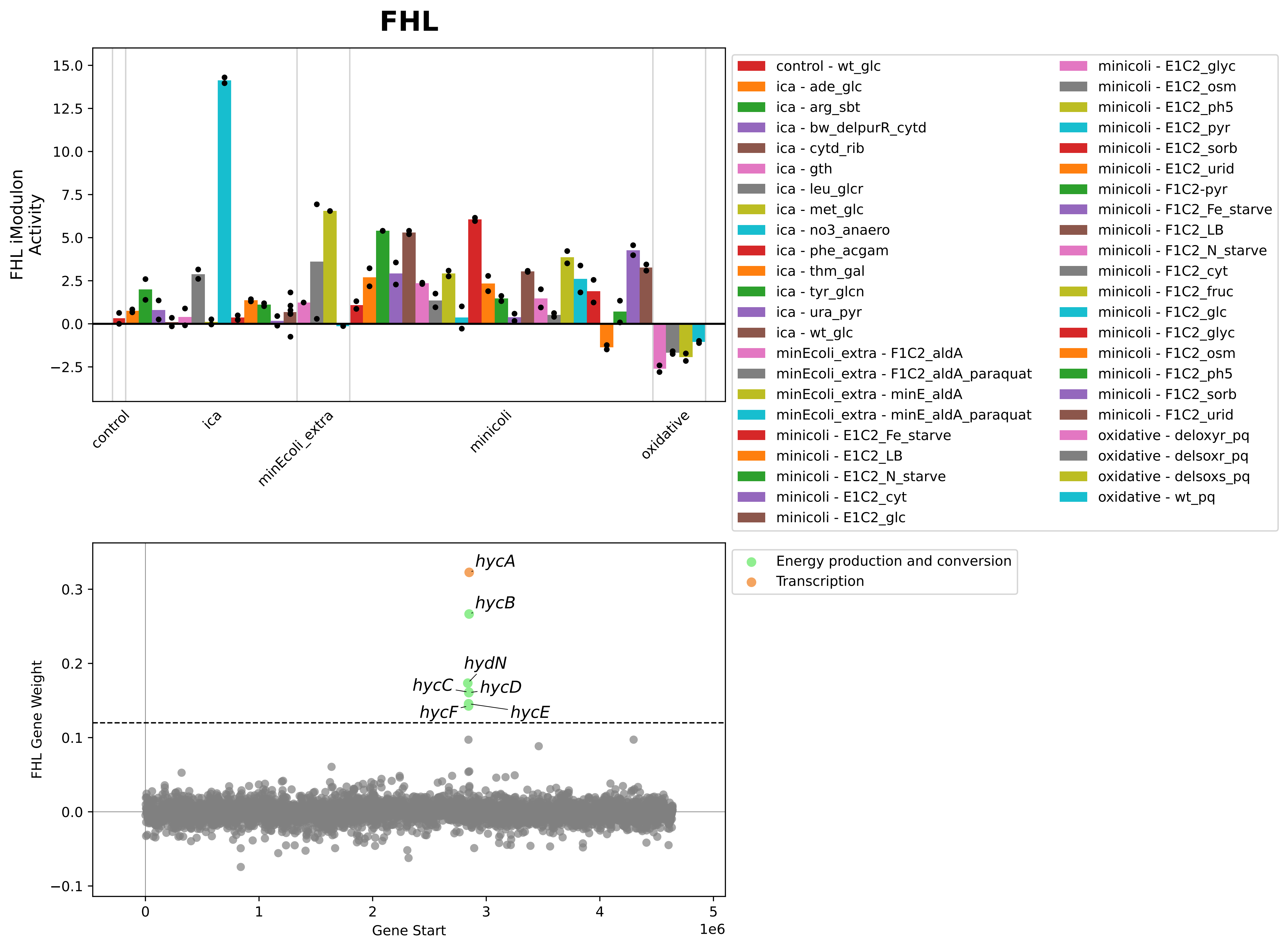

FHL
FHL iModulon Activity
control
ica
minEcoli_extra
minicoli
oxidative
control - wt_glc
ica - ade_glc
ica - arg_sbt
ica - bw_delpurR_cytd
ica - cytd_rib
ica - gth
ica - leu_glcr
ica - met_glc
ica - no3_anaero
ica - phe_acgam
ica - thm_gal
ica - tyr_glcn
ica - ura_pyr
ica - wt_glc
minEcoli_extra - F1C2_aldA
minEcoli_extra - F1C2_aldA_paraquat
minEcoli_extra - minE_aldA
minEcoli_extra - minE_aldA_paraquat
minicoli - E1C2_Fe_starve
minicoli - E1C2_LB
minicoli - E1C2_N_starve
minicoli - E1C2_cyt
minicoli - E1C2_glc
minicoli - E1C2_glyc
minicoli - E1C2_osm
minicoli - E1C2_ph5
minicoli - E1C2_pyr
minicoli - E1C2_sorb
minicoli - E1C2_urid
minicoli - F1C2-pyr
minicoli - F1C2_Fe_starve
minicoli - F1C2_LB
minicoli - F1C2_N_starve
minicoli - F1C2_cyt
minicoli - F1C2_fruc
minicoli - F1C2_glc
minicoli - F1C2_glyc
minicoli - F1C2_osm
minicoli - F1C2_ph5
minicoli - F1C2_sorb
minicoli - F1C2_urid
oxidative - deloxyr_pq
oxidative - delsoxr_pq
oxidative - delsoxs_pq
oxidative - wt_pq
FHL Gene Weight
Gene Start
1e6
hycA
hycB
hydN
hycC
hycD
hycF
hycE
Energy production and conversion
Transcription

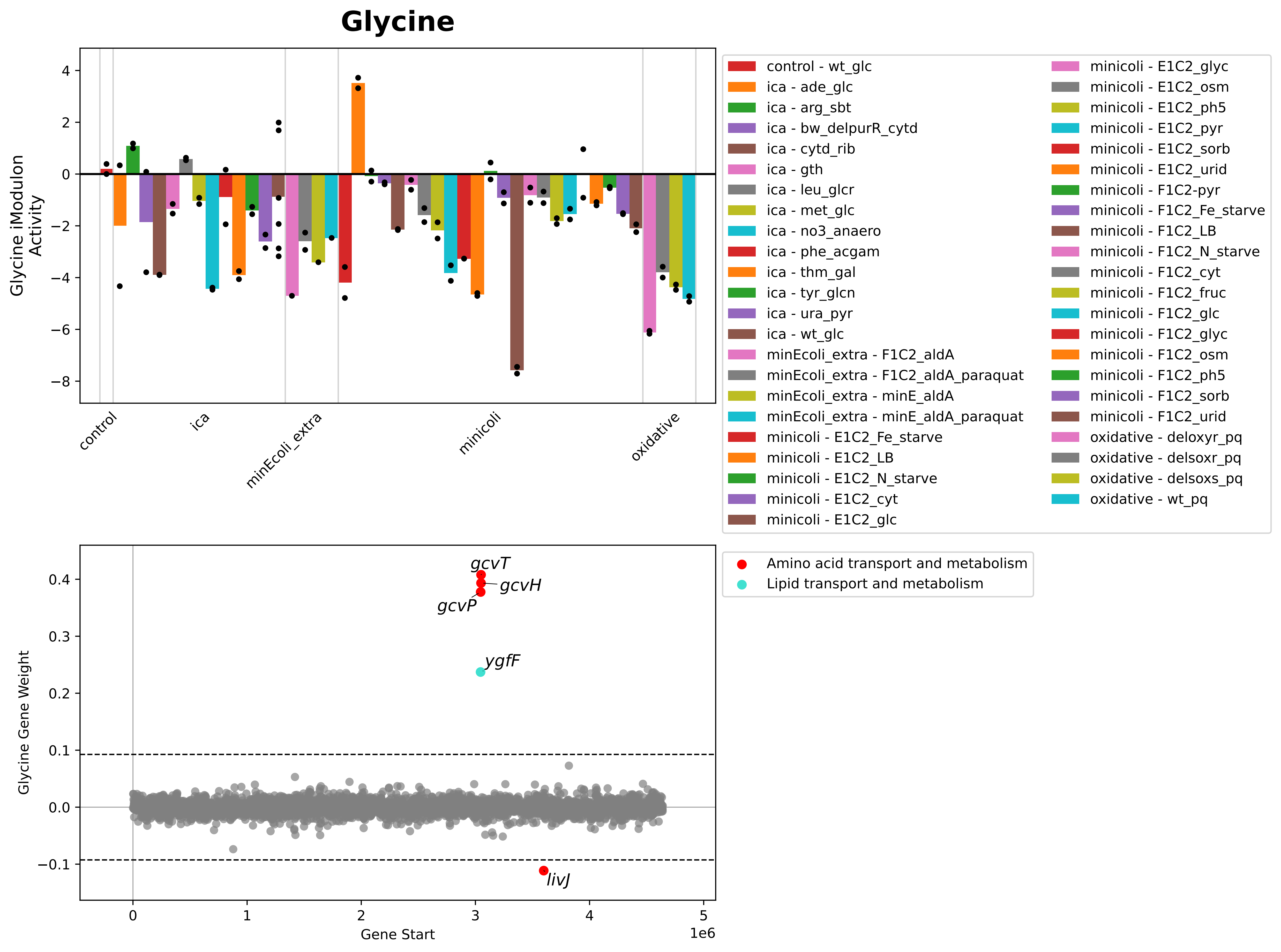

Glycine
Glycine iModulon Activity
control
ica
minEcoli_extra
minicoli
oxidative
control - wt_glc
ica - ade_glc
ica - arg_sbt
ica - bw_delpurR_cytd
ica - cytd_rib
ica - gth
ica - leu_glcr
ica - met_glc
ica - no3_anaero
ica - phe_acgam
ica - thm_gal
ica - tyr_glcn
ica - ura_pyr
ica - wt_glc
minEcoli_extra - F1C2_aldA
minEcoli_extra - F1C2_aldA_paraquat
minEcoli_extra - minE_aldA
minEcoli_extra - minE_aldA_paraquat
minicoli - E1C2_Fe_starve
minicoli - E1C2_LB
minicoli - E1C2_N_starve
minicoli - E1C2_cyt
minicoli - E1C2_glc
minicoli - E1C2_glyc
minicoli - E1C2_osm
minicoli - E1C2_ph5
minicoli - E1C2_pyr
minicoli - E1C2_sorb
minicoli - E1C2_urid
minicoli - F1C2-pyr
minicoli - F1C2_Fe_starve
minicoli - F1C2_LB
minicoli - F1C2_N_starve
minicoli - F1C2_cyt
minicoli - F1C2_fruc
minicoli - F1C2_glc
minicoli - F1C2_glyc
minicoli - F1C2_osm
minicoli - F1C2_ph5
minicoli - F1C2_sorb
minicoli - F1C2_urid
oxidative - deloxyr_pq
oxidative - delsoxr_pq
oxidative - delsoxs_pq
oxidative - wt_pq
Glycine Gene Weight
gcvT
gcvH
gcvP
ygfF
livJ
Gene Start
1e6
Amino acid transport and metabolism
Lipid transport and metabolism

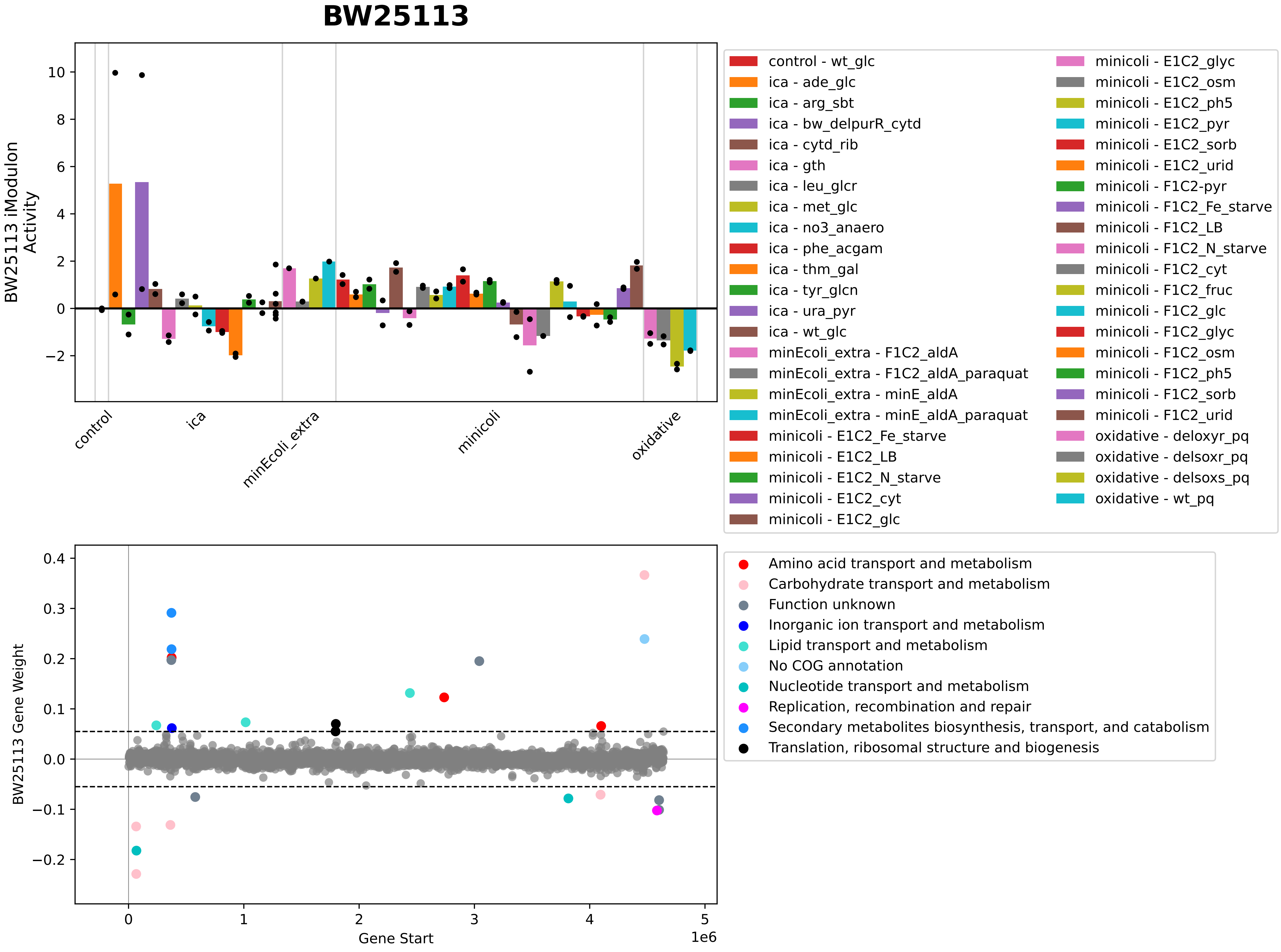

BW25113
BW25113 iModulon Activity
control
ica
minEcoli_extra
minicoli
oxidative
control - wt_glc
ica - ade_glc
ica - arg_sbt
ica - bw_delpurR_cytd
ica - cytd_rib
ica - gth
ica - leu_glcr
ica - met_glc
ica - no3_anaero
ica - phe_acgam
ica - thm_gal
ica - tyr_glcn
ica - ura_pyr
ica - wt_glc
minEcoli_extra - F1C2_aldA
minEcoli_extra - F1C2_aldA_paraquat
minEcoli_extra - minE_aldA
minEcoli_extra - minE_aldA_paraquat
minicoli - E1C2_Fe_starve
minicoli - E1C2_LB
minicoli - E1C2_N_starve
minicoli - E1C2_cyt
minicoli - E1C2_glc
minicoli - E1C2_glyc
minicoli - E1C2_osm
minicoli - E1C2_ph5
minicoli - E1C2_pyr
minicoli - E1C2_sorb
minicoli - E1C2_urid
minicoli - F1C2-pyr
minicoli - F1C2_Fe_starve
minicoli - F1C2_LB
minicoli - F1C2_N_starve
minicoli - F1C2_cyt
minicoli - F1C2_fruc
minicoli - F1C2_glc
minicoli - F1C2_glyc
minicoli - F1C2_osm
minicoli - F1C2_ph5
minicoli - F1C2_sorb
minicoli - F1C2_urid
oxidative - deloxyr_pq
oxidative - delsoxr_pq
oxidative - delsoxs_pq
oxidative - wt_pq
BW25113 Gene Weight
Gene Start
1e6
Amino acid transport and metabolism
Carbohydrate transport and metabolism
Function unknown
Inorganic ion transport and metabolism
Lipid transport and metabolism
No COG annotation
Nucleotide transport and metabolism
Replication, recombination and repair
Secondary metabolites biosynthesis, transport, and catabolism
Translation, ribosomal structure and biogenesis

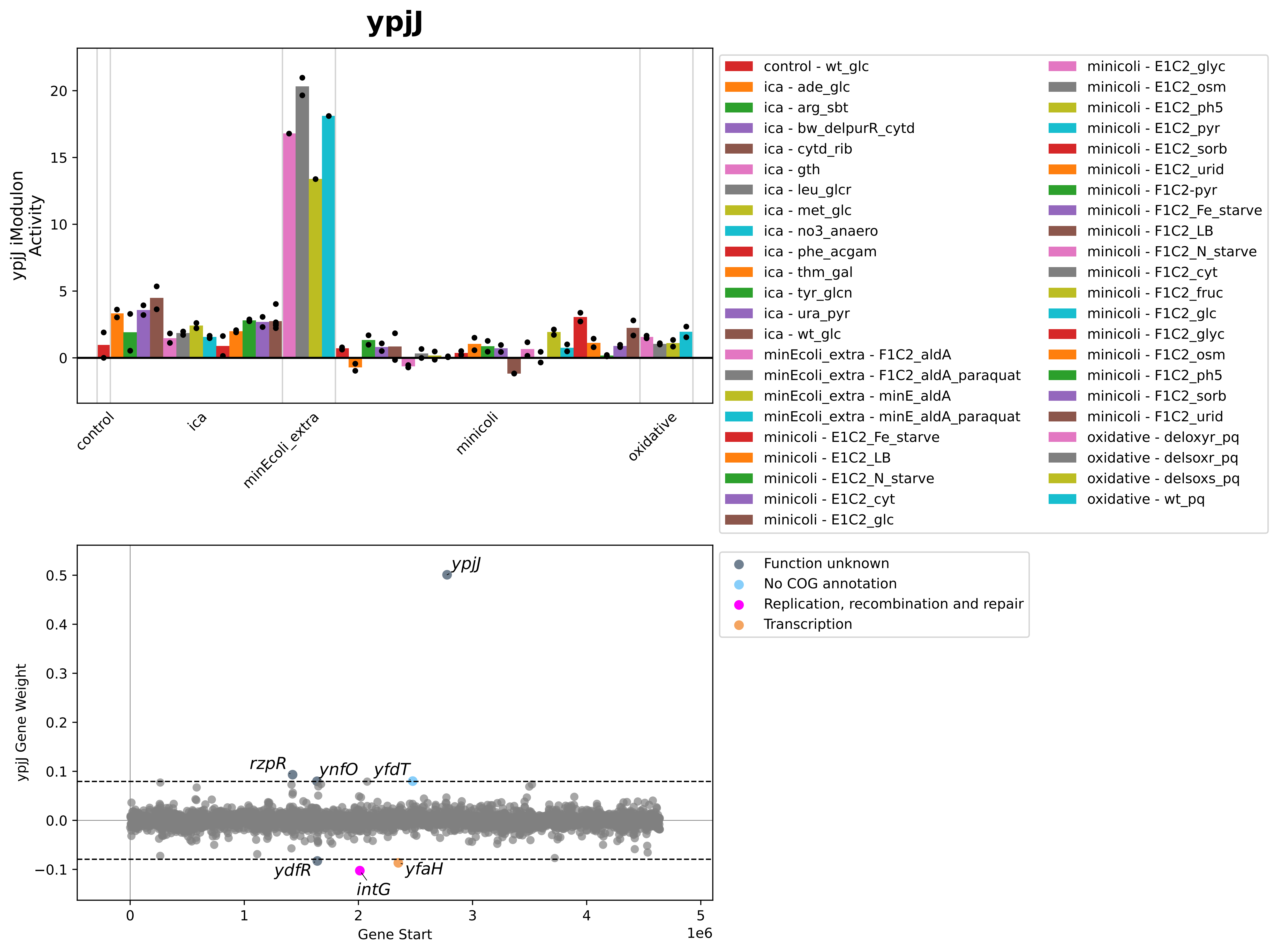
ypjJ
ypjJ iModulon Activity
control
ica
minEcoli_extra
minicoli
oxidative
control - wt_glc
ica - ade_glc
ica - arg_sbt
ica - bw_delpurR_cytd
ica - cytd_rib
ica - gth
ica - leu_glcr
ica - met_glc
ica - no3_anaero
ica - phe_acgam
ica - thm_gal
ica - tyr_glcn
ica - ura_pyr
ica - wt_glc
minEcoli_extra - F1C2_aldA
minEcoli_extra - F1C2_aldA_paraquat
minEcoli_extra - minE_aldA
minEcoli_extra - minE_aldA_paraquat
minicoli - E1C2_Fe_starve
minicoli - E1C2_LB
minicoli - E1C2_N_starve
minicoli - E1C2_cyt
minicoli - E1C2_glc
minicoli - E1C2_glyc
minicoli - E1C2_osm
minicoli - E1C2_ph5
minicoli - E1C2_pyr
minicoli - E1C2_sorb
minicoli - E1C2_urid
minicoli - F1C2-pyr
minicoli - F1C2_Fe_starve
minicoli - F1C2_LB
minicoli - F1C2_N_starve
minicoli - F1C2_cyt
minicoli - F1C2_fruc
minicoli - F1C2_glc
minicoli - F1C2_glyc
minicoli - F1C2_osm
minicoli - F1C2_ph5
minicoli - F1C2_sorb
minicoli - F1C2_urid
oxidative - deloxyr_pq
oxidative - delsoxr_pq
oxidative - delsoxs_pq
oxidative - wt_pq
ypjJ Gene Weight
Gene Start
1e6
ypjJ
rzpR
ynfO
yfdT
ydfR
intG
yfaH
Function unknown
No COG annotation
Replication, recombination and repair
Transcription

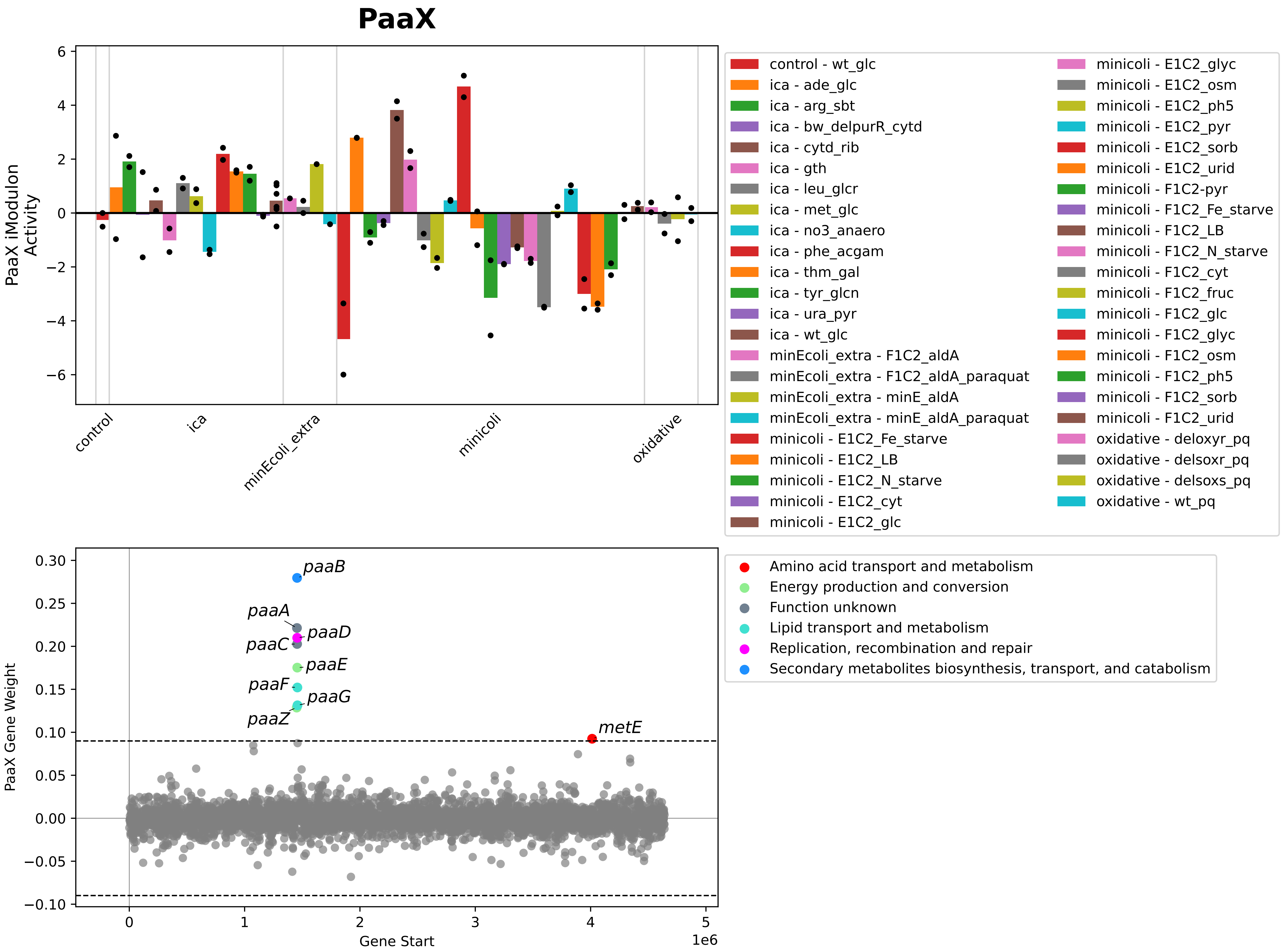

PaaX
PaaX iModulon Activity
control
ica
minEcoli_extra
minicoli
oxidative
control - wt_glc
ica - ade_glc
ica - arg_sbt
ica - bw_delpurR_cytd
ica - cytd_rib
ica - gth
ica - leu_glcr
ica - met_glc
ica - no3_anaero
ica - phe_acgam
ica - thm_gal
ica - tyr_glcn
ica - ura_pyr
ica - wt_glc
minEcoli_extra - F1C2_aldA
minEcoli_extra - F1C2_aldA_paraquat
minEcoli_extra - minE_aldA
minEcoli_extra - minE_aldA_paraquat
minicoli - E1C2_Fe_starve
minicoli - E1C2_LB
minicoli - E1C2_N_starve
minicoli - E1C2_cyt
minicoli - E1C2_glc
minicoli - E1C2_glyc
minicoli - E1C2_osm
minicoli - E1C2_ph5
minicoli - E1C2_pyr
minicoli - E1C2_sorb
minicoli - E1C2_urid
minicoli - F1C2-pyr
minicoli - F1C2_Fe_starve
minicoli - F1C2_LB
minicoli - F1C2_N_starve
minicoli - F1C2_cyt
minicoli - F1C2_fruc
minicoli - F1C2_glc
minicoli - F1C2_glyc
minicoli - F1C2_osm
minicoli - F1C2_ph5
minicoli - F1C2_sorb
minicoli - F1C2_urid
oxidative - deloxyr_pq
oxidative - delsoxr_pq
oxidative - delsoxs_pq
oxidative - wt_pq
PaaX Gene Weight
Gene Start
1e6
paaB
paaA
paaD
paaC
paaE
paaF
paaG
paaZ
metE
Amino acid transport and metabolism
Energy production and conversion
Function unknown
Lipid transport and metabolism
Replication, recombination and repair
Secondary metabolites biosynthesis, transport, and catabolism

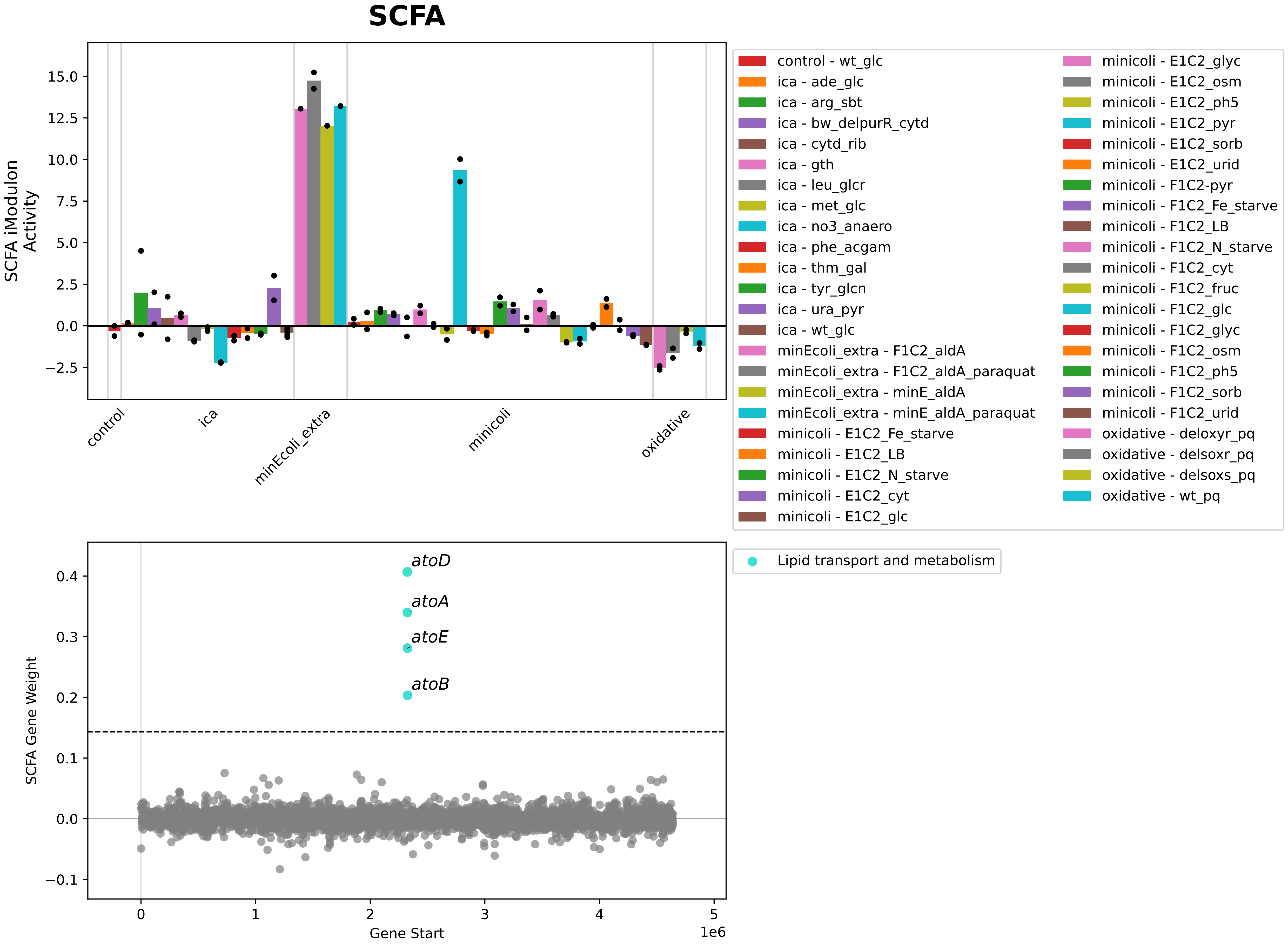
SCFA
SCFA iModulon Activity
control
ica
minEcoli_extra
minicoli
oxidative
control - wt_glc
ica - ade_glc
ica - arg_sbt
ica - bw_delpurR_cytd
ica - cytd_rib
ica - gth
ica - leu_glcr
ica - met_glc
ica - no3_anaero
ica - phe_acgam
ica - thm_gal
ica - tyr_glcn
ica - ura_pyr
ica - wt_glc
minEcoli_extra - F1C2_aldA
minEcoli_extra - F1C2_aldA_paraquat
minEcoli_extra - minE_aldA
minEcoli_extra - minE_aldA_paraquat
minicoli - E1C2_Fe_starve
minicoli - E1C2_LB
minicoli - E1C2_N_starve
minicoli - E1C2_cyt
minicoli - E1C2_glc
minicoli - E1C2_glyc
minicoli - E1C2_osm
minicoli - E1C2_ph5
minicoli - E1C2_pyr
minicoli - E1C2_sorb
minicoli - E1C2_urid
minicoli - F1C2-pyr
minicoli - F1C2_Fe_starve
minicoli - F1C2_LB
minicoli - F1C2_N_starve
minicoli - F1C2_cyt
minicoli - F1C2_fruc
minicoli - F1C2_glc
minicoli - F1C2_glyc
minicoli - F1C2_osm
minicoli - F1C2_ph5
minicoli - F1C2_sorb
minicoli - F1C2_urid
oxidative - deloxyr_pq
oxidative - delsoxr_pq
oxidative - delsoxs_pq
oxidative - wt_pq
SCFA Gene Weight
Gene Start
1e6
atoD
atoA
atoE
atoB
Lipid transport and metabolism

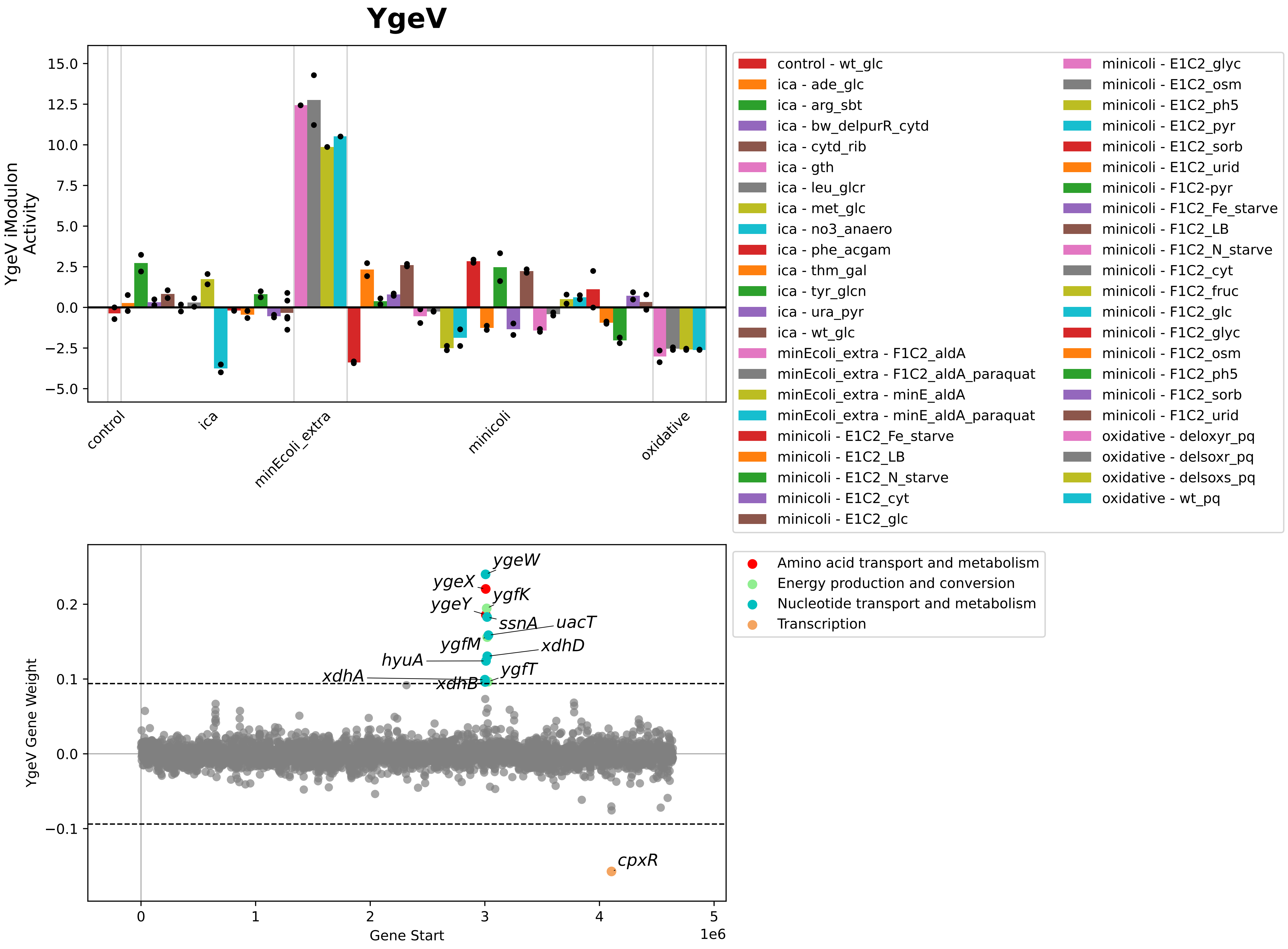
YgeV
YgeV iModulon Activity
control
ica
minEcoli_extra
minicoli
oxidative
control - wt_glc
ica - ade_glc
ica - arg_sbt
ica - bw_delpurR_cytd
ica - cytd_rib
ica - gth
ica - leu_glcr
ica - met_glc
ica - no3_anaero
ica - phe_acgam
ica - thm_gal
ica - tyr_glcn
ica - ura_pyr
ica - wt_glc
minEcoli_extra - F1C2_aldA
minEcoli_extra - F1C2_aldA_paraquat
minEcoli_extra - minE_aldA
minEcoli_extra - minE_aldA_paraquat
minicoli - E1C2_Fe_starve
minicoli - E1C2_LB
minicoli - E1C2_N_starve
minicoli - E1C2_cyt
minicoli - E1C2_glc
minicoli - E1C2_glyc
minicoli - E1C2_osm
minicoli - E1C2_ph5
minicoli - E1C2_pyr
minicoli - E1C2_sorb
minicoli - E1C2_urid
minicoli - F1C2-pyr
minicoli - F1C2_Fe_starve
minicoli - F1C2_LB
minicoli - F1C2_N_starve
minicoli - F1C2_cyt
minicoli - F1C2_fruc
minicoli - F1C2_glc
minicoli - F1C2_glyc
minicoli - F1C2_osm
minicoli - F1C2_ph5
minicoli - F1C2_sorb
minicoli - F1C2_urid
oxidative - deloxyr_pq
oxidative - delsoxr_pq
oxidative - delsoxs_pq
oxidative - wt_pq
YgeV Gene Weight
Gene Start
1e6
ygeW
ygeX
ygfK
ygeY
ssnA
uacT
ygfM
xdhD
hyuA
ygfT
xdhA
xdhB
cpxR
Amino acid transport and metabolism
Energy production and conversion
Nucleotide transport and metabolism
Transcription

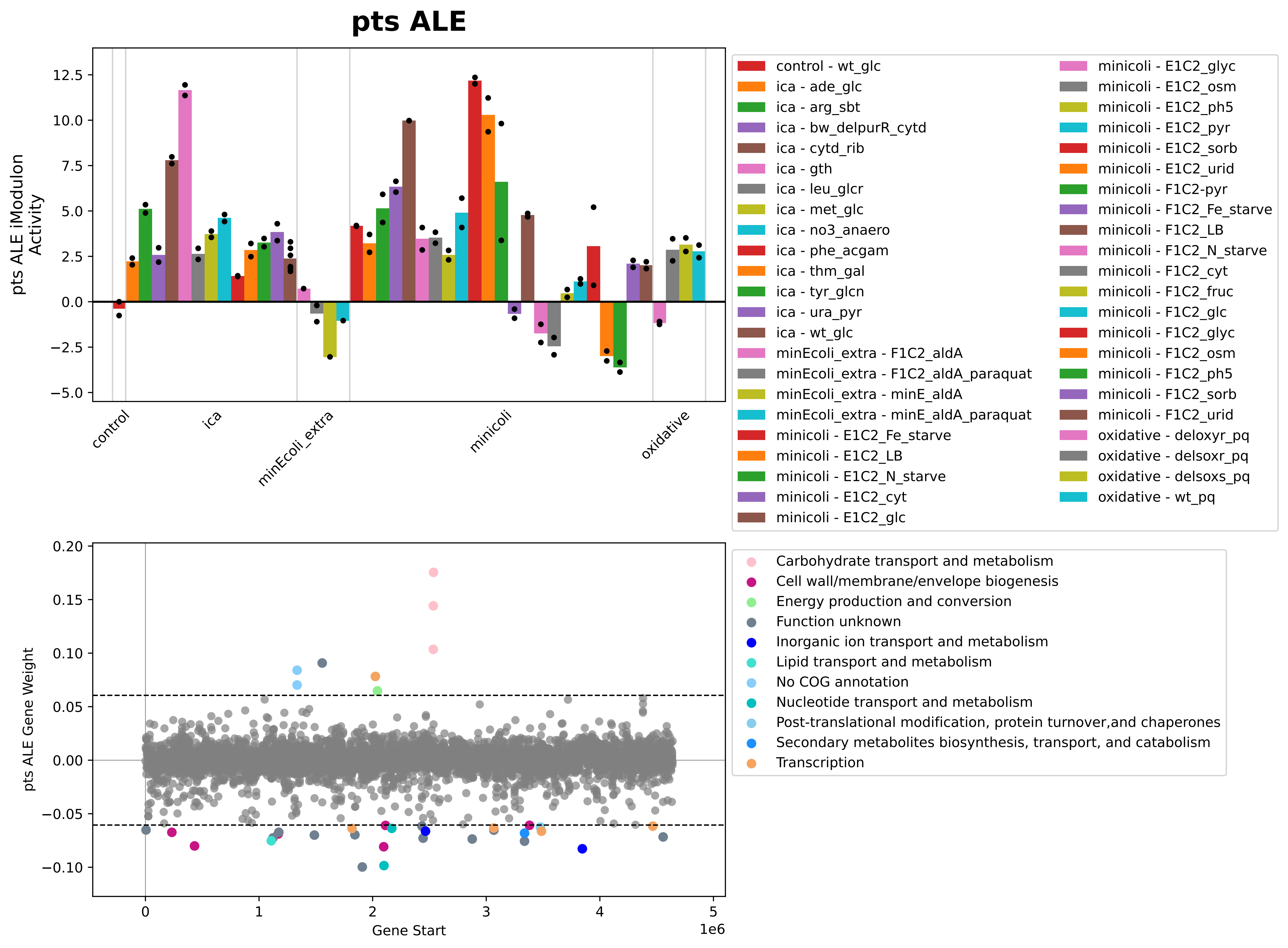

pts ALE
pts ALE iModulon Activity
12.5
10.0
7.5
5.0
2.5
0.0
−2.5
−5.0
control
ica
minEcoli_extra
minicoli
oxidative
control - wt_glc
ica - ade_glc
ica - arg_sbt
ica - bw_delpurR_cytd
ica - cytd_rib
ica - gth
ica - leu_glcr
ica - met_glc
ica - no3_anaero
ica - phe_acgam
ica - thm_gal
ica - tyr_glcn
ica - ura_pyr
ica - wt_glc
minEcoli_extra - F1C2_aldA
minEcoli_extra - F1C2_aldA_paraquat
minEcoli_extra - minE_aldA
minEcoli_extra - minE_aldA_paraquat
minicoli - E1C2_Fe_starve
minicoli - E1C2_LB
minicoli - E1C2_N_starve
minicoli - E1C2_cyt
minicoli - E1C2_glc
minicoli - E1C2_glyc
minicoli - E1C2_osm
minicoli - E1C2_ph5
minicoli - E1C2_pyr
minicoli - E1C2_sorb
minicoli - E1C2_urid
minicoli - F1C2-pyr
minicoli - F1C2_Fe_starve
minicoli - F1C2_LB
minicoli - F1C2_N_starve
minicoli - F1C2_cyt
minicoli - F1C2_fruc
minicoli - F1C2_glc
minicoli - F1C2_glyc
minicoli - F1C2_osm
minicoli - F1C2_ph5
minicoli - F1C2_sorb
minicoli - F1C2_urid
oxidative - deloxyr_pq
oxidative - delsoxr_pq
oxidative - delsoxs_pq
oxidative - wt_pq
pts ALE Gene Weight
0.20
0.15
0.10
0.05
0.00
−0.05
−0.10
0
1
2
3
4
5
Gene Start
1e6
Carbohydrate transport and metabolism
Cell wall/membrane/envelope biogenesis
Energy production and conversion
Function unknown
Inorganic ion transport and metabolism
Lipid transport and metabolism
No COG annotation
Nucleotide transport and metabolism
Post-translational modification, protein turnover,and chaperones
Secondary metabolites biosynthesis, transport, and catabolism
Transcription

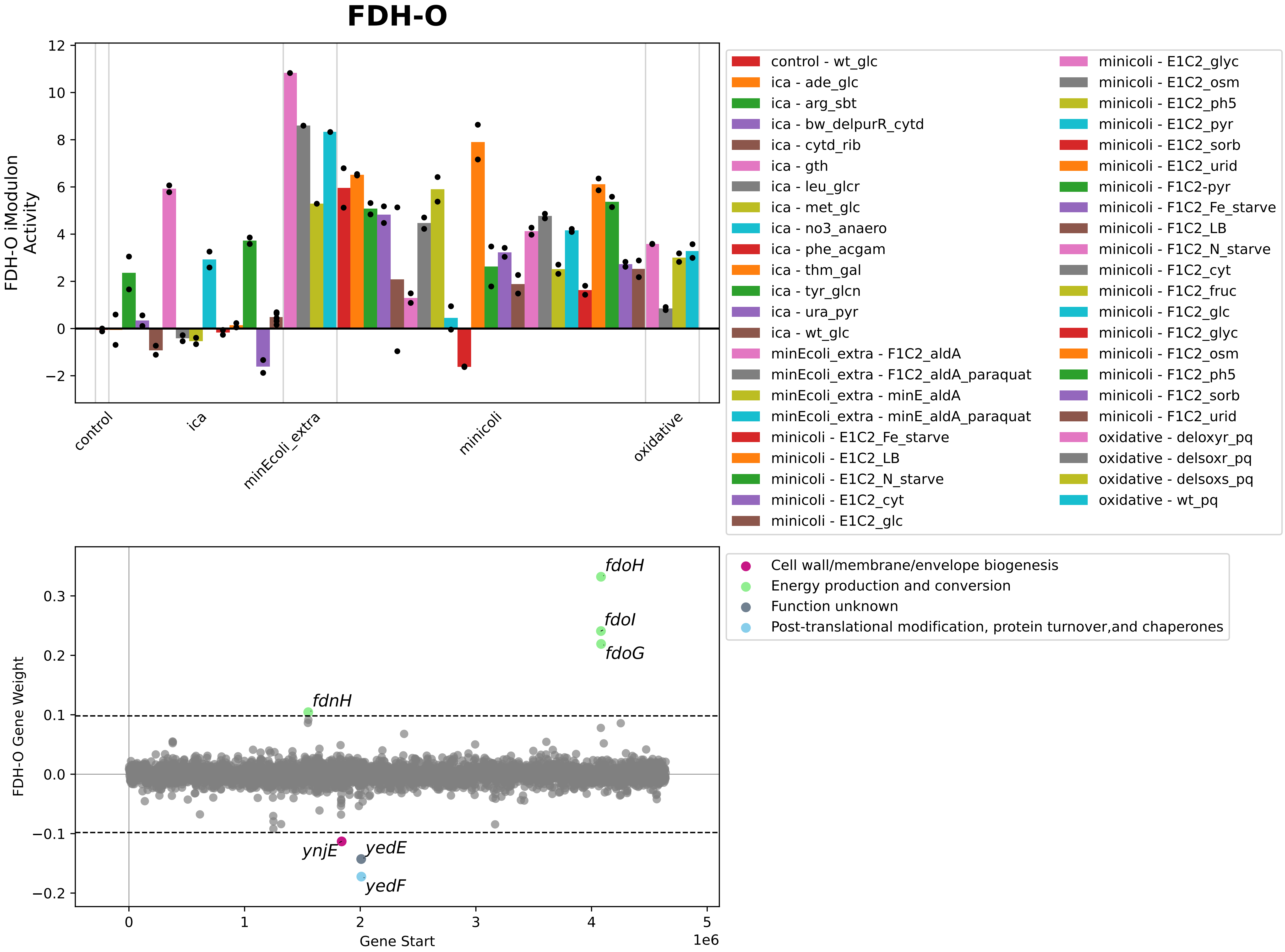
FDH-O
FDH-O iModulon Activity
control
ica
minEcoli_extra
minicoli
oxidative
control - wt_glc
ica - ade_glc
ica - arg_sbt
ica - bw_delpurR_cytd
ica - cytd_rib
ica - gth
ica - leu_glcr
ica - met_glc
ica - no3_anaero
ica - phe_acgam
ica - thm_gal
ica - tyr_glcn
ica - ura_pyr
ica - wt_glc
minEcoli_extra - F1C2_aldA
minEcoli_extra - F1C2_aldA_paraquat
minEcoli_extra - minE_aldA
minEcoli_extra - minE_aldA_paraquat
minicoli - E1C2_Fe_starve
minicoli - E1C2_LB
minicoli - E1C2_N_starve
minicoli - E1C2_cyt
minicoli - E1C2_glc
minicoli - E1C2_glyc
minicoli - E1C2_osm
minicoli - E1C2_ph5
minicoli - E1C2_pyr
minicoli - E1C2_sorb
minicoli - E1C2_urid
minicoli - F1C2-pyr
minicoli - F1C2_Fe_starve
minicoli - F1C2_LB
minicoli - F1C2_N_starve
minicoli - F1C2_cyt
minicoli - F1C2_fruc
minicoli - F1C2_glc
minicoli - F1C2_glyc
minicoli - F1C2_osm
minicoli - F1C2_ph5
minicoli - F1C2_sorb
minicoli - F1C2_urid
oxidative - deloxyr_pq
oxidative - delsoxr_pq
oxidative - delsoxs_pq
oxidative - wt_pq
FDH-O Gene Weight
Gene Start
1e6
fdoH
fdoI
fdoG
fdnH
ynjE
yedE
yedF
Cell wall/membrane/envelope biogenesis
Energy production and conversion
Function unknown
Post-translational modification, protein turnover,and chaperones

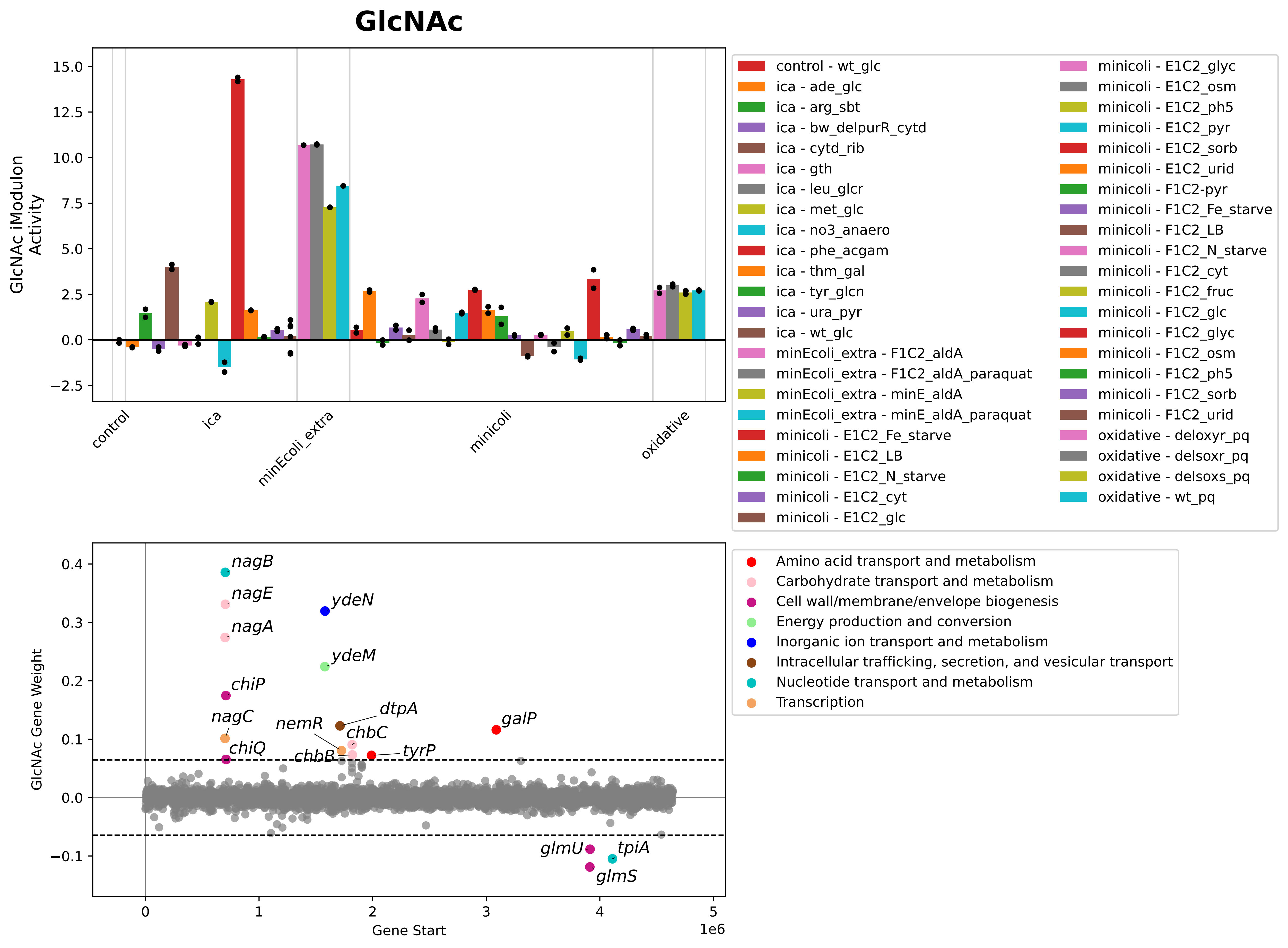
GlcNAc
GlcNAc iModulon Activity
control
ica
minEcoli_extra
minicoli
oxidative
control - wt_glc
ica - ade_glc
ica - arg_sbt
ica - bw_delpurR_cytd
ica - cytd_rib
ica - gth
ica - leu_glcr
ica - met_glc
ica - no3_anaero
ica - phe_acgam
ica - thm_gal
ica - tyr_glcn
ica - ura_pyr
ica - wt_glc
minEcoli_extra - F1C2_aldA
minEcoli_extra - F1C2_aldA_paraquat
minEcoli_extra - minE_aldA
minEcoli_extra - minE_aldA_paraquat
minicoli - E1C2_Fe_starve
minicoli - E1C2_LB
minicoli - E1C2_N_starve
minicoli - E1C2_cyt
minicoli - E1C2_glc
minicoli - E1C2_glyc
minicoli - E1C2_osm
minicoli - E1C2_ph5
minicoli - E1C2_pyr
minicoli - E1C2_sorb
minicoli - E1C2_urid
minicoli - F1C2-pyr
minicoli - F1C2_Fe_starve
minicoli - F1C2_LB
minicoli - F1C2_N_starve
minicoli - F1C2_cyt
minicoli - F1C2_fruc
minicoli - F1C2_glc
minicoli - F1C2_glyc
minicoli - F1C2_osm
minicoli - F1C2_ph5
minicoli - F1C2_sorb
minicoli - F1C2_urid
oxidative - deloxyr_pq
oxidative - delsoxr_pq
oxidative - delsoxs_pq
oxidative - wt_pq
GlcNAc Gene Weight
Gene Start
1e6
nagB
nagE
nagA
ydeN
ydeM
chiP
nagC
dtpA
nemR
chbC
galP
chiQ
chbB
tyrP
glmU
tpiA
glmS
Amino acid transport and metabolism
Carbohydrate transport and metabolism
Cell wall/membrane/envelope biogenesis
Energy production and conversion
Inorganic ion transport and metabolism
Intracellular trafficking, secretion, and vesicular transport
Nucleotide transport and metabolism
Transcription

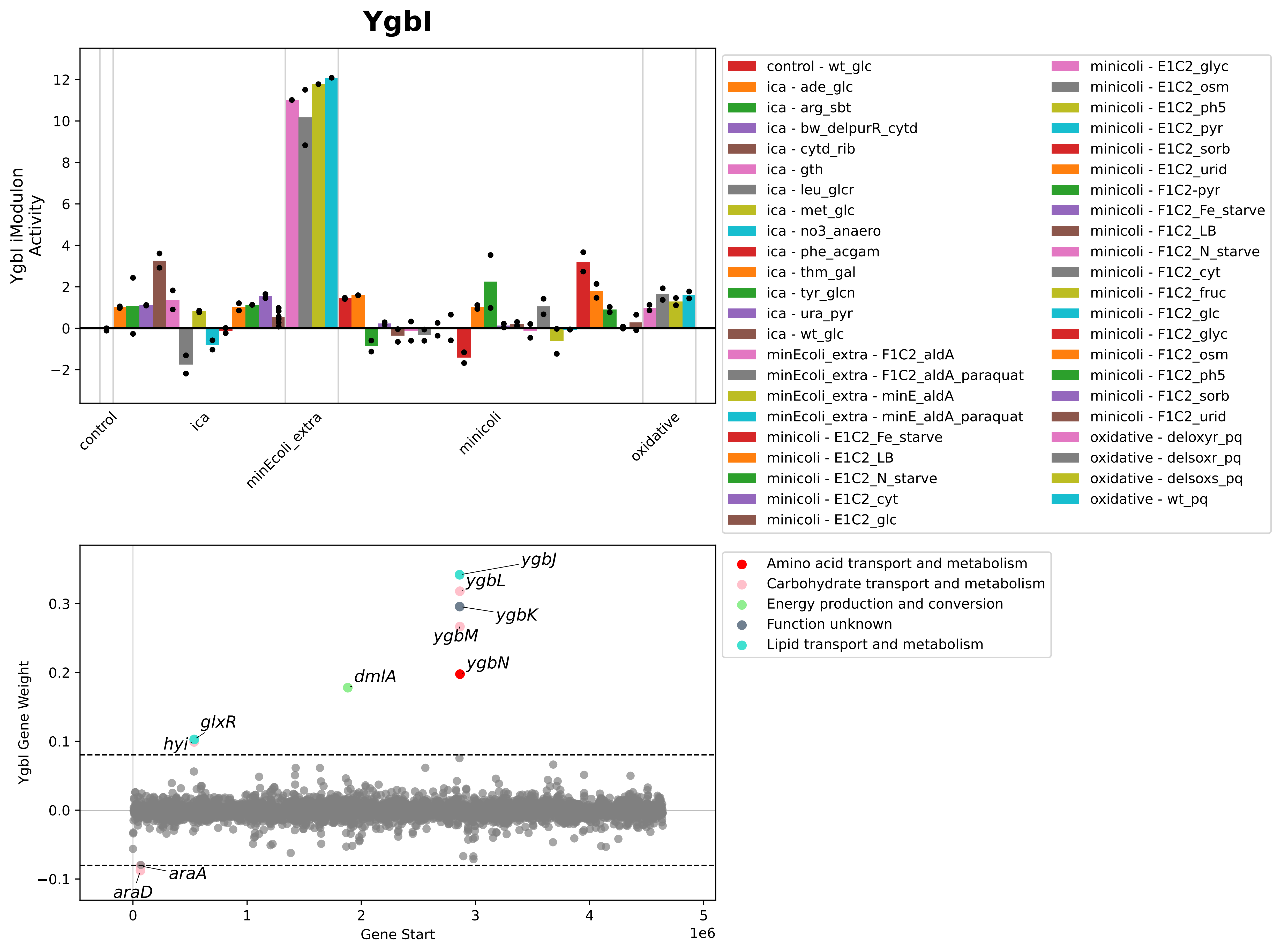
YgbI
YgbI iModulon Activity
control
ica
minEcoli_extra
minicoli
oxidative
control - wt_glc
ica - ade_glc
ica - arg_sbt
ica - bw_delpurR_cytd
ica - cytd_rib
ica - gth
ica - leu_glcr
ica - met_glc
ica - no3_anaero
ica - phe_acgam
ica - thm_gal
ica - tyr_glcn
ica - ura_pyr
ica - wt_glc
minEcoli_extra - F1C2_aldA
minEcoli_extra - F1C2_aldA_paraquat
minEcoli_extra - minE_aldA
minEcoli_extra - minE_aldA_paraquat
minicoli - E1C2_Fe_starve
minicoli - E1C2_LB
minicoli - E1C2_N_starve
minicoli - E1C2_cyt
minicoli - E1C2_glc
minicoli - E1C2_glyc
minicoli - E1C2_osm
minicoli - E1C2_ph5
minicoli - E1C2_pyr
minicoli - E1C2_sorb
minicoli - E1C2_urid
minicoli - F1C2-pyr
minicoli - F1C2_Fe_starve
minicoli - F1C2_LB
minicoli - F1C2_N_starve
minicoli - F1C2_cyt
minicoli - F1C2_fruc
minicoli - F1C2_glc
minicoli - F1C2_glyc
minicoli - F1C2_osm
minicoli - F1C2_ph5
minicoli - F1C2_sorb
minicoli - F1C2_urid
oxidative - deloxyr_pq
oxidative - delsoxr_pq
oxidative - delsoxs_pq
oxidative - wt_pq
ygbJ
ygbL
ygbK
ygbM
ygbN
dmlA
glxR
hyi
araA
araD
YgbI Gene Weight
Gene Start
1e6
Amino acid transport and metabolism
Carbohydrate transport and metabolism
Energy production and conversion
Function unknown
Lipid transport and metabolism

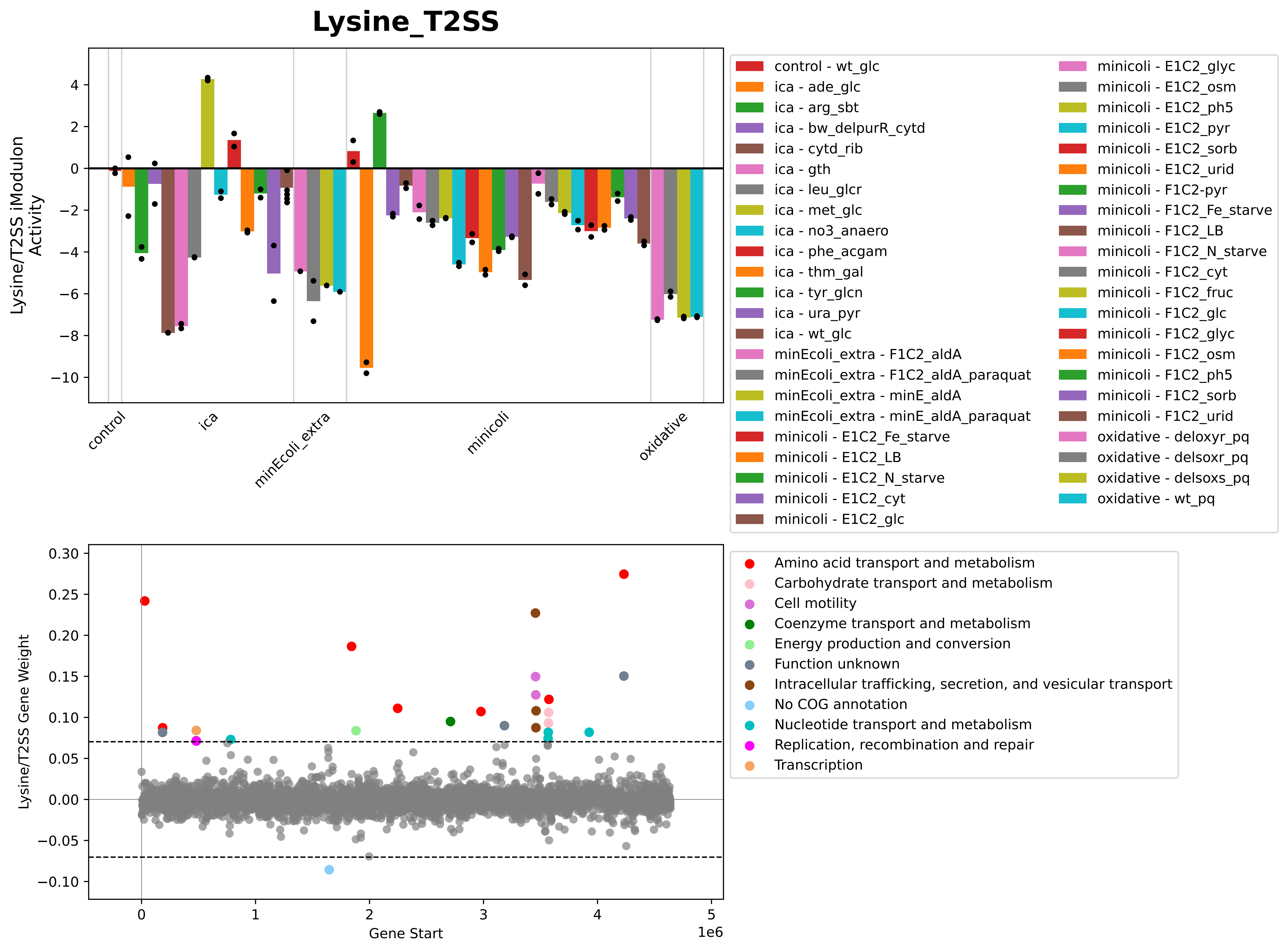

Lysine_T2SS
Lysine/T2SS iModulon Activity
control
ica
minEcoli_extra
minicoli
oxidative
control - wt_glc
ica - ade_glc
ica - arg_sbt
ica - bw_delpurR_cytd
ica - cytd_rib
ica - gth
ica - leu_glcr
ica - met_glc
ica - no3_anaero
ica - phe_acgam
ica - thm_gal
ica - tyr_glcn
ica - ura_pyr
ica - wt_glc
minEcoli_extra - F1C2_aldA
minEcoli_extra - F1C2_aldA_paraquat
minEcoli_extra - minE_aldA
minEcoli_extra - minE_aldA_paraquat
minicoli - E1C2_Fe_starve
minicoli - E1C2_LB
minicoli - E1C2_N_starve
minicoli - E1C2_cyt
minicoli - E1C2_glc
minicoli - E1C2_glyc
minicoli - E1C2_osm
minicoli - E1C2_ph5
minicoli - E1C2_pyr
minicoli - E1C2_sorb
minicoli - E1C2_urid
minicoli - F1C2-pyr
minicoli - F1C2_Fe_starve
minicoli - F1C2_LB
minicoli - F1C2_N_starve
minicoli - F1C2_cyt
minicoli - F1C2_fruc
minicoli - F1C2_glc
minicoli - F1C2_glyc
minicoli - F1C2_osm
minicoli - F1C2_ph5
minicoli - F1C2_sorb
minicoli - F1C2_urid
oxidative - deloxyr_pq
oxidative - delsoxr_pq
oxidative - delsoxs_pq
oxidative - wt_pq
Lysine/T2SS Gene Weight
Gene Start
1e6
Amino acid transport and metabolism
Carbohydrate transport and metabolism
Cell motility
Coenzyme transport and metabolism
Energy production and conversion
Function unknown
Intracellular trafficking, secretion, and vesicular transport
No COG annotation
Nucleotide transport and metabolism
Replication, recombination and repair
Transcription

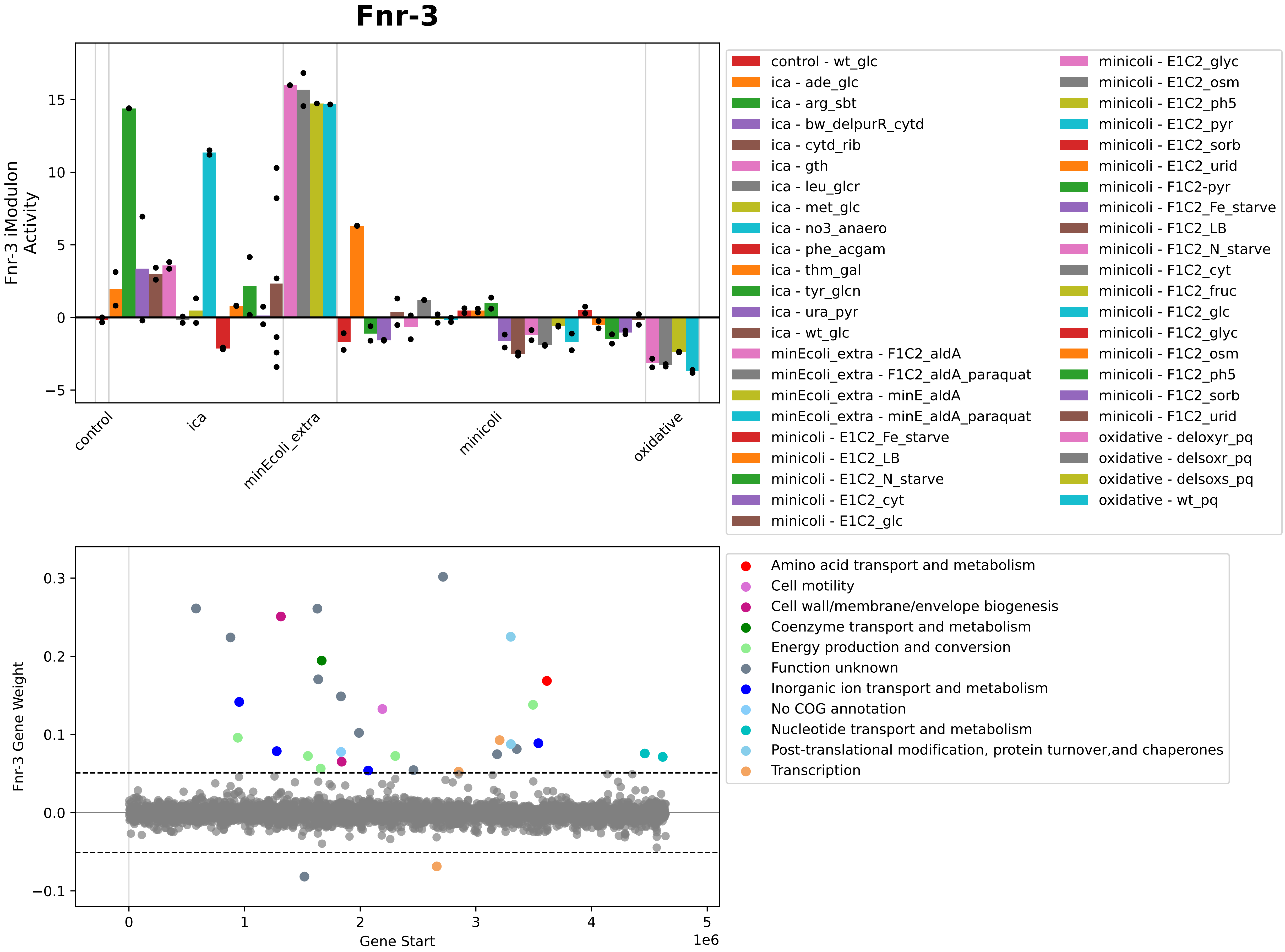

Fnr-3
Fnr-3 iModulon Activity
control
ica
minEcoli_extra
minicoli
oxidative
control - wt_glc
ica - ade_glc
ica - arg_sbt
ica - bw_delpurR_cytd
ica - cytd_rib
ica - gth
ica - leu_glcr
ica - met_glc
ica - no3_anaero
ica - phe_acgam
ica - thm_gal
ica - tyr_glcn
ica - ura_pyr
ica - wt_glc
minEcoli_extra - F1C2_aldA
minEcoli_extra - F1C2_aldA_paraquat
minEcoli_extra - minE_aldA
minEcoli_extra - minE_aldA_paraquat
minicoli - E1C2_Fe_starve
minicoli - E1C2_LB
minicoli - E1C2_N_starve
minicoli - E1C2_cyt
minicoli - E1C2_glc
minicoli - E1C2_glyc
minicoli - E1C2_osm
minicoli - E1C2_ph5
minicoli - E1C2_pyr
minicoli - E1C2_sorb
minicoli - E1C2_urid
minicoli - F1C2-pyr
minicoli - F1C2_Fe_starve
minicoli - F1C2_LB
minicoli - F1C2_N_starve
minicoli - F1C2_cyt
minicoli - F1C2_fruc
minicoli - F1C2_glc
minicoli - F1C2_glyc
minicoli - F1C2_osm
minicoli - F1C2_ph5
minicoli - F1C2_sorb
minicoli - F1C2_urid
oxidative - deloxyr_pq
oxidative - delsoxr_pq
oxidative - delsoxs_pq
oxidative - wt_pq
Fnr-3 Gene Weight
Gene Start
1e6
Amino acid transport and metabolism
Cell motility
Cell wall/membrane/envelope biogenesis
Coenzyme transport and metabolism
Energy production and conversion
Function unknown
Inorganic ion transport and metabolism
No COG annotation
Nucleotide transport and metabolism
Post-translational modification, protein turnover,and chaperones
Transcription

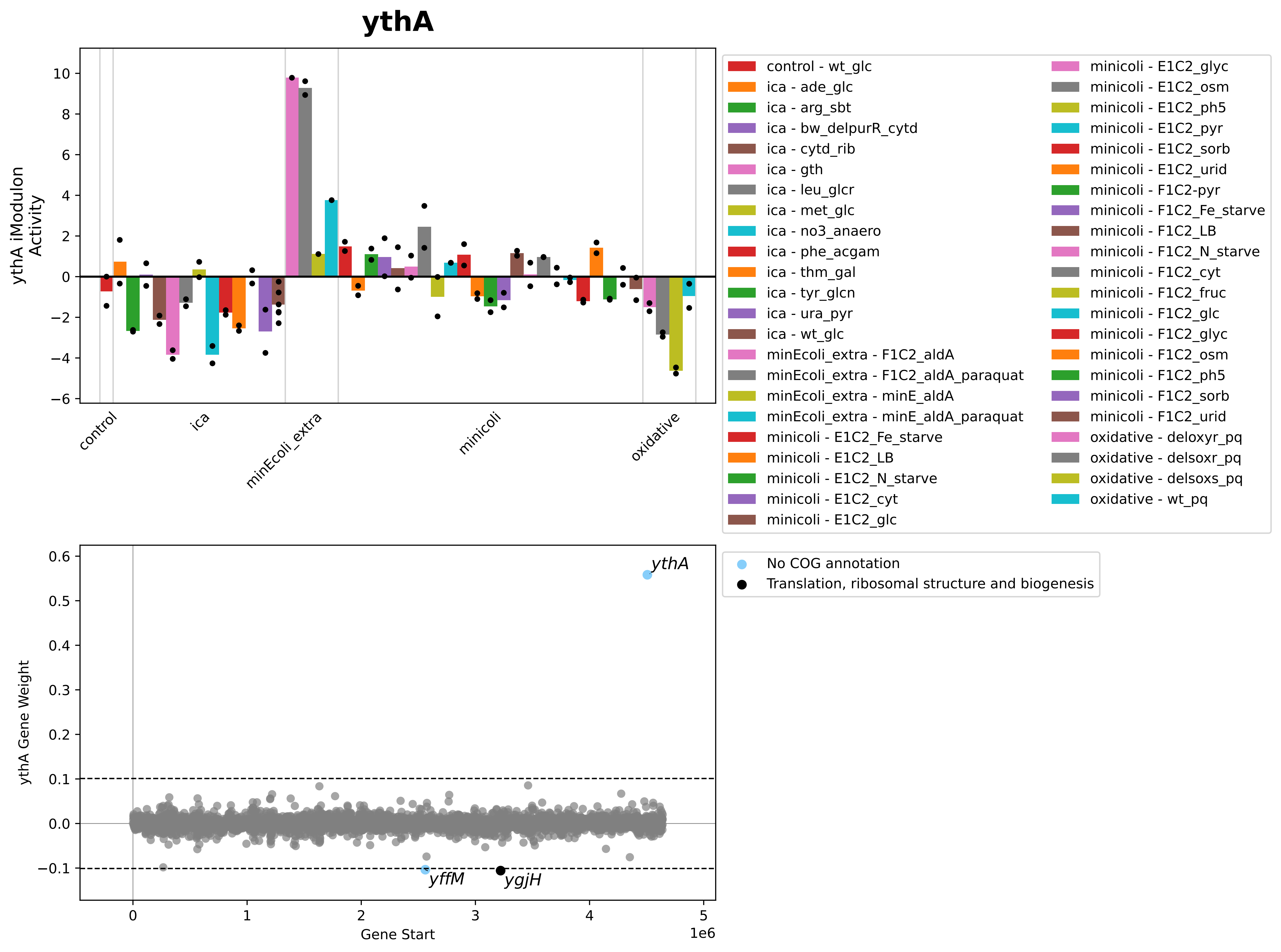
ythA
ythA iModulon Activity
10
8
6
4
2
0
−2
−4
−6
control
ica
minEcoli_extra
minicoli
oxidative
control - wt_glc
ica - ade_glc
ica - arg_sbt
ica - bw_delpurR_cytd
ica - cytd_rib
ica - gth
ica - leu_glcr
ica - met_glc
ica - no3_anaero
ica - phe_acgam
ica - thm_gal
ica - tyr_glcn
ica - ura_pyr
ica - wt_glc
minEcoli_extra - F1C2_aldA
minEcoli_extra - F1C2_aldA_paraquat
minEcoli_extra - minE_aldA
minEcoli_extra - minE_aldA_paraquat
minicoli - E1C2_Fe_starve
minicoli - E1C2_LB
minicoli - E1C2_N_starve
minicoli - E1C2_cyt
minicoli - E1C2_glc
minicoli - E1C2_glyc
minicoli - E1C2_osm
minicoli - E1C2_ph5
minicoli - E1C2_pyr
minicoli - E1C2_sorb
minicoli - E1C2_urid
minicoli - F1C2-pyr
minicoli - F1C2_Fe_starve
minicoli - F1C2_LB
minicoli - F1C2_N_starve
minicoli - F1C2_cyt
minicoli - F1C2_fruc
minicoli - F1C2_glc
minicoli - F1C2_glyc
minicoli - F1C2_osm
minicoli - F1C2_ph5
minicoli - F1C2_sorb
minicoli - F1C2_urid
oxidative - deloxyr_pq
oxidative - delsoxr_pq
oxidative - delsoxs_pq
oxidative - wt_pq
ythA Gene Weight
0.6
0.5
0.4
0.3
0.2
0.1
0.0
−0.1
ythA
yffM
ygjH
0
1
2
3
4
5
Gene Start
1e6
No COG annotation
Translation, ribosomal structure and biogenesis

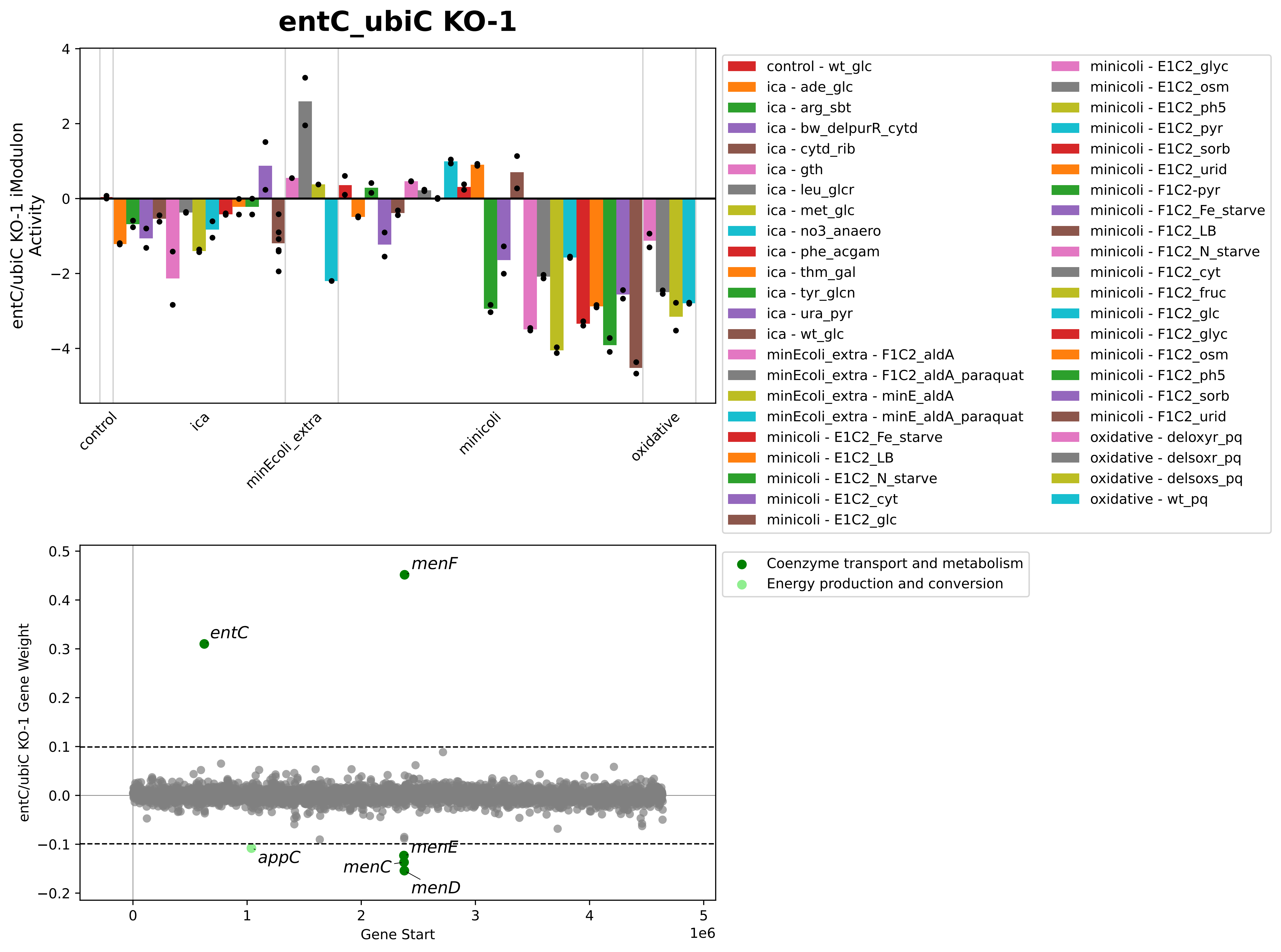
entC_ubiC KO-1
entC/ubiC KO-1 iModulon Activity
control
ica
minEcoli_extra
minicoli
oxidative
control - wt_glc
ica - ade_glc
ica - arg_sbt
ica - bw_delpurR_cytd
ica - cytd_rib
ica - gth
ica - leu_glcr
ica - met_glc
ica - no3_anaero
ica - phe_acgam
ica - thm_gal
ica - tyr_glcn
ica - ura_pyr
ica - wt_glc
minEcoli_extra - F1C2_aldA
minEcoli_extra - F1C2_aldA_paraquat
minEcoli_extra - minE_aldA
minEcoli_extra - minE_aldA_paraquat
minicoli - E1C2_Fe_starve
minicoli - E1C2_LB
minicoli - E1C2_N_starve
minicoli - E1C2_cyt
minicoli - E1C2_glc
minicoli - E1C2_glyc
minicoli - E1C2_osm
minicoli - E1C2_ph5
minicoli - E1C2_pyr
minicoli - E1C2_sorb
minicoli - E1C2_urid
minicoli - F1C2-pyr
minicoli - F1C2_Fe_starve
minicoli - F1C2_LB
minicoli - F1C2_N_starve
minicoli - F1C2_cyt
minicoli - F1C2_fruc
minicoli - F1C2_glc
minicoli - F1C2_glyc
minicoli - F1C2_osm
minicoli - F1C2_ph5
minicoli - F1C2_sorb
minicoli - F1C2_urid
oxidative - deloxyr_pq
oxidative - delsoxr_pq
oxidative - delsoxs_pq
oxidative - wt_pq
entC/ubiC KO-1 Gene Weight
Gene Start
1e6
menF
entC
appC
menE
menC
menD
Coenzyme transport and metabolism
Energy production and conversion

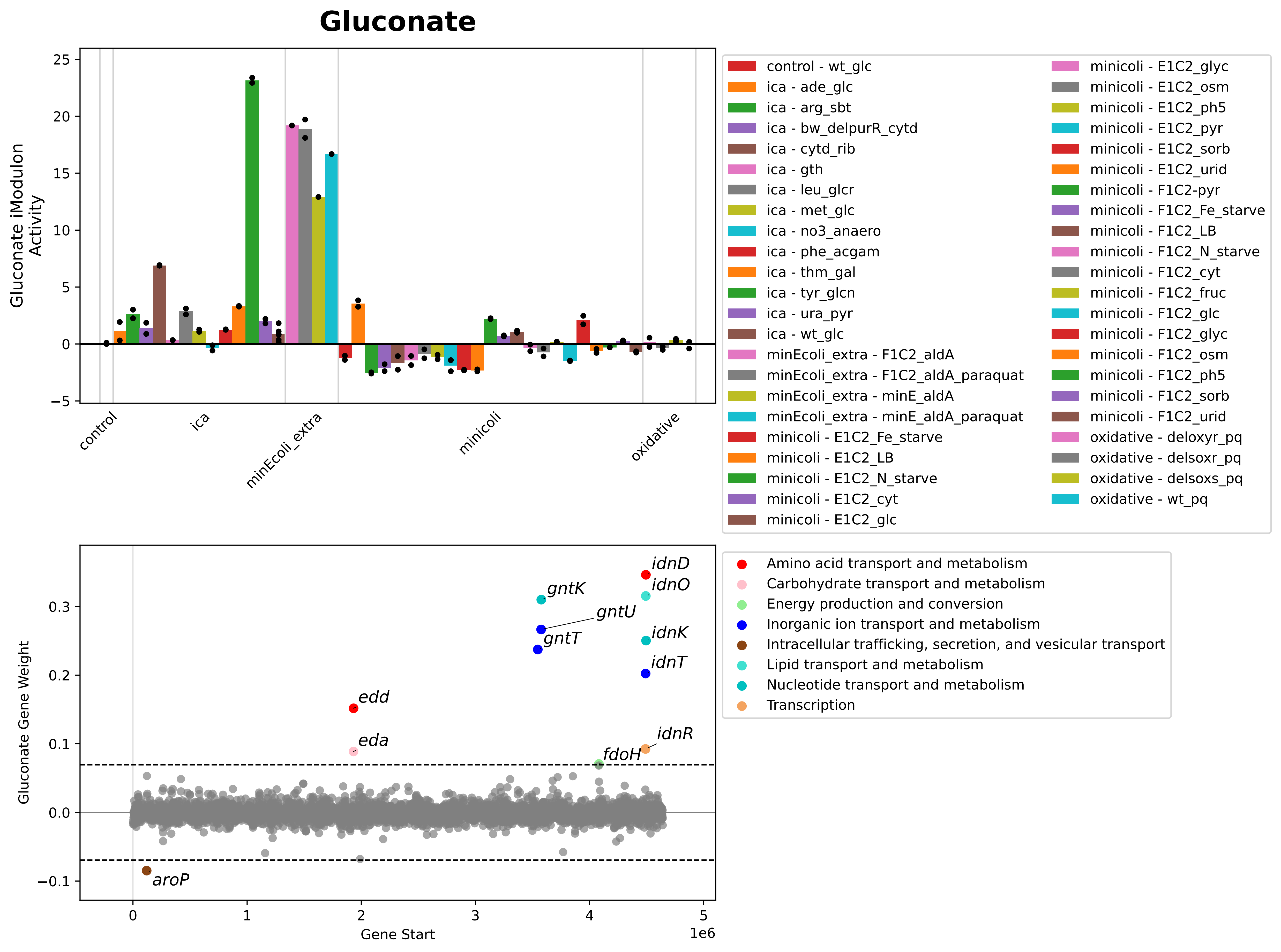
Gluconate
Gluconate iModulon Activity
control
ica
minEcoli_extra
minicoli
oxidative
control - wt_glc
ica - ade_glc
ica - arg_sbt
ica - bw_delpurR_cytd
ica - cytd_rib
ica - gth
ica - leu_glcr
ica - met_glc
ica - no3_anaero
ica - phe_acgam
ica - thm_gal
ica - tyr_glcn
ica - ura_pyr
ica - wt_glc
minEcoli_extra - F1C2_aldA
minEcoli_extra - F1C2_aldA_paraquat
minEcoli_extra - minE_aldA
minEcoli_extra - minE_aldA_paraquat
minicoli - E1C2_Fe_starve
minicoli - E1C2_LB
minicoli - E1C2_N_starve
minicoli - E1C2_cyt
minicoli - E1C2_glc
minicoli - E1C2_glyc
minicoli - E1C2_osm
minicoli - E1C2_ph5
minicoli - E1C2_pyr
minicoli - E1C2_sorb
minicoli - E1C2_urid
minicoli - F1C2-pyr
minicoli - F1C2_Fe_starve
minicoli - F1C2_LB
minicoli - F1C2_N_starve
minicoli - F1C2_cyt
minicoli - F1C2_fruc
minicoli - F1C2_glc
minicoli - F1C2_glyc
minicoli - F1C2_osm
minicoli - F1C2_ph5
minicoli - F1C2_sorb
minicoli - F1C2_urid
oxidative - deloxyr_pq
oxidative - delsoxr_pq
oxidative - delsoxs_pq
oxidative - wt_pq
Gluconate Gene Weight
Gene Start
1e6
idnD
idnO
gntK
gntU
gntT
idnK
idnT
edd
eda
idnR
fdoH
aroP
Amino acid transport and metabolism
Carbohydrate transport and metabolism
Energy production and conversion
Inorganic ion transport and metabolism
Intracellular trafficking, secretion, and vesicular transport
Lipid transport and metabolism
Nucleotide transport and metabolism
Transcription

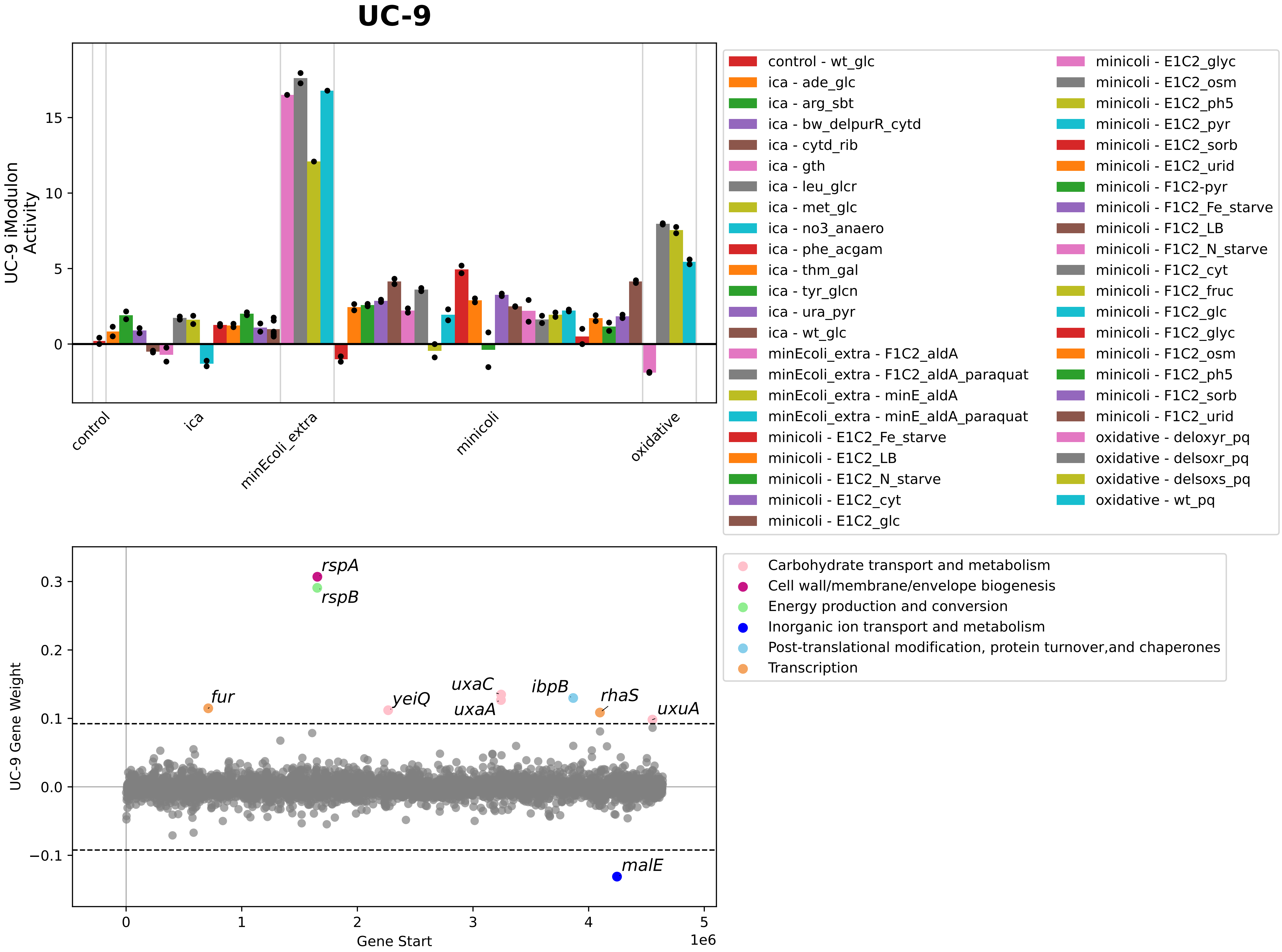
UC-9
UC-9 iModulon Activity
control
ica
minEcoli_extra
minicoli
oxidative
control - wt_glc
ica - ade_glc
ica - arg_sbt
ica - bw_delpurR_cytd
ica - cytd_rib
ica - gth
ica - leu_glcr
ica - met_glc
ica - no3_anaero
ica - phe_acgam
ica - thm_gal
ica - tyr_glcn
ica - ura_pyr
ica - wt_glc
minEcoli_extra - F1C2_aldA
minEcoli_extra - F1C2_aldA_paraquat
minEcoli_extra - minE_aldA
minEcoli_extra - minE_aldA_paraquat
minicoli - E1C2_Fe_starve
minicoli - E1C2_LB
minicoli - E1C2_N_starve
minicoli - E1C2_cyt
minicoli - E1C2_glc
minicoli - E1C2_glyc
minicoli - E1C2_osm
minicoli - E1C2_ph5
minicoli - E1C2_pyr
minicoli - E1C2_sorb
minicoli - E1C2_urid
minicoli - F1C2-pyr
minicoli - F1C2_Fe_starve
minicoli - F1C2_LB
minicoli - F1C2_N_starve
minicoli - F1C2_cyt
minicoli - F1C2_fruc
minicoli - F1C2_glc
minicoli - F1C2_glyc
minicoli - F1C2_osm
minicoli - F1C2_ph5
minicoli - F1C2_sorb
minicoli - F1C2_urid
oxidative - deloxyr_pq
oxidative - delsoxr_pq
oxidative - delsoxs_pq
oxidative - wt_pq
UC-9 Gene Weight
Gene Start
1e6
rspA
rspB
fur
yeiQ
uxaC
uxaA
ibpB
rhaS
uxuA
malE
Carbohydrate transport and metabolism
Cell wall/membrane/envelope biogenesis
Energy production and conversion
Inorganic ion transport and metabolism
Post-translational modification, protein turnover,and chaperones
Transcription

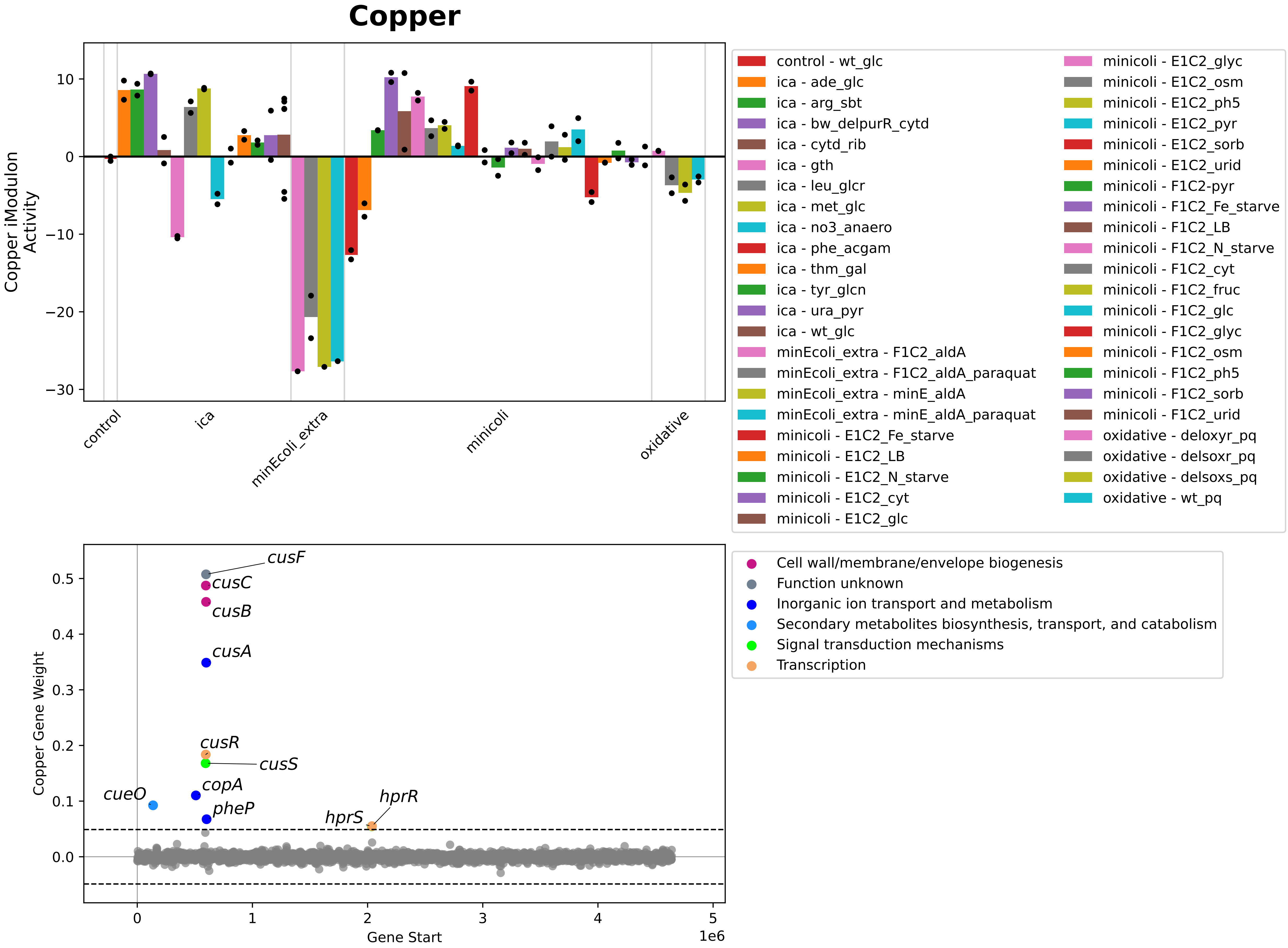
Copper
Copper iModulon Activity
control
ica
minEcoli_extra
minicoli
oxidative
control - wt_glc
ica - ade_glc
ica - arg_sbt
ica - bw_delpurR_cytd
ica - cytd_rib
ica - gth
ica - leu_glcr
ica - met_glc
ica - no3_anaero
ica - phe_acgam
ica - thm_gal
ica - tyr_glcn
ica - ura_pyr
ica - wt_glc
minEcoli_extra - F1C2_aldA
minEcoli_extra - F1C2_aldA_paraquat
minEcoli_extra - minE_aldA
minEcoli_extra - minE_aldA_paraquat
minicoli - E1C2_Fe_starve
minicoli - E1C2_LB
minicoli - E1C2_N_starve
minicoli - E1C2_cyt
minicoli - E1C2_glc
minicoli - E1C2_glyc
minicoli - E1C2_osm
minicoli - E1C2_ph5
minicoli - E1C2_pyr
minicoli - E1C2_sorb
minicoli - E1C2_urid
minicoli - F1C2-pyr
minicoli - F1C2_Fe_starve
minicoli - F1C2_LB
minicoli - F1C2_N_starve
minicoli - F1C2_cyt
minicoli - F1C2_fruc
minicoli - F1C2_glc
minicoli - F1C2_glyc
minicoli - F1C2_osm
minicoli - F1C2_ph5
minicoli - F1C2_sorb
minicoli - F1C2_urid
oxidative - deloxyr_pq
oxidative - delsoxr_pq
oxidative - delsoxs_pq
oxidative - wt_pq
Copper Gene Weight
Gene Start
1e6
cusF
cusC
cusB
cusA
cusR
cusS
copA
cueO
pheP
hprR
hprS
Cell wall/membrane/envelope biogenesis
Function unknown
Inorganic ion transport and metabolism
Secondary metabolites biosynthesis, transport, and catabolism
Signal transduction mechanisms
Transcription

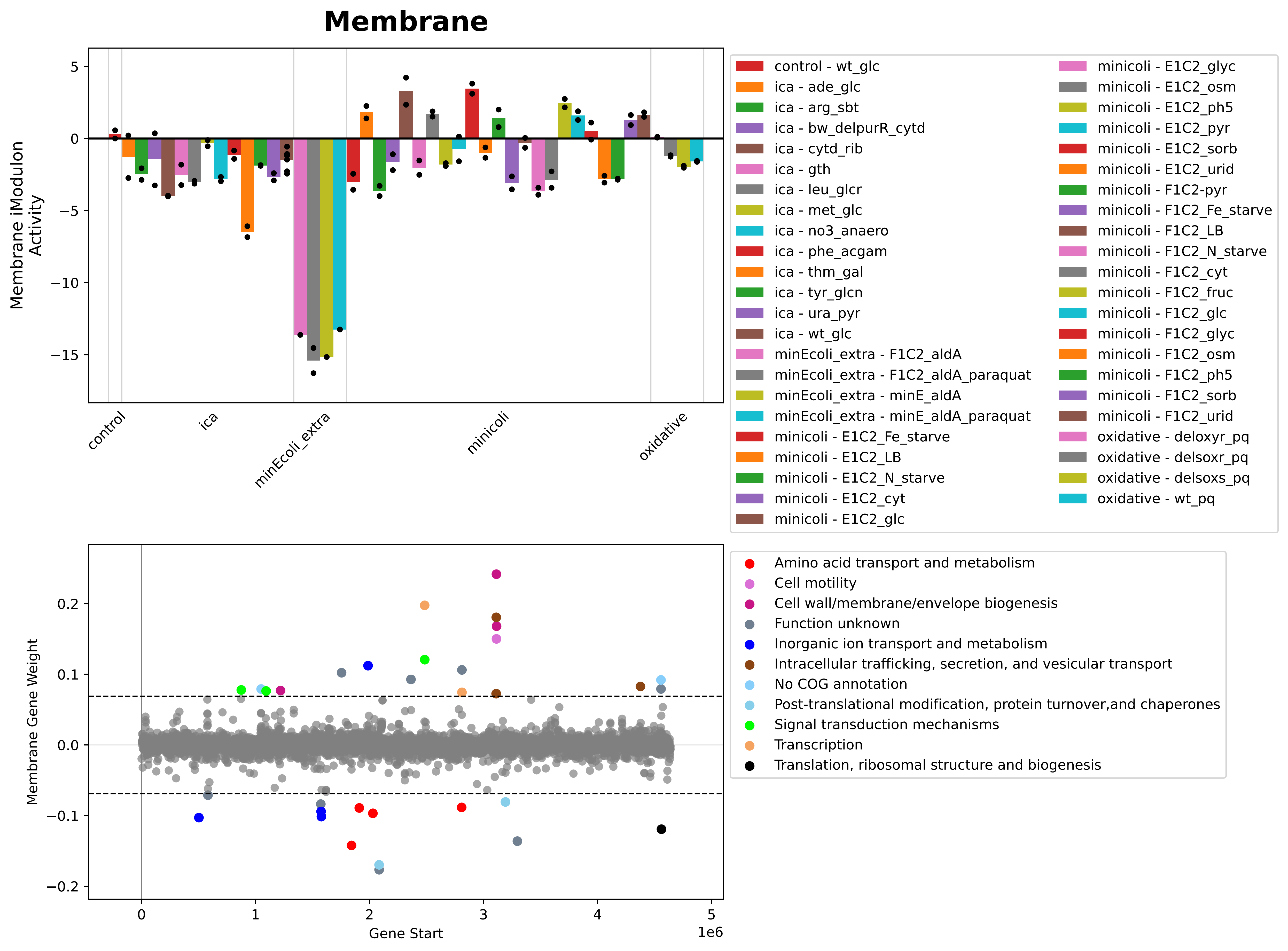

Membrane
Membrane iModulon Activity
control
ica
minEcoli_extra
minicoli
oxidative
control - wt_glc
ica - ade_glc
ica - arg_sbt
ica - bw_delpurR_cytd
ica - cytd_rib
ica - gth
ica - leu_glcr
ica - met_glc
ica - no3_anaero
ica - phe_acgam
ica - thm_gal
ica - tyr_glcn
ica - ura_pyr
ica - wt_glc
minEcoli_extra - F1C2_aldA
minEcoli_extra - F1C2_aldA_paraquat
minEcoli_extra - minE_aldA
minEcoli_extra - minE_aldA_paraquat
minicoli - E1C2_Fe_starve
minicoli - E1C2_LB
minicoli - E1C2_N_starve
minicoli - E1C2_cyt
minicoli - E1C2_glc
minicoli - E1C2_glyc
minicoli - E1C2_osm
minicoli - E1C2_ph5
minicoli - E1C2_pyr
minicoli - E1C2_sorb
minicoli - E1C2_urid
minicoli - F1C2-pyr
minicoli - F1C2_Fe_starve
minicoli - F1C2_LB
minicoli - F1C2_N_starve
minicoli - F1C2_cyt
minicoli - F1C2_fruc
minicoli - F1C2_glc
minicoli - F1C2_glyc
minicoli - F1C2_osm
minicoli - F1C2_ph5
minicoli - F1C2_sorb
minicoli - F1C2_urid
oxidative - deloxyr_pq
oxidative - delsoxr_pq
oxidative - delsoxs_pq
oxidative - wt_pq
Membrane Gene Weight
Gene Start
1e6
Amino acid transport and metabolism
Cell motility
Cell wall/membrane/envelope biogenesis
Function unknown
Inorganic ion transport and metabolism
Intracellular trafficking, secretion, and vesicular transport
No COG annotation
Post-translational modification, protein turnover,and chaperones
Signal transduction mechanisms
Transcription
Translation, ribosomal structure and biogenesis

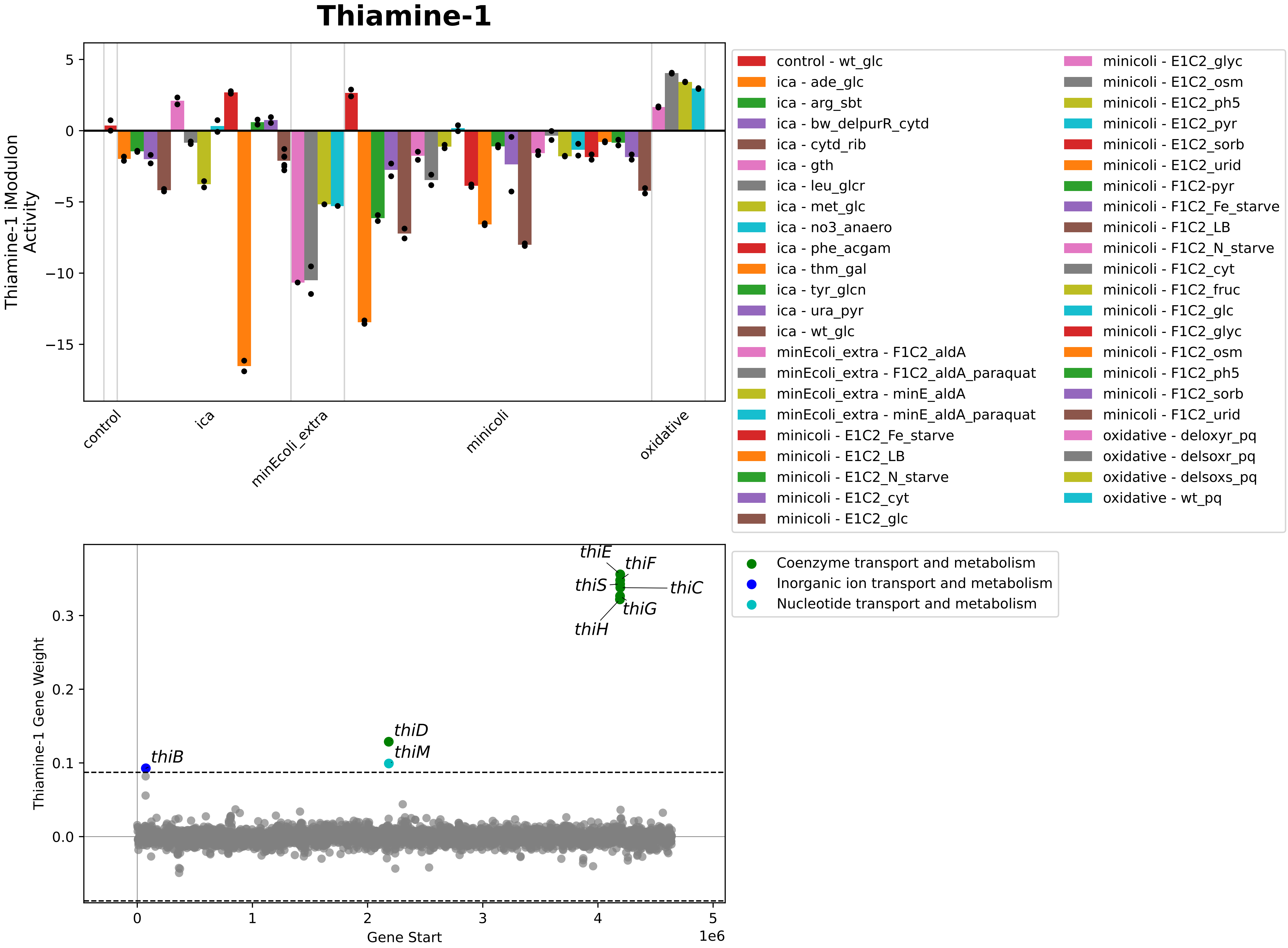
Thiamine-1
Thiamine-1 iModulon Activity
control
ica
minEcoli_extra
minicoli
oxidative
control - wt_glc
ica - ade_glc
ica - arg_sbt
ica - bw_delpurR_cytd
ica - cytd_rib
ica - gth
ica - leu_glcr
ica - met_glc
ica - no3_anaero
ica - phe_acgam
ica - thm_gal
ica - tyr_glcn
ica - ura_pyr
ica - wt_glc
minEcoli_extra - F1C2_aldA
minEcoli_extra - F1C2_aldA_paraquat
minEcoli_extra - minE_aldA
minEcoli_extra - minE_aldA_paraquat
minicoli - E1C2_Fe_starve
minicoli - E1C2_LB
minicoli - E1C2_N_starve
minicoli - E1C2_cyt
minicoli - E1C2_glc
minicoli - E1C2_glyc
minicoli - E1C2_osm
minicoli - E1C2_ph5
minicoli - E1C2_pyr
minicoli - E1C2_sorb
minicoli - E1C2_urid
minicoli - F1C2-pyr
minicoli - F1C2_Fe_starve
minicoli - F1C2_LB
minicoli - F1C2_N_starve
minicoli - F1C2_cyt
minicoli - F1C2_fruc
minicoli - F1C2_glc
minicoli - F1C2_glyc
minicoli - F1C2_osm
minicoli - F1C2_ph5
minicoli - F1C2_sorb
minicoli - F1C2_urid
oxidative - deloxyr_pq
oxidative - delsoxr_pq
oxidative - delsoxs_pq
oxidative - wt_pq
Thiamine-1 Gene Weight
Gene Start
1e6
thiE
thiF
thiS
thiC
thiG
thiH
thiD
thiM
thiB
Coenzyme transport and metabolism
Inorganic ion transport and metabolism
Nucleotide transport and metabolism

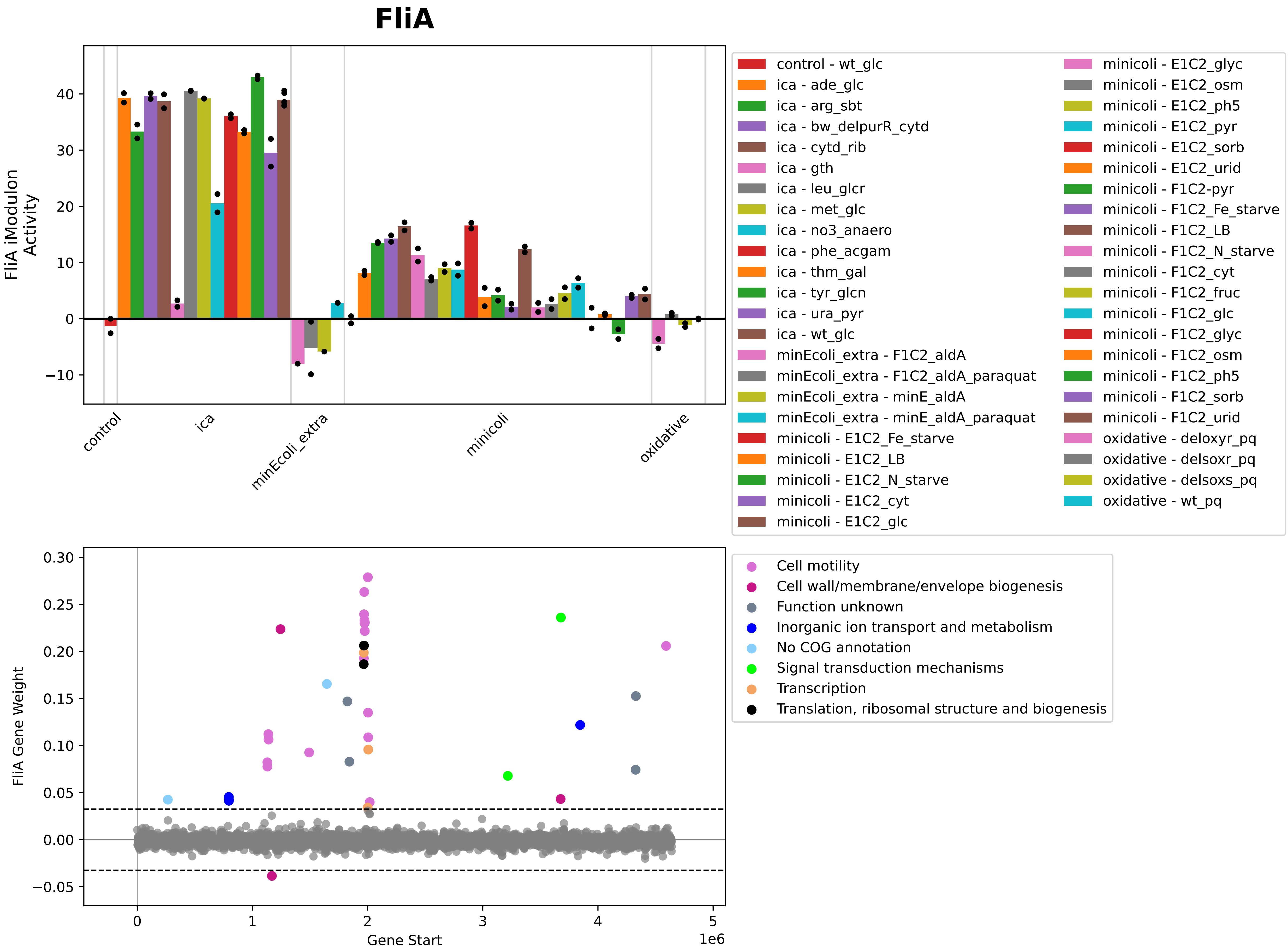

FliA
FliA iModulon Activity
control
ica
minEcoli_extra
minicoli
oxidative
control - wt_glc
ica - ade_glc
ica - arg_sbt
ica - bw_delpurR_cytd
ica - cytd_rib
ica - gth
ica - leu_glcr
ica - met_glc
ica - no3_anaero
ica - phe_acgam
ica - thm_gal
ica - tyr_glcn
ica - ura_pyr
ica - wt_glc
minEcoli_extra - F1C2_aldA
minEcoli_extra - F1C2_aldA_paraquat
minEcoli_extra - minE_aldA
minEcoli_extra - minE_aldA_paraquat
minicoli - E1C2_Fe_starve
minicoli - E1C2_LB
minicoli - E1C2_N_starve
minicoli - E1C2_cyt
minicoli - E1C2_glc
minicoli - E1C2_glyc
minicoli - E1C2_osm
minicoli - E1C2_ph5
minicoli - E1C2_pyr
minicoli - E1C2_sorb
minicoli - E1C2_urid
minicoli - F1C2-pyr
minicoli - F1C2_Fe_starve
minicoli - F1C2_LB
minicoli - F1C2_N_starve
minicoli - F1C2_cyt
minicoli - F1C2_fruc
minicoli - F1C2_glc
minicoli - F1C2_glyc
minicoli - F1C2_osm
minicoli - F1C2_ph5
minicoli - F1C2_sorb
minicoli - F1C2_urid
oxidative - deloxyr_pq
oxidative - delsoxr_pq
oxidative - delsoxs_pq
oxidative - wt_pq
FliA Gene Weight
Gene Start
1e6
Cell motility
Cell wall/membrane/envelope biogenesis
Function unknown
Inorganic ion transport and metabolism
No COG annotation
Signal transduction mechanisms
Transcription
Translation, ribosomal structure and biogenesis

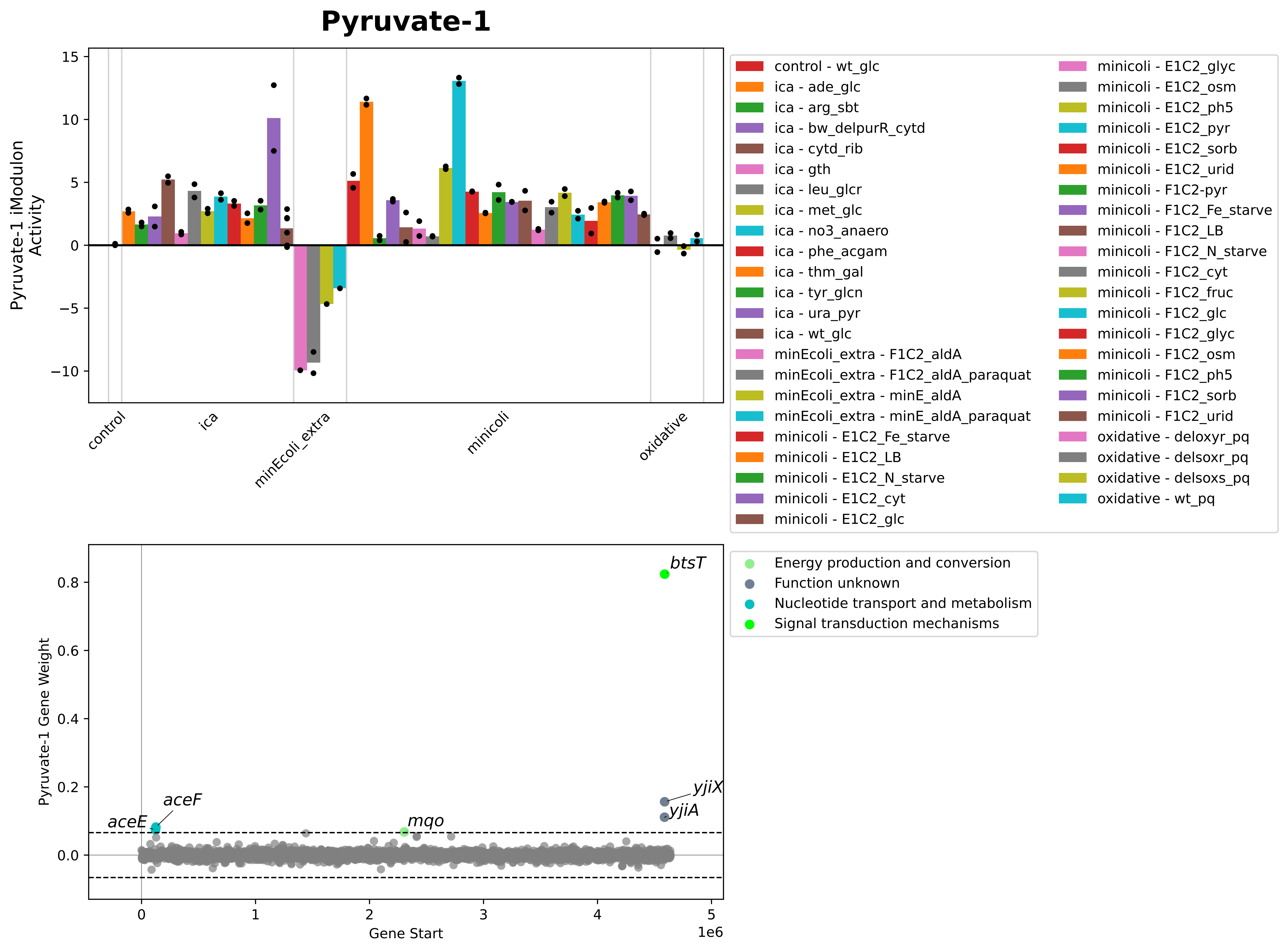

Pyruvate-1
Pyruvate-1 iModulon Activity
control
ica
minEcoli_extra
minicoli
oxidative
control - wt_glc
ica - ade_glc
ica - arg_sbt
ica - bw_delpurR_cytd
ica - cytd_rib
ica - gth
ica - leu_glcr
ica - met_glc
ica - no3_anaero
ica - phe_acgam
ica - thm_gal
ica - tyr_glcn
ica - ura_pyr
ica - wt_glc
minEcoli_extra - F1C2_aldA
minEcoli_extra - F1C2_aldA_paraquat
minEcoli_extra - minE_aldA
minEcoli_extra - minE_aldA_paraquat
minicoli - E1C2_Fe_starve
minicoli - E1C2_LB
minicoli - E1C2_N_starve
minicoli - E1C2_cyt
minicoli - E1C2_glc
minicoli - E1C2_glyc
minicoli - E1C2_osm
minicoli - E1C2_ph5
minicoli - E1C2_pyr
minicoli - E1C2_sorb
minicoli - E1C2_urid
minicoli - F1C2-pyr
minicoli - F1C2_Fe_starve
minicoli - F1C2_LB
minicoli - F1C2_N_starve
minicoli - F1C2_cyt
minicoli - F1C2_fruc
minicoli - F1C2_glc
minicoli - F1C2_glyc
minicoli - F1C2_osm
minicoli - F1C2_ph5
minicoli - F1C2_sorb
minicoli - F1C2_urid
oxidative - deloxyr_pq
oxidative - delsoxr_pq
oxidative - delsoxs_pq
oxidative - wt_pq
Pyruvate-1 Gene Weight
Gene Start
1e6
btsT
yjiX
yjiA
aceF
aceE
mqo
Energy production and conversion
Function unknown
Nucleotide transport and metabolism
Signal transduction mechanisms

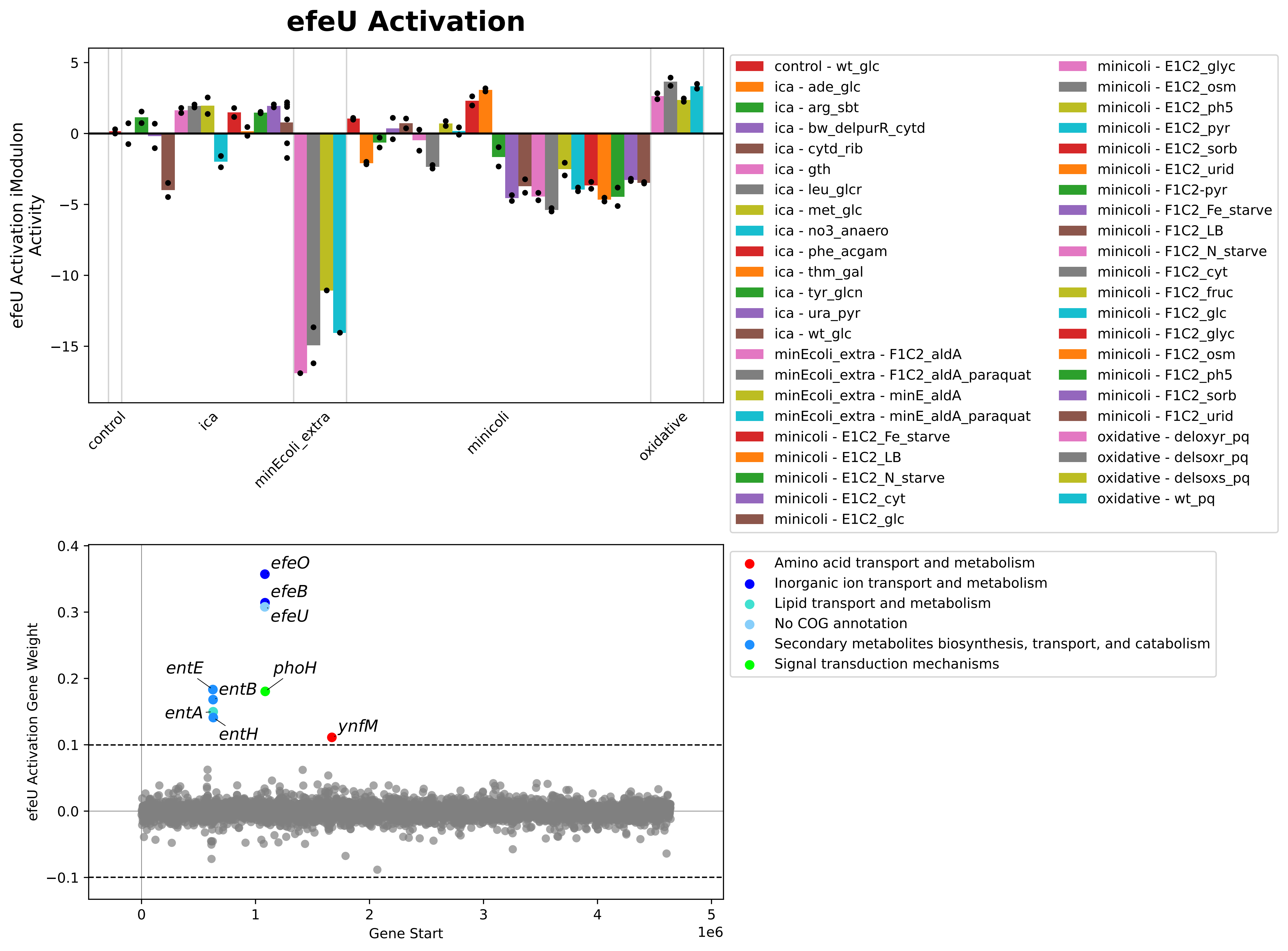
efeU Activation
efeU Activation iModulon Activity
control
ica
minEcoli_extra
minicoli
oxidative
control - wt_glc
ica - ade_glc
ica - arg_sbt
ica - bw_delpurR_cytd
ica - cytd_rib
ica - gth
ica - leu_glcr
ica - met_glc
ica - no3_anaero
ica - phe_acgam
ica - thm_gal
ica - tyr_glcn
ica - ura_pyr
ica - wt_glc
minEcoli_extra - F1C2_aldA
minEcoli_extra - F1C2_aldA_paraquat
minEcoli_extra - minE_aldA
minEcoli_extra - minE_aldA_paraquat
minicoli - E1C2_Fe_starve
minicoli - E1C2_LB
minicoli - E1C2_N_starve
minicoli - E1C2_cyt
minicoli - E1C2_glc
minicoli - E1C2_glyc
minicoli - E1C2_osm
minicoli - E1C2_ph5
minicoli - E1C2_pyr
minicoli - E1C2_sorb
minicoli - E1C2_urid
minicoli - F1C2-pyr
minicoli - F1C2_Fe_starve
minicoli - F1C2_LB
minicoli - F1C2_N_starve
minicoli - F1C2_cyt
minicoli - F1C2_fruc
minicoli - F1C2_glc
minicoli - F1C2_glyc
minicoli - F1C2_osm
minicoli - F1C2_ph5
minicoli - F1C2_sorb
minicoli - F1C2_urid
oxidative - deloxyr_pq
oxidative - delsoxr_pq
oxidative - delsoxs_pq
oxidative - wt_pq
efeU Activation Gene Weight
Gene Start
1e6
efeO
efeB
efeU
entE
entB
phoH
entA
entH
ynfM
Amino acid transport and metabolism
Inorganic ion transport and metabolism
Lipid transport and metabolism
No COG annotation
Secondary metabolites biosynthesis, transport, and catabolism
Signal transduction mechanisms

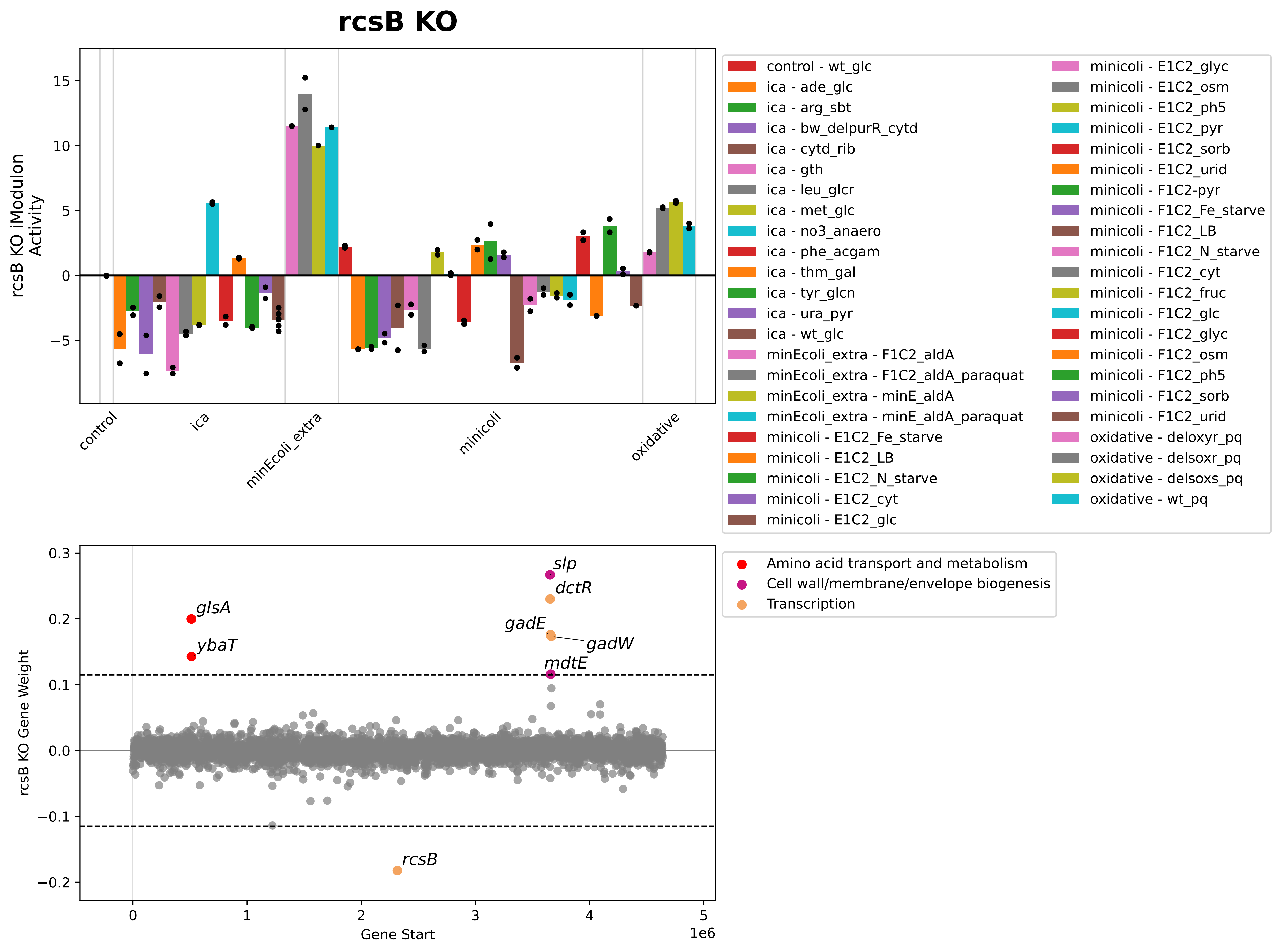
rcsB KO
rcsB KO iModulon Activity
control
ica
minEcoli_extra
minicoli
oxidative
control - wt_glc
ica - ade_glc
ica - arg_sbt
ica - bw_delpurR_cytd
ica - cytd_rib
ica - gth
ica - leu_glcr
ica - met_glc
ica - no3_anaero
ica - phe_acgam
ica - thm_gal
ica - tyr_glcn
ica - ura_pyr
ica - wt_glc
minEcoli_extra - F1C2_aldA
minEcoli_extra - F1C2_aldA_paraquat
minEcoli_extra - minE_aldA
minEcoli_extra - minE_aldA_paraquat
minicoli - E1C2_Fe_starve
minicoli - E1C2_LB
minicoli - E1C2_N_starve
minicoli - E1C2_cyt
minicoli - E1C2_glc
minicoli - E1C2_glyc
minicoli - E1C2_osm
minicoli - E1C2_ph5
minicoli - E1C2_pyr
minicoli - E1C2_sorb
minicoli - E1C2_urid
minicoli - F1C2-pyr
minicoli - F1C2_Fe_starve
minicoli - F1C2_LB
minicoli - F1C2_N_starve
minicoli - F1C2_cyt
minicoli - F1C2_fruc
minicoli - F1C2_glc
minicoli - F1C2_glyc
minicoli - F1C2_osm
minicoli - F1C2_ph5
minicoli - F1C2_sorb
minicoli - F1C2_urid
oxidative - deloxyr_pq
oxidative - delsoxr_pq
oxidative - delsoxs_pq
oxidative - wt_pq
rcsB KO Gene Weight
Gene Start
1e6
glsA
ybaT
slp
dctR
gadE
gadW
mdtE
rcsB
Amino acid transport and metabolism
Cell wall/membrane/envelope biogenesis
Transcription

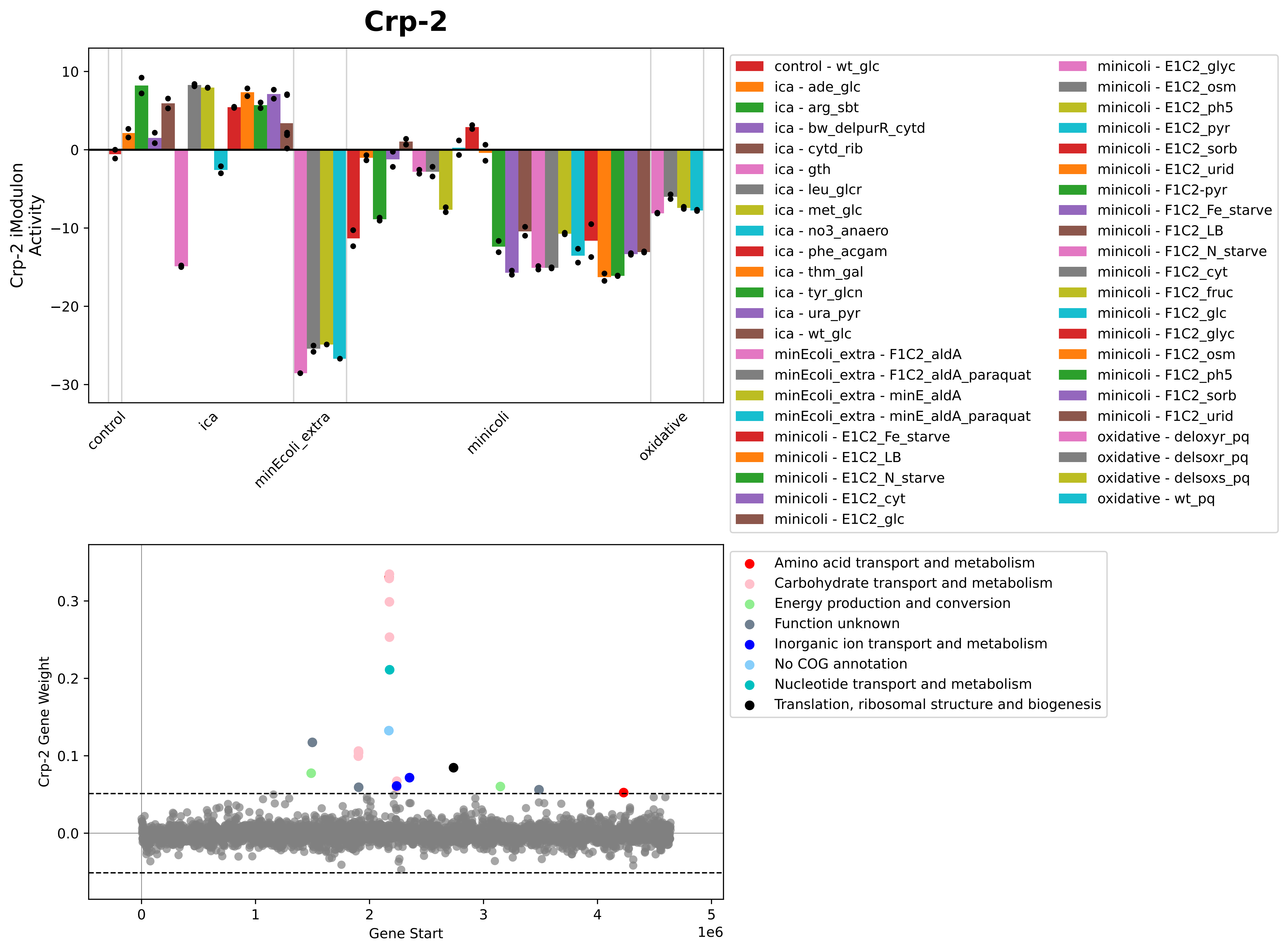
Crp-2
Crp-2 iModulon Activity
control
ica
minEcoli_extra
minicoli
oxidative
control - wt_glc
ica - ade_glc
ica - arg_sbt
ica - bw_delpurR_cytd
ica - cytd_rib
ica - gth
ica - leu_glcr
ica - met_glc
ica - no3_anaero
ica - phe_acgam
ica - thm_gal
ica - tyr_glcn
ica - ura_pyr
ica - wt_glc
minEcoli_extra - F1C2_aldA
minEcoli_extra - F1C2_aldA_paraquat
minEcoli_extra - minE_aldA
minEcoli_extra - minE_aldA_paraquat
minicoli - E1C2_Fe_starve
minicoli - E1C2_LB
minicoli - E1C2_N_starve
minicoli - E1C2_cyt
minicoli - E1C2_glc
minicoli - E1C2_glyc
minicoli - E1C2_osm
minicoli - E1C2_ph5
minicoli - E1C2_pyr
minicoli - E1C2_sorb
minicoli - E1C2_urid
minicoli - F1C2-pyr
minicoli - F1C2_Fe_starve
minicoli - F1C2_LB
minicoli - F1C2_N_starve
minicoli - F1C2_cyt
minicoli - F1C2_fruc
minicoli - F1C2_glc
minicoli - F1C2_glyc
minicoli - F1C2_osm
minicoli - F1C2_ph5
minicoli - F1C2_sorb
minicoli - F1C2_urid
oxidative - deloxyr_pq
oxidative - delsoxr_pq
oxidative - delsoxs_pq
oxidative - wt_pq
Crp-2 Gene Weight
Gene Start
1e6
Amino acid transport and metabolism
Carbohydrate transport and metabolism
Energy production and conversion
Function unknown
Inorganic ion transport and metabolism
No COG annotation
Nucleotide transport and metabolism
Translation, ribosomal structure and biogenesis

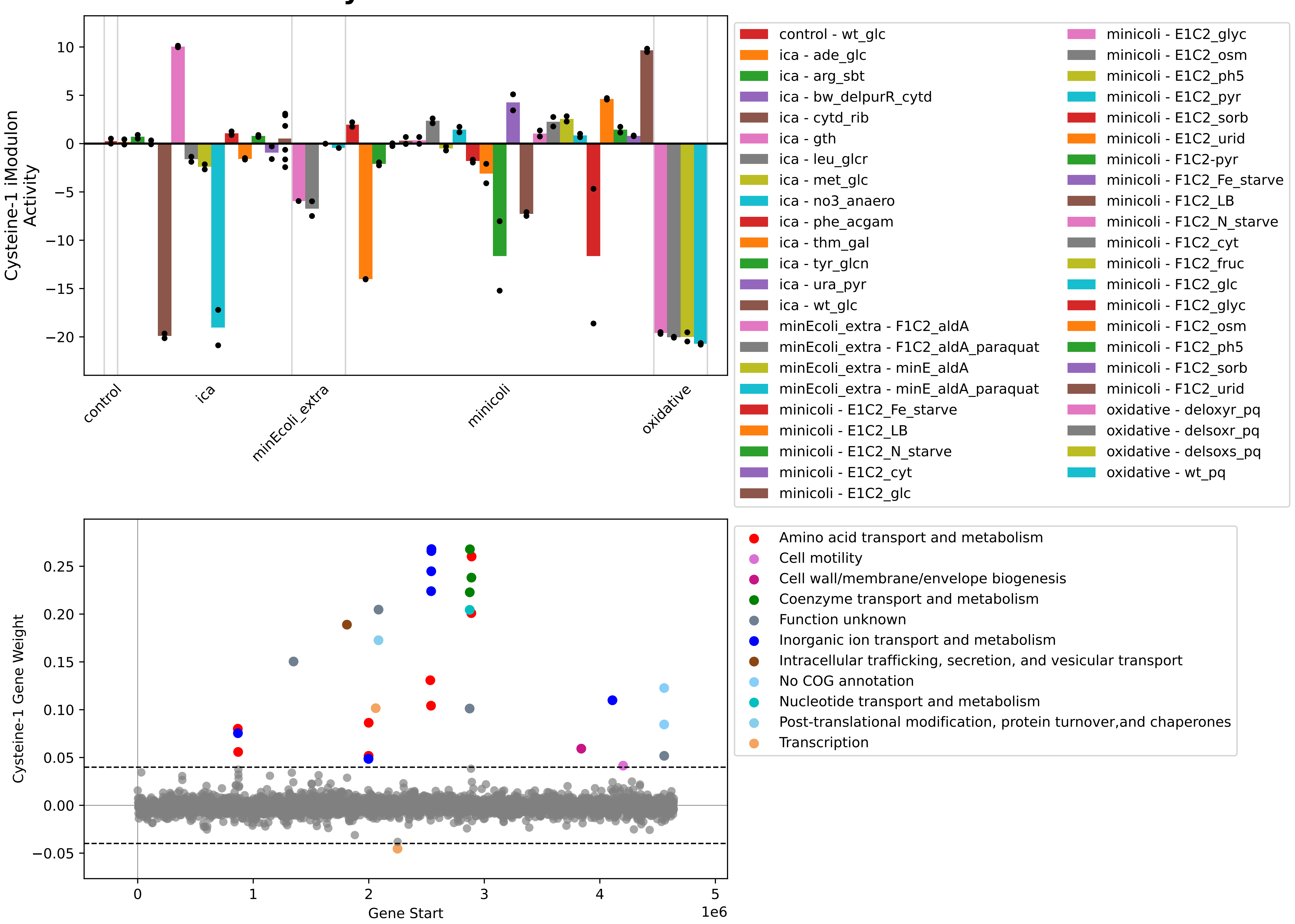
Cysteine-1
Cysteine-1 iModulon Activity
control
ica
minEcoli_extra
minicoli
oxidative
control - wt_glc
ica - ade_glc
ica - arg_sbt
ica - bw_delpurR_cytd
ica - cytd_rib
ica - gth
ica - leu_glcr
ica - met_glc
ica - no3_anaero
ica - phe_acgam
ica - thm_gal
ica - tyr_glcn
ica - ura_pyr
ica - wt_glc
minEcoli_extra - F1C2_aldA
minEcoli_extra - F1C2_aldA_paraquat
minEcoli_extra - minE_aldA
minEcoli_extra - minE_aldA_paraquat
minicoli - E1C2_Fe_starve
minicoli - E1C2_LB
minicoli - E1C2_N_starve
minicoli - E1C2_cyt
minicoli - E1C2_glc
minicoli - E1C2_glyc
minicoli - E1C2_osm
minicoli - E1C2_ph5
minicoli - E1C2_pyr
minicoli - E1C2_sorb
minicoli - E1C2_urid
minicoli - F1C2-pyr
minicoli - F1C2_Fe_starve
minicoli - F1C2_LB
minicoli - F1C2_N_starve
minicoli - F1C2_cyt
minicoli - F1C2_fruc
minicoli - F1C2_glc
minicoli - F1C2_glyc
minicoli - F1C2_osm
minicoli - F1C2_ph5
minicoli - F1C2_sorb
minicoli - F1C2_urid
oxidative - deloxyr_pq
oxidative - delsoxr_pq
oxidative - delsoxs_pq
oxidative - wt_pq
Cysteine-1 Gene Weight
Gene Start
1e6
Amino acid transport and metabolism
Cell motility
Cell wall/membrane/envelope biogenesis
Coenzyme transport and metabolism
Function unknown
Inorganic ion transport and metabolism
Intracellular trafficking, secretion, and vesicular transport
No COG annotation
Nucleotide transport and metabolism
Post-translational modification, protein turnover,and chaperones
Transcription

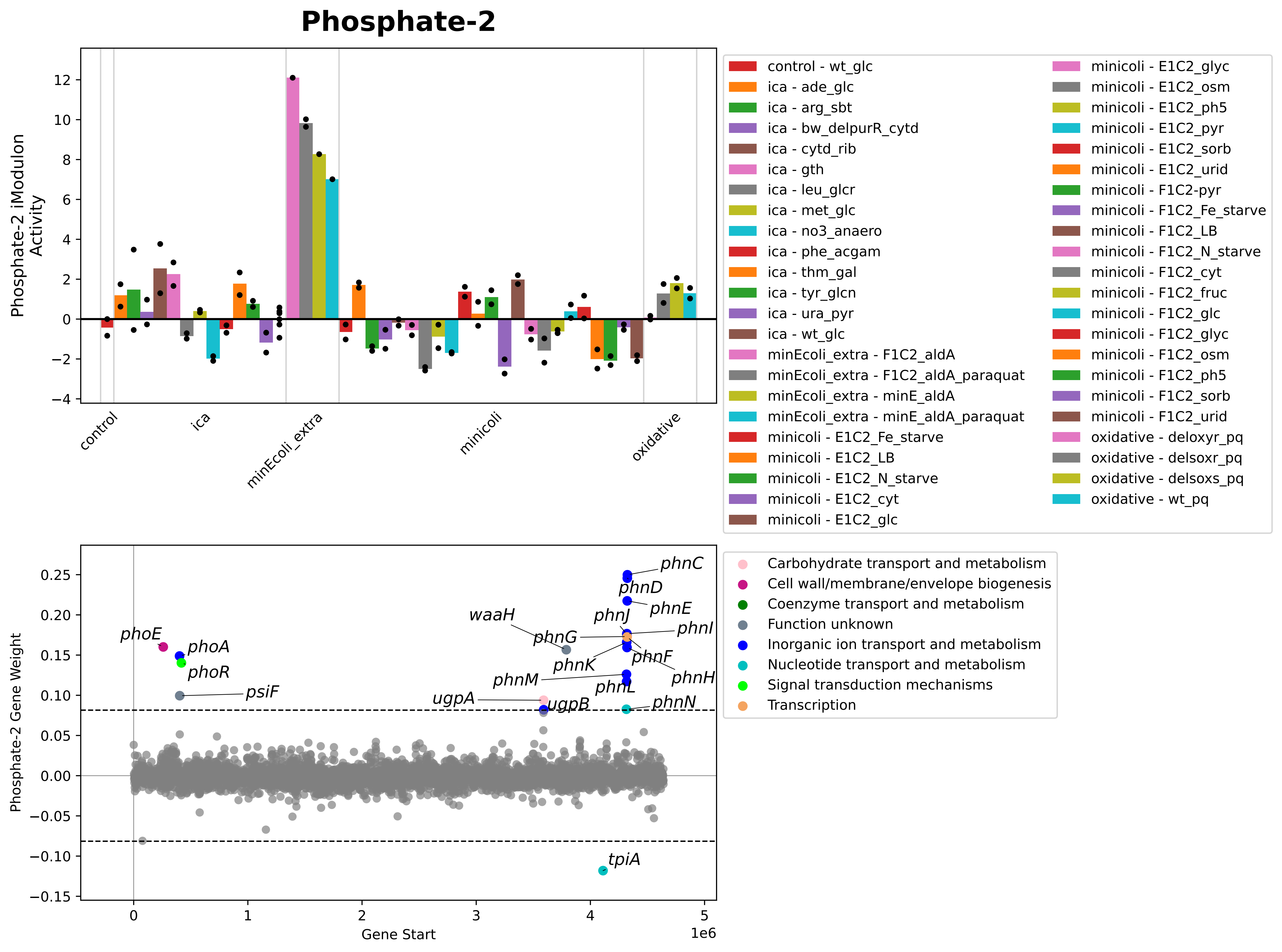
Phosphate-2
Phosphate-2 iModulon Activity
control
ica
minEcoli_extra
minicoli
oxidative
control - wt_glc
ica - ade_glc
ica - arg_sbt
ica - bw_delpurR_cytd
ica - cytd_rib
ica - gth
ica - leu_glcr
ica - met_glc
ica - no3_anaero
ica - phe_acgam
ica - thm_gal
ica - tyr_glcn
ica - ura_pyr
ica - wt_glc
minEcoli_extra - F1C2_aldA
minEcoli_extra - F1C2_aldA_paraquat
minEcoli_extra - minE_aldA
minEcoli_extra - minE_aldA_paraquat
minicoli - E1C2_Fe_starve
minicoli - E1C2_LB
minicoli - E1C2_N_starve
minicoli - E1C2_cyt
minicoli - E1C2_glc
minicoli - E1C2_glyc
minicoli - E1C2_osm
minicoli - E1C2_ph5
minicoli - E1C2_pyr
minicoli - E1C2_sorb
minicoli - E1C2_urid
minicoli - F1C2-pyr
minicoli - F1C2_Fe_starve
minicoli - F1C2_LB
minicoli - F1C2_N_starve
minicoli - F1C2_cyt
minicoli - F1C2_fruc
minicoli - F1C2_glc
minicoli - F1C2_glyc
minicoli - F1C2_osm
minicoli - F1C2_ph5
minicoli - F1C2_sorb
minicoli - F1C2_urid
oxidative - deloxyr_pq
oxidative - delsoxr_pq
oxidative - delsoxs_pq
oxidative - wt_pq
Phosphate-2 Gene Weight
Gene Start
1e6
phoE
phoA
phoR
psiF
waaH
phnG
phnK
phnM
ugpA
ugpB
phnC
phnD
phnE
phnJ
phnI
phnF
phnH
phnL
phnN
tpiA
Carbohydrate transport and metabolism
Cell wall/membrane/envelope biogenesis
Coenzyme transport and metabolism
Function unknown
Inorganic ion transport and metabolism
Nucleotide transport and metabolism
Signal transduction mechanisms
Transcription

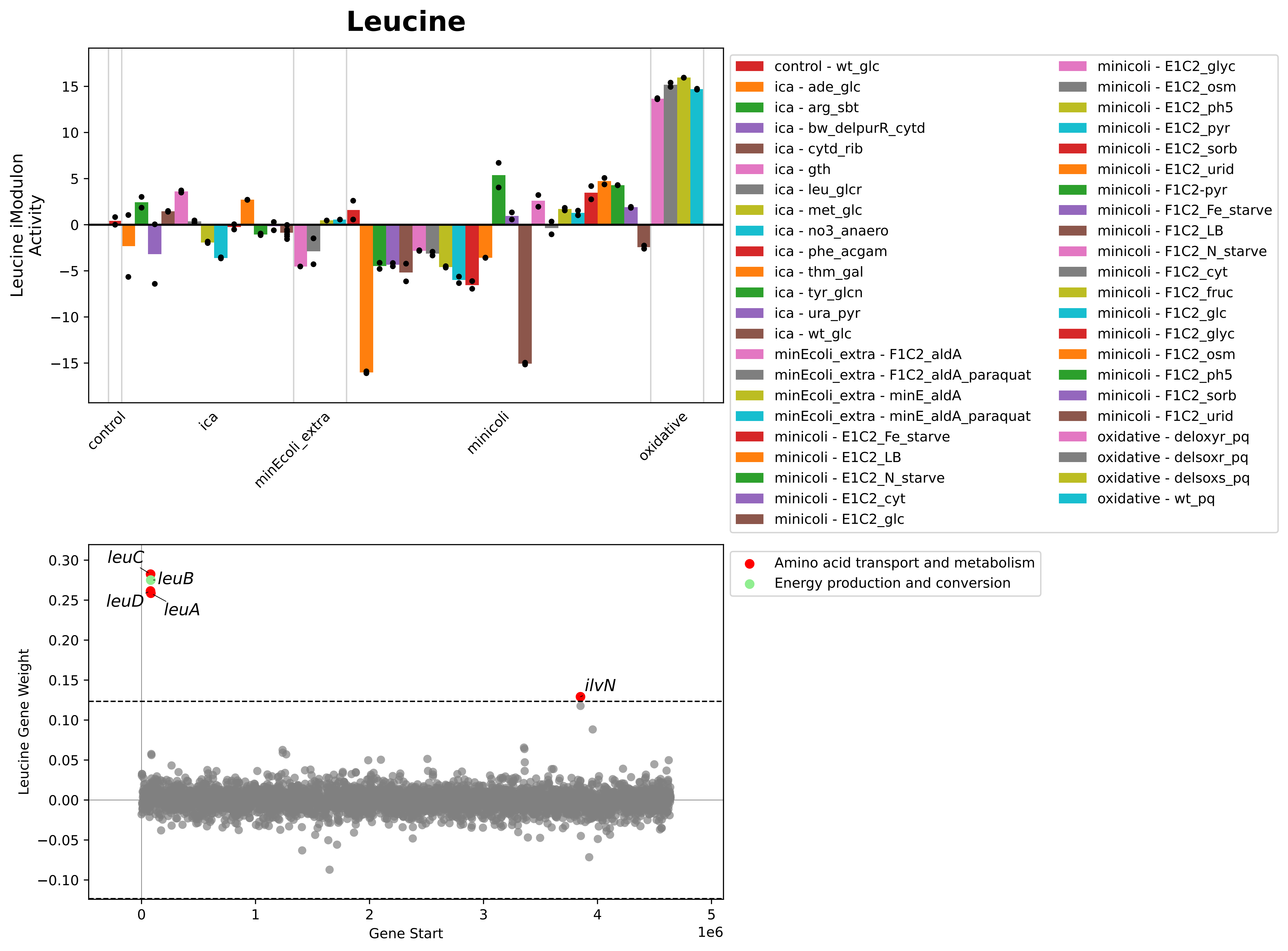
Leucine
Leucine iModulon Activity
control
ica
minEcoli_extra
minicoli
oxidative
15
10
5
0
−5
−10
−15
control - wt_glc
ica - ade_glc
ica - arg_sbt
ica - bw_delpurR_cytd
ica - cytd_rib
ica - gth
ica - leu_glcr
ica - met_glc
ica - no3_anaero
ica - phe_acgam
ica - thm_gal
ica - tyr_glcn
ica - ura_pyr
ica - wt_glc
minEcoli_extra - F1C2_aldA
minEcoli_extra - F1C2_aldA_paraquat
minEcoli_extra - minE_aldA
minEcoli_extra - minE_aldA_paraquat
minicoli - E1C2_Fe_starve
minicoli - E1C2_LB
minicoli - E1C2_N_starve
minicoli - E1C2_cyt
minicoli - E1C2_glc
minicoli - E1C2_glyc
minicoli - E1C2_osm
minicoli - E1C2_ph5
minicoli - E1C2_pyr
minicoli - E1C2_sorb
minicoli - E1C2_urid
minicoli - F1C2-pyr
minicoli - F1C2_Fe_starve
minicoli - F1C2_LB
minicoli - F1C2_N_starve
minicoli - F1C2_cyt
minicoli - F1C2_fruc
minicoli - F1C2_glc
minicoli - F1C2_glyc
minicoli - F1C2_osm
minicoli - F1C2_ph5
minicoli - F1C2_sorb
minicoli - F1C2_urid
oxidative - deloxyr_pq
oxidative - delsoxr_pq
oxidative - delsoxs_pq
oxidative - wt_pq
Leucine Gene Weight
leuC
leuB
leuD
leuA
ilvN
0.30
0.25
0.20
0.15
0.10
0.05
0.00
−0.05
−0.10
0
1
2
3
4
5
1e6
Gene Start
Amino acid transport and metabolism
Energy production and conversion

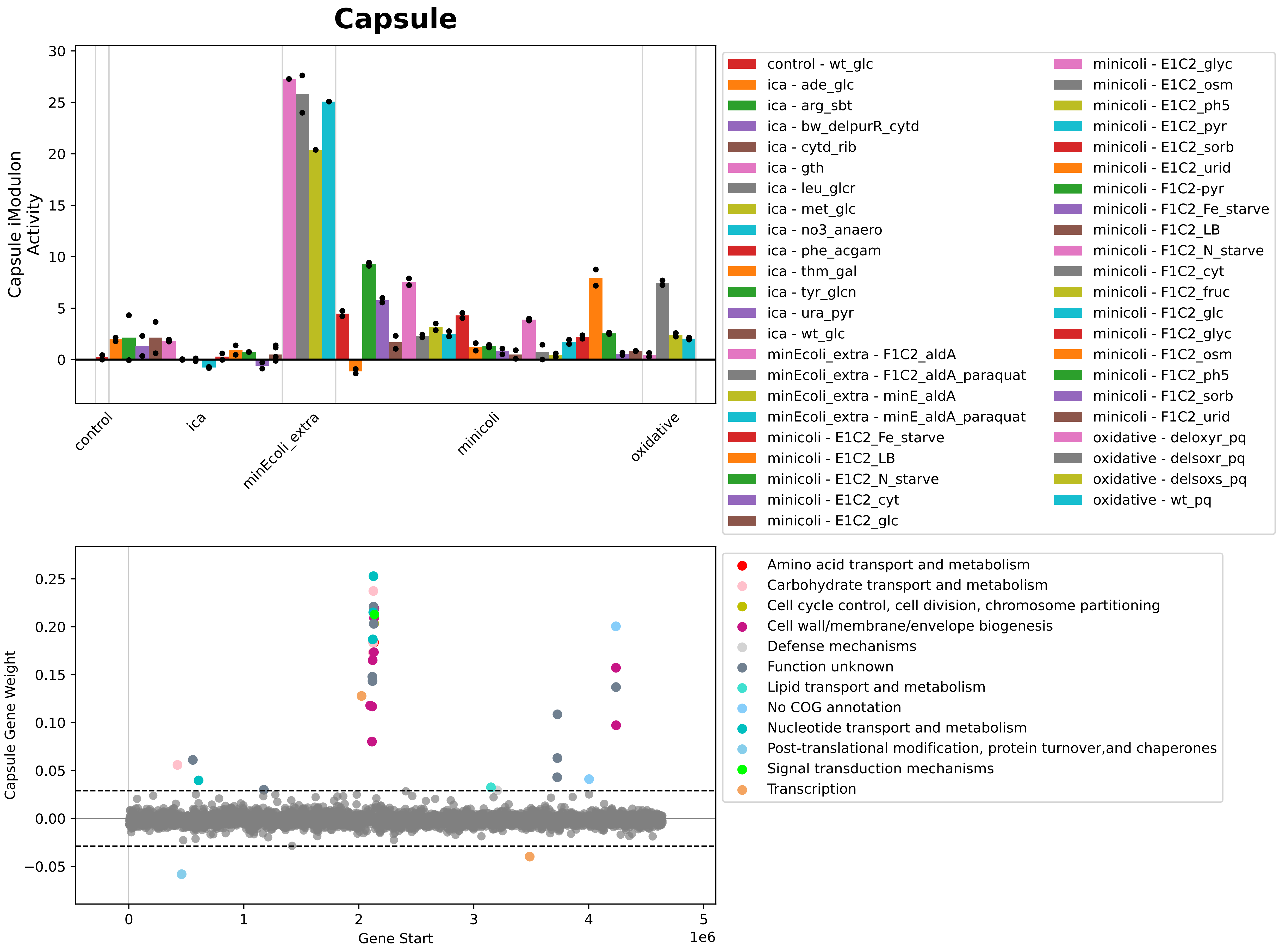
Capsule
Capsule iModulon Activity
control
ica
minEcoli_extra
minicoli
oxidative
control - wt_glc
ica - ade_glc
ica - arg_sbt
ica - bw_delpurR_cytd
ica - cytd_rib
ica - gth
ica - leu_glcr
ica - met_glc
ica - no3_anaero
ica - phe_acgam
ica - thm_gal
ica - tyr_glcn
ica - ura_pyr
ica - wt_glc
minEcoli_extra - F1C2_aldA
minEcoli_extra - F1C2_aldA_paraquat
minEcoli_extra - minE_aldA
minEcoli_extra - minE_aldA_paraquat
minicoli - E1C2_Fe_starve
minicoli - E1C2_LB
minicoli - E1C2_N_starve
minicoli - E1C2_cyt
minicoli - E1C2_glc
minicoli - E1C2_glyc
minicoli - E1C2_osm
minicoli - E1C2_ph5
minicoli - E1C2_pyr
minicoli - E1C2_sorb
minicoli - E1C2_urid
minicoli - F1C2-pyr
minicoli - F1C2_Fe_starve
minicoli - F1C2_LB
minicoli - F1C2_N_starve
minicoli - F1C2_cyt
minicoli - F1C2_fruc
minicoli - F1C2_glc
minicoli - F1C2_glyc
minicoli - F1C2_osm
minicoli - F1C2_ph5
minicoli - F1C2_sorb
minicoli - F1C2_urid
oxidative - deloxyr_pq
oxidative - delsoxr_pq
oxidative - delsoxs_pq
oxidative - wt_pq
Capsule Gene Weight
Gene Start
1e6
Amino acid transport and metabolism
Carbohydrate transport and metabolism
Cell cycle control, cell division, chromosome partitioning
Cell wall/membrane/envelope biogenesis
Defense mechanisms
Function unknown
Lipid transport and metabolism
No COG annotation
Nucleotide transport and metabolism
Post-translational modification, protein turnover,and chaperones
Signal transduction mechanisms
Transcription

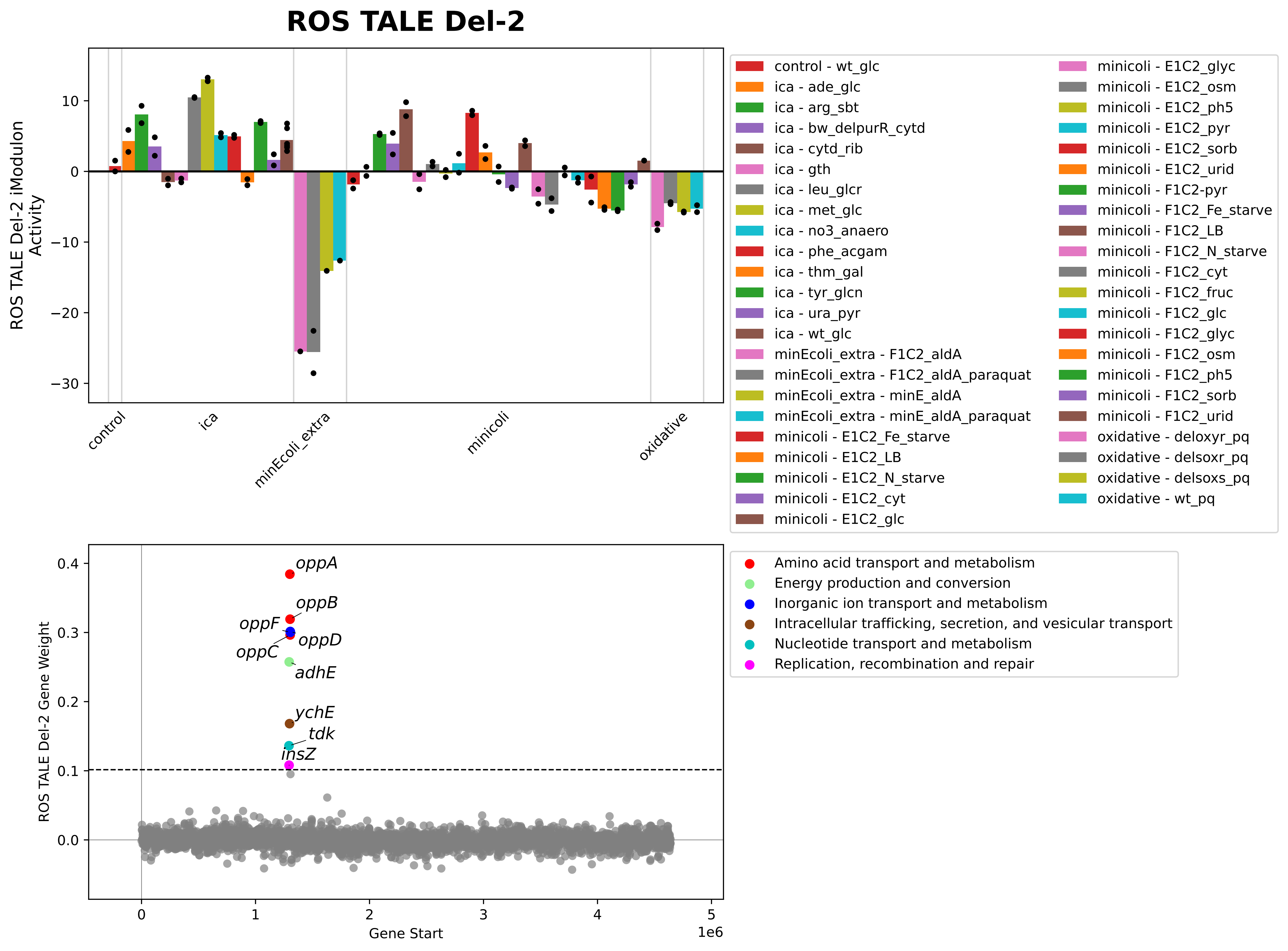

ROS TALE Del-2
ROS TALE Del-2 iModulon Activity
control
ica
minEcoli_extra
minicoli
oxidative
control - wt_glc
ica - ade_glc
ica - arg_sbt
ica - bw_delpurR_cytd
ica - cytd_rib
ica - gth
ica - leu_glcr
ica - met_glc
ica - no3_anaero
ica - phe_acgam
ica - thm_gal
ica - tyr_glcn
ica - ura_pyr
ica - wt_glc
minEcoli_extra - F1C2_aldA
minEcoli_extra - F1C2_aldA_paraquat
minEcoli_extra - minE_aldA
minEcoli_extra - minE_aldA_paraquat
minicoli - E1C2_Fe_starve
minicoli - E1C2_LB
minicoli - E1C2_N_starve
minicoli - E1C2_cyt
minicoli - E1C2_glc
minicoli - E1C2_glyc
minicoli - E1C2_osm
minicoli - E1C2_ph5
minicoli - E1C2_pyr
minicoli - E1C2_sorb
minicoli - E1C2_urid
minicoli - F1C2-pyr
minicoli - F1C2_Fe_starve
minicoli - F1C2_LB
minicoli - F1C2_N_starve
minicoli - F1C2_cyt
minicoli - F1C2_fruc
minicoli - F1C2_glc
minicoli - F1C2_glyc
minicoli - F1C2_osm
minicoli - F1C2_ph5
minicoli - F1C2_sorb
minicoli - F1C2_urid
oxidative - deloxyr_pq
oxidative - delsoxr_pq
oxidative - delsoxs_pq
oxidative - wt_pq
ROS TALE Del-2 Gene Weight
Gene Start
1e6
oppA
oppB
oppF
oppD
oppC
adhE
ychE
tdk
insZ
Amino acid transport and metabolism
Energy production and conversion
Inorganic ion transport and metabolism
Intracellular trafficking, secretion, and vesicular transport
Nucleotide transport and metabolism
Replication, recombination and repair

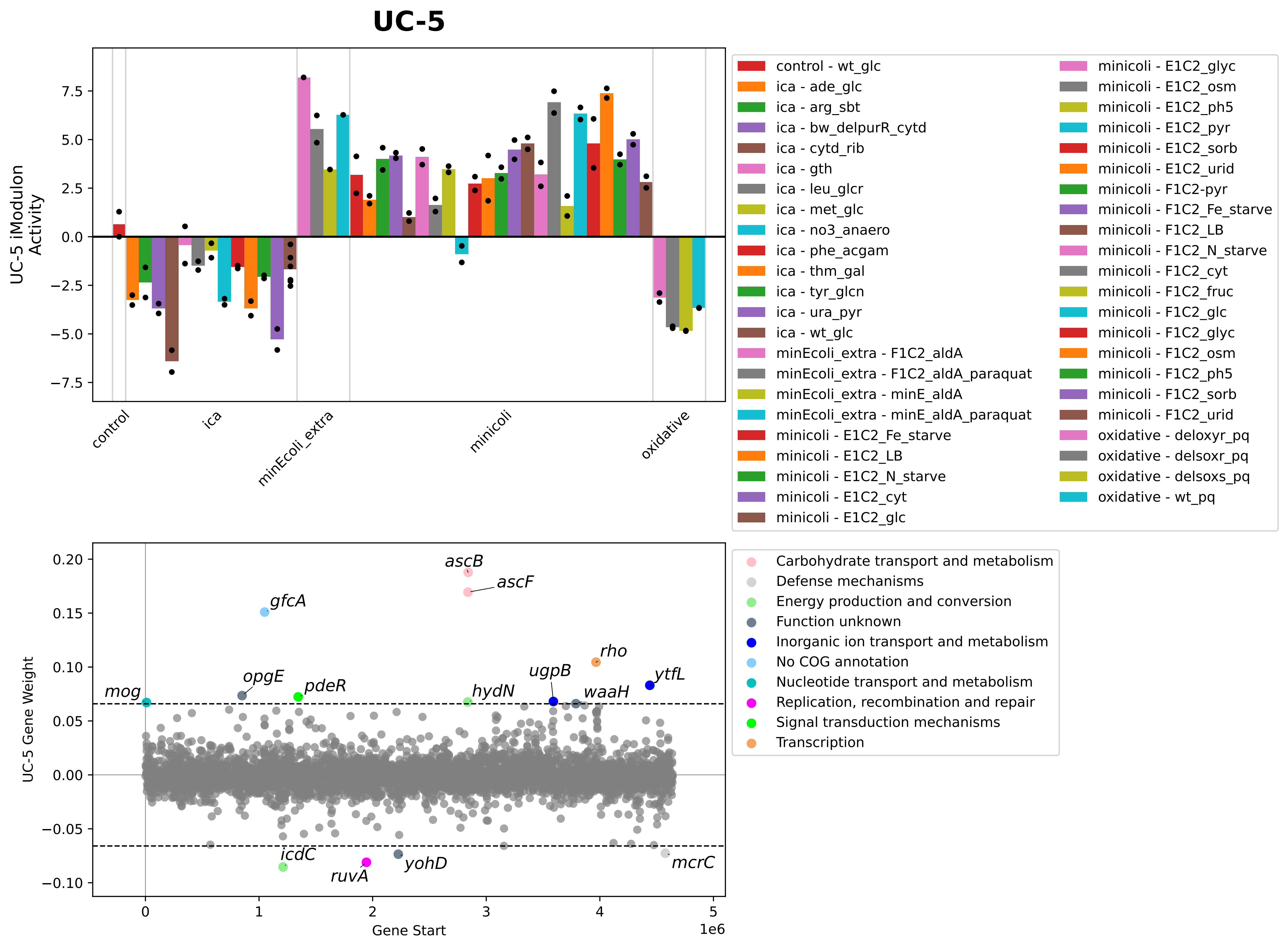
UC-5
UC-5 iModulon Activity
control
ica
minEcoli_extra
minicoli
oxidative
control - wt_glc
ica - ade_glc
ica - arg_sbt
ica - bw_delpurR_cytd
ica - cytd_rib
ica - gth
ica - leu_glcr
ica - met_glc
ica - no3_anaero
ica - phe_acgam
ica - thm_gal
ica - tyr_glcn
ica - ura_pyr
ica - wt_glc
minEcoli_extra - F1C2_aldA
minEcoli_extra - F1C2_aldA_paraquat
minEcoli_extra - minE_aldA
minEcoli_extra - minE_aldA_paraquat
minicoli - E1C2_Fe_starve
minicoli - E1C2_LB
minicoli - E1C2_N_starve
minicoli - E1C2_cyt
minicoli - E1C2_glc
minicoli - E1C2_glyc
minicoli - E1C2_osm
minicoli - E1C2_ph5
minicoli - E1C2_pyr
minicoli - E1C2_sorb
minicoli - E1C2_urid
minicoli - F1C2-pyr
minicoli - F1C2_Fe_starve
minicoli - F1C2_LB
minicoli - F1C2_N_starve
minicoli - F1C2_cyt
minicoli - F1C2_fruc
minicoli - F1C2_glc
minicoli - F1C2_glyc
minicoli - F1C2_osm
minicoli - F1C2_ph5
minicoli - F1C2_sorb
minicoli - F1C2_urid
oxidative - deloxyr_pq
oxidative - delsoxr_pq
oxidative - delsoxs_pq
oxidative - wt_pq
UC-5 Gene Weight
Gene Start
1e6
ascB
ascF
gfcA
rho
ugpB
ytfL
mog
opgE
pdeR
hydN
waaH
icdC
ruvA
yohD
mcrC
Carbohydrate transport and metabolism
Defense mechanisms
Energy production and conversion
Function unknown
Inorganic ion transport and metabolism
No COG annotation
Nucleotide transport and metabolism
Replication, recombination and repair
Signal transduction mechanisms
Transcription

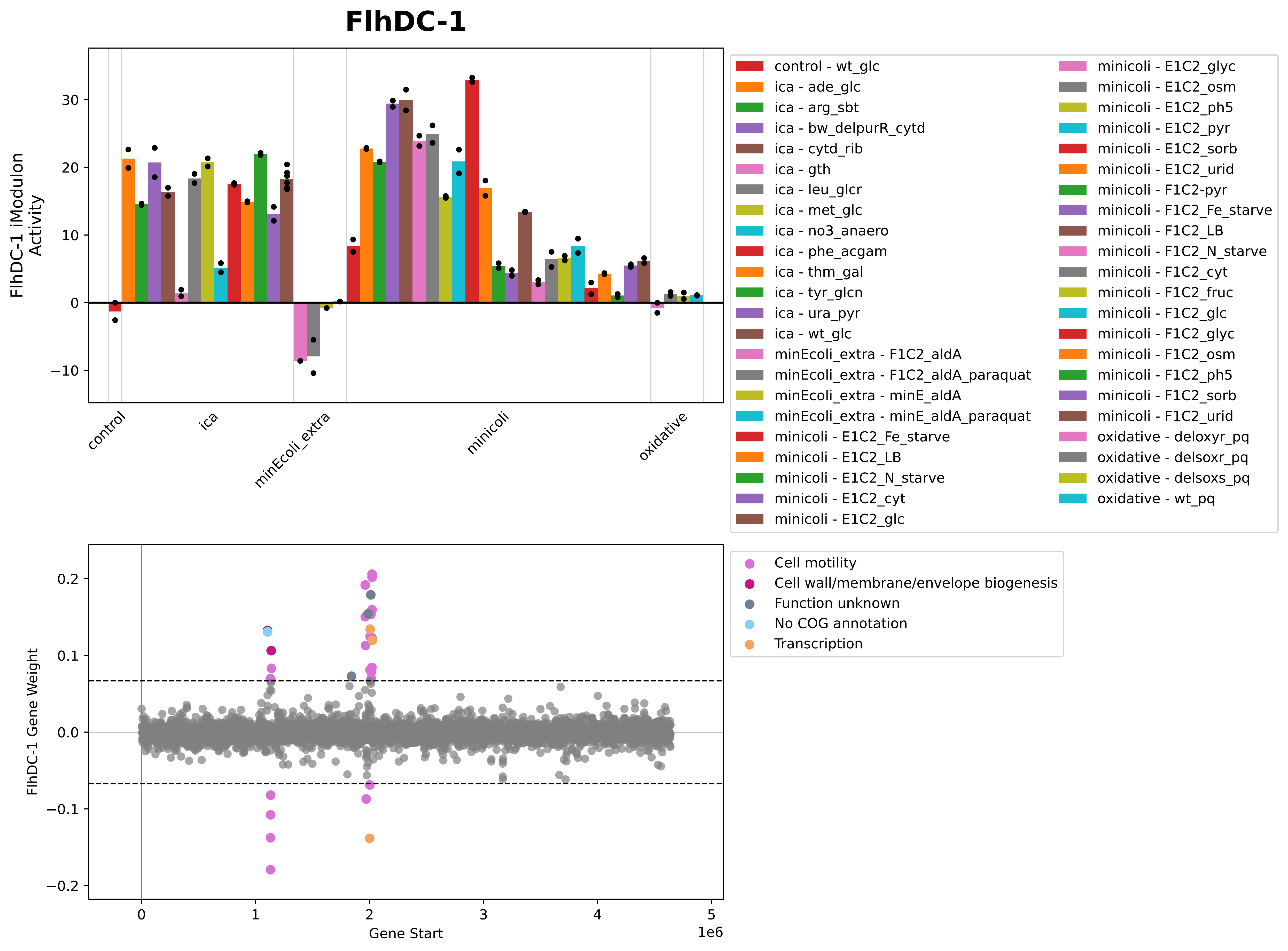
FlhDC-1
FlhDC-1 iModulon Activity
control
ica
minEcoli_extra
minicoli
oxidative
control - wt_glc
ica - ade_glc
ica - arg_sbt
ica - bw_delpurR_cytd
ica - cytd_rib
ica - gth
ica - leu_glcr
ica - met_glc
ica - no3_anaero
ica - phe_acgam
ica - thm_gal
ica - tyr_glcn
ica - ura_pyr
ica - wt_glc
minEcoli_extra - F1C2_aldA
minEcoli_extra - F1C2_aldA_paraquat
minEcoli_extra - minE_aldA
minEcoli_extra - minE_aldA_paraquat
minicoli - E1C2_Fe_starve
minicoli - E1C2_LB
minicoli - E1C2_N_starve
minicoli - E1C2_cyt
minicoli - E1C2_glc
minicoli - E1C2_glyc
minicoli - E1C2_osm
minicoli - E1C2_ph5
minicoli - E1C2_pyr
minicoli - E1C2_sorb
minicoli - E1C2_urid
minicoli - F1C2-pyr
minicoli - F1C2_Fe_starve
minicoli - F1C2_LB
minicoli - F1C2_N_starve
minicoli - F1C2_cyt
minicoli - F1C2_fruc
minicoli - F1C2_glc
minicoli - F1C2_glyc
minicoli - F1C2_osm
minicoli - F1C2_ph5
minicoli - F1C2_sorb
minicoli - F1C2_urid
oxidative - deloxyr_pq
oxidative - delsoxr_pq
oxidative - delsoxs_pq
oxidative - wt_pq
FlhDC-1 Gene Weight
Gene Start
1e6
Cell motility
Cell wall/membrane/envelope biogenesis
Function unknown
No COG annotation
Transcription

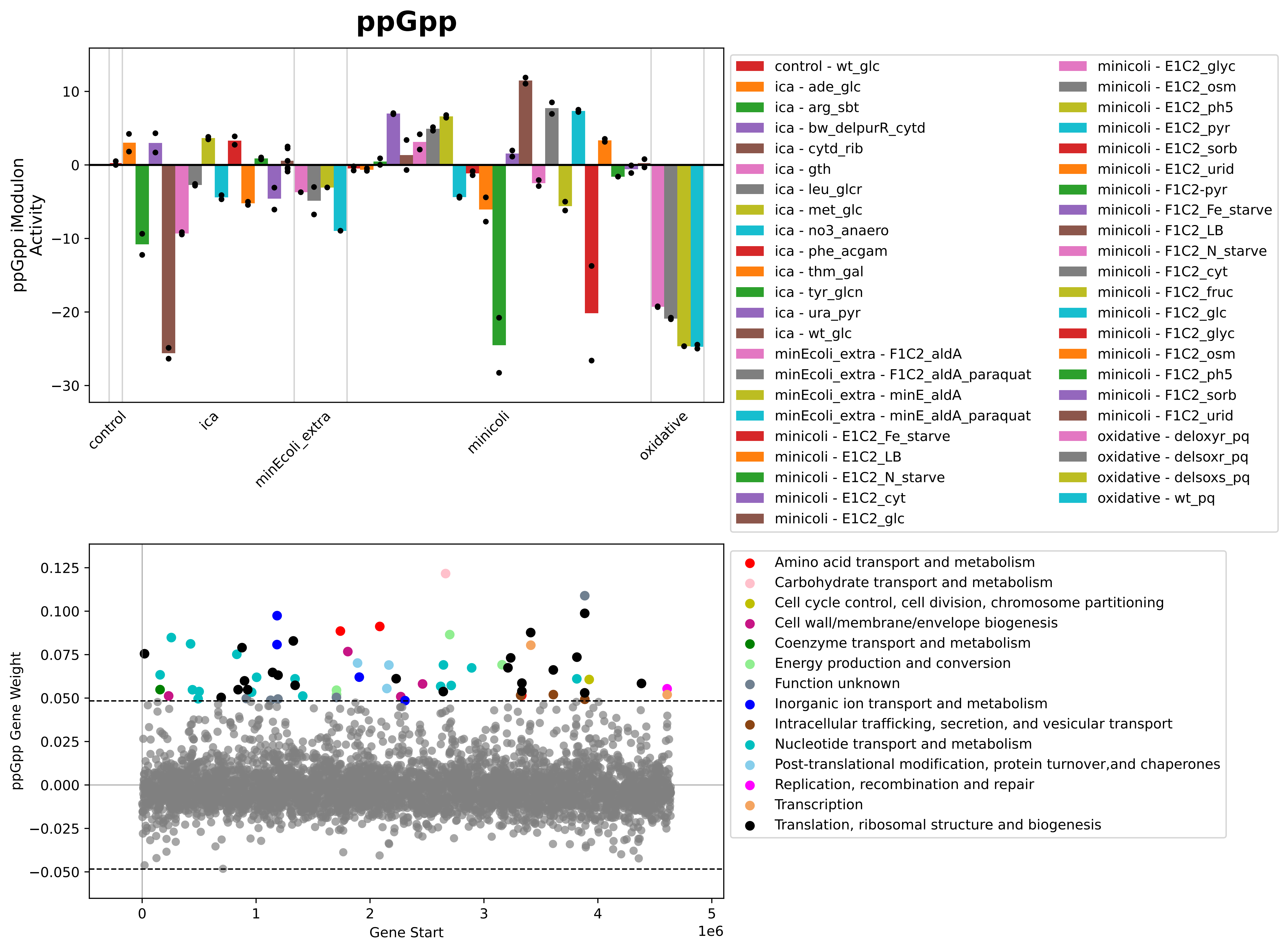
ppGpp
ppGpp iModulon Activity
control
ica
minEcoli_extra
minicoli
oxidative
control - wt_glc
ica - ade_glc
ica - arg_sbt
ica - bw_delpurR_cytd
ica - cytd_rib
ica - gth
ica - leu_glcr
ica - met_glc
ica - no3_anaero
ica - phe_acgam
ica - thm_gal
ica - tyr_glcn
ica - ura_pyr
ica - wt_glc
minEcoli_extra - F1C2_aldA
minEcoli_extra - F1C2_aldA_paraquat
minEcoli_extra - minE_aldA
minEcoli_extra - minE_aldA_paraquat
minicoli - E1C2_Fe_starve
minicoli - E1C2_LB
minicoli - E1C2_N_starve
minicoli - E1C2_cyt
minicoli - E1C2_glc
minicoli - E1C2_glyc
minicoli - E1C2_osm
minicoli - E1C2_ph5
minicoli - E1C2_pyr
minicoli - E1C2_sorb
minicoli - E1C2_urid
minicoli - F1C2-pyr
minicoli - F1C2_Fe_starve
minicoli - F1C2_LB
minicoli - F1C2_N_starve
minicoli - F1C2_cyt
minicoli - F1C2_fruc
minicoli - F1C2_glc
minicoli - F1C2_glyc
minicoli - F1C2_osm
minicoli - F1C2_ph5
minicoli - F1C2_sorb
minicoli - F1C2_urid
oxidative - deloxyr_pq
oxidative - delsoxr_pq
oxidative - delsoxs_pq
oxidative - wt_pq
ppGpp Gene Weight
Gene Start
1e6
Amino acid transport and metabolism
Carbohydrate transport and metabolism
Cell cycle control, cell division, chromosome partitioning
Cell wall/membrane/envelope biogenesis
Coenzyme transport and metabolism
Energy production and conversion
Function unknown
Inorganic ion transport and metabolism
Intracellular trafficking, secretion, and vesicular transport
Nucleotide transport and metabolism
Post-translational modification, protein turnover,and chaperones
Replication, recombination and repair
Transcription
Translation, ribosomal structure and biogenesis

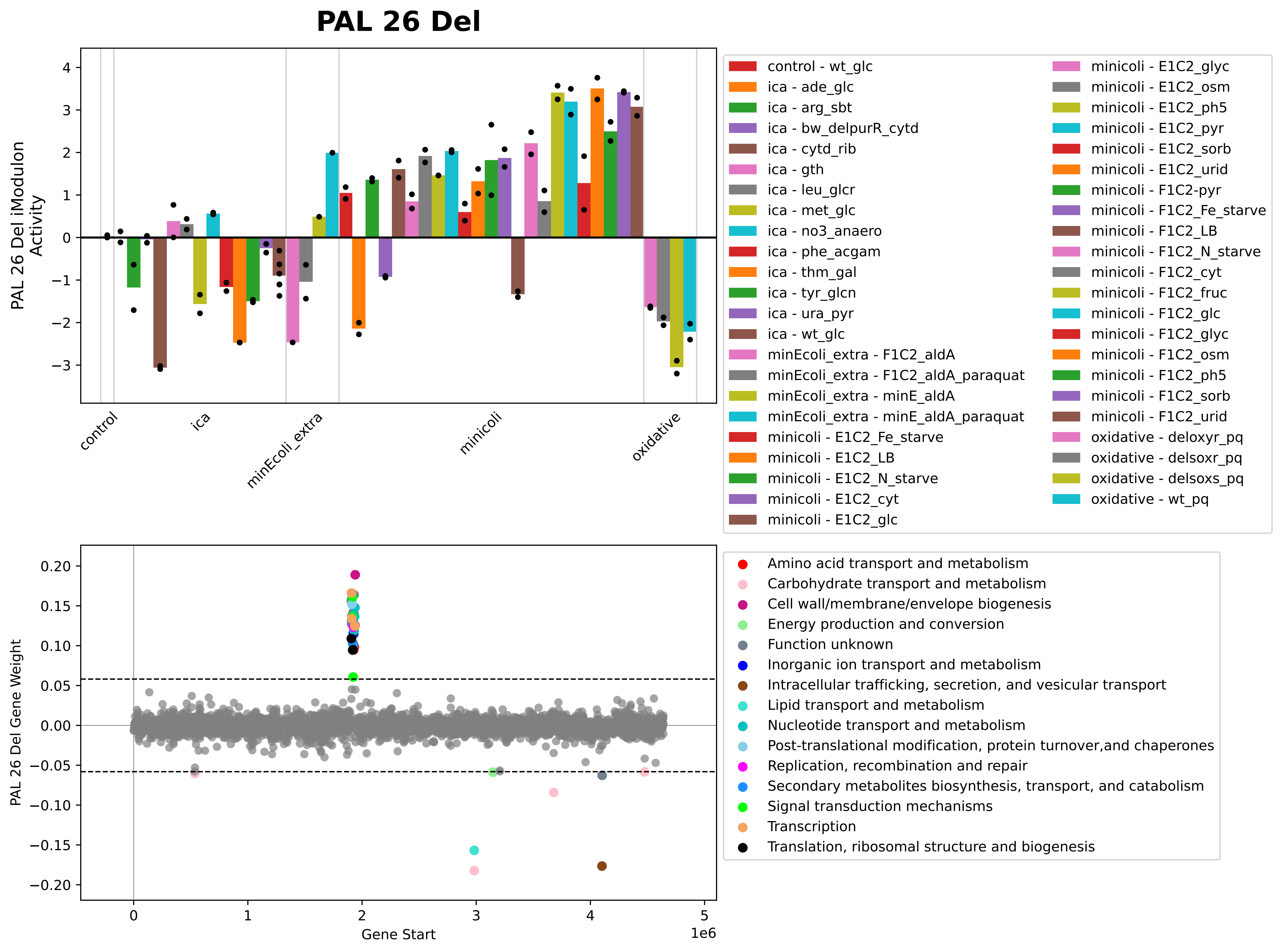

PAL 26 Del
PAL 26 Del iModulon Activity
control
ica
minEcoli_extra
minicoli
oxidative
control - wt_glc
ica - ade_glc
ica - arg_sbt
ica - bw_delpurR_cytd
ica - cytd_rib
ica - gth
ica - leu_glcr
ica - met_glc
ica - no3_anaero
ica - phe_acgam
ica - thm_gal
ica - tyr_glcn
ica - ura_pyr
ica - wt_glc
minEcoli_extra - F1C2_aldA
minEcoli_extra - F1C2_aldA_paraquat
minEcoli_extra - minE_aldA
minEcoli_extra - minE_aldA_paraquat
minicoli - E1C2_Fe_starve
minicoli - E1C2_LB
minicoli - E1C2_N_starve
minicoli - E1C2_cyt
minicoli - E1C2_glc
minicoli - E1C2_glyc
minicoli - E1C2_osm
minicoli - E1C2_ph5
minicoli - E1C2_pyr
minicoli - E1C2_sorb
minicoli - E1C2_urid
minicoli - F1C2-pyr
minicoli - F1C2_Fe_starve
minicoli - F1C2_LB
minicoli - F1C2_N_starve
minicoli - F1C2_cyt
minicoli - F1C2_fruc
minicoli - F1C2_glc
minicoli - F1C2_glyc
minicoli - F1C2_osm
minicoli - F1C2_ph5
minicoli - F1C2_sorb
minicoli - F1C2_urid
oxidative - deloxyr_pq
oxidative - delsoxr_pq
oxidative - delsoxs_pq
oxidative - wt_pq
PAL 26 Del Gene Weight
Gene Start
1e6
Amino acid transport and metabolism
Carbohydrate transport and metabolism
Cell wall/membrane/envelope biogenesis
Energy production and conversion
Function unknown
Inorganic ion transport and metabolism
Intracellular trafficking, secretion, and vesicular transport
Lipid transport and metabolism
Nucleotide transport and metabolism
Post-translational modification, protein turnover,and chaperones
Replication, recombination and repair
Secondary metabolites biosynthesis, transport, and catabolism
Signal transduction mechanisms
Transcription
Translation, ribosomal structure and biogenesis

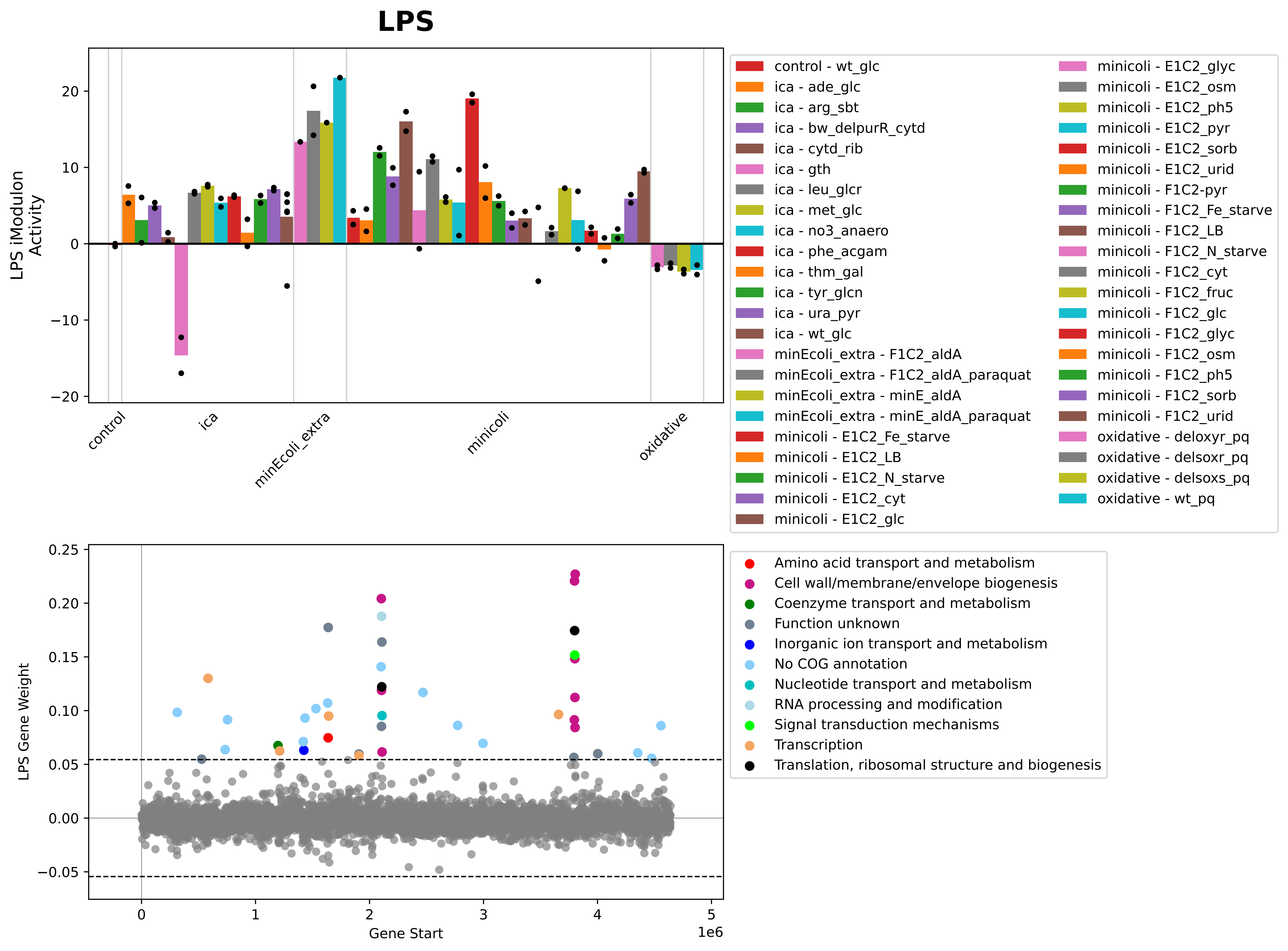

LPS
LPS iModulon Activity
20
10
0
−10
−20
control
ica
minEcoli_extra
minicoli
oxidative
control - wt_glc
ica - ade_glc
ica - arg_sbt
ica - bw_delpurR_cytd
ica - cytd_rib
ica - gth
ica - leu_glcr
ica - met_glc
ica - no3_anaero
ica - phe_acgam
ica - thm_gal
ica - tyr_glcn
ica - ura_pyr
ica - wt_glc
minEcoli_extra - F1C2_aldA
minEcoli_extra - F1C2_aldA_paraquat
minEcoli_extra - minE_aldA
minEcoli_extra - minE_aldA_paraquat
minicoli - E1C2_Fe_starve
minicoli - E1C2_LB
minicoli - E1C2_N_starve
minicoli - E1C2_cyt
minicoli - E1C2_glc
minicoli - E1C2_glyc
minicoli - E1C2_osm
minicoli - E1C2_ph5
minicoli - E1C2_pyr
minicoli - E1C2_sorb
minicoli - E1C2_urid
minicoli - F1C2-pyr
minicoli - F1C2_Fe_starve
minicoli - F1C2_LB
minicoli - F1C2_N_starve
minicoli - F1C2_cyt
minicoli - F1C2_fruc
minicoli - F1C2_glc
minicoli - F1C2_glyc
minicoli - F1C2_osm
minicoli - F1C2_ph5
minicoli - F1C2_sorb
minicoli - F1C2_urid
oxidative - deloxyr_pq
oxidative - delsoxr_pq
oxidative - delsoxs_pq
oxidative - wt_pq
LPS Gene Weight
0.25
0.20
0.15
0.10
0.05
0.00
−0.05
0
1
2
3
4
5
Gene Start
1e6
Amino acid transport and metabolism
Cell wall/membrane/envelope biogenesis
Coenzyme transport and metabolism
Function unknown
Inorganic ion transport and metabolism
No COG annotation
Nucleotide transport and metabolism
RNA processing and modification
Signal transduction mechanisms
Transcription
Translation, ribosomal structure and biogenesis

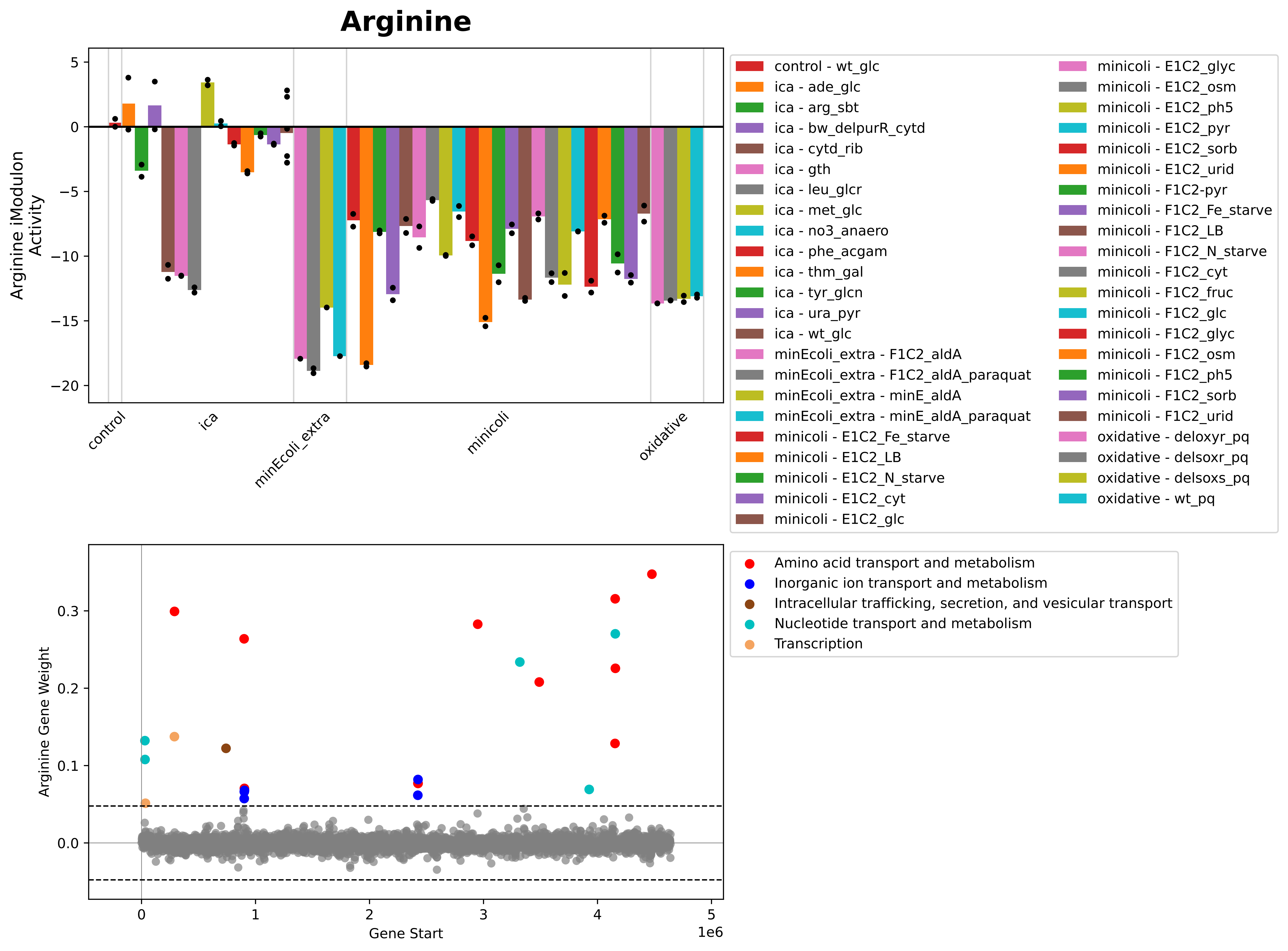
Arginine
Arginine iModulon Activity
control
ica
minEcoli_extra
minicoli
oxidative
control - wt_glc
ica - ade_glc
ica - arg_sbt
ica - bw_delpurR_cytd
ica - cytd_rib
ica - gth
ica - leu_glcr
ica - met_glc
ica - no3_anaero
ica - phe_acgam
ica - thm_gal
ica - tyr_glcn
ica - ura_pyr
ica - wt_glc
minEcoli_extra - F1C2_aldA
minEcoli_extra - F1C2_aldA_paraquat
minEcoli_extra - minE_aldA
minEcoli_extra - minE_aldA_paraquat
minicoli - E1C2_Fe_starve
minicoli - E1C2_LB
minicoli - E1C2_N_starve
minicoli - E1C2_cyt
minicoli - E1C2_glc
minicoli - E1C2_glyc
minicoli - E1C2_osm
minicoli - E1C2_ph5
minicoli - E1C2_pyr
minicoli - E1C2_sorb
minicoli - E1C2_urid
minicoli - F1C2-pyr
minicoli - F1C2_Fe_starve
minicoli - F1C2_LB
minicoli - F1C2_N_starve
minicoli - F1C2_cyt
minicoli - F1C2_fruc
minicoli - F1C2_glc
minicoli - F1C2_glyc
minicoli - F1C2_osm
minicoli - F1C2_ph5
minicoli - F1C2_sorb
minicoli - F1C2_urid
oxidative - deloxyr_pq
oxidative - delsoxr_pq
oxidative - delsoxs_pq
oxidative - wt_pq
Arginine Gene Weight
Gene Start
1e6
Amino acid transport and metabolism
Inorganic ion transport and metabolism
Intracellular trafficking, secretion, and vesicular transport
Nucleotide transport and metabolism
Transcription

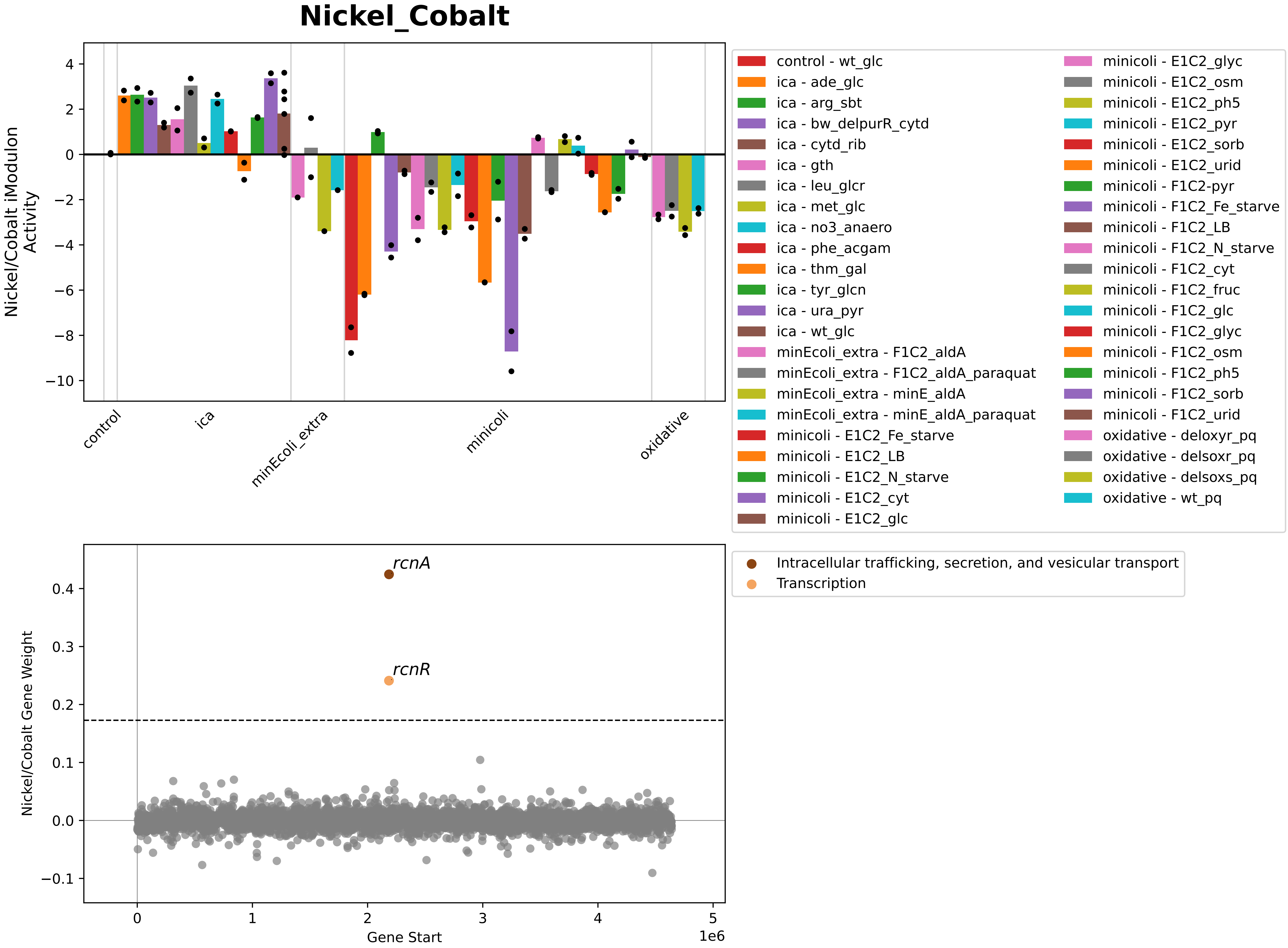
Nickel_Cobalt
Nickel/Cobalt iModulon Activity
control
ica
minEcoli_extra
minicoli
oxidative
control - wt_glc
ica - ade_glc
ica - arg_sbt
ica - bw_delpurR_cytd
ica - cytd_rib
ica - gth
ica - leu_glcr
ica - met_glc
ica - no3_anaero
ica - phe_acgam
ica - thm_gal
ica - tyr_glcn
ica - ura_pyr
ica - wt_glc
minEcoli_extra - F1C2_aldA
minEcoli_extra - F1C2_aldA_paraquat
minEcoli_extra - minE_aldA
minEcoli_extra - minE_aldA_paraquat
minicoli - E1C2_Fe_starve
minicoli - E1C2_LB
minicoli - E1C2_N_starve
minicoli - E1C2_cyt
minicoli - E1C2_glc
minicoli - E1C2_glyc
minicoli - E1C2_osm
minicoli - E1C2_ph5
minicoli - E1C2_pyr
minicoli - E1C2_sorb
minicoli - E1C2_urid
minicoli - F1C2-pyr
minicoli - F1C2_Fe_starve
minicoli - F1C2_LB
minicoli - F1C2_N_starve
minicoli - F1C2_cyt
minicoli - F1C2_fruc
minicoli - F1C2_glc
minicoli - F1C2_glyc
minicoli - F1C2_osm
minicoli - F1C2_ph5
minicoli - F1C2_sorb
minicoli - F1C2_urid
oxidative - deloxyr_pq
oxidative - delsoxr_pq
oxidative - delsoxs_pq
oxidative - wt_pq
rcnA
rcnR
Nickel/Cobalt Gene Weight
Gene Start
1e6
Intracellular trafficking, secretion, and vesicular transport
Transcription

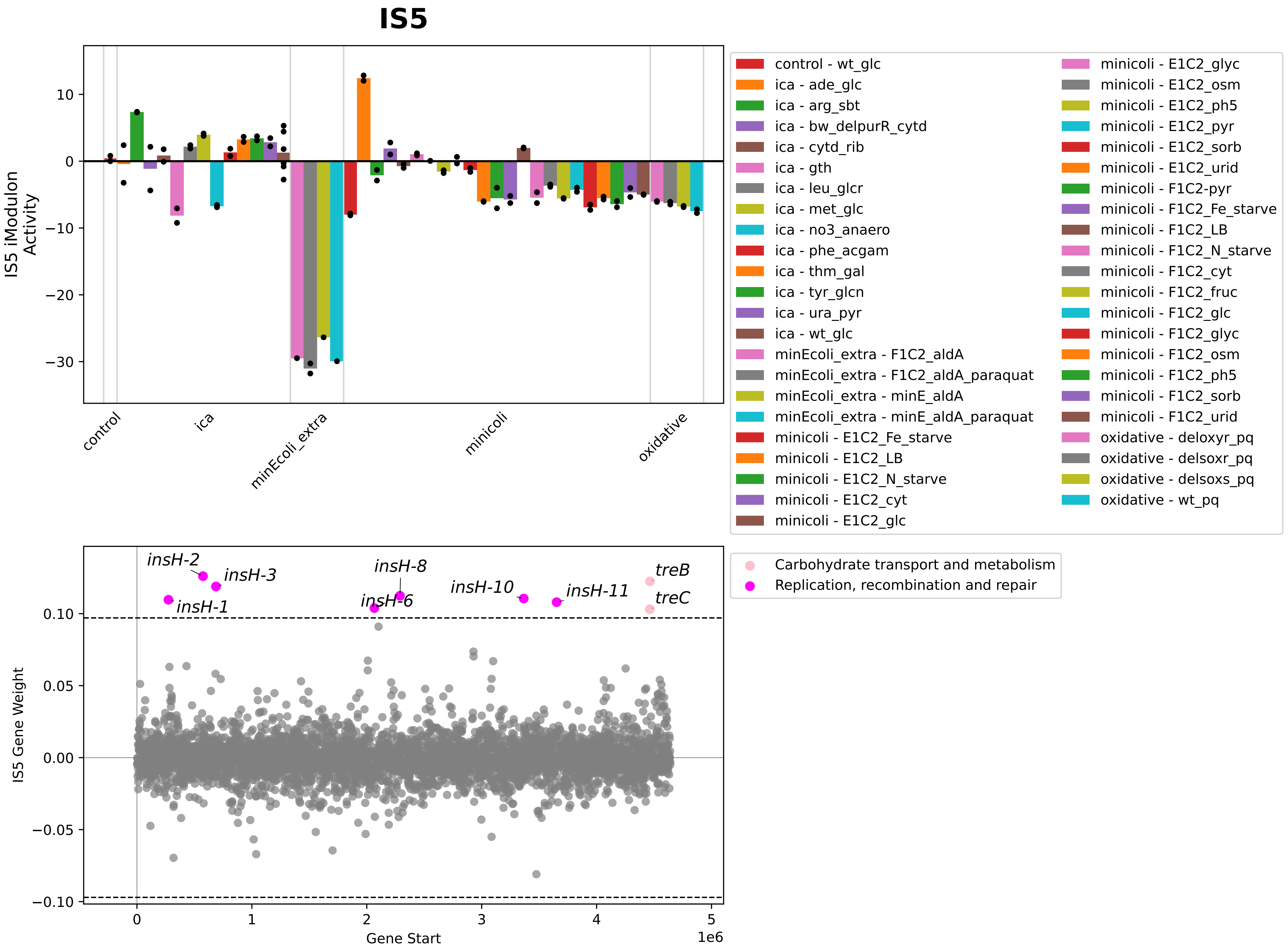
IS5
IS5 iModulon Activity
control
ica
minEcoli_extra
minicoli
oxidative
control - wt_glc
ica - ade_glc
ica - arg_sbt
ica - bw_delpurR_cytd
ica - cytd_rib
ica - gth
ica - leu_glcr
ica - met_glc
ica - no3_anaero
ica - phe_acgam
ica - thm_gal
ica - tyr_glcn
ica - ura_pyr
ica - wt_glc
minEcoli_extra - F1C2_aldA
minEcoli_extra - F1C2_aldA_paraquat
minEcoli_extra - minE_aldA
minEcoli_extra - minE_aldA_paraquat
minicoli - E1C2_Fe_starve
minicoli - E1C2_LB
minicoli - E1C2_N_starve
minicoli - E1C2_cyt
minicoli - E1C2_glc
minicoli - E1C2_glyc
minicoli - E1C2_osm
minicoli - E1C2_ph5
minicoli - E1C2_pyr
minicoli - E1C2_sorb
minicoli - E1C2_urid
minicoli - F1C2-pyr
minicoli - F1C2_Fe_starve
minicoli - F1C2_LB
minicoli - F1C2_N_starve
minicoli - F1C2_cyt
minicoli - F1C2_fruc
minicoli - F1C2_glc
minicoli - F1C2_glyc
minicoli - F1C2_osm
minicoli - F1C2_ph5
minicoli - F1C2_sorb
minicoli - F1C2_urid
oxidative - deloxyr_pq
oxidative - delsoxr_pq
oxidative - delsoxs_pq
oxidative - wt_pq
insH-2
insH-3
insH-1
insH-8
insH-6
insH-10
insH-11
treB
treC
Carbohydrate transport and metabolism
Replication, recombination and repair
IS5 Gene Weight
Gene Start
1e6

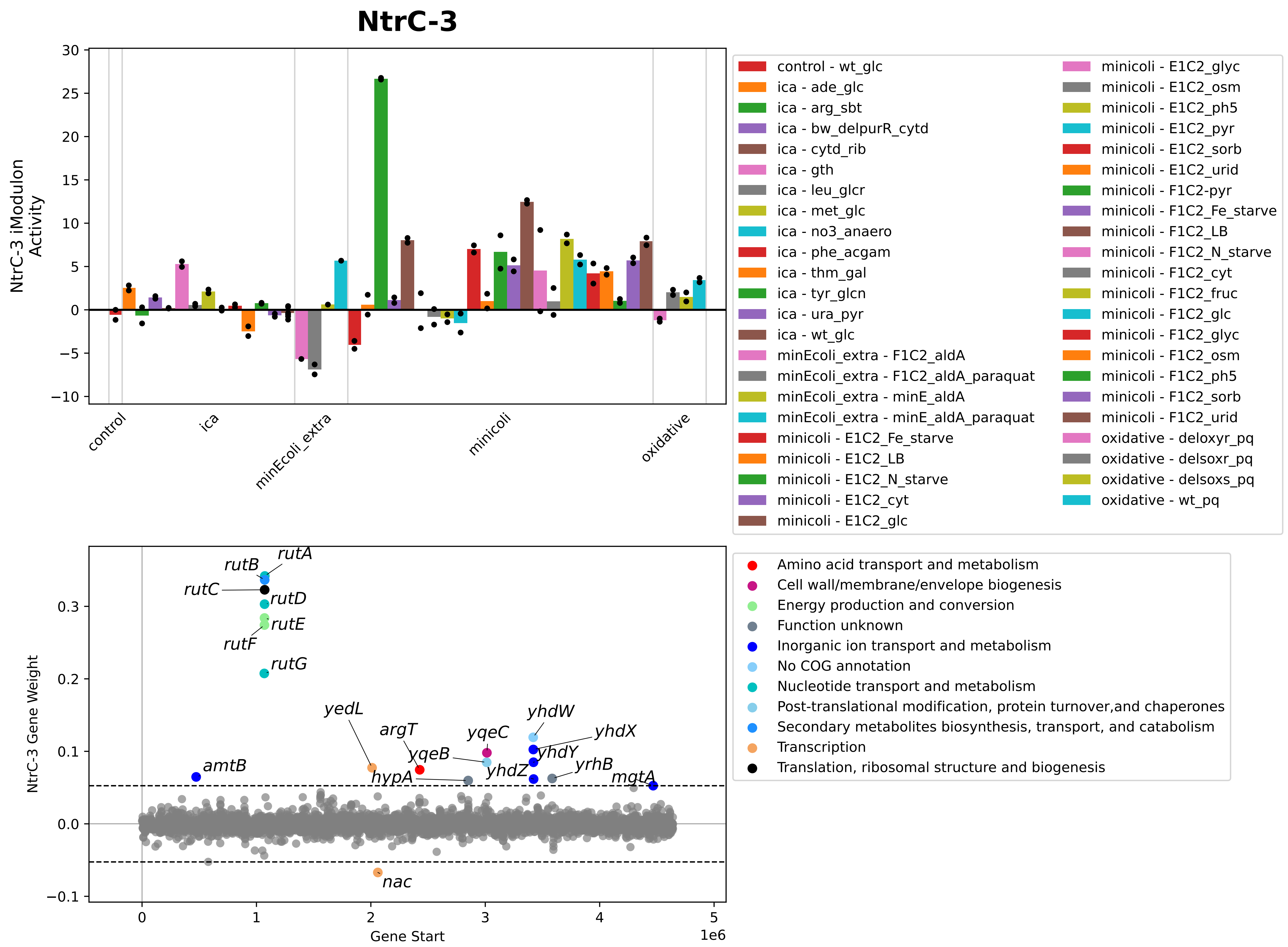
NtrC-3
NtrC-3 iModulon Activity
control
ica
minEcoli_extra
minicoli
oxidative
control - wt_glc
ica - ade_glc
ica - arg_sbt
ica - bw_delpurR_cytd
ica - cytd_rib
ica - gth
ica - leu_glcr
ica - met_glc
ica - no3_anaero
ica - phe_acgam
ica - thm_gal
ica - tyr_glcn
ica - ura_pyr
ica - wt_glc
minEcoli_extra - F1C2_aldA
minEcoli_extra - F1C2_aldA_paraquat
minEcoli_extra - minE_aldA
minEcoli_extra - minE_aldA_paraquat
minicoli - E1C2_Fe_starve
minicoli - E1C2_LB
minicoli - E1C2_N_starve
minicoli - E1C2_cyt
minicoli - E1C2_glc
minicoli - E1C2_glyc
minicoli - E1C2_osm
minicoli - E1C2_ph5
minicoli - E1C2_pyr
minicoli - E1C2_sorb
minicoli - E1C2_urid
minicoli - F1C2-pyr
minicoli - F1C2_Fe_starve
minicoli - F1C2_LB
minicoli - F1C2_N_starve
minicoli - F1C2_cyt
minicoli - F1C2_fruc
minicoli - F1C2_glc
minicoli - F1C2_glyc
minicoli - F1C2_osm
minicoli - F1C2_ph5
minicoli - F1C2_sorb
minicoli - F1C2_urid
oxidative - deloxyr_pq
oxidative - delsoxr_pq
oxidative - delsoxs_pq
oxidative - wt_pq
NtrC-3 Gene Weight
Gene Start
1e6
rutA
rutB
rutC
rutD
rutE
rutF
rutG
yedL
argT
yqeC
yhdW
yhdX
yqeB
yhdY
yhdZ
yrhB
amtB
hypA
mgtA
nac
Amino acid transport and metabolism
Cell wall/membrane/envelope biogenesis
Energy production and conversion
Function unknown
Inorganic ion transport and metabolism
No COG annotation
Nucleotide transport and metabolism
Post-translational modification, protein turnover,and chaperones
Secondary metabolites biosynthesis, transport, and catabolism
Transcription
Translation, ribosomal structure and biogenesis

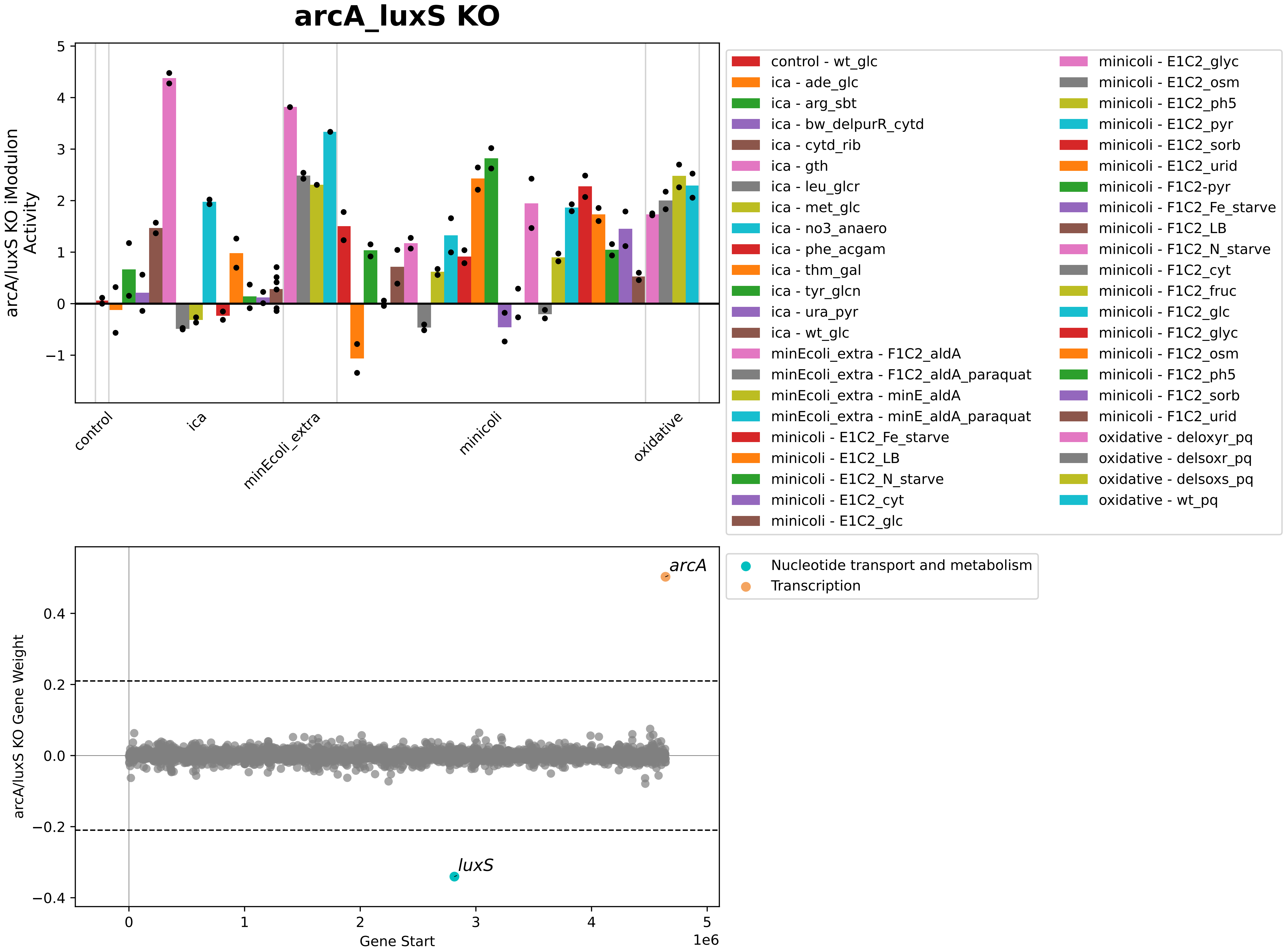
arcA_luxS KO
arcA/luxS KO iModulon Activity
control
ica
minEcoli_extra
minicoli
oxidative
control - wt_glc
ica - ade_glc
ica - arg_sbt
ica - bw_delpurR_cytd
ica - cytd_rib
ica - gth
ica - leu_glcr
ica - met_glc
ica - no3_anaero
ica - phe_acgam
ica - thm_gal
ica - tyr_glcn
ica - ura_pyr
ica - wt_glc
minEcoli_extra - F1C2_aldA
minEcoli_extra - F1C2_aldA_paraquat
minEcoli_extra - minE_aldA
minEcoli_extra - minE_aldA_paraquat
minicoli - E1C2_Fe_starve
minicoli - E1C2_LB
minicoli - E1C2_N_starve
minicoli - E1C2_cyt
minicoli - E1C2_glc
minicoli - E1C2_glyc
minicoli - E1C2_osm
minicoli - E1C2_ph5
minicoli - E1C2_pyr
minicoli - E1C2_sorb
minicoli - E1C2_urid
minicoli - F1C2-pyr
minicoli - F1C2_Fe_starve
minicoli - F1C2_LB
minicoli - F1C2_N_starve
minicoli - F1C2_cyt
minicoli - F1C2_fruc
minicoli - F1C2_glc
minicoli - F1C2_glyc
minicoli - F1C2_osm
minicoli - F1C2_ph5
minicoli - F1C2_sorb
minicoli - F1C2_urid
oxidative - deloxyr_pq
oxidative - delsoxr_pq
oxidative - delsoxs_pq
oxidative - wt_pq
arcA
luxS
Nucleotide transport and metabolism
Transcription
arcA/luxS KO Gene Weight
Gene Start
1e6

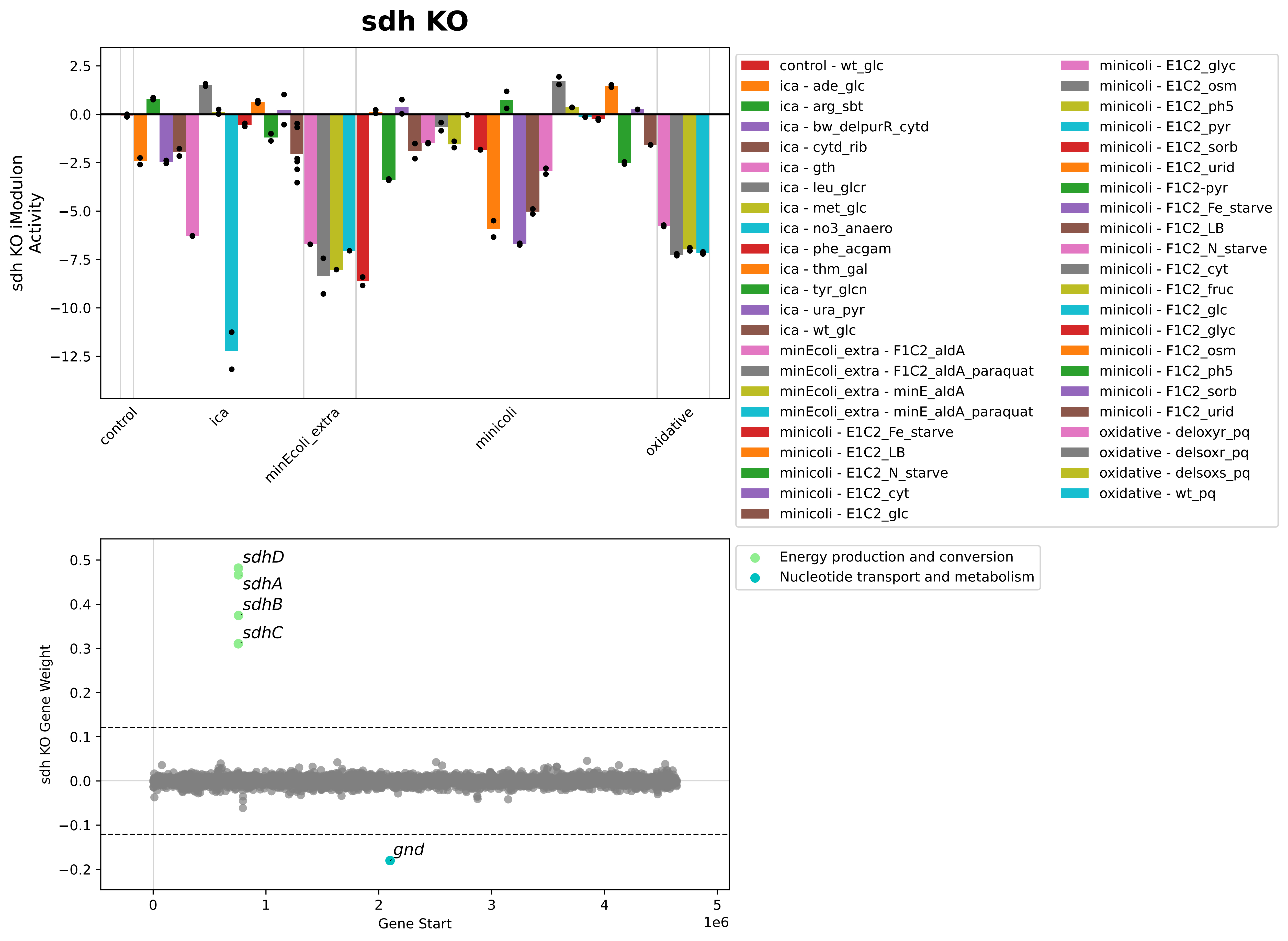

sdh KO
sdh KO iModulon Activity
control
ica
minEcoli_extra
minicoli
oxidative
control - wt_glc
ica - ade_glc
ica - arg_sbt
ica - bw_delpurR_cytd
ica - cytd_rib
ica - gth
ica - leu_glcr
ica - met_glc
ica - no3_anaero
ica - phe_acgam
ica - thm_gal
ica - tyr_glcn
ica - ura_pyr
ica - wt_glc
minEcoli_extra - F1C2_aldA
minEcoli_extra - F1C2_aldA_paraquat
minEcoli_extra - minE_aldA
minEcoli_extra - minE_aldA_paraquat
minicoli - E1C2_Fe_starve
minicoli - E1C2_LB
minicoli - E1C2_N_starve
minicoli - E1C2_cyt
minicoli - E1C2_glc
minicoli - E1C2_glyc
minicoli - E1C2_osm
minicoli - E1C2_ph5
minicoli - E1C2_pyr
minicoli - E1C2_sorb
minicoli - E1C2_urid
minicoli - F1C2-pyr
minicoli - F1C2_Fe_starve
minicoli - F1C2_LB
minicoli - F1C2_N_starve
minicoli - F1C2_cyt
minicoli - F1C2_fruc
minicoli - F1C2_glc
minicoli - F1C2_glyc
minicoli - F1C2_osm
minicoli - F1C2_ph5
minicoli - F1C2_sorb
minicoli - F1C2_urid
oxidative - deloxyr_pq
oxidative - delsoxr_pq
oxidative - delsoxs_pq
oxidative - wt_pq
sdh KO Gene Weight
sdhD
sdhA
sdhB
sdhC
gnd
Gene Start
1e6
Energy production and conversion
Nucleotide transport and metabolism

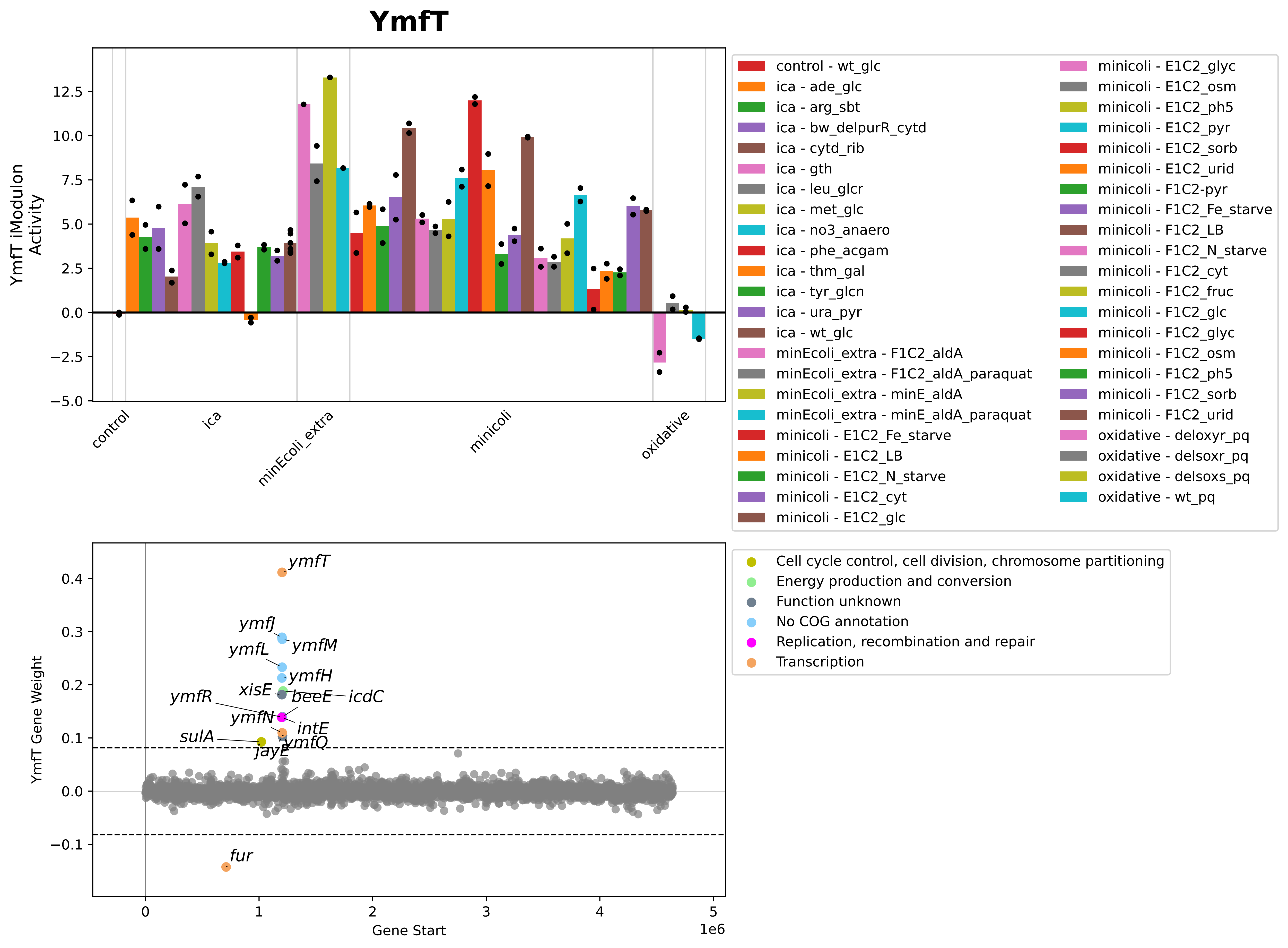
YmfT
YmfT iModulon Activity
control
ica
minEcoli_extra
minicoli
oxidative
control - wt_glc
ica - ade_glc
ica - arg_sbt
ica - bw_delpurR_cytd
ica - cytd_rib
ica - gth
ica - leu_glcr
ica - met_glc
ica - no3_anaero
ica - phe_acgam
ica - thm_gal
ica - tyr_glcn
ica - ura_pyr
ica - wt_glc
minEcoli_extra - F1C2_aldA
minEcoli_extra - F1C2_aldA_paraquat
minEcoli_extra - minE_aldA
minEcoli_extra - minE_aldA_paraquat
minicoli - E1C2_Fe_starve
minicoli - E1C2_LB
minicoli - E1C2_N_starve
minicoli - E1C2_cyt
minicoli - E1C2_glc
minicoli - E1C2_glyc
minicoli - E1C2_osm
minicoli - E1C2_ph5
minicoli - E1C2_pyr
minicoli - E1C2_sorb
minicoli - E1C2_urid
minicoli - F1C2-pyr
minicoli - F1C2_Fe_starve
minicoli - F1C2_LB
minicoli - F1C2_N_starve
minicoli - F1C2_cyt
minicoli - F1C2_fruc
minicoli - F1C2_glc
minicoli - F1C2_glyc
minicoli - F1C2_osm
minicoli - F1C2_ph5
minicoli - F1C2_sorb
minicoli - F1C2_urid
oxidative - deloxyr_pq
oxidative - delsoxr_pq
oxidative - delsoxs_pq
oxidative - wt_pq
YmfT Gene Weight
Gene Start
1e6
ymfT
ymfJ
ymfM
ymfL
ymfH
xisE
beeE
icdC
ymfR
ymfN
intE
sulA
ymfQ
jayE
fur
Cell cycle control, cell division, chromosome partitioning
Energy production and conversion
Function unknown
No COG annotation
Replication, recombination and repair
Transcription

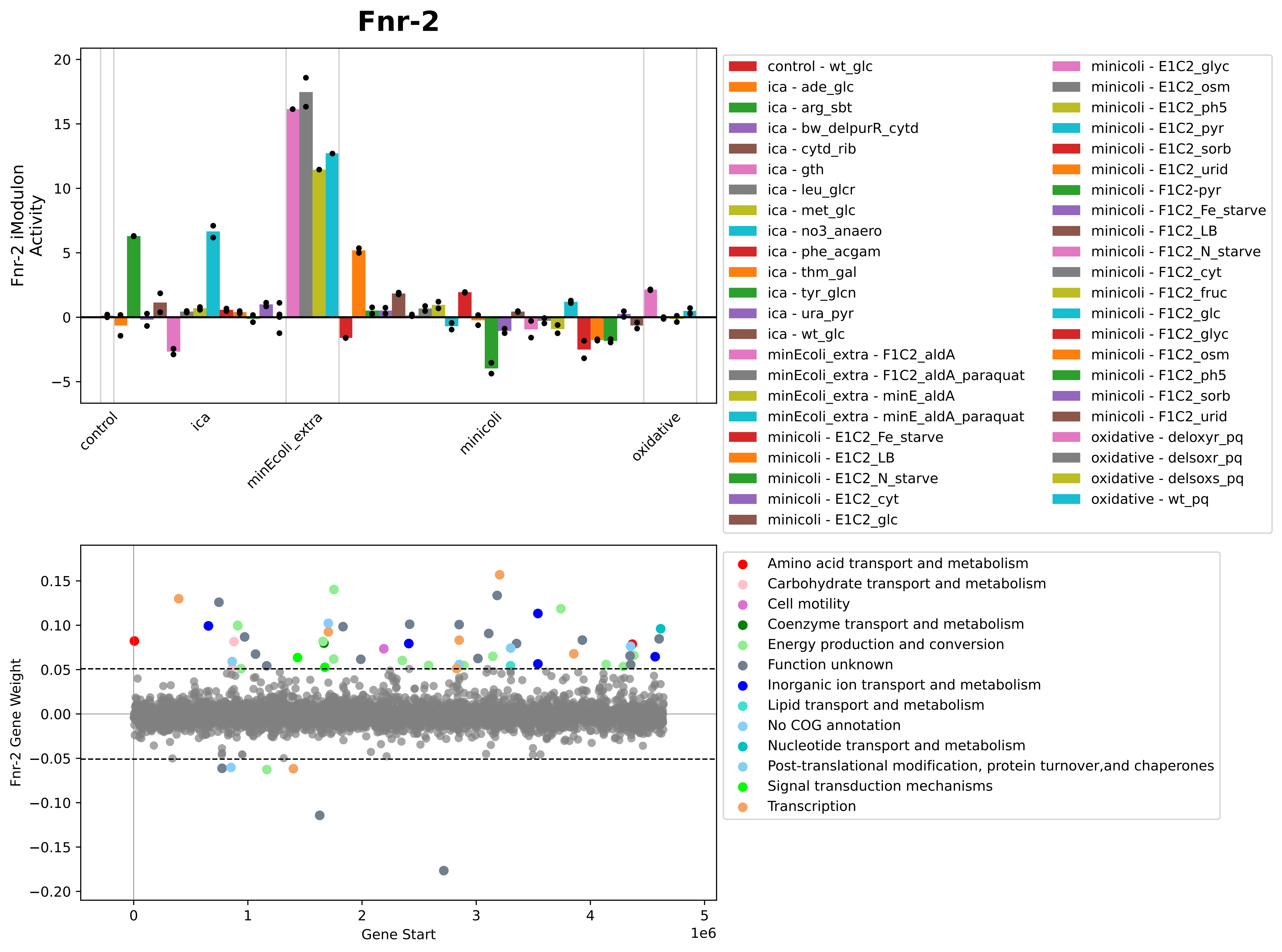
Fnr-2
Fnr-2 iModulon Activity
control
ica
minEcoli_extra
minicoli
oxidative
control - wt_glc
ica - ade_glc
ica - arg_sbt
ica - bw_delpurR_cytd
ica - cytd_rib
ica - gth
ica - leu_glcr
ica - met_glc
ica - no3_anaero
ica - phe_acgam
ica - thm_gal
ica - tyr_glcn
ica - ura_pyr
ica - wt_glc
minEcoli_extra - F1C2_aldA
minEcoli_extra - F1C2_aldA_paraquat
minEcoli_extra - minE_aldA
minEcoli_extra - minE_aldA_paraquat
minicoli - E1C2_Fe_starve
minicoli - E1C2_LB
minicoli - E1C2_N_starve
minicoli - E1C2_cyt
minicoli - E1C2_glc
minicoli - E1C2_glyc
minicoli - E1C2_osm
minicoli - E1C2_ph5
minicoli - E1C2_pyr
minicoli - E1C2_sorb
minicoli - E1C2_urid
minicoli - F1C2-pyr
minicoli - F1C2_Fe_starve
minicoli - F1C2_LB
minicoli - F1C2_N_starve
minicoli - F1C2_cyt
minicoli - F1C2_fruc
minicoli - F1C2_glc
minicoli - F1C2_glyc
minicoli - F1C2_osm
minicoli - F1C2_ph5
minicoli - F1C2_sorb
minicoli - F1C2_urid
oxidative - deloxyr_pq
oxidative - delsoxr_pq
oxidative - delsoxs_pq
oxidative - wt_pq
Fnr-2 Gene Weight
Gene Start
1e6
Amino acid transport and metabolism
Carbohydrate transport and metabolism
Cell motility
Coenzyme transport and metabolism
Energy production and conversion
Function unknown
Inorganic ion transport and metabolism
Lipid transport and metabolism
No COG annotation
Nucleotide transport and metabolism
Post-translational modification, protein turnover,and chaperones
Signal transduction mechanisms
Transcription

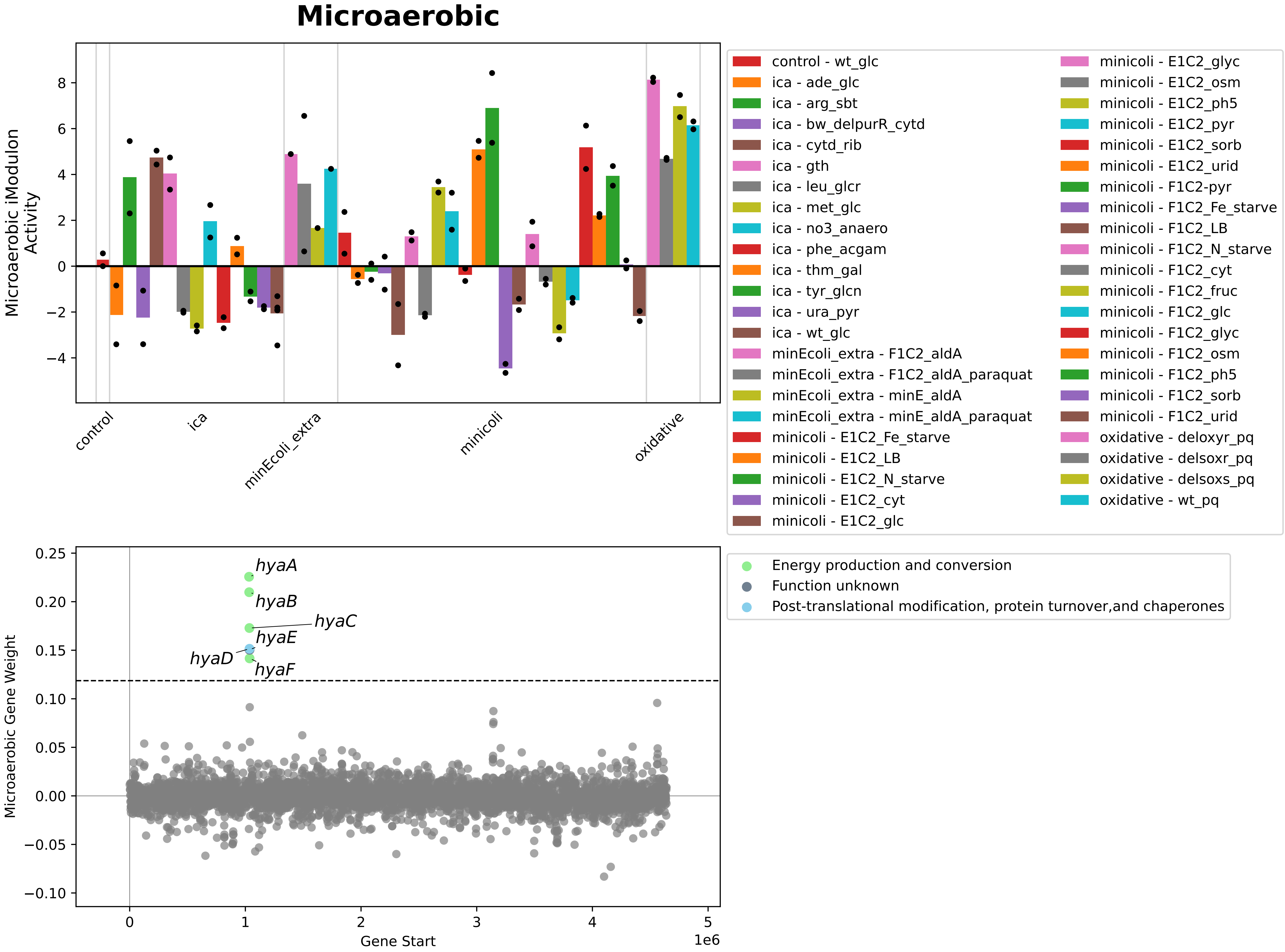
Microaerobic
Microaerobic iModulon Activity
control
ica
minEcoli_extra
minicoli
oxidative
control - wt_glc
ica - ade_glc
ica - arg_sbt
ica - bw_delpurR_cytd
ica - cytd_rib
ica - gth
ica - leu_glcr
ica - met_glc
ica - no3_anaero
ica - phe_acgam
ica - thm_gal
ica - tyr_glcn
ica - ura_pyr
ica - wt_glc
minEcoli_extra - F1C2_aldA
minEcoli_extra - F1C2_aldA_paraquat
minEcoli_extra - minE_aldA
minEcoli_extra - minE_aldA_paraquat
minicoli - E1C2_Fe_starve
minicoli - E1C2_LB
minicoli - E1C2_N_starve
minicoli - E1C2_cyt
minicoli - E1C2_glc
minicoli - E1C2_glyc
minicoli - E1C2_osm
minicoli - E1C2_ph5
minicoli - E1C2_pyr
minicoli - E1C2_sorb
minicoli - E1C2_urid
minicoli - F1C2-pyr
minicoli - F1C2_Fe_starve
minicoli - F1C2_LB
minicoli - F1C2_N_starve
minicoli - F1C2_cyt
minicoli - F1C2_fruc
minicoli - F1C2_glc
minicoli - F1C2_glyc
minicoli - F1C2_osm
minicoli - F1C2_ph5
minicoli - F1C2_sorb
minicoli - F1C2_urid
oxidative - deloxyr_pq
oxidative - delsoxr_pq
oxidative - delsoxs_pq
oxidative - wt_pq
hyaA
hyaB
hyaC
hyaE
hyaD
hyaF
Microaerobic Gene Weight
Gene Start
1e6
Energy production and conversion
Function unknown
Post-translational modification, protein turnover,and chaperones

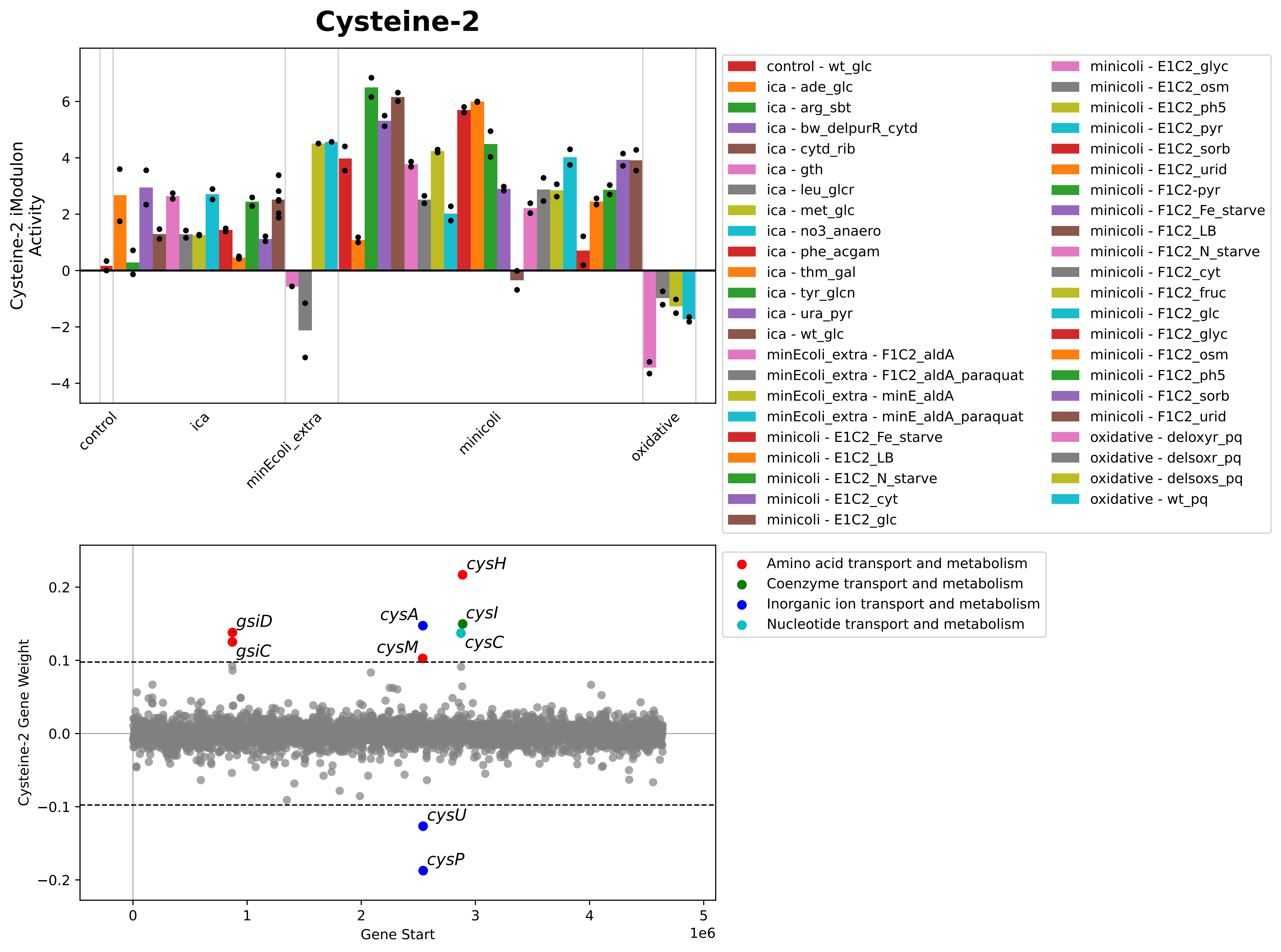

Cysteine-2
Cysteine-2 iModulon Activity
control
ica
minEcoli_extra
minicoli
oxidative
control - wt_glc
ica - ade_glc
ica - arg_sbt
ica - bw_delpurR_cytd
ica - cytd_rib
ica - gth
ica - leu_glcr
ica - met_glc
ica - no3_anaero
ica - phe_acgam
ica - thm_gal
ica - tyr_glcn
ica - ura_pyr
ica - wt_glc
minEcoli_extra - F1C2_aldA
minEcoli_extra - F1C2_aldA_paraquat
minEcoli_extra - minE_aldA
minEcoli_extra - minE_aldA_paraquat
minicoli - E1C2_Fe_starve
minicoli - E1C2_LB
minicoli - E1C2_N_starve
minicoli - E1C2_cyt
minicoli - E1C2_glc
minicoli - E1C2_glyc
minicoli - E1C2_osm
minicoli - E1C2_ph5
minicoli - E1C2_pyr
minicoli - E1C2_sorb
minicoli - E1C2_urid
minicoli - F1C2-pyr
minicoli - F1C2_Fe_starve
minicoli - F1C2_LB
minicoli - F1C2_N_starve
minicoli - F1C2_cyt
minicoli - F1C2_fruc
minicoli - F1C2_glc
minicoli - F1C2_glyc
minicoli - F1C2_osm
minicoli - F1C2_ph5
minicoli - F1C2_sorb
minicoli - F1C2_urid
oxidative - deloxyr_pq
oxidative - delsoxr_pq
oxidative - delsoxs_pq
oxidative - wt_pq
Cysteine-2 Gene Weight
Gene Start
1e6
cysH
gsiD
gsiC
cysA
cysM
cysI
cysC
cysU
cysP
Amino acid transport and metabolism
Coenzyme transport and metabolism
Inorganic ion transport and metabolism
Nucleotide transport and metabolism

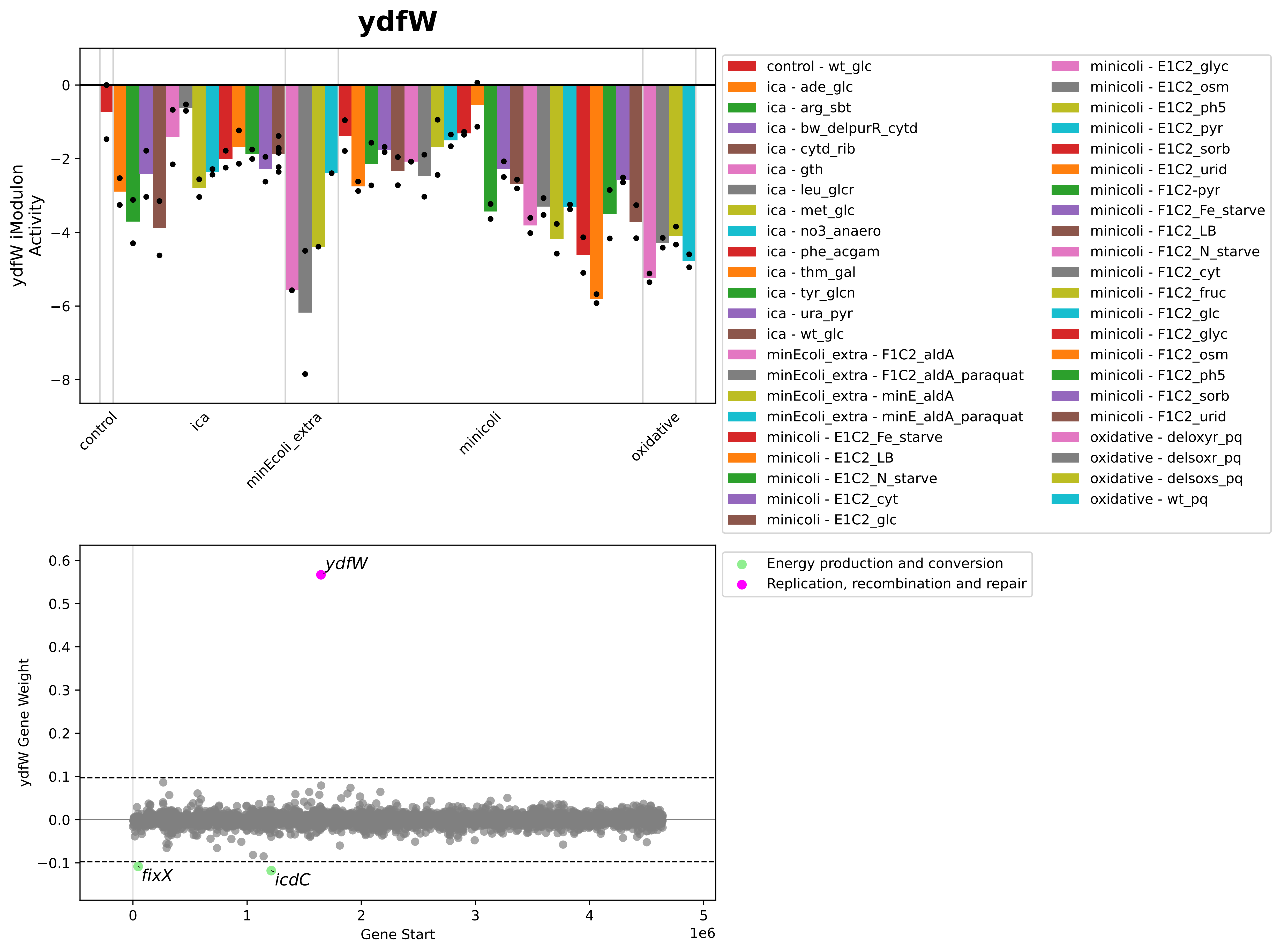

ydfW
ydfW iModulon Activity
0
−2
−4
−6
−8
control
ica
minEcoli_extra
minicoli
oxidative
control - wt_glc
ica - ade_glc
ica - arg_sbt
ica - bw_delpurR_cytd
ica - cytd_rib
ica - gth
ica - leu_glcr
ica - met_glc
ica - no3_anaero
ica - phe_acgam
ica - thm_gal
ica - tyr_glcn
ica - ura_pyr
ica - wt_glc
minEcoli_extra - F1C2_aldA
minEcoli_extra - F1C2_aldA_paraquat
minEcoli_extra - minE_aldA
minEcoli_extra - minE_aldA_paraquat
minicoli - E1C2_Fe_starve
minicoli - E1C2_LB
minicoli - E1C2_N_starve
minicoli - E1C2_cyt
minicoli - E1C2_glc
minicoli - E1C2_glyc
minicoli - E1C2_osm
minicoli - E1C2_ph5
minicoli - E1C2_pyr
minicoli - E1C2_sorb
minicoli - E1C2_urid
minicoli - F1C2-pyr
minicoli - F1C2_Fe_starve
minicoli - F1C2_LB
minicoli - F1C2_N_starve
minicoli - F1C2_cyt
minicoli - F1C2_fruc
minicoli - F1C2_glc
minicoli - F1C2_glyc
minicoli - F1C2_osm
minicoli - F1C2_ph5
minicoli - F1C2_sorb
minicoli - F1C2_urid
oxidative - deloxyr_pq
oxidative - delsoxr_pq
oxidative - delsoxs_pq
oxidative - wt_pq
ydfW Gene Weight
0.6
0.5
0.4
0.3
0.2
0.1
0.0
−0.1
ydfW
fixX
icdC
0
1
2
3
4
5
Gene Start
1e6
Energy production and conversion
Replication, recombination and repair

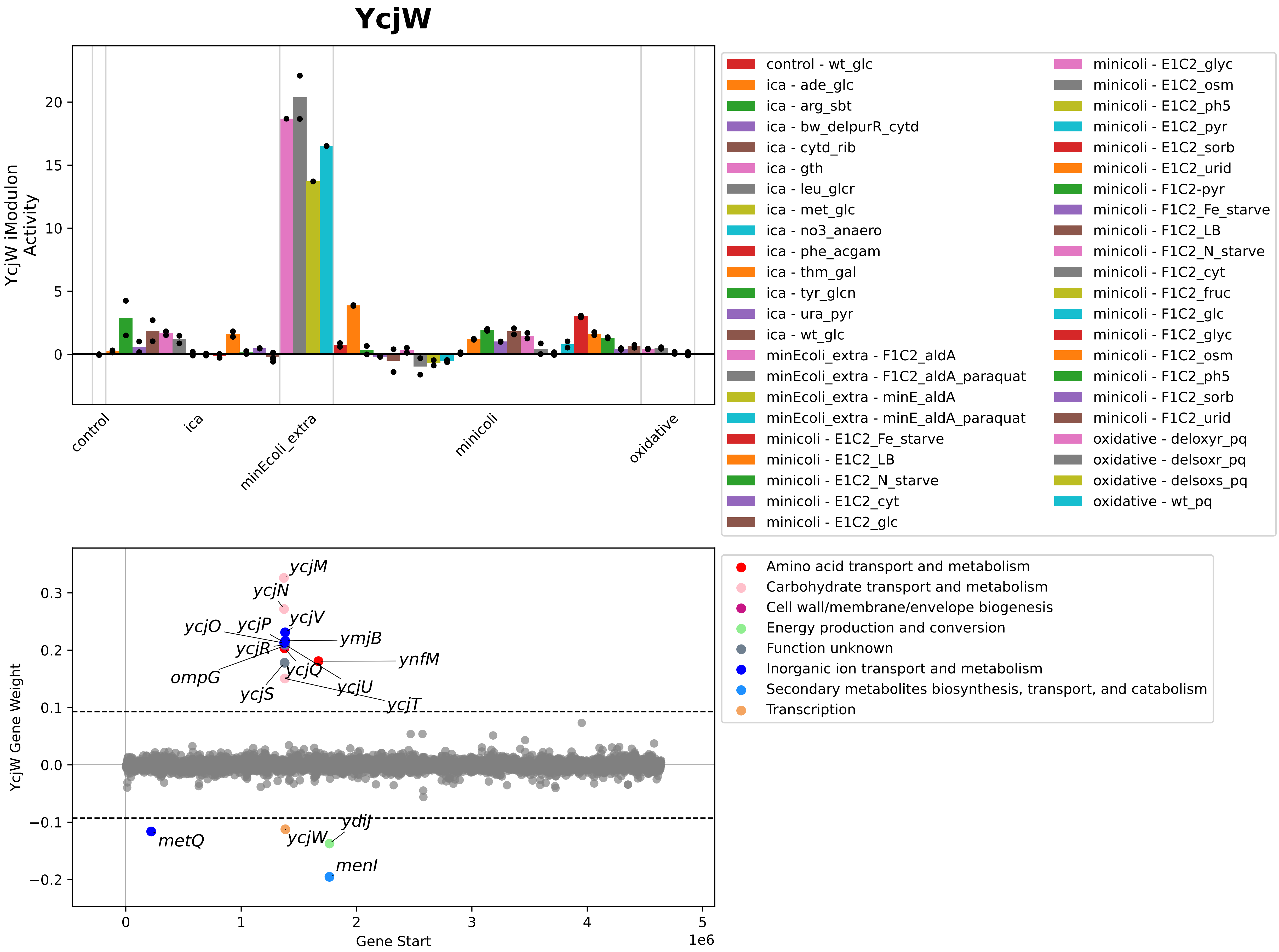

YcjW
YcjW iModulon Activity
control
ica
minEcoli_extra
minicoli
oxidative
control - wt_glc
ica - ade_glc
ica - arg_sbt
ica - bw_delpurR_cytd
ica - cytd_rib
ica - gth
ica - leu_glcr
ica - met_glc
ica - no3_anaero
ica - phe_acgam
ica - thm_gal
ica - tyr_glcn
ica - ura_pyr
ica - wt_glc
minEcoli_extra - F1C2_aldA
minEcoli_extra - F1C2_aldA_paraquat
minEcoli_extra - minE_aldA
minEcoli_extra - minE_aldA_paraquat
minicoli - E1C2_Fe_starve
minicoli - E1C2_LB
minicoli - E1C2_N_starve
minicoli - E1C2_cyt
minicoli - E1C2_glc
minicoli - E1C2_glyc
minicoli - E1C2_osm
minicoli - E1C2_ph5
minicoli - E1C2_pyr
minicoli - E1C2_sorb
minicoli - E1C2_urid
minicoli - F1C2-pyr
minicoli - F1C2_Fe_starve
minicoli - F1C2_LB
minicoli - F1C2_N_starve
minicoli - F1C2_cyt
minicoli - F1C2_fruc
minicoli - F1C2_glc
minicoli - F1C2_glyc
minicoli - F1C2_osm
minicoli - F1C2_ph5
minicoli - F1C2_sorb
minicoli - F1C2_urid
oxidative - deloxyr_pq
oxidative - delsoxr_pq
oxidative - delsoxs_pq
oxidative - wt_pq
YcjW Gene Weight
Gene Start
1e6
ycjM
ycjN
ycjV
ycjO
ycjP
ymjB
ycjR
ynfM
ompG
ycjQ
ycjU
ycjS
ycjT
metQ
ydiJ
ycjW
menI
Amino acid transport and metabolism
Carbohydrate transport and metabolism
Cell wall/membrane/envelope biogenesis
Energy production and conversion
Function unknown
Inorganic ion transport and metabolism
Secondary metabolites biosynthesis, transport, and catabolism
Transcription

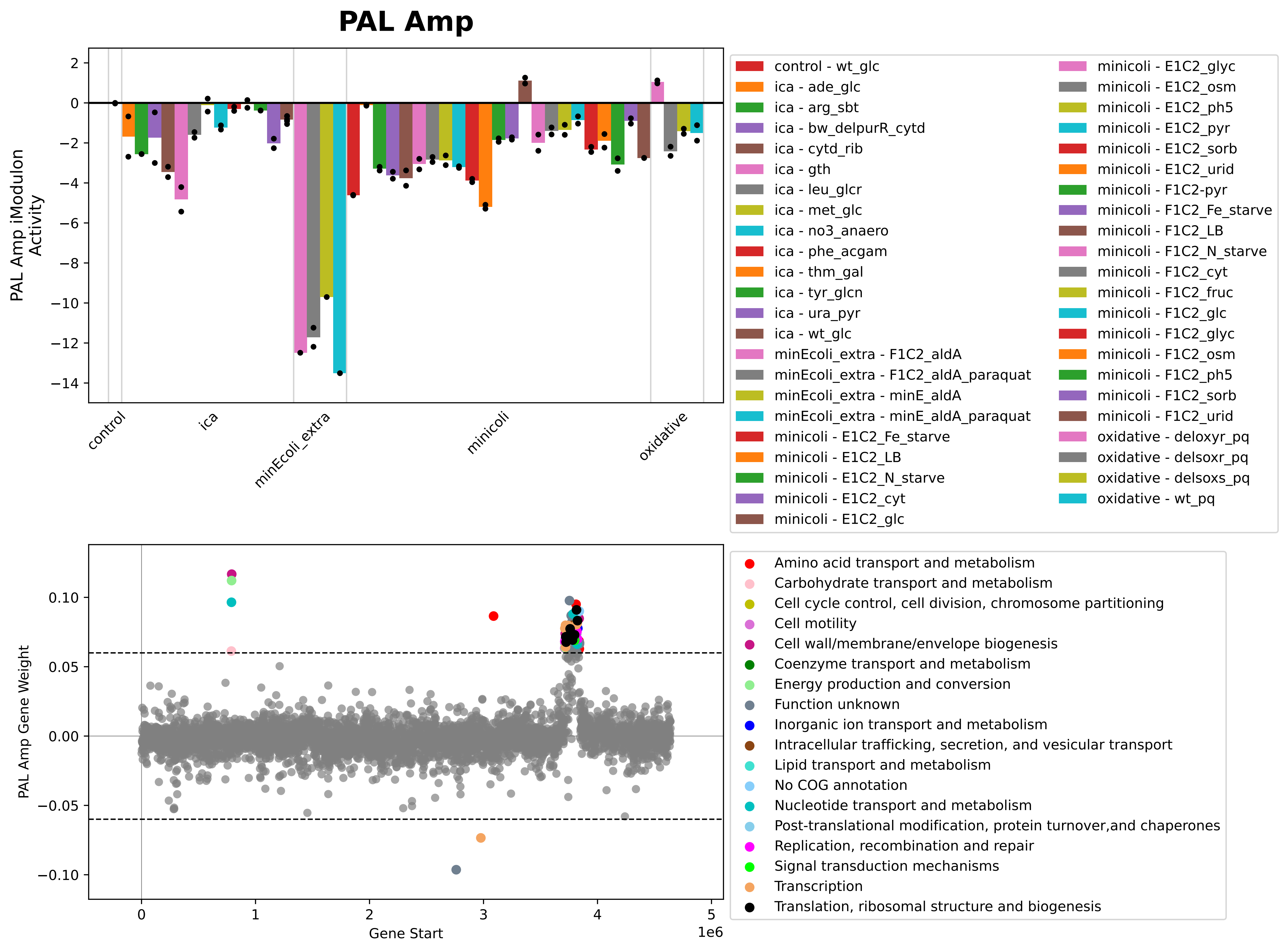
PAL Amp
PAL Amp iModulon Activity
control
ica
minEcoli_extra
minicoli
oxidative
control - wt_glc
ica - ade_glc
ica - arg_sbt
ica - bw_delpurR_cytd
ica - cytd_rib
ica - gth
ica - leu_glcr
ica - met_glc
ica - no3_anaero
ica - phe_acgam
ica - thm_gal
ica - tyr_glcn
ica - ura_pyr
ica - wt_glc
minEcoli_extra - F1C2_aldA
minEcoli_extra - F1C2_aldA_paraquat
minEcoli_extra - minE_aldA
minEcoli_extra - minE_aldA_paraquat
minicoli - E1C2_Fe_starve
minicoli - E1C2_LB
minicoli - E1C2_N_starve
minicoli - E1C2_cyt
minicoli - E1C2_glc
minicoli - E1C2_glyc
minicoli - E1C2_osm
minicoli - E1C2_ph5
minicoli - E1C2_pyr
minicoli - E1C2_sorb
minicoli - E1C2_urid
minicoli - F1C2-pyr
minicoli - F1C2_Fe_starve
minicoli - F1C2_LB
minicoli - F1C2_N_starve
minicoli - F1C2_cyt
minicoli - F1C2_fruc
minicoli - F1C2_glc
minicoli - F1C2_glyc
minicoli - F1C2_osm
minicoli - F1C2_ph5
minicoli - F1C2_sorb
minicoli - F1C2_urid
oxidative - deloxyr_pq
oxidative - delsoxr_pq
oxidative - delsoxs_pq
oxidative - wt_pq
PAL Amp Gene Weight
Gene Start
1e6
Amino acid transport and metabolism
Carbohydrate transport and metabolism
Cell cycle control, cell division, chromosome partitioning
Cell motility
Cell wall/membrane/envelope biogenesis
Coenzyme transport and metabolism
Energy production and conversion
Function unknown
Inorganic ion transport and metabolism
Intracellular trafficking, secretion, and vesicular transport
Lipid transport and metabolism
No COG annotation
Nucleotide transport and metabolism
Post-translational modification, protein turnover,and chaperones
Replication, recombination and repair
Signal transduction mechanisms
Transcription
Translation, ribosomal structure and biogenesis

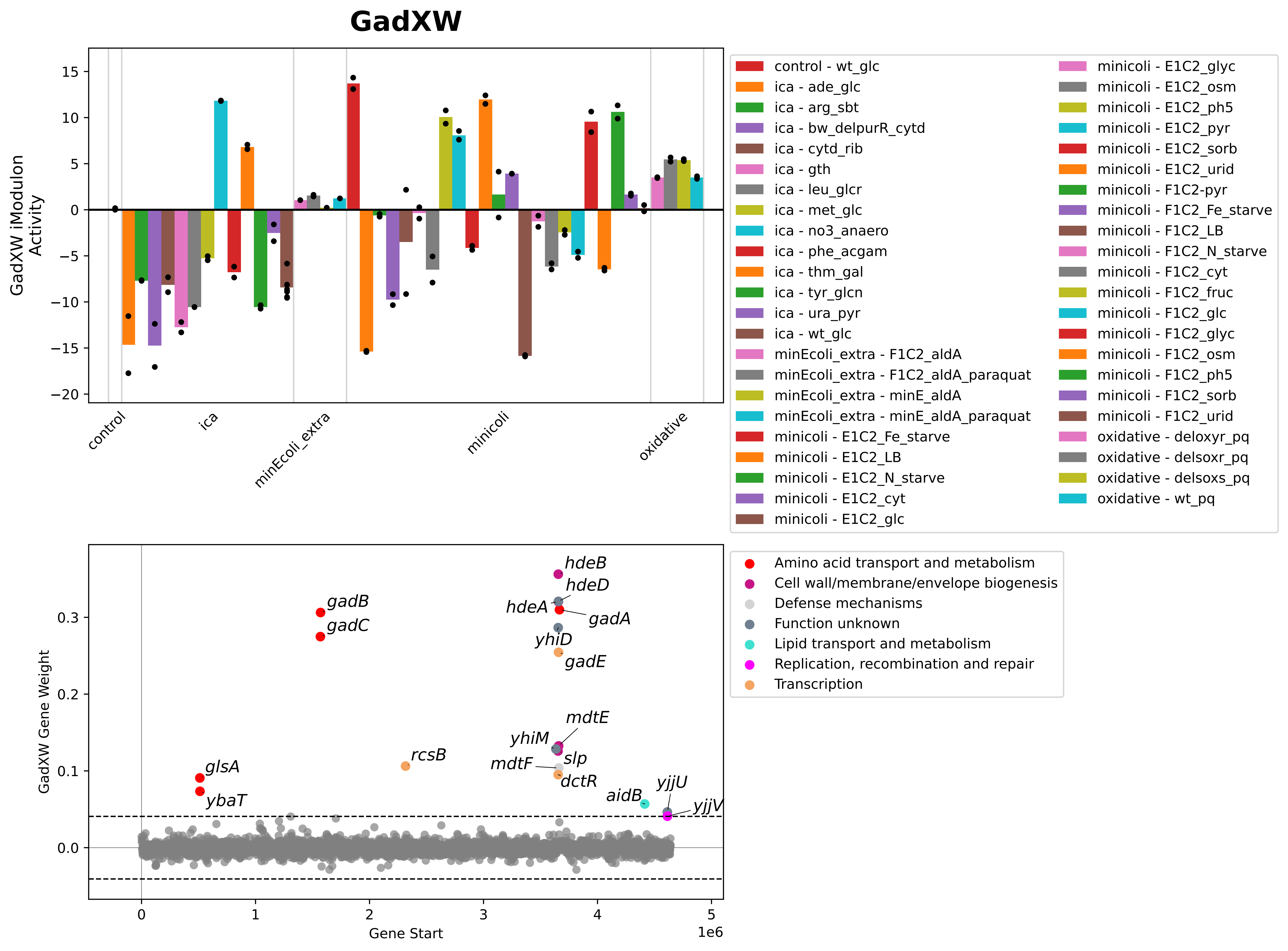
GadXW
GadXW iModulon Activity
control
ica
minEcoli_extra
minicoli
oxidative
control - wt_glc
ica - ade_glc
ica - arg_sbt
ica - bw_delpurR_cytd
ica - cytd_rib
ica - gth
ica - leu_glcr
ica - met_glc
ica - no3_anaero
ica - phe_acgam
ica - thm_gal
ica - tyr_glcn
ica - ura_pyr
ica - wt_glc
minEcoli_extra - F1C2_aldA
minEcoli_extra - F1C2_aldA_paraquat
minEcoli_extra - minE_aldA
minEcoli_extra - minE_aldA_paraquat
minicoli - E1C2_Fe_starve
minicoli - E1C2_LB
minicoli - E1C2_N_starve
minicoli - E1C2_cyt
minicoli - E1C2_glc
minicoli - E1C2_glyc
minicoli - E1C2_osm
minicoli - E1C2_ph5
minicoli - E1C2_pyr
minicoli - E1C2_sorb
minicoli - E1C2_urid
minicoli - F1C2-pyr
minicoli - F1C2_Fe_starve
minicoli - F1C2_LB
minicoli - F1C2_N_starve
minicoli - F1C2_cyt
minicoli - F1C2_fruc
minicoli - F1C2_glc
minicoli - F1C2_glyc
minicoli - F1C2_osm
minicoli - F1C2_ph5
minicoli - F1C2_sorb
minicoli - F1C2_urid
oxidative - deloxyr_pq
oxidative - delsoxr_pq
oxidative - delsoxs_pq
oxidative - wt_pq
GadXW Gene Weight
Gene Start
1e6
hdeB
hdeD
hdeA
gadA
gadB
gadC
yhiD
gadE
mdtE
yhiM
slp
mdtF
dctR
rcsB
glsA
ybaT
aidB
yjjU
yjjV
Amino acid transport and metabolism
Cell wall/membrane/envelope biogenesis
Defense mechanisms
Function unknown
Lipid transport and metabolism
Replication, recombination and repair
Transcription

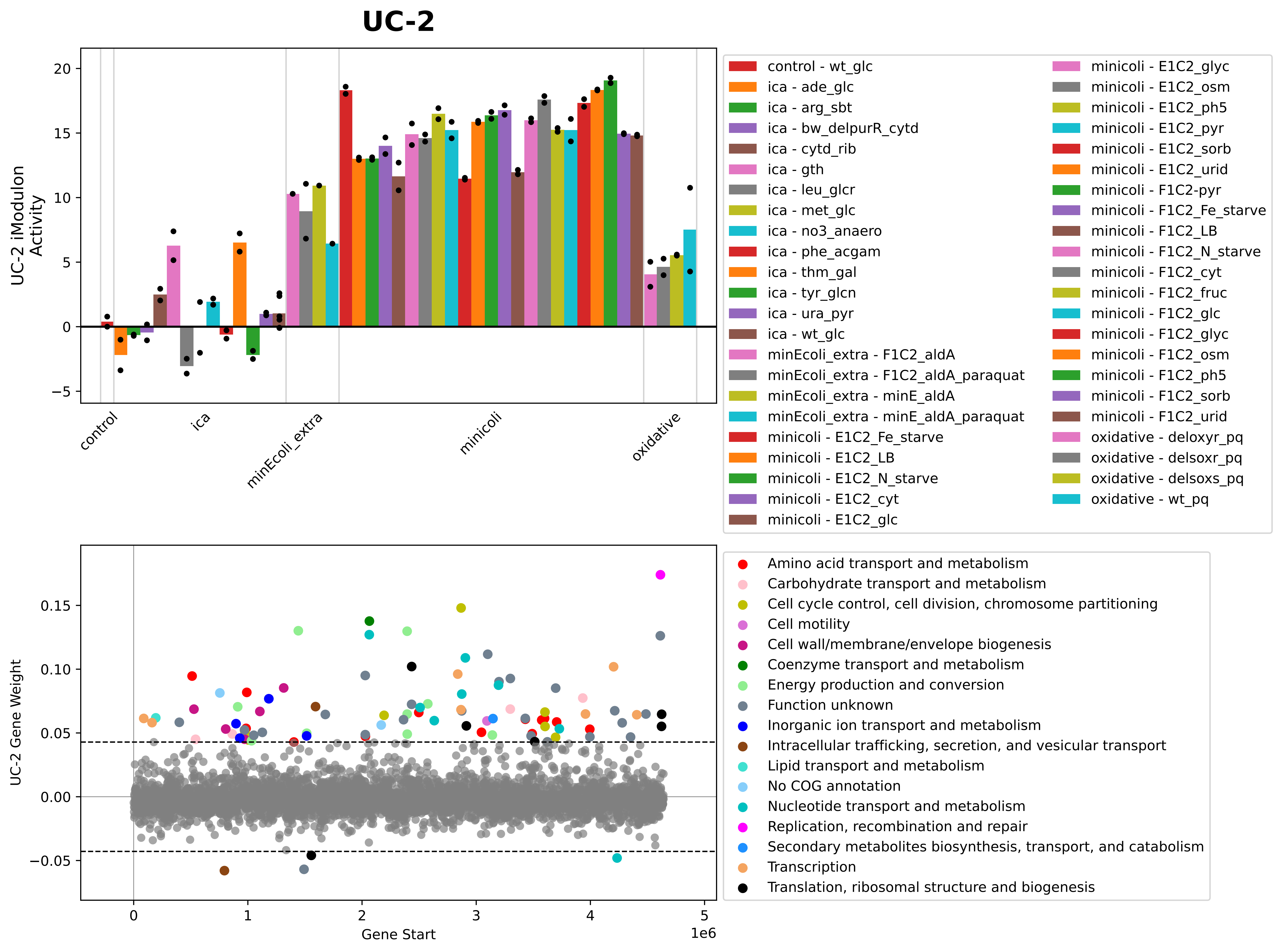
UC-2
UC-2 iModulon Activity
control
ica
minEcoli_extra
minicoli
oxidative
control - wt_glc
ica - ade_glc
ica - arg_sbt
ica - bw_delpurR_cytd
ica - cytd_rib
ica - gth
ica - leu_glcr
ica - met_glc
ica - no3_anaero
ica - phe_acgam
ica - thm_gal
ica - tyr_glcn
ica - ura_pyr
ica - wt_glc
minEcoli_extra - F1C2_aldA
minEcoli_extra - F1C2_aldA_paraquat
minEcoli_extra - minE_aldA
minEcoli_extra - minE_aldA_paraquat
minicoli - E1C2_Fe_starve
minicoli - E1C2_LB
minicoli - E1C2_N_starve
minicoli - E1C2_cyt
minicoli - E1C2_glc
minicoli - E1C2_glyc
minicoli - E1C2_osm
minicoli - E1C2_ph5
minicoli - E1C2_pyr
minicoli - E1C2_sorb
minicoli - E1C2_urid
minicoli - F1C2-pyr
minicoli - F1C2_Fe_starve
minicoli - F1C2_LB
minicoli - F1C2_N_starve
minicoli - F1C2_cyt
minicoli - F1C2_fruc
minicoli - F1C2_glc
minicoli - F1C2_glyc
minicoli - F1C2_osm
minicoli - F1C2_ph5
minicoli - F1C2_sorb
minicoli - F1C2_urid
oxidative - deloxyr_pq
oxidative - delsoxr_pq
oxidative - delsoxs_pq
oxidative - wt_pq
UC-2 Gene Weight
Gene Start
1e6
Amino acid transport and metabolism
Carbohydrate transport and metabolism
Cell cycle control, cell division, chromosome partitioning
Cell motility
Cell wall/membrane/envelope biogenesis
Coenzyme transport and metabolism
Energy production and conversion
Function unknown
Inorganic ion transport and metabolism
Intracellular trafficking, secretion, and vesicular transport
Lipid transport and metabolism
No COG annotation
Nucleotide transport and metabolism
Replication, recombination and repair
Secondary metabolites biosynthesis, transport, and catabolism
Transcription
Translation, ribosomal structure and biogenesis

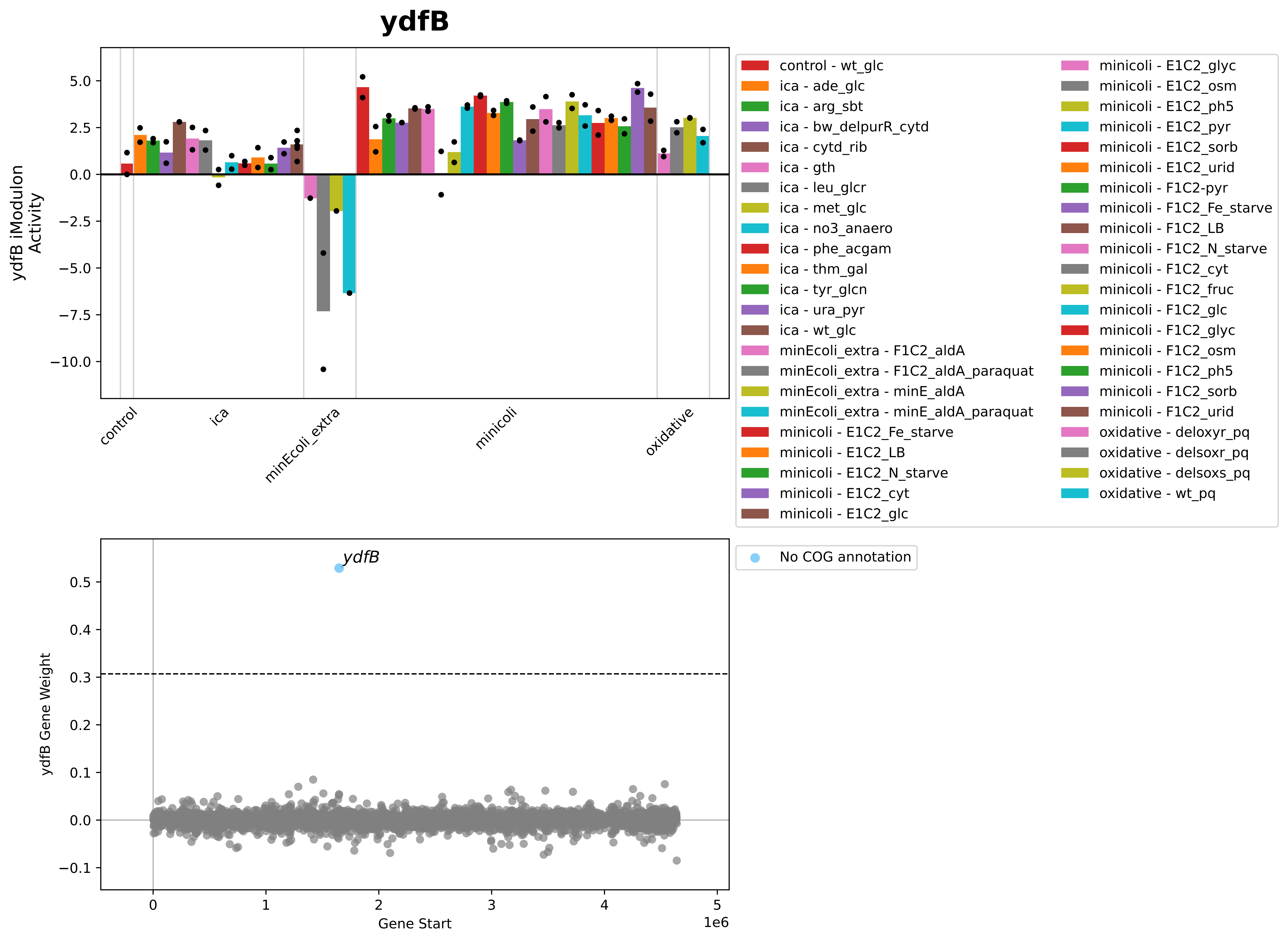
ydfB
ydfB iModulon Activity
5.0
2.5
0.0
−2.5
−5.0
−7.5
−10.0
control
ica
minEcoli_extra
minicoli
oxidative
control - wt_glc
ica - ade_glc
ica - arg_sbt
ica - bw_delpurR_cytd
ica - cytd_rib
ica - gth
ica - leu_glcr
ica - met_glc
ica - no3_anaero
ica - phe_acgam
ica - thm_gal
ica - tyr_glcn
ica - ura_pyr
ica - wt_glc
minEcoli_extra - F1C2_aldA
minEcoli_extra - F1C2_aldA_paraquat
minEcoli_extra - minE_aldA
minEcoli_extra - minE_aldA_paraquat
minicoli - E1C2_Fe_starve
minicoli - E1C2_LB
minicoli - E1C2_N_starve
minicoli - E1C2_cyt
minicoli - E1C2_glc
minicoli - E1C2_glyc
minicoli - E1C2_osm
minicoli - E1C2_ph5
minicoli - E1C2_pyr
minicoli - E1C2_sorb
minicoli - E1C2_urid
minicoli - F1C2-pyr
minicoli - F1C2_Fe_starve
minicoli - F1C2_LB
minicoli - F1C2_N_starve
minicoli - F1C2_cyt
minicoli - F1C2_fruc
minicoli - F1C2_glc
minicoli - F1C2_glyc
minicoli - F1C2_osm
minicoli - F1C2_ph5
minicoli - F1C2_sorb
minicoli - F1C2_urid
oxidative - deloxyr_pq
oxidative - delsoxr_pq
oxidative - delsoxs_pq
oxidative - wt_pq
ydfB
ydfB Gene Weight
0.5
0.4
0.3
0.2
0.1
0.0
−0.1
0
1
2
3
4
5
Gene Start
1e6
No COG annotation

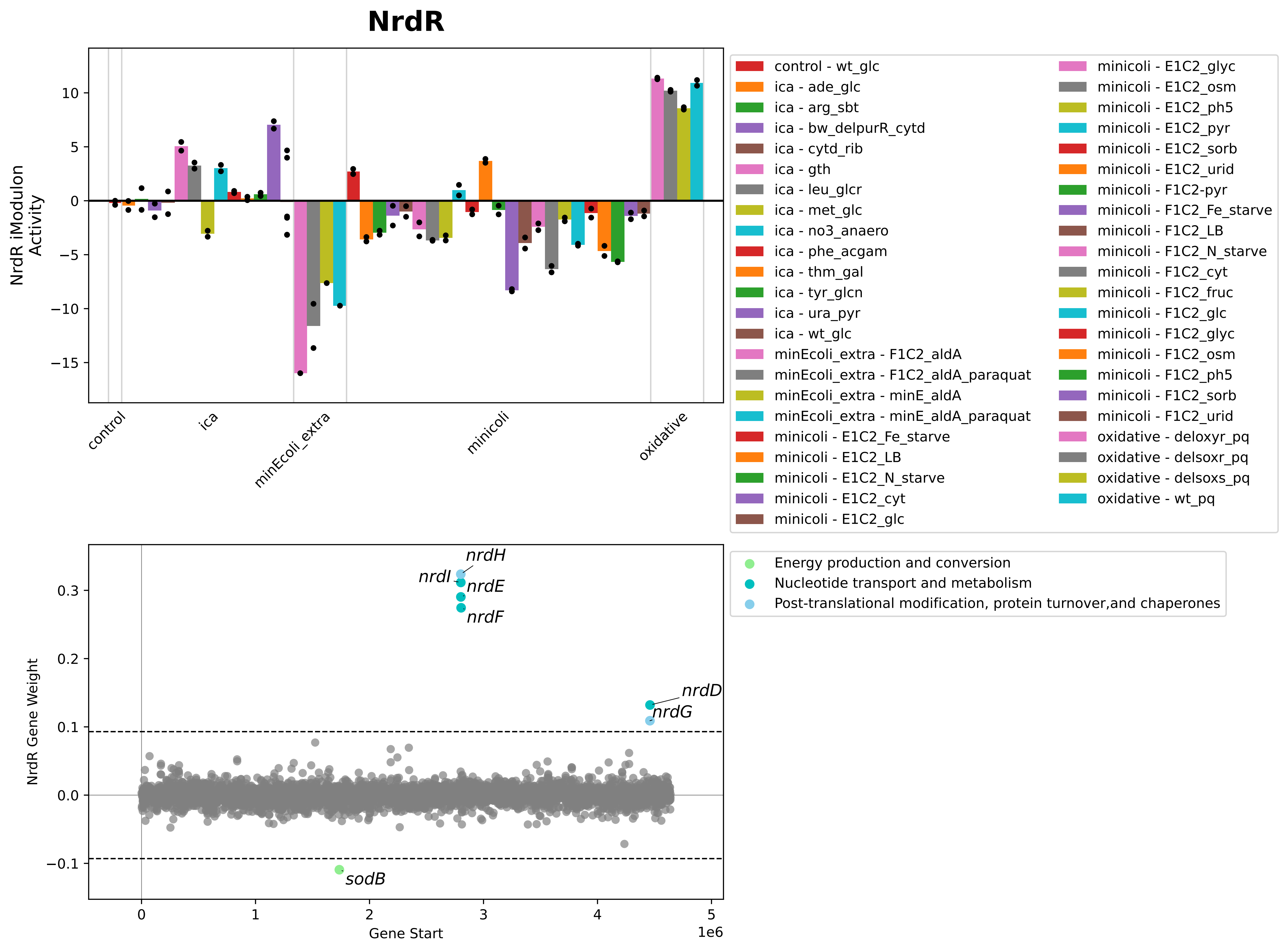
NrdR
NrdR iModulon Activity
control
ica
minEcoli_extra
minicoli
oxidative
control - wt_glc
ica - ade_glc
ica - arg_sbt
ica - bw_delpurR_cytd
ica - cytd_rib
ica - gth
ica - leu_glcr
ica - met_glc
ica - no3_anaero
ica - phe_acgam
ica - thm_gal
ica - tyr_glcn
ica - ura_pyr
ica - wt_glc
minEcoli_extra - F1C2_aldA
minEcoli_extra - F1C2_aldA_paraquat
minEcoli_extra - minE_aldA
minEcoli_extra - minE_aldA_paraquat
minicoli - E1C2_Fe_starve
minicoli - E1C2_LB
minicoli - E1C2_N_starve
minicoli - E1C2_cyt
minicoli - E1C2_glc
minicoli - E1C2_glyc
minicoli - E1C2_osm
minicoli - E1C2_ph5
minicoli - E1C2_pyr
minicoli - E1C2_sorb
minicoli - E1C2_urid
minicoli - F1C2-pyr
minicoli - F1C2_Fe_starve
minicoli - F1C2_LB
minicoli - F1C2_N_starve
minicoli - F1C2_cyt
minicoli - F1C2_fruc
minicoli - F1C2_glc
minicoli - F1C2_glyc
minicoli - F1C2_osm
minicoli - F1C2_ph5
minicoli - F1C2_sorb
minicoli - F1C2_urid
oxidative - deloxyr_pq
oxidative - delsoxr_pq
oxidative - delsoxs_pq
oxidative - wt_pq
NrdR Gene Weight
Gene Start
1e6
nrdH
nrdI
nrdE
nrdF
nrdD
nrdG
sodB
Energy production and conversion
Nucleotide transport and metabolism
Post-translational modification, protein turnover,and chaperones

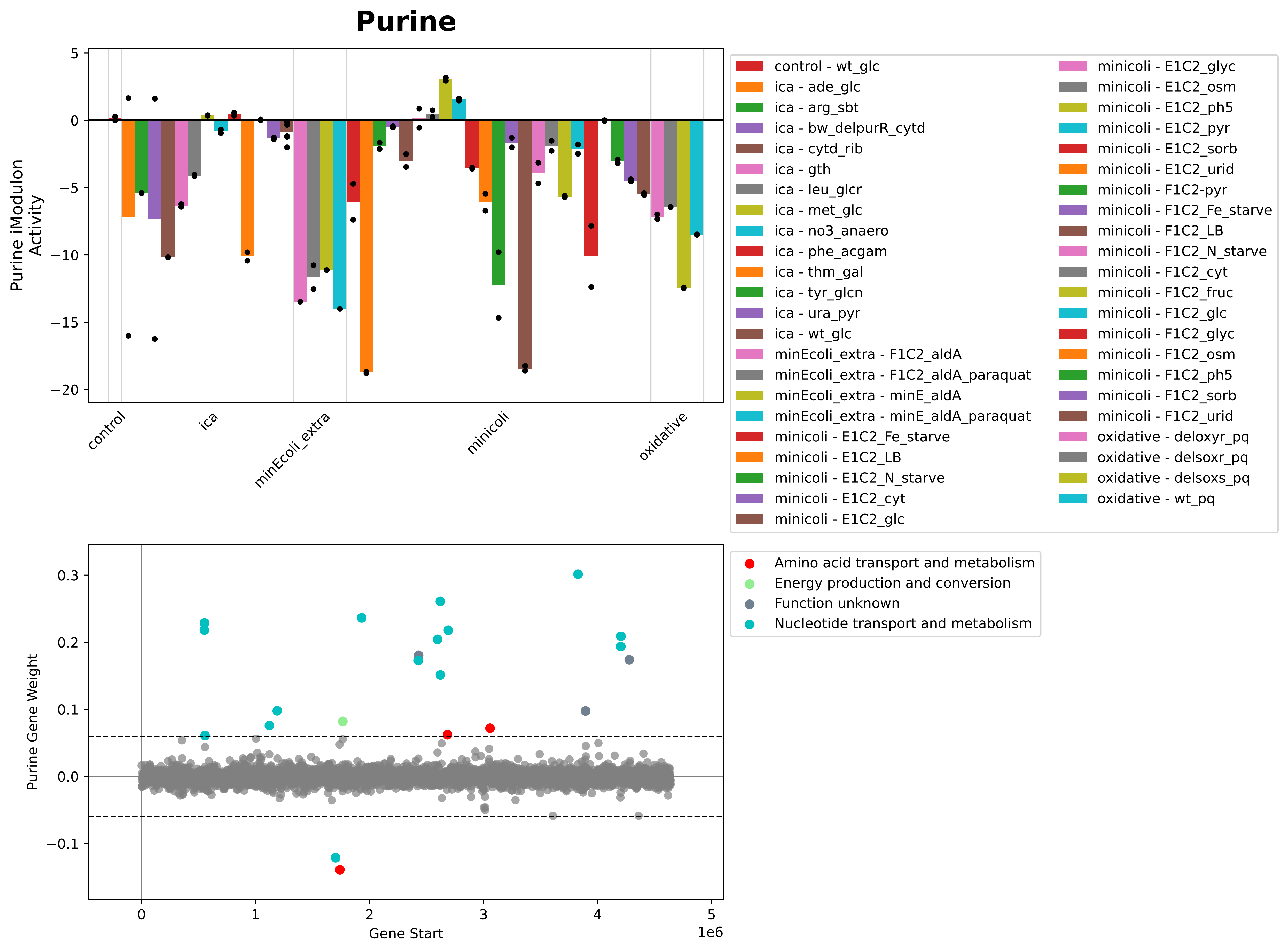

Purine
Purine iModulon Activity
control
ica
minEcoli_extra
minicoli
oxidative
control - wt_glc
ica - ade_glc
ica - arg_sbt
ica - bw_delpurR_cytd
ica - cytd_rib
ica - gth
ica - leu_glcr
ica - met_glc
ica - no3_anaero
ica - phe_acgam
ica - thm_gal
ica - tyr_glcn
ica - ura_pyr
ica - wt_glc
minEcoli_extra - F1C2_aldA
minEcoli_extra - F1C2_aldA_paraquat
minEcoli_extra - minE_aldA
minEcoli_extra - minE_aldA_paraquat
minicoli - E1C2_Fe_starve
minicoli - E1C2_LB
minicoli - E1C2_N_starve
minicoli - E1C2_cyt
minicoli - E1C2_glc
minicoli - E1C2_glyc
minicoli - E1C2_osm
minicoli - E1C2_ph5
minicoli - E1C2_pyr
minicoli - E1C2_sorb
minicoli - E1C2_urid
minicoli - F1C2-pyr
minicoli - F1C2_Fe_starve
minicoli - F1C2_LB
minicoli - F1C2_N_starve
minicoli - F1C2_cyt
minicoli - F1C2_fruc
minicoli - F1C2_glc
minicoli - F1C2_glyc
minicoli - F1C2_osm
minicoli - F1C2_ph5
minicoli - F1C2_sorb
minicoli - F1C2_urid
oxidative - deloxyr_pq
oxidative - delsoxr_pq
oxidative - delsoxs_pq
oxidative - wt_pq
Purine Gene Weight
Gene Start
1e6
Amino acid transport and metabolism
Energy production and conversion
Function unknown
Nucleotide transport and metabolism

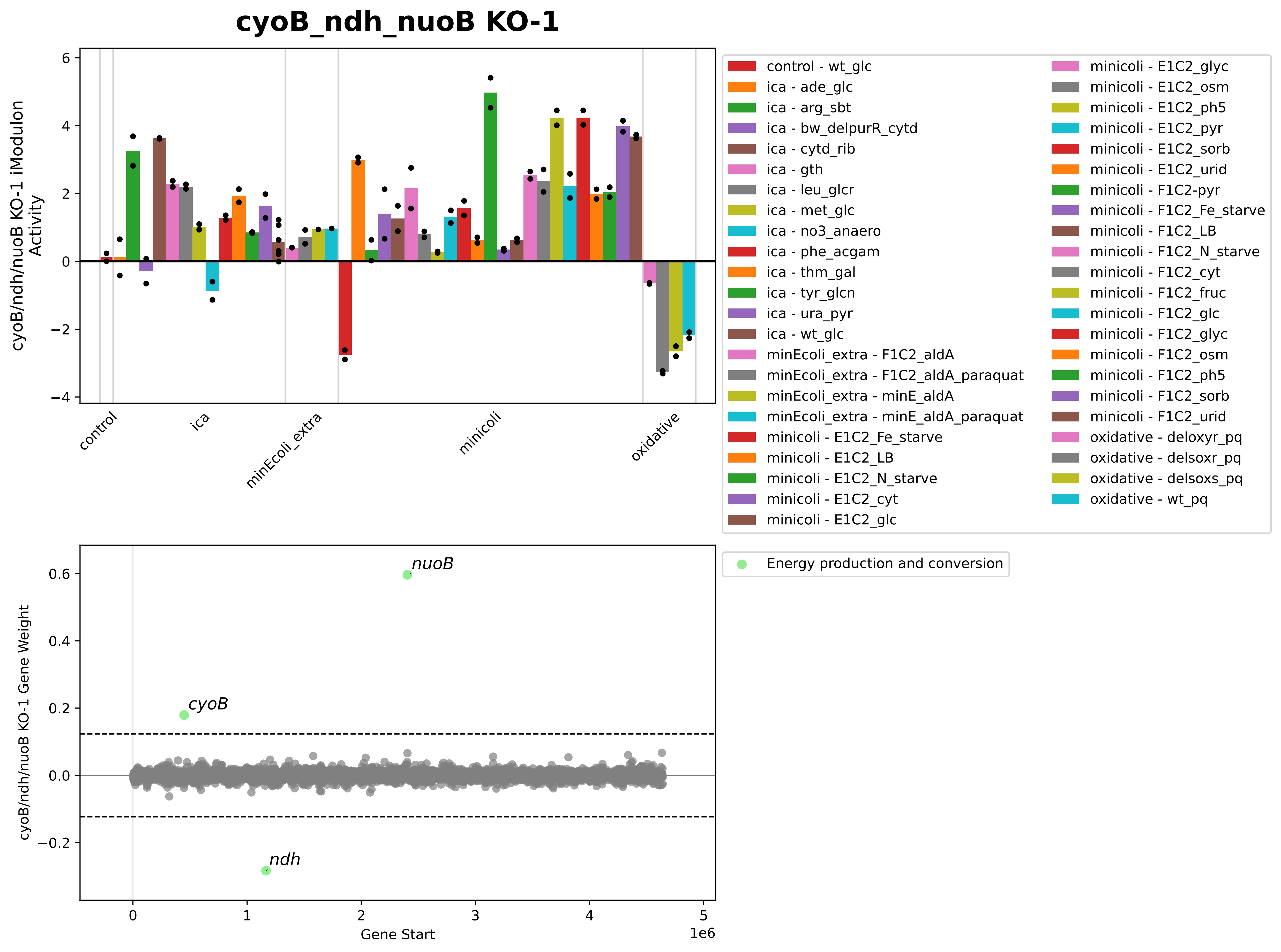
cyoB_ndh_nuoB KO-1
cyoB/ndh/nuoB KO-1 iModulon Activity
control
ica
minEcoli_extra
minicoli
oxidative
control - wt_glc
ica - ade_glc
ica - arg_sbt
ica - bw_delpurR_cytd
ica - cytd_rib
ica - gth
ica - leu_glcr
ica - met_glc
ica - no3_anaero
ica - phe_acgam
ica - thm_gal
ica - tyr_glcn
ica - ura_pyr
ica - wt_glc
minEcoli_extra - F1C2_aldA
minEcoli_extra - F1C2_aldA_paraquat
minEcoli_extra - minE_aldA
minEcoli_extra - minE_aldA_paraquat
minicoli - E1C2_Fe_starve
minicoli - E1C2_LB
minicoli - E1C2_N_starve
minicoli - E1C2_cyt
minicoli - E1C2_glc
minicoli - E1C2_glyc
minicoli - E1C2_osm
minicoli - E1C2_ph5
minicoli - E1C2_pyr
minicoli - E1C2_sorb
minicoli - E1C2_urid
minicoli - F1C2-pyr
minicoli - F1C2_Fe_starve
minicoli - F1C2_LB
minicoli - F1C2_N_starve
minicoli - F1C2_cyt
minicoli - F1C2_fruc
minicoli - F1C2_glc
minicoli - F1C2_glyc
minicoli - F1C2_osm
minicoli - F1C2_ph5
minicoli - F1C2_sorb
minicoli - F1C2_urid
oxidative - deloxyr_pq
oxidative - delsoxr_pq
oxidative - delsoxs_pq
oxidative - wt_pq
nuoB
cyoB
ndh
Energy production and conversion
cyoB/ndh/nuoB KO-1 Gene Weight
Gene Start
1e6

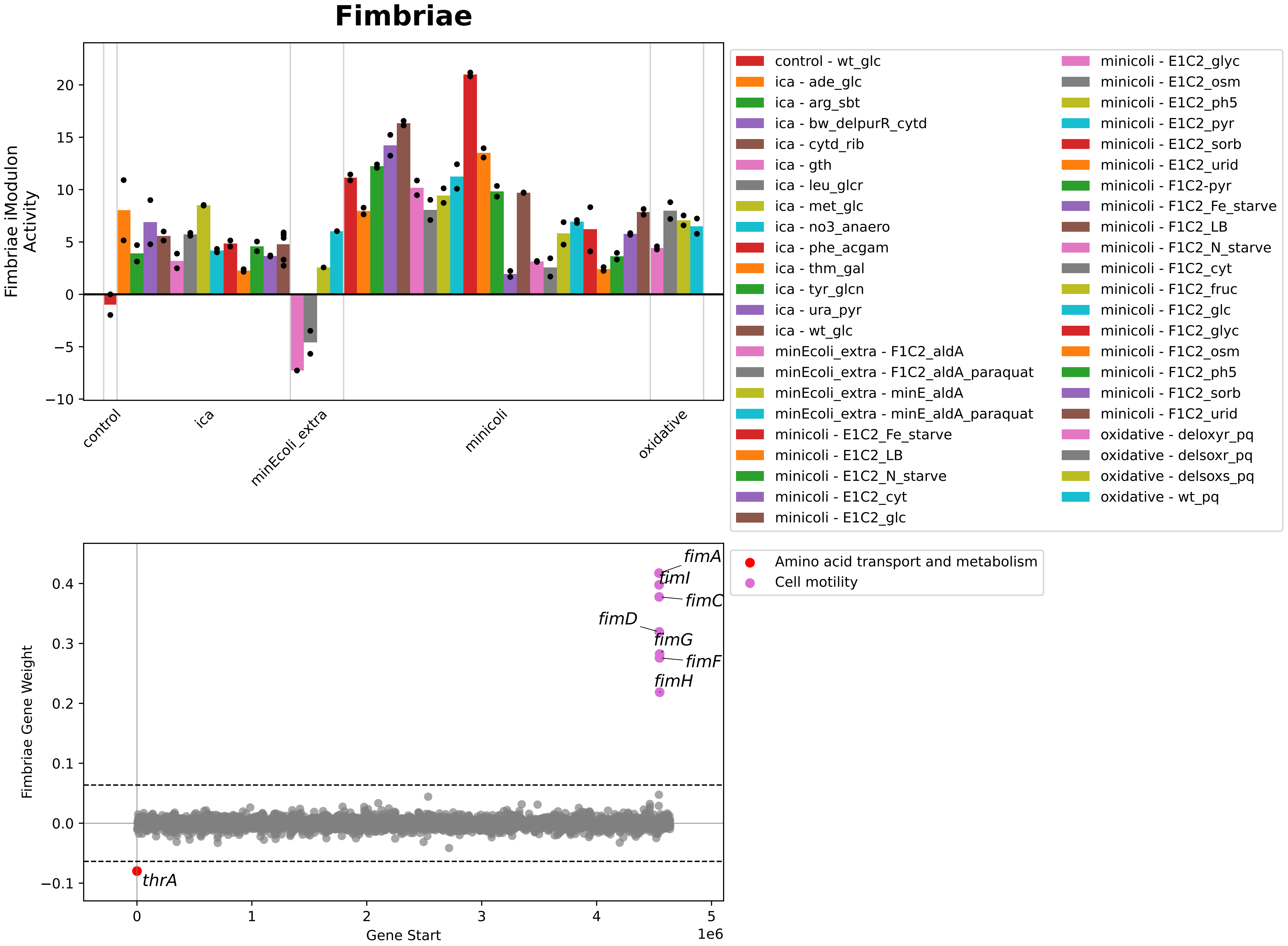

Fimbriae
Fimbriae iModulon Activity
control
ica
minEcoli_extra
minicoli
oxidative
control - wt_glc
ica - ade_glc
ica - arg_sbt
ica - bw_delpurR_cytd
ica - cytd_rib
ica - gth
ica - leu_glcr
ica - met_glc
ica - no3_anaero
ica - phe_acgam
ica - thm_gal
ica - tyr_glcn
ica - ura_pyr
ica - wt_glc
minEcoli_extra - F1C2_aldA
minEcoli_extra - F1C2_aldA_paraquat
minEcoli_extra - minE_aldA
minEcoli_extra - minE_aldA_paraquat
minicoli - E1C2_Fe_starve
minicoli - E1C2_LB
minicoli - E1C2_N_starve
minicoli - E1C2_cyt
minicoli - E1C2_glc
minicoli - E1C2_glyc
minicoli - E1C2_osm
minicoli - E1C2_ph5
minicoli - E1C2_pyr
minicoli - E1C2_sorb
minicoli - E1C2_urid
minicoli - F1C2-pyr
minicoli - F1C2_Fe_starve
minicoli - F1C2_LB
minicoli - F1C2_N_starve
minicoli - F1C2_cyt
minicoli - F1C2_fruc
minicoli - F1C2_glc
minicoli - F1C2_glyc
minicoli - F1C2_osm
minicoli - F1C2_ph5
minicoli - F1C2_sorb
minicoli - F1C2_urid
oxidative - deloxyr_pq
oxidative - delsoxr_pq
oxidative - delsoxs_pq
oxidative - wt_pq
Fimbriae Gene Weight
Gene Start
1e6
fimA
fimI
fimC
fimD
fimG
fimF
fimH
thrA
Amino acid transport and metabolism
Cell motility

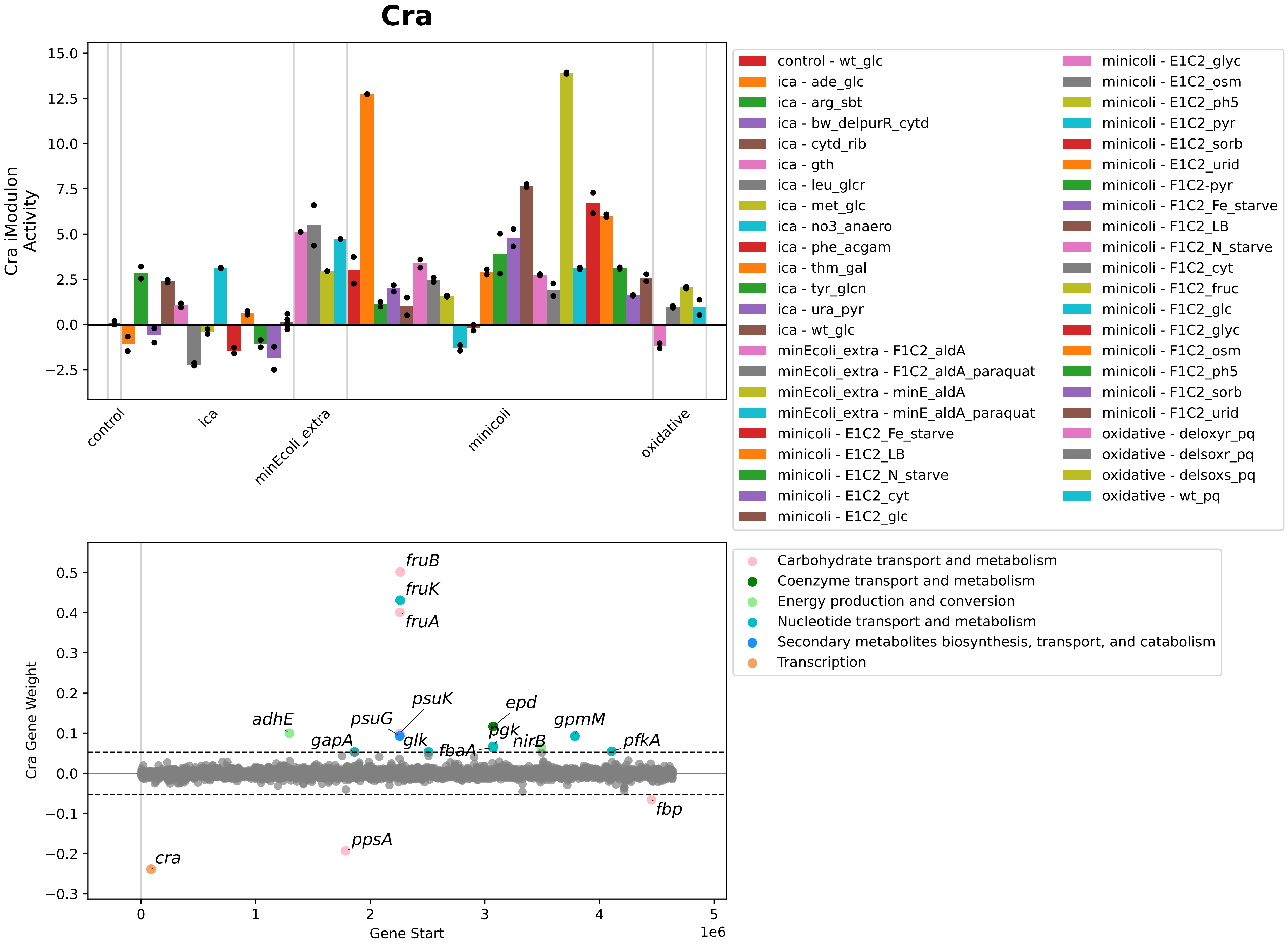

Cra
Cra iModulon Activity
15.0
12.5
10.0
7.5
5.0
2.5
0.0
−2.5
control
ica
minEcoli_extra
minicoli
oxidative
control - wt_glc
ica - ade_glc
ica - arg_sbt
ica - bw_delpurR_cytd
ica - cytd_rib
ica - gth
ica - leu_glcr
ica - met_glc
ica - no3_anaero
ica - phe_acgam
ica - thm_gal
ica - tyr_glcn
ica - ura_pyr
ica - wt_glc
minEcoli_extra - F1C2_aldA
minEcoli_extra - F1C2_aldA_paraquat
minEcoli_extra - minE_aldA
minEcoli_extra - minE_aldA_paraquat
minicoli - E1C2_Fe_starve
minicoli - E1C2_LB
minicoli - E1C2_N_starve
minicoli - E1C2_cyt
minicoli - E1C2_glc
minicoli - E1C2_glyc
minicoli - E1C2_osm
minicoli - E1C2_ph5
minicoli - E1C2_pyr
minicoli - E1C2_sorb
minicoli - E1C2_urid
minicoli - F1C2-pyr
minicoli - F1C2_Fe_starve
minicoli - F1C2_LB
minicoli - F1C2_N_starve
minicoli - F1C2_cyt
minicoli - F1C2_fruc
minicoli - F1C2_glc
minicoli - F1C2_glyc
minicoli - F1C2_osm
minicoli - F1C2_ph5
minicoli - F1C2_sorb
minicoli - F1C2_urid
oxidative - deloxyr_pq
oxidative - delsoxr_pq
oxidative - delsoxs_pq
oxidative - wt_pq
Cra Gene Weight
0.5
0.4
0.3
0.2
0.1
0.0
−0.1
−0.2
−0.3
fruB
fruK
fruA
psuK
epd
adhE
psuG
gpmM
pgk
gapA
glk
nirB
pfkA
fbaA
fbp
ppsA
cra
0
1
2
3
4
5
Gene Start
1e6
Carbohydrate transport and metabolism
Coenzyme transport and metabolism
Energy production and conversion
Nucleotide transport and metabolism
Secondary metabolites biosynthesis, transport, and catabolism
Transcription

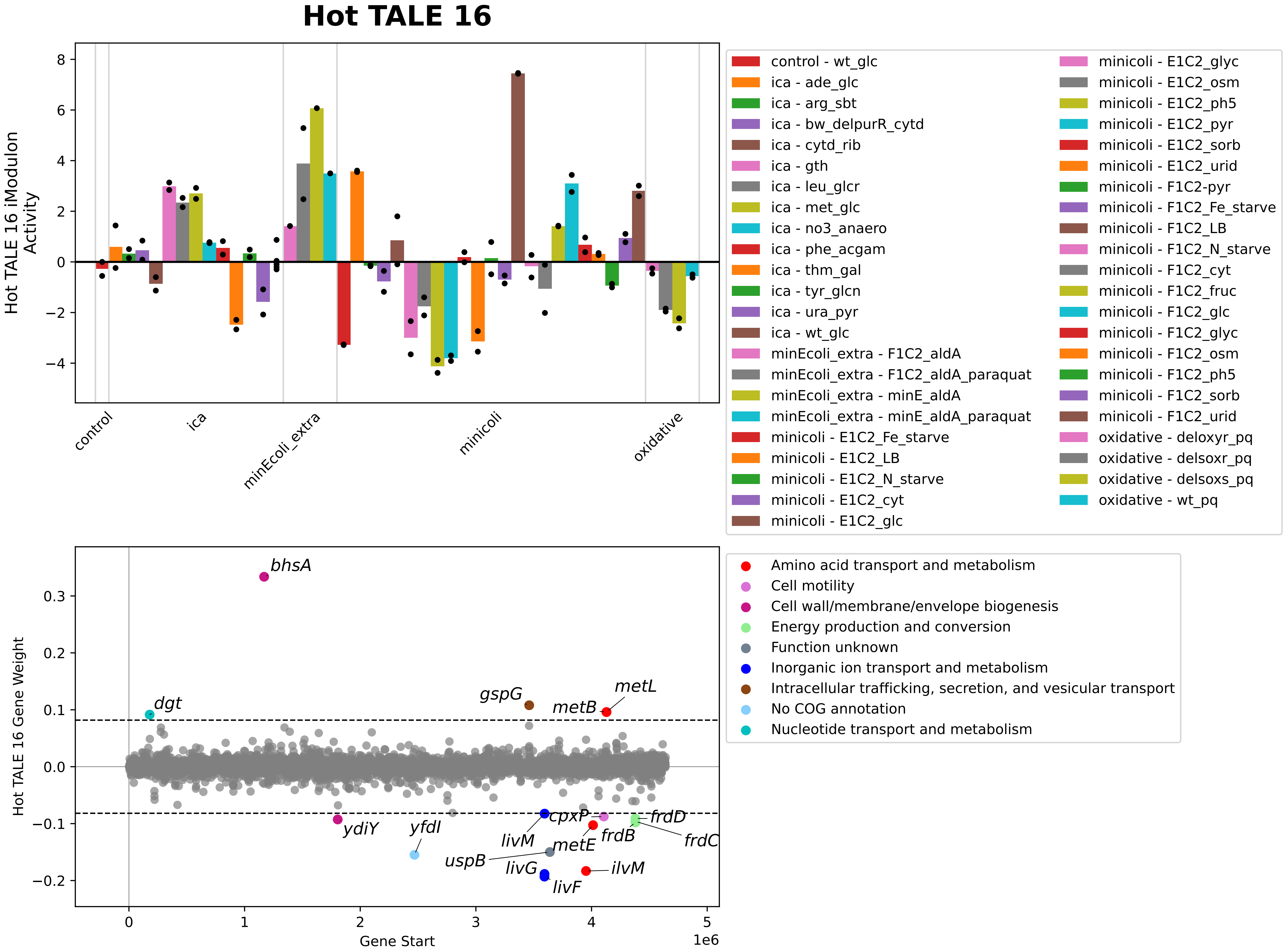
Hot TALE 16
Hot TALE 16 iModulon Activity
control
ica
minEcoli_extra
minicoli
oxidative
control - wt_glc
ica - ade_glc
ica - arg_sbt
ica - bw_delpurR_cytd
ica - cytd_rib
ica - gth
ica - leu_glcr
ica - met_glc
ica - no3_anaero
ica - phe_acgam
ica - thm_gal
ica - tyr_glcn
ica - ura_pyr
ica - wt_glc
minEcoli_extra - F1C2_aldA
minEcoli_extra - F1C2_aldA_paraquat
minEcoli_extra - minE_aldA
minEcoli_extra - minE_aldA_paraquat
minicoli - E1C2_Fe_starve
minicoli - E1C2_LB
minicoli - E1C2_N_starve
minicoli - E1C2_cyt
minicoli - E1C2_glc
minicoli - E1C2_glyc
minicoli - E1C2_osm
minicoli - E1C2_ph5
minicoli - E1C2_pyr
minicoli - E1C2_sorb
minicoli - E1C2_urid
minicoli - F1C2-pyr
minicoli - F1C2_Fe_starve
minicoli - F1C2_LB
minicoli - F1C2_N_starve
minicoli - F1C2_cyt
minicoli - F1C2_fruc
minicoli - F1C2_glc
minicoli - F1C2_glyc
minicoli - F1C2_osm
minicoli - F1C2_ph5
minicoli - F1C2_sorb
minicoli - F1C2_urid
oxidative - deloxyr_pq
oxidative - delsoxr_pq
oxidative - delsoxs_pq
oxidative - wt_pq
Hot TALE 16 Gene Weight
Gene Start
1e6
bhsA
dgt
gspG
metB
metL
ydiY
yfdI
cpxP
frdD
frdB
frdC
livM
metE
uspB
livG
livF
ilvM
Amino acid transport and metabolism
Cell motility
Cell wall/membrane/envelope biogenesis
Energy production and conversion
Function unknown
Inorganic ion transport and metabolism
Intracellular trafficking, secretion, and vesicular transport
No COG annotation
Nucleotide transport and metabolism

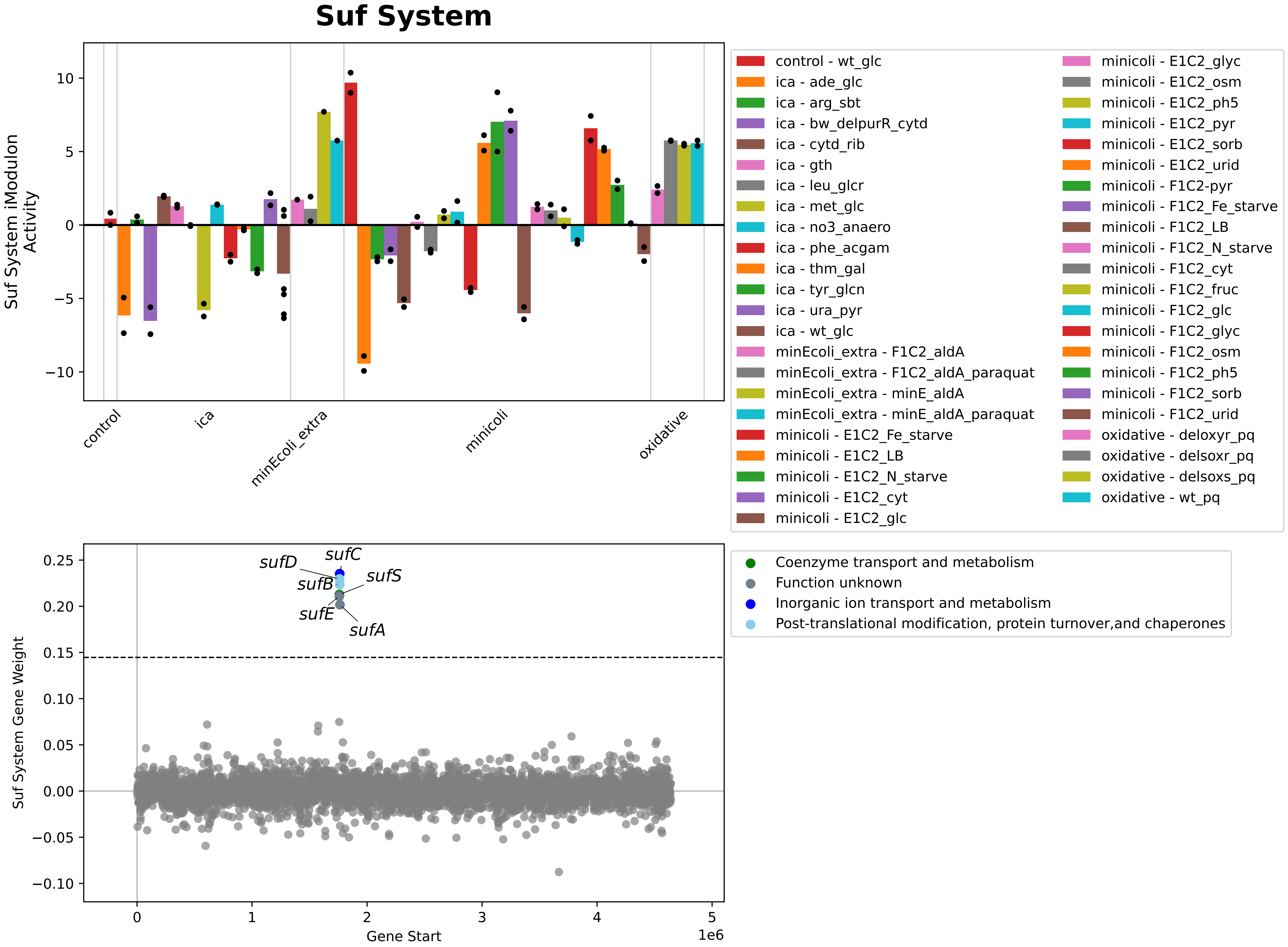

Suf System
Suf System iModulon Activity
control
ica
minEcoli_extra
minicoli
oxidative
control - wt_glc
ica - ade_glc
ica - arg_sbt
ica - bw_delpurR_cytd
ica - cytd_rib
ica - gth
ica - leu_glcr
ica - met_glc
ica - no3_anaero
ica - phe_acgam
ica - thm_gal
ica - tyr_glcn
ica - ura_pyr
ica - wt_glc
minEcoli_extra - F1C2_aldA
minEcoli_extra - F1C2_aldA_paraquat
minEcoli_extra - minE_aldA
minEcoli_extra - minE_aldA_paraquat
minicoli - E1C2_Fe_starve
minicoli - E1C2_LB
minicoli - E1C2_N_starve
minicoli - E1C2_cyt
minicoli - E1C2_glc
minicoli - E1C2_glyc
minicoli - E1C2_osm
minicoli - E1C2_ph5
minicoli - E1C2_pyr
minicoli - E1C2_sorb
minicoli - E1C2_urid
minicoli - F1C2-pyr
minicoli - F1C2_Fe_starve
minicoli - F1C2_LB
minicoli - F1C2_N_starve
minicoli - F1C2_cyt
minicoli - F1C2_fruc
minicoli - F1C2_glc
minicoli - F1C2_glyc
minicoli - F1C2_osm
minicoli - F1C2_ph5
minicoli - F1C2_sorb
minicoli - F1C2_urid
oxidative - deloxyr_pq
oxidative - delsoxr_pq
oxidative - delsoxs_pq
oxidative - wt_pq
sufC
sufD
sufS
sufB
sufE
sufA
Suf System Gene Weight
Gene Start
1e6
Coenzyme transport and metabolism
Function unknown
Inorganic ion transport and metabolism
Post-translational modification, protein turnover,and chaperones

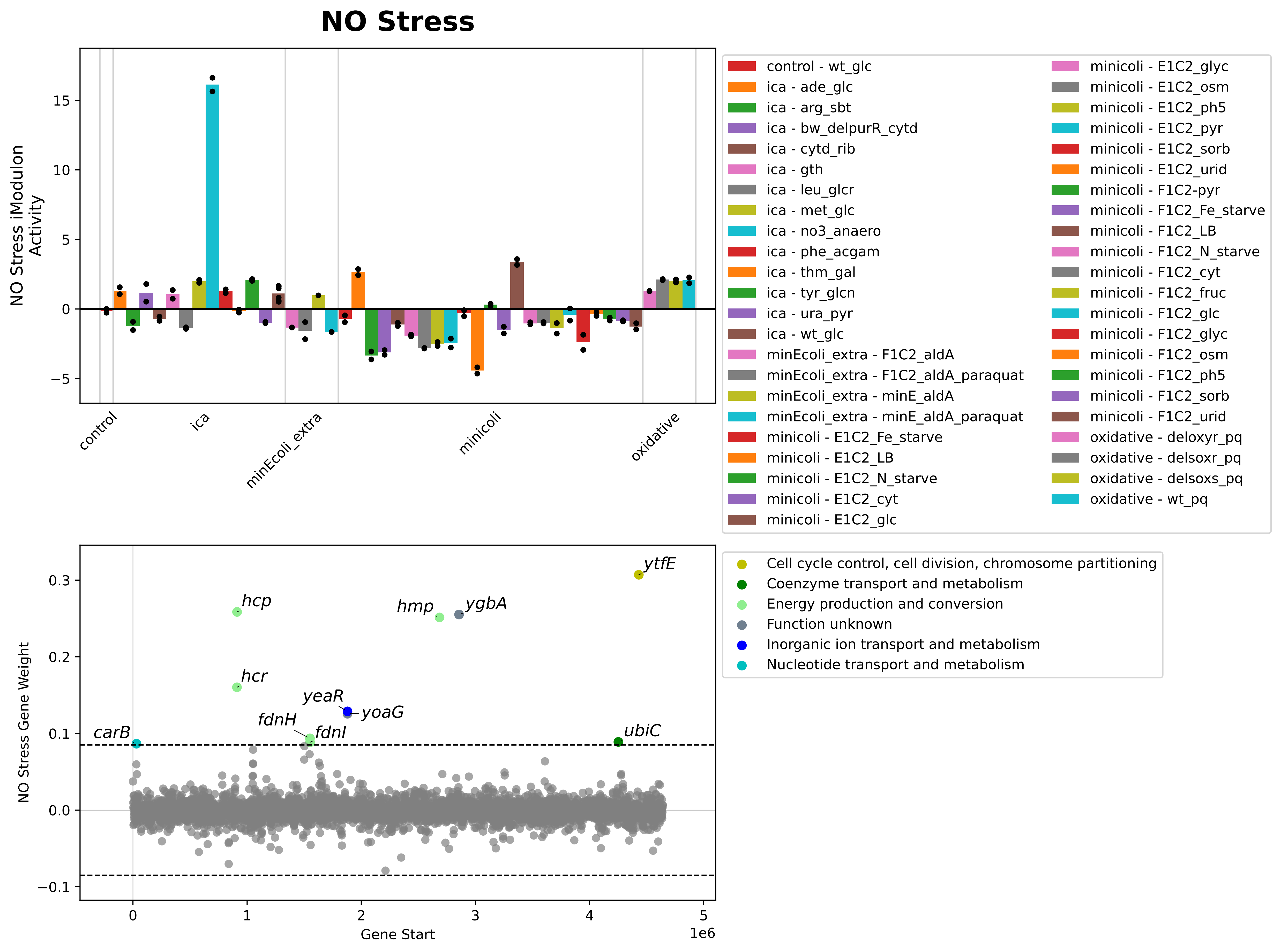
NO Stress
NO Stress iModulon Activity
control
ica
minEcoli_extra
minicoli
oxidative
control - wt_glc
ica - ade_glc
ica - arg_sbt
ica - bw_delpurR_cytd
ica - cytd_rib
ica - gth
ica - leu_glcr
ica - met_glc
ica - no3_anaero
ica - phe_acgam
ica - thm_gal
ica - tyr_glcn
ica - ura_pyr
ica - wt_glc
minEcoli_extra - F1C2_aldA
minEcoli_extra - F1C2_aldA_paraquat
minEcoli_extra - minE_aldA
minEcoli_extra - minE_aldA_paraquat
minicoli - E1C2_Fe_starve
minicoli - E1C2_LB
minicoli - E1C2_N_starve
minicoli - E1C2_cyt
minicoli - E1C2_glc
minicoli - E1C2_glyc
minicoli - E1C2_osm
minicoli - E1C2_ph5
minicoli - E1C2_pyr
minicoli - E1C2_sorb
minicoli - E1C2_urid
minicoli - F1C2-pyr
minicoli - F1C2_Fe_starve
minicoli - F1C2_LB
minicoli - F1C2_N_starve
minicoli - F1C2_cyt
minicoli - F1C2_fruc
minicoli - F1C2_glc
minicoli - F1C2_glyc
minicoli - F1C2_osm
minicoli - F1C2_ph5
minicoli - F1C2_sorb
minicoli - F1C2_urid
oxidative - deloxyr_pq
oxidative - delsoxr_pq
oxidative - delsoxs_pq
oxidative - wt_pq
NO Stress Gene Weight
Gene Start
1e6
ytfE
hcp
hmp
ygbA
hcr
yeaR
yoaG
fdnH
fdnI
carB
ubiC
Cell cycle control, cell division, chromosome partitioning
Coenzyme transport and metabolism
Energy production and conversion
Function unknown
Inorganic ion transport and metabolism
Nucleotide transport and metabolism

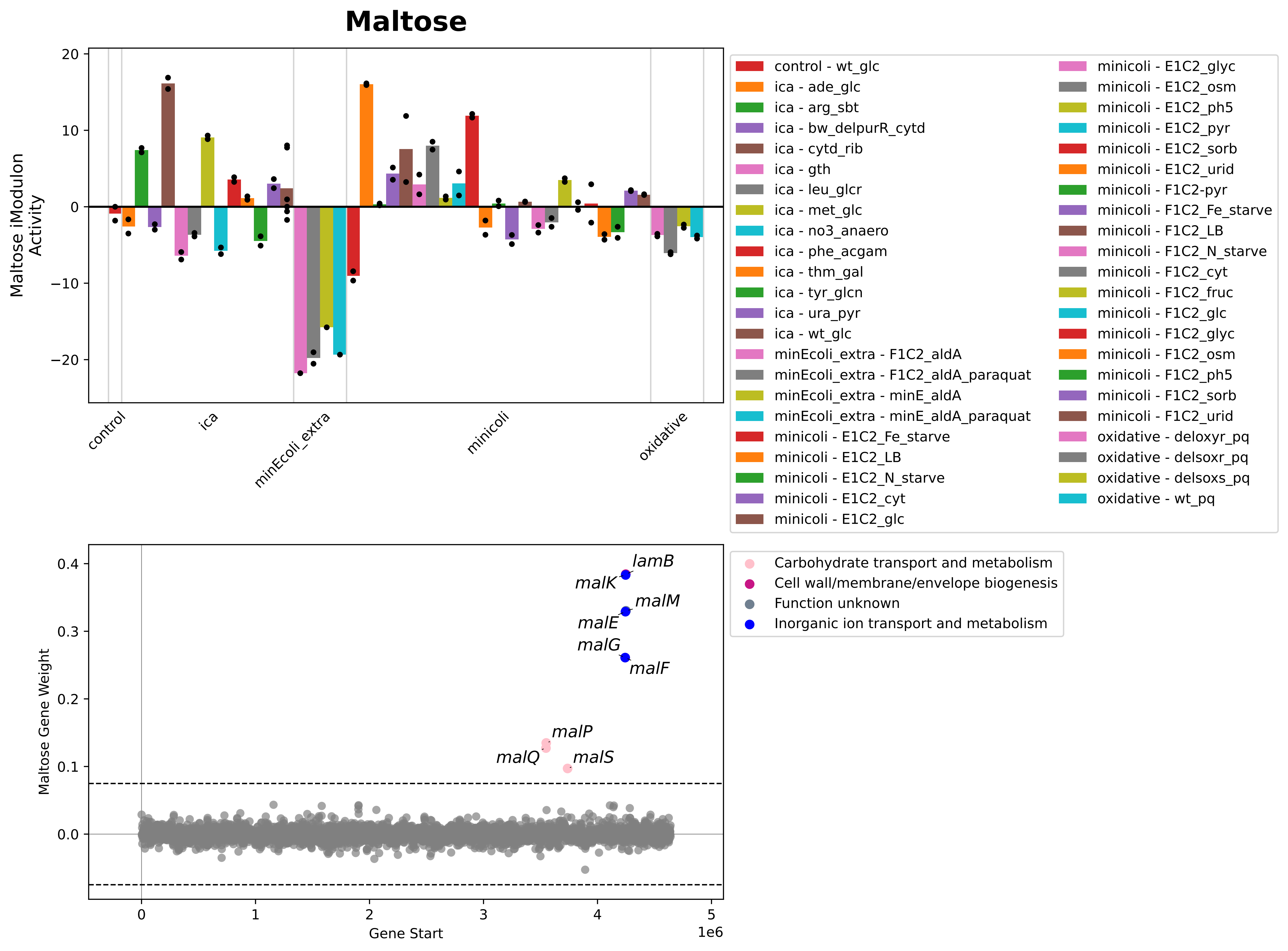
Maltose
Maltose iModulon Activity
control
ica
minEcoli_extra
minicoli
oxidative
control - wt_glc
ica - ade_glc
ica - arg_sbt
ica - bw_delpurR_cytd
ica - cytd_rib
ica - gth
ica - leu_glcr
ica - met_glc
ica - no3_anaero
ica - phe_acgam
ica - thm_gal
ica - tyr_glcn
ica - ura_pyr
ica - wt_glc
minEcoli_extra - F1C2_aldA
minEcoli_extra - F1C2_aldA_paraquat
minEcoli_extra - minE_aldA
minEcoli_extra - minE_aldA_paraquat
minicoli - E1C2_Fe_starve
minicoli - E1C2_LB
minicoli - E1C2_N_starve
minicoli - E1C2_cyt
minicoli - E1C2_glc
minicoli - E1C2_glyc
minicoli - E1C2_osm
minicoli - E1C2_ph5
minicoli - E1C2_pyr
minicoli - E1C2_sorb
minicoli - E1C2_urid
minicoli - F1C2-pyr
minicoli - F1C2_Fe_starve
minicoli - F1C2_LB
minicoli - F1C2_N_starve
minicoli - F1C2_cyt
minicoli - F1C2_fruc
minicoli - F1C2_glc
minicoli - F1C2_glyc
minicoli - F1C2_osm
minicoli - F1C2_ph5
minicoli - F1C2_sorb
minicoli - F1C2_urid
oxidative - deloxyr_pq
oxidative - delsoxr_pq
oxidative - delsoxs_pq
oxidative - wt_pq
Maltose Gene Weight
Gene Start
1e6
lamB
malK
malM
malE
malG
malF
malP
malQ
malS
Carbohydrate transport and metabolism
Cell wall/membrane/envelope biogenesis
Function unknown
Inorganic ion transport and metabolism

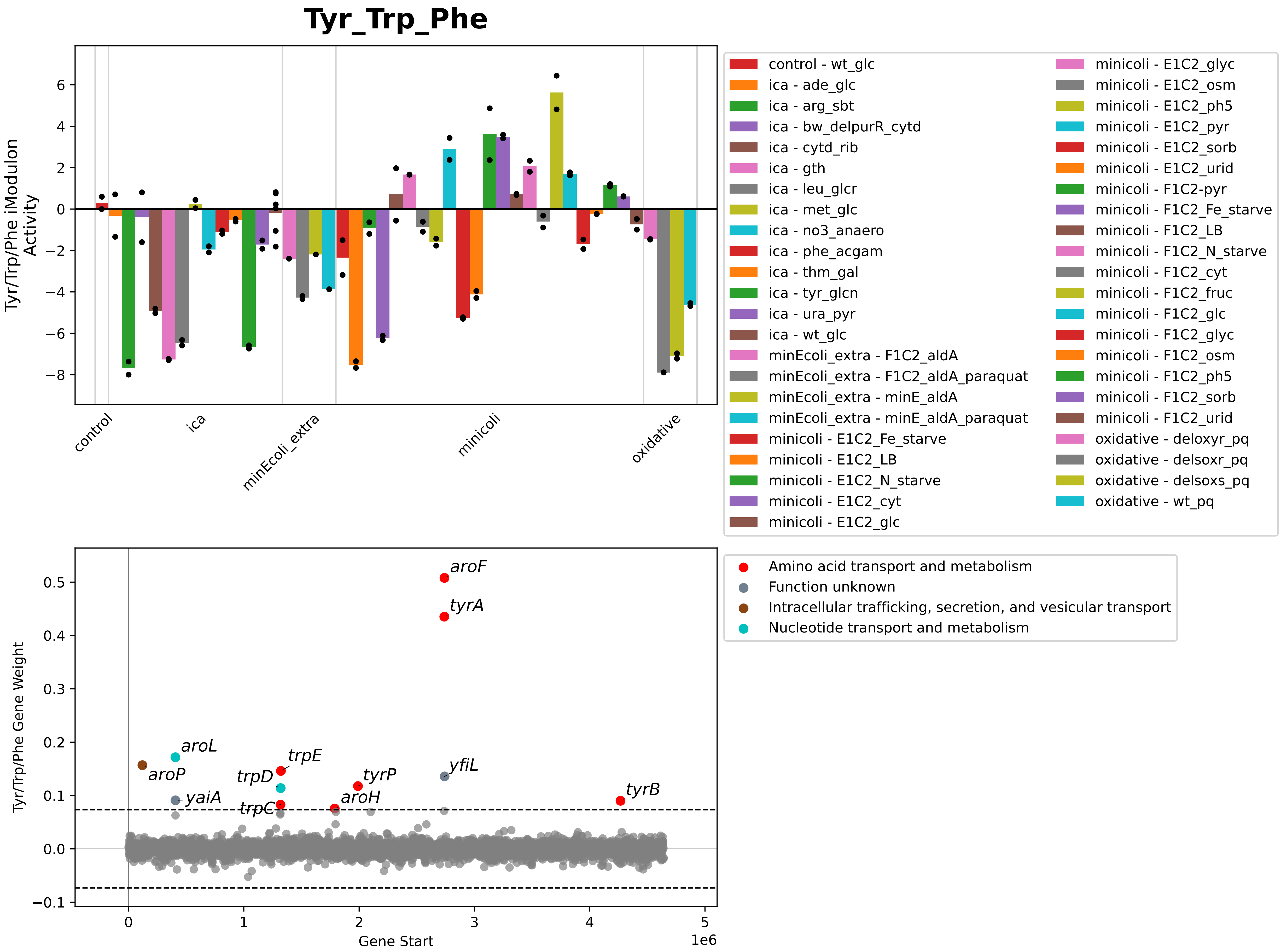
Tyr_Trp_Phe
Tyr/Trp/Phe iModulon Activity
control
ica
minEcoli_extra
minicoli
oxidative
control - wt_glc
ica - ade_glc
ica - arg_sbt
ica - bw_delpurR_cytd
ica - cytd_rib
ica - gth
ica - leu_glcr
ica - met_glc
ica - no3_anaero
ica - phe_acgam
ica - thm_gal
ica - tyr_glcn
ica - ura_pyr
ica - wt_glc
minEcoli_extra - F1C2_aldA
minEcoli_extra - F1C2_aldA_paraquat
minEcoli_extra - minE_aldA
minEcoli_extra - minE_aldA_paraquat
minicoli - E1C2_Fe_starve
minicoli - E1C2_LB
minicoli - E1C2_N_starve
minicoli - E1C2_cyt
minicoli - E1C2_glc
minicoli - E1C2_glyc
minicoli - E1C2_osm
minicoli - E1C2_ph5
minicoli - E1C2_pyr
minicoli - E1C2_sorb
minicoli - E1C2_urid
minicoli - F1C2-pyr
minicoli - F1C2_Fe_starve
minicoli - F1C2_LB
minicoli - F1C2_N_starve
minicoli - F1C2_cyt
minicoli - F1C2_fruc
minicoli - F1C2_glc
minicoli - F1C2_glyc
minicoli - F1C2_osm
minicoli - F1C2_ph5
minicoli - F1C2_sorb
minicoli - F1C2_urid
oxidative - deloxyr_pq
oxidative - delsoxr_pq
oxidative - delsoxs_pq
oxidative - wt_pq
Tyr/Trp/Phe Gene Weight
Gene Start
1e6
aroF
tyrA
aroL
aroP
yaiA
trpE
trpD
trpC
tyrP
aroH
yfiL
tyrB
Amino acid transport and metabolism
Function unknown
Intracellular trafficking, secretion, and vesicular transport
Nucleotide transport and metabolism

GadX

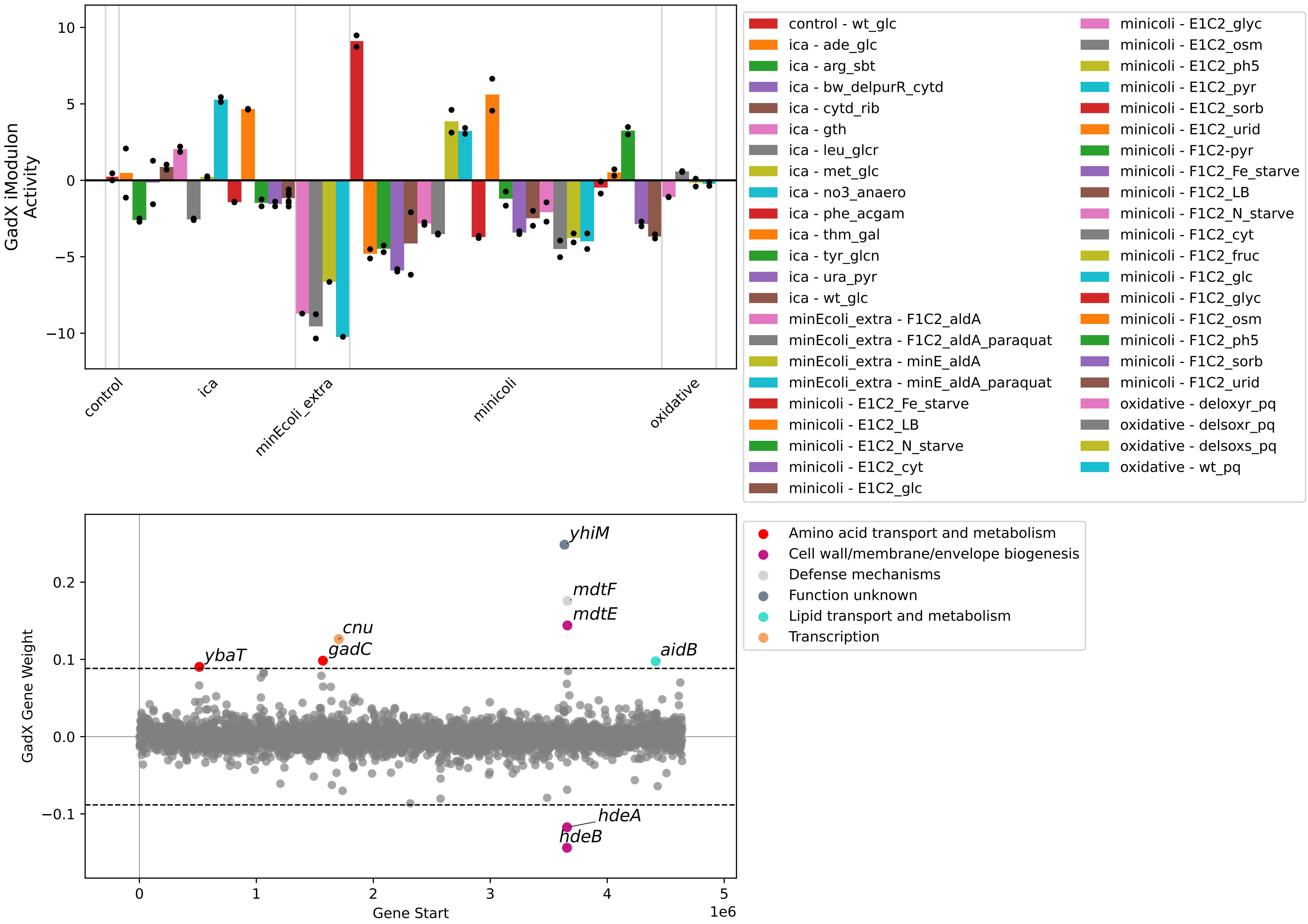

GadX iModulon Activity
control
ica
minEcoli_extra
minicoli
oxidative
control - wt_glc
ica - ade_glc
ica - arg_sbt
ica - bw_delpurR_cytd
ica - cytd_rib
ica - gth
ica - leu_glcr
ica - met_glc
ica - no3_anaero
ica - phe_acgam
ica - thm_gal
ica - tyr_glcn
ica - ura_pyr
ica - wt_glc
minEcoli_extra - F1C2_aldA
minEcoli_extra - F1C2_aldA_paraquat
minEcoli_extra - minE_aldA
minEcoli_extra - minE_aldA_paraquat
minicoli - E1C2_Fe_starve
minicoli - E1C2_LB
minicoli - E1C2_N_starve
minicoli - E1C2_cyt
minicoli - E1C2_glc
minicoli - E1C2_glyc
minicoli - E1C2_osm
minicoli - E1C2_ph5
minicoli - E1C2_pyr
minicoli - E1C2_sorb
minicoli - E1C2_urid
minicoli - F1C2-pyr
minicoli - F1C2_Fe_starve
minicoli - F1C2_LB
minicoli - F1C2_N_starve
minicoli - F1C2_cyt
minicoli - F1C2_fruc
minicoli - F1C2_glc
minicoli - F1C2_glyc
minicoli - F1C2_osm
minicoli - F1C2_ph5
minicoli - F1C2_sorb
minicoli - F1C2_urid
oxidative - deloxyr_pq
oxidative - delsoxr_pq
oxidative - delsoxs_pq
oxidative - wt_pq
GadX Gene Weight
Gene Start
1e6
yhiM
mdtF
mdtE
cnu
gadC
ybaT
aidB
hdeA
hdeB
Amino acid transport and metabolism
Cell wall/membrane/envelope biogenesis
Defense mechanisms
Function unknown
Lipid transport and metabolism
Transcription

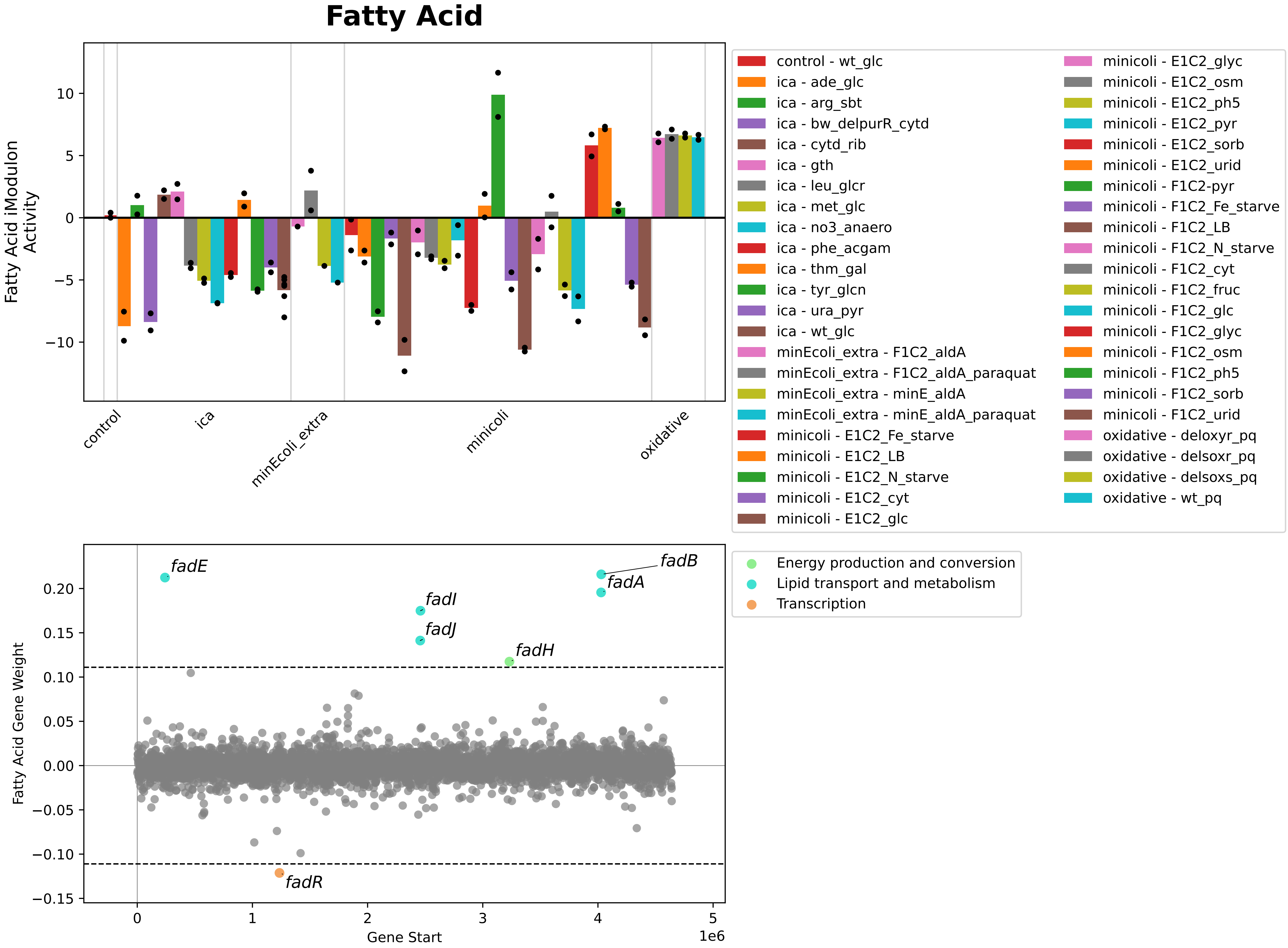

Fatty Acid
Fatty Acid iModulon Activity
control
ica
minEcoli_extra
minicoli
oxidative
control - wt_glc
ica - ade_glc
ica - arg_sbt
ica - bw_delpurR_cytd
ica - cytd_rib
ica - gth
ica - leu_glcr
ica - met_glc
ica - no3_anaero
ica - phe_acgam
ica - thm_gal
ica - tyr_glcn
ica - ura_pyr
ica - wt_glc
minEcoli_extra - F1C2_aldA
minEcoli_extra - F1C2_aldA_paraquat
minEcoli_extra - minE_aldA
minEcoli_extra - minE_aldA_paraquat
minicoli - E1C2_Fe_starve
minicoli - E1C2_LB
minicoli - E1C2_N_starve
minicoli - E1C2_cyt
minicoli - E1C2_glc
minicoli - E1C2_glyc
minicoli - E1C2_osm
minicoli - E1C2_ph5
minicoli - E1C2_pyr
minicoli - E1C2_sorb
minicoli - E1C2_urid
minicoli - F1C2-pyr
minicoli - F1C2_Fe_starve
minicoli - F1C2_LB
minicoli - F1C2_N_starve
minicoli - F1C2_cyt
minicoli - F1C2_fruc
minicoli - F1C2_glc
minicoli - F1C2_glyc
minicoli - F1C2_osm
minicoli - F1C2_ph5
minicoli - F1C2_sorb
minicoli - F1C2_urid
oxidative - deloxyr_pq
oxidative - delsoxr_pq
oxidative - delsoxs_pq
oxidative - wt_pq
Fatty Acid Gene Weight
Gene Start
1e6
fadE
fadI
fadJ
fadH
fadB
fadA
fadR
Energy production and conversion
Lipid transport and metabolism
Transcription

DhaR

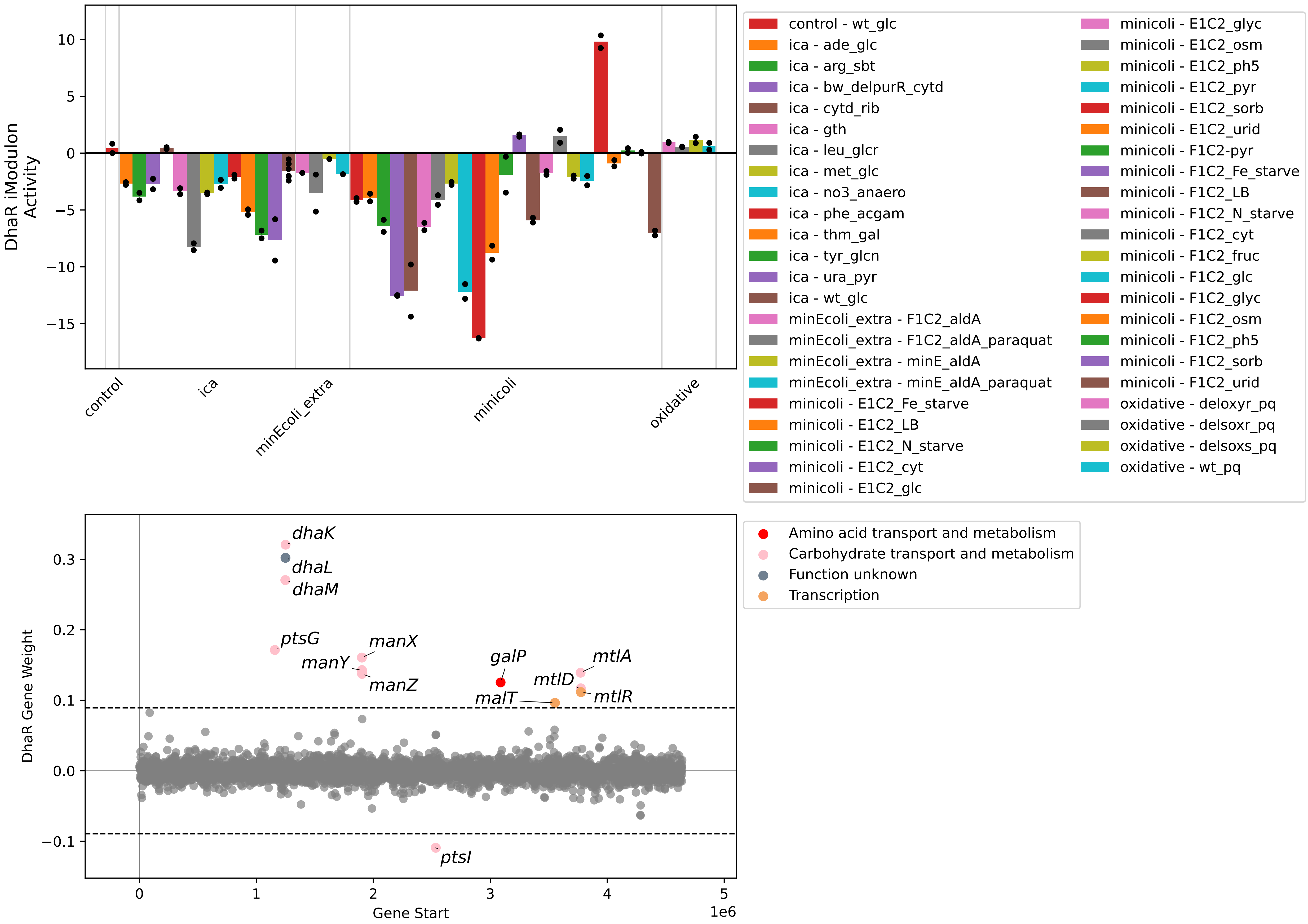
DhaR iModulon Activity
control
ica
minEcoli_extra
minicoli
oxidative
control - wt_glc
ica - ade_glc
ica - arg_sbt
ica - bw_delpurR_cytd
ica - cytd_rib
ica - gth
ica - leu_glcr
ica - met_glc
ica - no3_anaero
ica - phe_acgam
ica - thm_gal
ica - tyr_glcn
ica - ura_pyr
ica - wt_glc
minEcoli_extra - F1C2_aldA
minEcoli_extra - F1C2_aldA_paraquat
minEcoli_extra - minE_aldA
minEcoli_extra - minE_aldA_paraquat
minicoli - E1C2_Fe_starve
minicoli - E1C2_LB
minicoli - E1C2_N_starve
minicoli - E1C2_cyt
minicoli - E1C2_glc
minicoli - E1C2_glyc
minicoli - E1C2_osm
minicoli - E1C2_ph5
minicoli - E1C2_pyr
minicoli - E1C2_sorb
minicoli - E1C2_urid
minicoli - F1C2-pyr
minicoli - F1C2_Fe_starve
minicoli - F1C2_LB
minicoli - F1C2_N_starve
minicoli - F1C2_cyt
minicoli - F1C2_fruc
minicoli - F1C2_glc
minicoli - F1C2_glyc
minicoli - F1C2_osm
minicoli - F1C2_ph5
minicoli - F1C2_sorb
minicoli - F1C2_urid
oxidative - deloxyr_pq
oxidative - delsoxr_pq
oxidative - delsoxs_pq
oxidative - wt_pq
DhaR Gene Weight
Gene Start
1e6
dhaK
dhaL
dhaM
ptsG
manX
manY
manZ
galP
mtlA
mtlD
malT
mtlR
ptsI
Amino acid transport and metabolism
Carbohydrate transport and metabolism
Function unknown
Transcription

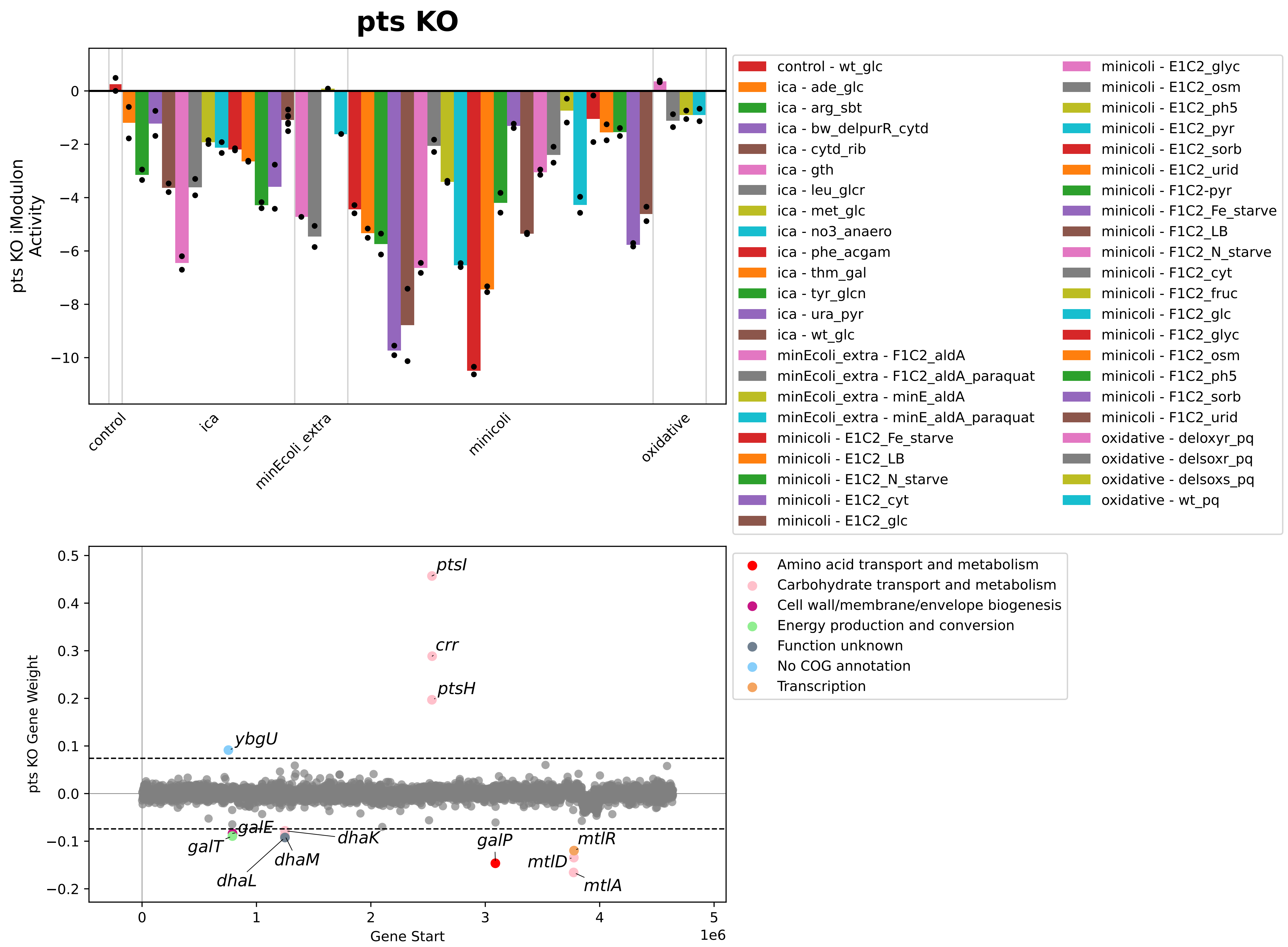
pts KO
pts KO iModulon Activity
control
ica
minEcoli_extra
minicoli
oxidative
control - wt_glc
ica - ade_glc
ica - arg_sbt
ica - bw_delpurR_cytd
ica - cytd_rib
ica - gth
ica - leu_glcr
ica - met_glc
ica - no3_anaero
ica - phe_acgam
ica - thm_gal
ica - tyr_glcn
ica - ura_pyr
ica - wt_glc
minEcoli_extra - F1C2_aldA
minEcoli_extra - F1C2_aldA_paraquat
minEcoli_extra - minE_aldA
minEcoli_extra - minE_aldA_paraquat
minicoli - E1C2_Fe_starve
minicoli - E1C2_LB
minicoli - E1C2_N_starve
minicoli - E1C2_cyt
minicoli - E1C2_glc
minicoli - E1C2_glyc
minicoli - E1C2_osm
minicoli - E1C2_ph5
minicoli - E1C2_pyr
minicoli - E1C2_sorb
minicoli - E1C2_urid
minicoli - F1C2-pyr
minicoli - F1C2_Fe_starve
minicoli - F1C2_LB
minicoli - F1C2_N_starve
minicoli - F1C2_cyt
minicoli - F1C2_fruc
minicoli - F1C2_glc
minicoli - F1C2_glyc
minicoli - F1C2_osm
minicoli - F1C2_ph5
minicoli - F1C2_sorb
minicoli - F1C2_urid
oxidative - deloxyr_pq
oxidative - delsoxr_pq
oxidative - delsoxs_pq
oxidative - wt_pq
pts KO Gene Weight
Gene Start
1e6
ptsI
crr
ptsH
ybgU
galE
galT
dhaK
dhaM
dhaL
galP
mtlR
mtlD
mtlA
Amino acid transport and metabolism
Carbohydrate transport and metabolism
Cell wall/membrane/envelope biogenesis
Energy production and conversion
Function unknown
No COG annotation
Transcription

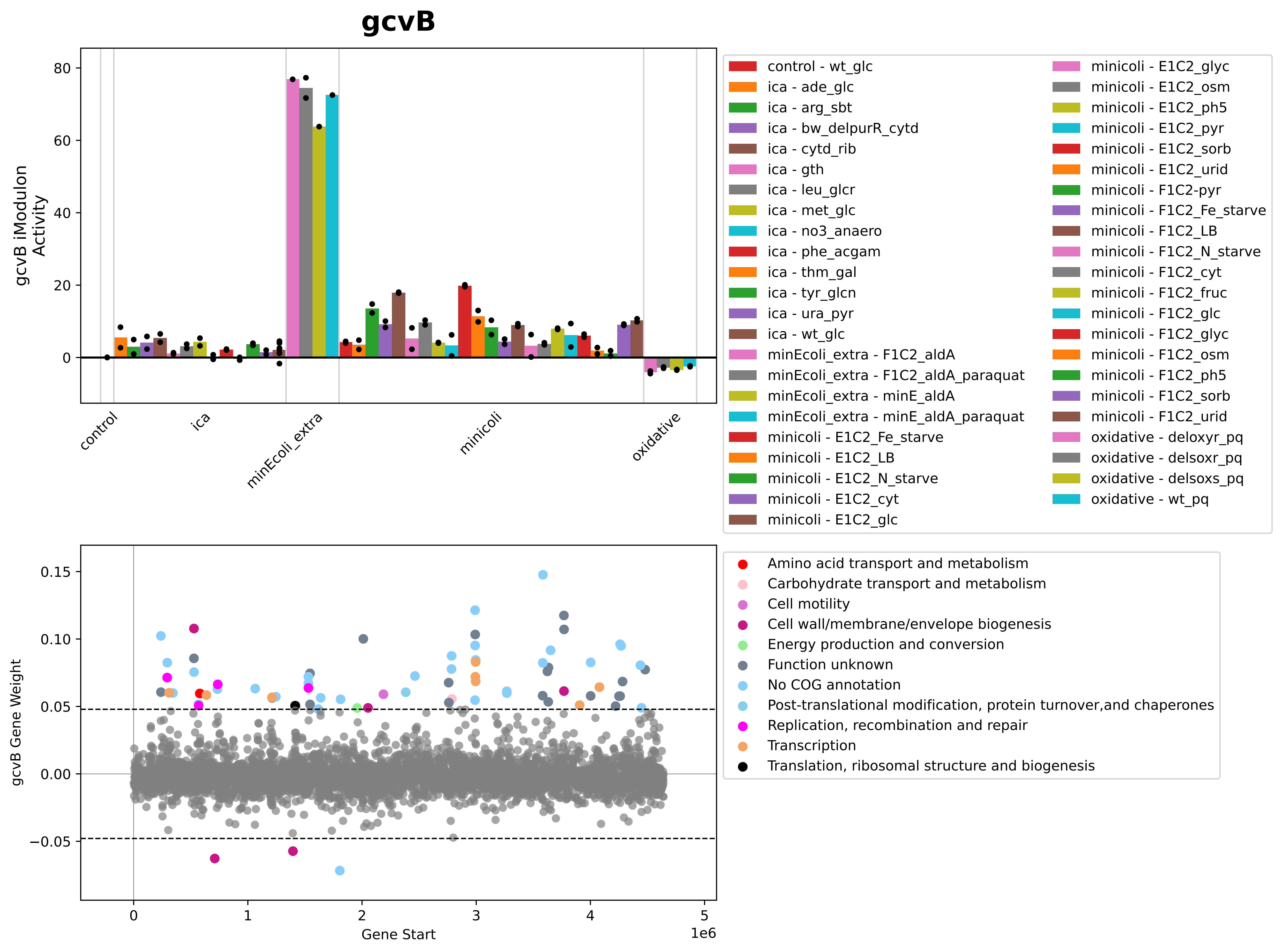
gcvB
gcvB iModulon Activity
control
ica
minEcoli_extra
minicoli
oxidative
control - wt_glc
ica - ade_glc
ica - arg_sbt
ica - bw_delpurR_cytd
ica - cytd_rib
ica - gth
ica - leu_glcr
ica - met_glc
ica - no3_anaero
ica - phe_acgam
ica - thm_gal
ica - tyr_glcn
ica - ura_pyr
ica - wt_glc
minEcoli_extra - F1C2_aldA
minEcoli_extra - F1C2_aldA_paraquat
minEcoli_extra - minE_aldA
minEcoli_extra - minE_aldA_paraquat
minicoli - E1C2_Fe_starve
minicoli - E1C2_LB
minicoli - E1C2_N_starve
minicoli - E1C2_cyt
minicoli - E1C2_glc
minicoli - E1C2_glyc
minicoli - E1C2_osm
minicoli - E1C2_ph5
minicoli - E1C2_pyr
minicoli - E1C2_sorb
minicoli - E1C2_urid
minicoli - F1C2-pyr
minicoli - F1C2_Fe_starve
minicoli - F1C2_LB
minicoli - F1C2_N_starve
minicoli - F1C2_cyt
minicoli - F1C2_fruc
minicoli - F1C2_glc
minicoli - F1C2_glyc
minicoli - F1C2_osm
minicoli - F1C2_ph5
minicoli - F1C2_sorb
minicoli - F1C2_urid
oxidative - deloxyr_pq
oxidative - delsoxr_pq
oxidative - delsoxs_pq
oxidative - wt_pq
gcvB Gene Weight
Gene Start
1e6
Amino acid transport and metabolism
Carbohydrate transport and metabolism
Cell motility
Cell wall/membrane/envelope biogenesis
Energy production and conversion
Function unknown
No COG annotation
Post-translational modification, protein turnover,and chaperones
Replication, recombination and repair
Transcription
Translation, ribosomal structure and biogenesis

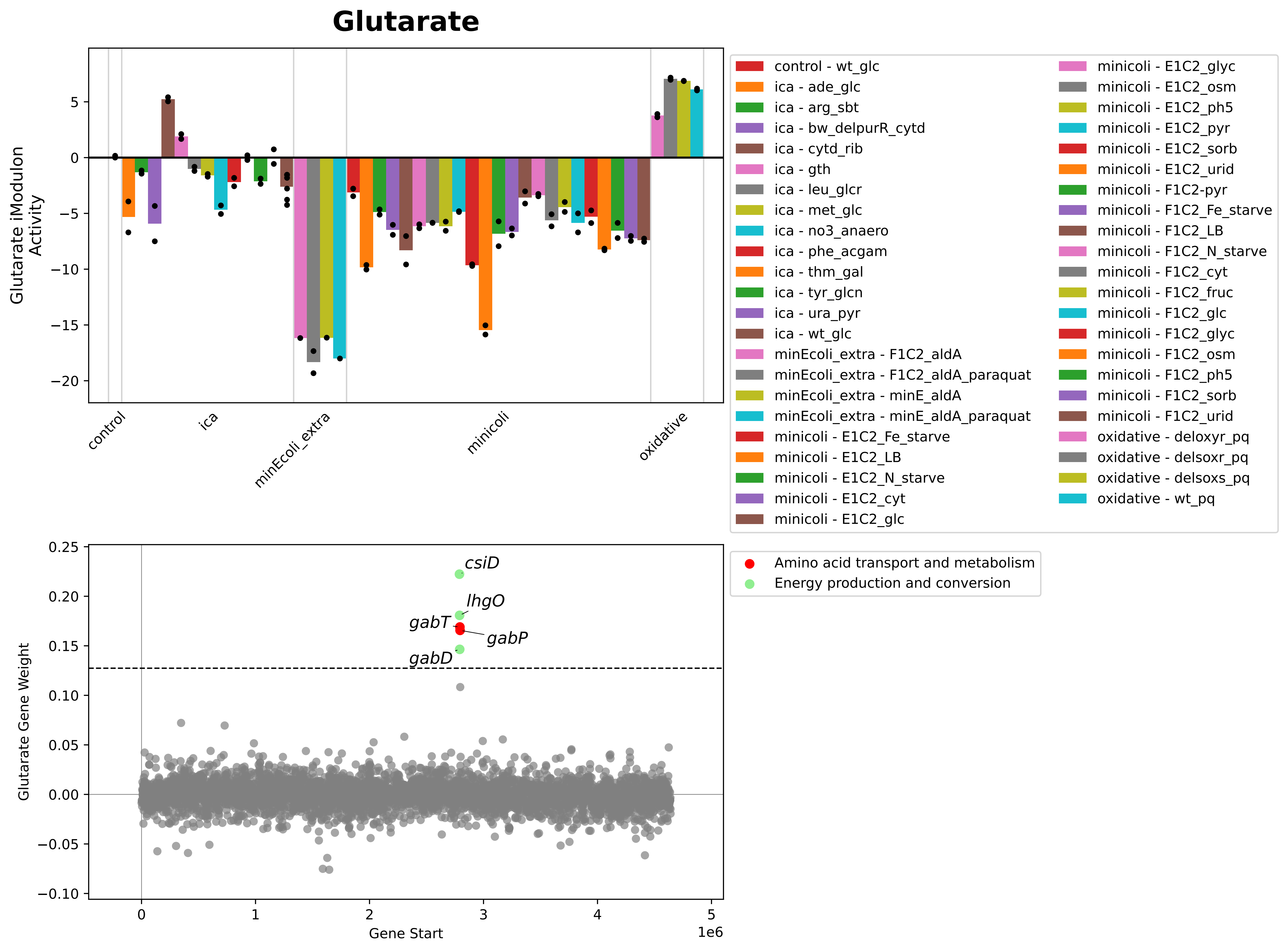

Glutarate
Glutarate iModulon Activity
control
ica
minEcoli_extra
minicoli
oxidative
control - wt_glc
ica - ade_glc
ica - arg_sbt
ica - bw_delpurR_cytd
ica - cytd_rib
ica - gth
ica - leu_glcr
ica - met_glc
ica - no3_anaero
ica - phe_acgam
ica - thm_gal
ica - tyr_glcn
ica - ura_pyr
ica - wt_glc
minEcoli_extra - F1C2_aldA
minEcoli_extra - F1C2_aldA_paraquat
minEcoli_extra - minE_aldA
minEcoli_extra - minE_aldA_paraquat
minicoli - E1C2_Fe_starve
minicoli - E1C2_LB
minicoli - E1C2_N_starve
minicoli - E1C2_cyt
minicoli - E1C2_glc
minicoli - E1C2_glyc
minicoli - E1C2_osm
minicoli - E1C2_ph5
minicoli - E1C2_pyr
minicoli - E1C2_sorb
minicoli - E1C2_urid
minicoli - F1C2-pyr
minicoli - F1C2_Fe_starve
minicoli - F1C2_LB
minicoli - F1C2_N_starve
minicoli - F1C2_cyt
minicoli - F1C2_fruc
minicoli - F1C2_glc
minicoli - F1C2_glyc
minicoli - F1C2_osm
minicoli - F1C2_ph5
minicoli - F1C2_sorb
minicoli - F1C2_urid
oxidative - deloxyr_pq
oxidative - delsoxr_pq
oxidative - delsoxs_pq
oxidative - wt_pq
csiD
lhgO
gabT
gabP
gabD
Amino acid transport and metabolism
Energy production and conversion
Glutarate Gene Weight
Gene Start
1e6

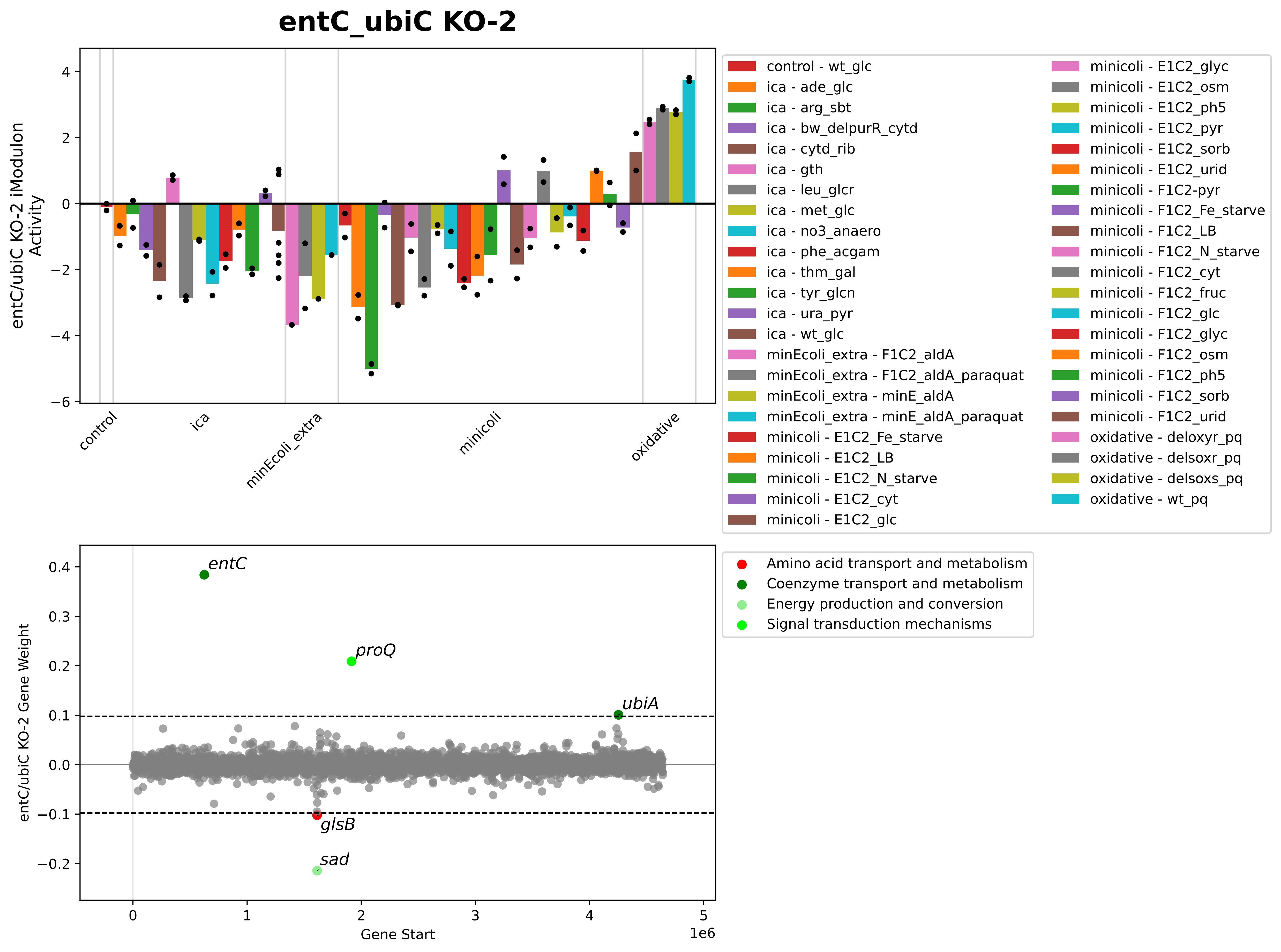

entC_ubiC KO-2
entC/ubiC KO-2 iModulon Activity
control
ica
minEcoli_extra
minicoli
oxidative
control - wt_glc
ica - ade_glc
ica - arg_sbt
ica - bw_delpurR_cytd
ica - cytd_rib
ica - gth
ica - leu_glcr
ica - met_glc
ica - no3_anaero
ica - phe_acgam
ica - thm_gal
ica - tyr_glcn
ica - ura_pyr
ica - wt_glc
minEcoli_extra - F1C2_aldA
minEcoli_extra - F1C2_aldA_paraquat
minEcoli_extra - minE_aldA
minEcoli_extra - minE_aldA_paraquat
minicoli - E1C2_Fe_starve
minicoli - E1C2_LB
minicoli - E1C2_N_starve
minicoli - E1C2_cyt
minicoli - E1C2_glc
minicoli - E1C2_glyc
minicoli - E1C2_osm
minicoli - E1C2_ph5
minicoli - E1C2_pyr
minicoli - E1C2_sorb
minicoli - E1C2_urid
minicoli - F1C2-pyr
minicoli - F1C2_Fe_starve
minicoli - F1C2_LB
minicoli - F1C2_N_starve
minicoli - F1C2_cyt
minicoli - F1C2_fruc
minicoli - F1C2_glc
minicoli - F1C2_glyc
minicoli - F1C2_osm
minicoli - F1C2_ph5
minicoli - F1C2_sorb
minicoli - F1C2_urid
oxidative - deloxyr_pq
oxidative - delsoxr_pq
oxidative - delsoxs_pq
oxidative - wt_pq
entC/ubiC KO-2 Gene Weight
entC
proQ
ubiA
glsB
sad
Gene Start
1e6
Amino acid transport and metabolism
Coenzyme transport and metabolism
Energy production and conversion
Signal transduction mechanisms

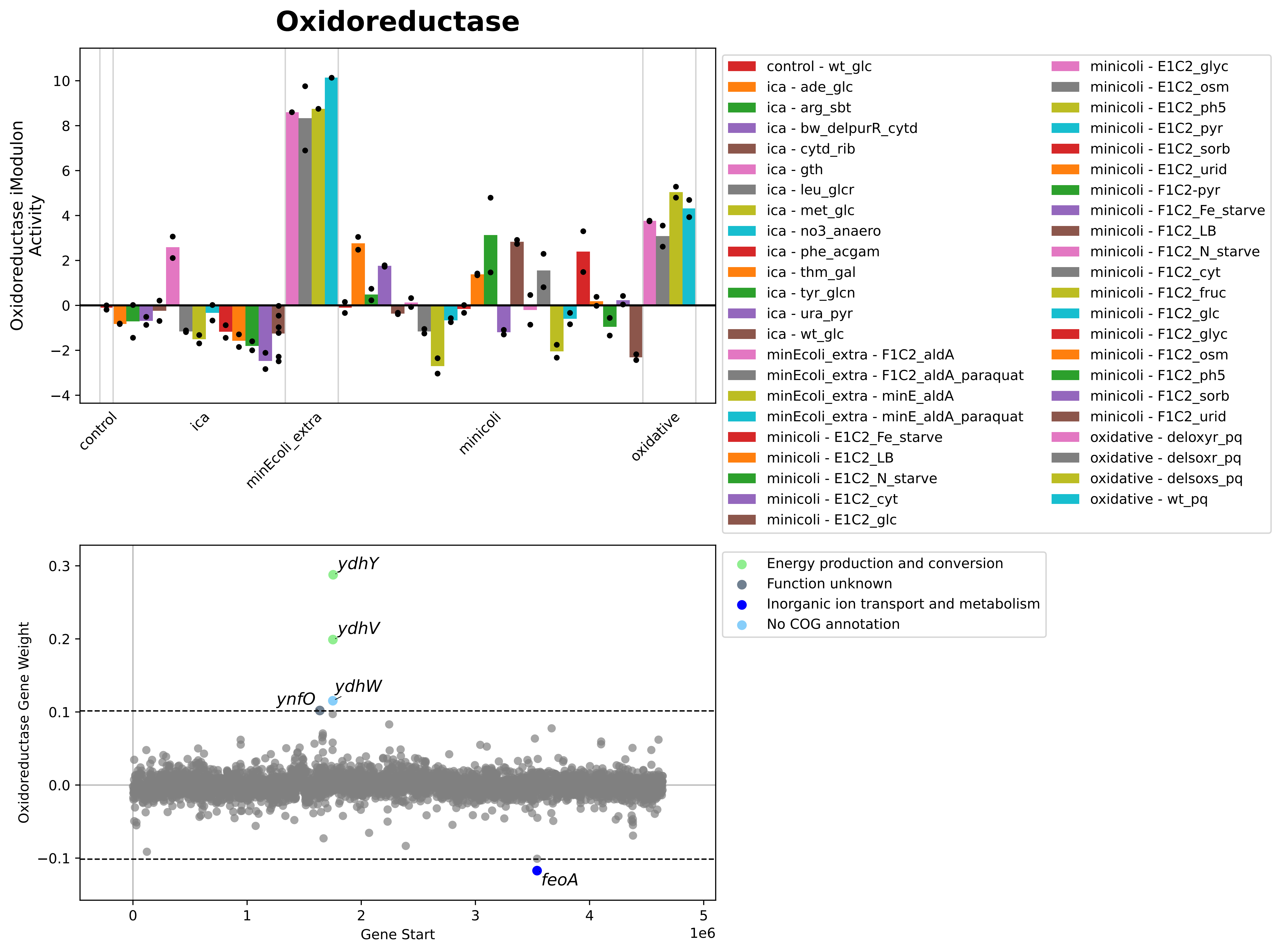
Oxidoreductase
Oxidoreductase iModulon Activity
control
ica
minEcoli_extra
minicoli
oxidative
control - wt_glc
ica - ade_glc
ica - arg_sbt
ica - bw_delpurR_cytd
ica - cytd_rib
ica - gth
ica - leu_glcr
ica - met_glc
ica - no3_anaero
ica - phe_acgam
ica - thm_gal
ica - tyr_glcn
ica - ura_pyr
ica - wt_glc
minEcoli_extra - F1C2_aldA
minEcoli_extra - F1C2_aldA_paraquat
minEcoli_extra - minE_aldA
minEcoli_extra - minE_aldA_paraquat
minicoli - E1C2_Fe_starve
minicoli - E1C2_LB
minicoli - E1C2_N_starve
minicoli - E1C2_cyt
minicoli - E1C2_glc
minicoli - E1C2_glyc
minicoli - E1C2_osm
minicoli - E1C2_ph5
minicoli - E1C2_pyr
minicoli - E1C2_sorb
minicoli - E1C2_urid
minicoli - F1C2-pyr
minicoli - F1C2_Fe_starve
minicoli - F1C2_LB
minicoli - F1C2_N_starve
minicoli - F1C2_cyt
minicoli - F1C2_fruc
minicoli - F1C2_glc
minicoli - F1C2_glyc
minicoli - F1C2_osm
minicoli - F1C2_ph5
minicoli - F1C2_sorb
minicoli - F1C2_urid
oxidative - deloxyr_pq
oxidative - delsoxr_pq
oxidative - delsoxs_pq
oxidative - wt_pq
Oxidoreductase Gene Weight
Gene Start
1e6
ydhY
ydhV
ydhW
ynfO
feoA
Energy production and conversion
Function unknown
Inorganic ion transport and metabolism
No COG annotation

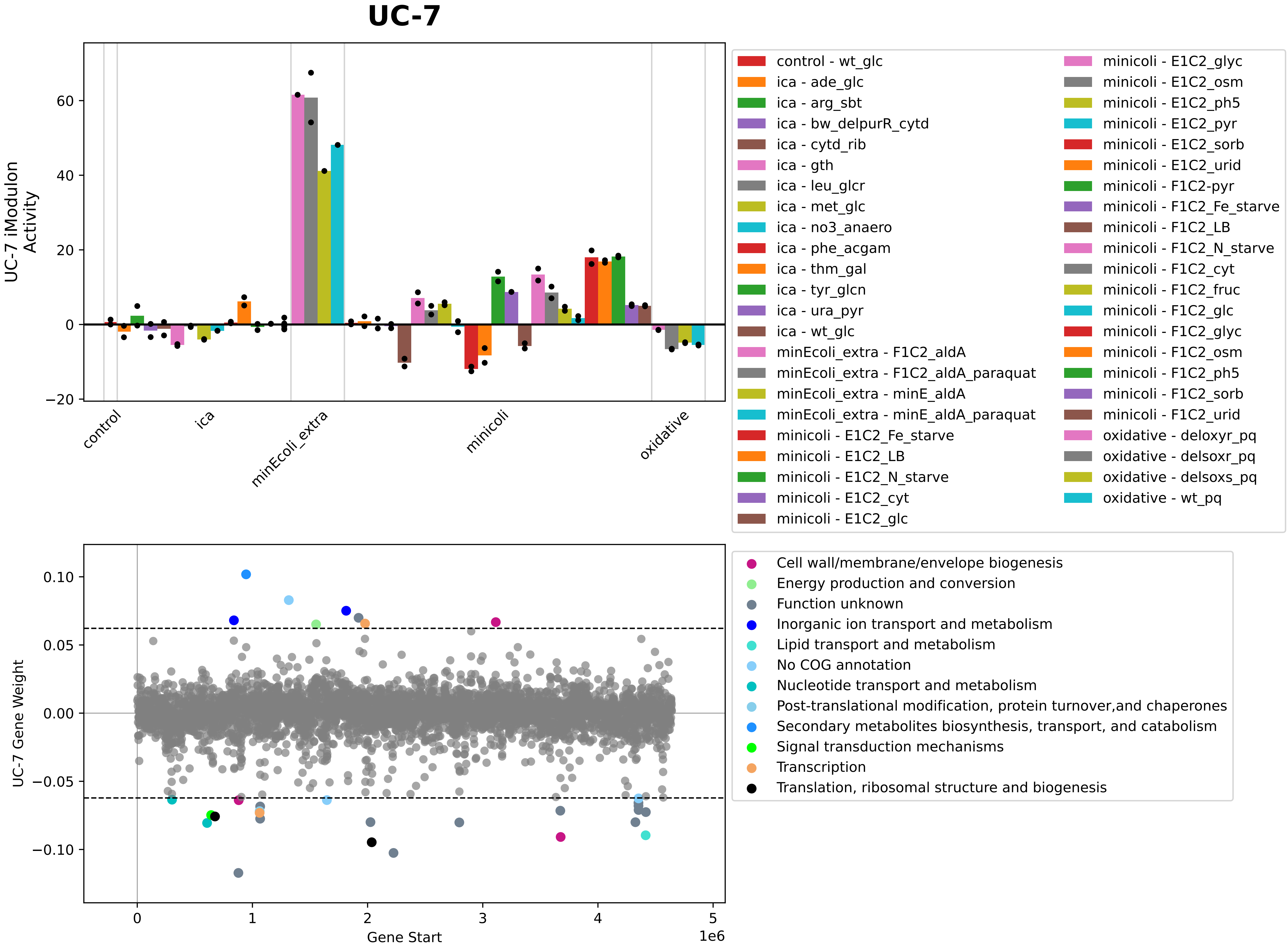

UC-7
UC-7 iModulon Activity
60
40
20
0
−20
control
ica
minEcoli_extra
minicoli
oxidative
control - wt_glc
ica - ade_glc
ica - arg_sbt
ica - bw_delpurR_cytd
ica - cytd_rib
ica - gth
ica - leu_glcr
ica - met_glc
ica - no3_anaero
ica - phe_acgam
ica - thm_gal
ica - tyr_glcn
ica - ura_pyr
ica - wt_glc
minEcoli_extra - F1C2_aldA
minEcoli_extra - F1C2_aldA_paraquat
minEcoli_extra - minE_aldA
minEcoli_extra - minE_aldA_paraquat
minicoli - E1C2_Fe_starve
minicoli - E1C2_LB
minicoli - E1C2_N_starve
minicoli - E1C2_cyt
minicoli - E1C2_glc
minicoli - E1C2_glyc
minicoli - E1C2_osm
minicoli - E1C2_ph5
minicoli - E1C2_pyr
minicoli - E1C2_sorb
minicoli - E1C2_urid
minicoli - F1C2-pyr
minicoli - F1C2_Fe_starve
minicoli - F1C2_LB
minicoli - F1C2_N_starve
minicoli - F1C2_cyt
minicoli - F1C2_fruc
minicoli - F1C2_glc
minicoli - F1C2_glyc
minicoli - F1C2_osm
minicoli - F1C2_ph5
minicoli - F1C2_sorb
minicoli - F1C2_urid
oxidative - deloxyr_pq
oxidative - delsoxr_pq
oxidative - delsoxs_pq
oxidative - wt_pq
UC-7 Gene Weight
0.10
0.05
0.00
−0.05
−0.10
0
1
2
3
4
5
1e6
Gene Start
Cell wall/membrane/envelope biogenesis
Energy production and conversion
Function unknown
Inorganic ion transport and metabolism
Lipid transport and metabolism
No COG annotation
Nucleotide transport and metabolism
Post-translational modification, protein turnover,and chaperones
Secondary metabolites biosynthesis, transport, and catabolism
Signal transduction mechanisms
Transcription
Translation, ribosomal structure and biogenesis

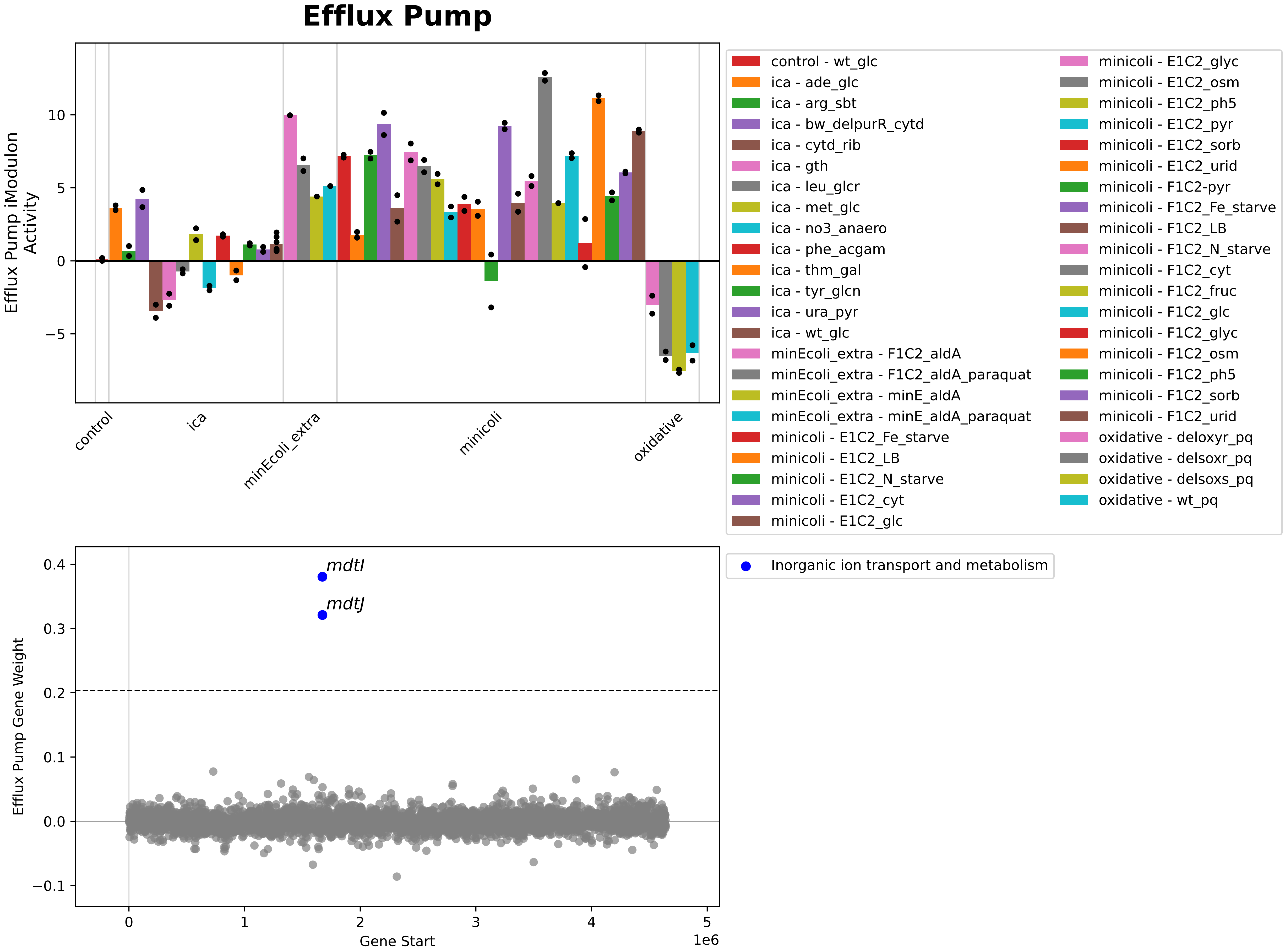
Efflux Pump
Efflux Pump iModulon Activity
10
5
0
−5
control
ica
minEcoli_extra
minicoli
oxidative
control - wt_glc
ica - ade_glc
ica - arg_sbt
ica - bw_delpurR_cytd
ica - cytd_rib
ica - gth
ica - leu_glcr
ica - met_glc
ica - no3_anaero
ica - phe_acgam
ica - thm_gal
ica - tyr_glcn
ica - ura_pyr
ica - wt_glc
minEcoli_extra - F1C2_aldA
minEcoli_extra - F1C2_aldA_paraquat
minEcoli_extra - minE_aldA
minEcoli_extra - minE_aldA_paraquat
minicoli - E1C2_Fe_starve
minicoli - E1C2_LB
minicoli - E1C2_N_starve
minicoli - E1C2_cyt
minicoli - E1C2_glc
minicoli - E1C2_glyc
minicoli - E1C2_osm
minicoli - E1C2_ph5
minicoli - E1C2_pyr
minicoli - E1C2_sorb
minicoli - E1C2_urid
minicoli - F1C2-pyr
minicoli - F1C2_Fe_starve
minicoli - F1C2_LB
minicoli - F1C2_N_starve
minicoli - F1C2_cyt
minicoli - F1C2_fruc
minicoli - F1C2_glc
minicoli - F1C2_glyc
minicoli - F1C2_osm
minicoli - F1C2_ph5
minicoli - F1C2_sorb
minicoli - F1C2_urid
oxidative - deloxyr_pq
oxidative - delsoxr_pq
oxidative - delsoxs_pq
oxidative - wt_pq
mdtI
mdtJ
Efflux Pump Gene Weight
0.4
0.3
0.2
0.1
0.0
−0.1
0
1
2
3
4
5
Gene Start
1e6
Inorganic ion transport and metabolism

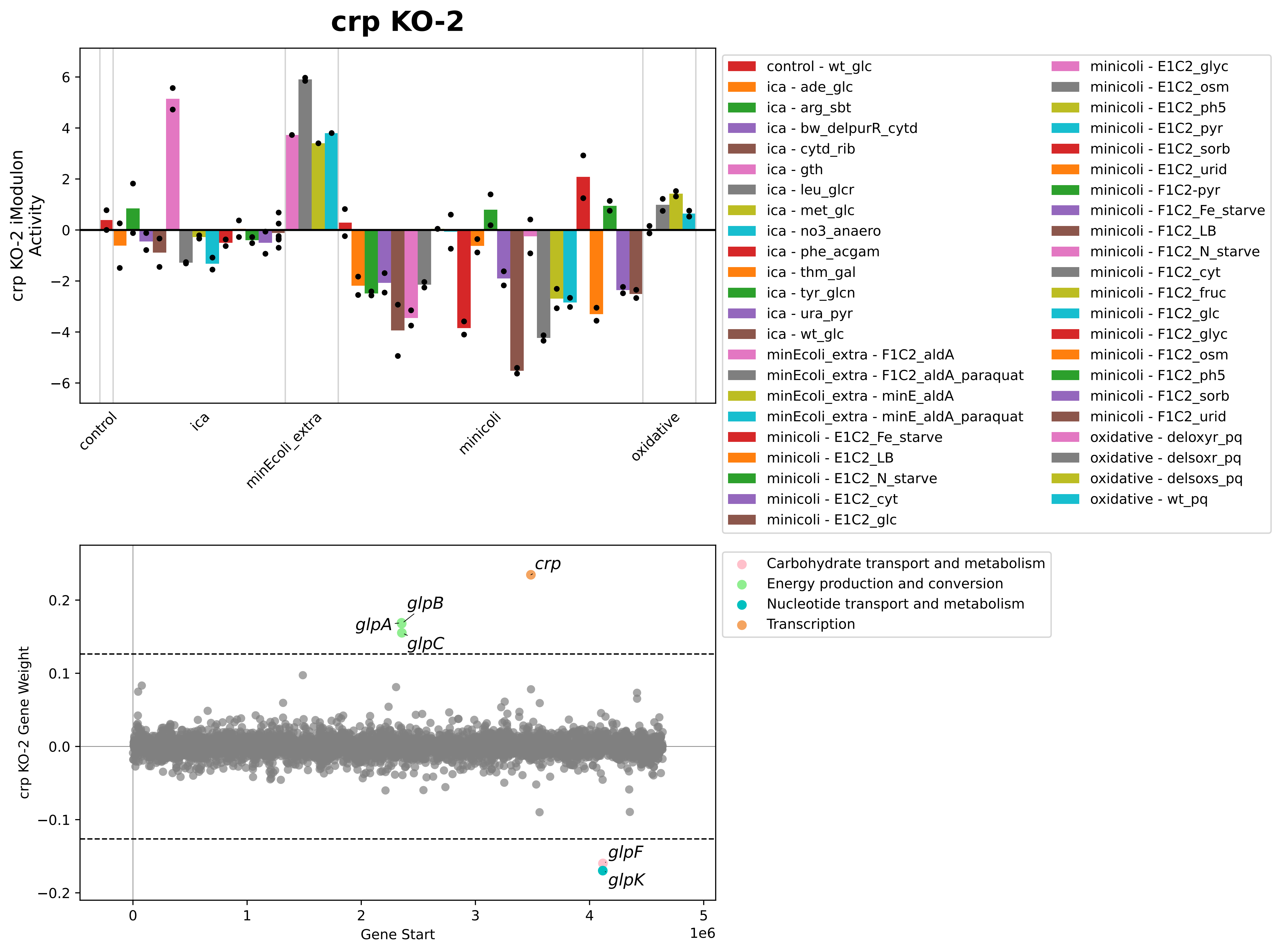
crp KO-2
crp KO-2 iModulon Activity
control
ica
minEcoli_extra
minicoli
oxidative
control - wt_glc
ica - ade_glc
ica - arg_sbt
ica - bw_delpurR_cytd
ica - cytd_rib
ica - gth
ica - leu_glcr
ica - met_glc
ica - no3_anaero
ica - phe_acgam
ica - thm_gal
ica - tyr_glcn
ica - ura_pyr
ica - wt_glc
minEcoli_extra - F1C2_aldA
minEcoli_extra - F1C2_aldA_paraquat
minEcoli_extra - minE_aldA
minEcoli_extra - minE_aldA_paraquat
minicoli - E1C2_Fe_starve
minicoli - E1C2_LB
minicoli - E1C2_N_starve
minicoli - E1C2_cyt
minicoli - E1C2_glc
minicoli - E1C2_glyc
minicoli - E1C2_osm
minicoli - E1C2_ph5
minicoli - E1C2_pyr
minicoli - E1C2_sorb
minicoli - E1C2_urid
minicoli - F1C2-pyr
minicoli - F1C2_Fe_starve
minicoli - F1C2_LB
minicoli - F1C2_N_starve
minicoli - F1C2_cyt
minicoli - F1C2_fruc
minicoli - F1C2_glc
minicoli - F1C2_glyc
minicoli - F1C2_osm
minicoli - F1C2_ph5
minicoli - F1C2_sorb
minicoli - F1C2_urid
oxidative - deloxyr_pq
oxidative - delsoxr_pq
oxidative - delsoxs_pq
oxidative - wt_pq
crp
glpB
glpA
glpC
glpF
glpK
crp KO-2 Gene Weight
Gene Start
1e6
Carbohydrate transport and metabolism
Energy production and conversion
Nucleotide transport and metabolism
Transcription

cyoB_ndh_nuoB KO-2

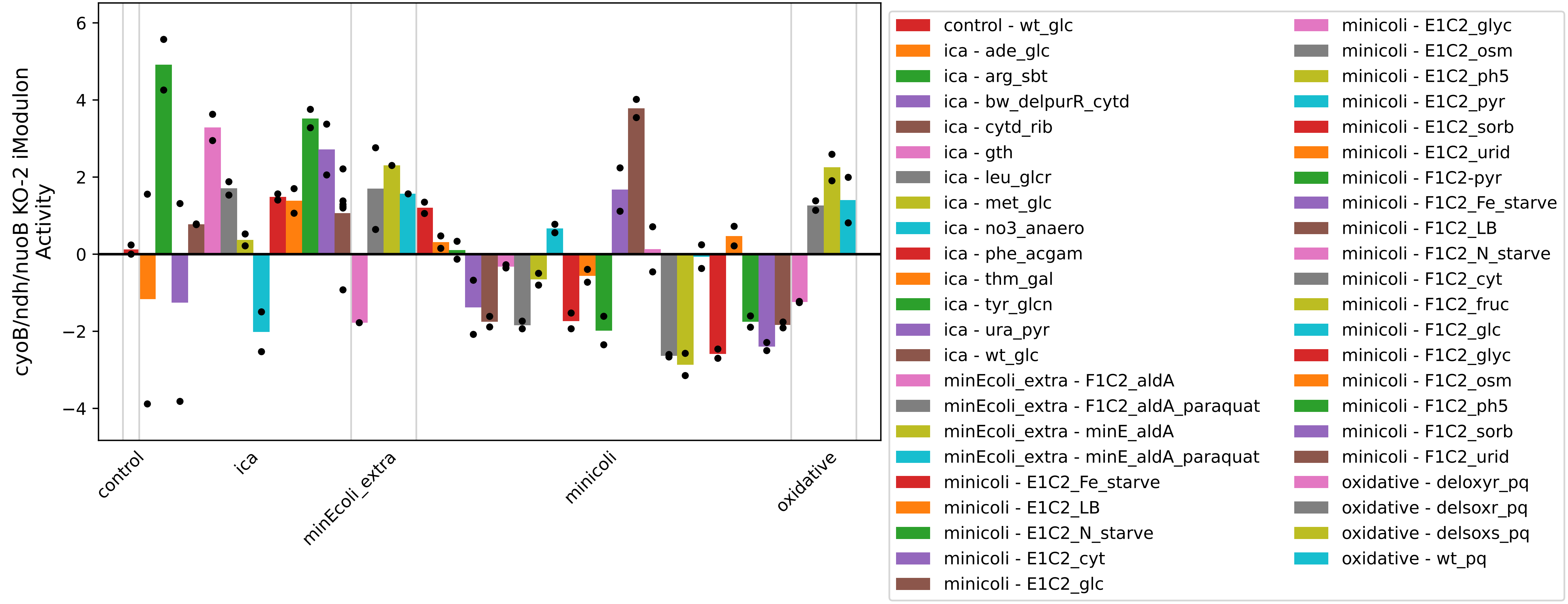

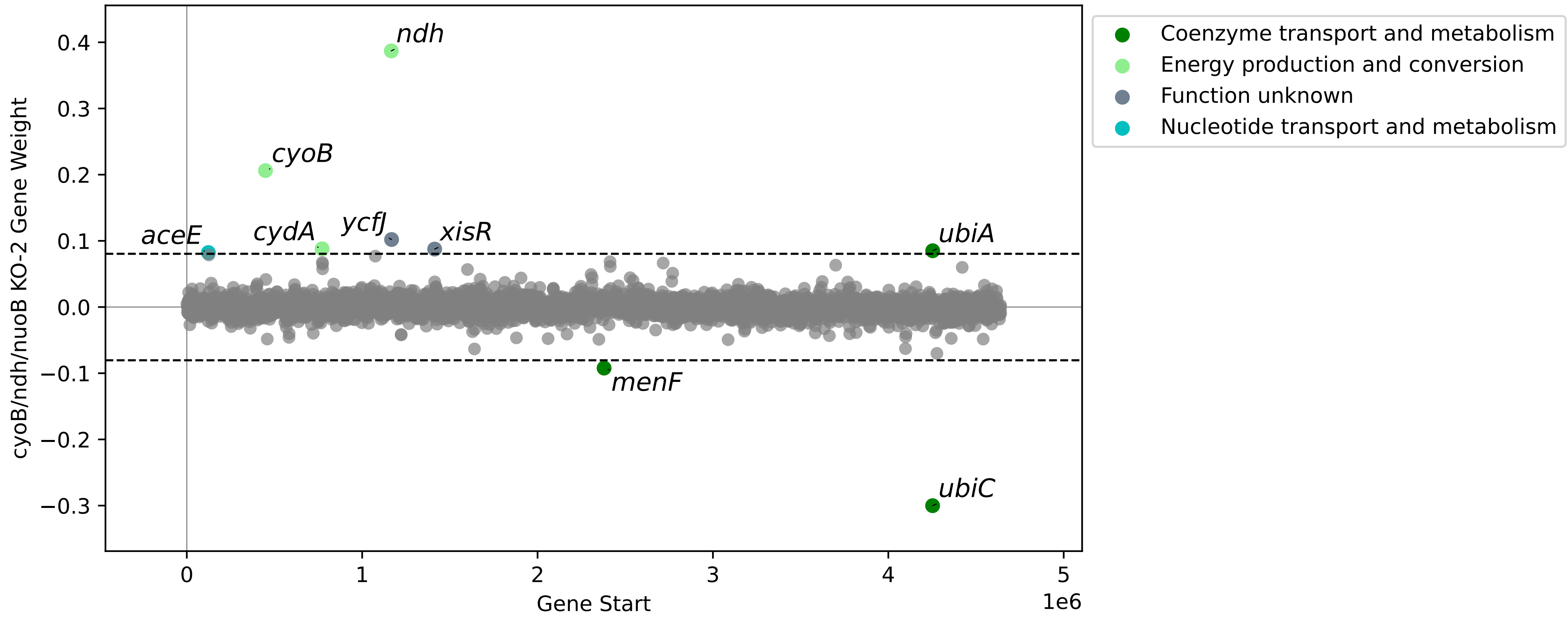

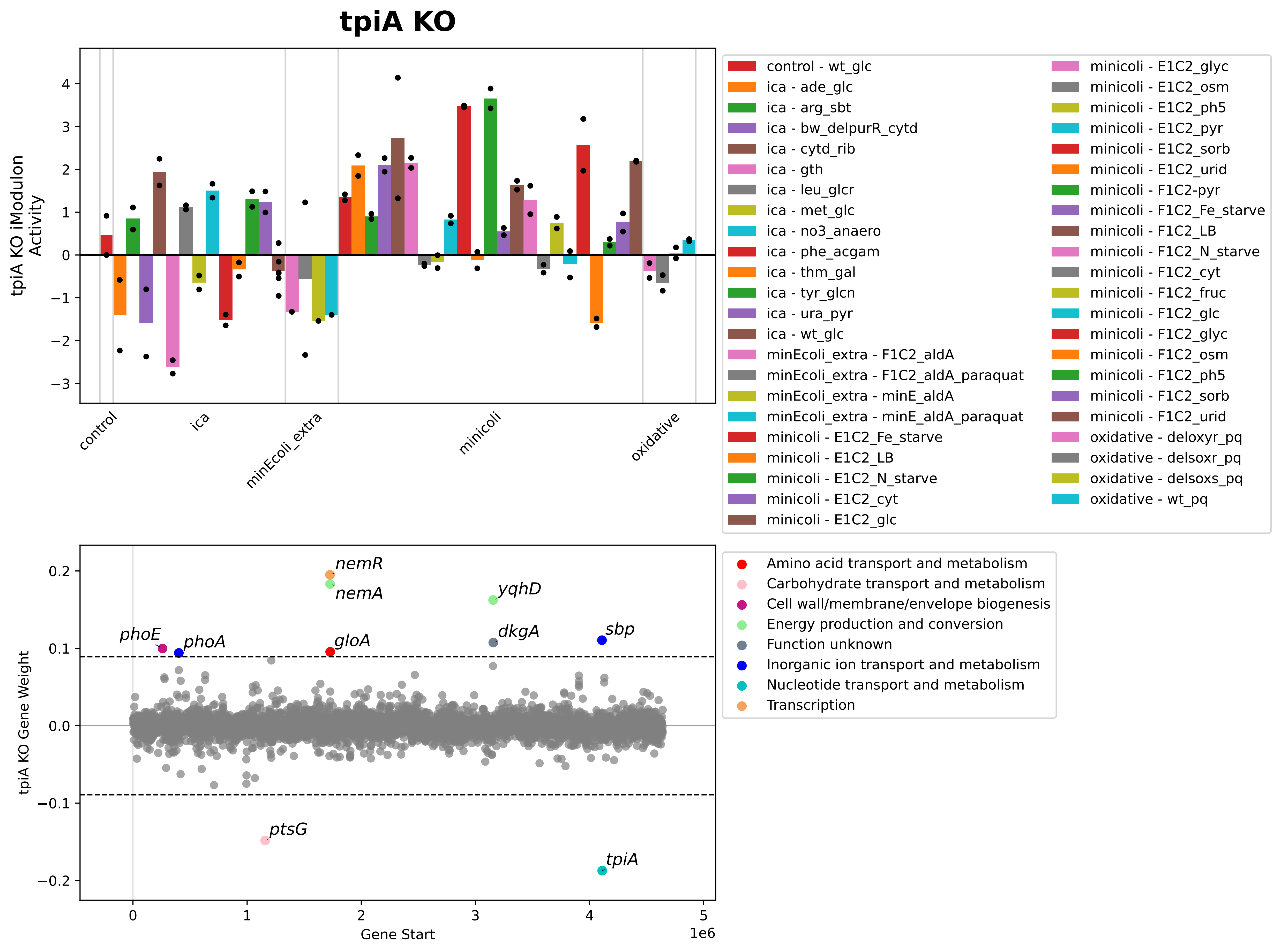

tpiA KO
tpiA KO iModulon Activity
control
ica
minEcoli_extra
minicoli
oxidative
control - wt_glc
ica - ade_glc
ica - arg_sbt
ica - bw_delpurR_cytd
ica - cytd_rib
ica - gth
ica - leu_glcr
ica - met_glc
ica - no3_anaero
ica - phe_acgam
ica - thm_gal
ica - tyr_glcn
ica - ura_pyr
ica - wt_glc
minEcoli_extra - F1C2_aldA
minEcoli_extra - F1C2_aldA_paraquat
minEcoli_extra - minE_aldA
minEcoli_extra - minE_aldA_paraquat
minicoli - E1C2_Fe_starve
minicoli - E1C2_LB
minicoli - E1C2_N_starve
minicoli - E1C2_cyt
minicoli - E1C2_glc
minicoli - E1C2_glyc
minicoli - E1C2_osm
minicoli - E1C2_ph5
minicoli - E1C2_pyr
minicoli - E1C2_sorb
minicoli - E1C2_urid
minicoli - F1C2-pyr
minicoli - F1C2_Fe_starve
minicoli - F1C2_LB
minicoli - F1C2_N_starve
minicoli - F1C2_cyt
minicoli - F1C2_fruc
minicoli - F1C2_glc
minicoli - F1C2_glyc
minicoli - F1C2_osm
minicoli - F1C2_ph5
minicoli - F1C2_sorb
minicoli - F1C2_urid
oxidative - deloxyr_pq
oxidative - delsoxr_pq
oxidative - delsoxs_pq
oxidative - wt_pq
tpiA KO Gene Weight
Gene Start
1e6
nemR
nemA
yqhD
phoE
phoA
gloA
dkgA
sbp
ptsG
tpiA
Amino acid transport and metabolism
Carbohydrate transport and metabolism
Cell wall/membrane/envelope biogenesis
Energy production and conversion
Function unknown
Inorganic ion transport and metabolism
Nucleotide transport and metabolism
Transcription

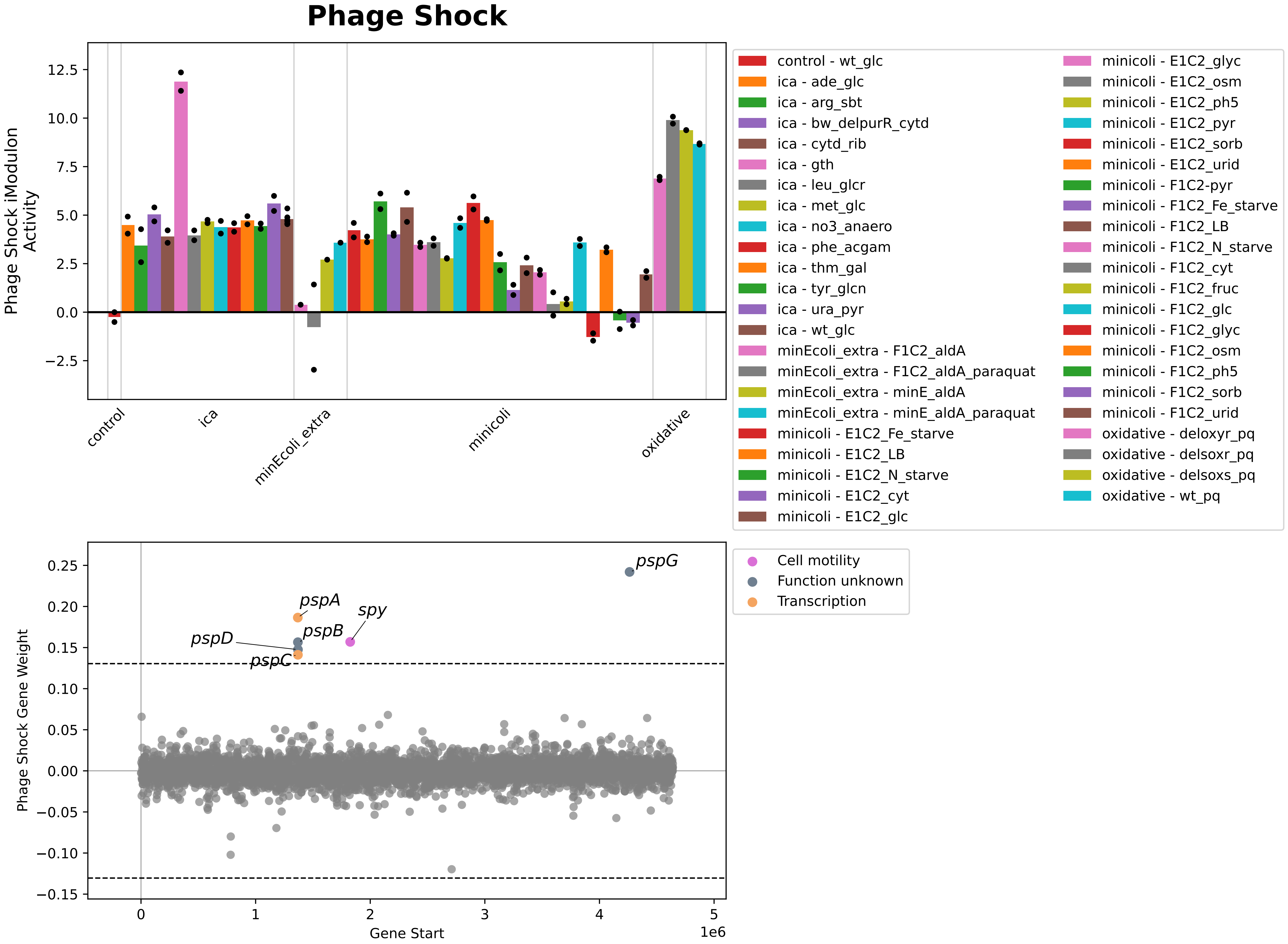

Phage Shock
Phage Shock iModulon Activity
12.5
10.0
7.5
5.0
2.5
0.0
−2.5
control
ica
minEcoli_extra
minicoli
oxidative
control - wt_glc
ica - ade_glc
ica - arg_sbt
ica - bw_delpurR_cytd
ica - cytd_rib
ica - gth
ica - leu_glcr
ica - met_glc
ica - no3_anaero
ica - phe_acgam
ica - thm_gal
ica - tyr_glcn
ica - ura_pyr
ica - wt_glc
minEcoli_extra - F1C2_aldA
minEcoli_extra - F1C2_aldA_paraquat
minEcoli_extra - minE_aldA
minEcoli_extra - minE_aldA_paraquat
minicoli - E1C2_Fe_starve
minicoli - E1C2_LB
minicoli - E1C2_N_starve
minicoli - E1C2_cyt
minicoli - E1C2_glc
minicoli - E1C2_glyc
minicoli - E1C2_osm
minicoli - E1C2_ph5
minicoli - E1C2_pyr
minicoli - E1C2_sorb
minicoli - E1C2_urid
minicoli - F1C2-pyr
minicoli - F1C2_Fe_starve
minicoli - F1C2_LB
minicoli - F1C2_N_starve
minicoli - F1C2_cyt
minicoli - F1C2_fruc
minicoli - F1C2_glc
minicoli - F1C2_glyc
minicoli - F1C2_osm
minicoli - F1C2_ph5
minicoli - F1C2_sorb
minicoli - F1C2_urid
oxidative - deloxyr_pq
oxidative - delsoxr_pq
oxidative - delsoxs_pq
oxidative - wt_pq
Phage Shock Gene Weight
0.25
0.20
0.15
0.10
0.05
0.00
−0.05
−0.10
−0.15
pspG
pspA
spy
pspB
pspD
pspC
0
1
2
3
4
5
Gene Start
1e6
Cell motility
Function unknown
Transcription

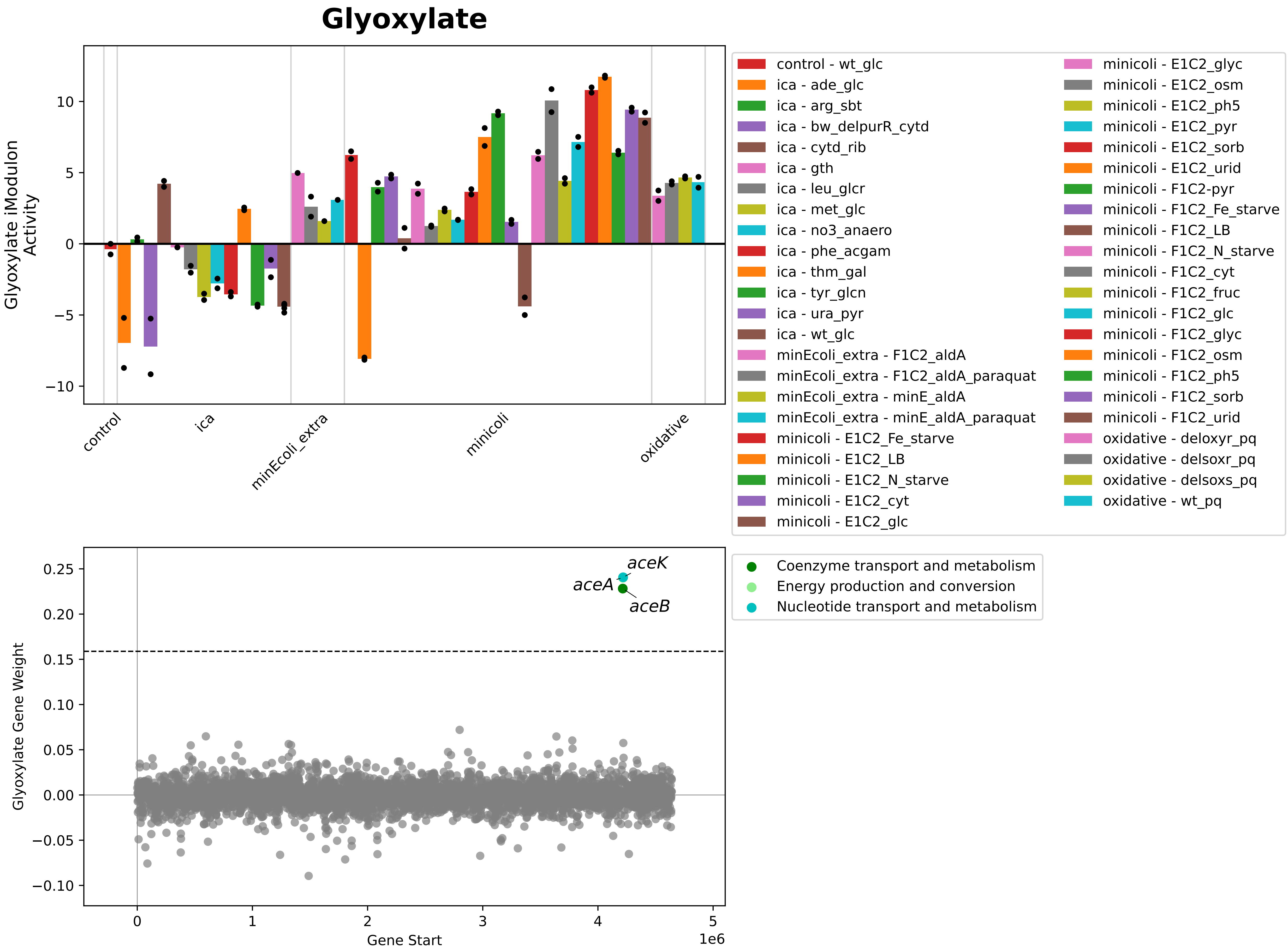
Glyoxylate
Glyoxylate iModulon Activity
control
ica
minEcoli_extra
minicoli
oxidative
control - wt_glc
ica - ade_glc
ica - arg_sbt
ica - bw_delpurR_cytd
ica - cytd_rib
ica - gth
ica - leu_glcr
ica - met_glc
ica - no3_anaero
ica - phe_acgam
ica - thm_gal
ica - tyr_glcn
ica - ura_pyr
ica - wt_glc
minEcoli_extra - F1C2_aldA
minEcoli_extra - F1C2_aldA_paraquat
minEcoli_extra - minE_aldA
minEcoli_extra - minE_aldA_paraquat
minicoli - E1C2_Fe_starve
minicoli - E1C2_LB
minicoli - E1C2_N_starve
minicoli - E1C2_cyt
minicoli - E1C2_glc
minicoli - E1C2_glyc
minicoli - E1C2_osm
minicoli - E1C2_ph5
minicoli - E1C2_pyr
minicoli - E1C2_sorb
minicoli - E1C2_urid
minicoli - F1C2-pyr
minicoli - F1C2_Fe_starve
minicoli - F1C2_LB
minicoli - F1C2_N_starve
minicoli - F1C2_cyt
minicoli - F1C2_fruc
minicoli - F1C2_glc
minicoli - F1C2_glyc
minicoli - F1C2_osm
minicoli - F1C2_ph5
minicoli - F1C2_sorb
minicoli - F1C2_urid
oxidative - deloxyr_pq
oxidative - delsoxr_pq
oxidative - delsoxs_pq
oxidative - wt_pq
aceK
aceA
aceB
Coenzyme transport and metabolism
Energy production and conversion
Nucleotide transport and metabolism
Glyoxylate Gene Weight
Gene Start
1e6

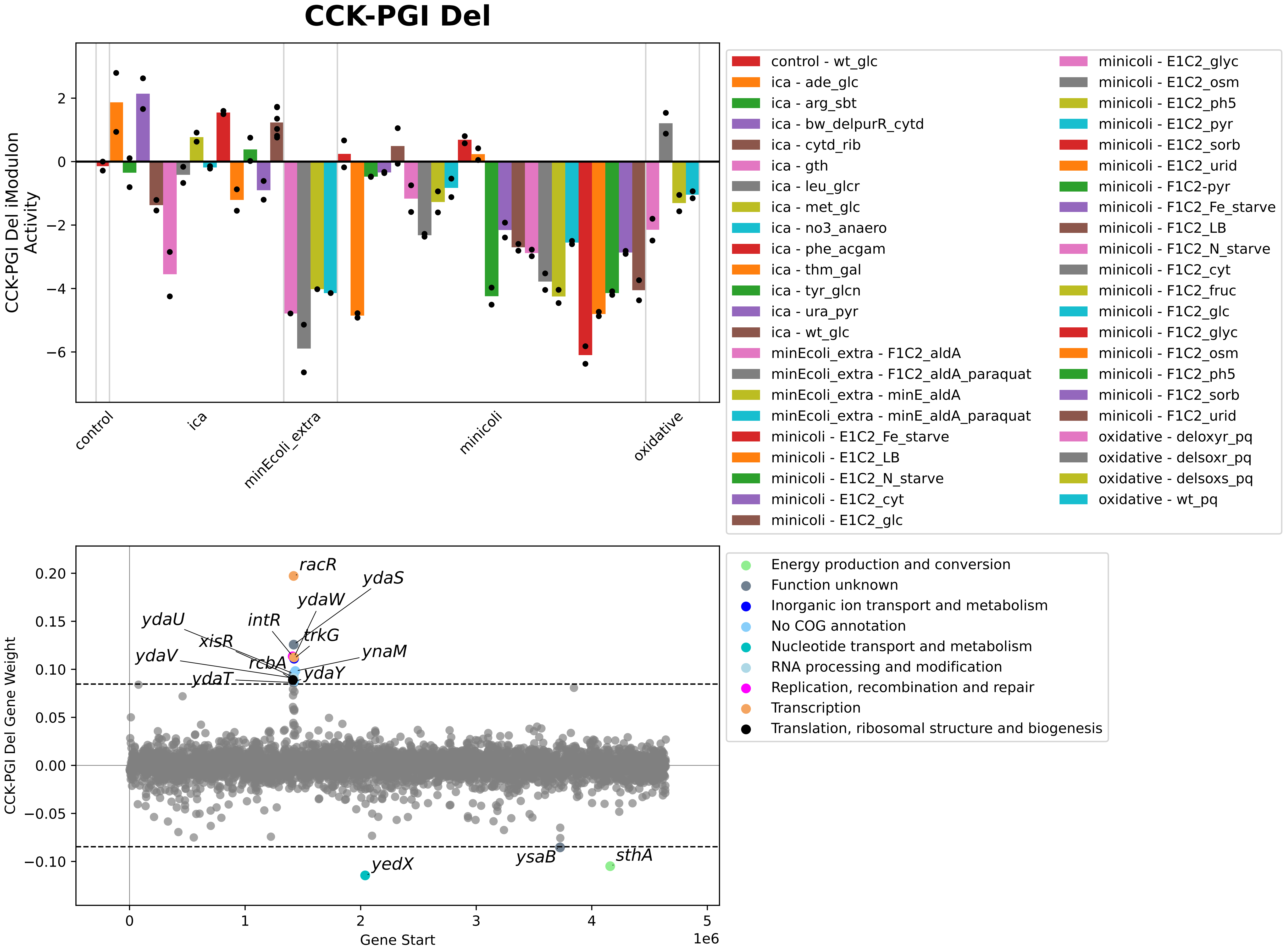

CCK-PGI Del
CCK-PGI Del iModulon Activity
control
ica
minEcoli_extra
minicoli
oxidative
control - wt_glc
ica - ade_glc
ica - arg_sbt
ica - bw_delpurR_cytd
ica - cytd_rib
ica - gth
ica - leu_glcr
ica - met_glc
ica - no3_anaero
ica - phe_acgam
ica - thm_gal
ica - tyr_glcn
ica - ura_pyr
ica - wt_glc
minEcoli_extra - F1C2_aldA
minEcoli_extra - F1C2_aldA_paraquat
minEcoli_extra - minE_aldA
minEcoli_extra - minE_aldA_paraquat
minicoli - E1C2_Fe_starve
minicoli - E1C2_LB
minicoli - E1C2_N_starve
minicoli - E1C2_cyt
minicoli - E1C2_glc
minicoli - E1C2_glyc
minicoli - E1C2_osm
minicoli - E1C2_ph5
minicoli - E1C2_pyr
minicoli - E1C2_sorb
minicoli - E1C2_urid
minicoli - F1C2-pyr
minicoli - F1C2_Fe_starve
minicoli - F1C2_LB
minicoli - F1C2_N_starve
minicoli - F1C2_cyt
minicoli - F1C2_fruc
minicoli - F1C2_glc
minicoli - F1C2_glyc
minicoli - F1C2_osm
minicoli - F1C2_ph5
minicoli - F1C2_sorb
minicoli - F1C2_urid
oxidative - deloxyr_pq
oxidative - delsoxr_pq
oxidative - delsoxs_pq
oxidative - wt_pq
CCK-PGI Del Gene Weight
Gene Start
1e6
racR
ydaS
ydaW
ydaU
intR
xisR
trkG
ynaM
ydaV
rcbA
ydaY
ydaT
yedX
ysaB
sthA
Energy production and conversion
Function unknown
Inorganic ion transport and metabolism
No COG annotation
Nucleotide transport and metabolism
RNA processing and modification
Replication, recombination and repair
Transcription
Translation, ribosomal structure and biogenesis

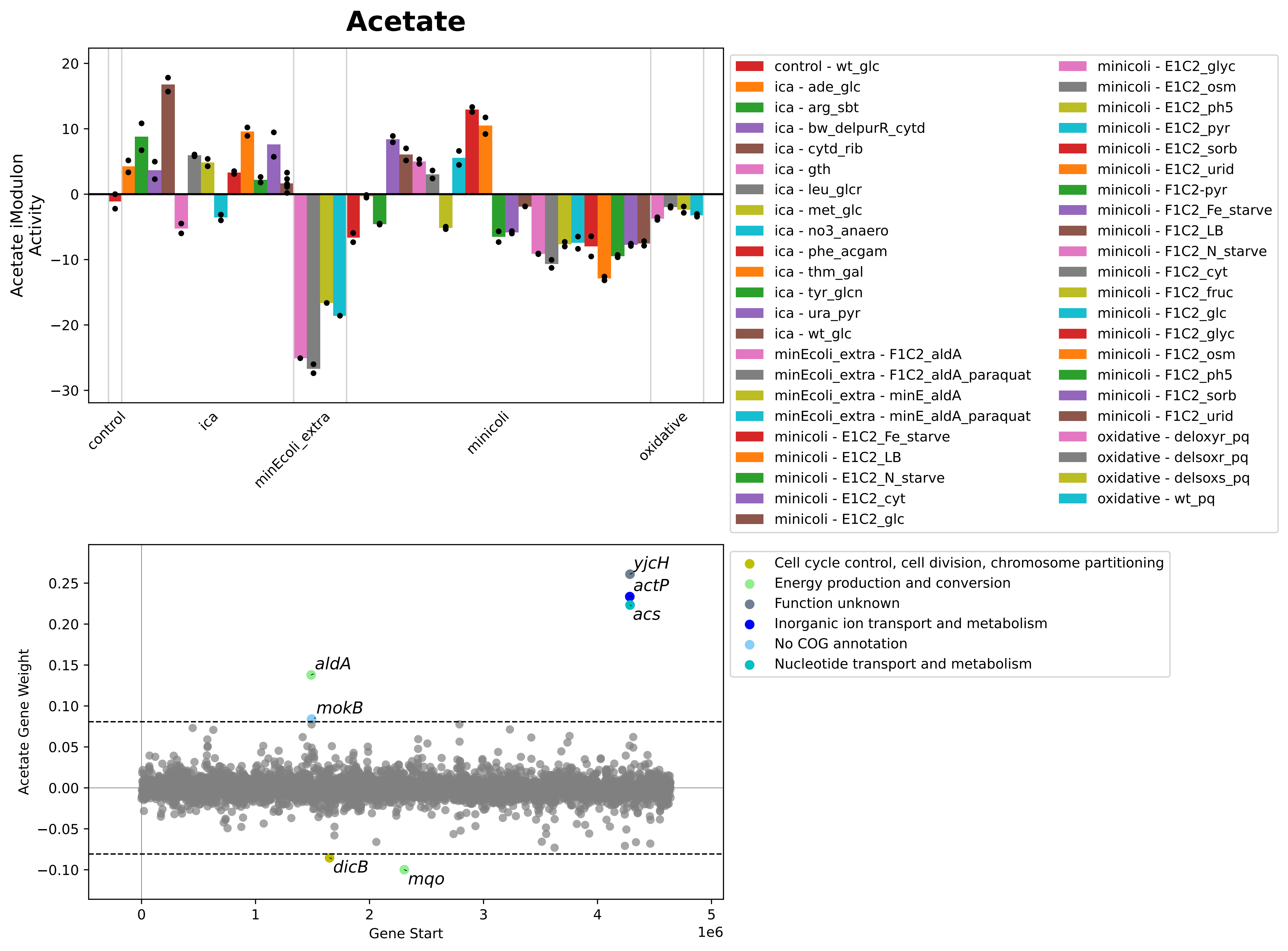

Acetate
Acetate iModulon Activity
20
10
0
−10
−20
−30
control
ica
minEcoli_extra
minicoli
oxidative
control - wt_glc
ica - ade_glc
ica - arg_sbt
ica - bw_delpurR_cytd
ica - cytd_rib
ica - gth
ica - leu_glcr
ica - met_glc
ica - no3_anaero
ica - phe_acgam
ica - thm_gal
ica - tyr_glcn
ica - ura_pyr
ica - wt_glc
minEcoli_extra - F1C2_aldA
minEcoli_extra - F1C2_aldA_paraquat
minEcoli_extra - minE_aldA
minEcoli_extra - minE_aldA_paraquat
minicoli - E1C2_Fe_starve
minicoli - E1C2_LB
minicoli - E1C2_N_starve
minicoli - E1C2_cyt
minicoli - E1C2_glc
minicoli - E1C2_glyc
minicoli - E1C2_osm
minicoli - E1C2_ph5
minicoli - E1C2_pyr
minicoli - E1C2_sorb
minicoli - E1C2_urid
minicoli - F1C2-pyr
minicoli - F1C2_Fe_starve
minicoli - F1C2_LB
minicoli - F1C2_N_starve
minicoli - F1C2_cyt
minicoli - F1C2_fruc
minicoli - F1C2_glc
minicoli - F1C2_glyc
minicoli - F1C2_osm
minicoli - F1C2_ph5
minicoli - F1C2_sorb
minicoli - F1C2_urid
oxidative - deloxyr_pq
oxidative - delsoxr_pq
oxidative - delsoxs_pq
oxidative - wt_pq
Acetate Gene Weight
0.25
0.20
0.15
0.10
0.05
0.00
−0.05
−0.10
yjcH
actP
acs
aldA
mokB
dicB
mqo
0
1
2
3
4
5
1e6
Gene Start
Cell cycle control, cell division, chromosome partitioning
Energy production and conversion
Function unknown
Inorganic ion transport and metabolism
No COG annotation
Nucleotide transport and metabolism

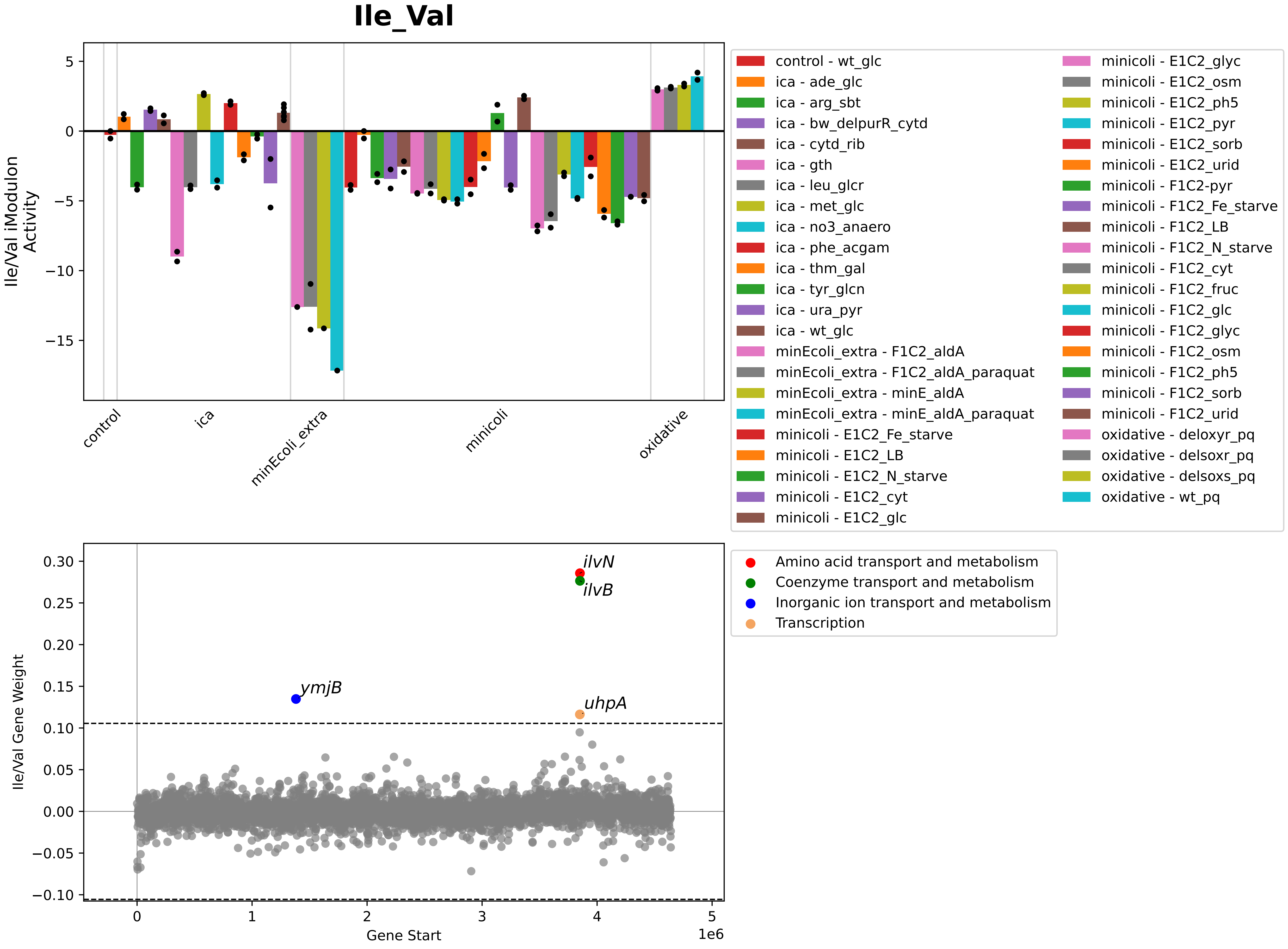
Ile_Val
Ile/Val iModulon Activity
control
ica
minEcoli_extra
minicoli
oxidative
control - wt_glc
ica - ade_glc
ica - arg_sbt
ica - bw_delpurR_cytd
ica - cytd_rib
ica - gth
ica - leu_glcr
ica - met_glc
ica - no3_anaero
ica - phe_acgam
ica - thm_gal
ica - tyr_glcn
ica - ura_pyr
ica - wt_glc
minEcoli_extra - F1C2_aldA
minEcoli_extra - F1C2_aldA_paraquat
minEcoli_extra - minE_aldA
minEcoli_extra - minE_aldA_paraquat
minicoli - E1C2_Fe_starve
minicoli - E1C2_LB
minicoli - E1C2_N_starve
minicoli - E1C2_cyt
minicoli - E1C2_glc
minicoli - E1C2_glyc
minicoli - E1C2_osm
minicoli - E1C2_ph5
minicoli - E1C2_pyr
minicoli - E1C2_sorb
minicoli - E1C2_urid
minicoli - F1C2-pyr
minicoli - F1C2_Fe_starve
minicoli - F1C2_LB
minicoli - F1C2_N_starve
minicoli - F1C2_cyt
minicoli - F1C2_fruc
minicoli - F1C2_glc
minicoli - F1C2_glyc
minicoli - F1C2_osm
minicoli - F1C2_ph5
minicoli - F1C2_sorb
minicoli - F1C2_urid
oxidative - deloxyr_pq
oxidative - delsoxr_pq
oxidative - delsoxs_pq
oxidative - wt_pq
ilvN
ilvB
ymjB
uhpA
Ile/Val Gene Weight
Gene Start
1e6
Amino acid transport and metabolism
Coenzyme transport and metabolism
Inorganic ion transport and metabolism
Transcription

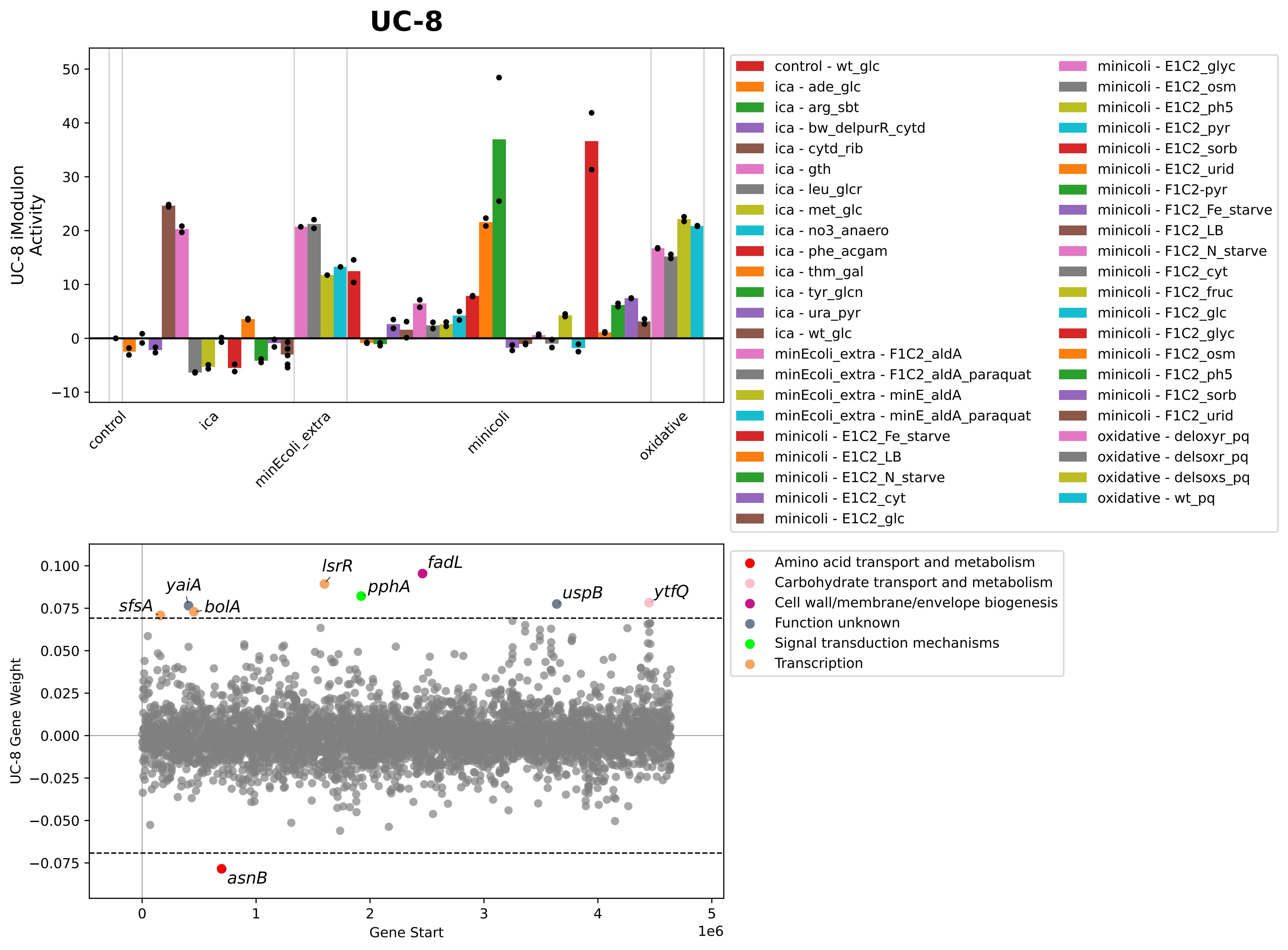
UC-8
UC-8 iModulon Activity
50
40
30
20
10
0
−10
control
ica
minEcoli_extra
minicoli
oxidative
control - wt_glc
ica - ade_glc
ica - arg_sbt
ica - bw_delpurR_cytd
ica - cytd_rib
ica - gth
ica - leu_glcr
ica - met_glc
ica - no3_anaero
ica - phe_acgam
ica - thm_gal
ica - tyr_glcn
ica - ura_pyr
ica - wt_glc
minEcoli_extra - F1C2_aldA
minEcoli_extra - F1C2_aldA_paraquat
minEcoli_extra - minE_aldA
minEcoli_extra - minE_aldA_paraquat
minicoli - E1C2_Fe_starve
minicoli - E1C2_LB
minicoli - E1C2_N_starve
minicoli - E1C2_cyt
minicoli - E1C2_glc
minicoli - E1C2_glyc
minicoli - E1C2_osm
minicoli - E1C2_ph5
minicoli - E1C2_pyr
minicoli - E1C2_sorb
minicoli - E1C2_urid
minicoli - F1C2-pyr
minicoli - F1C2_Fe_starve
minicoli - F1C2_LB
minicoli - F1C2_N_starve
minicoli - F1C2_cyt
minicoli - F1C2_fruc
minicoli - F1C2_glc
minicoli - F1C2_glyc
minicoli - F1C2_osm
minicoli - F1C2_ph5
minicoli - F1C2_sorb
minicoli - F1C2_urid
oxidative - deloxyr_pq
oxidative - delsoxr_pq
oxidative - delsoxs_pq
oxidative - wt_pq
UC-8 Gene Weight
0.100
0.075
0.050
0.025
0.000
−0.025
−0.050
−0.075
sfsA
yaiA
bolA
lsrR
pphA
fadL
uspB
ytfQ
asnB
0
1
2
3
4
5
1e6
Gene Start
Amino acid transport and metabolism
Carbohydrate transport and metabolism
Cell wall/membrane/envelope biogenesis
Function unknown
Signal transduction mechanisms
Transcription
